# Integrated analysis of long non-coding RNAs in human colorectal cancer

**DOI:** 10.18632/oncotarget.8192

**Published:** 2016-03-19

**Authors:** Xiaohua Chen, Binjian Liu, Rui Yang, Yong Guo, Feng Li, Lin Wang, Hanyang Hu

**Affiliations:** ^1^ School of Basic Medical Sciences, Wuhan University, Wuhan, China; ^2^ Department of Laboratory Medicine, No.161 Hospital of PLA, Wuhan, China; ^3^ Department of General Surgery, No.161 Hospital of PLA, Wuhan, China; ^4^ Department of Pathology, No.161 Hospital of PLA, Wuhan, China

**Keywords:** lncRNA, colorectal cancer, expression profiling, epigenetic regulation, poor-prognosis

## Abstract

Accumulating evidence highlights the role of long non-coding RNAs (lncRNAs) in tumors. However, the genome-wide expression and roles of lncRNAs in colorectal cancer (CRC) remain unknown. Here, we systematically examined the global gene expressions in primary and synchronous liver metastases CRC tissue, in which thousands of aberrantly expressed lncRNAs were characterized. Co-expression analysis revealed that some lncRNAs correlated to their neighboring mRNAs in expression levels, whereas others formed networks with protein-coding genes *in trans*. We observed H3K4me3 was enriched at expressed lncRNA transcription start sites (TSSs) and correlated to dysregulated lncRNAs. Furthermore, we identified primary and metastasis tumor linked lncRNA signatures positively correlated with poor-prognosis gene set. Finally, functional experiments demonstrated two candidate lncRNAs were required for proliferation and migration of CRC cells. In summary, we provided a new framework for lncRNA associated clinical prognosis evaluation and target selection of gene therapy in CRC.

## INTRODUCTION

Colorectal cancer (CRC) is the third most common cancer and the fourth leading cause of cancer related death worldwide, which has been a great threat to public health. To date, researches focused on the deregulation of protein-coding genes to identify oncogenes and tumor suppressors that serve as diagnostic and therapeutic targets [1] and/or genetic variations that serve as susceptibility loci [2] largely extended our knowledge to CRC pathogenesis. Additionally, studies on transcriptional regulatory networks [3] and epigenetics such as microRNAs [4, 5], DNA methylation [6] and enhancer elements [7] in CRC have emerged.

Recent advances in next generation sequencing (NGS) technologies allowed to comprehensively study the human transcriptome, thereby identified a new class of RNA called long non-coding RNA (lncRNA). lncRNA is longer than 200 nt and has little or no open reading frame (ORF). lncRNAs can be divided into five broad categories: sense, antisense, bidirectional, intronic and intergenic, according to the proximity between neighboring transcripts [8]. Accumulating evidence suggested that thousands of lncRNAs existed and exhibited highly tissue and cell-type specific manner [9]. Despite do not encode protein, lncRNAs have been proved to be involved in diverse physiological and pathological processes, such as cell growth, apoptosis, stem cell pluripotency, development and cancer biology by acting as transcriptional, posttranscriptional, or epigenetic regulators [10, 11]. LncRNA landscape has been depicted in several types of cancer by RNA-seq, such as lung cancer [12], prostate cancer [13], neuroblastoma [14], T-ALL [15], breast cancer [16] and endometrial cancer [17]. A panel of tumor-type specific differentially expressed lncRNAs are identified and some of which have been functionally verified to contribute to tumorgenesis or serve as putative diagnostic markers.

Initial studies in CRC have identified nearly 20 individual aberrantly expressed lncRNAs, many of which function as oncogenes or tumor suppressors by participating in critical signaling pathways such as MYC, WNT, and TP53 [18]. Of these lncRNAs, for example, the upregulated *E2F4* antisense in CRC was induced by WNT/beta-catenin signaling, which resulted in *E2F4* downregualtion [19]; *LOC285194/TUSC7* was found with lower expression in tumor and functioned as TP53-regulated tumor suppressor that inhibited cell growth through the repression of miR-211 [20, 21]. Recently, a genome-wide study of CRC lncRNAs based on microarray has obtained 762 aberrantly expressed lncRNAs [22]. However, the lack of large scale of functional annotation made the roles of the vast majority of those lncRNAs remain unclear. In addition, lncRNA expression pattern linked to liver metastasis, which is the most frequent site of metastases from CRC [23], has not yet been investigated.

To investigate the potential role of lncRNA in carcinogenesis and liver metastasis in a more comprehensive way, we systematically analyzed public RNA-seq data from patients with matched primary tumor, synchronous liver metastases and normal colon tissues. We identified thousands of differently expressed lncRNAs as well as mRNAs associated with primary and metastasis tumor. Functional predictions with advanced computational approaches revealed that some *cis*-lncRNAs were co-regulated with their neighboring mRNAs and implicated in several critical CRC signaling pathways, whereas other lncRNAs acted *in trans* by forming networks with protein-coding genes contributed to tumor development and progression. Additionally, we demonstrated the epigenetic control of lncRNA transcriptions by observing the enrichment of histone marks at the lncRNA TSSs loci. Furthermore, we identified primary cancer related 33-lncRNA and metastasis cancer related 46-lncRNA signatures positively correlated with a previously defined poor-prognosis gene set. Finally, functional experiments demonstrated that inhibition of two candidate lncRNAs, *LOC100190940* and *TCONS_l2_00022545*, significantly decreased the proliferation and migration of CRC cells. In summary, we have systematically characterized lncRNAs in CRC and provided evidence to support lncRNAs as key regulators in carcinogenesis and metastasis. All these results revealed these lncRNAs could serve as potential diagnostic biomarkers or therapeutic targets in patients with CRC.

## RESULTS

### lncRNA landscape in primary and metastasis CRC

**Figure 1:**
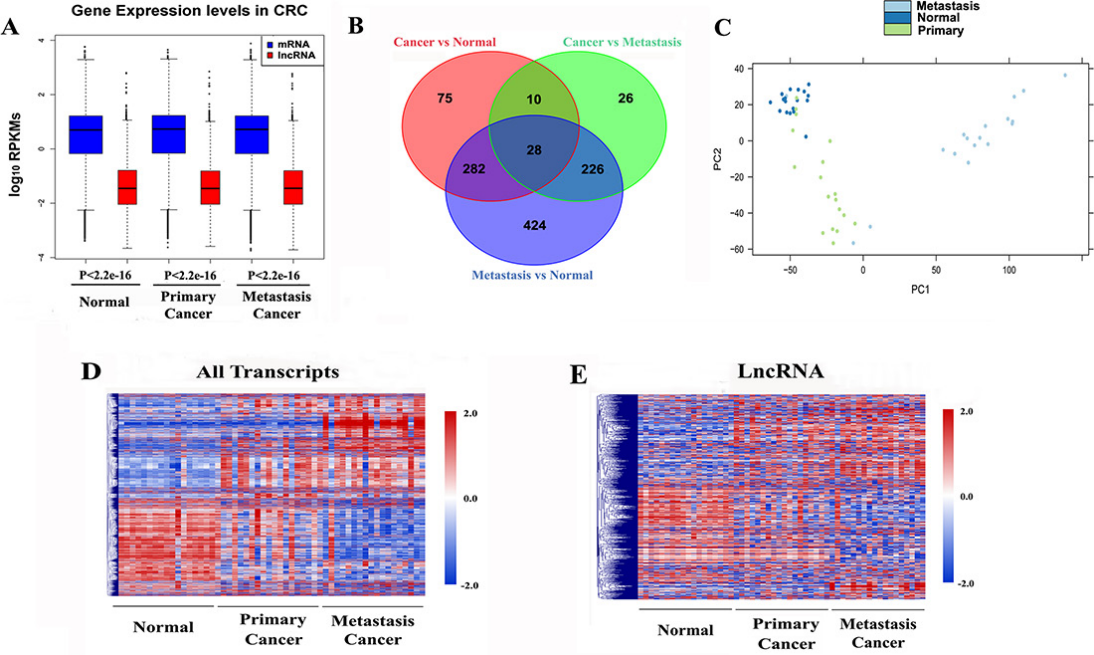
Global transcriptomic patterns in CRC (**A**) Boxplots of log_10_-transformed (RPKM) gene expression values for all transcribed lncRNA and mRNA in each group. *P* values were determined by Wilcoxon rank sum test with continuity correction. (**B**) Venn diagrams showing differentially expressed mRNAs and lncRNAs in CRC. (**C**) Principal components analyses of primary tumors (*n* = 18), metastasis tumors (*n* = 18) and normal colon tissues (*n* = 18). (**D**) Hierarchical clustering of all differentially expressed transcripts expression. (**E**) Hierarchical clustering of differentially expressed long non-coding gene expression.

To comprehensively analyze the lncRNA map in CRC, we first set out to characterize the global transcriptional alterations between CRC samples. We compared the transcriptomic patterns using RNA-seq data [24] derived from 54 samples containing 18 matched primary (PC), metastasis (MC) and non-tumor (NC) tissue. We generated the custom reference gene annotation file from UCSC, in which 18,870 protein-coding genes and 10,044 lncRNAs were included (see Supplementary Material). In total, we found 1,272 lncRNAs with RPKM > 1 out of the 2,954 expressed lncRNAs (RPKM ≥ 0.3, a detectable expression cutoff for lncRNAs [25]) in at least one sample, suggesting that these lncRNAs were actively expressed in cancerous and/or normal colonic tissue and might be involved in normal colon function. Additionally, lncRNAs displayed lower expression level relative to protein-coding genes both in cancerous and normal colon (*P* < 2.2e-16) (Figure 1A), consistent with previous reports that lncRNA was less actively transcribed than mRNA [9, 15].

To further investigate the gene expression changes between distinct cancer status, we performed differentially expression analysis upon the three expression profiles. Overall, 2,019 protein-coding genes and 395 lncRNAs were detected as differentially expressed (DE) between primary tumor and normal samples (FDR < 0.05 and fold change ≥ 2). Meanwhile, 1,655 DE protein-coding genes and 290 DE lncRNAs were detected between primary and metastasis tumor samples, and 4,108 DE protein-coding genes and 960 DE lncRNAs were detected between metastasis tumor and normal samples, respectively (Figure 1B and Supplementary Table S1). Both principal components analysis (PCA) (Figure 1C) and unsupervised hierarchical clustering of these DE protein-coding genes and lncRNAs revealed cancer stage-specific expression patterns (Figure 1D–1E). Specially, we performed unsupervised hierarchical clustering on the DE lncRNAs exclusively to investigate their expression pattern. As expected, our DE lncRNA expression profile exhibited distinct patterns corresponding to normal, primary and metastasis cancer samples, respectively (Figure 1E). In agreement with previous observations that lncRNAs exhibited greater tissue specificity in expression than mRNAs [9], our results suggested that lncRNAs could also show disease stage specific expression patterns compared to protein-coding genes in CRC.

### Functional characterization of the identified DE lncRNAs

**Figure 2:**
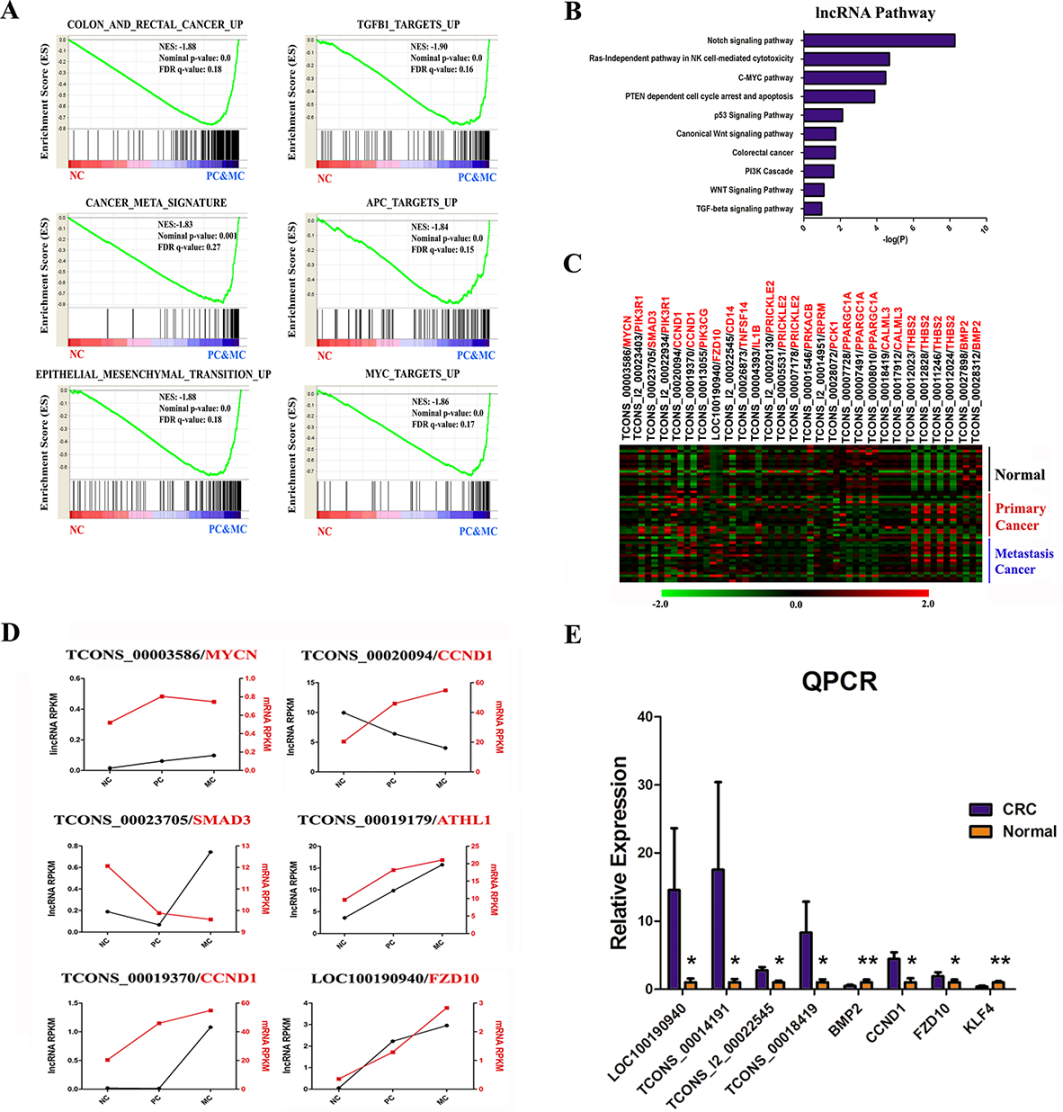
Functional interpretation of differentially expressed coding and long non-coding genes (**A**) Gene set enrichment analysis delineates biological pathways for altered protein coding genes. (**B**) Enriched KEGG pathways of differentially expressed lncRNAs. (**C**) Heatmap of 42 pairs of DE lncRNAs and their nearest DE mRNAs. (**D**) Examples of expression of co-regulated lncRNA-mRNA partners (black is lncRNA, red is mRNA). (**E**) qPCR validation of the RNA-seq data. Replicates (*n* = 6) of each sample were run and the Ct values averaged. All Ct values were normalized to β-Actin.

To verify the fidelity of our differentially expressed transcripts, we first compared them with the TCGA study. Of the total of 1,675 DE protein-coding genes between tumor and normal samples in TCGA, 730 were overlapped with our PC-NC profile, and 451 were overlapped with PC-MC or MC-NC profiles. Next, GSEA analysis revealed that genes dysregulated either in PC or MC were enriched in colorectal cancer signatures and critical singling pathways (Figure 2A). Therefore, our gene expression profiles seem to be accurate and robust.

We next explored if DE lncRNAs have functional roles in tumorgenesis or metastasis. By determining the signaling pathways of the protein-coding genes flanking DE lncRNA loci, we found the neighboring protein-coding genes were enriched in several critical pathways (Figure 2B). These observations suggested that some of the DE lncRNAs might be members of those signaling pathways and promoted cancer development. Additionally, we scanned each DE lncRNA and examined if its nearest mRNA was also differentially expressed. After expression level filtering, we collected 31 DE lncRNA-mRNA pairs to interrogate if they were co-regulated (Figure 2C). As expected, we found 6 lncRNA-mRNA *cis* pairs in which both the DE lncRNA and mRNA were regulated in at least two types of samples (Figure 2D). We validated the expressions of eight DE lncRNAs and mRNAs in our 6 matched CRC samples by qPCR experiment (Figure 2E), which were consistent with our RNA-seq results.

### Network-based prediction of lncRNA functions

**Figure 3:**
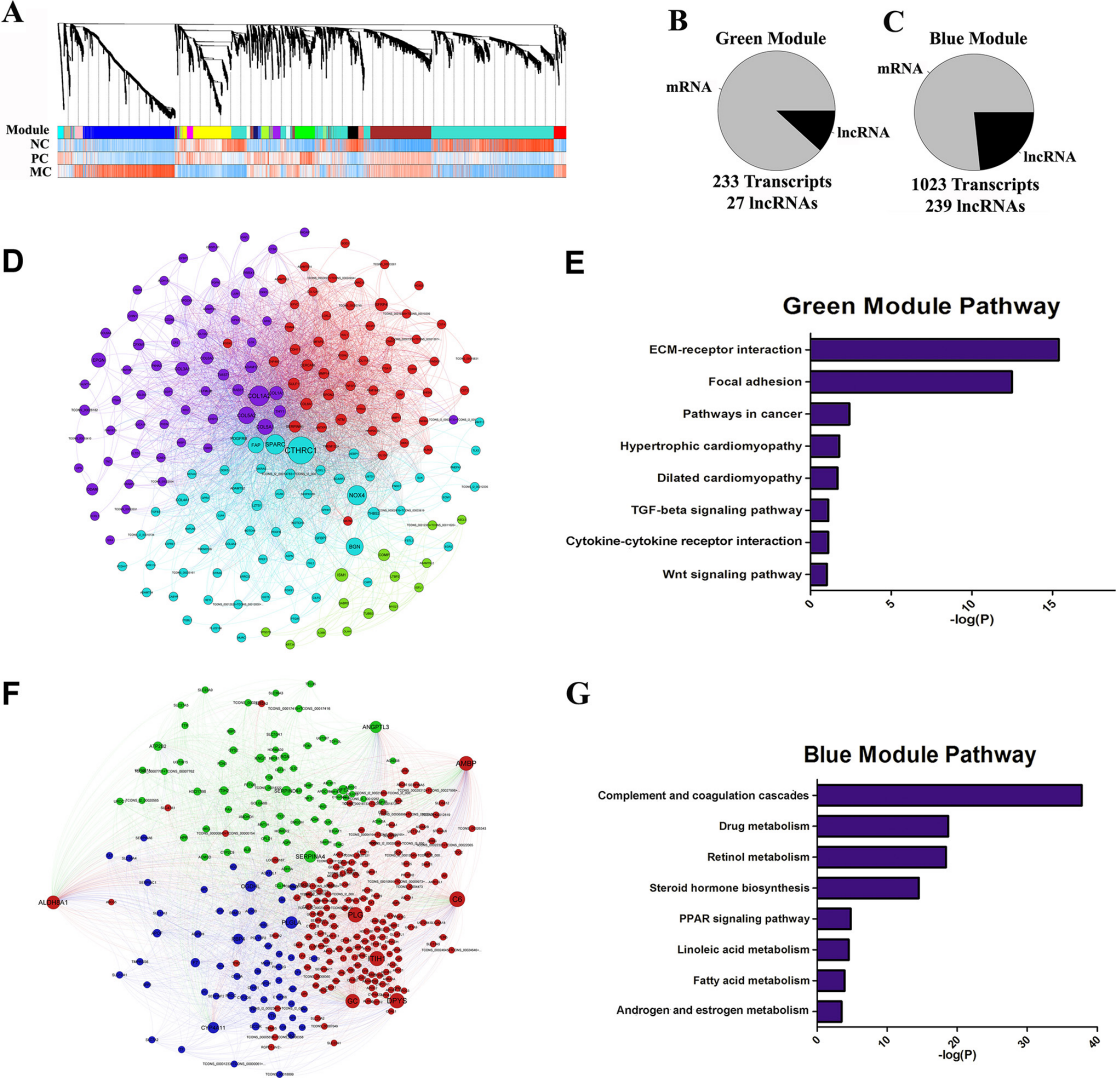
Network analysis of coding and long non-coding gene expression in CRC (**A**) Relationships between network modules and traits. Upper panel, dendrograms produced by average linkage hierarchical clustering of genes on the basis of topological overlap. Modules of co-expressed genes were assigned colors as indicated by the horizontal bar beneath each dendrogram. Modules from different networks with significant overlap (corrected hypergeometric *P* < 0.05) were assigned the same color. Lower leaves indicate greater similarity of transcript expression profiles within that module. Lower panel (three correlation bands), NC, PC and MC bands show correlations (cor.) to the corresponding modules. Positive (red) and negative (blue). (**B**–**C**) Pie charts indicating the abundance of lncRNAs within green and blue modules. Module members are defined as all transcripts that were positively correlated with the module eigengene. (**D**) The co-expression network of green module. (**E**) KEGG pathway enrichment annotations of green module. (**F**) The co-expression network of blue module. (**G**) KEGG pathway enrichment annotations of blue module.

As some lncRNAs function in alternative ways other than *cis*-regulation, we performed weighted correlation network analysis in order to identify highly interconnected genes. We constructed co-expression transcripts network comprising both mRNAs and lncRNAs, yielding a total of 20 modules, which then were ultimately merged based on module similarity (Figure 3A). These modules were quantitatively correlated to three traits: normal, primary and metastasis cancer. We found the green module was most positively correlated with primary cancer with 206 mRNAs and 27 lncRNAs (*P* = 0.001) (Figure 3B and Supplementary Table S2). In contrast, transcripts in the blue module were most positively correlated with metastasis cancer with 784 mRNAs and 239 lncRNAs (*P* = 5e-10) (Figure 3C and Supplementary Table S3). Thus, the subnetworks based on the transcripts from the modules corresponding to different traits were constructed, respectively.

Pathway analysis for primary cancer correlated green module subnetwork (Figure 3D) revealed that members in this module were highly enriched in the activated pathways such as ECM-receptor, TGF-β and WNT (Figure 3E). It therefore can be inferred that lncRNAs in the same subnetwork might be functionally related to those pathways as well. Likewise, members in the metastasis cancer correlated blue module subnetwork (Figure 3F) were significantly enriched in several cancer related metabolic pathways (Figure 3G), suggesting these lncRNAs in this module have roles in remodeling abnormal cellular metabolism in cancer cells.

Additionally, we found the turquoise module was correlated with normal samples. This module contained 1,475 transcripts, including 1,108 mRNAs and 307 lncRNAs. Pathway analysis revealed members in this module were highly enriched in Cytokine-cytokine receptor interaction, Neuroactive ligand-receptor interaction, Hedgehog signaling pathway and Intestinal immune network for IgA production.

### Epigenetic regulation of dysregulated lncRNAs in CRC

**Figure 4:**
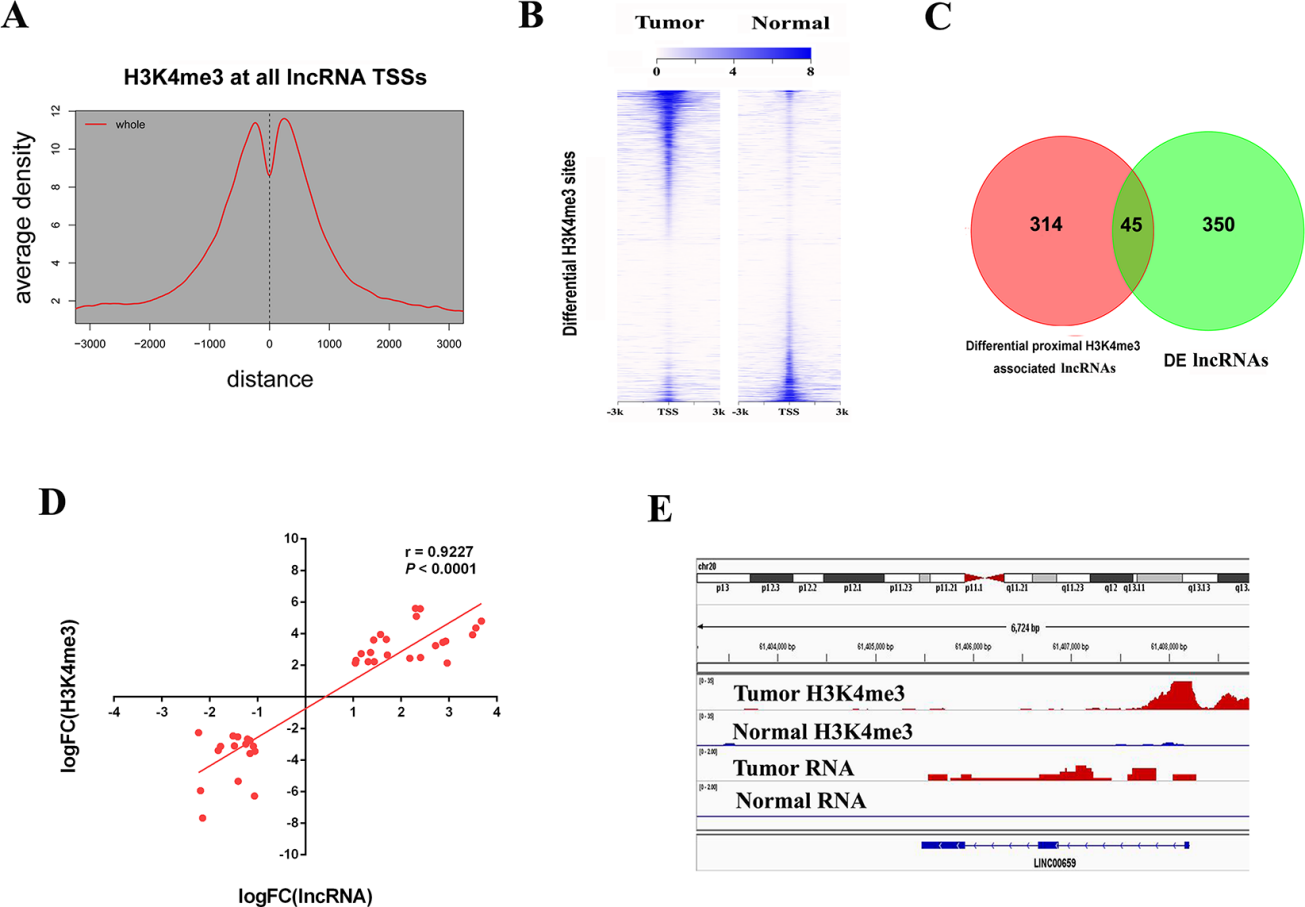
H3K4me3 modifications at lncRNA loci in CRC (**A**) Aggregate plots of H3K4me3 ChIP signal at TSSs ± 3 kb of lncRNAs in primary tumor. (**B**) Heatmap representation of differential ChIP signal for H3K4me3 centered on peak midpoint ± 3 kb in primary tumor and normal tissue. (**C**) Venn diagrams showing overlap between differential H3K4me3 sites linked lncRNAs and differentially expressed lncRNAs in CRC. (**D**) Correlations between differential H3K4me3 and target lncRNA expressions. (**E**) ChIP signal for H3K4me3 and RNA signal on tumor highly expressed lncRNA *LINC00659* TSS loci.

Previous work have shown that the enrichment of H3K4me3 at the TSS of lncRNA loci in embryonic stem cell, T-ALL, prostate cancer and lung cancer [12, 13, 15, 26]. To test the presence of histone modifications at expressed lncRNA loci, we analyzed H3K4me3 ChIP-seq data in CRC patient samples and identified 21,889 peaks. We observed enrichment of H3K4me3 signal at those lncRNA TSSs (Figure 4A), indicating that lncRNAs possessed actively regulated promoters in CRC.

Further, we tested the correlations between histone modifications and transcriptions. We performed differential modification analysis and identified 3,721 gained (up-H3K4me3) and 3,224 loss (down-H3K4me3) regions in cancer (FDR < 0.05, fold-change > 4) compared with normal samples (Figure 4B). By overlapping these regions with 395 DE lncRNAs between primary tumor and normal samples, 45 differential region-lncRNA pairs were found (Figure 4C and Supplementary Table S4), all of which displayed consistent direction in that gained H3K4me3 regions correlated with up-regulated lncRNAs whereas loss H3K4me3 regions correlated with down-regulated lncRNAs (*r* = 0.9227, *P* < 0.0001) (Figure 4D). As is shown in Figure 4E, *LINC00659* is highly expressed in primary tumor with higher H3K4me3 signal at its promoter region compared with normal samples. Thus, these results indicate that epigenetic mechanism is critical for lncRNA transcription in CRC.

### Identification of poor-prognosis associated lncRNAs in CRC

To evaluate the clinical significance of our discovered lncRNAs, we next set out to determine the expression relationships between lncRNAs and three recent CRC classification systems, named the Colon Cancer Subtype (CCS) system, the Colorectal Cancer Assigner (CRCA) and the Colon Cancer Molecular Subtype (CCMS) system. In these classification systems, poor-prognosis molecular subtypes were defined as CCS3, CRCA5 and CCMS4, respectively [27–29]. We collected and merged the gene classifiers from the three subtypes and obtained a 381-gene signature that were correlated with decreased disease-free survival intervals after surgery.

**Figure 5:**
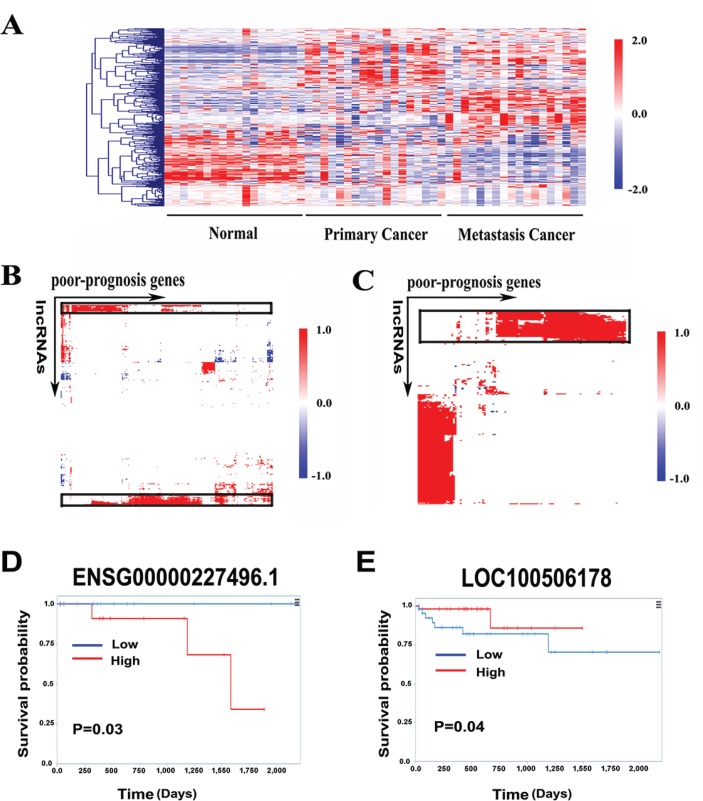
Identification of poor-prognosis gene signatures associated lncRNAs (**A**) Heatmap of 381 poor-prognosis genes expression pattern in RNA-seq data set. (**B**) A hierarchically clustered heatmap of the correlation between 381 poor-prognosis genes and 395 primary cancer associated DE lncRNAs. (**C**) A hierarchically clustered heatmap of the correlation between 122 poor-prognosis genes and 290 metastasis cancer associated DE lncRNAs. Red color indicates positive correlation, blue color indicates negative correlation and white represents no correlation. (**D**) Kaplan-Meier curves for overall survival time in patients with CRC according to expression of *ENSG00000227496.1*. (**E**) Kaplan-Meier curves for overall survival time in patients with CRC according to expression of *LOC100506178*.

We first explored the expression patterns of the 381-gene in our RNA-seq data. As a result, they successfully distinguished the primary, metastasis and normal samples (Figure 5A). We then clustered the primary cancer associated DE lncRNAs and protein-coding genes into sets with correlated expression patterns by constructing a matrix of the association of each lncRNA with each of the 381 genes using the 54 samples (Figure 5B). This analysis revealed a set of 33-lncRNA positively correlated with poor-prognosis gene signatures (Supplementary Table S5).

Furthermore, in the classification systems, patients classified as CCS3 metastasized more frequently; CCMS4 tumors showed enrichment for metastatic than other subtypes, indicating that 381-gene signature had the possibility to detect patients who tend to develop colorectal liver metastasis at early stages, e.g., Dukes stage B. We observed that a fraction of the 381 genes were up-regulated in metastatic tumor (Figure 5A). Combined with our primary-metastatic profile, 122 genes were significantly expressed. Similarly, we clustered the 290 metastatic cancer associated DE lncRNAs and those 122-gene signature into sets with correlated expression patterns across the 54 samples (Figure 5C). This analysis generated a set of 46-lncRNA positively associated with poor-prognosis gene signatures (Supplementary Table S6). We then examined the prognostic value of those lncRNA signatures in a recently developed lncRNAs exploration platform called TANRIC [30]. Higher expression of primary tumor upregulated lncRNA from the 33-lncRNA signatures, *ENSG00000227496.1* (*TCONS_00001306+TCONS_00001307+TCONS_00001308+TCONS_00002241*), was significantly correlated with poor overall survival of the patients (Figure 5D). In contrast, lower expression of metastatic tumor down-regulated lncRNA from the 46-lncRNA signatures, *LOC100506178*, was significantly correlated with poor overall survival of the patients (Figure 5E). Taken together, these results suggested that lncRNAs highly correlated with poor-prognosis genes had predictive and prognostic value in the management of CRC.

### Functional analysis of two dysregulated lncRNAs

**Figure 6:**
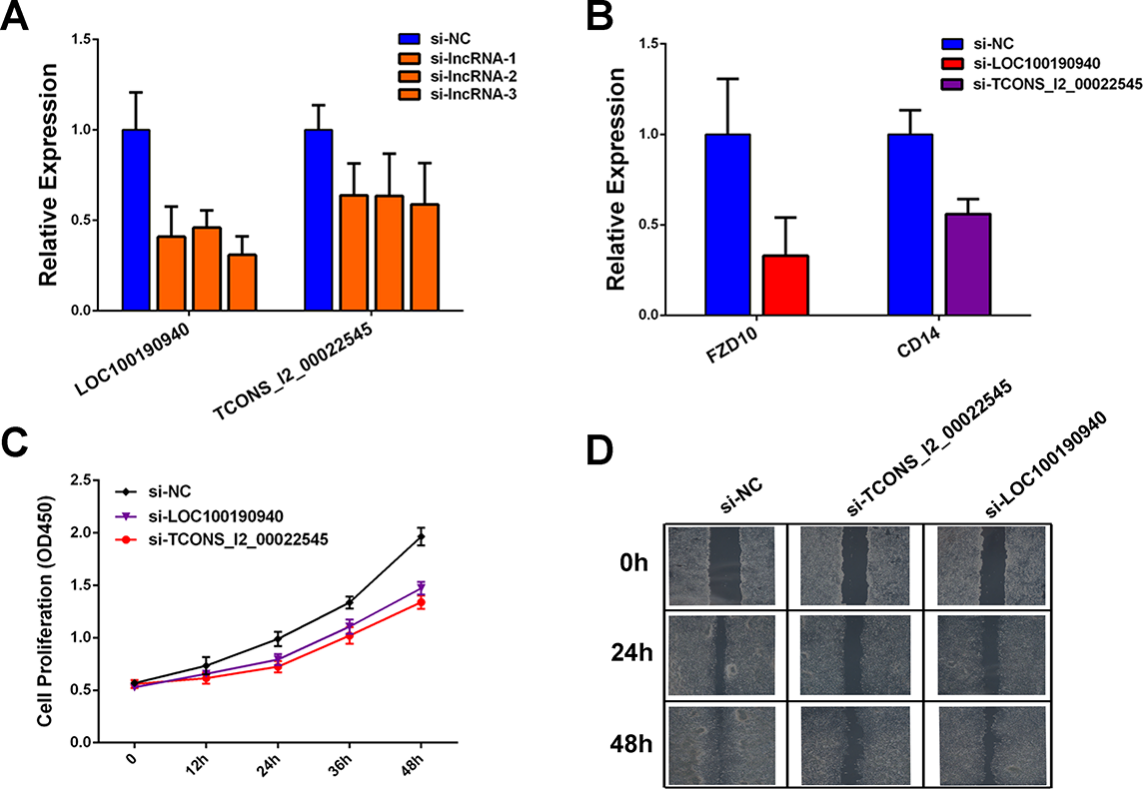
Functional analysis of candidate dysregulated lncRNAs (**A**) Relative expression of *LOC100190940* and *TCONS_l2_00022545* measured by qPCR in HCT-116 cells transfected with siRNA. (**B**) Relative expression of *FZD10* and *CD14* measured by qPCR after in HCT-116 cells transfected with siRNA. (**C**) Growth curves illustrated the relative cell proliferation in HCT-116 cells transfected with *LOC100190940* and *TCONS_l2_00022545* siRNAs. (**D**) Wound healing scratch assay in HCT-116 cells transfected with *LOC100190940* and *TCONS_l2_00022545* siRNAs.

We next tested the roles of dysregulated lncRNAs in CRC development. We selected two candidate lncRNAs to perform siRNA-mediated knockdown experiments, as *LOC100190940* was upregulated in primary tumor and *TCONS_l2_00022545* was upregulated in metastasis tumor. Moreover, both of the two lncRNAs were co-expressed with their neighboring protein-coding genes. Results showed that knockdown of *LOC100190940* and *TCONS_l2_00022545* (Figure 6A) significantly downregulated their neighboring protein-coding genes (Figure 6B). Additionally, knockdown of the two lncRNAs significantly decreased cell proliferation (Figure 6C) and migration (Figure 6D).

## DISCUSSION

In this study, we presented the first report on lncRNA expression landscape in patients with matched primary tumor and synchronous liver metastases. As expected, our bioinformatic approach validated several aberrantly expressed lncRNAs such as *H19*, *CRNDE* and *CCAT1* in the previous study [18]. In addition, we identified hundreds of novel dysregulated lncRNAs. Thus, our results provided a valuable resource for further lncRNA studies in CRC.

Genomic loci analysis revealed that some lncRNAs showed co-regulated expression patterns with their neighboring protein-coding genes, suggesting they displayed enhancer-like functions. For example, two lncRNAs, *TCONS_00020998* and *LOC100190940*, displayed co-expression manner with their proximal protein-coding genes, named *FZD10*, which is a member of WNT signaling. Further analysis discovered that some other lncRNAs formed networks with protein-coding genes. Interestingly, we found transcripts in metastasis cancer related module were enriched for several metabolic pathways. It has been well accepted that abnormal cellularmetabolism is a hallmark of cancer [31, 32]. In metastatic cancer, however, such phenomenon has not been fully investigated. It is speculated that mutations in metabolic pathways provided cancer cells with opportunities to evolve under the selective pressure of invaded microenvironments, which probably play a prominent role in favoring the emergence of metastatic traits [33]. Our findings thus provided new evidence for cancer metastases initiation and progression.

We also identified primary and metastasis cancer linked lncRNA signatures positively correlated with poor-prognosis gene set. To examine these lncRNA signatures in TCGA data through TANRIC, we mapped our lncRNAs to GENCODE V19 annotations. For the mapped lncRNAs, we proved that some lncRNAs were correlated with poor overall survival outcomes. Thus, our lncRNA signatures might provide new prognostic prediction and subtype classification markers. Furthermore, two studies have recently discovered that those poor-prognosis genes are prominently expressed by stromal cells rather than epithelial colon tumor cells [34, 35]. Accordingly, we suggested that primary cancer related 33-lncRNA might also expressed by stromal cells as their highly co-expression pattern with those poor-prognosis genes as well as the highly cell-type specific expression patterns for lncRNAs.

In addition to changes in expression levels, lncRNAs could be affected by driver mutations or somatically inheritable alterations. For example, a high-risk neuroblastoma-associated SNP located within the lncRNA-NBAT1 and is associated with its differential expression [14]. We mapped 83 GWAS CRC-associated SNPs to all of our lncRNA loci. Using 10 kb as the cutoff distance between a lncRNA and a SNP, we found that 9 (10.8%) of the index SNPs were near loci harboring lncRNAs. Notably, 5 of these lncRNAs showed differentially expressed levels. Functional connections between the SNPs and lncRNAs could be explained by further experiments directly, such as introducing such mutations into organoids derived from normal human intestinal epithelium [36, 37].

In conclusion, our study represented a comprehensive analysis of lncRNAs in CRC. By applying an integrative approach for the analysis of lncRNAs, we identified several dysregulated pathways activated by lncRNA in primary and metastasis cancer. Our study extended the knowledge of lncRNAs implicated in CRC and provided a new framework for future research upon lncRNA associated clinical prognosis evaluation. Altogether, our results revealed these lncRNAs could serve as potential diagnostic biomarkers or therapeutic targets in patients with CRC.

## MATERIALS AND METHODS

### RNA-seq data analysis

RNA-seq data were downloaded from NCBI GEO database under accession number GSE50760. A total of 54 pair-end SRA files were converted to raw FASTQ files then aligned to human reference genome hg19 using TopHat [38] with default parameters. We provided a custom reference gene annotation file by compiling the UCSC mRNA and lncRNA annotation files (Supplementary Material and Supplementary Table S7). The alignment BAM files were sorted and indexed, and converted into SAM files with SAMtools [39]. Then the SAM files were subjected to read counting using the python package HTSeq [40]. We chose the “union” mode of HTSeq to mask the overlapping regions between mRNA and lncRNA. R package edgeR [41] and DESeq2 [42] were used for all differential expression (DE) analysis from the raw counts and the results from the two packages were overlapped for further analysis. In all DE tests, a gene was considered significant if the FDR < 0.05 and fold change ≥ 2. The read counts were converted into RPKM (Reads Per Kilobase of exon model per Million mapped reads). The RPKM of each DE genes and lncRNAs were clustered and visualized as heatmaps in MultiExperiment Viewer (MeV version4.9.0).

### Gene co-expression network

We constructed the weighted gene co-expression networks for our expression profiles by using the WGCNA R package [43]. In total, 5,703 most variable transcripts containing both lncRNAs and mRNAs were selected for network construction. All modules that were clusters of genes that behaved similarly were assigned to a color. The module eigengene was used to represent each module, which was calculated by the first principal component, thereby capturing the maximal amount of variation of the module. Each module eigengene was related to traits (NC, PC or MC) by calculating the Pearson's correlations between the module eigengene and traits. The network was visualized by Gephi.

### Functional annotation

Gene Set Enrichment Analysis (GSEA) were performed to determine significant enrichment of genes found in a previously defined gene expression signatures using GSEA software [44]. The enrichment of KEGG pathways for lncRNAs were determined by analyzing its nearest neighboring mRNAs using DAVID [45] and PANTHER [46]. Functional annotations of significant modules were performed by DAVID.

### ChIP-seq data analysis

ChIP-seq data of H3K4me3 and input of primary CRC and matched normal colon tissue were downloaded from GSE36204. All data were aligned to hg19 reference genome using bowtie [47] with the following options: -n 2, -m 1. Enriched regions were called by MACS [48]. Differentially enriched H3K4me3 sites were determined by diffReps [49] with FDR < 0.05 and fold change > 4. Examples of ChIP-seq and RNA-seq data were visualized using the IGV browser [50].

### Correlation matrix clustering

Lists of CRCA, CCS and CCMS poor-prognosis signature genes were obtained from the supplementary tables in the respective publications. We generated a gene correlation matrix between DE lncRNAs and poor-prognosis gene sets by computing the Pearson correlation coefficient between each lncRNA and each gene. A matrix was constructed whose entries were the correlation coefficients. This matrix was clustered and visualized in MultiExperiment Viewer using a Euclidian distance metric and complete linkage clustering.

### Patients and tissue samples

To validate the results for RNA-seq data, we recruited 6 pairs of colorectal cancer tissue and corresponding non-tumor tissue samples, all of which were obtained from patients who underwent surgical operation in NO.161 hospital in 2014. All the participants were histologically confirmed to be colorectal adenocarcinoma and did not receive any other therapy on the time of enrollment. The informed consent was obtained from all the participants and procedures used in this study were approved by the institutional review boards of NO.161 hospital.

### RNA extraction and qRT-PCR

All samples were immediately frozen with liquid nitrogen after surgical resection. Total RNA was isolated using Trizol Reagent (Invitrogen) according to the manufacturer's instructions. After RNA extraction, the reverse transcription was synthesized using RevertAidTM First Strand cDNA Synthesis Kit from Fermentas according to the manufacturer's instructions using random primer. The PCR primers were designed with Primer Premier 5.0 software and β-Actin was used as a reference gene. qPCR was performed on iQ5 RealTime PCR Detection System (Bio-Rad, USA) using SYBR Green Realtime PCR Master Mix (TOYOBO CO., LTD, Japan) as the readout. All reactions were carried out in triplicates. Data was analyzed by the 2^−ΔΔCT^ method. All the primers are available on request.

### RNA interference

The siRNA oligonucleotides were synthesized by GenePharma, Inc. The target sequences were as follows:

si-LOC100190940-1: 5′- CACAGUGCCAGUAAC UUCA-3′;

si-LOC100190940-2: 5′-GGGUAGUGCUUACCU CUAU-3′;

si-LOC100190940-3: 5′- GCACACAGUUUAGAA CUUA-3′;

si-TCONS_l2_00022545-1: 5′-CCUAGAAACAGG AUGUCCU -3′;

si-TCONS_l2_00022545-2: 5′-CAGCUCAACAUG AAUCCUA-3′;

si-TCONS_l2_00022545-3: 5′-GAAGACAAUUU CUGAUAGA-3′.

HCT116 cells were transfected with 50 nM siRNA oligonucleotides in 6-well plates. The knockdown efficiency was measured by quantitative RT-PCR at 48–72 h after transfection.

### Cell viability assays

Cell viability was assessed by the Cell Counting Kit 8 (CCK-8). Briefly, siRNA and control treated HCT116 cells were seeded into 96-well plates at an initial density of 5000 cells/well. At each time points, 10 μl of CCK-8 solution was added to each well and incubated for 2 h. The absorbance was measured by scanning with a microplate reader at 450 nm. Data were expressed as the as follows: relative viability = A450 (treated) − A450 (blank) or A450 (control) − A450 (blank).

### Wound healing scratch assay

For the wound healing scratch assay, siRNA and control treated HCT116 cells were seeded in 6-well plates. After 24 h, wound was made by scratching a line across the bottom of the dish on the monolayer of the confluent cells with a sterile p-200 pipette tip. The cells were rinsed with PBS and then cultivated in the corresponding serum-deprived medium supplemented with 0.5% FBS. The same area of the gap was imaged at 50 × magnifications by using a microscope equipped with a digital camera (Olympus) at 0, 24 and 48 h after scratching.

## SUPPLEMENTARY MATERIALS TABLES




