# Supplementary material for: Integrated analysis of long non-coding RNAs in human colorectal cancer
Source: Oncotarget. 2016 Mar 19;7(17):23897–908. doi: 10.18632/oncotarget.8192 (PMC5029672; doi:10.18632/oncotarget.8192)
Supplement: Supplementary file 1 [file oncotarget-07-23897-s001.pdf]

Integrated analysis of long non–coding RNAs in human colorectal cancer

Supplementary Materials

Supplementary Table S1: Differentially expressed genes in CRC

DE Genes in primary and metastasis cancer

Supplementary Table S2: Transcripts in green module

Gene	module
ACTC1	green
ADAM12	green
ADAMTS12	green
ADAMTS14	green
ADAMTS2	green
ADAMTS4	green
ADAMTS6	green
ADAMTSL2	green
AEBP1	green
ALDH1A3	green
ANTXR1	green
ARSI	green
ASCL5	green
ASPN	green
BGN	green
BRSK2	green
C2orf27A	green
CABYR	green
CACNG4	green
CD248	green
CDH11	green
CERCAM	green
CHN1	green
CHPF	green
CHSY3	green
CILP2	green
CNIH3	green
COL10A1	green
COL11A1	green
COL12A1	green
COL15A1	green
COL1A1	green

COL1A2	green
COL3A1	green
COL4A1	green
COL4A2	green
COL5A1	green
COL5A2	green
COL5A3	green
COL6A3	green
COL6A6	green
COL8A1	green
COMP	green
CORIN	green
CPXM1	green
CPZ	green
CRABP2	green
CRISPLD1	green
CSMD2	green
CST2	green
CST4	green
CTHRC1	green
CTSK	green
CXXC5	green
DAZL	green
DKK2	green
DKK3	green
DOK5	green
ECEL1	green
ECM1	green
EGFL6	green
EGR2	green
ELTD1	green
EPGN	green
EPYC	green
FAM131C	green
FAM167B	green
FAM196B	green
FAM19A5	green
FAP	green
FGF3	green
FHL3	green
FKBP10	green
FLJ22184	green
FMO1	green
FNDC1	green
FOXC2	green
FOXS1	green
FSTL3	green

FUT7	green
GABRD	green
GALNT14	green
GEM	green
GGT5	green
GJA4	green
GPR116	green
GPR4	green
GPX8	green
GRP	green
GUCA1A	green
HAPLN3	green
HECW1	green
HEYL	green
HOXD9	green
HTRA3	green
IGFBP5	green
IGFBP7	green
IGFL1	green
IL36B	green
INHBA	green
ISM1	green
ITGA11	green
ITGA5	green
ITGBL1	green
KAL1	green
KCNE4	green
KRT23	green
KRT34	green
KRT40	green
KRT80	green
KRTAP2−3	green
LAMA4	green
LEPRE1	green
LIPN	green
LOC100862671	green
LOX	green
LOXL1	green
LOXL2	green
LPPR4	green
LRRC15	green
LRRC32	green
LTBP2	green
LUM	green
LYPD3	green
LZTS1	green
MATN3	green

MFAP2	green
MMP11	green
MMP14	green
MMP16	green
MRPS31P5	green
MSX2	green
MURC	green
MXRA5	green
MYL4	green
MYOZ1	green
NID2	green
NKD2	green
NOTCH3	green
NOTCH4	green
NOVA2	green
NOX4	green
NTM	green
ODAM	green
OLAH	green
OLFML2B	green
P4HA3	green
PCDH17	green
PDGFB	green
PDGFRB	green
PDPN	green
PERP	green
PMEPA1	green
PODNL1	green
PODXL	green
PPAPDC1A	green
PPEF1	green
PRRX1	green
PRSS22	green
PTGIR	green
PXDN	green
RAB31	green
RGS16	green
RUNX1	green
RXFP3	green
SCARF2	green
SELE	green
SERPINH1	green
SEZ6L2	green
SFRP4	green
SGIP1	green
SH3PXD2B	green
SHC2	green

SHISA2	green
SIX4	green
SLC2A1	green
SLN	green
SNCAIP	green
SOD3	green
SPARC	green
SPHK1	green
SPOCK1	green
SPON2	green
STRA6	green
SULF1	green
SYNDIG1	green
TCONS_00000740	green
TCONS_00001306+TCONS_00001307+TCONS_00001308+TCONS_00002241	green
TCONS_00002016	green
TCONS_00002976+TCONS_00003819	green
TCONS_00007178+TCONS_00006523	green
TCONS_00009563	green
TCONS_00011242+TCONS_00011243	green
TCONS_00012369+TCONS_00011626+TCONS_00011247	green
TCONS_00012828+TCONS_00012830+TCONS_00011622+TCONS_00011623+TCONS_00012368	green
TCONS_00013381	green
TCONS_00018410	green
TCONS_00019398+TCONS_00019399	green
TCONS_00019831	green
TCONS_00022031	green
TCONS_00022034	green
TCONS_00022104	green
TCONS_00022763+TCONS_00023118+TCONS_00023117+TCONS_00022764+TCONS_00023119+TCONS_00022765+TCONS_00022412	green
TCONS_00023486	green
TCONS_00024507+TCONS_00024904+TCONS_00024905+TCONS_00024508+TCONS_00024509+TCONS_00024510	green
TCONS_00025161	green
TCONS_00026520	green
TCONS_00028182	green
TCONS_l2_00008733+TCONS_l2_00008734	green
TCONS_l2_00010134	green
TCONS_l2_00012336	green

TCONS_l2_00014783+TCONS_l2_00014784+TCONS_l2_00014785+TCONS_l2_00014786+TCONS_l2_00014787+TCONS_l2_00014788+TCONS_l2_00014789+TCONS_l2_00014790+TCONS_l2_00014791+TCONS_l2_00015909+TCONS_l2_00014792+TCONS_l2_00014793+TCONS_l2_00014794+TCONS_l2_00014795+TCONS_l2_00015910+TCONS_l2_00015911+TCONS_l2_00014796+TCONS_l2_00014798+TCONS_l2_00014797+TCONS_l2_00014799+TCONS_l2_00015912+TCONS_l2_00015913+TCONS_l2_00015914+TCONS_l2_00014800+TCONS_l2_00015915+TCONS_l2_00014801+TCONS_l2_00014802+TCONS_l2_00015916+TCONS_l2_00015917+TCONS_l2_00015918+TCONS_l2_00014803+TCONS_l2_00015919+TCONS_l2_00015920+TCONS_l2_00014804+TCONS_l2_00014805+TCONS_l2_00015921+TCONS_l2_00015922	green
TENM4	green
TGFB3	green
THBS2	green
THY1	green
TLX3	green
TMEM132A	green
TMEM255B	green
TNC	green
TNFRSF12A	green
TNFRSF8	green
TNFSF4	green
TUBB3	green
TWIST1	green
UBTD1	green
VCAN	green
VPS37D	green
WISP1	green
WNT11	green
WNT9B	green
ZNF469	green
ZSWIM4	green

Supplementary Table S3: Transcripts in blue module

Gene	module
A1BG	blue
A1CF	blue
AADAC	blue
AADAT	blue
ABAT	blue
ABCA6	blue
ABCB11	blue
ABCB4	blue
ABCC11	blue
ABCC2	blue
ABCC6	blue
ABCC9	blue
ABCG5	blue
ABCG8	blue
ABHD1	blue
ABHD2	blue
ABLIM3	blue
ACAD11	blue
ACADL	blue
ACADSB	blue
ACAT1	blue
ACMSD	blue
ACOT12	blue
ACOT6	blue
ACOX2	blue
ACSL1	blue
ACSM2A	blue
ACSM2B	blue
ACSM5	blue
ACSS3	blue
ADAMTS13	blue
ADCY1	blue
ADCY10	blue
ADH1A	blue
ADH1B	blue
ADH4	blue
ADH6	blue
ADHFE1	blue
ADRA1A	blue
ADRA1B	blue
AFAP1L2	blue
AFM	blue
AFP	blue
AGMO	blue
AGT	blue

AGTR1	blue
AGXT	blue
AGXT2	blue
AGXT2L1	blue
AHSG	blue
AIG1	blue
AJAP1	blue
AKR1C1	blue
AKR1C2	blue
AKR1C4	blue
AKR1CL1	blue
AKR1D1	blue
ALB	blue
ALDH1A1	blue
ALDH1L1	blue
ALDH2	blue
ALDH4A1	blue
ALDH6A1	blue
ALDH8A1	blue
ALDOB	blue
ALLC	blue
ALPK2	blue
ALPL	blue
AMBP	blue
AMDHD1	blue
AMHR2	blue
ANG	blue
ANGPTL3	blue
ANGPTL4	blue
ANGPTL6	blue
ANKRD1	blue
ANKRD20A19P	blue
ANKRD55	blue
ANO3	blue
AOC2	blue
AOX1	blue
AOX2P	blue
APCS	blue
APOA1	blue
APOA2	blue
APOA4	blue
APOA5	blue
APOB	blue
APOC1	blue
APOC1P1	blue
APOC2	blue
APOC3	blue

APOC4	blue
APOE	blue
APOF	blue
APOH	blue
APOL5	blue
APOM	blue
AQP3	blue
AQP4	blue
AQP7P3	blue
AQP9	blue
AR	blue
ARG1	blue
ARID3C	blue
ARPP21	blue
ARSF	blue
ART4	blue
AS3MT	blue
ASGR1	blue
ASGR2	blue
ASPDH	blue
ATF5	blue
ATL1	blue
ATP2B2	blue
AVPR1A	blue
AZGP1	blue
B3GAT1	blue
BAAT	blue
BBOX1	blue
BCHE	blue
BCL6	blue
BCO2	blue
BHLHE22	blue
BHMT	blue
BHMT2	blue
BIRC7	blue
BMP10	blue
BNIP3	blue
C10orf10	blue
C10orf114	blue
C14orf105	blue
C14orf180	blue
C15orf43	blue
C19orf80	blue
C1orf168	blue
C1R	blue
C1RL	blue
C1S	blue

C2	blue
C2orf91	blue
C3	blue
C3P1	blue
C4B	blue
C4BPA	blue
C4BPB	blue
C5	blue
C5orf49	blue
C6	blue
C7	blue
C7orf76	blue
C8A	blue
C8B	blue
C8G	blue
C8orf34	blue
C8orf46	blue
C9	blue
CA5A	blue
CADM1	blue
CALR3	blue
CAMK2B	blue
CBS	blue
CCDC135	blue
CCDC158	blue
CCDC177	blue
CCDC38	blue
CCL11	blue
CCL16	blue
CCL17	blue
CD14	blue
CD302	blue
CD5L	blue
CDH2	blue
CDO1	blue
CECR2	blue
CERS4	blue
CES1	blue
CES5A	blue
CETP	blue
CFB	blue
CFH	blue
CFHR1	blue
CFHR2	blue
CFHR3	blue
CFHR4	blue
CFHR5	blue

CFI	blue
CFP	blue
CGNL1	blue
CHI3L1	blue
CHRD	blue
CHRNA4	blue
CHRNE	blue
CHST13	blue
CHST9	blue
CIDEB	blue
CLDN10	blue
CLDN14	blue
CLEC1B	blue
CLEC4C	blue
CLEC4G	blue
CLEC4M	blue
CLU	blue
CNDP1	blue
COBLL1	blue
COL26A1	blue
COL9A2	blue
COLEC10	blue
COLEC11	blue
COX6A2	blue
CP	blue
CPB2	blue
CPN1	blue
CPN2	blue
CPS1	blue
CREB3L3	blue
CRHBP	blue
CRP	blue
CRYAA	blue
CRYZ	blue
CSAD	blue
CSTA	blue
CTH	blue
CUBN	blue
CUX2	blue
CXCL2	blue
CXCL6	blue
CYB5A	blue
CYGB	blue
CYP11A1	blue
CYP17A1	blue
CYP1A1	blue
CYP1A2	blue

CYP1B1	blue
CYP21A2	blue
CYP26A1	blue
CYP27A1	blue
CYP27C1	blue
CYP2A6	blue
CYP2A7	blue
CYP2B6	blue
CYP2C18	blue
CYP2C19	blue
CYP2C8	blue
CYP2C9	blue
CYP2D6	blue
CYP2E1	blue
CYP3A4	blue
CYP3A43	blue
CYP3A5	blue
CYP3A7	blue
CYP3A7−CYP3AP1	blue
CYP4A11	blue
CYP4A22	blue
CYP4F11	blue
CYP4F2	blue
CYP4F22	blue
CYP4F3	blue
CYP4Z1	blue
CYP7A1	blue
CYP7B1	blue
CYP8B1	blue
CYTL1	blue
DAB1	blue
DAO	blue
DBH	blue
DCDC5	blue
DCXR	blue
DEFB132	blue
DEPDC7	blue
DGCR5	blue
DGCR6	blue
DHCR24	blue
DHODH	blue
DHRS2	blue
DIO1	blue
DLK1	blue
DLL1	blue
DMGDH	blue
DMRTA1	blue

DNAH11	blue
DNASE1L3	blue
DPPA4	blue
DPYS	blue
DSG1	blue
DTX1	blue
ECHDC3	blue
ECM2	blue
EFCAB12	blue
EFCC1	blue
EFHD1	blue
EHHADH	blue
ELFN1	blue
ELOVL2	blue
ENO3	blue
ENPEP	blue
ENPP1	blue
ENPP7	blue
EPHX1	blue
EPO	blue
ERO1LB	blue
ERRFI1	blue
ESR1	blue
ETNK2	blue
EVA1A	blue
EXOC3L4	blue
F10	blue
F11	blue
F12	blue
F13B	blue
F2	blue
F5	blue
F7	blue
F8	blue
F9	blue
FAH	blue
FAM167A	blue
FAM19A1	blue
FAM20A	blue
FAM9B	blue
FBN2	blue
FBN3	blue
FBP1	blue
FBXO17	blue
FBXO2	blue
FCN2	blue
FCN3	blue

FCRLB	blue
FETUB	blue
FGA	blue
FGB	blue
FGF21	blue
FGG	blue
FGL1	blue
FITM1	blue
FMO2	blue
FMO3	blue
FMO4	blue
FNDC4	blue
FOLH1	blue
FOLH1B	blue
FOXL1	blue
FRMD7	blue
FST	blue
FTCD	blue
FUOM	blue
FXYD1	blue
FXYD2	blue
G0S2	blue
G6PC	blue
GADD45B	blue
GADD45G	blue
GAMT	blue
GATA4	blue
GATM	blue
GBP7	blue
GC	blue
GCGR	blue
GCHFR	blue
GCKR	blue
GDF2	blue
GDF7	blue
GGCX	blue
GGTLC1	blue
GHR	blue
GLI1	blue
GLS2	blue
GLT1D1	blue
GLTPD2	blue
GLUD2	blue
GLYAT	blue
GLYATL1	blue
GLYCTK	blue
GMNC	blue

GNMT	blue
GOLGA6A	blue
GOLGA6B	blue
GOLGA6C	blue
GOLGA6D	blue
GOLT1A	blue
GOT1	blue
GPAM	blue
GPD1	blue
GPLD1	blue
GPR182	blue
GPR20	blue
GPR21	blue
GPR37	blue
GPR88	blue
GPR98	blue
GPT2	blue
GPX3	blue
GRB14	blue
GRHPR	blue
GRIP2	blue
GSTA1	blue
GSTA2	blue
GSTO1	blue
GULP1	blue
GYS2	blue
H19	blue
HAAO	blue
HABP2	blue
HAL	blue
HAMP	blue
HAO1	blue
HAO2	blue
HAPLN4	blue
HEPACAM	blue
HFE2	blue
HGD	blue
HGF	blue
HGFAC	blue
HHEX	blue
HLF	blue
HMOX1	blue
HOGA1	blue
HOMER2	blue
HORMAD2	blue
HP	blue
HPD	blue

HPN	blue
HPR	blue
HPX	blue
HRG	blue
HRSP12	blue
HS3ST3B1	blue
HSD11B1	blue
HSD17B13	blue
HSD17B6	blue
HSPB9	blue
HYAL1	blue
IDO2	blue
IFITM10	blue
IGDCC4	blue
IGF2BP1	blue
IGFALS	blue
IGFBP1	blue
IGFBP2	blue
IL17RB	blue
IL1RAP	blue
IL20RB	blue
IL27	blue
INHBB	blue
INHBC	blue
INHBE	blue
INMT	blue
IP6K3	blue
ISL2	blue
ITGAD	blue
ITIH1	blue
ITIH2	blue
ITIH3	blue
ITIH4	blue
KCNE1	blue
KCNH4	blue
KCNH7	blue
KCNJ8	blue
KCNN2	blue
KCNT2	blue
KDM8	blue
KHK	blue
KIAA1377	blue
KIRREL2	blue
KLB	blue
KLF15	blue
KLKB1	blue
KMO	blue

KNDC1	blue
KNG1	blue
KRT16P1	blue
KRT16P3	blue
KRT5	blue
KYNU	blue
LBP	blue
LCAT	blue
LEAP2	blue
LECT2	blue
LEPR	blue
LHX2	blue
LHX9	blue
LIN7A	blue
LINC00313	blue
LINC00478	blue
LINC00659	blue
LIPC	blue
LOC100422737	blue
LOC100505918	blue
LOC100506085	blue
LOC100506229	blue
LOC100507389	blue
LOC154092	blue
LOC157273	blue
LOC158434	blue
LOC200772	blue
LOC255167	blue
LOC283587	blue
LOC284751	blue
LOC728040	blue
LOXL4	blue
LPA	blue
LPAL2	blue
LRAT	blue
LRCOL1	blue
LRG1	blue
LRRC55	blue
LYNX1	blue
LYPD2	blue
MAOB	blue
MARCO	blue
MASP1	blue
MASP2	blue
MAT1A	blue
MBL2	blue
METTL7B	blue

MFAP3L	blue
MFSD2A	blue
MGC27382	blue
MIA2	blue
MLIP	blue
MLXIPL	blue
MOGAT1	blue
MPDZ	blue
MPPED1	blue
MRO	blue
MST1	blue
MST1P2	blue
MT1A	blue
MT1X	blue
MT2A	blue
MTHFD1	blue
MTHFS	blue
MTTP	blue
MYBPH	blue
MYO16	blue
MYO1B	blue
MYO7A	blue
MYOM1	blue
N4BP2L1	blue
NADKD1	blue
NAGS	blue
NALCN	blue
NAT8	blue
NDNF	blue
NDST3	blue
NECAB2	blue
NGFR	blue
NKX3−1	blue
NNMT	blue
NPBWR1	blue
NPC1L1	blue
NPFFR2	blue
NPPB	blue
NPW	blue
NR0B2	blue
NR1H4	blue
NR1I3	blue
NRG3	blue
NSUN6	blue
NUGGC	blue
OGDHL	blue
OIT3	blue

OLFM2	blue
ONECUT1	blue
ONECUT2	blue
ORM1	blue
ORM2	blue
OSGIN1	blue
OSR2	blue
OTC	blue
OTOA	blue
OXCT1	blue
OXER1	blue
OXT	blue
PAH	blue
PALM2	blue
PALMD	blue
PANX2	blue
PAQR9	blue
PC	blue
PCDH18	blue
PCDHA13	blue
PCDP1	blue
PCSK6	blue
PDZD2	blue
PDZK1	blue
PEBP1	blue
PECR	blue
PEMT	blue
PFKFB1	blue
PGLYRP2	blue
PIK3C2G	blue
PIPOX	blue
PKHD1	blue
PKLR	blue
PLA1A	blue
PLAT	blue
PLG	blue
PLGLA	blue
PLIN2	blue
PLIN5	blue
PNMA6C	blue
PNPLA3	blue
PNPLA5	blue
PNPO	blue
POM121L9P	blue
PON1	blue
PON3	blue
POR	blue

PPP1R1A	blue
PPP1R1C	blue
PPP4R4	blue
PRAME	blue
PRG4	blue
PROC	blue
PRODH2	blue
PROS1	blue
PROZ	blue
PRR18	blue
PRSS50	blue
PRTN3	blue
PTGR1	blue
PTH1R	blue
PVALB	blue
PXDC1	blue
PZP	blue
QDPR	blue
RANBP3L	blue
RAPGEF4	blue
RARRES2	blue
RBP4	blue
RBP5	blue
RD3	blue
RDH12	blue
RDH16	blue
REEP6	blue
RELN	blue
RGN	blue
RGPD1:chr2:−	blue
RGSL1	blue
RHBG	blue
RIPPLY1	blue
RNASE13	blue
RND1	blue
RNF165	blue
ROPN1B	blue
RORA	blue
RPGRIP1	blue
RTN4RL2	blue
RTP3	blue
RXFP1	blue
S100A12	blue
SAA1	blue
SAA2	blue
SAA2−SAA4	blue
SAA4	blue

SALL1	blue
SARDH	blue
SCTR	blue
SDC2	blue
SDS	blue
SEC14L2	blue
SEC14L3	blue
SEC16B	blue
SERPINA1	blue
SERPINA10	blue
SERPINA11	blue
SERPINA12	blue
SERPINA3	blue
SERPINA4	blue
SERPINA5	blue
SERPINA6	blue
SERPINA7	blue
SERPINC1	blue
SERPIND1	blue
SERPINF1	blue
SERPINF2	blue
SERPING1	blue
SFRP5	blue
SHBG	blue
SHMT1	blue
SIGLEC11	blue
SIGLEC7	blue
SIM1	blue
SLC10A1	blue
SLC13A5	blue
SLC16A11	blue
SLC16A13	blue
SLC16A2	blue
SLC17A1	blue
SLC17A2	blue
SLC17A3	blue
SLC1A2	blue
SLC22A1	blue
SLC22A10	blue
SLC22A25	blue
SLC22A7	blue
SLC22A9	blue
SLC23A2	blue
SLC25A18	blue
SLC25A42	blue
SLC25A47	blue
SLC26A1	blue

SLC27A2	blue
SLC27A5	blue
SLC28A1	blue
SLC2A2	blue
SLC2A9	blue
SLC34A1	blue
SLC34A2	blue
SLC38A2	blue
SLC38A3	blue
SLC38A4	blue
SLC43A3	blue
SLC47A1	blue
SLC6A1	blue
SLC6A12	blue
SLC6A13	blue
SLC6A16	blue
SLC7A2	blue
SLC7A9	blue
SLC9A7	blue
SLC9B2	blue
SLCO1A2	blue
SLCO1B1	blue
SLCO1B3	blue
SMAD9	blue
SMIM1	blue
SMLR1	blue
SMOC1	blue
SNRK−AS1	blue
SOD2	blue
SORD	blue
SOX5	blue
SPATA21	blue
SPDYC	blue
SPIC	blue
SPP2	blue
SPSB4	blue
SRD5A2	blue
ST3GAL1	blue
ST3GAL6	blue
ST6GAL1	blue
STAB2	blue
STEAP3	blue
SULT1E1	blue
SULT2A1	blue
SYT12	blue
SYT9	blue
TAT	blue

TBX15	blue
TBX5	blue
TCHH	blue
TCONS_00000209+TCONS_00000955+TCONS_00002041+TCONS_00000956	blue
TCONS_00000424+TCONS_00000070+TCONS_00001332	blue
TCONS_00000469	blue
TCONS_00000620+TCONS_00002455+TCONS_00001644	blue
TCONS_00000749	blue
TCONS_00000764	blue
TCONS_00000840+TCONS_00000154	blue
TCONS_00000942	blue
TCONS_00001142+TCONS_00000055+TCONS_00002156	blue
TCONS_00001233+TCONS_00000061+TCONS_00000367	blue
TCONS_00001384+TCONS_00000467	blue
TCONS_00001579+TCONS_00001580+TCONS_00001581+TCONS_00000088+TCONS_00000089+TCONS_00000563+TCONS_00001582	blue
TCONS_00001802+TCONS_00002577+TCONS_00002578+TCONS_00000724+TCONS_00001803+TCONS_00000725+TCONS_00000726+TCONS_00001804+TCONS_00001805	blue
TCONS_00001815+TCONS_00002584+TCONS_00002585	blue
TCONS_00001880	blue
TCONS_00002051+TCONS_00002052+TCONS_00002053+TCONS_00000971+TCONS_00002054+TCONS_00000219	blue
TCONS_00002812	blue
TCONS_00003100+TCONS_00003101+TCONS_00003102+TCONS_00003103+TCONS_00003104	blue
TCONS_00003230+TCONS_00004179+TCONS_00003231+TCONS_00003232	blue
TCONS_00003349	blue
TCONS_00003597+TCONS_00003598	blue
TCONS_00003901+TCONS_00003029	blue
TCONS_00004041+TCONS_00004042	blue
TCONS_00004139	blue
TCONS_00004164+TCONS_00004165+TCONS_00005098+TCONS_00005099+TCONS_00005101+TCONS_00004166+TCONS_00005102+TCONS_00005103+TCONS_00005104+TCONS_00005105+-TCONS_00004167	blue
TCONS_00004172	blue
TCONS_00004275+TCONS_00003300+TCONS_00004276+TCONS_00004277+TCONS_00004278+TCONS_00004279+TCONS_00002753+TCONS_00004280	blue
TCONS_00004393	blue
TCONS_00004473	blue
TCONS_00005194	blue
TCONS_00005441+TCONS_00005665	blue
TCONS_00005666	blue
TCONS_00005698+TCONS_00006358	blue
TCONS_00005825+TCONS_00005826	blue
TCONS_00005912+TCONS_00005913	blue
TCONS_00006262+TCONS_00007003	blue
TCONS_00006354+TCONS_00007061+TCONS_00007062+TCONS_00006355+TCONS_00007063+TCONS_00006356+TCONS_00005697	blue
TCONS_00006634+TCONS_00007250+TCONS_00006635+TCONS_00007251+TCONS_00005821+TCONS_00006636	blue

TCONS_00006898+TCONS_00006106+TCONS_00005550+TCONS_00006107+TCONS_00005551	blue
TCONS_00007014+TCONS_00006284+TCONS_00007015	blue
TCONS_00007049	blue
TCONS_00007321	blue
TCONS_00007487	blue
TCONS_00007580+TCONS_00008860+TCONS_00008181+TCONS_00008861+TCONS_00008862	blue
TCONS_00007761+TCONS_00007762	blue
TCONS_00008360	blue
TCONS_00008870+TCONS_00008198	blue
TCONS_00009378	blue
TCONS_00009687+TCONS_00009285+TCONS_00010364+TCONS_00009688	blue
TCONS_00009689	blue
TCONS_00009731	blue
TCONS_00009788+TCONS_00010492	blue
TCONS_00010062+TCONS_00010063	blue
TCONS_00010181	blue
TCONS_00010562+TCONS_00011091+TCONS_00011092+TCONS_00011093+TCONS_00011094+TCONS_00010563+TCONS_00011095	blue
TCONS_00010695+TCONS_00009973+TCONS_00009974	blue
TCONS_00010747+TCONS_00010748+TCONS_00010749+TCONS_00010750+TCONS_00010751	blue
TCONS_00010837+TCONS_00010838	blue
TCONS_00011219+TCONS_00011220+TCONS_00012189+TCONS_00011504+TCONS_00011505+TCONS_00011506+TCONS_00011507	blue
TCONS_00011310	blue
TCONS_00011406	blue
TCONS_00011687+TCONS_00012422	blue
TCONS_00011688+TCONS_00012423+TCONS_00012424	blue
TCONS_00011693+TCONS_00011694+TCONS_00012426	blue
TCONS_00011913	blue
TCONS_00012038	blue
TCONS_00012082	blue
TCONS_00012215	blue
TCONS_00012378+TCONS_l2_00025054+TCONS_l2_00025055+TCONS_l2_00025056+TCONS_l2_00025057	blue
TCONS_00012427+TCONS_00011695	blue
TCONS_00012819	blue
TCONS_00012836	blue
TCONS_00013008	blue
TCONS_00013649	blue
TCONS_00013751	blue
TCONS_00014512	blue
TCONS_00014599+TCONS_00014600+TCONS_00015212+TCONS_00014601+TCONS_00014602+TCONS_00015213+TCONS_00014603	blue
TCONS_00014655+TCONS_00015253	blue
TCONS_00014734	blue
TCONS_00014740	blue
TCONS_00014864	blue
TCONS_00014933	blue

TCONS_00015737	blue
TCONS_00016206	blue
TCONS_00016284	blue
TCONS_00016402	blue
TCONS_00016423	blue
TCONS_00016890	blue
TCONS_00017321	blue
TCONS_00017415+TCONS_00017416	blue
TCONS_00017608	blue
TCONS_00017779	blue
TCONS_00017969	blue
TCONS_00018322	blue
TCONS_00018476+TCONS_00017947	blue
TCONS_00018493+TCONS_00017964	blue
TCONS_00018506+TCONS_00017975+TCONS_00017976	blue
TCONS_00018703+TCONS_00018704	blue
TCONS_00018705	blue
TCONS_00018733+TCONS_00018734+TCONS_00018736+TCONS_00017830+TCONS_00018737+TCONS_00018738+TCONS_00018208+TCONS_00018739	blue
TCONS_00018899	blue
TCONS_00019109+TCONS_00019110+TCONS_00019462	blue
TCONS_00019303	blue
TCONS_00019351+TCONS_00019099+TCONS_00019352+TCONS_00019353	blue
TCONS_00019370+TCONS_00019369	blue
TCONS_00019684	blue
TCONS_00019785	blue
TCONS_00019800	blue
TCONS_00019986+TCONS_00019452	blue
TCONS_00020585	blue
TCONS_00020668+TCONS_00021269	blue
TCONS_00020678	blue
TCONS_00021280+TCONS_00021281	blue
TCONS_00022108+TCONS_00022109+TCONS_00022110+TCONS_00022111+TCONS_00022310+TCONS_00022311+TCONS_00022112+TCONS_00022113	blue
TCONS_00022301+TCONS_00022065	blue
TCONS_00022411	blue
TCONS_00022802+TCONS_00022376+TCONS_00022377+TCONS_00023134+TCONS_00023135+TCONS_00022803+TCONS_00022804+TCONS_00022378	blue
TCONS_00022841	blue
TCONS_00022878+TCONS_00022879	blue
TCONS_00023268	blue
TCONS_00023434+TCONS_00023435	blue
TCONS_00023538+TCONS_00023539	blue
TCONS_00023557+TCONS_00023558+TCONS_00024026+TCONS_00023559	blue
TCONS_00023680+TCONS_00024124+TCONS_00024125+TCONS_00023681+TCONS_00023682	blue
TCONS_00023687	blue
TCONS_00023705+TCONS_00024143+TCONS_00024144+TCONS_00023706	blue
TCONS_00023842+TCONS_00023843+TCONS_00023844+TCONS_00024221	blue

TCONS_00024332	blue
TCONS_00024645+TCONS_00024646+TCONS_00024647+TCONS_00024648	blue
TCONS_00024654+TCONS_00025008	blue
TCONS_00025185	blue
TCONS_00025313+TCONS_00025157	blue
TCONS_00025633+TCONS_00025634	blue
TCONS_00025741	blue
TCONS_00025742	blue
TCONS_00025782	blue
TCONS_00025996	blue
TCONS_00026343	blue
TCONS_00026468+TCONS_00026469	blue
TCONS_00026634+TCONS_00026635+TCONS_00026357+TCONS_00026637+TCONS_00026638	blue
TCONS_00026725	blue
TCONS_00026873	blue
TCONS_00027578+TCONS_00027580	blue
TCONS_00028047+TCONS_00028394	blue
TCONS_00028312+TCONS_00027998+TCONS_00028313+TCONS_00027999	blue
TCONS_00028332+TCONS_00028633+TCONS_00028333+TCONS_00028634+TCONS_00028635	blue
TCONS_00029027+TCONS_00029028+TCONS_00029225+TCONS_00029029	blue
TCONS_00029049+TCONS_00028853	blue
TCONS_00029436	blue
TCONS_00029614	blue
TCONS_00029707	blue
TCONS_l2_00001996+TCONS_l2_00001997+TCONS_l2_00000146	blue
TCONS_l2_00002008+TCONS_l2_00002009+TCONS_l2_00000169+TCONS_l2_00000170+TCONS_l2_00002010+TCONS_l2_00000171+TCONS_l2_00000172+TCONS_l2_00000173	blue
TCONS_l2_00002051+TCONS_l2_00002052+TCONS_l2_00002053+TCONS_l2_00000291	blue
TCONS_l2_00003554	blue
TCONS_l2_00003931+TCONS_l2_00003932+TCONS_l2_00002962+TCONS_l2_00002963	blue
TCONS_l2_00004830+TCONS_l2_00004831	blue
TCONS_l2_00005676+TCONS_l2_00005677+TCONS_l2_00005678+TCONS_l2_00005679	blue
TCONS_l2_00006655+TCONS_l2_00006289+TCONS_l2_00006290+TCONS_l2_00006291+TCONS_l2_00006292	blue
TCONS_l2_00008748+TCONS_l2_00008749+TCONS_l2_00008750+TCONS_l2_00008751	blue
TCONS_l2_00009081	blue
TCONS_l2_00009082+TCONS_l2_00009083	blue
TCONS_l2_00009224	blue
TCONS_l2_00010036+TCONS_l2_00010037	blue
TCONS_l2_00010038	blue
TCONS_l2_00011578	blue
TCONS_l2_00011633+TCONS_l2_00011137+TCONS_l2_00011138	blue
TCONS_l2_00012045+TCONS_l2_00012046	blue
TCONS_l2_00012262	blue
TCONS_l2_00012438+TCONS_l2_00012439	blue
TCONS_l2_00013506	blue
TCONS_l2_00013564+TCONS_l2_00013565+TCONS_l2_00015296+TCONS_l2_00013566+TCONS_l2_00015297	blue

TCONS_l2_00014464+TCONS_l2_00014463+TCONS_l2_00014462+TCONS_l2_00015737+TCONS_l2_00014465	blue
TCONS_l2_00014608+TCONS_l2_00014609+TCONS_l2_00014610+TCONS_00005162+TCONS_00005163+TCONS_l2_00014611	blue
TCONS_l2_00015780	blue
TCONS_l2_00016252+TCONS_l2_00016253+TCONS_l2_00016254+TCONS_l2_00016769	blue
TCONS_l2_00016931+TCONS_l2_00016666	blue
TCONS_l2_00016979+TCONS_l2_00016980+TCONS_l2_00016981+TCONS_l2_00017326+TCONS_l2_00017327	blue
TCONS_l2_00017085+TCONS_l2_00017086+TCONS_l2_00017087	blue
TCONS_l2_00017094+TCONS_l2_00017095	blue
TCONS_l2_00017600+TCONS_l2_00018152+TCONS_l2_00017601+TCONS_l2_00017602	blue
TCONS_l2_00019673+TCONS_l2_00020303+TCONS_l2_00019674+TCONS_l2_00019675+TCONS_l2_00020304+TCONS_l2_00020305+TCONS_l2_00019676+TCONS_l2_00019680+TCONS_l2_00019679+TCONS_l2_00019678+TCONS_l2_00019677+TCONS_l2_00020306+TCONS_l2_00019681+TCONS_l2_00019682	blue
TCONS_l2_00020561+TCONS_l2_00020562+TCONS_l2_00020563	blue
TCONS_l2_00020564	blue
TCONS_l2_00020565	blue
TCONS_l2_00020575	blue
TCONS_l2_00021371	blue
TCONS_l2_00021855+TCONS_l2_00021099+TCONS_l2_00021100+TCONS_l2_00021856+TCONS_l2_00021101	blue
TCONS_l2_00022545	blue
TCONS_l2_00022657	blue
TCONS_l2_00022742+TCONS_l2_00023629+TCONS_l2_00023630+TCONS_l2_00022743+TCONS_l2_00023631	blue
TCONS_l2_00022934+TCONS_l2_00023764	blue
TCONS_l2_00023026+TCONS_l2_00023027	blue
TCONS_l2_00023403+TCONS_l2_00022344+TCONS_l2_00022345+TCONS_l2_00022346+TCONS_l2_00023404	blue
TCONS_l2_00024299+TCONS_l2_00024300	blue
TCONS_l2_00024517+TCONS_l2_00024518+TCONS_l2_00024519	blue
TCONS_l2_00024926+TCONS_l2_00024927+TCONS_l2_00025478+TCONS_l2_00025479+TCONS_l2_00025480+TCONS_l2_00024928+TCONS_l2_00024929+TCONS_l2_00024930+TCONS_l2_00025481+TCONS_l2_00025482	blue
TCONS_l2_00025473+TCONS_l2_00025474+TCONS_l2_00025476+TCONS_l2_00024920+TCONS_l2_00025477+TCONS_l2_00024921+TCONS_l2_00024922+TCONS_l2_00024923+TCONS_l2_00024924+TCONS_l2_00024925	blue
TCONS_l2_00025688+TCONS_l2_00025689+TCONS_l2_00025690+TCONS_l2_00025691+TCONS_l2_00025692+TCONS_l2_00025693	blue
TCONS_l2_00025921+TCONS_l2_00025922+TCONS_l2_00025923+TCONS_l2_00027126+TCONS_l2_00025924+TCONS_l2_00025925	blue
TCONS_l2_00026819	blue
TCONS_l2_00026989+TCONS_l2_00026991	blue
TCONS_l2_00028086+TCONS_l2_00028087+TCONS_l2_00028088+TCONS_l2_00028089	blue
TCONS_l2_00028765	blue
TCONS_l2_00029403+TCONS_l2_00029404+TCONS_l2_00029991+TCONS_l2_00029990+TCONS_l2_00029989+TCONS_l2_00029405+TCONS_l2_00029406	blue
TCONS_l2_00029836+TCONS_l2_00028996	blue
TCONS_l2_00030590	blue

TCP10L	blue
TDO2	blue
TECTB	blue
TENM1	blue
TF	blue
TFPI	blue
TFR2	blue
TGM2	blue
THPO	blue
THRSP	blue
THSD7B	blue
TIMD4	blue
TM4SF4	blue
TM4SF5	blue
TM7SF2	blue
TMEM119	blue
TMEM132E	blue
TMEM170B	blue
TMEM27	blue
TMEM45A	blue
TMEM86B	blue
TMPRSS6	blue
TMPRSS9	blue
TMSB15A	blue
TNFSF14	blue
TNN	blue
TPPP2	blue
TRIM55	blue
TRPC5	blue
TRPC5OS	blue
TRPM8	blue
TSLP	blue
TTC36	blue
TTC39C	blue
TTPA	blue
TTR	blue
UGT1A1	blue
UGT1A3	blue
UGT1A4	blue
UGT1A6	blue
UGT1A9	blue
UGT2B11	blue
UGT2B15	blue
UGT2B4	blue
UGT2B7	blue
UGT3A1	blue
UPB1	blue

UPP2	blue
UROC1	blue
USH2A	blue
VMO1	blue
VNN1	blue
VTCN1	blue
VTN	blue
VWCE	blue
WDR65	blue
WDR72	blue
WNK3	blue
WWC2	blue
ZAN	blue
ZCCHC16	blue
ZIC1	blue
ZNF385B	blue

Supplementary Table S4: DE lncRNAs associated with differential modification of H3K4me3

Gene	logFC	logCPM	LR	*P* Value	FDR	H3K4me3 feature
TCONS_l2_00010508	−2.233471644	−1.899250429	10.92856921	0.000946926	0.008237251	Down
TCONS_00005877	−2.191989179	−0.461078984	11.46608904	0.000708777	0.006581532	Down
TCONS_00019766	−2.148945892	4.995485802	9.696654617	0.001846038	0.013855116	Down
TCONS_00029424	−1.979036827	−2.600356931	11.10126524	0.000862688	0.007682288	Down
LOC400891	−1.94626176	−2.777777192	14.61167054	0.000132094	0.001801667	Down
TCONS_l2_00011669	−1.822009347	−0.141738653	12.36008548	0.000438611	0.004532126	Down
TCONS_00001227+TCONS_00000060+TCONS_00002206+TCONS_00001228+TCONS_00000356+TCONS_00001229+TCONS_00001230+TCONS_00000357+TCONS_00000358	−1.774358575	−1.66497189	6.936285554	0.008446433	0.044709225	Down
ANKRD20A11P	−1.649838094	−2.609762017	8.056049368	0.004535197	0.027810243	Down
TCONS_00009789+TCONS_00009790	−1.512631138	−0.788342727	14.9655544	0.000109492	0.001548212	Down
TCONS_00020991	−1.483319666	2.011366371	10.61526046	0.001121582	0.00939955	Down
LOC344887	−1.414602756	1.417613113	11.2271903	0.000806076	0.007281401	Down
TCONS_l2_00009351	−1.406371556	−0.836845304	12.71229959	0.000363258	0.003946789	Down
TCONS_00011225	−1.246183006	4.546232083	10.00219376	0.001563539	0.012193573	Down
LOC285758	−1.21034988	1.346280281	14.21914908	0.000162706	0.002117229	Down
LOC100505633	−1.193086785	4.160713748	10.53167758	0.001173456	0.009752247	Down
TCONS_00028955	−1.157358378	−2.339718776	7.383646103	0.00658196	0.037049816	Down
TCONS_00013054+TCONS_00012887	−1.156219824	−0.770044452	12.13557078	0.000494691	0.004967202	Down
TCONS_00025946	−1.089488152	−0.415793631	9.319810537	0.002266894	0.016306286	Down
LINC00675	−1.064740758	5.155204051	10.17091707	0.001426733	0.011354121	Down
LINC00483	−1.055219595	4.122948978	11.46041128	0.000710946	0.006595311	Down

TCONS_00022098+TCONS_00021693	1.043323279	0.104165907	14.2725937	0.000158151	0.002074503	Up
TCONS_l2_00029859+TCONS_l2_00029055+TCONS_l2_00029056+TCONS_l2_00029057+TCONS_l2_00029059+TCONS_l2_00029860+TCONS_l2_00029060+TCONS_l2_00029061+TCONS_l2_00029062+TCONS_l2_00029063+TCONS_l2_00029861	1.055223931	0.306418198	9.078192251	0.002586756	0.018093515	Up
TCONS_00021692+TCONS_l2_00007598+TCONS_00021520+TCONS_00022096+TCONS_00022097	1.16323569	1.708619031	17.00554664	3.73E−05	0.000667087	Up
TCONS_00014745	1.308990668	0.67723582	15.35662342	8.90E−05	0.001316576	Up
GNAS−AS1	1.31325363	−1.912857373	8.084926045	0.004463495	0.027516315	Up
LINC00839	1.359077	−0.315914852	7.840443732	0.005109006	0.03063283	Up
TCONS_00017498+TCONS_00017315+TCONS_00017501+TCONS_00017500+TCONS_00017499+TCONS_00017503+TCONS_00016948	1.426473992	−1.482737937	12.57624024	0.000390682	0.004150798	Up
TCONS_00014977	1.43719828	0.556174429	14.58913858	0.000133683	0.001816579	Up
LOC100506810	1.567107612	−0.176547749	20.14433802	7.18E−06	0.000182456	Up

TCONS_ l2_00014783+TCONS_ l2_00014784+TCONS_ l2_00014785+TCONS_ l2_00014786+TCONS_ l2_00014787+TCONS_ l2_00014788+TCONS_ l2_00014789+TCONS_ l2_00014790+TCONS_ l2_00014791+TCONS_ l2_00015909+TCONS_ l2_00014792+TCONS_ l2_00014793+TCONS_ l2_00014794+TCONS_ l2_00014795+TCONS_ l2_00015910+TCONS_ l2_00015911+TCONS_ l2_00014796+TCONS_ l2_00014798+TCONS_ l2_00014797+TCONS_ l2_00014799+TCONS_ l2_00015912+TCONS_ l2_00015913+TCONS_ l2_00015914+TCONS_ l2_00014800+TCONS_ l2_00015915+TCONS_ l2_00014801+TCONS_ l2_00014802+TCONS_ l2_00015916+TCONS_ l2_00015917+TCONS_ l2_00015918+TCONS_ l2_00014803+TCONS_ l2_00015919+TCONS_ l2_00015920+TCONS_ l2_00014804+TCONS_ l2_00014805+TCONS_ l2_00015921+TCONS_ l2_00015922	1.688747865	3.659895274	46.27775803	1.03E−11	4.27E−09	Up
TCONS_00026520	1.711241917	−1.871712432	6.673136359	0.00978768	0.049934216	Up
TCONS_00028072	1.783211485	−1.720319892	6.965825829	0.008308108	0.044151724	Up
TCONS_00003267+TCONS_ 00003268+TCONS_00003269 +TCONS_00003270	1.887388366	−1.849160314	6.899649184	0.008621267	0.045405861	Up
TCONS_00028355	2.180220974	−2.270253825	11.49179326	0.000699042	0.006507868	Up
TCONS_00022411	2.298371205	−2.534634232	8.602566266	0.003356896	0.022145245	Up
TCONS_ l2_00023567+TCONS_ l2_00022571+TCONS_ l2_00023568+TCONS_ l2_00023569+TCONS_ l2_00023570+TCONS_ l2_00023571+TCONS_ l2_00023572	2.317159414	−2.56984515	14.37691568	0.000149625	0.001983652	Up
TCONS_00019596	2.398022804	−0.850243427	9.611683241	0.001933433	0.014372883	Up
TCONS_00018419	2.402928674	−2.124967081	11.49366698	0.000698337	0.006505704	Up

TCONS_00023842+TCONS _00023843+TCONS_0002384 4+TCONS_00024221	2.718506613	−0.842979623	16.31231118	5.37E−05	0.000890238	Up
TCONS_00025131+TCONS _00025523	2.869792051	0.102411786	32.45655695	1.22E−08	1.18E−06	Up
TCONS_00000531+TCONS _00001511	2.928182687	0.249074067	21.49061601	3.56E−06	0.00010428	Up
TCONS_00012039+TCONS _00012040	2.96180027	−2.455581339	14.84451218	0.000116747	0.00163016	Up
LINC00659	3.491281568	−0.509102382	19.20693694	1.17E−05	0.000268051	Up
TCONS_00026818	3.561771089	0.426270583	20.68390809	5.42E−06	0.000147488	Up
LOC286467	3.675118983	−0.513090784	24.95984064	5.85E−07	2.44E−05	Up

Supplementary Table S5: 33-lncRNA positively associated with poor-prognosis gene signatures

Gene
TCONS_l2_00024806+TCONS_l2_00024807+TCONS_l2_00024808+TCONS_l2_00024809+TCONS_ l2_00025414+TCONS_l2_00024810+TCONS_l2_00024811+TCONS_l2_00024812
TCONS_00017068+TCONS_00017335
TCONS_00002485
TCONS_00023755+TCONS_00024186+TCONS_00024187
TCONS_l2_00027080+TCONS_l2_00027081+TCONS_l2_00025736
LINC00648
TCONS_00016047+TCONS_00016048
CECR7
TCONS_l2_00013870+TCONS_l2_00013871+TCONS_l2_00013872
TCONS_l2_00009351
TCONS_l2_00028589+TCONS_l2_00028590+TCONS_l2_00028591+TCONS_l2_00029669+TCONS_ l2_00029670+TCONS_l2_00028592
TCONS_00001349
TCONS_00001546
TCONS_00025161
TCONS_00020476+TCONS_00020477+TCONS_00021166+TCONS_00021167+TCONS_00020478+TCONS_00020479
TCONS_00028194+TCONS_00028195+TCONS_00028559+TCONS_00028560+TCONS_00028196
TCONS_00022034
TCONS_00001306+TCONS_00001307+TCONS_00001308+TCONS_00002241
TCONS_l2_00021192+TCONS_l2_00021909+TCONS_l2_00021910+TCONS_l2_00021911+TCONS_ l2_00021912+TCONS_l2_00021193
TCONS_l2_00029645+TCONS_l2_00029647
TCONS_00022763+TCONS_00023118+TCONS_00023117+TCONS_00022764+TCONS_00023119+TCONS_00022765+ TCONS_00022412
TCONS_00026538+TCONS_00026539+TCONS_00026540
TCONS_00001808+TCONS_00001809+TCONS_00001810+TCONS_00002580+TCONS_00001811+TCONS_00002581+ TCONS_00002582
TCONS_l2_00029859+TCONS_l2_00029055+TCONS_l2_00029056+TCONS_l2_00029057+TCONS_ l2_00029059+TCONS_l2_00029860+TCONS_l2_00029060+TCONS_l2_00029061+TCONS_l2_00029062+TCONS_ l2_00029063+TCONS_l2_00029861
TCONS_00002016
FCGR1C
TCONS_00002215+TCONS_00002216+TCONS_00001243

TCONS_00018410
TCONS_00028182
TCONS_l2_00001295+TCONS_l2_00001296+TCONS_l2_00001297+TCONS_l2_00001298+TCONS_l2_00001299+TCONS_l2_00001300
TCONS_l2_00022568+TCONS_l2_00023566
TCONS_l2_00017861+TCONS_l2_00017862+TCONS_l2_00017863+TCONS_l2_00017864
TCONS_l2_00010134

Supplementary Table S6: 46-lncRNA signatures positively associated with poor-prognosis gene signatures

Gene
TCONS_00017748
TCONS_l2_00027080+TCONS_l2_00027081+TCONS_l2_00025736
CECR7
TCONS_00017714
TCONS_00008475+TCONS_00007751+TCONS_00007421+TCONS_00007420
TCONS_l2_00013918+TCONS_l2_00013919+TCONS_l2_00013920
TCONS_00026275+TCONS_00026274+TCONS_00026597+TCONS_00026596+TCONS_00026595
TCONS_l2_00024447
TCONS_l2_00004785+TCONS_l2_00005339+TCONS_l2_00004786
TCONS_l2_00015925+TCONS_l2_00015926+TCONS_l2_00014822+TCONS_l2_00015927+TCONS_l2_00014823+TCONS_l2_00015928+TCONS_l2_00015929+TCONS_l2_00014824+TCONS_l2_00015930+TCONS_l2_00015931+TCONS_l2_00014825+TCONS_l2_00014826
LOC100128164
TCONS_l2_00020176+TCONS_00006617+TCONS_00005820+TCONS_00007240+TCONS_00007241+TCONS_00006618+TCONS_00007242
TCONS_00017068+TCONS_00017335
TCONS_00028776
TCONS_l2_00021296+TCONS_l2_00021297+TCONS_l2_00021973
TCONS_00013289
LOC150622
TCONS_l2_00027297+TCONS_l2_00027298+TCONS_l2_00026429+TCONS_l2_00026430+TCONS_l2_00026431+TCONS_l2_00026432+TCONS_l2_00026433+TCONS_l2_00026434+TCONS_l2_00026435+TCONS_l2_00027299+TCONS_l2_00026436
TCONS_00010961+TCONS_00010962
TCONS_00012824+TCONS_00012825
MEIS1-AS3
LINC00578
TCONS_00025824+TCONS_00025825
TCONS_00007046
TCONS_00003719+TCONS_00002918+TCONS_00002919+TCONS_00002920+TCONS_00002921
TCONS_l2_00008040+TCONS_l2_00008041
TCONS_l2_00029085
TCONS_l2_00029086+TCONS_l2_00029087
TCONS_l2_00029088+TCONS_l2_00029089+TCONS_l2_00029090
TCONS_00014514
TCONS_00009538+TCONS_00009261+TCONS_00009539+TCONS_00010136+TCONS_00009262+TCONS_00009540+TCONS_00010137+TCONS_00010138

TCONS_l2_00004586+TCONS_l2_00004587+TCONS_l2_00004588+TCONS_l2_00004589+TCONS_l2_00004590+TCONS_l2_00004591+TCONS_l2_00004592
TCONS_00024759+TCONS_00025079+TCONS_00025080
BOLA3-AS1
TCONS_00021142+TCONS_00020439+TCONS_00020440
TCONS_l2_00014267+TCONS_l2_00014268+TCONS_l2_00014269+TCONS_l2_00014270+TCONS_l2_00014271+TCONS_l2_00015610
TCONS_00029612
TCONS_l2_00005718+TCONS_l2_00005719
TCONS_l2_00002951+TCONS_l2_00002952+TCONS_l2_00002953
TCONS_l2_00017861+TCONS_l2_00017862+TCONS_l2_00017863+TCONS_l2_00017864
TCONS_l2_00001295+TCONS_l2_00001296+TCONS_l2_00001297+TCONS_l2_00001298+TCONS_l2_00001299+TCONS_l2_00001300
TCONS_00028182
LOC100506178
TCONS_l2_00011610+TCONS_l2_00011032+TCONS_l2_00011033
TCONS_00001078+TCONS_00002106+TCONS_00001079+TCONS_00001080
LOC100506013

Supplementary Table S7: Genomic positions of lncRNAs used in this study

Chromosome	Start	End	lncRNA	Strand
chr6	161347401	161390803	TCONS_l2_00025281+TCONS_l2_00024510+TCONS_l2_00024511+TCONS_00011408	+
chr8	19103237	19103881	TCONS_00015245	+
chr2	2733680	2734729	TCONS_00003152	−
chr13	81592377	81592799	TCONS_00021860	+
chr1	168756179	168762126	LINC00626	+
chr1	152616701	152621742	TCONS_l2_00001539	−
chr19	35923859	35924720	TCONS_00026848	−
chr13	95351948	95355116	TCONS_00021688	−
chr2	123495841	123506009	TCONS_00003846	+
chr3	112455294	112468166	TCONS_00005818	−
chr14	44936162	44949833	TCONS_00022728+TCONS_00023100+TCONS_00022729	−
chr1	102251817	102252854	TCONS_l2_00001418	−
chr9	45393845	45415976	TCONS_l2_00029945	−
chr4	150877360	150879724	TCONS_00007657	+
chr3	25913584	26020527	TCONS_00005968+TCONS_00006808	+
chr2	78825037	78826532	TCONS_00003325	−
chr12	127758834	127760119	TCONS_00020985	−
chr12	9769880	9811010	LOC374443	+
chr3	193675161	193721448	LOC647323	−
chr1	156481487	156483108	TCONS_00001172+TCONS_00002182	+
chr2	67350489	67442451	LOC644838	−
chr4	14113592	14141676	LOC152742	+
chr6	165341509	165342411	TCONS_00012014	+
chr14	38385713	38397296	TCONS_00022479	+
chr2	112248850	112268567	TCONS_00002977+TCONS_00002978	+

chr1	240078739	240080255	TCONS_00000770	−
chr9	120761035	120786742	TCONS_00016472	−
chr14	76702195	76731228	TCONS_00022557+TCONS_00022558+TCONS_00022559 +TCONS_00022560	+
chr5	116078687	116097905	TCONS_00009757	−
chr19	9649409	9650405	TCONS_00026770+TCONS_00026769	+
chr7	3236258	3237888	TCONS_00013119	−
chr4	142223887	142254310	TCONS_l2_00021318+TCONS_l2_00021319+TCONS_ l2_00021320+TCONS_l2_00021321+TCONS_ l2_00022009+TCONS_l2_00021322+TCONS_ l2_00022010+TCONS_l2_00021323+TCONS_ l2_00021324+TCONS_l2_00021325+TCONS_l2_00021326	−
chr22	18761202	18779474	GGT3P	−
chr12	3477468	3480686	TCONS_00020275	+
chr15	24641974	24643464	TCONS_00023310	+
chr10	37064528	37068543	TCONS_00018161	+
chr6	8906332	8931375	TCONS_00011687+TCONS_00012422	+
chr19	55182825	55184200	TCONS_l2_00012703+TCONS_l2_00012704	+
chr1	96282861	96284231	TCONS_00001052	+
chr3	107113493	107183600	TCONS_l2_00018727+TCONS_l2_00018728+TCONS_ l2_00019853+TCONS_l2_00018729+TCONS_l2_00018730	+
chr1	96719625	96839681	TCONS_00000585	−
chr22	50111701	50118154	TCONS_00029494	−
chr21	10899127	10900124	TCONS_l2_00016963	+
chr21	44560633	44561669	TCONS_00028944+TCONS_00029155	−
chr1	142803531	142826641	TCONS_l2_00002153+TCONS_l2_00000549+TCONS_ l2_00000550+TCONS_l2_00000551+TCONS_l2_00000552	+
chr16	22823087	22824620	TCONS_00024609+TCONS_00024610	−
chr5	172700826	172708051	TCONS_00010175	+
chr12	174197	175802	TCONS_00020667	−
chr2	216341370	216351340	TCONS_00004013+TCONS_00003099	+
chr10	21629162	21661879	TCONS_00017938	−
chr12	132377835	132378419	TCONS_00021010	−
chr12	30975574	30976321	TCONS_00020752	−
chr9	134158845	134163625	TCONS_00016200	+
chr17	53900146	53930115	TCONS_00025438	+
chr20	56644110	56647126	TCONS_00028075+TCONS_00028076	−
chr6	6993424	6995787	TCONS_00011261	+
chrX	40122131	40140673	TCONS_00017417+TCONS_00016978	+
chr4	174439877	174440761	TCONS_00008936+TCONS_00008937	+
chr1	69486139	69499025	TCONS_00001522	−
chr2	421057	422303	TCONS_00002789+TCONS_00002790	+
chr4	175008679	175101572	TCONS_00008336	+
chr5	130355646	130364020	TCONS_00010087	+
chr16	76169538	76181621	TCONS_00024732	−
chr9	106006008	106015452	TCONS_00015729	+
chr5	37948593	37951157	TCONS_00009382	+
chr16	79748640	79804827	TCONS_00024736+TCONS_00025060+TCONS_00025059 +TCONS_00025058+TCONS_00024737	−

chr4	119532091	119539356	TCONS_00008207	+
chr1	230138432	230142912	TCONS_00001322+TCONS_00000414	+
chr21	25676863	25693690	TCONS_00028891	−
chr19	302198	304460	TCONS_l2_00012221+TCONS_l2_00012222+TCONS_l2_00012223+TCONS_l2_00012224+TCONS_l2_00012225	+
chr1	208434038	208440263	TCONS_00001256	+
chr13	49151110	49155037	LINC00462	−
chr5	82215195	82216938	TCONS_00009715	−
chr11	44994327	44995338	TCONS_00019627	−
chr10	27220135	27230930	LINC00202-1	−
chrX	45629146	45629608	TCONS_00017313	−
chr21	29327240	29357614	TCONS_00028826	+
chr2	102095852	102103284	TCONS_00002954	+
chr16	54279578	54304762	TCONS_00024659	−
chr13	19756124	19763579	TCONS_00021708+TCONS_00021709	+
chr5	4012822	4013763	TCONS_00009585	−
chr6	4186561	4189131	TCONS_l2_00024582+TCONS_l2_00025320	−
chr4	156363959	156368776	TCONS_00008301+TCONS_00008302	+
chr1	14548386	14549613	TCONS_00000471	−
chr12	116141253	116201346	TCONS_00020938	−
chr7	66018553	66043498	LOC493754	−
chr9	99254139	99258611	TCONS_00016423	−
chr7	3242576	3243666	TCONS_00013679	−
chr11	44995453	44999578	LOC221122	+
chr4	101733412	101735437	TCONS_00007800	−
chr13	27083364	27092904	TCONS_00021735	+
chr4	8747123	8749455	TCONS_l2_00021823+TCONS_l2_00021046+TCONS_l2_00021047	−
chr6	8790348	8792491	TCONS_00011686	+
chr6	98264473	98446788	TCONS_00012548+TCONS_00011893+TCONS_00011894+TCONS_00012549	+
chr5	140441997	140457585	TCONS_00010510	−
chr3	87138430	87206685	TCONS_00005419+TCONS_00006123+TCONS_00006124	+
chr4	174798622	174834403	TCONS_00007899+TCONS_00007900	−
chr2	238090659	238092946	TCONS_00004619	−
chr3	127002627	127006220	TCONS_l2_00019510	−
chr7	76680283	76751687	TCONS_l2_00026697+TCONS_l2_00026698+TCONS_l2_00026699+TCONS_l2_00026700+TCONS_l2_00026701+TCONS_l2_00026702+TCONS_l2_00026703+TCONS_l2_00026704+TCONS_l2_00026705+TCONS_l2_00026706+TCONS_l2_00026707+TCONS_l2_00026708+TCONS_l2_00026709+TCONS_l2_00026711+TCONS_l2_00026712+TCONS_l2_00026713+TCONS_l2_00026714	−
chr13	79361454	79414160	LINC00331	−
chr20	23979070	23980270	TCONS_00028347	−
chr6	12002391	12008853	TCONS_00012427+TCONS_00011695	+
chr5	3415160	3419427	TCONS_00009850	+
chr2	107998900	108001971	TCONS_00004369	−

chr4	69601916	69616639	TCONS_00008500+TCONS_00009069+TCONS_00008501	−
chr10	31012180	31012669	TCONS_00017808	+
chrX	136007156	136018228	TCONS_00017380	−
chr10	13425143	13442195	TCONS_00018124	+
chr8	1398398	1398683	TCONS_00014577	+
chr20	12631898	12637347	TCONS_00028318	−
chr1	33394669	33399878	TCONS_00001982	+
chr7	2757467	2764726	TCONS_00013117+TCONS_00014248	−
chr12	108308449	108309264	TCONS_l2_00005881	+
chr12	131245253	131248301	TCONS_00021257+TCONS_00020656	+
chr2	16636335	16659842	TCONS_00004156	−
chr19	16126444	16138272	LINC00661	+
chr11	81532343	81535946	TCONS_00019719	−
chr1	240902677	240906911	TCONS_00002626+TCONS_00002627+TCONS_00000771	−
chr20	36314427	36320099	TCONS_00028377	−
chr13	84638835	84656044	TCONS_00022070	−
chr8	16534479	16535141	TCONS_00014940	−
chr8	29605841	29656007	TCONS_00014486+TCONS_00014487	+
chr14	32020114	32023433	TCONS_00022459	+
chrY	14774286	14775639	TCONS_00017647	−
chr16	4304261	4305787	TCONS_00024320	+
chr10	11002114	11012779	TCONS_00017788	+
chr2	71063565	71088005	TCONS_00004303	−
chr15	26359034	26379663	TCONS_00023592+TCONS_00023593	−
chr5	103216169	103227087	TCONS_00010429+TCONS_00010430+TCONS_00011011 +TCONS_00011012	−
chr1	90976467	90999029	TCONS_00000562+TCONS_00001578	−
chr1	68297971	68668670	GNG12-AS1	+
chr19	13629790	13631071	TCONS_00026801	−
chr5	59798346	59822213	TCONS_00010350	−
chr1	5146519	5150959	TCONS_l2_00002422+TCONS_l2_00001000	−
chr16	73420704	73455295	LOC100506172	+
chr2	9243569	9244710	TCONS_00002813	+
chr6	72053204	72055909	TCONS_00012210	−
chr14	48663331	48697403	TCONS_l2_00007709+TCONS_l2_00007711+TCONS_ l2_00007710+TCONS_l2_00007712	+
chr1	147554488	147564821	TCONS_l2_00000568+TCONS_l2_00000569+TCONS_ l2_00000570+TCONS_l2_00000571	+
chr17	81054162	81063833	TCONS_l2_00011026+TCONS_l2_00011027+TCONS_ l2_00011028+TCONS_l2_00011029+TCONS_ l2_00011030+TCONS_l2_00011031	+
chr2	42242765	42252442	TCONS_00003260	−
chr6	28281281	28283677	TCONS_l2_00024682	−
chr5	52405687	52410952	LOC257396	+
chr5	132322666	132323777	TCONS_l2_00023133	−
chr11	12640871	12662326	TCONS_00019856	+
chrX	99405872	99406499	TCONS_l2_00030552	−
chr11	130596955	130605846	TCONS_00019540	+

chr8	19616323	19620042	TCONS_00014638	+
chr20	60807016	60811355	TCONS_00027874	+
chr6	77822661	77860954	TCONS_00012214	−
chr10	130114546	130118478	TCONS_00018067	−
chr10	5650438	5652756	TCONS_00017913	−
chr5	5932158	5932615	TCONS_00010257	−
chr13	39790914	39793772	TCONS_00021974	−
chr2	220658077	220659218	TCONS_00004032	+
chr18	46521050	46547823	TCONS_00026520	−
chr14	99439132	99471072	TCONS_l2_00008237+TCONS_00022867	−
chr1	168369427	168391894	LOC100505918	−
chr12	121555321	121558609	TCONS_00020595+TCONS_00021224	+
chr1	218216847	218232482	TCONS_00000393	+
chr15	92829081	92830420	TCONS_l2_00008829	+
chr4	187900644	187943127	TCONS_00007929	−
chr5	77161614	77178536	TCONS_00010003	+
chr9	137828997	137835041	TCONS_00015773	+
chr2	124597399	124671069	TCONS_00004434	−
chr8	30756833	30759195	TCONS_00014663	+
chr12	37969173	37970380	TCONS_l2_00005589	+
chr17	54614259	54616184	TCONS_00025700	−
chr9	128003881	128023167	TCONS_l2_00029021+TCONS_l2_00029022+TCONS_ l2_00029851	+
chr4	69916963	69955324	TCONS_l2_00020561+TCONS_l2_00020562+TCONS_ l2_00020563	+
chrX	114937328	114938501	TCONS_l2_00030333	+
chr7	38407246	38407746	TCONS_00013779	−
chr3	45351422	45370924	TCONS_00006007	+
chr9	99855064	99949981	TCONS_l2_00029500+TCONS_l2_00029501+TCONS_ l2_00029503+TCONS_l2_00029504+TCONS_ l2_00029505+TCONS_l2_00029506+TCONS_ l2_00029507+TCONS_l2_00029508+TCONS_ l2_00029509+TCONS_l2_00029510+TCONS_l2_00029511	−
chr2	84294294	84303339	TCONS_00003751	+
chr9	139505192	139508134	TCONS_00016231	+
chrY	9187189	9362877	FAM197Y2	−
chr5	169005469	169010607	TCONS_00010548	−
chr4	159411918	159431060	TCONS_00007889	−
chr7	35755720	35774497	TCONS_00014103+TCONS_00014104+TCONS_00012991 +TCONS_00012875+TCONS_00012992+TCONS_0001287 6+TCONS_00013411	+
chr2	108145943	108172782	TCONS_00002972+TCONS_00003816+TCONS_00002973	+
chrX	1895252	1902285	TCONS_00017125	+
chr12	117101870	117113731	TCONS_00020585	+
chr4	127964585	127998917	TCONS_00007821	−
chr10	114067936	114116353	GUCY2GP	−
chr19	9498047	9517851	TCONS_00027174	−
chrX	39226539	39251028	TCONS_00017053	−

chr12	52473526	52502031	TCONS_l2_00005676+TCONS_l2_00005677+TCONS_l2_00005678+TCONS_l2_00005679	+
chr2	216139715	216142670	TCONS_00003097	+
chr3	187824884	187864808	TCONS_00005689	+
chr2	10145573	10149991	TCONS_00004139	−
chr20	55305318	55306500	TCONS_00028234	+
chr5	37953486	38013620	TCONS_00009923+TCONS_00009383	+
chr12	147946	149412	FAM138D	−
chr17	60214314	60218096	TCONS_l2_00011390	−
chr5	63179306	63183220	TCONS_00010357	−
chr14	90959135	90959933	TCONS_00022597	+
chr12	10902708	10930035	TCONS_l2_00005518+TCONS_l2_00005519+TCONS_l2_00005520+TCONS_l2_00005521	+
chr13	114581537	114586528	TCONS_l2_00007343+TCONS_l2_00007606+TCONS_l2_00007607+TCONS_l2_00007344+TCONS_l2_00007608	−
chr4	170867132	170884485	TCONS_00007678	+
chrX	17988317	18122764	TCONS_00017043	−
chr2	105719098	105720244	TCONS_00003808	+
chr6	133409219	133427717	LINC00326	+
chrY	21094585	21239302	TTTY14	−
chr3	64875577	64878710	TCONS_00007179	−
chr12	95774452	95776303	TCONS_00020885+TCONS_00020886	−
chr14	19670792	19681016	LOC101101776	−
chr9	38433724	38462264	TCONS_l2_00028726+TCONS_l2_00028727	+
chr8	56595015	56595562	TCONS_00014702	+
chr8	48012211	48041943	TCONS_l2_00028190	−
chr15	75515790	75516799	TCONS_00023741	−
chr4	181985243	182080302	LINC00290	−
chr2	180138558	180150434	TCONS_00003059+TCONS_00003953	+
chr14	61119171	61122614	TCONS_00022526	+
chr4	58586201	58590821	TCONS_00007538	+
chr8	19780259	19782680	TCONS_00014943	−
chr1	113673733	113674746	TCONS_00001109	+
chr4	56244392	56254858	TCONS_00008077+TCONS_00008078	+
chr16	34264751	34266801	TCONS_00024373	+
chr17	74775769	74778303	TCONS_00026166	−
chr2	174162678	174163271	TCONS_l2_00014091	+
chr5	36871463	36876796	LOC646719	−
chr5	7924405	7925511	TCONS_00009345	+
chr16	33340193	33343609	TCONS_00024372	+
chr8	16990393	16996766	TCONS_00014630	+
chr5	1043539	1047475	TCONS_00010594+TCONS_00010595+TCONS_00010596+TCONS_00010597+TCONS_00009830+TCONS_00010598+TCONS_00010599	+
chr19	23197880	23207121	TCONS_00026841+TCONS_00027233+TCONS_00027710+TCONS_00027709+TCONS_00027708+TCONS_00027707+TCONS_00027706	−
chr1	150853705	150859519	TCONS_l2_00001532	−

chr14	101150209	101154155	TCONS_00022629	+
chr3	112414256	112416513	TCONS_l2_00019469	−
chr4	174264471	174291953	TCONS_00009166+TCONS_00008646+TCONS_00008647 +TCONS_00008648+TCONS_00008649	−
chr12	57480963	57482632	TCONS_00020445	+
chr2	43357103	43358647	TCONS_00003661	+
chr1	8278250	8280589	TCONS_00000468	−
chr15	84867600	84898920	LOC388152	−
chr1	13629938	13635298	TCONS_l2_00001979	+
chr2	203210998	203222427	TCONS_l2_00015083	−
chr6	114189179	114194512	LOC285758	−
chr22	25675496	25679061	TCONS_l2_00018373+TCONS_l2_00017984+TCONS_ l2_00018374+TCONS_l2_00018375	−
chr10	92213926	92290873	TCONS_00017713+TCONS_00017853+TCONS_00017854 +TCONS_00017855+TCONS_00017856	+
chr3	150618941	150621181	TCONS_00005848	−
chr5	61028617	61031526	TCONS_00009283+TCONS_00010937+TCONS_00009676	−
chr8	39389123	39409759	TCONS_l2_00027730	+
chr3	197863216	197866338	TCONS_00006776	−
chr21	36118122	36157168	LOC100506385	+
chr8	117544537	117587427	TCONS_00014836	+
chr19	29931964	29934368	TCONS_00026981	+
chr14	93357747	93359851	TCONS_00022604+TCONS_00022353+TCONS_00023035 +TCONS_00023036	+
chr17	57506496	57604193	TCONS_00025711	−
chr4	103339759	103371167	TCONS_l2_00021933+TCONS_l2_00021222+TCONS_ l2_00021223+TCONS_l2_00021224+TCONS_ l2_00021225+TCONS_l2_00021934+TCONS_ l2_00021226+TCONS_l2_00021935	−
chr4	116102449	116191383	TCONS_00008202	+
chr14	104672215	104676114	TCONS_00022643	+
chr9	136125807	136126767	TCONS_00016890	−
chr9	93224714	93345028	LOC340515	−
chr2	45240353	45240807	TCONS_00003273	−
chr20	2187141	2238703	TCONS_00028092+TCONS_00028093+TCONS_00027894 +TCONS_00027895+TCONS_00028094	+
chr10	129979400	130115990	TCONS_00017722	+
chr20	23495374	23499667	TCONS_00028345+TCONS_00028346	−
chr1	63604921	63628824	TCONS_00000217	+
chr13	30510668	30524625	LINC00544	+
chr4	179057815	179062306	TCONS_00008352	+
chr4	9376809	9388085	TCONS_00008431	−
chr21	15316096	15352765	ANKRD20A11P	−
chr13	31569299	31570275	TCONS_00021631+TCONS_00022252	−
chr15	48619765	48622979	TCONS_00023261	+
chr6	3594481	3632287	TCONS_00011435+TCONS_00012641+TCONS_00012053	−
chr1	149380676	149400614	TCONS_l2_00001517	−
chr2	242786532	242787188	TCONS_00003136	+
chr7	39659893	39662092	TCONS_00013782	−

chr19	76220	77690	FAM138F	−
chr17	21904062	21913070	FLJ36000	+
chr4	177302226	177372087	TCONS_00008342	+
chr9	132173539	132175738	TCONS_00016488	−
chr6	36641552	36643217	TCONS_l2_00024198	+
chr4	178302631	178304586	TCONS_00008347	+
chr15	48955013	48959129	TCONS_00023676+TCONS_00024120	−
chr18	12066753	12068489	TCONS_l2_00011980	−
chr13	19240085	19241008	TCONS_l2_00006689	+
chr6	137742184	137751988	TCONS_00011962+TCONS_00011963+TCONS_00011964	+
chr7	108238819	108254723	TCONS_l2_00027191+TCONS_l2_00026154+TCONS_ l2_00026155	+
chr4	25432810	25436119	TCONS_00008450	−
chr1	19658090	19660931	TCONS_00000855	+
chr6	146903783	146920067	TCONS_00012313+TCONS_00012314+TCONS_00012315 +TCONS_00011582	−
chr9	139541827	139548891	TCONS_00016232+TCONS_00016726+TCONS_00016233 +TCONS_00016234	+
chr1	20480831	20487061	TCONS_l2_00001996+TCONS_l2_00001997+TCONS_ l2_00000146	+
chr9	76218294	76221558	TCONS_00016014	+
chr7	43011866	43084204	TCONS_00013161	−
chr8	28536529	28558836	TCONS_00014954+TCONS_00014955+TCONS_00014513 +TCONS_00014553	−
chr7	119808399	119826118	TCONS_00013067	+
chr2	15026771	15047788	TCONS_00003209	−
chr4	144480625	144482613	GUSBP5	+
chr16	51788706	51847468	TCONS_00024399+TCONS_00024400+TCONS_00024401 +TCONS_00024402+TCONS_00024403+TCONS_0002440 4+TCONS_00024405	+
chr12	124060445	124062838	TCONS_00020961	−
chr5	56067335	56068811	TCONS_l2_00022912	−
chr9	93695308	93696328	TCONS_00016064	+
chr2	66305394	66312403	TCONS_00002704+TCONS_00003718	+
chr3	48247656	48256907	TCONS_00006506	−
chr4	148530703	148538396	TCONS_00007869	−
chr4	174620314	174851001	TCONS_l2_00020891+TCONS_l2_00020890+TCONS_ l2_00020892+TCONS_l2_00020893+TCONS_ l2_00021778+TCONS_l2_00021779+TCONS_ l2_00020894+TCONS_l2_00020895	+
chr12	64078484	64082180	TCONS_l2_00006295	−
chr1	111927141	111932473	PGCP1	−
chr9	74622745	74623052	TCONS_l2_00029401+TCONS_l2_00029402	−
chr12	65982911	66010730	TCONS_00020455+TCONS_00020456+TCONS_00020457	+
chr5	125412493	125621295	TCONS_00009774+TCONS_00011029+TCONS_00011030 +TCONS_00011031+TCONS_00010463	−
chr2	128168766	128169824	TCONS_00004436	−
chr7	5834113	5855979	TCONS_00013328+TCONS_00013329	+
chr1	202955580	202976393	LOC401980	−

chr2	151026010	151157594	TCONS_00003901+TCONS_00003029	+
chr12	70219289	70220910	TCONS_00020467	+
chr3	58165851	58173195	TCONS_00006033	+
chr6	86096939	86099904	TCONS_00011225	−
chr10	82003045	82009394	TCONS_l2_00003131	+
chr7	141987201	141991423	TCONS_l2_00027510	−
chr9	76026382	76044044	TCONS_00016616+TCONS_00016012	+
chr15	79044379	79045734	LOC646938	+
chr5	101479781	101482276	TCONS_00009472	+
chr21	30744821	30745886	TCONS_00028830	+
chr14	71148354	71166840	TCONS_00022548	+
chr10	106111349	106113333	TCONS_00018036	−
chr22	19010137	19011063	DGCR10	+
chr8	29386853	29387791	TCONS_00014959	−
chr2	47055003	47086145	LOC100134259	+
chr13	75824616	75826148	TCONS_00021568	+
chr1	56381282	56410636	TCONS_l2_00002502+TCONS_l2_00001243	−
chrX	404882	420768	TCONS_00017114	+
chr6	141167095	141219549	TCONS_l2_00025495+TCONS_l2_00024949+TCONS_ l2_00024950+TCONS_l2_00024951	−
chr9	72432133	72432722	TCONS_00016345	−
chr3	193919495	193967942	TCONS_00006752+TCONS_00005921+TCONS_00005922 +TCONS_00006753	−
chr6	58234692	58239687	TCONS_00012196	−
chr22	50775556	50778197	TCONS_00029742+TCONS_00029743	−
chr13	28042430	28043015	TCONS_00021948	−
chr12	130606749	130635314	TCONS_00020998	−
chr2	84106374	84114508	TCONS_00003750	+
chr3	182215996	182229636	TCONS_l2_00019638+TCONS_l2_00019637+TCONS_ l2_00020284	−
chr2	132741510	132751734	TCONS_00003406	−
chr5	135522563	135524381	TCONS_00010102	+
chr11	69240458	69244389	TCONS_00019102	+
chr15	70588401	70590244	TCONS_00024152+TCONS_00023725	−
chr2	61159963	61163467	TCONS_00002901	+
chr13	112843667	112855312	TCONS_00021912+TCONS_00021913+TCONS_00021602 +TCONS_00021914	+
chr2	64713487	64728038	TCONS_00002907	+
chr11	66774295	66778310	TCONS_00019347	+
chr12	31958675	31961607	TCONS_l2_00006194	−
chr8	60532182	60532773	TCONS_00014723	+
chr8	144362333	144363898	TCONS_00015189+TCONS_00015190	−
chr3	197171932	197185194	TCONS_00005932+TCONS_00005933	−
chr6_cox_ hap2	1380441	1384050	TCONS_00029945	+
chr2	177370974	177372430	TCONS_00002771	−
chr4	188119427	188123084	TCONS_00008705	−
chr5	168895989	168926516	TCONS_00010164	+

chr1	95820945	95846556	TCONS_00000092	−
chr1	155961181	155971578	TCONS_00000316	+
chr9	33705087	33708779	TCONS_00015947	+
chr1	183118844	183119157	TCONS_00001745	−
chr9	99995483	99997296	TCONS_00015722	+
chr17	29334910	29372673	TCONS_l2_00010686+TCONS_l2_00010687+TCONS_l2_00010688+TCONS_l2_00010689+TCONS_l2_00010690+TCONS_l2_00010691+TCONS_l2_00010692+TCONS_l2_00010693+TCONS_l2_00011546+TCONS_l2_00011547	+
chr10	45571370	45572131	TCONS_00018496	−
chr4	105151537	105332192	TCONS_00007582+TCONS_00007583	+
chr4	76103904	76105665	TCONS_00008505	−
chrY	8777989	8782196	TCONS_l2_00030979	+
chr3	38864386	38866793	TCONS_00005988+TCONS_00005513	+
chr4	159097459	159122065	TCONS_l2_00021763	+
chrX	50722362	50727003	TCONS_00017171	+
chr6	69339747	69344969	TCONS_l2_00024806+TCONS_l2_00024807+TCONS_l2_00024808+TCONS_l2_00024809+TCONS_l2_00025414+TCONS_l2_00024810+TCONS_l2_00024811+TCONS_l2_00024812	−
chr5	19035306	19038908	TCONS_l2_00023683+TCONS_l2_00023684+TCONS_l2_00022810	−
chr1	232979123	233033391	TCONS_00001328+TCONS_00002259+TCONS_00001329	+
chr21	40739091	40742922	TCONS_00029021	+
chr5	133249368	133252960	TCONS_00009511	+
chr16	20737620	20743136	TCONS_l2_00010039	−
chr2	130958857	130962255	TCONS_l2_00013983	+
chr3	84002801	84103276	TCONS_00006118+TCONS_00006117+TCONS_00006905+TCONS_00006904+TCONS_00006903+TCONS_00006902+TCONS_00006116+TCONS_00006114+TCONS_00006119	+
chr11	2891263	2893336	KCNQ1DN	+
chr1	38658716	38676024	TCONS_00000895	+
chr18	314887	318033	TCONS_00026226	+
chr7	20336279	20354142	TCONS_l2_00025704+TCONS_l2_00027066+TCONS_l2_00027067+TCONS_l2_00025705+TCONS_l2_00027068+TCONS_l2_00025706	+
chr2	42104695	42121186	LOC388942	+
chr7	24510871	24520115	TCONS_00013730	−
chr15	66143213	66152681	TCONS_00023444	+
chr2	16225344	16245963	TCONS_00003213+TCONS_00003214	−
chr20	51104461	51117130	TCONS_00028066	−
chr2	2875156	2876529	TCONS_00003548+TCONS_00002797+TCONS_00002798	+
chr17	70594180	70636611	LINC00511	−
chr1	121484057	121485434	TCONS_00001651+TCONS_00001652	−
chr6	2245987	2413825	LOC100508120	+
chr2	103726217	103792718	TCONS_00003345	−
chr5	33424131	33440725	TCONS_00009633	−
chr3	190506447	190511383	TCONS_00006734	−
chr13	51894020	51899105	TCONS_00022023	−

chr21	45626408	45627282	TCONS_00028955	−
chr8	7847937	7855043	DEFB109P1B	−
chr4	4323689	4335908	TCONS_l2_00021512+TCONS_l2_00020369	+
chr8	23316673	23336893	TCONS_00014653	+
chr1	99891593	99895068	TCONS_00001604	−
chr3	181251965	181253669	TCONS_00005898+TCONS_00005899+TCONS_00005900	−
chr3	125485334	125505694	TCONS_00006196	+
chr4	112723103	112761042	TCONS_00008197+TCONS_00007595	+
chr1	155596547	155618335	TCONS_l2_00002667+TCONS_l2_00001553	−
chr6	29163280	29168401	TCONS_00011767	+
chr22	46530796	46536813	TCONS_00029733	−
chr3	46459520	46464525	TCONS_l2_00019288	−
chr5	38710469	38720375	TCONS_00009644	−
chr20	61640735	61716423	LOC63930	+
chr2	240547392	240552533	TCONS_00003134	+
chr20	18251198	18268615	TCONS_00028324+TCONS_00028625	−
chr11	2397411	2398419	TCONS_00019823+TCONS_00019824	+
chr12	130800739	130801634	TCONS_00021003	−
chrX	73384661	73385615	TCONS_l2_00030261	+
chr7	64029793	64033609	TCONS_l2_00025937+TCONS_l2_00025938	+
chr20	44116253	44118888	TCONS_l2_00016778	+
chr1	91954577	91964760	TCONS_00002405+TCONS_00001583	−
chr3	64474647	64475112	TCONS_00006524	−
chr8	74281720	74282833	TCONS_00015039	−
chr2	4675808	4703812	LOC727982	−
chr13	72744954	72747692	TCONS_00021823	+
chr8	60959352	61028506	TCONS_00014724+TCONS_00014725	+
chrX	1851477	1882178	TCONS_l2_00030649+TCONS_l2_00030116+TCONS_ l2_00030650+TCONS_00017392+TCONS_ l2_00030117+TCONS_l2_00030118	+
chr17	70319306	70338712	TCONS_00026152+TCONS_00026153+TCONS_00026154 +TCONS_00025755+TCONS_00025756	−
chr5	177284946	177298195	TCONS_l2_00023950+TCONS_l2_00023951+TCONS_ l2_00023952	−
chr20	55039243	55043472	TCONS_00028450	−
chrX	121555856	121593433	TCONS_00017461+TCONS_00017462	+
chr5	21107540	21154785	TCONS_00009369	+
chr10	86953176	86958694	TCONS_l2_00003662+TCONS_l2_00004224+TCONS_ l2_00004225	−
chr10	71426984	71477999	TCONS_00017837+TCONS_00017838	+
chr4	99884748	99885532	TCONS_00008846	+
chr2	2614076	2617131	TCONS_00004098	−
chr4	301144	331029	TCONS_l2_00020981	−
chrY	27610233	27624537	TCONS_l2_00030915+TCONS_l2_00030916	+
chr10	89190752	89209410	TCONS_00018263	+
chr14	56961339	56963315	TCONS_00022517	+
chrX	48239521	48240390	TCONS_l2_00030483	−
chr4	157507131	157511191	TCONS_00007666	+

chr5	4512375	4516889	TCONS_00009333	+
chrX	139846725	139857448	TCONS_00017253+TCONS_00017468	+
chr6	41349175	41350213	TCONS_00012169	−
chr6_ssto_hap7	4275023	4278589	TCONS_00030013	+
chr13	68170509	68173105	TCONS_00021818	+
chr1	116966346	117021488	TCONS_l2_00002605+TCONS_l2_00001459+TCONS_00000609+TCONS_l2_00002607+TCONS_l2_00002608+TCONS_l2_00002609+TCONS_l2_00002610+TCONS_l2_00001460+TCONS_l2_00001461+TCONS_l2_00002611	−
chr10	6660597	6667308	TCONS_00018110+TCONS_00017778+TCONS_00018675	+
chr5	102744642	102748212	TCONS_00009742	−
chr5	79646424	79647785	CRSP8P	−
chr9	118774640	118775244	TCONS_00015740	+
chr19	35396205	35399631	TCONS_00026999+TCONS_00026787	+
chr21	19031594	19032702	TCONS_00028874	−
chr14	96343109	96391908	LINC00617	+
chr9	42004536	46833319	LOC643648	+
chr12	133488105	133491318	TCONS_00020261	−
chr19	28352561	28357463	TCONS_00026972	+
chr5	116156689	116165883	TCONS_00009758	−
chr9	120602600	120659534	TCONS_l2_00030072+TCONS_l2_00029573	−
chr1	48226249	48231744	TCONS_00000932	+
chr11	35086673	35095505	TCONS_00019615	−
chr2	105028685	105030466	TCONS_00003349	−
chr3	52217814	52220853	TCONS_00006511	−
chr4	183994216	183998865	TCONS_00008680+TCONS_00009183	−
chr9	66688088	66699951	TCONS_l2_00029962	−
chr5	177485726	177490591	TCONS_00010854	+
chrX	40690470	40692449	LOC100132831	−
chr18	77712626	77716325	TCONS_00026386	+
chr1	83911672	83928345	TCONS_l2_00000359+TCONS_l2_00002091+TCONS_l2_00000360+TCONS_l2_00000361	+
chr2	4005245	4021622	LOC100505964	−
chr9	43313921	43314368	TCONS_l2_00028743	+
chr1	2693739	2695364	TCONS_00001923	+
chr18	45194512	45206709	TCONS_00026328	+
chr4	174046340	174048952	TCONS_00008645	−
chr10	3935371	3937655	TCONS_00018415	−
chr4	14166079	14244437	TCONS_00007482+TCONS_00007483	+
chr6	26602961	26606885	TCONS_00011165	+
chr3	30392214	30433637	TCONS_00005976+TCONS_00006811	+
chr9	90463028	90468357	TCONS_00015840	−
chr10	9405820	9407057	TCONS_00017701	+
chr19	44699119	44702389	TCONS_00027051+TCONS_00027052	+
chr1	83485232	83568608	TCONS_l2_00001306	−
chrX	13970801	14011170	TCONS_00017294+TCONS_00017042	−

chr20	61055655	61066245	TCONS_00028260+TCONS_00028261	+
chr12	40914435	40931517	TCONS_00021098+TCONS_00020186+TCONS_00021099 +TCONS_00021100+TCONS_00021101	+
chrX	140232676	140233372	TCONS_l2_00030381	+
chrX	146981696	146982977	TCONS_00017099	−
chr6	170125600	170139088	TCONS_00011423	+
chr17	26588854	26593944	TCONS_00025601	−
chr2	78143033	78145135	TCONS_00004829+TCONS_00002941	+
chr6_qbl_ hap6	4076360	4079955	TCONS_00029989	+
chr15	93916795	93919457	TCONS_00023518	+
chr5	76446485	76449186	TCONS_00010001	+
chr9	110182565	110217258	TCONS_l2_00030058+TCONS_l2_00030059+TCONS_ l2_00029552+TCONS_l2_00029553	−
chrY	6124308	6131994	TCONS_l2_00031012+TCONS_l2_00031013	−
chr19	28926295	29218587	TCONS_l2_00013371+TCONS_l2_00013005+TCONS_ l2_00013006+TCONS_00026844	−
chr2	239419331	239464140	LOC151171	−
chrX	10866762	11129258	TCONS_00017290+TCONS_00016945+TCONS_00017038 +TCONS_00016946+TCONS_00017291	−
chr21	44777430	44782327	TCONS_00029156+TCONS_00029157+TCONS_00028946 +TCONS_00029158	−
chr17	69592479	69593420	TCONS_00025195+TCONS_00025196	+
chr11	124770622	124771240	TCONS_00019790	−
chr17	32982357	32983087	TCONS_00025611	−
chr8	81215192	81220555	TCONS_00015050+TCONS_00015456	−
chrX	51244942	51253857	TCONS_00017060	−
chr1	208047140	208052418	TCONS_00000718	−
chr5	38148582	38153817	TCONS_00009384	+
chr7	129423210	129425025	TCONS_00013253	−
chr1	234859789	234867390	LOC100506810	+
chr18	55685196	55686259	TCONS_l2_00012111	−
chr7	46300662	46334067	TCONS_00013005	+
chr7	15728003	15736507	TCONS_00012958+TCONS_00013351+TCONS_00014074	+
chr1	248881947	248894375	TCONS_l2_00002830+TCONS_l2_00001921	−
chr5	103966088	104109523	TCONS_l2_00023851+TCONS_l2_00023852+TCONS_ l2_00023043+TCONS_l2_00023044	−
chr1	239433025	239461237	TCONS_00000769	−
chrY	8572513	8573324	TTTY19	+
chr20	57721183	57734733	TCONS_00028579	+
chr10	37684566	37719648	TCONS_00018163	+
chr6	4688321	4704080	TCONS_l2_00023999	+
chr11	10948293	10949495	TCONS_00019236	+
chr20	21550602	21596659	TCONS_00028514+TCONS_00028515+TCONS_00028124	+
chr16	85433937	85434846	TCONS_00024751	−
chr3	120084474	120086653	TCONS_00006619+TCONS_00007244	−
chr2	229548253	229549296	TCONS_00003506	−
chr11	16789662	16790764	TCONS_00019585	−

chr6	38622108	38631831	TCONS_00011805+TCONS_00011806+TCONS_00011807+TCONS_00011808	+
chr4	23781076	23784409	TCONS_00008010+TCONS_00007493	+
chr7	117638912	117644099	TCONS_00013241	−
chr14	38365265	38371329	TCONS_00022365	−
chr15	39156696	39160766	TCONS_00023635	−
chr10	132237331	132281418	TCONS_00018071	−
chr4	125263314	125299036	TCONS_00007820	−
chr1	6843952	6844903	TCONS_00000466	−
chrX	134567463	134567866	TCONS_l2_00030367	+
chr6	110907715	110910218	TCONS_00011917	+
chr1	160901529	160904170	TCONS_00000651	−
chr1	246952919	246954788	LOC149134	+
chr7	105222224	105223390	TCONS_l2_00026833	−
chr3	40644616	40646918	TCONS_00005764	−
chr20	61665569	61668380	LINC00029	−
chr4	45724760	45909868	TCONS_00007758	−
chr13	19956534	19957261	TCONS_l2_00006703	+
chr3	119279389	119280416	TCONS_00006170	+
chr11	122103664	122238622	TCONS_00019785	−
chr5	124372381	124486527	TCONS_l2_00022505+TCONS_l2_00022506+TCONS_l2_00023529	+
chr2	96192399	96199699	TCONS_l2_00015401+TCONS_l2_00013803+TCONS_l2_00013804	+
chr1	3816968	3832011	LOC100133612	+
chr4	185545505	185546918	TCONS_l2_00021455	−
chr1	182667344	182667678	TCONS_00001741	−
chr14	52282313	52292090	TCONS_00022502+TCONS_00022989+TCONS_00022990+TCONS_00022503	+
chr9	117418589	117425007	TCONS_00016134+TCONS_00016674	+
chr21	44232380	44237997	TCONS_00029036	+
chr17	76356521	76361207	TCONS_00025512+TCONS_00025513+TCONS_00025984	+
chr17	62926709	62955048	TCONS_l2_00010917+TCONS_l2_00010918+TCONS_l2_00010919+TCONS_l2_00010920	+
chr6	79314184	79315753	TCONS_00011521	−
chr18	27737536	27755955	TCONS_00026472	−
chr8	64346402	64382761	TCONS_00015024	−
chr2	193614571	193641625	PCGEM1	+
chr4	120328235	120329039	TCONS_00008877	+
chr2	210045261	210045746	TCONS_l2_00014214	+
chr14	51422977	51428720	TCONS_00022409	−
chr2	104669960	104675714	TCONS_00002717	+
chr1	103228878	103319398	TCONS_00000591	−
chr7	153097005	153111048	TCONS_l2_00027522+TCONS_l2_00026982+TCONS_l2_00027523+TCONS_l2_00027524	−
chr16	31519835	31520850	TCONS_00024366+TCONS_00024367	+
chrX	117973519	117991849	TCONS_00016936+TCONS_00017008	+
chr12	9718839	9721707	TCONS_l2_00006130+TCONS_l2_00006131	−

chr14	27300735	27312008	TCONS_00022690+TCONS_00023087	−
chr6	43834218	43836122	TCONS_00012176	−
chr4	190394301	190396344	TCONS_l2_00021806+TCONS_l2_00020973	+
chr9	135894816	135896553	TCONS_l2_00030085	−
chrY	6311475	6315118	TTTY21B	+
chr12	27274886	27350376	TCONS_l2_00005567+TCONS_l2_00005568+TCONS_l2_00005569	+
chr1	231658134	231664321	TCONS_00000756+TCONS_00000757+TCONS_00000758+TCONS_00000759+TCONS_00001869+TCONS_00000760+TCONS_00001870	−
chr7	100930775	100933033	TCONS_l2_00026819	−
chr20	10727753	10734662	TCONS_l2_00016153+TCONS_l2_00016154+TCONS_l2_00016732	+
chr7	62812765	62841245	TCONS_l2_00025908+TCONS_l2_00025909+TCONS_l2_00025910+TCONS_l2_00027123+TCONS_l2_00025911+TCONS_l2_00025912	+
chr13	24901868	24903164	TCONS_l2_00006747	+
chr8	9757574	9760839	LINC00599	−
chr13	97824723	97831876	TCONS_00021690	−
chr9	43004012	43007184	TCONS_l2_00029283	−
chr16	34269170	34294714	TCONS_l2_00009701+TCONS_l2_00009702	+
chr2	199747297	199936514	TCONS_00003480	−
chr2	217664302	217668255	TCONS_00004023	+
chr6	40326483	40337213	TCONS_00011487	−
chr21	21272111	21339039	TCONS_00028973	+
chr16	24263259	24264172	TCONS_00024353	+
chr11	128160192	128163363	TCONS_00019532	+
chr5	158875564	158893284	LOC285627	−
chr5	133758501	133763748	TCONS_l2_00022523	+
chr1	206164084	206166270	TCONS_00000373	+
chr4	122411144	122436093	TCONS_00008214	+
chr12	13301829	13329452	TCONS_00020339	+
chr14	42052848	42075914	TCONS_l2_00008040+TCONS_l2_00008041	−
chr5	115703287	115710267	TCONS_00009756	−
chr18	53670850	53704521	TCONS_00026527	−
chr5	146559767	146614422	TCONS_00009801+TCONS_00010527	−
chr8	142335132	142337228	TCONS_00014878+TCONS_00014879	+
chr2	102168065	102184370	TCONS_00005215+TCONS_00004354	−
chr7	136428551	136431731	TCONS_00013979	−
chr1	149186303	149187373	TCONS_00000301	+
chr4	3760475	3765117	TCONS_00007710	−
chr13	95357954	95359564	TCONS_00022087	−
chr19	15937891	15947556	TCONS_l2_00012888	−
chr13	53775495	53777383	TCONS_l2_00006863	+
chr2	130680435	130691890	LOC389033	−
chr1	95393584	95428826	LOC729970	+
chr12	115334603	115336010	TCONS_00020575	+
chr2	121807050	121808265	TCONS_00003838	+
chr7	105954987	105955711	TCONS_l2_00026147	+

chr15	24803304	24832926	PWRN1	+
chr12	108306497	108307830	TCONS_00020558	+
chr5	99384684	99390171	TCONS_l2_00023835	−
chr6	171045261	171046865	TCONS_00011629+TCONS_00012841	−
chr20	49002366	49011265	TCONS_00028441	−
chr1	199916539	199929079	TCONS_l2_00001700	−
chr11	2357487	2363246	TCONS_00019822	+
chr8	137543022	137768320	TCONS_00014869+TCONS_00015374	+
chr14	87372122	87389099	LOC283585	+
chr12	92641531	92662777	TCONS_00020869	−
chr2	65257861	65283317	TCONS_00003306+TCONS_00003307+TCONS_00004287+TCONS_00005169+TCONS_00004288+TCONS_00004289	−
chr5	118070910	118073917	TCONS_l2_00023062	−
chr17	6856979	6859935	TCONS_00025544	−
chr5	72633733	72634360	TCONS_00009422	+
chr12	18949215	18953305	TCONS_00020349	+
chr9	70181139	70195743	TCONS_l2_00029981+TCONS_l2_00029384	−
chr22	32359344	32366387	TCONS_00029412	+
chr1	142950835	142956716	TCONS_00000629	−
chr14	90527211	90528063	TCONS_00023029	+
chr3	48755308	48767171	TCONS_00006507	−
chr17	6778553	6784435	TCONS_00025543	−
chr4	153602155	153602597	TCONS_00008291	+
chr16	68756328	68761392	TCONS_00024446	+
chr15	53375036	53393924	TCONS_00023943+TCONS_00023414	+
chr16	4230069	4233668	TCONS_00024566	−
chr3	129832346	129839595	TCONS_00007264	−
chr2	121334545	121362263	TCONS_00003833+TCONS_00003834+TCONS_00004878+TCONS_00003835+TCONS_00003836	+
chr10	124432392	124439010	TCONS_l2_00003295+TCONS_l2_00003294+TCONS_l2_00003296	+
chr10	81679934	81682875	MBL1P	+
chr5	125515270	125531403	TCONS_00009503+TCONS_00010081+TCONS_00010783+TCONS_00010784+TCONS_00010082	+
chr12	108646296	108647414	TCONS_00020912	−
chr5	10493403	10502840	TCONS_00009603+TCONS_00009604	−
chr2	202038291	202043285	TCONS_00003986+TCONS_00003987	+
chr18	74506688	74534251	LOC100131655	−
chr12	109791858	109797359	TCONS_00020918+TCONS_00021407	−
chr3	117367600	117374817	TCONS_00006166	+
chr4	190209831	190212490	TCONS_00007939	−
chr16	33573557	33582964	TCONS_l2_00009698	+
chr10	89356248	89419036	TCONS_l2_00003148+TCONS_l2_00004012+TCONS_l2_00004013	+
chr6	44012160	44012495	TCONS_00012494	+
chr12	130551606	130553098	TCONS_00020997	−
chr20	22034728	22055292	LOC100270679	+

chrX	102778694	102792880	TCONS_00017354+TCONS_00017355+TCONS_00017356 +TCONS_00017357	−
chr2	217259851	217264406	TCONS_00004016	+
chr9	65629315	65635808	TCONS_l2_00028757	+
chr1	24822823	24828850	RCAN3AS	−
chr1	228154670	228162651	TCONS_l2_00000879+TCONS_l2_00000880	+
chr9	69080244	69147854	PGM5P2	−
chr7	67615270	67753220	TCONS_l2_00027410	−
chr8	39949028	40004064	TCONS_00014982	−
chr9	98577585	98579179	TCONS_00016657	+
chr6	31439006	31440185	HCG26	+
chr14	88598540	88628279	TCONS_00022830	−
chr1	207574928	207589566	TCONS_00001789	−
chr15	78191819	78193908	TCONS_l2_00008752	+
chr2	13141015	13414889	TCONS_00003580+TCONS_00003581+TCONS_00004702	+
chr8	69901720	69942176	TCONS_00014746	+
chr4	136713539	136727059	TCONS_00007840	−
chr10	127393859	127408062	FLJ37035	−
chr1	114749049	114804900	TCONS_00000279	+
chr3	148942375	148944127	TCONS_00006262+TCONS_00007003	+
chr11	133913046	133914138	TCONS_00019544	+
chr1	85063635	85086673	TCONS_00001552+TCONS_00002400+TCONS_00001553 +TCONS_00002401	−
chr19	56207591	56215917	TCONS_00027629	+
chr1	80839049	80840306	TCONS_00000552	−
chr4	76194278	76286392	TCONS_00007781+TCONS_00007782	−
chr11	2004439	2011150	MRPL23-AS1	−
chr6	137994549	138008360	TCONS_00012607+TCONS_00011967+TCONS_00011381	+
chr6	132929364	132930441	TAAR3	−
chr7	63924219	63929615	TCONS_l2_00025936	+
chr4	186936734	186940103	TCONS_00008366	+
chr4	135257942	135273960	TCONS_l2_00021299	−
chr7	63496665	63498931	TCONS_00014311	−
chr4	9713132	9735249	TCONS_l2_00020408	+
chr17	37750971	37754703	TCONS_00025629	−
chr11	112151235	112214061	TCONS_l2_00004761+TCONS_l2_00004762+TCONS_ l2_00004763+TCONS_l2_00005335+TCONS_ l2_00004764+TCONS_l2_00004766+TCONS_ l2_00004767+TCONS_l2_00004768+TCONS_l2_00004769	+
chr13	96056526	96057258	TCONS_00021875	+
chr20	46606965	46618804	TCONS_00028420+TCONS_00028421+TCONS_00028057	−
chr6	109629773	109659068	TCONS_l2_00024368+TCONS_l2_00025219+TCONS_ l2_00024369+TCONS_l2_00024370+TCONS_l2_00024371	+
chr13	46850582	46851832	TCONS_00021993	−
chr2	65196936	65197921	TCONS_00004286	−
chr6	3254645	3258407	TCONS_00012396+TCONS_00011650+TCONS_00011651	+
chr13	77238289	77266147	TCONS_00021836	+
chr6	136871939	136872729	TCONS_00011960	+

chr5	60497136	60560756	TCONS_l2_00023749+TCONS_l2_00023750	−
chr8	7812535	7866277	FAM66E	+
chr10	82289325	82293237	TCONS_00018255	+
chr11	356462	358128	TCONS_00019182+TCONS_00019183+TCONS_00019184	+
chr13	27819823	27824854	TCONS_00022239	−
chr3	48885370	48893739	TCONS_00005515+TCONS_00005409+TCONS_00006016 +TCONS_00006017	+
chr9	747413	761551	TCONS_00016245+TCONS_00016244+TCONS_00016246	−
chr20	26171625	26174582	TCONS_00027921	+
chr7	2360640	2393863	TCONS_00013673	−
chr20	44080765	44085455	TCONS_00028177	+
chr19	18315535	18316371	TCONS_00026930	+
chr12	119210845	119212397	TCONS_00021415+TCONS_00020946	−
chr11	43662375	43694932	TCONS_00019283+TCONS_00019284	+
chr2	128143756	128163669	TCONS_l2_00013966+TCONS_l2_00013967+TCONS_ l2_00013968+TCONS_l2_00013969+TCONS_ l2_00013970+TCONS_l2_00013971+TCONS_ l2_00013972+TCONS_l2_00015477	+
chr11	17249828	17250737	TCONS_l2_00004424	+
chr11	3533505	3566749	TCONS_00019829+TCONS_00019201	+
chr6	166315365	166324507	TCONS_00012824+TCONS_00012825	−
chr6	136128286	136129604	TCONS_00011567	−
chr8	23569205	23584509	TCONS_l2_00028124+TCONS_l2_00028126	−
chr4	95664951	95679011	TCONS_00007797+TCONS_00009086	−
chr1	231464488	231467017	TCONS_00000416	+
chr15	90827638	90828974	TCONS_l2_00009281	−
chr9	3181589	3198517	TCONS_00015641	+
chr8	118406988	118531528	TCONS_00015143+TCONS_00015491+TCONS_00015144	−
chr7	44887843	44889231	TCONS_00014118+TCONS_00014117+TCONS_00013001	+
chr5	1614371	1625423	TCONS_l2_00022139	+
chr16	21353828	21358759	TCONS_l2_00009629	+
chr16	21559239	21610715	TCONS_l2_00010042+TCONS_l2_00010043+TCONS_ l2_00010044	−
chr3	52279002	52279536	TCONS_00006852	+
chr21	34528938	34531416	TCONS_00028835	+
chr9	22203989	22214671	TCONS_00015797	−
chr11	30065623	30068917	TCONS_00019598	−
chr5	65220460	65221176	TCONS_00010939	−
chr10	45086656	45097552	TCONS_00017965	−
chr5	2268285	2278578	TCONS_00009837	+
chr14	19505896	19507493	TCONS_00022416	+
chr13	97593535	97636426	LINC00359	−
chr10	31097184	31099571	TCONS_00018466	−
chr12	11638990	11639612	TCONS_00020721	−
chr10	77039484	77043129	TCONS_00017997+TCONS_00017998	−
chr19	36802245	36803570	LOC100134317	+
chr2	236174489	236182803	TCONS_00004058	+
chr2	103583866	103600887	TCONS_00003344	−

chr19	50993628	51003656	TCONS_l2_00013179	−
chr10	114610673	114615127	TCONS_00017678	+
chr22	47857049	47882860	TCONS_00029354	−
chr2	192559715	192563971	TCONS_00004538	−
chr17	18430583	18434197	TCONS_l2_00010596	+
chr4	188123140	188135298	TCONS_00007692+TCONS_00008969+TCONS_00008970 +TCONS_00007693+TCONS_00008369	+
chr13	30982444	30993138	TCONS_00021745	+
chr2	111368493	111369981	TCONS_l2_00015445	+
chr4	132301168	132513139	TCONS_l2_00021711+TCONS_l2_00021712+TCONS_ l2_00020765	+
chr11	97933035	97940762	TCONS_00019738	−
chr3	98621203	98623886	TCONS_00006134	+
chr12	130819471	130820477	TCONS_00021004	−
chr15	72757151	72766615	TCONS_00024154+TCONS_00024153+TCONS_00024155	−
chr10	93335036	93340512	TCONS_00018284	+
chr1	150583859	150587960	TCONS_00000640	−
chr3	14266506	14272302	TCONS_00005951+TCONS_00006784+TCONS_00005952	+
chr15	67223698	67234279	TCONS_00023704	−
chr4	9682416	9693365	TCONS_00008433+TCONS_00008434	−
chr9	125369138	125371333	TCONS_l2_00029018	+
chr1	60238467	60254499	TCONS_00000042+TCONS_00002047	+
chr19	18133184	18144152	TCONS_l2_00012362	+
chr9	44170438	44174635	TCONS_l2_00029297+TCONS_l2_00029298	−
chr8	28621266	28622725	TCONS_00014659	+
chr10	131910186	131919004	TCONS_00017723	+
chr10	89578070	89605369	CFL1P1	+
chr11	49327437	49401071	TCONS_l2_00004513+TCONS_l2_00004515+TCONS_ l2_00004516+TCONS_l2_00004517	+
chr17	47308234	47316621	TCONS_00025419+TCONS_00025926+TCONS_00025420	+
chrX	154696201	154723771	TMLHE-AS1	+
chr5	71817327	71956514	TCONS_00010958+TCONS_00010957+TCONS_000 10956+TCONS_00009693+TCONS_00009694+TCO NS_00009695	−
chr4	130645327	130692662	TCONS_l2_00021970+TCONS_l2_00021294+TCONS_ l2_00021295+TCONS_l2_00021971	−
chr12	111458653	111463625	TCONS_00020922	−
chr16	72317200	72342088	TCONS_00024273+TCONS_00024720	−
chr3	195341850	195356380	TCONS_l2_00019086+TCONS_l2_00019087+TCONS_ l2_00020040+TCONS_l2_00019088	+
chr3	153069868	153091975	TCONS_00007010+TCONS_00005627	+
chr5	69812079	70555122	GUSBP9	−
chr13	46255225	46256554	TCONS_00021645	−
chr3	182164758	182204150	FLJ46066	−
chr9	125693949	125702605	TCONS_00016692+TCONS_00015585	+
chr17	56154494	56160565	TCONS_00025245	−
chr12	57477095	57481646	TCONS_00020805+TCONS_00020806+TCONS_00020807 +TCONS_00021326+TCONS_00021327	−
chr22	20383731	20398695	PI4KAP1	−

chr14	86797908	86798865	TCONS_00022579	+
chr1	99937918	99999571	TCONS_00000586	−
chr20	46523123	46527679	TCONS_00028191+TCONS_00028190+TCONS_00028189+TCONS_00028188+TCONS_00028187+TCONS_00028186+TCONS_00028184+TCONS_00028192+TCONS_00028193	+
chr19	35914755	35920593	TCONS_l2_00013055+TCONS_l2_00013378	−
chr2	113402607	113403284	TCONS_00002980	+
chr18	15313555	15325918	LOC644669	−
chr14	88490894	88553688	LOC283587	+
chr10	92162278	92300562	TCONS_00018021	−
chr11	94380265	94386363	TCONS_l2_00004724+TCONS_l2_00004725	+
chr6	16153166	16164118	TCONS_l2_00024057	+
chr4	147162958	147164821	TCONS_00007649	+
chr9	136100111	136106316	TCONS_l2_00029619+TCONS_l2_00029620+TCONS_l2_00029621	−
chr8	127781646	127859220	TCONS_00015166	−
chr6	155135	156639	TCONS_00011249	+
chr3	194570164	194575044	TCONS_00006372	+
chr20	19738352	19780320	TCONS_00027907	+
chr11	64161814	64163303	TCONS_00019171	−
chr14	105559181	105565891	TCONS_00023060+TCONS_00022650+TCONS_00022651+TCONS_00022652+TCONS_00022653+TCONS_00022654+TCONS_00022655	+
chr3	185677758	185698665	LOC344887	+
chr5	37899465	37920971	TCONS_00009640	−
chr14	69723736	69725784	TCONS_00022776	−
chr14	95856125	95857241	TCONS_00022844	−
chr21	25527484	25560655	TCONS_00028890	−
chr2	724966	731224	TCONS_00003145+TCONS_00003146	−
chr1	148876040	148902123	TCONS_l2_00002184+TCONS_l2_00000577	+
chr1	149042011	149045703	TCONS_l2_00000584	+
chr16	31711934	31718745	CLUHP3	+
chr2	181940169	181941195	TCONS_00004960	+
chr11	96176446	96240041	JRKL-AS1	−
chr3	55394471	55395857	TCONS_00005777	−
chr2	207731519	207733013	TCONS_00003084	+
chr7	65995452	66006867	TCONS_l2_00025988	+
chr1	207422412	207482466	TCONS_00000374	+
chr13	47793226	47844449	TCONS_00021787	+
chr12	29294264	29301212	TCONS_00020749	−
chr2	27957840	27958477	TCONS_00004186	−
chr12	12508342	12510001	LOH12CR2	−
chr10	122952987	123072879	TCONS_00018619	−
chr3	69613257	69620273	TCONS_00005781	−
chr11	110199130	110278336	TCONS_00019109+TCONS_00019110+TCONS_00019462	+
chr2	23429445	23431272	TCONS_00004180	−
chr1	112635477	112686569	TCONS_00001629	−
chr20	26035250	26067553	FAM182A	+

chr12	40550041	40561509	TCONS_00020232+TCONS_00020233	+
chr17	70017992	70036353	TCONS_00025247+TCONS_00026151+TCONS_00025746<br>+TCONS_00025747	−
chr13	107124147	107125586	TCONS_00022104	−
chr4	161460406	161507969	TCONS_00007671	+
chr10	44273010	44274317	TCONS_00017959+TCONS_00017960+TCONS_00018925	−
chr1	30882407	30884485	TCONS_00000497	−
chr10	122574130	122584805	TCONS_00018359+TCONS_00018839	+
chr11	38210660	38211384	TCONS_00019275	+
chr1	206296385	206306131	TCONS_00000717	−
chr4	174275061	174278723	TCONS_00008324	+
chr4	9400848	9405280	TCONS_l2_00020401	+
chr22	46533092	46539488	TCONS_00029614	+
chr8	104255773	104258560	TCONS_00014543	+
chr3	16729493	16738986	TCONS_l2_00020088	−
chr3	166552809	166562744	TCONS_l2_00020255	−
chr5	35938924	35940095	TCONS_00009379	+
chr10	81421262	81591027	TCONS_l2_00003632+TCONS_l2_00003633+TCONS_<br>l2_00003634+TCONS_l2_00003636+TCONS_<br>l2_00003637+TCONS_l2_00004214+TCONS_<br>l2_00003638+TCONS_l2_00003640+TCONS_<br>l2_00003641+TCONS_l2_00003642+TCONS_<br>l2_00003643+TCONS_l2_00004215+TCONS_<br>l2_00003644+TCONS_l2_00003645+TCONS_<br>l2_00003646+TCONS_l2_00003647+TCONS_<br>l2_00003648+TCONS_l2_00003649+TCONS_<br>l2_00004216+TCONS_l2_00004217+TCONS_<br>l2_00004218+TCONS_l2_00004219+TCONS_<br>l2_00004220+TCONS_l2_00004221	−
chr19	45240862	45250906	TCONS_00027578+TCONS_00027580	+
chr4	135988388	136018813	TCONS_00007629+TCONS_00007630+TCONS_00007631	+
chr7	1548291	1549063	TCONS_00013116	−
chr8	91546765	91566828	TCONS_00015084	−
chr3	80813985	80838518	TCONS_00006103+TCONS_00005549+TCONS_000<br>06104+TCONS_00006105+TCONS_00006896+TCO<br>NS_00006897	+
chr1	213029946	213031480	FLVCR1-AS1	−
chr20	43077417	43079245	TCONS_00028047+TCONS_00028394	−
chr7	62787052	62814704	TCONS_l2_00027371	−
chr17	16878448	16884667	TCONS_l2_00011116+TCONS_l2_00011624	−
chr18	12890077	12892246	TCONS_00026291	+
chr2	145445980	145447752	TCONS_00003413	−
chr6	104785424	104786550	TCONS_00012235	−
chr19	781568	786255	TCONS_00026862	+
chr15	101708499	101709271	TCONS_00023566	+
chr11	35150431	35158084	TCONS_00019616	−
chr5	51585585	51621194	TCONS_00009959+TCONS_00009960+TCONS_00010682	+
chr18	68003294	68004545	TCONS_00026725	−
chr10	48510125	48532615	TCONS_00018500+TCONS_00018501+TCONS_00018502<br>+TCONS_00018503	−

chr12	47364907	47366007	TCONS_00020397	+
chr1	55353236	55354946	TCONS_l2_00002051+TCONS_l2_00002052+TCONS_l2_00002053+TCONS_l2_00000291	+
chr4	44018878	44024080	TCONS_00007524+TCONS_00008781+TCONS_00007525	+
chr3	41203778	41210038	TCONS_00005766	−
chr1	93795119	93805355	TCONS_00001027+TCONS_00002090	+
chr13	62139938	62147990	TCONS_00021807	+
chr11	131056917	131059648	TCONS_00019158	+
chr3	124788640	124798378	TCONS_00007245	−
chr15	101413885	101418827	TCONS_l2_00008900	+
chr13	110053200	110054833	TCONS_00022107	−
chr17	15170778	15175607	TCONS_00025211	−
chr17	20340137	20350555	TCONS_l2_00011534	+
chr12	119609311	119612391	TCONS_00020591	+
chr1	208442559	208443453	TCONS_00001791	−
chrX	80065677	80068219	TCONS_00017204	+
chr12	128151536	128155451	TCONS_00020988	−
chr5	51281812	51283777	TCONS_00009958	+
chr5	50261453	50266021	TCONS_00010327+TCONS_00009660+TCONS_00009661	−
chr13	38052747	38064928	TCONS_00021754	+
chr1	222683786	222685126	TCONS_l2_00001794	−
chr18	11910714	11918913	TCONS_00026425	−
chrX	135886490	135930749	TCONS_l2_00030613+TCONS_l2_00030614+TCONS_l2_00030615+TCONS_l2_00030616+TCONS_l2_00030617	−
chrX	56644824	56650891	TCONS_l2_00030697	+
chr13	25746966	25754217	TCONS_l2_00007499+TCONS_l2_00007088+TCONS_l2_00007500	−
chrX	72218440	72222695	TCONS_00017069	−
chr18	73219889	73221885	TCONS_00026568	−
chr21	44783212	44786446	TCONS_00028851	+
chr6	53095403	53096831	TCONS_00012744	−
chrX	73247971	73513409	FTX	−
chr2	65663864	65928945	TCONS_l2_00015349+TCONS_00002909+TCONS_l2_00015350	+
chr18	22069210	22070990	TCONS_00026298	+
chr12	6258941	6289931	TCONS_00020692+TCONS_00020693+TCONS_00020694	−
chr2	176748704	176749791	TCONS_00004502	−
chr13	22322473	22326718	TCONS_00021716	+
chr2	195595319	195626159	TCONS_00003071	+
chr2	3160527	3161568	TCONS_00002799	+
chr2	205092860	205099708	TCONS_00003485	−
chr11	76463774	76469359	TCONS_00019394+TCONS_00019395+TCONS_00019396+TCONS_00019397	+
chr1	210438848	210440481	TCONS_l2_00001744	−
chr14	101985786	101989640	TCONS_00022883	−
chr5	10137250	10138477	TCONS_00009351	+
chr4	4923787	4924981	TCONS_00008414	−
chr5	117931883	117963787	TCONS_00009496	+

chr7	42869271	42884037	TCONS_00013160	−
chr3	147539274	147541246	TCONS_00006256	+
chr19	43158078	43173162	TCONS_l2_00012563	+
chr2	150143468	150146685	TCONS_00003896+TCONS_00004912+TCONS_00003897	+
chr6	31789991	31795104	TCONS_00011779	+
chr17	29914339	29986632	TCONS_00025605	−
chr3	28038701	28070095	TCONS_l2_00020100+TCONS_l2_00020101	−
chr4	6765264	6769333	TCONS_00007972	+
chr21	23470936	23488847	LINC00308	+
chr17	2659041	2675976	TCONS_l2_00010508	+
chr1	90251627	90253148	TCONS_00001024	+
chr10	27255640	27264921	TCONS_l2_00003442+TCONS_l2_00003443	−
chr3	180131962	180220736	TCONS_00005667+TCONS_00006321+TCONS_00006320 +TCONS_00005668+TCONS_00007047	+
chr6	158658351	158663662	TCONS_l2_00024998+TCONS_l2_00024999	−
chr5	6868672	6886900	TCONS_00009341	+
chr11	71159586	71163182	TCONS_00019374	+
chr15	32698812	32727250	ULK4P1	−
chr14	86378385	86596675	TCONS_00022572+TCONS_00022573+TCONS_00022574 +TCONS_00022575+TCONS_00022576+TCONS_0002257 7+TCONS_00022578	+
chr14	104341297	104346670	TCONS_00022642	+
chr10	112874075	112877848	TCONS_00018335	+
chr6	19290550	19321252	TCONS_00011459	−
chr1	247996652	247998156	TCONS_l2_00001916	−
chr1	84937581	84943596	TCONS_00001551	−
chr7	128103508	128110691	TCONS_l2_00026188	+
chr6	729068	730060	TCONS_00012035	−
chr16	75499272	75499773	TCONS_00024729	−
chr5	144717495	144761233	TCONS_00009796	−
chr3	86531093	86546146	TCONS_00005554	+
chr2	9144182	9148968	TCONS_00003558	+
chr4	124454586	124457636	TCONS_00008566	−
chr11	110792737	110794664	TCONS_00019754	−
chr4	12471482	12611001	TCONS_00008437+TCONS_00008438	−
chr12	111395036	111395608	TCONS_00020920	−
chr19	14898316	14902721	TCONS_00026905+TCONS_00026906	+
chr8	123673025	123682739	TCONS_00015152	−
chr2	1753646	1757701	TCONS_00003544+TCONS_00004671	+
chr5	134374528	134375737	TCONS_00009789+TCONS_00009790	−
chrX	64808261	64809649	TCONS_00016990	+
chr3	169661772	169684522	LOC100128164	−
chr14	84761936	84765469	TCONS_00022821	−
chr6	52505256	52506083	TCONS_00011838	+
chr12	88775675	88813029	TCONS_00020862+TCONS_00020863	−
chr1	143424333	143467651	TCONS_l2_00002643+TCONS_l2_00001500+TCONS_ l2_00002644+TCONS_l2_00002645+TCONS_l2_00002646	−
chr18	7272467	7277412	TCONS_00026273	+

chr7	149581362	149606795	TCONS_l2_00026968+TCONS_l2_00026969	−
chr21	28731204	28820898	TCONS_l2_00017473+TCONS_l2_00017474	−
chr2	215674995	215698897	TCONS_00004010	+
chr12	109154469	109162474	TCONS_00020916	−
chr3	28616769	28799828	LINC00693	+
chr3	128719320	128720388	TCONS_00005825+TCONS_00005826	−
chr8	19134	20808	TCONS_00014550	−
chr21	30554348	30560116	TCONS_00028911	−
chr1	148352316	148354746	TCONS_00001140	+
chr6	170462441	170466134	TCONS_00012029+TCONS_00012030	+
chr22	46000312	46016302	TCONS_00029353+TCONS_00029723+TCONS_00029924	−
chr2	9705799	9715751	TCONS_00003561+TCONS_00003562+TCONS_00003563+TCONS_00003564+TCONS_00004691+TCONS_00003565	+
chr12	127645747	127647390	TCONS_00020984	−
chr5	10775170	10777174	TCONS_00009605	−
chr2	158066906	158067991	TCONS_l2_00014959	−
chr21	21265593	21272069	TCONS_00028878	−
chr4	100573939	100575021	TCONS_l2_00020652	+
chr10	44787546	44789628	TCONS_00018493+TCONS_00017964	−
chr4	57975928	58071465	LOC255130	+
chr2	190405248	190406731	TCONS_00003970	+
chr9	105052402	105053194	TCONS_00015728	+
chr16	85316564	85321685	LINC00311	+
chrY	25082602	26753172	TTTY4C	+
chr5	173006646	173012075	LOC285593	+
chr8	66439243	66474901	LOC286186	−
chr5	135712036	135732730	TCONS_l2_00023905	−
chr2	174745531	174764191	TCONS_l2_00014990+TCONS_l2_00016019	−
chr13	98714196	98716549	TCONS_00022093	−
chr12	88006398	88043289	TCONS_00020860	−
chr1	696291	697369	TCONS_00000126	+
chr5	154258474	154263999	TCONS_00010144	+
chr2	222501967	222548299	TCONS_00004036+TCONS_00003113	+
chr7	62809448	62812152	LOC100287834	+
chr9	90717701	90728354	TCONS_00016395+TCONS_00016825	−
chr5	135802366	135855987	TCONS_00009522+TCONS_00009523	+
chr7	31537026	31539781	TCONS_00013774	−
chr15	31126795	31129026	TCONS_l2_00008535	+
chr4	6672410	6675587	TCONS_00009022+TCONS_00007711+TCONS_00007712+TCONS_00008417	−
chr17	47556110	47566925	TCONS_00025234	−
chr4	206389	249773	ZNF876P	+
chr4	136034060	136044934	TCONS_00007838+TCONS_00007839	−
chr8	8780049	8784509	TCONS_00014594	+
chr8	97465313	97469664	TCONS_00014801	+
chr1	247337101	247349239	TCONS_l2_00000939+TCONS_l2_00000940+TCONS_l2_00000941	+

chr7	27268955	27275492	TCONS_00013752	−
chr6	137764901	137782759	TCONS_00011965+TCONS_00012606+TCONS_00011966	+
chr5	110382223	110405612	TCONS_00010438	−
chr6	108596846	108597750	TCONS_00011350	+
chr13	90201048	90216667	LINC00353	−
chr5	56946519	56976592	TCONS_00010695+TCONS_00009973+TCONS_00009974	+
chr2	53091373	53093769	TCONS_00002893	+
chr19	56123598	56128560	TCONS_l2_00013242+TCONS_l2_00013243+TCONS_ l2_00013244	−
chr2	186815194	186817647	TCONS_00004522	−
chr17	72376325	72377340	TCONS_00025483	+
chr2	200835633	200875929	TCONS_00004551	−
chr7	113704262	113715998	TCONS_00013929+TCONS_00013930	−
chr12	115059312	115060725	TCONS_00020574	+
chr17	15668017	15669003	CDRT15P2	+
chr6	115954706	115965044	TCONS_l2_00025460+TCONS_l2_00024891	−
chr8	123713327	123746233	TCONS_00015154	−
chr3	117438744	117447915	TCONS_00006933+TCONS_00006932	+
chr10	45642842	45646066	TCONS_l2_00003966+TCONS_l2_00003035+TCONS_ l2_00003036	+
chr14	103585407	103586942	TCONS_00022640	+
chr11	131104433	131121711	TCONS_00019160	+
chr13	24532747	24545841	TCONS_l2_00006745+TCONS_l2_00006746	+
chr2	218659739	218660211	TCONS_l2_00015120	−
chr9	25613547	25617675	TCONS_00015935	+
chr1	93775666	93811368	LOC100131564	−
chr4	177787768	177796611	TCONS_00007908	−
chr4	190470757	190498185	TCONS_00008984+TCONS_00008388+TCONS_00008985 +TCONS_00008986+TCONS_00008987+TCONS_0000898 8+TCONS_00008390+TCONS_00008389	+
chr4	3641197	3643784	TCONS_00007958	+
chr8	8836695	8846327	TCONS_00014596	+
chr2	34902480	34963298	TCONS_l2_00013564+TCONS_l2_00013565+TCONS_ l2_00015296+TCONS_l2_00013566+TCONS_l2_00015297	+
chr5	43357143	43363282	TCONS_00010324	−
chr6	169390444	169423958	TCONS_00012023	+
chr17	76613491	76615641	TCONS_00025772+TCONS_00025773	−
chr6	123427049	123433459	TCONS_00011945+TCONS_00012594	+
chr8	109264295	109271716	TCONS_00014830	+
chr18	9290915	9334462	TCONS_00026407+TCONS_00026408+TCONS_00026409 +TCONS_00026668+TCONS_00026410+TCONS_00026411	−
chr5	77253954	77254925	TCONS_00010004	+
chr10	5319063	5327888	TCONS_l2_00004090+TCONS_l2_00003376+TCONS_ l2_00003377	−
chr12	115740640	115741687	TCONS_00020936	−
chr8	1200380	1212592	TCONS_00015204+TCONS_00014572	+
chr3	182082935	182086044	TCONS_00005674	+
chr3	129869290	129872415	TCONS_00006230	+

chr2	129999746	130031421	TCONS_l2_00015957+TCONS_l2_00014879+TCONS_l2_00014880	−
chr1	329784	342806	TCONS_l2_00001926+TCONS_l2_00001927	+
chr15	88120160	88122917	LINC00052	+
chr2	140582465	140583446	TCONS_00003410	−
chr3	180244943	180319723	TCONS_l2_00019628+TCONS_l2_00019629+TCONS_l2_00020278	−
chr3	168619733	168639784	TCONS_00005654	+
chr1	147494090	147498172	TCONS_00000298+TCONS_00000299	+
chr21	46398627	46399161	TCONS_00029180+TCONS_00029181	−
chr5	135078142	135082167	TCONS_l2_00023903+TCONS_l2_00023904	−
chr16	52112549	52132980	TCONS_00024406+TCONS_00024407+TCONS_00024408+TCONS_00024409+TCONS_00024410	+
chr6	155844869	155847204	TCONS_00011997	+
chr13	28312873	28314262	TCONS_00021737	+
chr16	18475644	18483397	TCONS_l2_00010023+TCONS_l2_00010024	−
chr22	23668803	23670680	TCONS_00029366	+
chr2	70321029	70322418	TCONS_00003320+TCONS_00004301	−
chr1	192498709	192507232	TCONS_l2_00002268	+
chr1	57093429	57110930	TCONS_00002384+TCONS_00001504+TCONS_00001505	−
chr5	1002178	1003982	TCONS_00010231	−
chr6	5892673	5986353	TCONS_l2_00025079+TCONS_l2_00024003	+
chr9	127961813	127962755	TCONS_00016868	−
chr7	130480541	130517579	TCONS_l2_00026892	−
chr6	167655379	167659404	TCONS_00011415	+
chr6	23416372	23455725	TCONS_00011737	+
chr19	31702094	31716030	TCONS_00026984	+
chr20	50827178	51166657	TCONS_l2_00016333+TCONS_l2_00016335+TCONS_l2_00016336	+
chr2	109684999	109686355	TCONS_00004377	−
chr12	132906471	132912612	TCONS_l2_00006027	+
chr16	65405003	65427604	TCONS_00024862	+
chr19	22486560	22496723	TCONS_l2_00012404	+
chr1	47846387	47874257	TCONS_00001484+TCONS_00000516+TCONS_00001485	−
chr10	13554373	13570545	TCONS_00018885+TCONS_00018886+TCONS_00018887+TCONS_00018888+TCONS_00018890+TCONS_00018889	−
chr10	6779344	6780988	TCONS_00017779	+
chr3	193560349	193561798	TCONS_l2_00020301+TCONS_l2_00019669	−
chr16	32399582	32416332	TCONS_l2_00009679	+
chr9	110348379	110355877	TCONS_00016124	+
chr10	91408695	91410349	TCONS_00018273	+
chr13	44670879	44711480	TCONS_00021982+TCONS_00021983+TCONS_00021984+TCONS_00022266+TCONS_00021641	−
chr2	171627604	171634757	LOC100505695	+
chr6	100546759	100555634	TCONS_l2_00024354	+
chr15	81699830	81702193	TCONS_00023483	+
chr3	163192855	163197153	TCONS_00005639	+

chr8	38408981	38410871	TCONS_00014978	−
chrY	3904538	3968361	TCONS_00017608	+
chr11	18610355	18612385	TCONS_00019588	−
chr12	85763589	85818671	TCONS_00021176+TCONS_00020499	+
chr1	234852452	234855189	TCONS_00002622	−
chr1	147169914	147171461	TCONS_00000296	+
chr1	30726193	30734008	TCONS_00000879	+
chr15	101630821	101649239	TCONS_00023839	−
chr2	109686929	109690815	TCONS_00003817	+
chr18	75701645	75702115	TCONS_00026582	−
chr8	49608009	49609394	TCONS_00014693	+
chr12	4207356	4208695	TCONS_l2_00005430	+
chr2	140017509	140021549	TCONS_00003886+TCONS_00003887+TCONS_00004903	+
chr22	23734736	23735566	TCONS_l2_00017637	+
chrY	6339072	6341671	TTTY8	−
chr3	142802859	142803787	TCONS_00006252	+
chr6	153115631	153153048	TCONS_00011596+TCONS_00011597	−
chr12	5618667	5634785	TCONS_00021026+TCONS_00020283+TCONS_00020284	+
chr13	109934308	109959966	TCONS_00021897+TCONS_00021594+TCONS_00021898	+
chr22	39317232	39320713	TCONS_00029424	+
chr20	23105705	23113273	LINC00656	−
chr16	80562541	80574385	TCONS_00024739	−
chr6	132929270	132929899	TCONS_l2_00024424	+
chr13	110459260	110460349	TCONS_00021899	+
chr7	124812271	125019375	TCONS_l2_00027211	+
chr7	126019024	126064239	TCONS_00013075	+
chr1	77533605	77544167	TCONS_00000237	+
chr6	89673583	89675075	TCONS_00011882	+
chr14	99624753	99626006	TCONS_00022399	+
chr12	48405088	48405950	TCONS_00020405	+
chr16	1336272	1336612	TCONS_l2_00009929	−
chr17	74788575	74799876	TCONS_00025497	+
chr15	34947820	34962370	TCONS_00023622	−
chr9	110761849	110769666	TCONS_00016127	+
chr4	158558839	158588196	TCONS_00007669	+
chr16	49488185	49494684	TCONS_00024390	+
chr1	244232122	244256328	TCONS_00000777	−
chr13	30677366	30683012	TCONS_00021510+TCONS_00021617	−
chr6	24797606	24799099	TCONS_l2_00024648	−
chr16	52305432	52314661	TCONS_00024411+TCONS_00024412+TCONS_00024413+TCONS_00024841+TCONS_00024842+TCONS_00024843+TCONS_00024414+TCONS_00024290+TCONS_00024845	+
chr22	45659261	45664778	TCONS_00029720	−
chr12	75626059	75645645	TCONS_00021162+TCONS_00021163+TCONS_00020190+TCONS_00021164	+
chr13	75126980	75131257	LINC00347	+

chr5	92906435	92910714	TCONS_00010741+TCONS_00010742+TCONS_00010743 +TCONS_00010744	+
chr14	38066369	38067552	TCONS_00022712	−
chr2	16060521	16076286	TCONS_00002830+TCONS_00004703	+
chr10	123495818	123499245	TCONS_00017883	+
chr9	2535655	2622373	FLJ35024	−
chr13	76587159	76588637	TCONS_00021570	+
chrX	38629584	38658911	TCONS_00016977	+
chr12	97623899	97651188	TCONS_00020889	−
chr5	26711660	26750106	TCONS_l2_00023695	−
chrX	40693887	40753995	TCONS_l2_00030181+TCONS_l2_00030182	+
chr1	108815565	108880726	TCONS_l2_00002124+TCONS_l2_00000473	+
chr1	244393073	244401962	TCONS_00001899	−
chr8	2447078	2480453	TCONS_00014506	−
chr13	114053727	114065280	TCONS_l2_00007341	−
chr2	54904602	54908989	TCONS_00003682	+
chr2	105810305	105813861	TCONS_00004364+TCONS_00005222	−
chr16	74226291	74250474	TCONS_00024274+TCONS_00025050+TCONS_00024727 +TCONS_00025051+TCONS_00024728	−
chr2	114879851	114880870	TCONS_00003384	−
chr2	105050805	105129215	LOC150568	+
chr2	47550560	47572162	TCONS_l2_00015793+TCONS_l2_00015794+TCONS_ l2_00014562+TCONS_l2_00014563	−
chr14	69283616	69284659	TCONS_00022775	−
chr1	189118917	189119860	TCONS_00001225	+
chr21	30868367	30872706	TCONS_00028831	+
chr3	167582561	167601170	TCONS_00005645+TCONS_00005646+TCONS_00005647 +TCONS_00006304	+
chr1	5621769	5728355	TCONS_00000077+TCONS_00000464	−
chr12	51567052	51568459	TCONS_00020424	+
chr2	136753804	136765112	TCONS_l2_00014003+TCONS_l2_00014004+TCONS_ l2_00015492+TCONS_l2_00014005	+
chr12	132641631	132642248	TCONS_00020660	+
chr7	29730428	29734203	TCONS_l2_00025774	+
chr1	5058062	5080898	TCONS_00001928+TCONS_00001929+TCONS_00001930 +TCONS_00001931+TCONS_00001932+TCONS_0000082 8+TCONS_00000829	+
chr7	124869633	124904345	TCONS_00012927	−
chr15	22541418	22546063	TCONS_00023573+TCONS_00023574+TCONS_00024033 +TCONS_00024034	−
chr4	58291143	58291939	TCONS_00008492	−
chr21	33937196	33940382	TCONS_l2_00017201	−
chr3	193296834	193310880	TCONS_l2_00019666+TCONS_l2_00019667	−
chr1	219092182	219098202	TCONS_00002231+TCONS_00001276+TCONS_00001277	+
chr1	81000663	81050048	TCONS_00000553	−
chr14	65170511	65170923	TCONS_00022769	−
chr12	8941101	8944136	TCONS_00020708	−
chr22	35107899	35120910	TCONS_00029477	−
chr3	148947143	148959226	TCONS_l2_00020216+TCONS_l2_00020217	−

chr6	26331953	26340507	TCONS_00011744+TCONS_00012439	+
chr2	193494645	193510432	TCONS_00003068	+
chr1	117838142	117863958	TCONS_00000283+TCONS_00002143	+
chr4	153955363	154012317	TCONS_00007662	+
chr11	20145064	20151881	TCONS_00019252	+
chr7	65564508	65565273	TCONS_00014317	−
chr6	10428018	10435055	LINC00518	−
chr6	170920275	170921789	TCONS_00012383	−
chr8	92072137	92082384	TCONS_l2_00028313+TCONS_l2_00028314+TCONS_l2_00028315	−
chr12	54356092	54368740	HOTAIR	−
chr14	85991477	85996332	TCONS_00022380	−
chr9	128819859	128921892	TCONS_00015752	+
chr7	17473626	17598533	TCONS_l2_00026398+TCONS_l2_00027281+TCONS_l2_00026399+TCONS_l2_00026400+TCONS_l2_00027282+TCONS_l2_00026401+TCONS_l2_00026402+TCONS_l2_00026403+TCONS_l2_00026404+TCONS_l2_00027283+TCONS_l2_00026405	−
chr5	113911738	113915667	TCONS_00009487	+
chr8	18949228	19002851	TCONS_00015242+TCONS_00014635+TCONS_00015243+TCONS_00014636+TCONS_00014637+TCONS_00015244	+
chr10	16320700	16337857	TCONS_00017792+TCONS_00017793	+
chr2	226264426	226265304	TCONS_00003118	+
chr19	23734519	23741642	TCONS_00026969+TCONS_00026970	+
chr8	92477067	92478128	TCONS_00015092	−
chr1	143118704	143211070	TCONS_l2_00002628+TCONS_l2_00002629+TCONS_l2_00002630+TCONS_l2_00002631+TCONS_l2_00002632+TCONS_l2_00002633+TCONS_l2_00002634+TCONS_l2_00001495	−
chr2	490845	492693	TCONS_00004093+TCONS_00003143	−
chr9	66514486	66522897	TCONS_l2_00029309+TCONS_l2_00029310	−
chr14	65708499	65714846	TCONS_00022770	−
chr22	21570320	21571057	TCONS_l2_00017908	−
chr15	99934870	99936875	TCONS_00023832	−
chr6	149912891	149913799	TCONS_00011593	−
chr7	39899608	39905982	TCONS_l2_00025816	+
chr6	80247285	80259817	TCONS_00012530+TCONS_00012532+TCONS_00012531+TCONS_00012533+TCONS_00012534+TCONS_00012535	+
chr2	663814	667014	TCONS_00003533+TCONS_00003534+TCONS_00004657+TCONS_00003535	+
chr2	65816700	65867311	TCONS_00003308	−
chr11	95431988	95432639	TCONS_00019448	+
chr8	125474740	125486804	TCONS_00014530	−
chr20	56174751	56176256	TCONS_00028073	−
chr4	125421097	125479589	TCONS_00007433	−
chr6	116222978	116247857	TCONS_00012777+TCONS_00012778	−
chr1	238110308	238164646	TCONS_l2_00000915	+
chr9	32643101	32645024	TCONS_l2_00029910	−

chr3	145657699	145679323	TCONS_00005604+TCONS_00005605	+
chr14	101540301	101544008	TCONS_00022414	−
chr22	32426317	32433930	TCONS_l2_00017736	+
chrY	9154670	9160483	RBMY1A3P	−
chrY	28425289	28475268	TCONS_l2_00030951+TCONS_l2_00030952+TCONS_l2_00030953	−
chrX	56755718	56844004	LOC550643	+
chr11	75469500	75479692	LOC283214	−
chr2	60586351	60618510	TCONS_l2_00015809+TCONS_l2_00014586+TCONS_l2_00015810	−
chr15	25355584	25361586	TCONS_00023883	+
chr4	29215886	29288006	TCONS_00008030+TCONS_00007501	+
chr5	108572821	108662070	TCONS_l2_00023858+TCONS_l2_00023048+TCONS_l2_00023049	−
chr20	23046042	23052105	TCONS_00028340	−
chr7	465456	468067	TCONS_00013289	+
chr9	41277733	41298538	TCONS_00015671+TCONS_00015672	+
chr12	114448893	114450445	TCONS_00020568	+
chr9	66707912	66711336	TCONS_l2_00029317	−
chr17	42723425	42725050	TCONS_00025662	−
chr3	193723150	193788780	TCONS_l2_00019673+TCONS_l2_00020303+TCONS_l2_00019674+TCONS_l2_00019675+TCONS_l2_00020304+TCONS_l2_00020305+TCONS_l2_00019676+TCONS_l2_00019680+TCONS_l2_00019679+TCONS_l2_00019678+TCONS_l2_00019677+TCONS_l2_00020306+TCONS_l2_00019681+TCONS_l2_00019682	−
chr10	1202378	1203685	TCONS_00018398	−
chr17	75249856	75266597	TCONS_00025500+TCONS_00025501+TCONS_00025982+TCONS_00025502	+
chr4	138586686	138601440	TCONS_00008260	+
chr1	207978671	207980881	TCONS_00002573	−
chr4	152808695	152812415	TCONS_00007879	−
chr14	87869204	87906600	TCONS_00022823	−
chr3	150588832	150612332	TCONS_l2_00020222	−
chr9	100638612	100658103	TCONS_00016434+TCONS_00016843	−
chr16	32881355	32884886	TCONS_l2_00009689+TCONS_l2_00009690	+
chr9	27608293	27609508	TCONS_l2_00029230+TCONS_l2_00029231	−
chr15	98097549	98102321	TCONS_00023821	−
chrY	6110487	6111651	TTTY23B	−
chr10	133730128	133733387	TCONS_00018649+TCONS_00018650	−
chr13	19940817	19955755	TCONS_l2_00006699+TCONS_l2_00006700+TCONS_l2_00006701+TCONS_l2_00006702	+
chr17	76982943	76987493	TCONS_00025519	+
chr5	173133322	173155306	TCONS_00011088+TCONS_00010559+TCONS_00010560+TCONS_00010561+TCONS_00011089+TCONS_00011090	−
chr4	144738948	144750505	TCONS_00007864	−
chr15	86296037	86300336	TCONS_00023766	−
chr16	75885329	75894749	TCONS_00024731	−
chr12	106642520	106646559	TCONS_00021206+TCONS_00021207	+

chrX	119251428	119253610	TCONS_00017536+TCONS_00017087+TCONS_00017537 +TCONS_00017538	−
chr8	29139378	29161475	TCONS_00014956	−
chr9	7960413	7961080	TCONS_00015792	−
chr10	38536850	38622083	TCONS_l2_00003944	+
chr7	80804827	80828289	TCONS_l2_00026075+TCONS_l2_00026076	+
chr4	184251303	184253742	TCONS_00008681	−
chr6	40132651	40139354	TCONS_00011811	+
chr18	15178761	15197762	TCONS_l2_00011993+TCONS_l2_00011994	−
chr19	23502462	23504093	TCONS_00026968	+
chr6	53488292	53489514	TCONS_00011842	+
chr1	232863000	232878755	TCONS_00000762+TCONS_00001871+TCONS_00001873 +TCONS_00001874	−
chr21	28577173	28603039	TCONS_00028980	+
chr4	75806109	75808265	TCONS_00008108	+
chr8	6261077	6264069	LOC100287015	−
chr6	166756119	166764957	LOC100289495	+
chr5	171906349	171935321	TCONS_00010167+TCONS_00010168	+
chr11	28724162	29085806	TCONS_l2_00005299+TCONS_l2_00005300+TCONS_ l2_00004461	+
chr4	152246102	152246795	TCONS_l2_00021353	−
chr2	779837	864112	LOC339822	−
chr13	110686399	110709651	TCONS_00022108+TCONS_00022109+TCONS_00022110 +TCONS_00022111+TCONS_00022310+TCONS_0002231 1+TCONS_00022112+TCONS_00022113	−
chr16	32769028	32769666	TCONS_l2_00009682	+
chr7	33900208	33917087	TCONS_00013408	+
chr5	176134357	176153244	TCONS_00009815	−
chr1	185527511	185590112	TCONS_00000349+TCONS_00002202	+
chr22	36841097	36851999	TCONS_00029693+TCONS_00029480	−
chr6	80017386	80023101	TCONS_l2_00025426	−
chr7	125071401	125121652	TCONS_l2_00027212+TCONS_l2_00026181+TCONS_ l2_00027213+TCONS_l2_00026182	+
chr3	127574634	127577830	TCONS_00006207	+
chrX	47657379	47670374	TCONS_l2_00030193	+
chr1	98389797	98394621	TCONS_00001602	−
chr1	32307620	32321169	TCONS_00000499	−
chr8	74817620	74827134	TCONS_l2_00028242+TCONS_l2_00028243	−
chr16	51231626	51239729	TCONS_00024644	−
chr2	70630066	70649810	TCONS_00002932	+
chr11	1923877	1929145	TCONS_00019134	+
chrY	2979379	2981965	TCONS_00017629	+
chr4	55199037	55224233	TCONS_00008792+TCONS_00007530	+
chr11	64834082	64835205	TCONS_00019123	−
chr8	2532111	2585957	TCONS_00014913+TCONS_00014507+TCONS_00015396	−
chr4	10760725	10810544	TCONS_00007985	+
chr4	111715559	111718500	TCONS_00007593	+
chr10	109631334	109829052	TCONS_00018040	−
chr5	88761965	88771325	TCONS_l2_00022430	+

chr13	40917903	40918383	TCONS_00021762	+
chr6	170475885	170478251	TCONS_l2_00024541+TCONS_l2_00025299	+
chr5	3178209	3181346	TCONS_00009330	+
chr11	63287301	63292203	TCONS_00019319	+
chr2	30147350	30152828	TCONS_00002862	+
chr18	32486955	32513606	TCONS_00026702+TCONS_00026483+TCONS_00026484	−
chr3	176321931	176354567	TCONS_00005886+TCONS_00007309+TCONS_00005887	−
chr12	120377247	120413171	TCONS_l2_00005930+TCONS_l2_00005931+TCONS_ l2_00005933+TCONS_l2_00005934	+
chr17	53290142	53323665	TCONS_00025435	+
chr9	75973785	75984932	TCONS_00016009+TCONS_00016010+TCONS_00016011	+
chr4	27209127	27220026	TCONS_00007735	−
chr10	133110511	133114038	TCONS_l2_00003329+TCONS_l2_00003330	+
chr21	20616277	20633315	TCONS_l2_00016983	+
chr9	76738436	76753959	TCONS_00016358	−
chr2	182547840	182550434	TCONS_00003064	+
chr12	68738446	68743558	TCONS_00021156	+
chr2	29319709	29320391	TCONS_00003245	−
chr11	107607278	107607590	TCONS_l2_00005184	−
chr4	129213906	129440563	TCONS_l2_00021700+TCONS_l2_00021701+TCONS_ l2_00021702+TCONS_l2_00021703+TCONS_ l2_00020755+TCONS_l2_00020756+TCONS_ l2_00020757+TCONS_l2_00020758+TCONS_ l2_00021704+TCONS_l2_00021705+TCONS_ l2_00020759+TCONS_l2_00020760+TCONS_l2_00020761	+
chr15	35014273	35016108	TCONS_00023344	+
chr11	23826000	23829778	TCONS_00019593	−
chr10	113109697	113111861	TCONS_00018049	−
chr4	119763229	119772488	TCONS_00008209	+
chr15	38431798	38519066	TCONS_l2_00009500+TCONS_l2_00009029	−
chr9	7304600	7343465	TCONS_00015916	+
chr2	30543397	30548751	TCONS_00003620	+
chr2	20724742	20739937	TCONS_00004164+TCONS_00004165+TCONS_00005098 +TCONS_00005099+TCONS_00005101+TCONS_0000416 6+TCONS_00005102+TCONS_00005103+TCONS_000051 04+TCONS_00005105+TCONS_00004167	−
chr3	34200826	34604551	TCONS_00005510+TCONS_00006813+TCONS_0 0005511+TCONS_00005982+TCONS_00006814+ TCONS_00005983+TCONS_00005512+TCONS_0 0006815+TCONS_00005984+TCONS_00005985+- TCONS_00005986+TCONS_00006816	+
chr8	21539899	21541011	TCONS_00014648	+
chr9	82120719	82182686	TCONS_00015836+TCONS_00016369+TCONS_00016370 +TCONS_00015837+TCONS_00016817	−
chr19	35391801	35396094	TCONS_00027312+TCONS_00027736+TCONS_00027314 +TCONS_00027313	−
chr20	4544975	4552736	TCONS_00028604+TCONS_00028299+TCONS_00028603	−
chr14	19894369	19904572	LOC101101776	+
chr5	178804601	178805760	TCONS_00010580	−
chr2	241166977	241195452	TCONS_00003521+TCONS_00004628	−

chr3	65093399	65094518	TCONS_00006525	−
chr7	85050438	85118609	TCONS_00013034+TCONS_00013035	+
chr10	135451047	135465409	TCONS_l2_00003340	+
chr8	82538937	82543901	TCONS_l2_00028267	−
chr2	78769292	78793155	TCONS_00004830	+
chr6	161216349	161228213	TCONS_00012350	−
chr4	66567920	66571271	TCONS_00007545	+
chr13	46852591	46853940	TCONS_00021994	−
chr8	8844846	8846067	TCONS_00014926	−
chr22	49441051	49446197	TCONS_00029735	−
chr3	81811857	81813907	TCONS_00005794	−
chr7	35985936	36013288	TCONS_l2_00025794+TCONS_l2_00025795	+
chr9	70654323	70662639	TCONS_l2_00028818	+
chr7	12907230	12913136	TCONS_00013699	−
chr10	6816116	6821160	TCONS_00018425	−
chr1	90254473	90256051	TCONS_00000245	+
chr20	17115519	17133156	TCONS_00028114	+
chr22	27703444	27714499	TCONS_00029461	−
chr15	74267677	74268524	TCONS_00023462	+
chr2	8007544	8009432	TCONS_00004125	−
chr19	20845800	20857891	TCONS_00026943	+
chr9	91825615	91826830	TCONS_00016056	+
chr10	46349534	46387931	TCONS_l2_00004166	−
chr5	77638189	77656217	TCONS_00009709+TCONS_00009291	−
chr3	33002605	33016096	TCONS_00006466	−
chr17	2615677	2616453	TCONS_00025273	+
chr18	9646153	9648542	TCONS_00026276	+
chr3	191469931	191500590	TCONS_00006737	−
chr2	156329468	156346085	TCONS_00003908+TCONS_00003909	+
chr2	5848371	5859865	TCONS_00004111	−
chr5	39520511	39524809	TCONS_00009927+TCONS_00010670	+
chr8	63924580	63925933	TCONS_00014734	+
chr4	64328225	64359996	TCONS_00008497+TCONS_00009065+TCONS_00007774	−
chr12	91720964	91756189	TCONS_00020516	+
chr6	108444719	108479222	TCONS_00011349+TCONS_00011185	+
chr1	63489878	63491329	TCONS_00000216	+
chr2	44286099	44294916	TCONS_l2_00015780	−
chr14	77352715	77371404	TCONS_l2_00008156	−
chr3	69640492	69642272	TCONS_00005538	+
chr7	16955704	17061246	TCONS_l2_00025688+TCONS_l2_00025689+TCONS_ l2_00025690+TCONS_l2_00025691+TCONS_ l2_00025692+TCONS_l2_00025693	+
chr10	132011022	132050307	TCONS_00018386+TCONS_00018387+TCONS_00018388	+
chr19	7837320	7850059	TCONS_l2_00012262	+
chr3	153102723	153105157	TCONS_00005471	−
chr17	80172103	80175228	TCONS_00025256	−
chr6	27012402	27026687	TCONS_00011755	+
chr8	9208767	9217269	TCONS_00014929	−

chr18	14179096	14227049	ANKRD20A5P	+
chr9	68411270	68418932	TCONS_l2_00029343	−
chr18	35461781	35462430	TCONS_00026325	+
chr7	82984470	82991582	TCONS_00013032	+
chr14	31959233	31995393	TCONS_00022458	+
chr5	3452930	3461774	TCONS_00009331+TCONS_00009851	+
chr4	26072413	26105853	TCONS_00008014+TCONS_00008769	+
chr13	27810419	27810948	TCONS_00021615	−
chr13	111474463	111491493	TCONS_00021904+TCONS_00021505+TCONS_00021905	+
chr6	114736744	114777802	TCONS_00011932+TCONS_00011933	+
chr5	115040463	115044690	TCONS_00010061	+
chr10	2616301	2620226	TCONS_00018404	−
chr12	7491434	7494514	TCONS_00021032	+
chr4	13492921	13520604	TCONS_00007998+TCONS_00008763	+
chr9	38949737	38998487	TCONS_00015979+TCONS_00016579+TCONS_00016580 +TCONS_00015980	+
chr2	15941871	15951001	TCONS_00003211	−
chr17	14629046	14633003	TCONS_00025158	+
chr5	87008952	87029086	TCONS_00009717	−
chr5	106150898	106346937	TCONS_00009748+TCONS_00010435+TCONS_00011014 +TCONS_00009749	−
chr1	230728406	230731329	TCONS_00000415	+
chr3	166255715	166261364	TCONS_00006302	+
chr5	67944452	67951025	TCONS_00009988+TCONS_00009989	+
chr7	9775337	9809237	TCONS_l2_00025652+TCONS_l2_00027046	+
chr2	52918447	52925210	TCONS_00004253	−
chr19	49521505	49522741	LOC101059948	−
chr2	9283295	9286235	TCONS_00002815	+
chr2	84542232	84577898	TCONS_l2_00015394	+
chrX	52651985	52662998	SSX8	+
chr5	179894886	179910503	TCONS_00010211	+
chr17	38957676	38965769	TCONS_00025387	+
chr12	54121449	54125008	TCONS_00020433+TCONS_00021137+TCONS_00021138	+
chr2	11566019	11566997	TCONS_00003578	+
chr7	30216003	30218630	TCONS_00013148	−
chr18	72877670	72883527	TCONS_00026566+TCONS_00026567+TCONS_00026739	−
chr5	180111766	180113546	TCONS_00009564	+
chr2	114425313	114426158	TCONS_l2_00014840	−
chr2	43055063	43180319	TCONS_00003658+TCONS_00003659+TCONS_00003660	+
chrX	63264305	63265448	TCONS_l2_00030233	+
chr15	82391267	82393834	TCONS_00023486	+
chr6	4399913	4401474	TCONS_00012060	−
chr7	63093061	63096994	TCONS_l2_00025915+TCONS_l2_00025916	+
chr3	78071949	78079091	TCONS_00006545	−
chr10	46798370	46809021	TCONS_00017969	−
chr13	66569686	66628131	TCONS_00021815+TCONS_00021816	+
chr10	21702311	21703628	TCONS_00018449	−
chr1	30159178	30161573	TCONS_00000876	+

chr8	43096867	43097508	TCONS_00014997	−
chrX	26704160	26706126	TCONS_l2_00030166+TCONS_l2_00030167	+
chr16	61263482	61264311	TCONS_00024694	−
chr3	96335999	96337060	TCONS_00007196+TCONS_00007197+TCONS_00007198 +TCONS_00006556	−
chr8	112754207	112770625	TCONS_00014833+TCONS_00014834+TCONS_00014835	+
chr3	141468729	141493540	TCONS_00006247	+
chr18	2636526	2637206	TCONS_00026236	+
chr19	52196593	52208443	LINC00085	+
chr14	94449442	94453395	TCONS_00022840	−
chrX	125606112	125607249	TCONS_l2_00030350	+
chr8	129571390	129576800	TCONS_00014537+TCONS_00015175	−
chr12	49681292	49683068	TCONS_00020779	−
chr2	65952596	65953735	TCONS_00004290+TCONS_00004291	−
chr1	83982445	84039951	TCONS_00001008	+
chrX	106756213	106789051	TCONS_00017080	−
chr4	75808588	75828412	TCONS_l2_00020579+TCONS_l2_00020580	+
chr11	30878802	30880597	TCONS_00019603	−
chr4	570752	617100	TCONS_00008727+TCONS_00008728+TCONS_00007 943+TCONS_00007944+TCONS_00007945+TCONS_0 0007946+TCONS_00007947+TCONS_00007948+TCO NS_00008729	+
chrX	48074036	48077405	TCONS_l2_00030195	+
chr1	53904043	53905693	SLC25A3P1	−
chr12	128115820	128120990	TCONS_00021447+TCONS_00020986+TCONS_00021448 +TCONS_00020987+TCONS_00021449	−
chr5	7344692	7346034	TCONS_00009343	+
chr1	177669659	177679465	TCONS_00000341	+
chr1	13983806	13987033	TCONS_00000470	−
chr22	38420126	38439988	TCONS_00029593+TCONS_00029594+TCONS_00029595	+
chr3	59365750	59374430	TCONS_00005779	−
chr3	127041151	127109513	TCONS_00005467+TCONS_00006643+TCONS_00006644	−
chr11	69680126	69681839	TCONS_00019371	+
chr14	83108883	83117334	TCONS_00022567	+
chr6	43812608	43820084	TCONS_00011216+TCONS_00012175+TCONS_00012722 +TCONS_00012723+TCONS_00011494	−
chr20	38414363	38441871	TCONS_00027927	+
chr12	65277554	65371302	FLJ41278	+
chr3	136977158	137039591	TCONS_00006675	−
chr22	36784610	36806108	TCONS_00029589+TCONS_00029419	+
chr15	97972959	97975172	TCONS_00024018	+
chr17	38850769	38854069	TCONS_00025386	+
chr12	10705979	10710816	TCONS_00021063	+
chr4	12860725	12866585	TCONS_00007717	−
chr8	118652591	118695922	TCONS_00015145+TCONS_00015146	−
chr4	172523834	172577960	TCONS_00007898+TCONS_00008644	−
chr13	21872264	21967061	MIPEPP3	+
chr20	49012776	49013558	TCONS_00028216	+
chr7	46341718	46383219	TCONS_00013006	+

chr16	85722932	85733297	TCONS_00024754	−
chr11	50238999	50257633	LOC441601	−
chr7	419391	422845	LOC442497	+
chr17	19483256	19537652	TCONS_l2_00011530+TCONS_l2_00011531+TCONS_l2_00010601+TCONS_l2_00010602+TCONS_l2_00010603+TCONS_l2_00010604+TCONS_l2_00010605+TCONS_l2_00010606+TCONS_l2_00010607	+
chr10	82093527	82094182	TCONS_00017846	+
chr20	62110073	62111644	TCONS_00028472	−
chr2	147595317	147607878	TCONS_00003027	+
chr5	8839844	8881632	TCONS_00009348+TCONS_00009235	+
chr1	153146994	153164152	TCONS_00000314	+
chr6	153678484	153688435	TCONS_00011993	+
chr2	182555107	182558142	TCONS_00003462	−
chr11	113887644	113888813	TCONS_00019764	−
chr16	15016370	15017566	TCONS_l2_00009606	+
chr2	114464370	114467816	TCONS_00003380	−
chr14	74674902	74684477	TCONS_00022796+TCONS_00023132	−
chr9	88068944	88069347	TCONS_00016037	+
chr16	54399916	54404348	TCONS_00025011+TCONS_00025012	−
chr13	88096242	88323528	TCONS_00021476+TCONS_00021678+TCONS_00022080+TCONS_00021679	−
chr9	102153603	102161927	TCONS_00016437	−
chr4	132925965	133045186	TCONS_00007831+TCONS_00007832	−
chr13	21512703	21523567	TCONS_l2_00007488	−
chr11	62178650	62179162	TCONS_00019652	−
chr14	105543608	105543911	TCONS_00022649	+
chr2	67267678	67523207	TCONS_l2_00015367+TCONS_l2_00013697+TCONS_00003724+TCONS_00002705+TCONS_00002925+TCONS_l2_00015369+TCONS_00002927	+
chr6	131910	148170	TCONS_l2_00025304+TCONS_l2_00024548	−
chr16	65166562	65173706	TCONS_00024436+TCONS_00024861	+
chr6	153188277	153188667	TCONS_00012335	−
chr1	238648745	238701605	TCONS_l2_00002344	+
chr7	119344610	119547429	TCONS_00013242+TCONS_00013243	−
chr18	19745127	19745640	TCONS_00026443	−
chr9	81484525	81496839	TCONS_00016364+TCONS_00016365	−
chr9	79571754	79603477	TCONS_l2_00028853	+
chrX	75364973	75368267	TCONS_00017346+TCONS_00017347	−
chr4	140539293	140544386	TCONS_00007858	−
chr6	171045718	171055061	TCONS_l2_00024547	+
chr6	28953518	28959134	TCONS_l2_00024132+TCONS_l2_00024133+TCONS_l2_00025115+TCONS_l2_00025116	+
chr5	1363706	1380166	TCONS_l2_00022745+TCONS_l2_00022744+TCONS_l2_00023632	−
chr2	49141327	49171820	TCONS_l2_00014567	−
chr3	134294594	134299804	TCONS_00006666	−
chr2	177714314	177722672	TCONS_00003951	+
chr4	185262184	185275130	LOC728175	−

chr16	432241	442960	LOC100134368	+
chr22	21457305	21476575	BCRP2	+
chrX	145997665	146036027	TCONS_00017254+TCONS_00017469	+
chr13	45948379	45957978	TCONS_00021992+TCONS_00022270+TCONS_00022271 +TCONS_00022272	−
chr6	13328900	13330028	TCONS_00011697	+
chr17	4062960	4063932	TCONS_00026020	−
chr5	45227480	45228451	TCONS_00009956	+
chr1	77223809	77244952	TCONS_00000550	−
chr16	87157905	87158268	TCONS_00024775	−
chr7	109162038	109237223	TCONS_00013224	−
chr2	154122026	154122587	TCONS_00003906	+
chr13	111969464	111972339	TCONS_00022315	−
chr10	91583143	91585093	TCONS_00018995	−
chr4	178134766	178222346	TCONS_00009176+TCONS_00009177+TCONS_00008667 +TCONS_00008668	−
chr1	119805547	119818393	TCONS_00001129	+
chr6	35511376	35514379	TCONS_l2_00024192+TCONS_l2_00025142+TCONS_ l2_00024193	+
chr13	22225659	22245083	TCONS_00021936	−
chr13	77558949	77564770	TCONS_l2_00006925	+
chr1	88994518	88995833	TCONS_00001565+TCONS_00001566	−
chr5	72542337	72570767	TCONS_00010961+TCONS_00010962	−
chr21	16189151	16254319	TCONS_l2_00017147+TCONS_l2_00017148+TCONS_ l2_00017149+TCONS_l2_00017150+TCONS_ l2_00017151+TCONS_l2_00017429	−
chr7	65292534	65293652	TCONS_00014153	+
chr13	63246418	63306867	TCONS_l2_00007561+TCONS_l2_00007227+TCONS_ l2_00007562	−
chr2	52600256	52617150	TCONS_00002892	+
chr1	249153363	249158995	TCONS_00000435	+
chr14	70892663	70922152	TCONS_00023123+TCONS_00022777	−
chr20	23160769	23170944	TCONS_00028518+TCONS_00028519+TCONS_00028130 +TCONS_00028131+TCONS_00027914	+
chr14	100070893	100092074	TCONS_00022400	+
chr4	186491250	186499648	TCONS_00008697	−
chr5	115292235	115297015	TCONS_00010065+TCONS_00010773+TCONS_00010774	+
chr7	141870970	141923474	TCONS_00012852+TCONS_00013611+TCONS_00014223	+
chr17	41754165	41768355	TCONS_00025656	−
chr8	133508866	133513616	TCONS_00014861	+
chrX	13066231	13104187	TCONS_00017139	+
chr12	93484265	93486711	TCONS_00020520+TCONS_00020521	+
chr20	7126793	7238913	TCONS_00028312+TCONS_00027998+TCONS_00028313 +TCONS_00027999	−
chr11	44545397	44547608	TCONS_00019626	−
chr1	102665295	102855186	TCONS_00000590+TCONS_00001611+TCONS_00001612 +TCONS_00001613	−
chr15	95619720	95622243	TCONS_00023529	+
chr6	31165537	31171745	HCG27	+

chr8	8077586	8085905	TCONS_00014923+TCONS_00015403+TCONS_00014924	−
chr17	46036701	46037822	TCONS_00025685	−
chr5	135148256	135162271	TCONS_00010100+TCONS_00009520+TCONS_00010791+TCONS_00010792+TCONS_00010793+TCONS_00010101+TCONS_00010794+TCONS_00010795	+
chr9	116072041	116072854	TCONS_00016132	+
chr21	47013568	47017005	TCONS_00028861+TCONS_00028862	+
chr10	22760544	22762101	TCONS_00018135	+
chr5	43336266	43348818	TCONS_l2_00023383	+
chr8	93470514	93513128	TCONS_00015478+TCONS_00015093+TCONS_00015094+TCONS_00015479	−
chr21	30365399	30366528	TCONS_00028986+TCONS_00029209	+
chr2	130750494	130751706	TCONS_l2_00013981	+
chr10	29032481	29046401	TCONS_00018145+TCONS_00018691+TCONS_00017803+TCONS_00018692	+
chr7	22450336	22452159	TCONS_00013372	+
chr9	39925026	39926970	TCONS_00015809	−
chr16	13925217	13929301	TCONS_00024333	+
chr2	177710721	177714156	TCONS_00003454+TCONS_00003455	−
chr5	123791066	123795791	TCONS_00009500	+
chr16	56126899	56225006	LOC283856	−
chr21	16741148	16776189	TCONS_l2_00017152+TCONS_l2_00017153+TCONS_l2_00017430+TCONS_l2_00017154	−
chr4	17484658	17487209	TCONS_00007487	+
chr3	95888	111980	TCONS_00006777+TCONS_00005936+TCONS_00006778	+
chr6	89830998	89853352	TCONS_00012543	+
chr2	114298969	114328105	TCONS_l2_00013918+TCONS_l2_00013919+TCONS_l2_00013920	+
chr3	181670146	181728454	TCONS_l2_00019023+TCONS_l2_00019024+TCONS_l2_00019995+TCONS_l2_00019996+TCONS_l2_00019997+TCONS_l2_00019998+TCONS_l2_00019025+TCONS_l2_00019999	+
chr2	81152586	81155939	TCONS_00004319	−
chr19	56116597	56130307	TCONS_l2_00012717+TCONS_l2_00012718+TCONS_l2_00012719	+
chr6	106060542	106080071	TCONS_00011530	−
chr12	116797182	116797559	TCONS_00020580	+
chr11	49010697	49012724	TCONS_l2_00004999	−
chr4	49310505	49513303	TCONS_l2_00021133+TCONS_l2_00021878	−
chr6	77296178	77303510	TCONS_00011862	+
chr9	23500690	23672397	TCONS_l2_00029907+TCONS_l2_00029224+TCONS_l2_00029908	−
chr4	184687063	184688453	TCONS_00008357	+
chr11	134306376	134375555	LOC283177	+
chr12	131254365	131260323	TCONS_00021005	−
chr7	29685425	29699931	TCONS_l2_00025769+TCONS_l2_00025770	+
chr1	64652512	64657832	TCONS_00000970	+
chr1	152627953	152629280	TCONS_00000313	+
chr5	2831135	2835253	TCONS_00009583	−
chr8	38721756	38725271	TCONS_00014980	−

chr9	40715524	40722679	FAM74A3	+
chr3	187513975	187523046	TCONS_00005687+TCONS_00005688	+
chr5	33519721	33522335	TCONS_00009634	−
chr1	53793905	53802890	TCONS_00000041+TCONS_00000199	+
chr13	30914407	30948036	LINC00426	−
chr12	67485325	67490160	TCONS_00020822	−
chr1	47308767	47366147	CYP4Z2P	−
chr5	68263568	68325992	TCONS_00009687+TCONS_00009285+TCONS_00010364 +TCONS_00009688	−
chr5	172720132	172721732	TCONS_00009813	−
chr22	18958011	18982142	DGCR5	+
chr4	106058437	106061776	TCONS_00007807	−
chr7	42701325	42746046	TCONS_l2_00027334+TCONS_l2_00026521	−
chr21	44869904	44873771	LINC00319	+
chr10	89739717	89740717	TCONS_00018266	+
chrX	53122892	53124040	TCONS_00017061	−
chr5	89454156	89585870	TCONS_l2_00023813+TCONS_l2_00022996+TCONS_ l2_00022997+TCONS_l2_00022998	−
chr11	115754860	115757277	TCONS_00019769	−
chr7	56718266	56730711	TCONS_l2_00026592	−
chr5	163151152	163158626	TCONS_00010162	+
chr12	4672223	4675732	TCONS_00021025	+
chr2	9250563	9257183	TCONS_l2_00013455+TCONS_l2_00013456+TCONS_ l2_00015236	+
chr13	64560504	64650144	TCONS_l2_00007568+TCONS_l2_00007569	−
chr11	34592489	34600868	TCONS_l2_00004475	+
chr7	106102459	106280354	TCONS_00013220+TCONS_00013221	−
chr21	20325582	20339376	TCONS_00028877	−
chr16	4295826	4303790	LOC100507501	−
chr18	56498966	56501573	TCONS_00026345	+
chr16	55461463	55478095	TCONS_00024667+TCONS_00024668	−
chr13	27746396	27757135	TCONS_00021532	+
chr5	4773620	4775065	TCONS_00009853+TCONS_00009232	+
chr19	23101230	23103160	TCONS_00026960	+
chr2	8683722	8723922	TCONS_00003197+TCONS_l2_00015704+TCONS_ l2_00014416+TCONS_l2_00015705+TCONS_l2_00014417	−
chr18	14145	16900	TCONS_00026387+TCONS_00026388	−
chr12	9475632	9477188	TCONS_00021285	−
chr9	90489018	90493879	TCONS_00016047+TCONS_00016048	+
chr11	18283800	18284297	TCONS_00020046	−
chr2	71251205	71257060	OR7E91P	+
chr3	149095953	149104368	TCONS_00006264+TCONS_00005618	+
chr12	132642562	132642945	TCONS_00021012	−
chr6	161236582	161238383	TCONS_00011606	−
chr18	51882005	51884231	TCONS_00026716+TCONS_00026715	−
chr2	83749938	83750626	TCONS_00002943	+
chr17	44907958	44910608	TCONS_00025674	−

chr5	141704858	141909695	TCONS_l2_00023557+TCONS_l2_00023558+TCONS_l2_00022553+TCONS_00010121+TCONS_l2_00022555+TCONS_l2_00023559	+
chr14	21189090	21190291	TCONS_l2_00007972	−
chr5	1887446	1900607	TCONS_00009325	+
chr2	7199703	7218093	TCONS_l2_00014406+TCONS_l2_00015692+TCONS_l2_00014407	−
chr19	22701232	22703274	TCONS_00026955	+
chr15	60544653	60589787	TCONS_00023428	+
chr19	48824029	48827644	TCONS_00027388	−
chr6	4490201	4495989	TCONS_00011659	+
chr4	12949198	12950294	TCONS_00007718	−
chr2	23570525	23574735	TCONS_00002854	+
chr19	43877100	43878596	TCONS_l2_00013127+TCONS_l2_00013128	−
chr11	82805693	82808326	TCONS_00019720	−
chr2	224363491	224369329	TCONS_l2_00015611	+
chr7	93701071	93706469	TCONS_00013207	−
chr9	101945655	101947173	TCONS_00015723	+
chr9	6408525	6411865	TCONS_00016264	−
chrY	9187189	9362877	FAM197Y5	−
chr18	21978415	21982646	TCONS_00026297	+
chr15	80665712	80696455	TCONS_00023755+TCONS_00024186+TCONS_00024187	−
chr15	81952145	81954442	TCONS_00023756	−
chr9	95432687	95435900	TCONS_00016073	+
chr22	16062157	16063236	TCONS_00029356	+
chr2	6968645	6973662	TCONS_00003184+TCONS_00004118	−
chr1	59592661	59598505	TCONS_00000963	+
chr5	50668571	50679166	LOC642366	−
chr16	50913948	50918656	TCONS_00024393+TCONS_00024394	+
chr6	36907863	36912451	TCONS_00011299	+
chr6	139348761	139349782	TCONS_00012612	+
chr11	77350820	77352358	TCONS_00019400	+
chrX	114928845	114930778	TCONS_00017364	−
chr7	62505205	62514809	TCONS_00013176	−
chr8	135845015	135855171	TCONS_00014866+TCONS_00015371+TCONS_00015372+TCONS_00014867+TCONS_00015373	+
chr5	59783540	59843484	PART1	+
chrY	15863608	15983586	TCONS_l2_00030899+TCONS_l2_00030994+TCONS_l2_00030995+TCONS_l2_00030900+TCONS_l2_00030901	+
chr4	146502620	146509110	TCONS_00008606	−
chr2	28931819	28959071	TCONS_00003242+TCONS_00005119+TCONS_00004187+TCONS_00003243+TCONS_00003244+TCONS_00005120	−
chr9	71158457	71161505	TCONS_00015828+TCONS_00015829	−
chr3	149101966	149103587	TCONS_00005845	−
chr12	81156933	81164492	TCONS_00020191+TCONS_00020496	+
chr4	164029937	164041790	TCONS_00007673+TCONS_00008310+TCONS_00008311+TCONS_00008312+TCONS_00007674	+
chr18	12438422	12439075	TCONS_00026290	+
chr7	10489448	10601205	TCONS_l2_00027270	−

chr15	83379223	83382745	LOC338963	−
chr10	87192619	87204685	TCONS_00017849+TCONS_00018257	+
chr17	53588361	53609981	TCONS_l2_00011353	−
chr15	45127378	45154015	TCONS_l2_00009082+TCONS_l2_00009083	−
chr10	119321595	119331968	TCONS_00018057	−
chr7	135710489	135769416	TCONS_00013606+TCONS_00013085	+
chr9	79168048	79179658	TCONS_00016020	+
chr1	224623157	224624723	TCONS_00001840	−
chr13	54916093	54927951	TCONS_00021796	+
chr13	69885465	69896233	TCONS_00021564	+
chr9	12098660	12159147	TCONS_00015794+TCONS_00016273	−
chr14	57265363	57266023	TCONS_00022519	+
chr6	168224570	168227476	MLLT4-AS1	−
chr2	132715850	132723108	TCONS_00003006	+
chr4	95596396	95623721	TCONS_00007574	+
chr7	75795969	75807924	TCONS_l2_00026015+TCONS_l2_00026016+TCONS_l2_00026017+TCONS_l2_00026018+TCONS_l2_00027139	+
chr5	147729741	147763363	TCONS_00010529	−
chr9	113901526	114047138	TCONS_l2_00030061+TCONS_l2_00029558+TCONS_l2_00029559	−
chr7	135853	149532	TCONS_00013111+TCONS_00013665+TCONS_00013112+TCONS_00013666+TCONS_00014244+TCONS_00013667+TCONS_00013669+TCONS_00013668+TCONS_00013670+TCONS_00014245+-TCONS_00013113+TCONS_00013114	−
chr15	51132073	51200796	TCONS_00023247+TCONS_00023277+TCONS_00023677	−
chr9	91586553	91591213	TCONS_00015704	+
chr13	75017246	75018926	TCONS_00022202+TCONS_00021833	+
chr17	55122438	55128557	TCONS_00025701	−
chr3	127256956	127259967	TCONS_00006963	+
chr16	88210869	88220350	TCONS_00024781+TCONS_00025093+TCONS_00024782+TCONS_00024783+TCONS_00025094+TCONS_00024784+TCONS_00024785	−
chr12	49696891	49697718	TCONS_00020780	−
chr2	41370920	41383954	TCONS_l2_00015313	+
chr22	27028314	27042295	TCONS_l2_00017992	−
chr15	100882802	100892899	TCONS_00023557+TCONS_00023558+TCONS_00024026+TCONS_00023559	+
chrX	73167080	73225243	TCONS_00017518	−
chr9	42411969	42435225	TCONS_l2_00029722+TCONS_l2_00029723	+
chr15	95822519	95870329	LOC400456	−
chr6	119773712	119812467	LOC285762	−
chr7	125870287	125924312	TCONS_00013583+TCONS_00013582+TCONS_00014207	+
chr17	48074253	48094208	TCONS_00025183	+
chr9	90750371	90755756	TCONS_l2_00029454	−
chr2	58747888	59290901	FLJ30838	+
chr16	87615694	87618184	TCONS_00024503	+
chr5	157897831	157905362	TCONS_00010536	−
chr20	23969936	23976596	TCONS_l2_00016200	+

chr15	98538125	98561008	TCONS_00024212+TCONS_00023824+TCONS_00023825+TCONS_00024213	−
chr11	68039587	68052321	TCONS_00019358+TCONS_00019359+TCONS_00019940+TCONS_00019360	+
chr16	87806997	87812948	TCONS_00024778+TCONS_00024779	−
chr13	74125367	74162782	TCONS_l2_00006907+TCONS_l2_00007425	+
chr2	111160496	111230652	LIMS3-LOC440895	−
chr3	46876675	46888307	TCONS_00006505	−
chr11	119897461	119899696	TCONS_00019503+TCONS_00020006+TCONS_00019504+TCONS_00019505	+
chr1	148928286	148953054	LOC645166	+
chr13	23733391	23733727	TCONS_00021727	+
chr17	81174666	81188573	FLJ43681	+
chr16	65175263	65210664	TCONS_00024696+TCONS_00025033+TCONS_00025032	−
chr5	175570088	175626298	LOC643201	−
chr3	153480918	153482081	TCONS_00007011	+
chr9	138054581	138057152	TCONS_00016213	+
chr2	150683558	150704743	TCONS_00003900	+
chrX	53187817	53200365	TCONS_l2_00030495	−
chr17	80662013	80674131	TCONS_00025788+TCONS_00025789+TCONS_00025257	−
chr6	134846174	135028487	TCONS_l2_00024926+TCONS_l2_00024927+TCONS_l2_00025478+TCONS_l2_00025479+TCONS_l2_00025480+TCONS_l2_00024928+TCONS_l2_00024929+TCONS_l2_00024930+TCONS_l2_00025481+TCONS_l2_00025482	−
chr10	45432956	45451176	TCONS_00017823+TCONS_00018192	+
chr4	130220330	130226177	TCONS_00007827	−
chr1	243192814	243215554	TCONS_l2_00002811+TCONS_l2_00002812+TCONS_l2_00002813+TCONS_l2_00002814	−
chr15	82109566	82110703	TCONS_00023757	−
chr6	169754595	169789822	TCONS_00012369+TCONS_00011626+TCONS_00011247	−
chrX	64254714	64272117	TCONS_00017186	+
chr2	105199610	105201812	TCONS_00002960	+
chr7	149097269	149097769	TCONS_00013995	−
chr19	19867181	19887222	LINC00663	−
chr4	182741172	182751126	TCONS_00007919	−
chr9	71925	88826	TCONS_l2_00028589+TCONS_l2_00028590+TCONS_l2_00028591+TCONS_l2_00029669+TCONS_l2_00029670+TCONS_l2_00028592	+
chr1	87992047	87995624	TCONS_l2_00001335	−
chr2	169105104	169121783	TCONS_00004943	+
chr12	116971227	116974318	LINC00173	+
chr1	25370198	25439611	TCONS_00000488+TCONS_00000489	−
chr1	2730997	2731563	TCONS_00000458	−
chr19	15939757	15947131	UCA1	+
chr19	5558178	5568005	TINCR	−
chr4	181652317	181680191	TCONS_00007917	−
chr5	75075708	75158886	TCONS_l2_00022964	−
chr4	135680554	135738444	TCONS_00007628	+

chr12	4071057	4106135	TCONS_00020679	−
chr2	124179064	124203757	TCONS_00003849	+
chr1	637316	659930	TCONS_l2_00002386+TCONS_l2_00002387+TCONS_l2_00002388+TCONS_l2_00002389	−
chr2	36581892	36582713	LOC100288911	−
chr20	57393973	57425958	GNAS-AS1	−
chr7	72299952	72307978	SBDSP1	+
chr3	159924296	159943086	TCONS_00005864	−
chr4	26846790	26860575	TCONS_00008018+TCONS_00008019+TCONS_00008020+TCONS_00008770	+
chr22	16414984	16415911	TCONS_l2_00017540	+
chr11	71213028	71213692	TCONS_00019696	−
chr7	25805268	25807263	TCONS_00014088	+
chr7	129780903	129782455	TCONS_00013078+TCONS_00013079	+
chr20	62921738	62934707	LINC00266-1	+
chr19	10349736	10361691	TCONS_00027180	−
chr6	96461406	96462915	TCONS_00012231	−
chr3	73857752	73952017	TCONS_00005543	+
chr6	34203398	34204471	TCONS_00011790	+
chr4	152326614	152330005	TCONS_00008621+TCONS_00009146+TCONS_00008622+TCONS_00007878	−
chr5	74244127	74271554	TCONS_00010716	+
chr4	18489685	18491131	TCONS_00007724	−
chr11	105328612	105386680	TCONS_00019746	−
chr4	24473715	24474219	TCONS_00008449	−
chr2	200732121	200775905	TCONS_l2_00015065+TCONS_l2_00016063+TCONS_l2_00016064+TCONS_l2_00015066+TCONS_l2_00015067+TCONS_l2_00016065+TCONS_l2_00016066+TCONS_l2_00015068+TCONS_l2_00015069	−
chr2	40973627	40994592	TCONS_l2_00015312+TCONS_l2_00013593+TCONS_l2_00013594+TCONS_l2_00013595	+
chr1	2499407	2500424	TCONS_00001378	−
chr1	189744595	189784048	TCONS_00000355	+
chr4	185910151	185926340	TCONS_00007689	+
chr6	34247383	34249098	TCONS_00012702	−
chr19	33177954	33182862	TCONS_00027301	−
chr18	72877670	72892146	TCONS_00026369+TCONS_00026641+TCONS_00026642+TCONS_00026643	+
chr2	35223345	35293597	TCONS_l2_00015761+TCONS_l2_00014507+TCONS_l2_00014508	−
chr6	141724594	141724930	TCONS_00011974	+
chr5	1856084	1856682	TCONS_00009581	−
chr10	103044290	103045131	TCONS_00018312	+
chr8	58375466	58405506	TCONS_00014718	+
chr19	42656721	42661675	TCONS_00026792	+
chr10	38742109	38764837	TCONS_l2_00003949+TCONS_l2_00003950	+
chr7	149095295	149098547	TCONS_00013092	+
chr3	50300398	50304841	TCONS_00006840+TCONS_00006841	+
chr4	26789737	26790828	TCONS_00008017	+

chr5	150554213	150556044	TCONS_00011070	−
chr2	145279356	145337001	TCONS_l2_00014013+TCONS_l2_00015502	+
chr14	19670792	19681016	LINC00516	−
chr2	37552100	37553940	TCONS_00003636+TCONS_00004749+TCONS_00002872	+
chr8	37374023	37386629	TCONS_00014976	−
chr6	169116745	169123604	TCONS_00011419	+
chr8	107157947	107167702	TCONS_00015133	−
chr1	55681081	55683128	LOC100507634	+
chr6	148476261	148478197	TCONS_00012328	−
chr4	69049896	69051921	TCONS_l2_00021898	−
chr11	43590813	43591947	TCONS_l2_00004492	+
chr19	40018980	40019553	TCONS_00027541	+
chr18	109065	122222	ROCK1P1	+
chr11	124981873	124997298	TCONS_l2_00004830+TCONS_l2_00004831	+
chr16	53080976	53087097	TCONS_l2_00010169	−
chr5	101917072	101953293	TCONS_00009475+TCONS_00009476	+
chr6	42423793	42424759	TCONS_00011816	+
chr8	105318737	105331133	TCONS_00015131	−
chrX	118380534	118400772	TCONS_l2_00030583+TCONS_l2_00030847	−
chrX	75876640	75913441	TCONS_l2_00030542	−
chr7	64103412	64116285	TCONS_00013480+TCONS_00013481	+
chr1	37568178	37618282	TCONS_l2_00001147+TCONS_l2_00001148	−
chr8	99374348	99401181	TCONS_00015112	−
chrX	89294189	89295476	TCONS_l2_00030550	−
chrX	152661098	152666834	TCONS_l2_00030633+TCONS_l2_00030877	−
chr11	131074894	131085058	TCONS_00019159	+
chr1	54878381	54879139	TCONS_00000524	−
chr13	19479579	19500896	TCONS_00021523+TCONS_00021478+TCONS_00021705 +TCONS_00022134+TCONS_00021524	+
chr5	90608491	90621000	TCONS_00010038	+
chr10	43250484	43265051	TCONS_l2_00003008	+
chr1	243219616	243265046	LOC731275	−
chr22	21655279	21679433	TCONS_l2_00018337+TCONS_l2_00018338+TCONS_ l2_00018339+TCONS_l2_00018340+TCONS_ l2_00018341+TCONS_l2_00017909+TCONS_ l2_00018342+TCONS_l2_00018343+TCONS_l2_00018344	−
chrX	144314449	144315139	TCONS_00017098	−
chr10	79492934	79494076	TCONS_l2_00003118	+
chr11	60803037	60807639	TCONS_00019908+TCONS_00019310	+
chr10	96901682	96928182	TCONS_00018027+TCONS_00019001	−
chr7	44824649	44826939	TCONS_00013162	−
chr19	781007	789792	TCONS_00027152+TCONS_00027153	−
chr2	208041221	208124809	TCONS_00004563+TCONS_00004564+TCONS_00005325 +TCONS_00005327+TCONS_00005329+TCONS_0000456 5+TCONS_00003487+TCONS_00002775+TCONS_000034 88+TCONS_00003489	−
chr4	190598741	190600367	TCONS_00008392	+
chr10	112629501	112631352	TCONS_00017869	+
chr5	92264	139978	TCONS_l2_00022102+TCONS_l2_00023297	+

chr4	153071426	153073566	TCONS_00008906	+
chr1	40289924	40303684	TCONS_00000911+TCONS_00001992	+
chr21	45576307	45580684	TCONS_00029171+TCONS_00029311+TCONS_00029312 +TCONS_00028953+TCONS_00028954	−
chr13	113804993	113807530	TCONS_00022128	−
chr9	109377359	109378263	TCONS_00015864+TCONS_00016455+TCONS_00016852	−
chr12	103941572	103972694	TCONS_00020194+TCONS_00020546+TCONS_00020547 +TCONS_00021202	+
chr21	46222008	46224632	TCONS_00029055+TCONS_00028857	+
chr7	55640864	55656029	TCONS_00013014	+
chr4	25554574	25556581	TCONS_00008451	−
chr4	31351906	31353347	TCONS_00007503	+
chr14	81908345	81916490	TCONS_00022814	−
chr18	39766366	39805980	TCONS_00026503	−
chr5	14889632	14892615	TCONS_00009877	+
chr11	112236380	112248544	TCONS_00019475+TCONS_00019476	+
chr5	76287641	76292870	TCONS_00009427	+
chr2	193718809	193745990	TCONS_00004539	−
chr11	22004519	22141438	TCONS_00019257	+
chr19	23582038	23598873	TCONS_l2_00013364+TCONS_l2_00013365+TCONS_ l2_00012963	−
chr1	157152695	157157512	TCONS_l2_00000650+TCONS_l2_00002223	+
chr14	102097395	102100579	TCONS_00022639	+
chr3	57957604	57970238	TCONS_00006032	+
chr15	78996146	79029199	TCONS_l2_00008761+TCONS_l2_00008762+TCONS_ l2_00008763+TCONS_l2_00008764	+
chr5	55807130	55828960	TCONS_00010342+TCONS_00010343	−
chr2	43268332	43270921	TCONS_00003265	−
chr1	102569886	102571564	TCONS_00001081	+
chr2	83083927	83084893	LOC1720	+
chr19	60951	70966	WASH5P	−
chr3	25900023	25915186	LINC00692	−
chr15	85070427	85114026	UBE2Q2P1	−
chr19	22777787	22778883	TCONS_00027230	−
chr17	29939643	29957144	TCONS_00025346	+
chr2	111132686	111142113	LOC151009	−
chr2	172345084	172347291	TCONS_00003937	+
chr5	179868559	179870238	TCONS_00009563	+
chr4	3940240	3951474	TCONS_l2_00020368	+
chr17	74826756	74831140	TCONS_00025499	+
chr3	127208396	127209630	TCONS_00005583	+
chr17	48838395	48844876	LINC00483	−
chr11	69902338	69911477	TCONS_00019173	−
chr1	684693	689194	TCONS_00002275	−
chr9	94763171	94766591	TCONS_00016407	−
chr4	185882457	185883921	TCONS_00008952	+
chr5	96392026	96392565	TCONS_l2_00023029	−
chr20	52873712	52904357	TCONS_00028444	−

chr10	110307733	110321573	TCONS_00018041	−
chr6	92100031	92102718	TCONS_00011527	−
chr22	16953632	16954957	TCONS_l2_00017858	−
chr2	175190755	175195370	LOC285084	+
chr2	118982820	118983456	TCONS_00003829	+
chr5	115638051	115639831	TCONS_00009755	−
chr7	135862010	135864666	TCONS_00013607	+
chrY	25082602	26753172	TTTY4B	+
chr9	16035383	16061855	TCONS_00015925+TCONS_00016539	+
chr10	115282517	115291897	TCONS_00018340	+
chr4	171264239	171293462	TCONS_00007680	+
chr2	132227281	132230483	TCONS_00004445+TCONS_00004446	−
chr13	19174124	19187652	TCONS_l2_00007348	+
chr3	124780922	124782903	TCONS_00006193	+
chr2	113696552	113698598	TCONS_00003822	+
chr16	86115627	86123132	TCONS_00024306+TCONS_00025078	−
chr14	104689744	104709704	TCONS_00022644	+
chr5	56066626	56068123	TCONS_00009404	+
chr4	99579918	99599748	TCONS_00008170+TCONS_00008171+TCONS_00008172 +TCONS_00008845	+
chr10	26953135	26965088	TCONS_l2_00004124	−
chr15	20353045	20359916	TCONS_l2_00008392	+
chr2	205334799	205338560	TCONS_00003486	−
chr2	227044760	227050087	TCONS_00003502	−
chr4	34834396	34848387	TCONS_00008462	−
chr9	137419028	137437090	TCONS_00015892	−
chr6	126498339	126523385	TCONS_00011368	+
chr15	92006569	92038078	TCONS_00023240	+
chr10	29520260	29524860	TCONS_00018150	+
chr22	17517460	17539682	CECR7	+
chr1	209320564	209328446	TCONS_l2_00001739+TCONS_l2_00002758	−
chr6	31349688	31350115	TCONS_l2_00024711	−
chr9	137444584	137445743	TCONS_00015770	+
chr17	18414576	18424566	USP32P2	−
chr6	48180919	48181910	TCONS_l2_00024781	−
chr9	46685763	46688137	TCONS_l2_00029951+TCONS_l2_00029952+TCONS_ l2_00029953+TCONS_l2_00029954	−
chrX	43036243	43085847	TCONS_00016931	+
chr1	225634945	225653045	TCONS_00000745+TCONS_00000746+TCONS_00001845	−
chr12	128504490	128506621	TCONS_00020990	−
chr7	20875050	21061771	TCONS_l2_00027069	+
chr10	88161760	88162970	TCONS_00018561+TCONS_00018986	−
chr15	30240229	30241270	TCONS_00023896	+
chr4	161730282	161740018	TCONS_l2_00020853	+
chr2	88584000	88584638	TCONS_00003760+TCONS_00003761	+
chr16	63764145	63765854	TCONS_00024432+TCONS_00024433	+
chr9	138466771	138478958	LOC100130954	+
chr1	30184549	30185414	TCONS_00000877	+

chr7	26591458	26594895	TCONS_l2_00027080+TCONS_l2_00027081+TCONS_l2_00025736	+
chr9	130873450	130881013	LOC100289019	−
chr1	184090200	184091540	TCONS_00001218	+
chr5	17684693	17955966	TCONS_l2_00023346+TCONS_l2_00022217+TCONS_l2_00023347+TCONS_l2_00022218+TCONS_l2_00022219+TCONS_l2_00023348+TCONS_l2_00022220	+
chr6	120200454	120201751	TCONS_00011940	+
chrX	134530354	134531689	TCONS_00017014	+
chr12	116985551	116989617	TCONS_00020583+TCONS_00020584	+
chr1	44404330	44406129	TCONS_00002373	−
chr7	27255062	27261329	TCONS_00013397	+
chr9	90790427	90791995	TCONS_00016054	+
chr4	14473680	14889793	TCONS_l2_00021830+TCONS_l2_00021831+TCONS_l2_00021832	−
chr10	19620314	19641181	TCONS_l2_00002906	+
chr22	47596306	47604489	TCONS_00029617+TCONS_00029837	+
chrX	73040486	73072588	XIST	−
chr8	40788310	40810979	TCONS_00014986	−
chr16	11290476	11318498	TCONS_00024807	+
chr9	36307039	36315276	TCONS_00015666	+
chr5	172923687	172951116	TCONS_00010179	+
chr10	35262922	35264491	TCONS_l2_00002966	+
chr21	30742340	30743547	TCONS_00028912	−
chr6	57125324	57128144	TCONS_00011222+TCONS_00011511	−
chr18	71016037	71320960	TCONS_00026734+TCONS_00026558+TCONS_00026559+TCONS_00026735	−
chr5	126567725	126618000	TCONS_00010469	−
chr12	47802261	47814022	TCONS_00020399+TCONS_00021113+TCONS_00021114	+
chr20	46611916	46613124	TCONS_00028558+TCONS_00027940	+
chrX	33744625	33960397	TCONS_00016974+TCONS_00016975	+
chr11	114518757	114522234	TCONS_00019766	−
chr1	23940283	23945957	TCONS_00000483	−
chr8	1741068	1744122	TCONS_00014909	−
chr7	47661537	47669491	TCONS_00013008	+
chr9	132173378	132174364	TCONS_00016184	+
chr7	62910803	62917227	TCONS_l2_00025913	+
chr15	31685046	31697027	TCONS_00023259+TCONS_00023185+TCONS_00023218+TCONS_00023342	+
chr1	63154153	63176365	TCONS_00000214	+
chr10	5307996	5313199	TCONS_00017911	−
chr10	3308195	3309207	TCONS_00017767	+
chr10	60064313	60065381	TCONS_00017829	+
chrY	9544433	9552871	TTTY7	−
chr15	23873169	23874538	TCONS_00023300	+
chr6	169072847	169074629	TCONS_00012021	+
chr3	75379075	75388094	TCONS_l2_00019357	−
chr1	4631587	4653707	TCONS_00000826+TCONS_00000827+TCONS_00000145	+
chr3	63752130	63768775	TCONS_l2_00019333+TCONS_l2_00020127	−

chr7	119856572	119867454	TCONS_00014415+TCONS_00013939	−
chr19	24101883	24159636	TCONS_l2_00013366+TCONS_l2_00013367+TCONS_l2_00012968+TCONS_l2_00012970+TCONS_l2_00012969	−
chr21	46707967	46717269	LOC642852	+
chr5	175399604	175414364	TCONS_00010185+TCONS_00009548+TCONS_00009549+TCONS_00009550+TCONS_00010842	+
chr19	45456828	45457419	TCONS_00027378	−
chr17	27339799	27343731	TCONS_00025343+TCONS_00025344+TCONS_00025163+TCONS_00025164	+
chr19	10152032	10184813	C3P1	+
chr5	80243530	80256189	TCONS_00010388+TCONS_00010389+TCONS_00010390+TCONS_00010391+TCONS_00009714	−
chr11	117005796	117009567	TCONS_l2_00004785+TCONS_l2_00005339+TCONS_l2_00004786	+
chr1	56620356	56621430	TCONS_00001500	−
chr11	119858517	119865411	TCONS_00019779+TCONS_00020140+TCONS_00019780	−
chr11	45760623	45762678	TCONS_00019632	−
chr1	112549543	112550756	TCONS_00001103	+
chr22	25723209	25742265	TCONS_l2_00017656+TCONS_l2_00017657+TCONS_l2_00017658	+
chr12	91155689	91200553	TCONS_00020867	−
chr2	238121308	238121926	TCONS_00004060	+
chr2	45181803	45195897	TCONS_00002884	+
chr10	33362428	33405711	TCONS_00018476+TCONS_00017947	−
chr13	66503637	66510480	TCONS_00021813	+
chr19	9375882	9381015	TCONS_l2_00012838+TCONS_l2_00012839	−
chr20	1166983	1170031	TCONS_00028475	+
chr11	77282132	77290777	TCONS_00019398+TCONS_00019399	+
chr1	14531974	14532868	TCONS_00000842	+
chr1	191685359	191686611	TCONS_00001761	−
chr21	24849960	24863043	TCONS_00028807	+
chr7	62693493	62702194	TCONS_l2_00027370+TCONS_l2_00026607	−
chr9	12738519	12770158	TCONS_00015920	+
chr4	171147756	171204866	TCONS_00008931+TCONS_00008932+TCONS_00008319+TCONS_00007679+TCONS_00008320	+
chr4	183958818	183961272	FAM92A1P2	+
chr14	40880506	40882879	TCONS_00022723	−
chr7	24215206	24222766	TCONS_00013728	−
chr1	24526730	24538180	LOC284632	+
chr16	32926412	32926831	TCONS_l2_00009693	+
chrX	46182883	46187090	TCONS_00017498+TCONS_00017315+TCONS_00017501+TCONS_00017500+TCONS_00017499+TCONS_00017503+TCONS_00016948	−
chr2	241871930	241904967	TCONS_l2_00014323+TCONS_l2_00014324+TCONS_l2_00014325+TCONS_l2_00014326	+
chr6	72038645	72039509	TCONS_00011323	+
chr5	171222600	171222948	TCONS_00010555	−
chr9	37079893	37090398	LOC100506710	+
chr4	185395862	185398943	TCONS_00008951	+

chr9	138506142	138511218	TCONS_l2_00029880+TCONS_l2_00029116	+
chr20	22585356	22599674	TCONS_00028126	+
chrX	113214010	113220920	TCONS_00017454+TCONS_00017230	+
chr1	179895425	179896015	TCONS_00002525	−
chr8	9182561	9192590	LOC157273	+
chr20	62032242	62033399	TCONS_00028471	−
chr16	20602035	20607617	TCONS_l2_00010038	−
chr20	10298534	10299368	TCONS_00028615	−
chr4	25610333	25621087	TCONS_00007494	+
chr21	22114913	22175426	LINC00320	−
chrX	23093748	23096498	TCONS_l2_00030163+TCONS_l2_00030164	+
chr4	188291802	188293075	TCONS_00007694	+
chr3	151488244	151502682	LOC201651	+
chr3	145679542	145681410	TCONS_00005606	+
chr3	102636251	102871281	TCONS_00006138+TCONS_00006923	+
chr7	141998745	141999096	TCONS_l2_00026248	+
chr14	39901834	39962715	TCONS_00022972+TCONS_00022482+TCONS_00022974+TCONS_00022973+TCONS_00022975+TCONS_00022976+TCONS_00022483+TCONS_00022484+TCONS_00022485	+
chr17	20744007	20747791	TCONS_l2_00010619+TCONS_l2_00010620+TCONS_l2_00011535	+
chr15	34967556	35012708	TCONS_00023623	−
chr8	98200364	98204150	TCONS_l2_00027902	+
chr12	48849393	48853296	TCONS_00020409+TCONS_00020410	+
chr4	79892902	80229953	LOC100505875	+
chr9	115505051	115512795	TCONS_00016465+TCONS_00015870	−
chr16	18487032	18487427	TCONS_l2_00010025	−
chr16	49196605	49203932	TCONS_00024386+TCONS_00024839+TCONS_00024387	+
chr3	20383960	20392420	TCONS_00005498+TCONS_00005964	+
chrX	150084286	150089411	TCONS_00017024+TCONS_00017025	+
chr2	146672658	146688714	TCONS_00003026	+
chr13	43416550	43417023	TCONS_00021640	−
chr2	54950821	54951863	TCONS_00003684	+
chr8	132065846	132142145	TCONS_00014859+TCONS_00014860	+
chr15	24992110	24997809	TCONS_00023877	+
chr22	18848964	18851914	TCONS_00029753+TCONS_00029754	+
chr9	45441937	45563561	TCONS_l2_00029741+TCONS_00015680+TCONS_l2_00028751	+
chr2	146051845	146089415	TCONS_00003414	−
chr13	36731086	36735999	TCONS_00021967	−
chr5	54895899	54899362	TCONS_00010337	−
chr1	150507528	150508415	TCONS_00001678	−
chr20	23499783	23522655	CST13P	+
chr3	26660821	26664181	TCONS_00005751	−
chr17	71272639	71274712	TCONS_00025198	+
chr20	37075297	37079564	SNHG11	+
chr2	111003215	111024135	LOC100507334	+

chr17	20322663	20327916	TCONS_l2_00010615	+
chr20	44216630	44218039	TCONS_00028408	−
chr3	186194516	186196159	TCONS_00005684	+
chr12	124558542	124559839	TCONS_00020607	+
chr4	70999321	71012421	TCONS_00007425	−
chr18	46466	49388	TCONS_l2_00011910	−
chr17	50939481	50976948	TCONS_00025191	+
chr1	142553293	142559170	TCONS_00000291	+
chr7	53626907	53635942	TCONS_00013173	−
chr1	228155359	228158853	TCONS_l2_00002788	−
chr2	132905164	133015542	ANKRD30BL	−
chr6	169557257	169563089	TCONS_00012828+TCONS_00012830+TCONS_00011622 +TCONS_00011623+TCONS_00012368	−
chrY	9555262	9558905	TTTY21	−
chr1	168773292	168838565	TCONS_00001191	+
chr20	48788628	48793180	TCONS_00028211+TCONS_00028212+TCONS_00028213+ TCONS_00027947+TCONS_00027948+TCONS_00028563	+
chr1	60708563	60724248	TCONS_00000966	+
chr2	143628158	143628636	TCONS_00004464	−
chr10	5605288	5606443	TCONS_00018420	−
chr17	19398722	19410608	TCONS_l2_00010598+TCONS_l2_00010599+TCONS_ l2_00010600	+
chr1	79786813	79787278	TCONS_00001534	−
chr16	87807655	87808766	TCONS_00024504	+
chr9	140119094	140119403	TCONS_00016732	+
chr3	11912786	11926479	TCONS_l2_00018438	+
chr5	102749590	102766255	TCONS_l2_00023040	−
chr7	62669577	62672161	TCONS_l2_00025907	+
chr22	41454665	41470331	TCONS_l2_00017775	+
chr4	187137130	187137707	TCONS_00008968	+
chr15	33528677	33539756	TMCO5B	−
chr11	3875548	3876739	TCONS_00019165	−
chr10	3266008	3269599	TCONS_00017766	+
chr6	14513058	14514697	TCONS_00011703	+
chr5	86093502	86096097	TCONS_00009440	+
chr9	106761762	106847977	TCONS_00016109+TCONS_00016108+TCONS_00016110	+
chr16	76803213	76806959	TCONS_00024472	+
chr7	17319458	17320930	TCONS_l2_00027275+TCONS_l2_00027277	−
chr11	39183024	39266897	TCONS_l2_00004485	+
chr13	38055604	38058906	TCONS_00021635	−
chr1	57289361	57292593	TCONS_00000080	−
chr3	182362002	182373668	TCONS_l2_00020002+TCONS_l2_00019027	+
chr12	124634145	124725562	TCONS_00020611+TCONS_00020612+TCONS_00021242	+
chr1	79490858	79493876	TCONS_00001528	−
chr20	58042934	58044890	TCONS_00028456	−
chr3	116271320	116287111	TCONS_00005574	+
chr4	187653484	187659015	TCONS_00008367	+
chr4	125070532	125173199	TCONS_00007819	−

chr6	51274335	51275340	TCONS_l2_00024783	−
chr11	67889047	67897983	TCONS_00019356+TCONS_00019939	+
chr4	32185971	32194332	TCONS_00008033	+
chr11	19329683	19332235	TCONS_00019251	+
chr20	61199863	61200695	TCONS_00028462	−
chr22	27672201	27682310	TCONS_00029401+TCONS_00029564	+
chr20	26167655	26189869	LOC284801	−
chr1	162530263	162531146	TCONS_00001707	−
chr4	185972914	186028403	TCONS_l2_00020933+TCONS_l2_00021786+TCONS_l2_00021787+TCONS_l2_00020934+TCONS_l2_00020936+TCONS_l2_00021788+TCONS_l2_00020937	+
chr16	33647308	33661699	TCONS_l2_00010131	−
chr13	98327265	98330217	TCONS_00021691	−
chr19	32516636	32539753	TCONS_00026985+TCONS_00026785+TCONS_00026829+TCONS_00026987+TCONS_00027515+TCONS_00027516+TCONS_00026988	+
chr2	138830230	138836624	TCONS_00004460	−
chrX	13973512	13982023	TCONS_00016969	+
chr22	45057492	45063704	TCONS_00029709	−
chr13	55260674	55264577	TCONS_00021797	+
chr2	32018892	32029049	TCONS_00003249	−
chr15	62022063	62023589	TCONS_00023436+TCONS_00023437	+
chr3	75606117	75608433	TCONS_00006100+TCONS_00006101	+
chr6	160320218	160323542	TCONS_00011405	+
chr15	63723374	63730499	TCONS_00023441	+
chr2	97930723	97935466	TCONS_00004339+TCONS_00004340+TCONS_00004341+TCONS_00004342	−
chr12	96506541	96545912	TCONS_00020532	+
chrX	6685657	6895201	TCONS_00017287+TCONS_00017288	−
chr13	105316721	105330726	TCONS_00022100	−
chr4	76744128	76746496	TCONS_00008111	+
chr11	65222665	65234212	TCONS_l2_00004561+TCONS_l2_00004562+TCONS_l2_00004563+TCONS_l2_00004564	+
chr4	174990969	175001205	TCONS_l2_00020896	+
chr9	68773916	68778906	TCONS_l2_00029366	−
chr19	21155029	21167734	TCONS_00026944	+
chr15	39804622	39812137	TCONS_00023639+TCONS_00024081+TCONS_00024082	−
chr12	45870534	45875130	TCONS_00020765	−
chr7	10716286	10747385	TCONS_00013336+TCONS_00012950	+
chr7	119781093	119783079	TCONS_l2_00026861	−
chr21	15142253	15144061	TCONS_00028791	+
chr3	16283254	16290513	TCONS_00007127	−
chr21	20502841	20506741	TCONS_00028802	+
chr9	35013512	35015067	TCONS_00015964	+
chr4	172042680	172142533	TCONS_l2_00021387	−
chr14	56042875	56046810	KTN1-AS1	−
chr16	82578752	82609064	TCONS_00024743+TCONS_00024744	−
chr21	18382324	18386536	TCONS_00029084	−
chr21	40687633	40695144	BRWD1-AS1	+

chr6	80773211	80780323	TCONS_l2_00024305	+
chrY	6258472	6279605	TTTY1	+
chr8	30593693	30594132	TCONS_00014964	−
chr2	217374176	217394310	TCONS_00005010+TCONS_00004017+TCONS_00003107 +TCONS_00004018	+
chr1	846815	850351	TCONS_00000032+TCONS_00000134	+
chr4	9569105	9648636	TCONS_l2_00021062+TCONS_l2_00021063+TCONS_ l2_00021064	−
chr17	65255054	65258409	TCONS_l2_00010931	+
chr2	235742959	235822028	TCONS_00003514+TCONS_00003515+TCONS_00002782	−
chr17	48292055	48292781	TCONS_00025185	+
chr5	177479413	177479818	TCONS_00010578	−
chr11	103436304	103509798	TCONS_00019740	−
chr4	119584229	119596884	TCONS_00008555+TCONS_00008556	−
chr2	693211	697855	TCONS_00003537	+
chr2	17424591	17443007	TCONS_00003588	+
chrX	109700008	109839249	TCONS_00017362	−
chr6	25992927	26002137	TCONS_00011742+TCONS_00011743+TCONS_00012437 +TCONS_00012438	+
chr2	79146572	79158506	TCONS_00004318	−
chr7	22871220	22874253	TCONS_00013378	+
chr1	116461997	116468528	TCONS_00000052	+
chr10	94178418	94179363	MARK2P9	+
chrX	48995951	49003812	TCONS_l2_00030488	−
chr11	74035160	74035785	TCONS_00019389	+
chr18	19495223	19496693	TCONS_00026293	+
chr8	145143470	145148644	TCONS_00015198	−
chr10	134309449	134350303	TCONS_l2_00003843+TCONS_l2_00004301+TCONS_ l2_00003844	−
chr8	93725190	93798288	FLJ46284	−
chrX	18373300	18376810	TCONS_00017146	+
chr4	28754425	28754905	TCONS_00007497	+
chr11	309549	311060	TCONS_00020033	−
chr17	47535223	47539111	TCONS_00025144	−
chr7	103642282	103672626	TCONS_00013910	−
chr15	37156644	37178734	LOC145845	−
chr3	153599315	153658625	TCONS_00005630	+
chr6	122448371	122452271	TCONS_00012265	−
chr1	4007209	4009040	TCONS_00000462+TCONS_00002282	−
chr8	91604969	91631400	TCONS_00015085+TCONS_00015086	−
chr16	85170756	85183049	LOC400548	+
chr6	19068774	19180711	TCONS_l2_00025343+TCONS_l2_00024637	−
chr1	31283225	31288400	TCONS_00000498	−
chr12	54151158	54155853	TCONS_00020434	+
chr5	110306650	110344598	TCONS_l2_00023859	−
chr8	8947524	8957733	TCONS_00014597	+
chrX	49966653	49969247	TCONS_00017322	−
chr10	3810107	3810943	TCONS_00017769	+

chr16	89883185	89894373	TCONS_00024918+TCONS_00024919	+
chr14	41423822	41445070	TCONS_00022487+TCONS_00022390+TCONS_00022346 +TCONS_00022488	+
chr9	86710486	86711662	TCONS_00016029	+
chr1	79346570	79351681	TCONS_00001527	−
chr8	21155710	21166960	TCONS_00014646+TCONS_00014484	+
chr19	29938475	29939292	TCONS_00026982	+
chr4	55306669	55312890	TCONS_00007759	−
chr6	30807303	30815936	TCONS_00011480+TCONS_00012141	−
chrY	58883380	58887164	TCONS_00017658	+
chr11	3429919	3432082	TCONS_l2_00004893	−
chr22	27959800	27969534	TCONS_00029463	−
chr7	36790292	36802686	TCONS_00012995	+
chr7	24234875	24295338	TCONS_00013729+TCONS_00013142	−
chr7	46930223	47118726	TCONS_l2_00027349+TCONS_l2_00027350+TCONS_ l2_00027351+TCONS_l2_00027352	−
chr16	51051483	51069696	TCONS_00024395+TCONS_00024396+TCONS_00024840	+
chr2	105469951	105471715	TCONS_l2_00013870+TCONS_l2_00013871+TCONS_ l2_00013872	+
chr7	65216092	65228662	CCT6P1	+
chr2	130970622	130970942	TCONS_l2_00014894	−
chr12	9546891	9560098	TCONS_l2_00006111+TCONS_l2_00006112+TCONS_ l2_00006113+TCONS_l2_00006114+TCONS_ l2_00006115+TCONS_l2_00006116+TCONS_ l2_00006117+TCONS_l2_00006118+TCONS_ l2_00006119+TCONS_l2_00006120+TCONS_ l2_00006121+TCONS_l2_00006122+TCONS_ l2_00006123+TCONS_l2_00006124+TCONS_ l2_00006125+TCONS_l2_00006126+TCONS_l2_00006127	−
chr9	94895116	94900911	LOC100128076	+
chr6	166471807	166473129	TCONS_00012359	−
chr13	64196102	64204813	TCONS_00022042	−
chr1	170567995	170616480	TCONS_00000332	+
chr11	60794935	60810810	TCONS_00019644	−
chr4	153128573	153147022	TCONS_00007882+TCONS_00007883	−
chr10	31893292	31895146	TCONS_00018154+TCONS_00017809	+
chr2	242458387	242459662	TCONS_l2_00015630	+
chr2	60566749	60567614	TCONS_00004264	−
chr3	184880689	184909743	EHHADH-AS1	+
chr11	33958152	33976322	TCONS_00019611	−
chr12	124572821	124631821	TCONS_00020608+TCONS_00020610+TCONS_00021240 +TCONS_00020609+TCONS_00021241	+
chr3	75691158	75695597	TCONS_00006544	−
chr3	195435002	195438746	TCONS_00005713+TCONS_00005714+TCONS_00005715 +TCONS_00006379+TCONS_00006380+TCONS_0000638 1+TCONS_00006382	+
chr15	23656903	23662589	TCONS_00023298	+
chr9	67281399	67288130	TCONS_l2_00028765	+
chr20	61726845	61733671	HAR1B	−
chr2	96465026	96468984	TCONS_l2_00013806	+

chr20	51288415	51315089	TCONS_00028223+TCONS_00027951+TCONS_00027952 +TCONS_00027953+TCONS_00028224	+
chr6	102575603	102580400	TCONS_00012234	−
chr10	43818459	43823417	TCONS_00018174+TCONS_00017818	+
chrX	119144675	119149501	TCONS_00017086	−
chr4	187207252	187422212	LOC285441	−
chr5	104079736	104099084	TCONS_00010757	+
chr21	9826394	9827720	TCONS_00029068+TCONS_00029069+TCONS_00029071 +TCONS_00029072	−
chr2	468568	470794	TCONS_00003529	+
chr4	9167676	9168601	TCONS_l2_00021058	−
chr13	38624954	38717369	LINC00571	−
chr1	156657489	156661789	TCONS_00000646	−
chr3	71834486	71835757	TCONS_00006534	−
chr19	20929683	20934053	TCONS_l2_00012920+TCONS_l2_00012921+TCONS_ l2_00012922	−
chr3	165197868	165244265	TCONS_00005474	−
chr1	143498344	143517299	TCONS_00000295	+
chr3	97910049	97915894	TCONS_00006131+TCONS_00006132	+
chr12	16573561	16600358	TCONS_00021084+TCONS_00020346+TCONS_00020347	+
chr2	181436439	181557181	TCONS_00003459	−
chr16	19934892	19940581	TCONS_00024342+TCONS_00024814	+
chr17	14112887	14121239	TCONS_00025311+TCONS_00025844+TCONS_00025312 +TCONS_00025156	+
chr2	92150358	92151786	TCONS_l2_00013794	+
chr7	64601603	64694599	TCONS_l2_00027131+TCONS_l2_00025960+TCONS_ l2_00025961+TCONS_l2_00025962+TCONS_ l2_00025963+TCONS_l2_00025964+TCONS_ l2_00025965+TCONS_l2_00025966	+
chrX	36197474	36223194	TCONS_00017151+TCONS_00017152	+
chr7	158766826	158775604	TCONS_00013659+TCONS_00013660	+
chr16	72910	75121	TCONS_00024242	+
chr5	30102585	30106518	TCONS_00009916	+
chr14	105506822	105509821	TCONS_00022897	−
chr20	34674174	34676114	TCONS_00028041	−
chr10	60759263	60825619	TCONS_00018733+TCONS_00018734+TCONS_00018736 +TCONS_00017830+TCONS_00018737+TCONS_0001873 8+TCONS_00018208+TCONS_00018739	+
chr16	34477761	34479038	TCONS_00024376	+
chr4	109592411	109602985	TCONS_00008193	+
chr6	71897277	71943366	TCONS_00012208	−
chrY	24442945	24445023	TTTY5	−
chr6	159550411	159552322	TCONS_00012006	+
chr20	21074190	21086975	TCONS_00028014+TCONS_00028015	−
chr8	143073635	143078899	TCONS_00014881	+
chr2	132160474	132165801	LOC389043	+
chr2	112641882	112645656	TCONS_l2_00013902	+
chr6	6659635	6733623	TCONS_00011665+TCONS_00011666+TCO NS_l2_00025080+TCONS_l2_00024007+TCONS_ l2_00024008+TCONS_l2_00025081	+

chr18	5748818	5795900	LOC645355	+
chr10	121861759	121863424	TCONS_00018618	−
chr17	6921468	6923198	TCONS_00026027	−
chr4	145122623	145134247	TCONS_00009133+TCONS_00008603	−
chr6	37484564	37509148	TCONS_00011802+TCONS_00012487+TCONS_00012488 +TCONS_00011803+TCONS_00011804	+
chr5	171150035	171164435	TCONS_00010554+TCONS_00011086+TCONS_00011087	−
chr6	26494960	26500809	TCONS_00011746	+
chr16	74411388	74411717	TCONS_00025055	−
chr4	5034987	5035431	TCONS_00007966	+
chr6	8284311	8307763	TCONS_00011678	+
chr16	1041085	1043057	TCONS_00024536	−
chr1	226723319	226730469	TCONS_l2_00002786+TCONS_l2_00001826	−
chr4	89206094	89262948	TCONS_00007396+TCONS_00008155+TCONS_00008154	+
chr14	98561563	98576723	TCONS_00022623	+
chr11	126888995	126891438	TCONS_00019527+TCONS_00020017	+
chr8	95649513	95651695	LOC100288748	−
chr20	10855981	10889920	TCONS_l2_00016848	−
chr5	142938784	142942113	TCONS_00009532	+
chr10	69993008	70002136	TCONS_00017991+TCONS_00017992	−
chr10	92754377	92766147	TCONS_00017857	+
chr2	74375108	74379516	BOLA3-AS1	+
chr1	212679674	212688157	TCONS_00001807	−
chr12	97415531	97417027	TCONS_00020888	−
chr10	124480829	124493914	TCONS_l2_00004048	+
chr4	166605791	166683930	TCONS_00007677	+
chr12	4320970	4322471	TCONS_00020279	+
chr5	89705872	89712311	TCONS_00010036+TCONS_00010037	+
chr1	50671756	50688799	TCONS_00000520	−
chr4	26765709	26766256	TCONS_00008015	+
chr17	20384375	20387265	TCONS_l2_00011134+TCONS_l2_00011135	−
chr19	58412462	58413066	TCONS_00027818	−
chr4	129489127	129491599	TCONS_00007434	−
chrX	38342053	38344145	TCONS_l2_00030467	−
chr6	168630412	168641395	TCONS_00011417+TCONS_00012627+TCONS_00012628	+
chr9	18360595	18362218	TCONS_00015796	−
chr2	63902748	64067902	TCONS_l2_00014608+TCONS_l2_00014609+TCONS_l2 _00014610+TCONS_00005162+TCONS_00005163+TCO NS_l2_00014611	−
chr6	44698855	44705091	TCONS_00011169	+
chr20	12286261	12297133	TCONS_00028006	−
chr19	57808920	57811732	TCONS_00027635	+
chr1	81001440	81112834	TCONS_00000240+TCONS_00000239+TCONS_00002080 +TCONS_00000241+TCONS_00001004	+
chr10	118904091	118928566	TCONS_00018610+TCONS_00018611+TCONS_00019033+ TCONS_00018612+TCONS_00018613+TCONS_00018052	−
chr5	119015892	119016527	TCONS_l2_00022494	+
chr8	102145614	102151881	TCONS_00014810	+

chr6	170401013	170442052	TCONS_00012376	−
chr1	700245	714068	LOC100288069	−
chr4	118755556	118791103	TCONS_00007602+TCONS_00008206	+
chr5	71865288	71867520	TCONS_00009995	+
chr11	134605490	134633704	TCONS_l2_00005352	+
chr21	34331196	34332874	TCONS_00028833	+
chr5	133735186	133737612	TCONS_00009512	+
chr9	66922967	66933827	TCONS_l2_00029754	+
chr6	158137090	158210465	TCONS_00012620	+
chr12	72101600	72102204	TCONS_00020839	−
chr7	77619701	77624488	TCONS_00013024+TCONS_00012883+TCONS_00013025	+
chr12	77667458	77710985	TCONS_00020850+TCONS_00021370	−
chr16	14914651	14915183	TCONS_l2_00010020	−
chr20	1448863	1454463	TCONS_l2_00016424+TCONS_l2_00016425	−
chr10	128591477	128593410	TCONS_00018637	−
chr6	75303895	75443995	TCONS_l2_00025422+TCONS_l2_00024825+TCONS_ l2_00025423	−
chr3	106086838	106146799	TCONS_00006572+TCONS_00006573+TCONS_00006575 +TCONS_00006574+TCONS_00006576	−
chr19	31398106	31400062	TCONS_00026783	+
chr2	89110844	89124720	TCONS_l2_00014679+TCONS_l2_00015858	−
chr12	48672796	48677977	TCONS_00020776	−
chr5	107114579	107117792	TCONS_l2_00022460	+
chr6	111598892	111619807	TCONS_00011918+TCONS_00011355+TCONS_00012569	+
chr13	38109077	38125673	LINC00547	+
chr6	33857867	33896060	TCONS_00011787+TCONS_00012478+TCONS_00011788 +TCONS_00012479+TCONS_00012480+TCONS_0001178 9+TCONS_00011168+TCONS_00011295	+
chr17	37916402	37920067	TCONS_00025171	+
chr11	63261875	63268083	TCONS_00019317+TCONS_00019914+TCONS_00019318	+
chr2	242671429	242673870	TCONS_00004641	−
chr2	65681085	65683653	TCONS_00005170	−
chr6	159586941	159588385	TCONS_00011403	+
chr2	166651361	166666520	TCONS_00003042	+
chr19	15716475	15718461	TCONS_00027203	−
chr21	44299768	44303945	TCONS_00029037+TCONS_00029038+TCONS_00029039 +TCONS_00029230	+
chr2	216582766	216584147	TCONS_00003105	+
chr16	86337504	86338079	TCONS_00024494	+
chr14	24161843	24168143	TCONS_00022429+TCONS_00022430+TCONS_00022431	+
chr10	100995869	100996402	TCONS_00018301	+
chr2	66650475	66660602	MEIS1-AS3	−
chr18	106602	108335	TCONS_00026389	−
chr20	23484350	23487749	TCONS_00028132+TCONS_00028133	+
chr6	128969666	129015458	TCONS_00011948+TCONS_00012597+TCONS_00011949	+
chr5	99989792	99993674	TCONS_00009735+TCONS_00009736	−
chr15	85054686	85055992	TCONS_l2_00008795	+

chr11	66176718	66186277	TCONS_l2_00004586+TCONS_l2_00004587+TCONS_l2_00004588+TCONS_l2_00004589+TCONS_l2_00004590+TCONS_l2_00004591+TCONS_l2_00004592	+
chr5	56869854	56913301	TCONS_00009405+TCONS_00009972+TCONS_00009406+TCONS_00010694	+
chr6	167647448	167655400	TCONS_00012361+TCONS_00011614+TCONS_00012826	−
chr18	56112363	56117785	TCONS_00026343	+
chr1	13162851	13164468	TCONS_l2_00002430	−
chr13	98191827	98202373	TCONS_00021877	+
chr4	19457059	19911315	TCONS_l2_00021543+TCONS_l2_00020442	+
chr1	18044182	18076473	TCONS_00000160+TCONS_00000852	+
chr4	42283847	42393285	TCONS_00007753+TCONS_00007754+TCONS_00007755	−
chrX	28061039	28069653	TCONS_00017149	+
chr8	8817861	8819988	TCONS_l2_00028091	−
chr9	86678358	86709492	TCONS_l2_00029784+TCONS_l2_00029785	+
chr7	31454259	31460900	TCONS_l2_00027313+TCONS_l2_00026501	−
chr12	74310012	74313300	TCONS_00020471	+
chr5	15112486	15117116	TCONS_00009608	−
chr20	21928285	21951421	TCONS_00027911	+
chr18	12228875	12234483	TCONS_00026427	−
chr21	32965036	32966020	TCONS_00028992	+
chr4	38749927	38754586	TCONS_00008049+TCONS_00008777	+
chr19	44326209	44331358	TCONS_00026815	−
chr21	40447036	40454676	TCONS_00029141+TCONS_00029142+TCONS_00029143	−
chr10	133821407	133824860	TCONS_00018392	+
chr2	292148	293374	TCONS_00005051	−
chr15	30699085	30702032	TCONS_l2_00008967+TCONS_l2_00008968+TCONS_l2_00008969	−
chr5	16180033	16181223	TCONS_00010903	−
chr8	12052268	12060127	TCONS_00015223+TCONS_00014620+TCONS_00015224	+
chr7	125557925	125573886	TCONS_00013074	+
chr19	56905045	56910544	ZNF582-AS1	+
chr6	3056124	3056995	TCONS_00011254	+
chr4	34859924	34862148	TCONS_00008037	+
chr9	40332917	40337603	TCONS_l2_00028740	+
chr4	74234052	74235567	TCONS_l2_00020575	+
chr16	86191731	86192470	TCONS_00024758	−
chr2	10675219	10682163	TCONS_l2_00014437	−
chr4	31999019	32157028	TCONS_l2_00021562+TCONS_l2_00020471+TCONS_l2_00020472	+
chr7	123977434	123992154	TCONS_00013250+TCONS_00012926	−
chr1	69898938	69901092	TCONS_00000221	+
chr22	25956903	25960789	TCONS_00029662+TCONS_00029882+TCONS_00029454+TCONS_00029455+TCONS_00029883+TCONS_00029456+TCONS_00029664+TCONS_00029663+TCONS_00029665	−
chr17	71940479	71945993	TCONS_00025761	−
chr15	30336704	30337753	TCONS_l2_00008516	+
chr1	244270667	244272313	TCONS_00000428	+

chr22	17134599	17156430	ANKRD62P1-PARP4P3	−
chr11	131123317	131170666	TCONS_00019161	+
chr15	95222488	95222914	TCONS_00023527	+
chrX	154644932	154649138	TCONS_00017391	−
chr6	143703394	143704591	TCONS_00011978	+
chr3	125505729	125508117	TCONS_l2_00019482	−
chr18	12031121	12032132	TCONS_00026678	−
chr9	106124234	106192174	TCONS_00015858	−
chr2	3523465	3527853	TCONS_l2_00013434	+
chr4	138676241	138678728	TCONS_00008261	+
chrX	45992911	45993894	TCONS_00017159	+
chr20	49752748	49759296	TCONS_00028218	+
chr11	3875842	3876629	TCONS_00019138	+
chr6	1080164	1105181	TCONS_00011147	+
chr5	37939467	37948057	TCONS_00009641	−
chr7	17414406	17506843	TCONS_l2_00025695+TCONS_l2_00027059+TCONS_l2_00025696	+
chr12	119651506	119658735	TCONS_00020592	+
chr1	113392650	113420493	TCONS_00000274+TCONS_00000275	+
chr5	174045167	174052778	TCONS_00009544	+
chr1	180121717	180123589	TCONS_00001737	−
chr3	74048956	74054760	TCONS_00005544	+
chr6	21522707	21524003	TCONS_00011465	−
chrX	30648436	30649080	TCONS_l2_00030171	+
chr1	238025475	238091619	LOC100130331	+
chr2	95534430	95613087	LOC442028	−
chr12	19705712	19706887	TCONS_00020350	+
chrX	72744111	72782921	MAP2K4P1	−
chr2	31990952	31997991	TCONS_00004197	−
chr14	95988349	95992377	TCONS_00022845	−
chr2	8026901	8039813	TCONS_l2_00015694+TCONS_l2_00014410+TCONS_l2_00015695+TCONS_l2_00014411	−
chr1	23878413	23881254	TCONS_00001418	−
chr4	7132607	7133131	TCONS_00008418	−
chr9	120410884	120419305	TCONS_00015741	+
chr17	53657960	53667280	TCONS_00025698	−
chr22	19847802	19854904	TCONS_00029514	+
chr2	38635914	38636326	TCONS_00004755	+
chr9	70922309	70923361	TCONS_00015687	+
chr11	130086480	130087479	TCONS_00019804	−
chr12	103346732	103347726	TCONS_00020542	+
chrY	28732670	28733627	TCONS_l2_00030955	−
chr9	45944184	45949407	TCONS_l2_00028752	+
chr18	33023961	33025707	TCONS_00026486	−
chr2	43401092	43411668	TCONS_00004231	−
chr2	174062441	174146764	MLK7-AS1	−
chr4	163378689	163379279	TCONS_00008638	−
chr5	7326177	7326722	TCONS_00009342	+

chr11	15933277	15952476	TCONS_00019858+TCONS_00019244	+
chr9	21696914	21699564	TCONS_l2_00028644	+
chr9	105902810	106087312	TCONS_l2_00030050+TCONS_l2_00029537+TCONS_ l2_00030051+TCONS_l2_00029538+TCONS_ l2_00029539+TCONS_l2_00029540	−
chrY	6274285	6296485	TTTY2B	−
chr4	120988666	121337921	TCONS_l2_00021689+TCONS_l2_00020731+TCONS_ l2_00020732	+
chr10	18942634	18948218	TCONS_l2_00003421+TCONS_l2_00004119+TCONS_ l2_00003422+TCONS_l2_00003423	−
chr14	30791434	30793829	TCONS_00022457	+
chr10	26190204	26194338	TCONS_00018142	+
chr1	212840987	212848586	TCONS_00001270+TCONS_00001269+TCONS_00002229	+
chr12	115542151	115542887	TCONS_00020577	+
chr11	106120746	106134796	TCONS_00019108+TCONS_00019458	+
chr1	22582820	22584588	TCONS_00000863+TCONS_00001956	+
chr16	68646657	68648289	TCONS_00024445	+
chr8	74791943	74816962	TCONS_00014761+TCONS_00014759+TCONS_00015305 +TCONS_00015306+TCONS_00015307	+
chr6	92921652	92955912	TCONS_00011888	+
chr7	151574127	151576308	PRKAG2-AS1	+
chr2	101218847	101225992	TCONS_l2_00013850+TCONS_l2_00015413+TCONS_ l2_00015414	+
chr8	125947429	125951274	TCONS_00015162	−
chr8	100012216	100014726	TCONS_00014805	+
chr14	103783646	103785383	TCONS_00022641	+
chr18	70821293	70931733	LOC400655	−
chr1	843465	845308	TCONS_00001368	−
chr2	216476286	216708259	LINC00607	−
chr3	47205860	47285606	KIF9-AS1	+
chr7	3122886	3126055	TCONS_00013118	−
chr14	56980668	56982196	TCONS_00022755+TCONS_00023112	−
chr6	26987145	26988085	LOC100270746	−
chr8	58256346	58277413	TCONS_l2_00028495+TCONS_l2_00027790+TCONS_ l2_00027791+TCONS_l2_00027792+TCONS_l2_00027793	+
chr19	12561749	12571519	TCONS_00026825	+
chr13	112928667	112929999	TCONS_00021915	+
chrX	73012040	73049066	TSIX	+
chr6	40846474	40966970	TCONS_00012166+TCONS_00012167+TCONS_00012713	−
chr14	27322321	27323813	TCONS_00022691	−
chr3	151835962	151869513	TCONS_00005849	−
chr17	80311630	80315022	TCONS_00025784	−
chrX	75122940	75124693	TCONS_00016997	+
chr20	50448336	50479451	TCONS_00028064	−
chr4	38533853	38548334	TCONS_00008047	+
chr1	20686294	20755287	LOC339505	−
chr3	184264502	184274706	TCONS_00005903	−
chr3	39280589	39300798	TCONS_00005993+TCONS_00006817	+
chr3	42716784	42724788	TCONS_00006498	−

chr9	94186604	94189429	TCONS_00016069+TCONS_00015712	+
chr14	19894369	19904572	LINC00516	+
chr19	22426867	22439237	TCONS_00027228+TCONS_00027703+TCONS_00027704+TCONS_00027705	−
chr15	99940270	99946149	TCONS_00023548	+
chr1	2482415	2486841	TCONS_00000807+TCONS_00001917	+
chr12	50302666	50305646	LOC283332	−
chr12	58475010	58484682	TCONS_00020814	−
chr12	108817310	108826418	TCONS_00020559	+
chrX	65014186	65015782	TCONS_l2_00030240+TCONS_l2_00030241	+
chr2	16497592	16500834	TCONS_00003216	−
chr6	142847592	142959026	LOC153910	−
chr6_cox_hap2	4288783	4292377	TCONS_00029951	+
chr1	31168512	31181236	TCONS_00002330+TCONS_00001446	−
chr8	50409334	50424778	TCONS_00015001	−
chr14	36683885	36685567	TCONS_00022468	+
chr9	108823635	108827274	TCONS_00015862	−
chr2	237957242	237964810	TCONS_l2_00016116	−
chr15	99557907	99561165	TCONS_00023545	+
chr22	17741490	17742178	TCONS_00029500	+
chr10	19579152	19580409	TCONS_00017937	−
chr4	27904190	27995255	TCONS_00008022+TCONS_00008023	+
chr6	139790132	139795733	LOC645434	−
chr12	20162821	20164493	TCONS_00020355	+
chr17	33640518	33652444	TCONS_00025355+TCONS_00025356+TCONS_00025358+TCONS_00025359+TCONS_00025361+TCONS_00025360+TCONS_00025896+TCONS_00025897+TCONS_00025362	+
chr4	139585879	139586723	TCONS_00007855	−
chr2	157644303	157722241	TCONS_00003913	+
chr10	99588235	99609535	TCONS_00018031	−
chr5	13986055	13987341	TCONS_00009876	+
chr21	16049036	16126186	TCONS_00029078+TCONS_00029256+TCONS_00028866+TCONS_00028867	−
chr1	246679341	246687589	LOC255654	−
chr8	29238673	29248125	TCONS_00014957	−
chr14	38073403	38075919	TCONS_00022477	+
chr2	106544669	106545197	TCONS_00004368	−
chr22	43796337	43805731	TCONS_l2_00018274+TCONS_l2_00017796	+
chr2	194746280	194755854	TCONS_00003473	−
chr13	62798230	62824080	TCONS_00021559+TCONS_00021808	+
chr3	162895031	163021089	CT64	−
chr1	142569266	142588234	TCONS_00000628	−
chr6	27634762	27635927	TCONS_00012129	−
chr12	76531795	76532210	TCONS_l2_00005758	+
chr13	112836837	112849301	TCONS_00022120+TCONS_00022316+TCONS_00022121	−
chr22	21022101	21025525	TCONS_l2_00017903	−
chr2	217455149	217471661	TCONS_00003496	−

chr4	38422283	38524801	TCONS_00007745	−
chr13	107270158	107284006	LINC00551	+
chr9	69450477	69481675	TCONS_l2_00028814+TCONS_l2_00028815+TCONS_l2_00029766	+
chr10	85671410	85672678	TCONS_00018011	−
chr12	56904734	56906923	TCONS_l2_00005705	+
chr22	20325590	20350461	LOC729444	+
chr7	76264152	76264572	TCONS_00014163	+
chr7	123454193	123459484	HYALP1	+
chr7	47801074	47806370	LINC00525	+
chr10	27269561	27271723	TCONS_00018897	−
chr1	27555597	27560974	TCONS_00000491+TCONS_00002324	−
chr16	66585262	66586381	TCONS_00025037	−
chr15	39676042	39684838	TCONS_00023358+TCONS_00023916+TCONS_00023917	+
chr17	6070242	6093823	TCONS_00025540	−
chr10	33867859	33888968	TCONS_l2_00004133+TCONS_l2_00003472	−
chr14	30938438	30971066	TCONS_00022698+TCONS_00022699	−
chrY	9650924	9655122	TCONS_00017612	+
chr13	110774616	110774945	TCONS_00021901	+
chr7	149903393	149918381	TCONS_00014003+TCONS_00014004	−
chr18	53497615	53506331	TCONS_00026336	+
chr16	54923451	54927395	TCONS_00024661	−
chr12	49672836	49676768	TCONS_00020244	−
chr22	42665759	42670868	LOC388906	+
chr2	132440113	132457434	TCONS_l2_00013998+TCONS_l2_00015489	+
chr17	10178300	10182496	TCONS_00025558	−
chr14	90921574	90925249	LINC00642	+
chr6	44492996	44494491	TCONS_00011306	+
chr10	114648492	114704954	TCONS_00017870+TCONS_00018338+TCONS_00018339	+
chr3	172871527	173054755	TCONS_00005660	+
chr12	29565419	29573303	TCONS_00020751	−
chr1	158101834	158110430	LOC646268	−
chr1	90224688	90229610	TCONS_00001575+TCONS_00001576	−
chr4	8321887	8325694	TCONS_00007975+TCONS_00007468+TCONS_00007469+TCONS_00008748	+
chr15	62682796	62897079	TCONS_00023956+TCONS_00023957+TCONS_00023958+TCONS_00023959	+
chr4	17173381	17187680	TCONS_00007417	−
chr17	49517014	49523163	TCONS_l2_00011578	+
chr1	238588741	238608261	TCONS_00001340	+
chr2	224904215	224907185	TCONS_00004043	+
chr9	104998737	105000407	TCONS_00016107	+
chr18	57053839	57056221	TCONS_00026722	−
chrX	122867024	122868144	TCONS_l2_00030743	+
chr18	137413	141924	TCONS_00026225	+
chr2	111203460	111230652	LIMS3	−
chr8	137809518	137909333	TCONS_00015182+TCONS_00015529	−
chr18	74334676	74350976	TCONS_00026573+TCONS_00026574+TCONS_00026575	−

chr2	164307189	164309847	TCONS_00003924	+
chr16	14416113	14420687	TCONS_00024334+TCONS_00024335+TCONS_00024336	+
chr1	38765781	38777050	TCONS_00001460	−
chr1	219150089	219164653	TCONS_00001821	−
chr3	129831393	129840161	TCONS_l2_00019880+TCONS_l2_00019881+TCONS_ l2_00018852+TCONS_l2_00018853+TCONS_l2_00018854	+
chr10	5196655	5227150	AKR1CL1	−
chr21	34483981	34496009	TCONS_l2_00017202+TCONS_l2_00017489	−
chr6	9124454	9162010	TCONS_00011445	−
chr20	44058264	44077210	TCONS_00028551+TCONS_00027937	+
chr18	68423514	68438194	TCONS_00026358	+
chr4	3943669	3957148	FAM86EP	−
chr7	149730165	149773744	TCONS_l2_00026265+TCONS_l2_00026266+TCONS_ l2_00026267+TCONS_l2_00026268+TCONS_ l2_00027238+TCONS_l2_00026269+TCONS_ l2_00026270+TCONS_l2_00027239+TCONS_ l2_00027240+TCONS_l2_00026271+TCONS_ l2_00026272+TCONS_l2_00027241	+
chr1	14675450	14677518	TCONS_00000472	−
chr13	85741303	85743317	TCONS_00022078	−
chr10	115787548	115802439	TCONS_00018344	+
chr20	22433900	22447980	TCONS_00028330	−
chr2	227007510	227044778	LOC646736	+
chr22	27853208	27856021	TCONS_00029462	−
chr2	237642025	237663006	TCONS_00003130+TCONS_00003131	+
chr12	64215703	64227164	TCONS_l2_00005718+TCONS_l2_00005719	+
chr9	97094758	97123230	LOC100132077	+
chrX	46062922	46067256	TCONS_00017160	+
chr3	12512285	12518022	TCONS_00005946+TCONS_00005947	+
chr3	70048728	70064449	TCONS_00005539+TCONS_00005416	+
chr4	120299287	120326770	TCONS_l2_00021682	+
chr1	22350487	22352724	TCONS_00000481+TCONS_00001413+TCONS_00001414	−
chr10	92720833	92757505	TCONS_00018022	−
chr4	72687022	72689697	TCONS_00007777	−
chr15	65100372	65100715	TCONS_00023443	+
chr19	38038730	38041971	TCONS_00027356	−
chr2	173117101	173145603	TCONS_00003940	+
chrX	56999943	57001229	TCONS_00016987	+
chr5	169758435	169762104	LOC257358	+
chr5	133304443	133306019	TCONS_00010096	+
chr5	132446761	132450167	TCONS_00009311	−
chr6	32685782	32686612	TCONS_00011291+TCONS_00011292+TCONS_00011293	+
chr16	86223827	86234547	TCONS_00024759+TCONS_00025079+TCONS_00025080	−
chr13	85937738	86118797	LINC00351	+
chr2	140861152	140889349	TCONS_00003411	−
chr5	12554002	12574749	TCONS_00009606	−
chr7	57469684	57472194	TCONS_00013820	−
chr6	154679596	154683278	TCONS_00012337	−
chr7	149253258	149294625	TCONS_00014231	+

chr6	26321469	26332220	TCONS_l2_00024656+TCONS_l2_00024657+TCONS_l2_00024658	−
chr8	61878680	61880307	LOC100130298	−
chr16	55758837	55784123	CES1P2	+
chr5	36871457	36876605	TCONS_00010663	+
chr18	56751133	56753134	TCONS_l2_00011859	+
chr1	142618769	142625251	TCONS_00000292+TCONS_00000293+TCONS_00001133+TCONS_00002148+TCONS_00001134+TCONS_00001135+TCONS_00001136	+
chr18	22515987	22520458	TCONS_00026300	+
chr7	26097439	26101262	TCONS_00013739	−
chr11	60383224	60454621	LINC00301	+
chr7	12713477	12714328	TCONS_00013698	−
chr1	162004995	162009357	TCONS_00001180+TCONS_00000325	+
chr21	44881974	44898103	LINC00313	−
chr1	158130281	158132748	TCONS_00001176	+
chr14	97229347	97257011	TCONS_00022619	+
chr14	101723669	101758324	TCONS_00022403+TCONS_00022635	+
chr7	19152097	19153891	TCONS_00012869	+
chrY	9573895	9596085	TTTY2	+
chr18	74277425	74323162	TCONS_00026377	+
chr5	139120562	139125808	TCONS_00010500+TCONS_00009793	−
chr16	73798065	73802501	TCONS_00024463+TCONS_00024886	+
chr20	24003973	24011949	TCONS_00028137	+
chr14	101536248	101539273	MEG9	+
chr14	104992838	104994118	TCONS_l2_00007938	+
chr5	55609732	55617224	TCONS_00009667	−
chr3	196363400	196366498	TCONS_00007354	−
chr15	32812049	32825942	WHAMMP1	−
chr9	5308118	5311063	TCONS_00016263	−
chr9	110570350	110574037	TCONS_00016126	+
chr5	96559075	96563661	TCONS_00009732	−
chr11	11173944	11177996	TCONS_00019574	−
chr7	56446010	56448602	TCONS_00013809+TCONS_00013810	−
chr12	93493122	93503259	TCONS_00020522	+
chr9	132902889	132906498	TCONS_00015635	−
chr1	182098445	182283196	TCONS_l2_00002728+TCONS_l2_00002729+TCONS_l2_00001664+TCONS_l2_00002730	−
chr2	91925029	91927269	TCONS_l2_00013785	+
chr12	25537425	25539101	TCONS_00020732	−
chr15	101835623	101838894	TCONS_00023567	+
chr1	159931014	159948876	LOC100505633	+
chr4	150587209	150632301	TCONS_00007656	+
chr4	104792988	104799595	TCONS_l2_00020663	+
chr22	48440527	48444366	TCONS_00029492	−
chr5	16442343	16446407	TCONS_00009882	+
chr14	52884400	52889572	TCONS_00023107	−
chr6	122007930	122017769	TCONS_00011941	+

chr7	47622655	47630846	TCONS_00013440	+
chr20	31781411	31798268	BPIFA4P	+
chr13	78236635	78237275	TCONS_00021571	+
chr4	143837531	143839844	TCONS_00008601	−
chr5	123731296	123736088	TCONS_00009499	+
chr1	37920480	37940044	LOC728431	−
chr4	122151606	122155366	TCONS_00008213	+
chr8	69824038	70016425	LOC100505718	−
chr15	42265998	42273657	TCONS_00023386	+
chr9	129032403	129038194	TCONS_l2_00030075+TCONS_l2_00029586	−
chr6	123392316	123393064	TCONS_00011363	+
chr22	45002208	45021299	LINC00229	−
chr14	104755161	104756411	TCONS_l2_00008273+TCONS_l2_00008274	−
chr7	49269733	49294412	TCONS_00013009	+
chr7	57687757	57706334	TCONS_l2_00026601	−
chr8	47490845	47526157	TCONS_l2_00028184+TCONS_l2_00028185+TCONS_l2_00028186+TCONS_l2_00028187+TCONS_l2_00028188	−
chr7	128050311	128090500	TCONS_l2_00026880	−
chr10	19035362	19036782	TCONS_00017796	+
chr3	5671632	5675990	TCONS_00006402	−
chr4	4562120	4577798	TCONS_00009012+TCONS_00009013+TCONS_00009014+TCONS_00009015+TCONS_00009018+TCONS_00009017+TCONS_00009016	−
chr2	6072819	6120350	LOC150622	+
chr8	22532054	22537228	TCONS_00014649	+
chr9	68407368	68410095	TCONS_l2_00029342	−
chr3	84934087	84942530	TCONS_00005553	+
chr7	39585249	39605943	TCONS_00013157+TCONS_00014283	−
chr16	61163021	61185962	TCONS_00024428	+
chr12	128510533	128511677	TCONS_00020991	−
chr1	95007608	95008571	TCONS_00001037	+
chr1	109621258	109627542	TCONS_00001623	−
chr10	116754416	116756352	TCONS_00017871	+
chr5	654144	654462	TCONS_00010868	−
chr6	45633188	45634483	TCONS_00011820	+
chr3	75436009	75441293	TCONS_l2_00019358	−
chr3	153101578	153108386	TCONS_00006281	+
chr20	37842424	37853391	LOC339568	−
chr2	10174464	10177284	TCONS_00004140+TCONS_00004141	−
chr10	58872623	58982557	TCONS_00018523	−
chr5	1597672	1634120	LOC728613	−
chr2	75049907	75059445	TCONS_00004314	−
chr3	52312783	52314314	TCONS_00006853	+
chrX	120152107	120165436	TCONS_l2_00030590	−
chr9	38360424	38376427	TCONS_00015669	+
chr13	43732767	43733602	TCONS_00021542	+
chr15	61935730	61945600	TCONS_00023689	−
chr16	80587205	80603584	TCONS_00024740+TCONS_00024275+TCONS_00024741	−

chr12	121813196	121830215	TCONS_00020596+TCONS_00020597	+
chr20	26157211	26160098	TCONS_00028359	−
chrX	48504419	48521938	TCONS_00017167	+
chr9	65880533	65883885	TCONS_00015821	−
chr1	244129381	244142778	TCONS_00001897	−
chr1	46912345	46915376	TCONS_00000514	−
chr21	24735960	24744550	TCONS_00028974	+
chrY	6317509	6325947	TTTY7	+
chr11	60085057	60085782	TCONS_00019307	+
chr6_cox_ hap2	77359	81728	TCONS_00029956	+
chr6	93689708	93695847	TCONS_00011890	+
chr6	49783206	49792495	TCONS_00011217+TCONS_00011499	−
chr9	14987300	15146399	TCONS_l2_00029903+TCONS_l2_00029211+TCONS_ l2_00029212+TCONS_l2_00029213+TCONS_l2_00029214	−
chr9	44125009	44130454	TCONS_l2_00029295+TCONS_l2_00029941	−
chr6	167604944	167614963	TCONS_l2_00024524+TCONS_l2_00025290	+
chr14	29288806	29299700	TCONS_00022692+TCONS_00022693+TCONS_00022694	−
chr9	33512957	33514771	TCONS_00015799	−
chr9	7208936	7209962	TCONS_00015915	+
chr17	19321236	19327806	TCONS_l2_00010597+TCONS_l2_00011529	+
chr5	113391703	113441459	TCONS_00009486+TCONS_00010060	+
chr6	43858765	43905944	LOC100132354	+
chr3	81257344	81258065	TCONS_00005791	−
chr17	16721307	16725893	TCONS_l2_00011114+TCONS_l2_00011623	−
chr2	186001935	186031910	TCONS_00004518+TCONS_00005289+TCONS_00005290	−
chr5	6582287	6588613	LOC255167	+
chr1	106469905	106565836	TCONS_l2_00001430+TCONS_l2_00002592+TCONS_ l2_00002591+TCONS_l2_00002593	−
chr2	105760571	105761882	TCONS_00002964+TCONS_00002965+TCONS_00002966	+
chr12	133183169	133183977	TCONS_00020662	+
chr5	92355369	92404375	TCONS_00009730	−
chr15	28936497	28944750	TCONS_00023891	+
chr5	146556614	146559486	TCONS_00010526	−
chr20	56578911	56579620	TCONS_00028235	+
chr11	30338482	30344533	TCONS_00019599	−
chr3	55212200	55223242	TCONS_00005519	+
chr3	78101914	78114598	TCONS_00006102	+
chr2	204955180	204974572	TCONS_00003081	+
chr14	42512800	42542003	TCONS_00022725	−
chr3	142830644	142831201	TCONS_00005602	+
chr15	45115622	45124567	TCONS_l2_00009081	−
chr19	45430060	45434643	APOC1P1	+
chr11	78133641	78140882	TCONS_l2_00005134+TCONS_l2_00005385	−
chr5	28286496	28287772	TCONS_00009625	−
chr9	70630786	70652522	TCONS_l2_00029386+TCONS_l2_00029982	−
chr4	6013585	6020234	TCONS_l2_00021036	−
chr2	3891937	3895579	TCONS_l2_00013442	+

chr6	68095844	68096267	TCONS_00011850	+
chr1	118139454	118148443	TCONS_00000615+TCONS_00002449+TCONS_00001642+TCONS_00002450+TCONS_00002451+TCONS_00002452+TCONS_00002453+TCONS_00000616+TCONS_00000617	−
chr4	174563946	174584210	TCONS_l2_00020888	+
chr20	36247700	36251521	LINC00489	−
chr13	39659262	39665864	TCONS_00021759	+
chr11	72920555	72929202	TCONS_00019702	−
chr2	75145004	75170618	TCONS_00003738+TCONS_00002936+TCONS_00004823+TCONS_00002709+TCONS_00002937+TCONS_00004824+TCONS_00004825+TCONS_00004826+TCONS_00002938	+
chr7	84568662	84569351	TCONS_00013877	−
chr4	150075447	150199275	TCONS_l2_00021749+TCONS_l2_00020816	+
chr6	113852320	113864995	TCONS_00011359	+
chr5	102580709	102589507	TCONS_00010428+TCONS_00009741	−
chr2	15808808	15820608	TCONS_00003210	−
chr17	20328042	20334520	TCONS_l2_00011533	+
chr5	6359189	6362527	TCONS_00010258	−
chr5	858994	860350	TCONS_00010872	−
chr3	7925434	7925857	TCONS_00007109	−
chr5	17369334	17375686	TCONS_00009611	−
chr12	128243393	128246816	TCONS_00020989	−
chr5	82683936	82694107	TCONS_00010726+TCONS_00010009	+
chr11	27238617	27240219	TCONS_00019596	−
chr1	182709783	182710939	TCONS_00000698	−
chr11	38653972	38667942	TCONS_00019618+TCONS_00019619+TCONS_00020058	−
chr10	29217508	29264218	TCONS_00018147	+
chr8	103819050	103822203	TCONS_00014499+TCONS_00015332	+
chr20	54748010	54750796	TCONS_00028232	+
chr1	103957501	103968087	TCONS_00000265	+
chr1	233067054	233068773	TCONS_00001330	+
chr2	92075522	92102148	TCONS_l2_00014691	−
chr13	60203617	60207719	TCONS_00022034	−
chr5	57403459	57418168	TCONS_00009673+TCONS_00010349	−
chr19	20408538	20416213	TCONS_l2_00012383	+
chr2	232250256	232259695	TCONS_l2_00014291+TCONS_l2_00015621+TCONS_l2_00014292+TCONS_l2_00014293	+
chr4	2765473	2771553	TCONS_00007956	+
chr10	92862925	92872371	TCONS_00018283	+
chr13	27775343	27776444	TCONS_00022236	−
chr2	170960845	170961555	TCONS_00003930	+
chr20	20951089	20953203	TCONS_00027908	+
chr3	50304073	50304947	TCONS_00005774+TCONS_00006510	−
chr4	6689212	6694797	TCONS_00007971+TCONS_00008745	+
chr1	27202624	27204736	TCONS_00000170	+
chr17	49012350	49022074	TCONS_00025242	−
chr2	162949939	162951364	TCONS_00003040	+

chr18	71095876	71109029	TCONS_00026365	+
chr6	46459789	46500495	TCONS_00011307	+
chr18	68004562	68004915	TCONS_00026726	−
chr2	34292293	34294617	TCONS_00002868	+
chr12	131649556	131697476	LOC116437	+
chr4	11315420	11352918	TCONS_00007986+TCONS_00008753+TCONS_00007987+TCONS_00007988+TCONS_00008754+TCONS_00008755	+
chr9	132337688	132346835	TCONS_l2_00029867+TCONS_l2_00029082+TCONS_l2_00029083+TCONS_l2_00029084	+
chr6	3183052	3196001	LOC100507194	−
chr2	163175544	163176543	TCONS_00004935	+
chr20	46987427	46988560	TCONS_00028424+TCONS_00028058	−
chr5	102865377	102877686	TCONS_00009743	−
chr10	38736775	38739473	TCONS_00018486	−
chr10	8301294	8310268	TCONS_00017923+TCONS_00017731	−
chr20	61775640	61783415	TCONS_00028469	−
chr14	21341079	21343904	TCONS_00022669	−
chr11	59661570	59663119	TCONS_00019306	+
chr21	35339558	35340977	TCONS_00029279+TCONS_00029280	−
chr2	114261182	114273062	TCONS_00002981+TCONS_00004874+TCONS_00004875+TCONS_00002982+TCONS_00002983+TCONS_00004876	+
chr9	92170521	92172058	TCONS_00016058	+
chrX	65219593	65220913	TCONS_00016991	+
chr11	130270905	130271852	TCONS_00020154	−
chr2	177695967	177708327	TCONS_00004508	−
chr2	192657515	192684976	TCONS_00003066+TCONS_00003975	+
chr20	25677708	25715217	TCONS_l2_00016212+TCONS_l2_00016213	+
chr4	74374520	74394250	LOC728040	+
chr11	287702	288773	TCONS_00019179	+
chr20	12930986	12951800	TCONS_00027905+TCONS_00028503+TCONS_00028110+TCONS_00028111	+
chr14	41052979	41098827	TCONS_l2_00008039	−
chr19	42108584	42111870	TCONS_l2_00013326	+
chrX	90562780	90563877	TCONS_l2_00030720	+
chr1	118359009	118363642	TCONS_l2_00001469+TCONS_l2_00001470	−
chr1	51617575	51621124	TCONS_00000939	+
chr10	89832454	89840364	TCONS_00018569	−
chr1	235092978	235095736	TCONS_00000425	+
chr15	98563877	98565376	TCONS_00023543	+
chr4	43135884	43236973	TCONS_00007522	+
chr16	47999593	48005582	TCONS_00024379	+
chr6	94873638	94904376	TCONS_00011340	+
chr5	26382628	26399928	TCONS_00009623	−
chr22	46032074	46036262	TCONS_00029724+TCONS_00029725	−
chr10	72370073	72428508	TCONS_00017839	+
chr2	219841006	219842644	LINC00608	+
chr18	20685925	20686303	TCONS_00026294	+

chr5	90538753	90560495	TCONS_00010401	−
chr1	223564509	223566572	TCONS_00001834	−
chr7	63761528	63763122	TCONS_00014314	−
chr15	23094331	23115254	LOC283683	−
chr8	49277962	49337122	TCONS_l2_00027747+TCONS_l2_00027748+TCONS_l2_00027749+TCONS_l2_00027750+TCONS_l2_00027751+TCONS_l2_00027752	+
chr1	71172136	71252214	TCONS_00000044+TCONS_00000975	+
chr9	134200180	134222466	TCONS_00016201	+
chr13	111516334	111522655	LINC00346	−
chr1	246948952	246949406	TCONS_00002634	−
chr2	186999554	187002215	TCONS_00003966	+
chr13	53507289	53508268	TCONS_00022026	−
chr12	118552481	118573755	TCONS_00020940+TCONS_00021410+TCONS_00021411+TCONS_00020941+TCONS_00021412+TCONS_00021413+TCONS_00020942+TCONS_00021414	−
chr6	28756565	28757297	TCONS_00012134	−
chr10	88281702	88282443	TCONS_00017851+TCONS_00018262	+
chr6	113943171	113971276	TCONS_00012254+TCONS_00012255+TCONS_00012256+TCONS_00012774+TCONS_00012775+TCONS_00012257+TCONS_00012776+TCONS_00011543+TCONS_00011544+TCONS_00012258+TCONS_00012259	−
chr5	173205286	173220885	TCONS_00010837+TCONS_00010838	+
chr5	24352418	24353552	TCONS_00009618	−
chr7	157294016	157309535	TCONS_00013105+TCONS_00014239+TCONS_00014240+TCONS_00014241+TCONS_00013106	+
chr16	28270021	28301674	TCONS_l2_00009650	+
chr8	13487163	13495100	TCONS_00014627+TCONS_00015233+TCONS_00014628+TCONS_00015234	+
chr15	22512033	22512452	TCONS_00023296	+
chr15	96885117	96888057	TCONS_00024016	+
chrX	47531909	47544867	TCONS_00017163	+
chr11	102511869	102552089	TCONS_00019986+TCONS_00019452	+
chr13	25154346	25171812	TPTE2P6	+
chr1	247802918	247803681	TCONS_00001363	+
chr1	240170824	240176560	RPS7P5	+
chr17	72530814	72534060	TCONS_00026160	−
chrY	9225731	9233636	TCONS_l2_00031032+TCONS_l2_00031033	−
chrX	18884025	18884823	TCONS_l2_00030158	+
chr2	208104374	208110611	TCONS_00003087+TCONS_00003088	+
chr3	112021325	112051596	TCONS_l2_00019858+TCONS_l2_00018738	+
chr4	120370776	120375764	TCONS_l2_00021269+TCONS_l2_00021270	−
chr10	8340860	8343630	TCONS_00017783	+
chr22	42545890	42548179	TCONS_l2_00018070+TCONS_l2_00018071	−
chr6	8931543	8941204	TCONS_00011688+TCONS_00012423+TCONS_00012424	+
chr16	71463533	71466202	TCONS_l2_00010265	−
chr15	41901667	41904936	TCONS_00023651+TCONS_00024101	−
chr17	655573	658576	DBIL5P	+
chr10	124130697	124133962	TCONS_00018622	−

chr20	55152619	55171182	TCONS_00027958	+
chr14	103760410	103766231	TCONS_00022894	−
chr1	16972069	16976915	MST1P2	+
chr5	55828977	55832351	TCONS_00010344+TCONS_00010345	−
chr16	86081434	86082946	TCONS_00024756+TCONS_00024757	−
chr14	32669704	32671152	TCONS_00022700	−
chr12	124723833	124727323	TCONS_00020964	−
chr2	242823514	242919427	TCONS_l2_00015632+TCONS_l2_00015633+TCONS_l2_00014335+TCONS_l2_00014336+TCONS_l2_00014337+TCONS_l2_00015634+TCONS_l2_00014338+TCONS_l2_00014339+TCONS_l2_00014340+TCONS_l2_00014341+TCONS_l2_00014342+TCONS_l2_00014343+TCONS_l2_00014344+TCONS_l2_00015635+TCONS_l2_00015636+TCONS_l2_00014345+TCONS_l2_00014346+TCONS_l2_00014347+TCONS_l2_00014348	+
chr12	9502422	9504480	TCONS_00021052	+
chr10	38933913	38982200	TCONS_l2_00004140+TCONS_l2_00004141+TCONS_l2_00004142+TCONS_l2_00003489	−
chr2	138636324	138683977	TCONS_00003408	−
chr9	69494689	69652731	TCONS_l2_00029375+TCONS_l2_00029376+TCONS_l2_00029377+TCONS_l2_00029378+TCONS_l2_00029379+TCONS_l2_00029380+TCONS_l2_00029381+TCONS_l2_00029382+TCONS_l2_00029979	−
chr22	48256420	48257811	TCONS_00029438	+
chr7	158968747	158975451	TCONS_00014243	+
chr5	87564699	87732491	TMEM161B-AS1	+
chr22	48735894	48736582	TCONS_00029622	+
chr1	45672404	45683543	TCONS_00002017	+
chr17	49389970	49412526	TCONS_00025243	−
chr10	67073543	67074728	TCONS_00017834	+
chr4	41978227	41980408	TCONS_00008057	+
chr18	13203782	13216366	TCONS_00026201	−
chr18	23537979	23539215	TCONS_00026462	−
chr1	38512986	38584697	TCONS_l2_00002029+TCONS_l2_00000218+TCONS_l2_00002030+TCONS_l2_00000219	+
chr5	76382623	76444176	ZBED3-AS1	+
chr6	277864	286279	TCONS_00012034	−
chr9	110102599	110109891	TCONS_00016456	−
chr1	185292979	185304171	LOC100288079	−
chr5	44495203	44510384	TCONS_l2_00022884+TCONS_l2_00023724+TCONS_l2_00023725	−
chrX	5571462	5644346	TCONS_l2_00030779+TCONS_l2_00030780+TCONS_l2_00030781+TCONS_l2_00030437+TCONS_l2_00030438	−
chr2	223605212	223642292	TCONS_00004041+TCONS_00004042	+
chr6	113505723	113518840	TCONS_00011926	+
chr1	225657547	225660157	TCONS_00001304	+
chr15	98208535	98213519	TCONS_00023823	−
chr1	56410760	56415792	TCONS_00000207	+
chr8	91516514	91516880	TCONS_00015083	−

chr19	13227694	13228971	TCONS_00027488	+
chr19	36803963	36822620	LINC00665	−
chr19	37742795	37759148	TCONS_l2_00012516+TCONS_l2_00012515+TCONS_l2_00012517+TCONS_l2_00012518+TCONS_l2_00012519+TCONS_l2_00012520+TCONS_l2_00013323+TCONS_l2_00012521+TCONS_l2_00012522+TCONS_l2_00012523+TCONS_l2_00012524	+
chr8	138720633	138729172	TCONS_00014870	+
chr10	71754382	71768162	TCONS_00017994+TCONS_00018945+TCONS_00018946	−
chr3	40684765	40685532	TCONS_00005996	+
chr9	115661341	115665598	TCONS_00016466	−
chr2	241625415	241628617	TCONS_00004071+TCONS_00005044+TCONS_00004072+TCONS_00004073	+
chr1	31331005	31333101	TCONS_l2_00002021	+
chr22	21400249	21418457	LOC400891	+
chr2	176769099	176770230	TCONS_00003442	−
chr9	122735372	122736600	TCONS_00015745	+
chr5	160486911	160509390	TCONS_00010156	+
chrX	57667298	57680873	TCONS_00017185	+
chr2	132795681	132796357	TCONS_00004897	+
chr5	174413298	174414053	TCONS_00009547	+
chr13	19978749	19979239	TCONS_l2_00007486	−
chr8	144868909	144872708	TCONS_l2_00028016	+
chr7	57233377	57247863	GUSBP10	−
chr12	124742379	124750677	TCONS_00020965+TCONS_00020966	−
chr7	74440309	74445630	TCONS_00012913	−
chr2	154194219	154213936	TCONS_00003037	+
chr7	56594599	56611556	TCONS_00013812+TCONS_00012910	−
chr2	86130446	86131926	TCONS_00005191+TCONS_00005192	−
chrY	10035732	10036602	TCONS_l2_00030929+TCONS_l2_00030930	−
chr19	47137991	47142802	TCONS_00027384	−
chr7	153220300	153221300	TCONS_00014011	−
chr1	149065284	149067756	TCONS_l2_00000585+TCONS_l2_00000586	+
chr9	37509147	37510296	TCONS_00015668	+
chr5	38682172	38685228	TCONS_00009386	+
chr1	235108774	235116505	TCONS_00001887+TCONS_00001888	−
chr10	47571665	47575852	TCONS_00018197	+
chr7	156803551	156809118	LOC645249	+
chr16	89314224	89314707	TCONS_l2_00009900	+
chrX	151972983	151974329	TCONS_00017273	+
chr13	69796478	69866286	TCONS_00021563	+
chr12	115026038	115026592	TCONS_l2_00005906	+
chr2	136846226	136869280	TCONS_00003883	+
chr19	45742030	45748605	TCONS_l2_00013151	−
chr6	11909099	11920977	TCONS_00011692	+
chr8	138835266	138841150	TCONS_l2_00028422	−
chr4	60018552	60041864	TCONS_00007541	+
chr11	68943123	68945424	TCONS_00019366	+

chr1	521369	523833	TCONS_00000442	−
chr20	55116823	55130744	TCONS_00028233+TCONS_00028573	+
chr2	96610862	96626412	TCONS_00004336+TCONS_00005201	−
chr18	951160	1050261	TCONS_00026229	+
chr5	177477770	177483269	TCONS_l2_00022669+TCONS_l2_00022670	+
chr21	40388683	40390969	TCONS_00029138	−
chrY	16017648	16018927	TCONS_l2_00030902	+
chr9	35786643	35790429	TCONS_00015664	+
chr4	185764450	185768200	LOC731424	−
chr6	156130117	156133176	TCONS_00012338	−
chrY	25687556	25693043	TCONS_l2_00031057	−
chr4	157607816	157613788	TCONS_00007667	+
chr6	27031383	27037234	TCONS_00012127	−
chr5	117260703	117601730	TCONS_00009493+TCONS_00010073+TCONS_00010074 +TCONS_00009494	+
chr12	46777115	46965148	TCONS_00020388+TCONS_00020389+TCONS_00020 390+TCONS_00020391+TCONS_00020392+TCONS_0 0020393+TCONS_00021112+TCONS_00020394+TCO NS_00020395	+
chr1	39508264	39509883	TCONS_00001989	+
chr12	92751373	92757553	TCONS_00020870	−
chr1	191844625	191980390	TCONS_00000360	+
chr17	68047418	68064235	TCONS_00025127	+
chr7	149597	155427	TCONS_00012862+TCONS_00013286+TCONS_000 12932+TCONS_00012863+TCONS_00012933+TCO NS_00014041	+
chr17	32546137	32578928	TCONS_00025610	−
chr6	53605547	53659072	TCONS_00011508+TCONS_00012190+TCONS_00012191 +TCONS_00012745+TCONS_00011509	−
chr2	91963368	91970153	GGT8P	+
chr13	100139216	100149038	TCONS_00021692+TCONS_l2_00007598+TCONS_000215 20+TCONS_00022096+TCONS_00022097	−
chr12	80818224	80819003	TCONS_l2_00005793	+
chr21	20290252	20308177	TCONS_00028876	−
chr20	60695577	60696630	TCONS_00028254	+
chr20	24180403	24205224	FLJ33581	+
chr6	33857288	33864684	LOC100507584	−
chr13	24911522	24913410	TCONS_00021940	−
chr10	67330425	67332771	TCONS_00018220	+
chr8	37264139	37351420	TCONS_00014973+TCONS_00014974+TCONS_00015426 +TCONS_00014975+TCONS_00015427	−
chr6	33112560	33115544	TCONS_00011294	+
chr10	8442823	8503826	TCONS_00017784	+
chr18	63671549	63672340	TCONS_00026547	−
chr2	21059531	21061421	TCONS_00002846	+
chr6	156318584	156327735	TCONS_00011999	+
chr1	48567387	48648100	SKINTL	−
chr15	64165701	64168589	TCONS_00023697	−
chr1	222215047	222238105	TCONS_00000740	−

chr4	185814154	185820615	LOC100506229	−
chr9	46698574	46705305	TCONS_l2_00029955	−
chr2	220768772	220769635	TCONS_l2_00016094+TCONS_l2_00015126	−
chrX	119277416	119279301	TCONS_00017541	−
chr17	30410198	30424129	TCONS_l2_00010700	+
chr7	52839107	52842788	TCONS_00013448+TCONS_00014134	+
chr9	42468589	42474238	FAM95B1	+
chr6	113749742	113754623	TCONS_00011541+TCONS_00011542	−
chr1	198985262	199045864	TCONS_00000366	+
chrX	72300006	72304474	TCONS_00016993	+
chr1	205831207	205865215	LOC284581	+
chr16	89748722	89752704	TCONS_00024915+TCONS_00024916	+
chr7	127921571	127924015	TCONS_00013950	−
chr7	144740900	144857358	TCONS_00013619+TCONS_00014227+TCONS_00014228 +TCONS_00013620	+
chr6	68936864	69039791	TCONS_00011512	−
chr12	119305042	119305817	TCONS_00020590	+
chr12	78720460	78753526	TCONS_00020216	−
chr17	79885705	79888629	MAFG-AS1	+
chr3	129101677	129118282	RPL32P3	−
chr15	24605885	24638802	TCONS_00023308+TCONS_00023309+TCONS_00023868 +TCONS_00023869	+
chr17	62073508	62075304	TCONS_00025728	−
chr4	104346199	104374914	TCONS_00007580+TCONS_00008860+TCONS_00008181 +TCONS_00008861+TCONS_00008862	+
chr8	50080642	50105812	TCONS_00014695+TCONS_00014696	+
chr17	6756895	6803668	ALOX12P2	+
chr5	144809431	144830662	TCONS_00009533+TCONS_00009534	+
chr12	121625339	121627249	TCONS_00020238	+
chr4	158850634	158951637	TCONS_l2_00021371	−
chr1	110624852	110652861	TCONS_00001092+TCONS_00002116+TCONS_00001093 +TCONS_00001094	+
chr1	36522535	36549583	TCONS_l2_00001143+TCONS_l2_00001144+TCONS_ l2_00001145	−
chr2	107159393	107160753	TCONS_00003811+TCONS_00002968	+
chr21	14823452	14824110	TCONS_l2_00017131	−
chr8	119739223	119741079	TCONS_00015150	−
chr5	38821815	38845924	TCONS_l2_00022858+TCONS_l2_00022859+TCONS_ l2_00022860	−
chr12	105789468	105806934	TCONS_00020555	+
chr3	55369490	55384943	TCONS_00005776	−
chr7	98836534	98855779	TCONS_l2_00027181	+
chr10	789932	790898	TCONS_00017754	+
chr15	65526818	65540781	TCONS_00023702+TCONS_00024142	−
chr6	74364304	74365877	TCONS_00011856	+
chr1	96979646	96989651	TCONS_00001055+TCONS_00002104	+
chr12	15049541	15052183	TCONS_00020342	+
chr18	12073319	12075074	TCONS_00026283	+
chr15	73939905	73947347	TCONS_00023458	+

chr2	52215564	52332226	TCONS_l2_00013631	+
chr10	31109147	31110214	TCONS_00018467	−
chr5	1851064	1851611	TCONS_00009324	+
chr1	177320721	177339442	TCONS_l2_00000700+TCONS_l2_00002247+TCONS_l2_00000701	+
chr3	81889698	81972012	TCONS_00005552+TCONS_00006109+TCONS_00006899+TCONS_00006110	+
chr4	188891435	188892472	TCONS_l2_00020957	+
chr5	27570754	27575000	TCONS_00009912	+
chr22	35099117	35100876	TCONS_00029476	−
chr7	134944533	134951028	TCONS_00013978	−
chr4	184718946	184719817	TCONS_l2_00020925	+
chr4	37075393	37135530	TCONS_l2_00020480+TCONS_l2_00020481+TCONS_l2_00020482+TCONS_l2_00020483+TCONS_l2_00021571+TCONS_l2_00021572+TCONS_l2_00020484	+
chr6	14280358	14285376	TCONS_00011456	−
chr4	11466108	11815582	TCONS_00008756+TCONS_l2_00021523+TCONS_00007472+TCONS_00007991+TCONS_00007992+TCONS_00007993+TCONS_00008757+TCONS_00008758+TCONS_00008760+TCONS_00008761+TCONS_00007473+TCONS_00007474+TCONS_00007994+TCONS_00007475+TCONS_00007476+TCONS_00007477	+
chr9	123555775	123561009	LOC100288842	+
chr2	109328731	109329171	TCONS_l2_00014773	−
chr13	19919189	19920889	LINC00421	+
chr9	11313363	11325750	TCONS_00015919	+
chr1	238643684	238649317	LOC339535	−
chr22	21419901	21424360	TCONS_l2_00017628	+
chr3	19045197	19053734	TCONS_00006453+TCONS_00007128	−
chr19	21780079	21797405	TCONS_00026949	+
chr19	58787665	58788877	TCONS_00027643	+
chr21	17442842	17982094	LINC00478	+
chr3	197387436	197392628	TCONS_00005934	−
chr1	104668179	104672483	TCONS_00001083	+
chr3	108897012	108904108	LINC00488	+
chr4	187812099	187831845	TCONS_l2_00020946	+
chr11	121899035	121938108	TCONS_00019783+TCONS_00020141+TCONS_00020142+TCONS_00020143+TCONS_00020144+TCONS_00019784	−
chr9	37073528	37075789	TCONS_00015805	−
chr21	15095903	15097830	TCONS_l2_00016971	+
chr20	58713548	58897314	LOC284757	+
chr12	24857334	24893683	TCONS_00020358	+
chr5	56593591	56595368	TCONS_00010347	−
chr6	28287011	28288162	TCONS_l2_00024683	−
chr11	79306950	79311418	TCONS_00019404	+
chr14	78122343	78123921	TCONS_00022805	−
chr15	83681104	83683005	TCONS_00023489	+
chr2	85958823	85967668	TCONS_00004324	−
chr2	11547409	11561393	TCONS_00003577	+

chr20	23899188	23909544	TCONS_l2_00016522	−
chr8	59168331	59185146	TCONS_00014516+TCONS_00015016	−
chr1	241577019	241597079	TCONS_l2_00000923+TCONS_l2_00000924+TCONS_l2_00000925+TCONS_l2_00002345	+
chr7	82323790	82324640	TCONS_00013875	−
chr1	96403457	96488437	TCONS_00000255+TCONS_00000256+TCONS_00001053+TCONS_00002102+TCONS_00001054+TCONS_00000257+TCONS_00000258	+
chr1	143718513	147931980	FLJ39739	−
chr15	24409926	24415053	PWRN2	−
chr11	112352898	112356504	TCONS_00019477	+
chr10	122685059	122740213	TCONS_00018060	−
chrX	70403409	70418025	TCONS_00017066	−
chr4	172207154	172209571	TCONS_00008322	+
chr16	86193597	86200988	TCONS_00024491	+
chr3	23635941	23643659	TCONS_00005965+TCONS_00005966	+
chr1	201424770	201430083	TCONS_00001771	−
chr3	112769760	112782290	TCONS_00005464	−
chr10	4790107	4806336	TCONS_00018417+TCONS_00018418	−
chr19	16013126	16014007	TCONS_l2_00012890	−
chr4	124949081	124949446	TCONS_00008230	+
chr2	214020530	214032436	TCONS_00003490+TCONS_00004569	−
chr9	76866286	76889937	TCONS_00016359	−
chr16	67011992	67021978	TCONS_00024711	−
chr10	7138249	7139637	TCONS_l2_00002879+TCONS_l2_00003892	+
chr4	62999462	63027983	TCONS_00008091+TCONS_00008798+TCONS_00008799+TCONS_00008800+TCONS_00008801	+
chr7	35297992	35299340	TCONS_00013155	−
chr3	125985639	126010400	TCONS_00005580+TCONS_00006201+TCONS_00005581+TCONS_00006202	+
chrX	119264664	119270963	TCONS_00017539+TCONS_00017540	−
chr15	89128802	89148433	TCONS_00023252+TCONS_00023772+TCONS_00023773+TCONS_00023774	−
chr13	38444084	38446356	TCONS_00021755	+
chr2	118889704	118965745	TCONS_l2_00015943+TCONS_l2_00014852+TCONS_l2_00014853+TCONS_l2_00015944+TCONS_l2_00014854+TCONS_l2_00014855	−
chr6	141079071	141105797	TCONS_l2_00024454	+
chr4	62009374	62019680	TCONS_00007542+TCONS_00007543+TCONS_00008797	+
chr9	104652669	104654712	TCONS_00016106	+
chr12	77511553	77514022	TCONS_00020481	+
chr6	169434292	169435737	TCONS_00011621	−
chr4	174428627	174434499	TCONS_00008327+TCONS_00008328	+
chr12	111834639	111841107	TCONS_00020562	+
chr6	21354642	21355527	TCONS_00011274	+
chr14	56247853	56263392	LINC00520	−
chr7	48120412	48122554	TCONS_00013798	−
chr1	16679040	16679908	TCONS_00000847	+
chr2	106575540	106578462	TCONS_00003358	−

chr9	129987565	129998173	TCONS_00016695	+
chr5	4967877	4974687	TCONS_l2_00023652+TCONS_l2_00022763	−
chr7	55774879	55780208	TCONS_l2_00026574	−
chr13	60849731	60851370	TCONS_l2_00006877	+
chr1	220667811	220674043	TCONS_00001284	+
chr10	70817727	70830098	TCONS_00018746	+
chr13	84714737	85180903	LINC00333	+
chr18	69500337	69504170	TCONS_l2_00011879	+
chr3	61284244	61299032	TCONS_00006040	+
chr6	168080306	168096970	TCONS_00011242+TCONS_00011243	−
chr2	42104214	42105095	TCONS_00003259	−
chr5	162180833	162181273	TCONS_00010158	+
chr4	189678833	189707064	TCONS_l2_00021803+TCONS_l2_00020967+TCONS_00008383+TCONS_00007702+TCONS_00008384	+
chr5	92011663	92023961	TCONS_00009728	−
chr22	17053606	17055481	TCONS_l2_00017541+TCONS_l2_00017542	+
chr12	40959314	40964628	TCONS_l2_00006587+TCONS_l2_00005598	+
chr19	15674921	15684691	TCONS_l2_00012336	+
chr5	23013157	23014636	TCONS_00009370	+
chr7	67156045	67162016	TCONS_00013189	−
chr2	201577028	201599900	LOC100507140	−
chr1	48544459	48547868	TCONS_00000518	−
chr20	46579543	46582691	TCONS_00027938+TCONS_00028556	+
chr5	95300703	95322015	TCONS_l2_00023832	−
chr16	86962017	86964962	TCONS_00024774	−
chrX	117956470	117956982	TCONS_00017365	−
chrX	1780739	1781265	TCONS_00017121	+
chr7	32511483	32512531	TCONS_l2_00025783	+
chr3	14240060	14242620	TCONS_00005744	−
chr6_dbb_hap3	4125589	4129180	TCONS_00029965	+
chr1	48519217	48532873	TCONS_00000196+TCONS_00000197	+
chr12	47643134	47652378	TCONS_00020398	+
chr2	222563382	222565211	TCONS_00004037	+
chr9	137110234	137112139	TCONS_00016892	−
chr13	30993656	30996374	TCONS_00021627	−
chr3	164326845	164337782	TCONS_00006703	−
chr10	6182721	6183545	TCONS_00018097	+
chr11	28700730	28705016	TCONS_00019142	+
chr2	234731113	234732317	TCONS_l2_00014301	+
chr8	99188751	99192272	TCONS_00014803	+
chr4	86167563	86168180	TCONS_l2_00020628	+
chr4	38565624	38572292	TCONS_00007516	+
chr7	4645446	4646569	TCONS_00013683	−
chr4	175750819	175802532	TCONS_00007413+TCONS_00008339+TCONS_00007414+TCONS_00008340	+
chr8	8086092	8102387	FAM86B3P	+
chr20	24660183	24661627	TCONS_00027916	+

chr15	74800497	74806312	TCONS_00023466	+
chr7	26584208	26590534	TCONS_00013748	−
chr13	103532449	103548383	METTL21EP	+
chr1	213665644	213719790	TCONS_00000388	+
chr11	82843319	82859757	TCONS_l2_00004688	+
chr14	35396821	35442867	TCONS_l2_00008015+TCONS_l2_00008016	−
chr10	99094513	99102955	TCONS_00018786+TCONS_00018294	+
chr7	56491397	56516068	LOC650226	−
chr3	72712458	72713404	TCONS_00007189+TCONS_00007190	−
chr1	63361680	63367310	TCONS_00000215	+
chr9	98892212	98894541	TCONS_00016088+TCONS_00016089	+
chr5	17063337	17089310	TCONS_00010631+TCONS_00009885	+
chr20	42521159	42524108	TCONS_00028163+TCONS_00028164+TCONS_00027934	+
chr6	97233013	97243697	TCONS_00011341	+
chr12	90494479	90501571	TCONS_00020512	+
chr2	173162904	173232618	TCONS_00004946+TCONS_00003941+TCONS_00003942 +TCONS_00003050+TCONS_00003943	+
chr1	121138614	121204983	TCONS_00000289+TCONS_00001132+TCONS_00000290 +TCONS_00002147	+
chr1	245293033	245295590	TCONS_00001349	+
chr1	714143	745440	TCONS_l2_00000004+TCONS_l2_00000003+TCONS_ l2_00001938+TCONS_l2_00001939+TCONS_ l2_00001940+TCONS_l2_00001941+TCONS_l2_00001942	+
chr5	767618	769262	TCONS_l2_00022725	−
chr20	55849537	55869440	TCONS_00028452+TCONS_00028710	−
chr5	29141837	29143508	TCONS_00010288	−
chr8	134584414	134586014	TCONS_00014502	+
chr6	160536101	160539659	TCONS_00012819	−
chr5	172663704	172671727	TCONS_00010558	−
chr6	168700863	168702794	TCONS_00011619	−
chr1	36916215	36917200	TCONS_00000890	+
chr1	229455027	229455445	TCONS_00001320	+
chr1	48283738	48285909	TCONS_00000195	+
chr3	24079061	24086331	TCONS_00006456	−
chrX	155255811	155257756	TCONS_l2_00030643+TCONS_l2_00030644	−
chr9	38694281	38703988	TCONS_l2_00029272	−
chr10	132001138	132001392	TCONS_00018070	−
chr11	77185455	77186106	TCONS_00019715	−
chr12	104781715	104795844	TCONS_00020551	+
chr3	183602712	183605782	TCONS_l2_00020003	+
chr10	11715227	11722506	TCONS_00017703	+
chr1	1870244	1877855	TCONS_l2_00000982	−
chr15	67332912	67340583	TCONS_00023265	+
chrX	107963202	107968338	TCONS_00017361	−
chr15	31110239	31119986	HERC2P10	+
chr4	8508555	8514337	TCONS_l2_00021521+TCONS_l2_00020399	+
chr9	111463650	111465372	TCONS_00015734	+
chr3	182200735	182206014	TCONS_00005675	+

chr1	182376756	182383948	LINC00272	+
chr10	25940662	25975337	TCONS_00018686+TCONS_00017799	+
chr6	134758854	134825158	LOC154092	+
chr22	42760406	42765214	TCONS_00029485+TCONS_00029486+TCONS_00029350	−
chr2	16909391	16948822	TCONS_00002839+TCONS_00002840	+
chr12	3411880	3417067	TCONS_l2_00006056	−
chrY	2871037	2970313	LINC00278	+
chr3	194548619	194553321	TCONS_00005707	+
chr13	20676840	20677369	TCONS_00021527	+
chr3	147657792	147724874	TCONS_00005435+TCONS_00005612+TCONS_00006257	+
chr9	134222638	134224656	TCONS_00016496	−
chr4	31544377	31560351	TCONS_00008774	+
chr11	79848192	79868111	TCONS_00019405	+
chr2	184769256	184813839	TCONS_00003065	+
chr6	32358287	32361468	HCG23	+
chr12	82532346	82537292	TCONS_00020857	−
chr4	80584915	80617991	TCONS_00007567	+
chr7	112658441	112699063	TCONS_00013568	+
chr6	2272911	2273285	TCONS_00012046	−
chr9	118501942	118661023	TCONS_00015739+TCONS_00016140	+
chr13	30890497	30894040	TCONS_00021619	−
chr1	220552594	220554659	TCONS_00001282	+
chr1	189837131	189837658	TCONS_00000703	−
chr1	90090408	90098453	FLJ27354	−
chr9	93881420	93925369	TCONS_00015847	−
chr10	44755050	44757261	TCONS_00017963	−
chr16	77028205	77042204	TCONS_00024733	−
chr1	180864246	180869874	TCONS_00001212	+
chr11	71116792	71134400	FLJ42102	−
chr13	45958266	45961128	TCONS_00021644	−
chr4	101581436	101596270	EMCN-IT3	−
chr15	68775547	68779860	TCONS_00024147+TCONS_00023714+TCONS_00023717 +TCONS_00024148	−
chrX	123466548	123470714	TCONS_l2_00030347	+
chr21	32996944	32997467	TCONS_00028993	+
chr7	130082644	130083216	TCONS_00013590	+
chr11	65190269	65194003	NEAT1	+
chr17	54586446	54587918	TCONS_00025699	−
chr15	45742463	45771942	TCONS_00023224	+
chr7	27226866	27232305	TCONS_00013751	−
chr8	5715806	5757253	TCONS_00014915	−
chrX	65041569	65041930	TCONS_l2_00030513	−
chr5	70671612	70681820	PMCHL2	+
chr7	35411878	35414509	TCONS_00013775	−
chr4	172474151	172485418	TCONS_00007897	−
chr8	104493481	104511823	TCONS_00015128+TCONS_00015129+TCONS_00015130	−
chr13	112967781	112976072	TCONS_00021603	+
chr8	47995198	47999207	TCONS_00014687	+

chr14	62584075	62600903	LINC00643	+
chr10	81263829	81266037	TCONS_l2_00004211	−
chr14	49992410	50010274	TCONS_00022739	−
chr1	208132709	208134955	TCONS_00001254	+
chr15	22447232	22473375	TCONS_l2_00008913+TCONS_l2_00008914+TCONS_l2_00008915+TCONS_l2_00008916+TCONS_l2_00008917+TCONS_l2_00009483	−
chr2	24626593	24633454	TCONS_00003602	+
chr3	39462167	39465596	TCONS_00005995	+
chr2	65073264	65090760	TCONS_00002754	−
chr16	73804649	73809182	TCONS_00024464	+
chr13	20161258	20162490	TCONS_00021526	+
chr19	34286903	34287633	TCONS_00026997	+
chr3	141382041	141439915	TCONS_l2_00019899+TCONS_l2_00019900	+
chr12	104565675	104571402	TCONS_00020549	+
chr4	140489678	140499616	TCONS_00008271+TCONS_00008899	+
chr12	108229330	108232440	TCONS_00020909+TCONS_00020910	−
chr12	24920107	24927568	TCONS_00020359	+
chr22	23701793	23724313	CES5AP1	−
chr2	200704678	200708029	TCONS_00004973	+
chrY	8506335	8513440	TCONS_00017609	+
chr15	52375567	52378754	TCONS_00023404	+
chr9	118426662	118454445	TCONS_00016138+TCONS_00016139	+
chr3	196358369	196359460	TCONS_00005931+TCONS_00006767	−
chr1	110825156	110825722	TCONS_00000272	+
chr10	119585588	119590667	TCONS_00019037+TCONS_00018616+TCONS_00018617	−
chr2	8806773	8810423	TCONS_00004690	+
chr6	116918004	116937516	TCONS_00012263	−
chr10	25401987	25406286	TCONS_00017798	+
chr13	19292907	19293869	TCONS_00021607	−
chr12	126630053	126650670	TCONS_00020970+TCONS_00021427+TCONS_00021428+TCONS_00021429+TCONS_00021430+TCONS_00021431	−
chr12	16814696	16902754	TCONS_00021085	+
chr13	104073592	104080432	TCONS_l2_00006986+TCONS_l2_00006987+TCONS_l2_00007459	+
chr9	139736279	139736848	TCONS_00016727	+
chr2	16060895	16062162	TCONS_00004153	−
chr20	58609422	58621469	TCONS_l2_00016358	+
chr8	49502961	49507181	TCONS_00014541	+
chr2	45220642	45224037	TCONS_l2_00013610	+
chr4	110351119	110354973	SEC24B-AS1	−
chr17	38673267	38683253	TCONS_00025382+TCONS_00025120+TCONS_00025383	+
chr21	38004979	38009331	TCONS_00028921	−
chr13	85639222	85653055	TCONS_l2_00007579+TCONS_l2_00007277	−
chr5	25404842	25446034	TCONS_l2_00023691+TCONS_l2_00023692+TCONS_l2_00023693	−
chr6	123193680	123194423	TCONS_00011944	+
chr3	42055124	42075359	TCONS_00006818	+

chr12	116795421	116796812	TCONS_00020939	−
chr11	107181806	107186290	TCONS_00019750	−
chr4	175444202	175451355	TCONS_00008337+TCONS_00008338	+
chr6	160887587	160932156	LPAL2	−
chr7	63019284	63023112	TCONS_l2_00025914	+
chr20	31728546	31733779	TCONS_00028152	+
chr18	59568318	59577618	TCONS_00026542	−
chr5	97217124	97258399	TCONS_00009468+TCONS_00009469	+
chr21	44885009	44887755	TCONS_00029049+TCONS_00028853	+
chr3	120587125	120626902	TCONS_00006621	−
chr3	65151134	65160500	TCONS_00006526	−
chr13	46687933	46695391	TCONS_00022273	−
chr20	31938669	31941866	TCONS_l2_00016762	+
chr3	83433607	83486868	TCONS_00006112+TCONS_00006113+TCONS_00006900	+
chr8	83020804	83072758	TCONS_00015057+TCONS_00015056	−
chr3	67291605	67292386	TCONS_00006528	−
chr16	32334099	32335382	TCONS_00024632	−
chr5	50154594	50155854	TCONS_00009658	−
chr1	173833039	173837125	GAS5	−
chr2	127782107	127788497	TCONS_l2_00013965	+
chr4	7099151	7105103	FLJ36777	−
chr7	141940556	141946886	MOXD2P	−
chr12	132674531	132675482	TCONS_00020661	+
chr10	80455819	80456779	TCONS_00017999	−
chr21	25415572	25422601	TCONS_00028808+TCONS_00028809	+
chr17	33615	50139	TCONS_l2_00011610+TCONS_l2_00011032+TCONS_ l2_00011033	−
chr6	94416801	94486199	TSG1	+
chr1	29459862	29462475	TCONS_00000873+TCONS_00000874	+
chr2	9814152	9848542	TCONS_00002817	+
chr4	146117592	146124660	TCONS_00008604	−
chr14	96039924	96048611	TCONS_00022614+TCONS_00022356+TCONS_00022615 +TCONS_00023048	+
chr17	41455317	41458968	TCONS_00025395	+
chr3	72553957	72599703	TCONS_00005788	−
chr6	22660654	22664446	TCONS_00011731	+
chr2	95873283	95889565	TCONS_l2_00015400+TCONS_l2_00013800+TCONS_ l2_00013801	+
chr2	121060714	121085769	TCONS_00003831+TCONS_00002990+TCONS_00003832	+
chr10	6622387	6627323	PRKCQ-AS1	+
chr6	31021227	31027655	HCG22	+
chr8	17659188	17679981	TCONS_00014631+TCONS_00014632+TCONS_00014633 +TCONS_00015235	+
chrX	64295361	64333726	TCONS_00017187+TCONS_00017188	+
chr4	185210996	185215360	TCONS_00009187+TCONS_00008688	−
chr12	103513592	103518106	TCONS_00020900	−
chr13	27752302	27760746	TCONS_00021946	−

chr5	1171450	1178720	TCONS_l2_00022742+TCONS_l2_00023629+TCONS_l2_00023630+TCONS_l2_00022743+TCONS_l2_00023631	−
chr3	40914087	40916190	TCONS_00005997	+
chr2	123823727	123831057	TCONS_00003395	−
chr20	47465977	47470614	TCONS_00028430	−
chr20	61197337	61198501	TCONS_00028585	+
chr4	3051281	3064341	TCONS_00008412	−
chr3	117509520	117512612	TCONS_00005578	+
chr4	181448689	181453970	TCONS_00008670	−
chr15	79000312	79020109	TCONS_00023746+TCONS_00023747	−
chr12	9518908	9520002	TCONS_00021053	+
chr1	232449868	232450795	TCONS_00001326	+
chr2	10857899	10861078	TCONS_00002819	+
chr9	112937186	112938141	TCONS_00016461	−
chr4	63031380	63036192	TCONS_00008092	+
chr11	72964638	72970234	TCONS_00019385	+
chr1	54982085	54982932	TCONS_00000525	−
chr20	46972053	46974725	TCONS_00028199	+
chr7	46472579	46484899	TCONS_00013163	−
chr1	168433452	168464882	TCONS_00000673	−
chr20	45085596	45086250	TCONS_l2_00016635	−
chr17	49412622	49413601	TCONS_00025190	+
chr17	46710869	46724385	TCONS_00025230+TCONS_00025231+TCONS_00025232+TCONS_00025233	−
chr13	30170020	30170919	TCONS_00021740	+
chr4	147032734	147034564	TCONS_00008278+TCONS_00008279	+
chr4	28437071	28601897	TCONS_l2_00021553+TCONS_l2_00020464	+
chr5	5034472	5070115	LOC340094	+
chr10	9800746	9801200	TCONS_00017924	−
chr21	33917053	33919308	TCONS_00028995+TCONS_00028996	+
chr9	44345604	44402079	TCONS_l2_00029300	−
chr6	168152126	168152763	TCONS_00012018	+
chr14	27278523	27296976	TCONS_00022445+TCONS_00022939+TCONS_00022446+TCONS_00022940+TCONS_00022447	+
chr15	36743229	36777325	TCONS_00023349	+
chr17	46124254	46125435	TCONS_00025686+TCONS_00025687+TCONS_00025688	−
chr8	29722186	29735477	TCONS_00014661+TCONS_00014662	+
chr18	1963908	1972876	TCONS_00026231	+
chr1	184632075	184634810	TCONS_00001219	+
chr4	112561651	112569964	TCONS_00007594	+
chr10	106375860	106376731	TCONS_00017862	+
chr8	59116115	59131915	TCONS_00014719+TCONS_00014720	+
chrX	13405671	13438072	TCONS_00017293+TCONS_00017488	−
chr8	77414974	77509924	TCONS_00015044	−
chr11	1049391	1057094	TCONS_00019194+TCONS_00019195	+
chr6	138144831	138182053	TCONS_00012608+TCONS_00012609+TCONS_00012610	+
chr5	67482632	67485177	TCONS_00010944+TCONS_00010360	−
chr19	22638827	22715295	TCONS_l2_00012940+TCONS_l2_00012941	−

chr5	167700929	167704729	TCONS_00010547	−
chr6	30259314	30271912	TCONS_00012453	+
chr18	36786888	37331959	LINC00669	−
chr2	6506142	6515554	TCONS_00003175	−
chr7	128171398	128266709	TCONS_l2_00026190+TCONS_l2_00026191+TCONS_l2_00026192+TCONS_l2_00026193+TCONS_l2_00026194+TCONS_l2_00026195+TCONS_l2_00026196+TCONS_l2_00026197+TCONS_l2_00026198+TCONS_l2_00026199+TCONS_l2_00026200+TCONS_l2_00026201+TCONS_l2_00026202+TCONS_l2_00026203+TCONS_l2_00026204+TCONS_l2_00026205+TCONS_l2_00026206+TCONS_l2_00026207+TCONS_l2_00026208	+
chr14	77507407	77535846	TCONS_00022395	+
chr1	106015903	106029242	TCONS_00001617+TCONS_00002424	−
chr7	27225027	27228912	HOXA11-AS	+
chr2	220551709	220579408	TCONS_l2_00015607	+
chr3	186528669	186543310	TCONS_00005910	−
chr18	74401986	74405592	TCONS_00026196	+
chr1	92010899	92035089	TCONS_00001584	−
chr2	67776347	67777990	TCONS_00003725	+
chr2	42300323	42301745	TCONS_00003655	+
chr2	21350513	21367638	TCONS_l2_00013506	+
chr8	82608603	82610929	TCONS_00014559	−
chr10	7575169	7578527	TCONS_00017781	+
chr21	10164261	10166109	TCONS_00028962	+
chr6	27729523	27730966	LOC100131289	+
chr17	37081421	37085637	LINC00672	+
chr10	110469961	110600418	TCONS_00018328+TCONS_00017868+TCONS_00018329	+
chr1	230011297	230027874	TCONS_l2_00002792+TCONS_l2_00001842+TCONS_l2_00001843+TCONS_l2_00001844+TCONS_l2_00001845	−
chr13	81724538	81801108	TCONS_00022067+TCONS_00022068	−
chr2	7811735	7812377	TCONS_00003190	−
chr6	50061424	50066995	TCONS_00011310	+
chr6	26569552	26574925	TCONS_00011164	+
chr12	68323054	68327145	TCONS_00020461	+
chr12	128366162	128383184	FLJ37505	+
chr14	52306567	52311050	TCONS_00022743	−
chr2	139658590	139659807	TCONS_l2_00014935	−
chr5	124255640	124257291	TCONS_00009501	+
chr21	47395659	47397653	TCONS_00029187	−
chr1	756056	758975	TCONS_00001909+TCONS_00000779	+
chr1	11418037	11424587	TCONS_00000838	+
chr1	320162	321056	TCONS_00000120	+
chr14	75761107	75763111	LOC731223	+
chr6	127195166	127198827	TCONS_00011947	+
chr2	208973041	208977232	TCONS_l2_00016074	−
chr8	118724630	118725297	TCONS_00015147	−
chr9	44992703	44997587	TCONS_l2_00028747	+

chr11	123325106	123351603	TCONS_l2_00005340+TCONS_l2_00004816	+
chr7	63386113	63411851	TCONS_l2_00025926	+
chr15	62352738	62353462	TCONS_00023231	+
chr4	13990450	14094956	TCONS_00007999+TCONS_00008000	+
chr3	180565317	180588352	TCONS_00006715	−
chr5	123731640	123736348	TCONS_00009772	−
chr1	50731521	50745851	TCONS_00001491	−
chrX	49121664	49123331	TCONS_00017321	−
chr19	51718770	51719361	TCONS_00027083	+
chr8	57014015	57014848	TCONS_l2_00028203+TCONS_l2_00028204	−
chr17	66097696	66132070	LINC00674	+
chr3	195663403	195673039	TCONS_l2_00019097	+
chr10	130803350	130804436	TCONS_00018640	−
chr1	214427429	214452637	TCONS_00001818+TCONS_00001819	−
chr14	37125439	37126057	TCONS_00022475	+
chr19	23115571	23128510	TCONS_00027232	−
chr1	212341549	212363601	TCONS_00000386	+
chr3	126036819	126041638	TCONS_l2_00019874+TCONS_l2_00018819	+
chr22	38428172	38430235	TCONS_00029482	−
chr1	2357419	2362089	TCONS_00000138	+
chr4	135855418	135868307	TCONS_l2_00020770+TCONS_l2_00020771	+
chr1	219086099	219089526	TCONS_00000731	−
chr22	23775735	23806986	TCONS_l2_00017638+TCONS_l2_00018190+TCONS_ l2_00018191+TCONS_l2_00017639+TCONS_ l2_00018192+TCONS_l2_00018193+TCONS_l2_00017640	+
chr7	174920	176013	TCONS_00012934	+
chr4	23562546	23770275	TCONS_00007491+TCONS_00007492	+
chr1	187061974	187329455	TCONS_00000350	+
chr22	47741319	47769291	TCONS_00029436	+
chr11	71825906	71832451	TCONS_00019944+TCONS_00019945	+
chr14	24081354	24089221	TCONS_00022428	+
chr10	72762837	72765495	TCONS_00018949+TCONS_00018950	−
chr2	172167776	172170351	TCONS_00003048	+
chr9	130342616	130343098	TCONS_00016159	+
chr10	107446143	107447532	TCONS_l2_00003230+TCONS_l2_00003231	+
chr9	137474325	137477036	TCONS_00015771	+
chr2	216318430	216328594	TCONS_00003098+TCONS_00005009	+
chr13	19246967	19250303	TCONS_00021522	+
chr3	27844253	27932792	TCONS_00006809+TCONS_00005502+TCONS_00005503 +TCONS_00005504+TCONS_00006810	+
chr2	123822897	123825298	TCONS_00002998	+
chr4	78315645	78415440	TCONS_00007784	−
chr1	38607365	38628221	TCONS_00000894+TCONS_00001987	+
chr2	111160496	111181393	LOC100288570	−
chr10	19425689	19444121	TCONS_l2_00002904	+
chr11	29319915	29339844	TCONS_l2_00004945	−
chr2	69135047	69145027	TCONS_00004299	−
chr13	21769075	21771935	TCONS_00021712	+

chr2	132176511	132182503	TCONS_l2_00014902	−
chr5	122081291	122082649	TCONS_00010456	−
chr16	32772018	32784809	TCONS_l2_00009685+TCONS_l2_00009686+TCONS_l2_00010407+TCONS_l2_00009687+TCONS_l2_00009688	+
chr9	33167637	33172203	TCONS_00016752	−
chr6	29091987	29096685	TCONS_00011281	+
chr17	20404827	20407811	KRT16P3	−
chr2	133062359	133080309	TCONS_l2_00015491+TCONS_l2_00013999	+
chr19	43783155	43788569	TCONS_l2_00013124+TCONS_l2_00013125	−
chr4	11370661	11375365	TCONS_l2_00020419+TCONS_l2_00020420	+
chr4	184772683	184773868	TCONS_l2_00020926	+
chr2	234702857	234730909	TCONS_l2_00014299+TCONS_l2_00014300	+
chr5	4033826	4041877	TCONS_l2_00023648	−
chr2	17349864	17351882	TCONS_00003587	+
chr17	2591628	2592285	TCONS_00025272	+
chr6_mcf_hap5	4181125	4184690	TCONS_00029977	+
chr6	38130577	38131580	TCONS_00011483	−
chrX	134555868	134560225	LINC00086	+
chrX	71089382	71091271	TCONS_00017336	−
chr12	80496697	80497140	TCONS_l2_00006336	−
chr18	70992176	71017124	LOC100505817	+
chr15	20456666	20462694	TCONS_l2_00008908	−
chr18	53453841	53462359	TCONS_00026335+TCONS_00026623	+
chr11	58897610	58903816	TCONS_00019641+TCONS_00019642+TCONS_00019643	−
chr3	70216957	70221713	TCONS_00006086	+
chr5	34384488	34415455	TCONS_00009920	+
chr1	60429956	60433906	TCONS_00001510	−
chr17	20747401	20747792	TCONS_l2_00011635	−
chr11	13862201	13869500	TCONS_00019239	+
chr4	185193150	185195410	TCONS_00008950	+
chr3	168746750	168749572	TCONS_00006707	−
chrY	23479732	23480448	TCONS_l2_00030911	+
chr1	213107182	213108781	TCONS_00001814	−
chr2	91933014	91943767	TCONS_l2_00013786+TCONS_l2_00013787	+
chr20	9966736	9987764	TCONS_00027899	+
chr13	53673540	53704701	TCONS_00022027+TCONS_00022028	−
chr4	134792401	134931845	TCONS_00007836	−
chr8	119728690	119737625	TCONS_00015492+TCONS_00015149	−
chr11	18230685	18235111	LOC494141	+
chrY	6311475	6315118	TTTY21	+
chr11	80457209	80473820	TCONS_00020107+TCONS_00019718+TCONS_00020108	−
chr16	34685257	34686664	TCONS_00024377	+
chr9	68995847	68998913	TCONS_00015999	+
chr5	42153043	42175444	TCONS_00010920+TCONS_00010921+TCONS_00010922+TCONS_00010923+TCONS_00010314+TCONS_00010315+TCONS_00009276+TCONS_00010316+TCONS_00010317+TCONS_00010925+TCONS_00010924	−

chr4	58462106	58524966	TCONS_00007761+TCONS_00007762	−
chr12	126920761	126921631	TCONS_00020971	−
chr2	82704259	82765835	TCONS_00003330	−
chr13	19931515	19933464	TCONS_l2_00006698	+
chr2	11528149	11530493	TCONS_00003206	−
chr18	51094569	51104096	TCONS_00026331	+
chr4	171014149	171021478	TCONS_l2_00020871	+
chr19	14444555	14454723	TCONS_00026773	+
chr7	68689683	68895909	TCONS_l2_00027412+TCONS_l2_00027413+TCONS_00014334+TCONS_l2_00027414+TCONS_l2_00026680	−
chr4	189698770	189739775	TCONS_00008713+TCONS_00007937+TCONS_00008714+TCONS_00009205+TCONS_00008715	−
chr16	66273432	66300927	TCONS_00024703	−
chr11	30763103	30814677	TCONS_00019270	+
chr4	61616304	61655817	TCONS_l2_00021603+TCONS_l2_00020545	+
chr13	53063128	53161225	TPTE2P3	+
chr10	33819696	33826083	TCONS_00018158	+
chr13	48270851	48279452	TCONS_00021999	−
chr8	72315677	72459892	TCONS_l2_00028559	−
chr7	41004266	41019537	TCONS_00013158	−
chr9	139440652	139447353	TCONS_l2_00029132+TCONS_l2_00029131+TCONS_l2_00029883+TCONS_l2_00029133+TCONS_l2_00029134+TCONS_l2_00029135+TCONS_l2_00029136+TCONS_l2_00029137+TCONS_l2_00029138+TCONS_l2_00029140+TCONS_l2_00029139+TCONS_l2_00029884	+
chr9	37383175	37384431	TCONS_00015807	−
chr1	203343730	203369257	TCONS_00002218+TCONS_00002219+TCONS_00001247+TCONS_00001248	+
chr19	44395956	44405955	LOC100505715	−
chr3	176018236	176037166	TCONS_00006710	−
chr6	98158829	98165073	TCONS_00011529	−
chr12	8177708	8179309	TCONS_00020294	+
chr5	121165112	121176422	TCONS_00010076	+
chr13	22182229	22183804	TCONS_00021715	+
chr2	47090678	47102328	TCONS_00003670	+
chr8	75522030	75541107	TCONS_00015452+TCONS_00015040+TCONS_00015041	−
chr8	131535158	131541575	TCONS_00015176	−
chr2	44475176	44476732	TCONS_00004236	−
chr1	59521671	59553867	TCONS_00000527+TCONS_00000528	−
chr5	158758526	158789842	LOC285626	+
chr17	16886104	16886565	TCONS_00025320	+
chr19	24097689	24104295	TCONS_l2_00012430	+
chr6	9225547	9238560	TCONS_00011691	+
chr5	131520569	131523063	TCONS_00010092	+
chr4	1546985	1555291	TCONS_00007953+TCONS_00007954+TCONS_00008737+TCONS_00007955+TCONS_00008738+TCONS_00008739+TCONS_00008740	+
chr20	54129021	54201563	TCONS_l2_00016674	−

chr18	51105996	51108994	TCONS_00026332	+
chr2	231555636	231565244	LOC151475	−
chr9	43140537	43145484	LOC642929	−
chr10	10100685	10105465	TCONS_00017702	+
chr1	224051082	224051801	TCONS_l2_00001799	−
chr3	86954827	86959897	TCONS_00006553	−
chr15	89943395	89981066	TCONS_00024003+TCONS_00023502	+
chr11	127957141	128053879	TCONS_00019800	−
chr10	95469445	95515325	TCONS_l2_00003714+TCONS_l2_00003715	−
chr9	85020103	85021154	TCONS_00015700	+
chr6	15995175	16000028	TCONS_00011271	+
chr2	129338065	129339150	TCONS_00003400	−
chr11	69159846	69167800	TCONS_00019684	−
chr5	50265051	50265902	TCONS_l2_00023390+TCONS_l2_00022305	+
chr4	153099994	153101432	TCONS_00008625	−
chr6	15674470	15682336	TCONS_00012089	−
chr15	67276460	67280258	TCONS_00023705+TCONS_00024143+TCONS_00024144 +TCONS_00023706	−
chrX	55681154	55683398	TCONS_l2_00030817	−
chr10	74857802	74861055	TCONS_00017996	−
chr14	51800111	51832275	LINC00640	+
chr13	38603792	38625695	TCONS_l2_00006799	+
chr2	114579400	114647368	TCONS_l2_00014842+TCONS_l2_00014843+TCONS_ l2_00015938+TCONS_l2_00015939+TCONS_ l2_00015940+TCONS_l2_00014844+TCONS_ l2_00014845+TCONS_l2_00014846+TCONS_ l2_00014847+TCONS_l2_00014848+TCONS_l2_00014849	−
chr20	54871023	54874534	TCONS_00028070+TCONS_00028449	−
chr5	53939760	53942025	TCONS_00009962	+
chr5	60475557	60479613	TCONS_l2_00022336	+
chr1	5058062	5058786	TCONS_00001382	−
chr5	131514927	131516821	TCONS_00009785	−
chr2	96477742	96479705	TCONS_00004852	+
chr6	35118455	35132003	TCONS_00011296	+
chr6	3051335	3053998	TCONS_00011430+TCONS_00011431+TCONS_00011432	−
chr14	101292445	101327360	MEG3	+
chr1	181143620	181151343	TCONS_00000059+TCONS_00000344	+
chr21	44251150	44257805	TCONS_00028942+TCONS_00029300	−
chr11	72859381	72892257	TCONS_00019382	+
chr14	56859650	56893148	TCONS_00022516	+
chr13	23474438	23493355	TCONS_l2_00006731+TCONS_l2_00007364	+
chr6	144550948	144605936	TCONS_l2_00024960	−
chr9	83484673	83486210	TCONS_00015839	−
chr21	36096105	36109479	TCONS_00028746	+
chr16	8976217	8979196	TCONS_l2_00009583+TCONS_l2_00009584	+
chr18	61880318	61927290	LOC400654	−
chr7	90917076	90919075	TCONS_00013525	+

chr9	34663495	34666315	TCONS_l2_00029247+TCONS_l2_00029248+TCONS_l2_00029917+TCONS_l2_00029916+TCONS_l2_00029249+TCONS_l2_00029250	−
chr9	67914829	67919763	TCONS_00015685	+
chr12	44054685	44055207	TCONS_l2_00005603	+
chr11	91596258	91605626	TCONS_00019440	+
chr4	131802182	131806796	TCONS_00007829	−
chr10	28288717	28338544	TCONS_l2_00003921	+
chr17	37186159	37209458	LRRC37A11P	+
chr13	19520899	19532561	TCONS_l2_00007049+TCONS_l2_00007481	−
chr12	4318463	4320836	TCONS_00020680	−
chr13	62895438	62896531	TCONS_00021560	+
chr5	23980843	24178799	TCONS_l2_00022231+TCONS_l2_00022232	+
chr20	22541192	22559280	LINC00261	−
chr3	44462619	44465499	TCONS_00005769+TCONS_00006500	−
chr17	13202466	13210077	TCONS_l2_00011093+TCONS_l2_00011616+TCONS_l2_00011094	−
chr10	2047667	2056542	LINC00700	−
chr6	88717726	88729647	TCONS_00011881	+
chr8	58055272	58063293	TCONS_00014707	+
chr14	80410178	80412524	TCONS_00022564	+
chr20	44797043	44799876	TCONS_00028055	−
chr10	6162514	6183674	TCONS_l2_00003384+TCONS_l2_00004096+TCONS_l2_00004097+TCONS_l2_00003385	−
chr2	154168183	154189764	TCONS_00003907	+
chr8	124460171	124478254	TCONS_00015340	+
chr10	48116504	48118029	TCONS_00018199	+
chr15	25729978	25823938	TCONS_00023273+TCONS_00023591	−
chr9	118650544	118687377	LINC00474	−
chr11	57386014	57388699	TCONS_00019303	+
chr16	85613416	85617197	TCONS_00024752+TCONS_00024753	−
chr7	149927175	149934873	TCONS_00014005+TCONS_00014006	−
chr9	137110941	137112411	TCONS_00016206	+
chr21	9698812	9699252	TCONS_00029192	+
chr2	107318213	107417250	TCONS_00003813+TCONS_00004869+TCONS_00003814	+
chr10	22737507	22743141	TCONS_00018134	+
chr2	68023186	68052694	TCONS_00003317	−
chr6	114225551	114242806	FLJ34503	+
chr16	20365462	20367740	TCONS_00024598	−
chr11	38703057	38708253	TCONS_00019278	+
chr15	89339304	89342033	TCONS_00023775	−
chr7	45431940	45436006	TCONS_00013434	+
chr4	40318502	40332436	TCONS_l2_00021582+TCONS_l2_00020497+TCONS_l2_00020498	+
chr7	122709303	122743643	TCONS_00013247	−
chr15	53229050	53229852	TCONS_l2_00009118	−
chr11	122282463	122289181	TCONS_00019510	+
chr7	153119376	153120601	TCONS_00013636	+

chr3	181782596	181787995	TCONS_00006718	−
chr1	184746600	184748797	TCONS_00001220	+
chr11	15725576	15726906	TCONS_00019243	+
chr18	11619234	11640034	TCONS_l2_00011960+TCONS_l2_00011961+TCONS_l2_00011962+TCONS_l2_00011963+TCONS_l2_00011964+TCONS_l2_00011965+TCONS_l2_00011966+TCONS_l2_00011967+TCONS_l2_00011968+TCONS_l2_00011969+TCONS_l2_00011970+TCONS_l2_00011971+TCONS_l2_00011972+TCONS_l2_00011973+TCONS_l2_00011974+TCONS_l2_00011975+TCONS_l2_00011976	−
chr9	42363048	42367092	TCONS_00015811	−
chr14	25571045	25614751	TCONS_00022685+TCONS_00022686+TCONS_00023084+TCONS_00023085+TCONS_00022687	−
chr1	87099959	87121059	CLCA3P	+
chr8	27029284	27068608	TCONS_00014655+TCONS_00015253	+
chr8	11197146	11225961	TDH	+
chr8	128128656	128241550	TCONS_00015498+TCONS_00015168+TCONS_00015169+TCONS_00015170+TCONS_00014531+TCONS_00015171	−
chr16	32262073	32268534	TCONS_00024630	−
chr7	132443730	132445392	TCONS_00013080	+
chr7	57639696	57672355	TCONS_l2_00025904	+
chr11	97808029	97813764	TCONS_00020122	−
chr5	79232402	79263449	TCONS_00009433	+
chr17	42098476	42101170	TCONS_l2_00011262	−
chr1	115813553	115819102	TCONS_00001634	−
chr14	88039860	88192324	TCONS_00022824	−
chr8	687587	1087777	ERICH1-AS1	+
chr6	11810835	11811481	TCONS_00011268	+
chr14	56512566	56515402	TCONS_00022514	+
chr1	218066242	218094146	LINC00210	+
chr10	134830882	134831148	TCONS_00018397	+
chr15	91221600	91256537	TCONS_00023781+TCONS_00023782	−
chrX	118011939	118012717	TCONS_l2_00030581	−
chr12	117147983	117150681	TCONS_00020237	+
chr2	217927047	217931700	TCONS_00004577+TCONS_00004578	−
chr3	44465602	44470995	TCONS_00006821+TCONS_00006820+TCONS_00006822+TCONS_00006823+TCONS_00006004	+
chr1	23875630	23877406	TCONS_00000482	−
chr1	1317609	1318166	TCONS_00000801+TCONS_00000802	+
chr4	70265427	70267738	TCONS_l2_00020564	+
chr22	45019994	45020914	TCONS_00029834	+
chr1	95143554	95155440	TCONS_00001038+TCONS_00002094+TCONS_00002095	+
chr4	55702327	55711568	TCONS_00007760	−
chr12	28723548	28734479	TCONS_00020365+TCONS_00021089+TCONS_00021090	+
chr12	91069065	91078196	TCONS_00020514	+
chr6	29718584	29718925	IFITM4P	−
chr22	51174259	51176567	TCONS_00029355	−
chr14	81636146	81638207	TCONS_00022566	+

chr1	56701944	56719436	TCONS_00001501+TCONS_00001502	−
chr2	22156119	22753977	TCONS_l2_00014471+TCONS_l2_00015742	−
chr1	111823146	111828730	CHIAP2	+
chr1	201084904	201096312	TCONS_00000713	−
chr7	41975695	41976543	TCONS_00013427	+
chr9	109984579	110017878	TCONS_00016664+TCONS_00016116+TCONS_00016117	+
chr6	14661560	14665451	TCONS_00011704+TCONS_00011705	+
chr17	896643	897577	TCONS_00025267	+
chr1	83439565	83451891	TCONS_l2_00002537+TCONS_l2_00002538+TCONS_ l2_00001305	−
chr6	153004435	153010302	TCONS_00011198	+
chr2	74212259	74213470	TCONS_00002934	+
chr8	102064282	102088479	FLJ42969	+
chr15	30121815	30261051	TCONS_l2_00008966	−
chr1	211431656	211432110	TCONS_00001800	−
chr5	25083407	25162815	TCONS_00009372	+
chr4	189911869	189923229	TCONS_00007938	−
chr9	68397878	68401759	TCONS_00015825	−
chr6	95256290	95317650	TCONS_00012226	−
chr2	108845324	108848661	TCONS_00004374+TCONS_00004375	−
chr1	205425186	205438152	TCONS_00000064	+
chr2	105275771	105283314	TCONS_l2_00013868+TCONS_l2_00015424+TCONS_ l2_00015425	+
chr10	101286107	101290934	TCONS_00017748	−
chr3	95031	96029	TCONS_00005722	−
chr2	156082423	156088030	TCONS_00004477	−
chr12	19755760	19804413	TCONS_00020351	+
chr12	126588449	126591880	TCONS_00020626	+
chr5	79287167	79315919	TCONS_l2_00022386+TCONS_l2_00022387	+
chr4	118281779	118294008	TCONS_00008204+TCONS_00007596+TCONS_000 07597+TCONS_00008873+TCONS_00007598+TCO NS_00007599	+
chr21	26887785	26890650	TCONS_00028903	−
chr11	87562336	87670780	TCONS_00019425+TCONS_00019426+TCONS_00019427	+
chr17	25985468	25995444	TCONS_l2_00011163+TCONS_l2_00011164	−
chr21	38580804	38594037	DSCR9	+
chr10	43916037	43918820	TCONS_00018175+TCONS_00017819	+
chr14	53418583	53419221	TCONS_00022748	−
chr21	26467335	26475983	TCONS_00028814+TCONS_00028815	+
chr9	11822678	11834988	TCONS_00016270+TCONS_00016271+TCONS_00016272	−
chr12	20257338	20262820	TCONS_00020726+TCONS_00021293+TCONS_00020727	−
chr3	7994492	8057994	TCONS_l2_00020069+TCONS_l2_00019134	−
chr15	39764938	39785033	TCONS_00023919+TCONS_00023362+TCONS_00023361 +TCONS_00023360	+
chr13	39141144	39141923	TCONS_00021972+TCONS_00021971	−
chr10	120058544	120060036	TCONS_00018353	+
chr16	85378887	85387253	TCONS_00024750	−
chr4	80508539	80521036	TCONS_l2_00021187	−

chr18	12200778	12224709	C18orf61	+
chr4	69650341	69652061	TCONS_l2_00020559	+
chr2	9246672	9250451	TCONS_00004136	−
chr3	191747669	191754346	TCONS_00005692+TCONS_00005693+TCONS_00005694	+
chr7	158383260	158383964	TCONS_00013656+TCONS_00013107	+
chr11	34714000	34723562	TCONS_00019273	+
chr20	38632912	38642086	TCONS_l2_00016252+TCONS_l2_00016253+TCONS_l2_00016254+TCONS_l2_00016769	+
chr4	184386042	184393909	TCONS_00008683	−
chr10	64883695	64885314	TCONS_00018939+TCONS_00017988+TCONS_00018940	−
chr2	61971122	61992046	TCONS_00004271	−
chr1	157606100	157609826	TCONS_l2_00001567	−
chr6	10423373	10426409	TCONS_00011446	−
chr16	58783542	59233253	TCONS_l2_00010415+TCONS_l2_00009773+TCONS_l2_00009774+TCONS_l2_00009775+TCONS_l2_00009776	+
chr16	54880945	54882400	TCONS_00025013+TCONS_00024660	−
chr2	139667504	139674941	TCONS_00004462	−
chr16	11501179	11511667	TCONS_00024581	−
chr5	61173936	61181956	TCONS_00009409	+
chr1	120906034	120914842	HIST2H2BA	+
chr1	9474622	9489003	TCONS_00001385+TCONS_00002288	−
chr11	67653917	67661561	TCONS_00019351+TCONS_00019099+TCONS_00019352+TCONS_00019353	+
chr3	142850116	142850733	TCONS_00005603	+
chr19	28340562	28380105	TCONS_00027281	−
chr8	82066546	82075134	TCONS_00014780+TCONS_00014781	+
chr12	68099583	68100337	TCONS_00020826	−
chr15	101709636	101711509	TCONS_00023840	−
chr15	21122021	22145802	NF1P2	−
chr12	47432453	47436922	TCONS_l2_00005629	+
chr5	144769379	144775848	TCONS_00010520	−
chr4	32352660	32354842	TCONS_00007505	+
chr11	128081358	128083924	TCONS_00019531	+
chr2	81928462	81929613	TCONS_00003748	+
chr12	80799677	80815204	TCONS_l2_00005792	+
chr4	75850938	75857875	TCONS_00008503	−
chr16	68601107	68610173	TCONS_00024872+TCONS_00024873	+
chr22	17075907	17082026	TCONS_l2_00018301	−
chr13	59395728	59397386	TCONS_00022033	−
chr12	113847692	113851849	TCONS_00020565	+
chr7	13770981	13783205	TCONS_00013350	+
chr9	32840596	32841760	TCONS_l2_00029911	−
chr21	43136596	43137742	LINC00112	+
chr12	54472623	54475607	LOC100240735	−
chr6	72124149	72130448	LINC00472	−
chrY	9573895	9596085	TTTY2B	+
chr12	5131731	5135165	TCONS_l2_00005433	+
chr2	158733219	158734099	TCONS_00003916	+

chr8	82271922	82306229	TCONS_00014782	+
chr3	108462080	108464336	TCONS_00006604+TCONS_00007229	−
chr11	86666663	86711989	LOC100506368	+
chr2	48133221	48135298	TCONS_00002890+TCONS_00003671+TCONS_00002891	+
chr21	46409779	46414001	LINC00163	−
chr2	19911476	19917337	TCONS_00002843	+
chr3	81255529	81258035	TCONS_00006108	+
chr4	9036244	9154859	TCONS_l2_00021049+TCONS_l2_00021050+TCONS_ l2_00021051+TCONS_l2_00021052+TCONS_ l2_00021053+TCONS_l2_00021054+TCONS_ l2_00021055+TCONS_l2_00021056+TCONS_l2_00021057	−
chr1	179798744	179805259	TCONS_00000693	−
chr10	106355799	106357399	TCONS_00018321	+
chr19	9897281	9905142	TCONS_l2_00012843+TCONS_l2_00012844+TCONS_ l2_00012845+TCONS_l2_00012846	−
chr4	39135533	39137228	TCONS_00007517	+
chr17	72601129	72603313	TCONS_00025487+TCONS_00025488+TCONS_00025489	+
chr10	6821560	6884868	LINC00707	+
chr22	35515817	35627049	TCONS_l2_00018401+TCONS_l2_00018032+TCONS_ l2_00018033	−
chr14	26851403	26866526	TCONS_00022688+TCONS_00023086	−
chr16	90244125	90289178	TCONS_l2_00010440+TCONS_l2_00009912+TCONS_ l2_00009913+TCONS_l2_00009914+TCONS_l2_00009915	+
chr2	71115001	71115478	TCONS_00002933	+
chr20	47099591	47107082	TCONS_00028561+TCONS_00028202+TCONS_00028204 +TCONS_00028203+TCONS_00027943	+
chr9	23850848	23947021	TCONS_00015931+TCONS_00016543	+
chr10	83552592	83577279	TCONS_00018557	−
chr7	88339945	88386039	TCONS_l2_00027164	+
chr20	16555756	16559931	TCONS_00028112	+
chr2	101286354	101292500	TCONS_00003340	−
chr12	58287870	58290670	TCONS_00020446	+
chr1	120140325	120141914	LINC00622	−
chr13	19982182	19983669	TCONS_00021609	−
chr10	46780490	46790500	TCONS_l2_00004170	−
chr9	93825576	93837414	LOC100129316	−
chr17	7771139	7777031	TCONS_00025133	−
chr19	23708438	23748325	TCONS_00027247+TCONS_00027248+TCONS_00027718	−
chr1	83368866	83632498	TCONS_00000242+TCONS_00000243	+
chr10	101286705	101288223	TCONS_00017714	+
chr13	24481423	24523454	ANKRD20A19P	−
chr19	51320937	51322134	MGC45922	+
chr5	9621489	9623718	TCONS_00009601	−
chr11	64195633	64200142	TCONS_00019144	+
chr13	45373640	45383766	LINC00330	−
chr6	140299286	140312236	TCONS_00012301	−
chr16	30597224	30604442	TCONS_00024362	+
chr9	109378339	109464943	TCONS_00015584+TCONS_00015732+TCONS_00016663 +TCONS_00016114+TCONS_00016115+TCONS_00015733	+

chr14	29530333	29533749	TCONS_00022695+TCONS_00022696	−
chr11	69300569	69308547	TCONS_00019370+TCONS_00019369	+
chr2	10170631	10178720	TCONS_00003566	+
chr10	9832482	9920039	TCONS_00018429+TCONS_00018880+TCONS_00018430+TCONS_00018431	−
chr3	154958732	154976547	TCONS_00007012+TCONS_00007013+TCONS_00005436+TCONS_00005631+TCONS_00006282+TCONS_00006283	+
chr6	106938972	106950059	TCONS_00012765+TCONS_00012240	−
chr4	57488955	57492377	TCONS_00007535	+
chr7	19853308	19871039	TCONS_00013136	−
chr10	65633370	65647064	TCONS_00017737	−
chr10	66451214	66452672	TCONS_00018219	+
chr12	10089185	10096037	TCONS_00020204+TCONS_00021287+TCONS_00021288+TCONS_00020714	−
chr2	243030844	243102469	LOC728323	+
chr12	50323159	50324107	TCONS_00021310	−
chr6	1128390	1132009	TCONS_00012038	−
chr13	107028911	107030142	LINC00460	+
chr7	63168770	63176135	TCONS_00013466+TCONS_00013467	+
chr4	187981253	187982084	TCONS_00007691	+
chr19	9903552	9913112	TCONS_00027473+TCONS_00027474	+
chr4	139346677	139358023	TCONS_00007640	+
chr8	33613300	33654115	TCONS_00014669	+
chr12	33470704	33475320	TCONS_00020374	+
chr5	73665201	73832801	TCONS_00009702	−
chr15	55802948	55806712	TCONS_00023685	−
chr6	68665336	68708615	TCONS_00011851	+
chr13	112513482	112514507	TCONS_00021907	+
chr18	6728833	6729916	TCONS_00026397	−
chr11	65254018	65256453	TCONS_00019665	−
chr1	21744782	21746509	TCONS_00000861	+
chr7	56943078	56949839	LOC100130849	−
chr3	97819578	97823722	TCONS_l2_00018703	+
chr2	105950391	105953445	TCONS_00004365+TCONS_00004366	−
chr9	87027028	87112569	TCONS_00016032	+
chr22	17126460	17127459	TCONS_l2_00017859	−
chr9	139534285	139541598	TCONS_l2_00029645+TCONS_l2_00029647	−
chr17	21383658	21405497	TCONS_00025591+TCONS_00025592	−
chr15	100346547	100379411	TCONS_l2_00008876+TCONS_l2_00008877+TCONS_l2_00008878+TCONS_l2_00008879+TCONS_l2_00009476+TCONS_00023549	+
chr12	5336170	5353372	TCONS_l2_00006067+TCONS_l2_00006068+TCONS_l2_00006621+TCONS_l2_00006069+TCONS_l2_00006070	−
chr1	47223947	47233300	TCONS_00000192	+
chr7	67582818	67584196	TCONS_00013019	+
chr7	30586782	30590397	TCONS_00012989	+
chr4	80319090	80320316	TCONS_00008129	+

chr15	29963428	29973149	TCONS_l2_00008512+TCONS_l2_00008513+TCONS_l2_00009421+TCONS_l2_00008514+TCONS_l2_00008515	+
chr8	39172182	39260375	ADAM5	+
chr12	133485301	133490880	TCONS_l2_00006617+TCONS_l2_00006033	+
chr16	11465883	11484301	TCONS_l2_00009997+TCONS_l2_00009998+TCONS_l2_00009999+TCONS_l2_00010000+TCONS_l2_00010001	−
chr18	53750587	53804767	LOC100505474	−
chr17	18330175	18333940	TCONS_l2_00011527+TCONS_l2_00010594	+
chr1	156681159	156682982	TCONS_00002495+TCONS_00000647	−
chrX	25896456	25911661	TCONS_00016973	+
chr1	242692666	242693831	TCONS_00001895	−
chr8	58112470	58135373	TCONS_00015015	−
chr1	168732603	168743728	TCONS_00000674	−
chr11	109817287	109843963	TCONS_00019461	+
chr10	48324788	48332197	TCONS_00017974	−
chr5	31175318	31176072	TCONS_00010294	−
chr13	112834895	112835527	TCONS_00022119	−
chr21	46756622	46758580	TCONS_00028860	+
chr2	85190235	85194331	TCONS_00003752	+
chr2	207504797	207506006	TCONS_00003083	+
chr20	57329406	57331891	TCONS_l2_00016808	+
chr18	12288322	12291290	TCONS_00026289	+
chr3	5882629	6407572	TCONS_l2_00018428+TCONS_l2_00018429+TCONS_l2_00018430+TCONS_l2_00019753+TCONS_l2_00018431+TCONS_l2_00019754	+
chr11	14934959	14969520	TCONS_00019241	+
chr4	188891433	188892446	TCONS_l2_00021481	−
chr13	111748185	111755444	TCONS_l2_00007472+TCONS_l2_00007012	+
chr6	169322148	169327972	TCONS_00012366	−
chr3	153149645	153161320	TCONS_00005629	+
chr7	95289645	95311461	TCONS_00013539+TCONS_00014176+TCONS_00013540	+
chr6	96367270	96371958	TCONS_00012230	−
chr12	55001971	55004246	GLYCAM1	−
chr21	10206072	10208330	TCONS_00028963	+
chr22	34587560	34614763	TCONS_00029582+TCONS_00029583	+
chr14	95786503	95800794	TCONS_00022612+TCONS_00022613+TCONS_00023044+TCONS_00023045+TCONS_00023046	+
chr8	33716885	33717718	TCONS_l2_00028151	−
chrY	8551411	8551919	TTTY18	−
chr8	8090820	8102380	TCONS_l2_00028086+TCONS_l2_00028087+TCONS_l2_00028088+TCONS_l2_00028089	−
chr12	67834936	67836838	TCONS_00020824	−
chr9	115875176	115882126	FAM225A	+
chr3	170128300	170136403	TCONS_00005657	+
chr10	65929847	65930169	TCONS_l2_00003601	−
chr10	97051412	97052039	TCONS_00018289	+
chr11	64177548	64187687	TCONS_00019172+TCONS_00019658	−
chr7	124791380	124793665	TCONS_00013251	−
chr20	24160867	24164530	TCONS_00028349	−

chr19	35695955	35706795	TCONS_00027001+TCONS_00027002+TCONS_00027003+TCONS_00027004	+
chr1	106132315	106161581	TCONS_00001618+TCONS_00000596+TCONS_00000093	−
chr1	94792784	94808287	TCONS_00001034+TCONS_00001035+TCONS_00000248+TCONS_00001036	+
chr1	247348512	247405125	TCONS_l2_00001907+TCONS_l2_00001908+TCONS_l2_00001909+TCONS_l2_00001910+TCONS_l2_00001911+TCONS_l2_00001912+TCONS_l2_00002828	−
chr15	31507884	31511740	TCONS_00023341+TCONS_00023909+TCONS_00023910	+
chr20	17868007	17868804	TCONS_00028010	−
chr4	3064973	3076241	HTT-AS1	−
chr3	74665436	74671785	TCONS_00006096	+
chr13	30726023	30733758	TCONS_00021618	−
chr17	72560057	72563679	TCONS_l2_00010961	+
chr12	127544671	127566513	TCONS_00020640	+
chr13	53631291	53650848	TCONS_00021794	+
chrX	70430035	70948962	BCYRN1	−
chr10	30252179	30257684	TCONS_00018705	+
chrY	6339072	6341671	TTTY8B	−
chr2	23240997	23421927	TCONS_00003230+TCONS_00004179+TCONS_00003231+TCONS_00003232	−
chrX	47566590	47596027	CXXC1P1	+
chr20	12224543	12224944	TCONS_00028005	−
chr7	139782542	139783219	TCONS_00013610	+
chr3	75263634	75264910	TCONS_l2_00018657+TCONS_l2_00018656	+
chrX	100918589	100926042	TCONS_00017217+TCONS_00017435	+
chr8	12294522	12424354	LOC100506990	+
chr6	138144807	138189370	LOC100130476	−
chr19	58916679	58919843	TCONS_00027438+TCONS_00027828+TCONS_00027829+TCONS_00027439+TCONS_00027830+TCONS_00027440	−
chr7	66434531	66440267	TCONS_00013850+TCONS_00013851+TCONS_00013852	−
chr22	21636714	21652015	POM121L8P	+
chr2	154028501	154058240	TCONS_00003422	−
chr12	130555901	130557218	TCONS_00020654	+
chr6	84692248	84717042	TCONS_00012538+TCONS_00012539+TCONS_00011335+TCONS_00012540+TCONS_00012541	+
chr15	83511246	83514392	TCONS_00023268	+
chr17	16936688	16944627	TCONS_00025579	−
chr2	146358475	146360991	TCONS_00003416	−
chr16	49151037	49154982	TCONS_00024385	+
chr3	181328151	181459005	SOX2-OT	+
chr1	55683534	55699850	TCONS_l2_00002055	+
chr15	93583948	93584641	TCONS_00023514	+
chr20	43592440	43595099	STK4-AS1	−
chr2	160761405	160763529	TCONS_00003921	+
chr12	132302149	132303828	TCONS_00021009	−
chr18	71678883	71682149	TCONS_00026367	+
chr1	228382416	228391302	TCONS_l2_00000881	+

chr7	149244153	149250817	TCONS_l2_00026263	+
chr3	34566310	34585372	TCONS_00005755	−
chr16	79539486	79550170	TCONS_00024474	+
chr22	46043024	46045536	TCONS_00029835	+
chr1	243169147	243170770	TCONS_00000772	−
chr4	59390680	59431369	TCONS_00007763+TCONS_00009060+TCONS_00008493+TCONS_00007764	−
chr3	195362629	195366726	TCONS_00006375+TCONS_00007072+TCONS_00006376	+
chr18	29065357	29076959	TCONS_00026475	−
chr7	22689207	22705809	TCONS_l2_00026419+TCONS_l2_00027291+TCONS_l2_00026420	−
chr5	180777137	180792134	TCONS_00009572	+
chr14	98098984	98152995	LOC100129345	−
chr1	244210526	244212010	TCONS_00001898	−
chr20	43808589	43821571	TCONS_00028171+TCONS_00028547+TCONS_00028548+TCONS_00028172+TCONS_00028173+TCONS_00028174	+
chr5	125998813	126009286	TCONS_l2_00023084	−
chr2	88986204	88991016	TCONS_00004327+TCONS_00005195+TCONS_00005196	−
chr8	89578146	89611125	TCONS_00015068+TCONS_00015069+TCONS_00015070	−
chr9	92142766	92168617	TCONS_00016057	+
chr9	42858152	42893137	AQP7P3	+
chr3	150180867	150184187	TCONS_00006268	+
chr2	118421687	118424589	TCONS_00003823	+
chr16	85995141	85995657	TCONS_00024755	−
chr2	140123735	140136717	TCONS_00003012	+
chr10	102133333	102148111	LINC00263	+
chr11	76154345	76155704	TCONS_00019712	−
chr21	23872924	23892377	TCONS_00028886	−
chr7	123517895	123520268	TCONS_00013944	−
chr5	7299487	7306827	LOC442132	−
chr14	87613822	87614350	TCONS_00022581	+
chr21	37952487	37953282	TCONS_00029012	+
chr1	115724043	115765741	TCONS_00001113	+
chr14	38205181	38208450	TCONS_00022478	+
chr4	149862093	149863530	TCONS_00007870	−
chr9	33500946	33502751	TCONS_00015659	+
chr4	27969345	27986685	TCONS_00007737	−
chr17	40180921	40250469	TCONS_l2_00011220+TCONS_l2_00011221+TCONS_l2_00011222+TCONS_l2_00011223+TCONS_l2_00011224+TCONS_l2_00011225	−
chr1	19852670	19858268	TCONS_00000856	+
chr3	139397464	139401406	TCONS_00005837	−
chr15	91446090	91446964	TCONS_00024005	+
chr20	61265124	61267214	TCONS_00027981	+
chrX	13268791	13269231	TCONS_00017292	−
chr8	11500333	11506826	TCONS_00014617	+
chr8	94896996	94910815	TCONS_00015486	−
chr5	123828468	123872041	TCONS_00009773	−

chr21	45225639	45232448	LOC284837	−
chr12	8941118	8948420	TCONS_00020306	+
chr2	147637477	147659500	TCONS_00003028	+
chr10	54698381	54703837	TCONS_00018516	−
chr21	26310745	26365129	TCONS_00028893+TCONS_00028894+TCONS_00028895	−
chr4	42240722	42253256	TCONS_00008058	+
chr6	156693389	156695660	TCONS_00012340	−
chr5	180708376	180722552	TCONS_l2_00023622+TCONS_l2_00022707+TCONS_ l2_00022708+TCONS_l2_00022709+TCONS_ l2_00022710+TCONS_l2_00022711+TCONS_l2_00022712	+
chr21	44808523	44809763	TCONS_00029159	−
chr9	43027663	43033312	FAM95B1	−
chr12	89404903	89413469	LOC728084	−
chr1	2496461	2516050	TCONS_l2_00000050+TCONS_l2_00001960+TCONS_ l2_00000051+TCONS_l2_00000052+TCONS_ l2_00000053+TCONS_l2_00000054+TCONS_ l2_00000055+TCONS_l2_00000056+TCONS_l2_00000057	+
chrX	135991554	136104280	TCONS_l2_00030751+TCONS_l2_00030752+TCONS_ l2_00030370+TCONS_l2_00030371+TCONS_ l2_00030372+TCONS_l2_00030753+TCONS_ l2_00030754+TCONS_l2_00030373	+
chr17	66116838	66140971	TCONS_l2_00011427+TCONS_l2_00011429+TCONS_ l2_00011430	−
chr6	131018457	131018923	TCONS_00011369	+
chr13	80585259	80733471	TCONS_00021856+TCONS_00021857	+
chr6	140092210	140181608	LOC100132735	+
chr1	53798053	53812604	TCONS_00001496+TCONS_00002383	−
chr3	147810962	147845020	TCONS_00005613	+
chr7	67997952	67998711	TCONS_00013857	−
chr1	222626082	222628047	TCONS_00000741	−
chr15	71373658	71385917	TCONS_00023452	+
chr6	14230006	14231801	TCONS_00011455+TCONS_00012085	−
chr8	94507989	94508523	TCONS_00015324	+
chr10	99173633	99179327	TCONS_00018787	+
chr3	157734005	157806786	TCONS_00005861+TCONS_00005862	−
chr5	71957506	71986512	TCONS_00010371+TCONS_00010372	−
chr21	44068413	44071354	TCONS_00028786	−
chrX	15621004	15639607	TCONS_00016970	+
chr7	66800296	66821094	TCONS_00013494+TCONS_00014159+TCONS_00012881 +TCONS_00014160	+
chrX	39164210	39186616	LOC286442	−
chr11	119703727	119737875	TCONS_00019499	+
chr1	158164426	158173692	TCONS_l2_00001572	−
chr2	155328062	155345430	TCONS_00004474+TCONS_00005256	−
chr7	62856718	62859419	LOC100287834	−
chr11	74691592	74695088	TCONS_00019151	+
chr1	193648024	193696313	TCONS_00000362	+
chr9	99967253	99979711	TCONS_l2_00029513+TCONS_l2_00029514	−
chrX	22277914	23311263	LOC100873065	−

chr10	54056608	54073888	PRKG1-AS1	−
chr4	14392063	14395616	TCONS_00007484	+
chrX	75107994	75109321	TCONS_l2_00030716	+
chr6	165206864	165241153	TCONS_00011240+TCONS_00012356	−
chr2	495731	496628	TCONS_00004655+TCONS_00003530+TCONS_00003531	+
chr4	152877106	152890531	TCONS_00008905+TCONS_00007408+TCONS_00007409	+
chr10	120116475	120124826	TCONS_l2_00003280+TCONS_l2_00003281+TCONS_l2_00004042+TCONS_l2_00004043+TCONS_l2_00004044+TCONS_l2_00003282	+
chr5	45035301	45098749	TCONS_00009395	+
chrX	44600286	44601039	TCONS_l2_00030186	+
chr6	113064917	113066036	TCONS_00011924	+
chr2	242989846	243026749	TCONS_00003524+TCONS_00005375+TCONS_00005376+TCONS_00005379+TCONS_00005378+TCONS_00005377+TCONS_00004652	−
chr13	64159590	64159977	TCONS_00022041	−
chr21	26931716	26936957	TCONS_00028904	−
chrX	25212221	25213284	TCONS_l2_00030465	−
chr18	44337249	44340578	TCONS_00026327+TCONS_00026616	+
chr14	20988477	20990339	TCONS_00022417	+
chrX	48306415	48307284	TCONS_l2_00030196+TCONS_l2_00030197	+
chr10	46764668	48958083	GLUD1P7	+
chr4	170770217	170782640	TCONS_00008642	−
chrX	86979061	86983512	TCONS_00017351	−
chr3	106403340	106436193	TCONS_00006577+TCONS_00007221+TCONS_00006578	−
chr20	38749682	38754998	TCONS_00027931	+
chr15	75517502	75519599	TCONS_00023742	−
chr2	91824709	91847975	LOC654342	−
chr8	141473583	141478663	TCONS_00014872+TCONS_00014873+TCONS_00014874+TCONS_00015375	+
chr2	47452933	47466364	TCONS_00002888+TCONS_00002889	+
chr8	31246487	31249450	TCONS_00014667+TCONS_00015268	+
chr11	65593701	65601267	TCONS_l2_00005064+TCONS_l2_00005065	−
chr21	40110879	40145401	LINC00114	−
chr20	24911303	24912191	TCONS_00028139	+
chr7	91258911	91262062	TCONS_l2_00026747	−
chr14	52656512	52726892	TCONS_00022745	−
chr19	38037568	38042298	TCONS_00027536+TCONS_00027537	+
chr17	32733821	32739611	TCONS_00025353	+
chr9	137397318	137413288	TCONS_00015891	−
chr4	115602947	115611114	TCONS_00008872	+
chr6	135376171	135381688	TCONS_00011378	+
chr10	3793259	3805418	TCONS_00017768	+
chrY	26356114	26360978	GOLGA2P3Y	−
chr20	1166482	1168514	TCONS_00028278	−
chr14	46178990	46326043	TCONS_00022492+TCONS_00022980	+
chr6	4016675	4021449	TCONS_00012055+TCONS_00011436	−
chr22	25957850	25960789	TCONS_00029538	+

chrX	133684054	133694428	LINC00629	+
chr13	69435417	69459457	LINC00550	−
chr22	43782060	43787399	TCONS_00029707	−
chr16	87275181	87296485	TCONS_00024777	−
chr3	88387573	88517304	TCONS_00006909+TCONS_00006910+TCONS_00006125 +TCONS_00006911	+
chr11	76092016	76125663	TCONS_l2_00004657+TCONS_l2_00005328+TCONS_ l2_00004658	+
chr17	5142428	5145921	TCONS_00025536+TCONS_00025537+TCONS_00025538 +TCONS_00025539	−
chr6	150590872	150616896	TCONS_00011986+TCONS_00011987+TCONS_00011988	+
chr18	53804985	53817316	TCONS_00026625+TCONS_00026626	+
chr5	24292127	24322125	TCONS_00009904+TCONS_00009905	+
chr16	63880952	63881349	TCONS_00024434	+
chr10	43474465	43476955	TCONS_00017958	−
chr3	126011603	126013479	TCONS_00006203	+
chr19	29777918	30016659	LOC284395	−
chr1	32814795	32816264	TCONS_00000501	−
chr20	57927250	57932829	TCONS_00028078	−
chr4	76286417	76359659	TCONS_00007783	−
chr18	69187200	69246192	LOC100505776	−
chr2	66554481	66556116	TCONS_00002916+TCONS_00002917	+
chr2	6869300	6910442	LINC00487	−
chr5	175343890	175367290	TCONS_l2_00023251	−
chr2	177494309	177502302	LOC375295	−
chr4	170838912	170897053	LOC100506085	−
chr20	62771381	62774940	TCONS_00028474	−
chr19	23214621	23244067	TCONS_00027234+TCONS_00026806	−
chr2	214101741	214103567	TCONS_00004570	−
chr1	23877741	23878134	TCONS_00001417	−
chr5	148786252	148808237	TCONS_00009538+TCONS_00009261+TCONS_00009539 +TCONS_00010136+TCONS_00009262+TCONS_0000954 0+TCONS_00010137+TCONS_00010138	+
chr4	124411163	124448834	TCONS_00008218+TCONS_00008881+TCONS_00007609 +TCONS_00007610	+
chr13	88079469	88080528	TCONS_00021677	−
chrX	61998718	61999787	TCONS_l2_00030232	+
chr2	43254969	43266686	TCONS_l2_00014535+TCONS_l2_00015776+TCONS_ l2_00014536+TCONS_l2_00014537+TCONS_ l2_00015777+TCONS_l2_00015778	−
chr6	14444896	14448968	TCONS_00012087	−
chr1	95099372	95285834	TCONS_l2_00001386+TCONS_l2_00002568+TCONS_ l2_00001387+TCONS_l2_00002569+TCONS_ l2_00001388+TCONS_l2_00001389+TCONS_ l2_00002570+TCONS_l2_00001390+TCONS_ l2_00002571+TCONS_l2_00001391+TCONS_ l2_00002572+TCONS_l2_00001392+TCONS_ l2_00002573+TCONS_l2_00001393+TCONS_ l2_00001394+TCONS_l2_00002574+TCONS_ l2_00001395+TCONS_l2_00001396	−
chr16	33710592	33725908	TCONS_l2_00010132+TCONS_l2_00010133	−

chr1	69521558	69650686	TCONS_l2_00000323+TCONS_l2_00002068	+
chr14	25715800	25731971	TCONS_00022437+TCONS_00022932+TCONS_00022438 +TCONS_00022439	+
chr8	10436255	10438247	TCONS_00014933	−
chr5	169615274	169626145	TCONS_00010550+TCONS_00011082+TCONS_00009319 +TCONS_00009811	−
chr2	60621992	60628362	TCONS_00004266	−
chr9	70501272	70505069	TCONS_00016338	−
chr4	112725574	112730850	TCONS_00007814	−
chr2	107978738	108023785	TCONS_00002969+TCONS_00002970+TCONS_00002971	+
chr7	29685538	29724754	LOC646762	−
chr5	157681876	157714915	TCONS_00010148	+
chr3	187676548	187694195	TCONS_00006730+TCONS_00005911+TCONS_00005478	−
chr3	33839482	33840004	TCONS_00006467	−
chr9	44053330	44078630	TCONS_l2_00029290+TCONS_l2_00029291+TCONS_ l2_00029292+TCONS_l2_00029293	−
chr2	114285145	114300537	TCONS_l2_00015925+TCONS_l2_00015926+TCONS_ l2_00014822+TCONS_l2_00015927+TCONS_ l2_00014823+TCONS_l2_00015928+TCONS_ l2_00015929+TCONS_l2_00014824+TCONS_ l2_00015930+TCONS_l2_00015931+TCONS_ l2_00014825+TCONS_l2_00014826	−
chr1	90224276	90227625	TCONS_00001023	+
chr4	43982023	43996284	TCONS_l2_00021876+TCONS_l2_00021130	−
chr11	64268325	64272858	TCONS_00019145	+
chr9	27529917	27536721	TCONS_00015940	+
chr6	134749378	134846048	TCONS_l2_00025473+TCONS_l2_00025474+TCONS_ l2_00025476+TCONS_l2_00024920+TCONS_ l2_00025477+TCONS_l2_00024921+TCONS_ l2_00024922+TCONS_l2_00024923+TCONS_ l2_00024924+TCONS_l2_00024925	−
chr5	153476606	153537442	TCONS_00010815+TCONS_00010816	+
chr1	4036189	4069751	TCONS_00000822+TCONS_00001926	+
chr9	89623366	89657041	LOC440173	−
chr3	12918726	12920698	TCONS_00006443	−
chr6	23399154	23404502	TCONS_00011735+TCONS_00011736	+
chr22	39673785	39674133	TCONS_00029597	+
chr12	111376095	111396124	TCONS_00021212+TCONS_00021213+TCONS_00020561	+
chr1	90421225	90422281	TCONS_00000561	−
chr4	99064931	99090451	TCONS_00008169	+
chr8	101464412	101513327	TCONS_00014806+TCONS_00014807+TCONS_00014808 +TCONS_00014809	+
chr1	203507263	203525190	TCONS_00001776	−
chr19	17502024	17502873	TCONS_l2_00012894	−
chr15	23094157	23106207	TCONS_l2_00008416+TCONS_l2_00008417+TCONS_ l2_00008418+TCONS_l2_00008419	+
chr1	224795999	224803922	TCONS_00001841+TCONS_00001842+TCONS_00001843 +TCONS_00000744	−
chr6	138266503	138293659	TCONS_00011383+TCONS_00011382	+
chr16	33365340	33374151	TCONS_l2_00010123+TCONS_l2_00010124+TCONS_ l2_00010125+TCONS_l2_00010126	−

chr1	84920352	84924713	TCONS_00001550	−
chr5	85649621	85664672	TCONS_00009436+TCONS_00009437	+
chr6	26724185	26752237	TCONS_00011753	+
chr4	13487674	13491548	TCONS_00007997	+
chr6	161176480	161178657	TCONS_l2_00025018	−
chr3	36810216	36825658	TCONS_l2_00020104	−
chr12	19804540	19807186	TCONS_00020352+TCONS_00020353	+
chr13	61190522	61193336	TCONS_00022035+TCONS_00022289	−
chr11	3402191	3430378	LOC650368	+
chr7	65841031	65865395	LINC00174	−
chr7	66274980	66309813	GTF2IRD1P1	−
chrX	390760	398438	TCONS_00017111+TCONS_00017112+TCONS_00017113	+
chr1	63715694	63717442	TCONS_00000218	+
chr3	55527858	55539289	TCONS_00005778	−
chr16	85319914	85337002	TCONS_00024305	−
chr1	146644430	146645134	TCONS_00001662	−
chr3	148658605	148673471	TCONS_00007002	+
chrY	22627554	22681114	TTTY10	−
chr10	44404752	44465355	LINC00841	+
chr7	96047846	96051707	TCONS_00013543	+
chr18	24327859	24333995	TCONS_00026468+TCONS_00026469	−
chr8	82526030	82530464	TCONS_l2_00028266	−
chr18	11638006	11677400	TCONS_l2_00011771	+
chr1	225071656	225072471	TCONS_00001844	−
chr12	9499309	9500599	TCONS_00021051	+
chr17	77889984	77900524	TCONS_00025255	−
chr2	1556217	1558473	TCONS_00004663+TCONS_00004664+TCONS_00004665 +TCONS_00004666	+
chr5	72408841	72413568	TCONS_00010376	−
chr9	18419837	18437603	TCONS_00016279	−
chr13	23550010	23551904	TCONS_00021939	−
chr1	95776484	95783970	TCONS_00000583+TCONS_00002410+TCONS_00001601	−
chr12	6387277	6391856	TCONS_00020287	+
chr3	196710761	196713813	TCONS_l2_00020322	−
chr19	56633053	56635753	TCONS_00027116	+
chr5	82026363	82026823	TCONS_00010008	+
chr7	54398390	54417542	TCONS_00013011+TCONS_00013012	+
chr4	123621606	123629294	TCONS_00008880	+
chr5	169735705	169739720	TCONS_00009812	−
chr16	8148851	8149733	TCONS_00024321	+
chr2	45550668	45560508	TCONS_00003664	+
chr1	8062789	8067529	TCONS_00001384+TCONS_00000467	−
chr12	102707354	102709939	TCONS_00020235	+
chr9	97075822	97076871	TCONS_00016076	+
chr2	545805	546667	TCONS_00002791	+
chr14	61108286	61109382	TCONS_00022761	−
chr20	48779907	48782702	TCONS_00028698+TCONS_00028697+TCONS_00028435	−
chr13	24072935	24077013	TCONS_00021530	+

chr10	3916276	3922623	TCONS_00018414	−
chr2	151091406	151113521	TCONS_00003419	−
chr12	111450041	111456213	TCONS_00020921	−
chr18	55471012	55474049	TCONS_00026215	+
chr12	124065613	124068874	TCONS_00020259	−
chr6	140413889	140415734	TCONS_00012805	−
chr17	16438939	16439449	TCONS_00025577	−
chr12	20387841	20417097	TCONS_00020729	−
chr14	71761992	71764863	TCONS_00023013	+
chr2	176881550	176885377	TCONS_l2_00014098	+
chr1	204337558	204338847	LINC00628	−
chr1	79518696	79654137	TCONS_l2_00001295+TCONS_l2_00001296+TCONS_ l2_00001297+TCONS_l2_00001298+TCONS_ l2_00001299+TCONS_l2_00001300	−
chr8	58131648	58145388	LOC100507651	+
chr12	8332805	8353596	FAM66C	+
chr2	19657981	19668722	TCONS_00003221	−
chr10	42827314	42863493	LOC441666	−
chr2	208038358	208087514	TCONS_00003085	+
chr19	44288541	44290302	TCONS_00027758	−
chr21	47456966	47459205	TCONS_00028776	+
chr10	3094708	3109519	TCONS_00018406+TCONS_00018868+TCONS_00018869	−
chr4	114793925	114799347	TCONS_00007401	+
chr9	43949	52582	TCONS_l2_00029157	−
chr12	9799616	9801004	TCONS_00020713	−
chr9	69174214	69181041	LOC440896	−
chr6	8328328	8342688	TCONS_l2_00024019+TCONS_l2_00025084	+
chr12	127630756	127637627	TCONS_00020642+TCONS_00020643+TCONS_00021254 +TCONS_00020645+TCONS_00020644	+
chr5	27406639	27436518	TCONS_00010908+TCONS_00010285+TCONS_000 10909+TCONS_00010286+TCONS_00009624+TCO NS_00010910	−
chr1	4472111	4484744	LOC284661	+
chr2	220360912	220363411	TCONS_l2_00014248+TCONS_l2_00014249+TCONS_ l2_00014250+TCONS_l2_00014251+TCONS_l2_00014252	+
chr15	65376899	65381887	TCONS_00023701	−
chr11	77282102	77287689	TCONS_00020103	−
chr13	24079402	24099623	TCONS_00021730+TCONS_00021731+TCONS_00022143	+
chr2	5689912	5696163	TCONS_00003169	−
chr11	15932520	15933620	TCONS_00019584	−
chr18	67873174	67874456	TCONS_00026355+TCONS_00026356	+
chr1	180528110	180535654	TCONS_00000058+TCONS_00001211+TCONS_00002198	+
chr8	2584690	2662202	TCONS_00014580	+
chr1	108614104	108617141	TCONS_l2_00002122+TCONS_l2_00000472+TCONS_ l2_00002123	+
chr21	17998030	18013444	TCONS_l2_00016979+TCONS_l2_00016980+TCONS_ l2_00016981+TCONS_l2_00017326+TCONS_l2_00017327	+
chr21	29385682	29395528	LINC00314	+
chr21	42948062	42953246	TCONS_00028940	−

chr1	73315920	73320112	TCONS_00000984	+
chr16	21360684	21397254	SNX29P1	+
chr1	25534630	25535360	TCONS_00000168	+
chr18	10405018	10406793	TCONS_00026417	−
chr21	14410487	14490571	ANKRD30BP2	+
chr10	72728284	72728748	TCONS_00018229	+
chr10	106326751	106345352	TCONS_00018320	+
chr4	45011557	45053669	TCONS_l2_00021587+TCONS_l2_00020508	+
chr8	118750472	118753715	TCONS_00015148	−
chr4	171961753	171980311	LOC100506122	+
chr2	47958514	47959167	TCONS_l2_00014564	−
chr3	184429982	184433265	TCONS_00006331	+
chr8	123781577	123782123	TCONS_l2_00027964	+
chr5	68259073	68263672	TCONS_00009414	+
chr7	52232931	52260609	TCONS_00013171	−
chr17	44324301	44338723	TCONS_l2_00010812+TCONS_l2_00010813+TCONS_l2_00010814	+
chr21	19093368	19110972	TCONS_l2_00017159+TCONS_l2_00017160	−
chr5	170171869	170174581	TCONS_00010552+TCONS_00011083+TCONS_00011084	−
chr2	239207351	239209261	TCONS_00003519	−
chr3	131080689	131083966	NUDT16P1	+
chrX	52942044	52944043	TCONS_00017174	+
chr2	175647774	175652656	TCONS_00003441	−
chr5	26322	37720	TCONS_00010226	−
chr14	27267115	27275559	TCONS_00022444	+
chr18	36693454	36739037	TCONS_00026498	−
chr19	7852370	7855898	CLEC4GP1	+
chr2	22600466	22704876	TCONS_00002852	+
chr4	122484906	122486182	TCONS_00008565	−
chr16	52641000	52648675	TCONS_00024416+TCONS_00024417+TCONS_00024418+TCONS_00024846	+
chr11	49013021	49023051	TCONS_l2_00005000	−
chr9	44136621	44145101	TCONS_00016322	−
chr7	36134923	36140254	TCONS_00012877+TCONS_00013412	+
chr3	106018137	106019880	TCONS_00006571	−
chrX	68250822	68256955	TCONS_00017190	+
chr4	49244795	49247226	TCONS_l2_00021132	−
chr8	8422143	8427048	TCONS_00014593	+
chr4	137047064	137060029	TCONS_00007632	+
chr4	120355717	120366886	TCONS_l2_00021266+TCONS_l2_00021267+TCONS_l2_00021268	−
chr17	68147096	68154278	TCONS_00025744	−
chr2	131588551	131594567	TCONS_00002765+TCONS_00003404	−
chr7	112257752	112348135	TCONS_00013565+TCONS_00014195+TCONS_00014196+TCONS_00013058+TCONS_l2_00027194+TCONS_l2_00026160	+
chr10	125710890	125722836	TCONS_00018625+TCONS_00018626	−
chr12	34369805	34372111	TCONS_00020758	−

chr5	117618269	117620455	TCONS_00009258	+
chr20	54717440	54744424	TCONS_00028448	−
chr14	82071691	82089405	TCONS_00022379+TCONS_00022815+TCONS_00023140+TCONS_00022816	−
chr7	155056157	155059857	TCONS_00014018+TCONS_00014019+TCONS_00014446	−
chr1	73020893	73023568	TCONS_00000982	+
chr4	3634756	3635702	TCONS_00007462	+
chr2	32534100	32538803	TCONS_00004743	+
chr22	28112468	28117665	TCONS_00029403	+
chr2	186897872	186981910	TCONS_l2_00016041+TCONS_l2_00016042+TCONS_l2_00016043+TCONS_l2_00015024+TCONS_l2_00015025+TCONS_l2_00015026+TCONS_l2_00015027+TCONS_l2_00015028+TCONS_l2_00015029	−
chr16	61555	64090	DDX11L10	+
chr6	138264216	138266939	TCONS_00011573	−
chr1	207058697	207064884	TCONS_00002568	−
chr4	29048213	29050387	TCONS_00007499	+
chr10	35386934	35415588	TCONS_00017949+TCONS_00017950+TCONS_00018480+TCONS_00017951	−
chr6	58778112	58778756	TCONS_00012197	−
chr5	3337723	3345371	TCONS_00009848+TCONS_00010608	+
chr20	44963603	44968432	TCONS_00028412	−
chr5	133772402	133828210	TCONS_00009513	+
chr12	93397338	93413688	TCONS_00020519+TCONS_00021188	+
chr6	84732774	84734272	TCONS_00011336	+
chr12	103203061	103218177	LINC00485	−
chr10	44409347	44409954	TCONS_00017962	−
chr2	220489781	220491415	TCONS_00003500	−
chr20	57090435	57194948	APCDD1L-AS1	+
chr20	54299642	54320069	TCONS_00028447	−
chr15	74445141	74448531	TCONS_00023739	−
chr2	317912	342118	TCONS_00002786+TCONS_00002787	+
chr6	112221562	112230600	TCONS_00011228+TCONS_00011539	−
chr14	101361107	101373305	MEG8	+
chr9	93682784	93683343	TCONS_00016401	−
chr1	55461470	55462838	TCONS_00000949	+
chr7	22549923	22551684	TCONS_l2_00026417	−
chr5	29143616	29183428	TCONS_00010650+TCONS_00010651+TCONS_00009913+TCONS_00009246+TCONS_00009247+TCONS_00009914+TCONS_00010652+TCONS_00010660+TCONS_00010659+TCONS_00010658+TCONS_00010657+TCONS_00010656+TCONS_00010655+TCONS_00010654+TCONS_00010653	+
chr3	132441186	132593050	NPHP3-AS1	+
chr11	113886127	113887961	TCONS_00019478	+
chr6	110858794	110869129	TCONS_00012247	−
chr14	24169032	24198920	TCONS_00022673+TCONS_00023067+TCONS_00023068+TCONS_00023069+TCONS_00023070+TCONS_00023071+TCONS_00022675+TCONS_00022676	−
chr9	69727436	69820708	TCONS_l2_00028816	+

chr4	150068954	150071438	TCONS_00007872+TCONS_00008616+TCONS_00009137	−
chr11	91572863	91589989	TCONS_00019971+TCONS_00019439+TCONS_00019972	+
chr18	26646813	26665388	TCONS_00026471	−
chr10	5591591	5592847	TCONS_00018419	−
chr2	136843289	136844734	TCONS_00004459	−
chr7	15880922	15881985	TCONS_00013125	−
chr3	167613736	167641797	LOC646168	+
chr17	66793605	66796516	TCONS_00025741	−
chr7	36820613	36822394	TCONS_00012996	+
chr7	6676931	6697399	TCONS_l2_00025633+TCONS_l2_00025635+TCONS_ l2_00025638+TCONS_l2_00025639+TCONS_ l2_00025640+TCONS_l2_00025641+TCONS_ l2_00025642+TCONS_l2_00025643+TCONS_l2_00025644	+
chr2	54662633	54673837	TCONS_00003681	+
chr2	54609065	54610925	TCONS_00003679	+
chr16	33629698	33630084	TCONS_l2_00009699	+
chr13	114053727	114064046	TCONS_l2_00007025	+
chr2	20678463	20680000	TCONS_00004163	−
chr9	40740776	40742447	TCONS_00015810	−
chr12	96941535	96948490	TCONS_l2_00005839	+
chr2	85568532	85568853	TCONS_l2_00014667	−
chr14	69446399	69454180	ACTN1-AS1	+
chr6	166167677	166189268	TCONS_00011412	+
chr10	131164498	131166754	TCONS_00018385	+
chr15	20084119	20086550	TCONS_l2_00008388	+
chr21	43188149	43196304	TCONS_00029027+TCONS_00029028+TCONS_00029225 +TCONS_00029029	+
chr7	89467410	89468610	TCONS_00013039	+
chr5	8020150	8052115	TCONS_00009346	+
chrX	130823930	130826881	TCONS_00017372	−
chr3	5732230	5741025	TCONS_00006403	−
chr12	54556923	54562379	TCONS_00020234	+
chr6	169467913	169469519	TCONS_00011420+TCONS_00011421	+
chr13	19379529	19393469	TCONS_l2_00007046	−
chr15	50655998	50660476	TCONS_00023262	+
chr2	16704444	16710706	TCONS_00002837+TCONS_00002838	+
chr16	33790306	33794149	TCONS_l2_00010135+TCONS_l2_00010136	−
chr14	87499717	87515943	TCONS_00022822	−
chr15	36399180	36475165	TCONS_00023347+TCONS_00023348	+
chr13	50964334	51205735	TCONS_00021487+TCONS_00021486+TCONS_00021791 +TCONS_00021488+TCONS_00021489+TCONS_0002149 0+TCONS_00021491+TCONS_00021492	+
chr9	90475210	90480763	TCONS_00016392	−
chr17	26553589	26555085	PYY2	+
chr20	39323050	39346184	TCONS_00028388+TCONS_00028389+TCONS_00028045	−
chr18	72824305	72837517	TCONS_00026565	−
chr10	134247237	134254988	TCONS_00018072	−
chr18	22080015	22081308	TCONS_00026299+TCONS_00026607	+
chr2	70351168	70352448	LOC100133985	−

chr3	113432278	113433137	TCONS_00007239	−
chr4	176329377	176346862	TCONS_00008341	+
chr12	109029645	109036526	TCONS_00020560	+
chrX	74960373	74962914	TTC3P1	−
chr3	141570621	141580150	TCONS_00006248+TCONS_00006992	+
chrY	20952602	20981484	TCONS_l2_00030937	−
chr13	66515195	66551943	TCONS_00021814	+
chr3	181584138	181584824	TCONS_l2_00019022	+
chr11	116645826	116646592	TCONS_00019175	−
chr11	10315852	10324233	TCONS_00019230	+
chr1	9509253	9514916	TCONS_00001386	−
chr15	102040557	102041937	TCONS_00023841	−
chr2	122407230	122486136	LOC254128	+
chr19	19852148	19866484	TCONS_l2_00012377	+
chr13	23500257	23509087	TCONS_00021720+TCONS_00021721+TCONS_00022140 +TCONS_00021722	+
chr3	180585947	180589258	TCONS_00006322+TCONS_00005442	+
chr6	137316307	137320641	TCONS_00011380	+
chr6	167313394	167318514	TCONS_00012016+TCONS_00011414	+
chr12	32803414	32810771	TCONS_00020373	+
chr22	25844054	25857645	CRYBB2P1	+
chr20	52463387	52466442	TCONS_00028226+TCONS_00028227	+
chrX	4545241	4551613	TCONS_00017032	−
chr13	35003658	35075454	TCONS_00021749	+
chr3	164169033	164405798	TCONS_00007028+TCONS_00006293+TCONS_000 07029+TCONS_00007030+TCONS_00006294+TCO NS_00007031	+
chr5	2303866	2312419	TCONS_00010247	−
chr4	138948577	139051839	LINC00616	−
chr1	180875787	180878302	TCONS_l2_00002250+TCONS_l2_00002251+TCONS_ l2_00000708	+
chr1	29656132	29676626	TCONS_00000494	−
chr4	55469380	55473298	TCONS_00007422	−
chrY	6258442	6279605	TTTY1B	+
chr20	16838607	16844793	TCONS_00028009	−
chr1	98398902	98440557	TCONS_00001603+TCONS_00002412	−
chr12	91203014	91204357	TCONS_00020515	+
chr10	66662885	66684276	TCONS_00017990	−
chr5	71370758	71374133	TCONS_00010369	−
chr17	32800813	32803129	TCONS_00025354	+
chr4	115379252	115412059	TCONS_00008199+TCONS_00008871	+
chr12	119082654	119083867	TCONS_00020943	−
chr2	64747476	64751427	TCONS_00005168	−
chr5	92242887	92275298	TCONS_00009729	−
chr14	66219257	66220716	TCONS_00022531	+
chr5	108061406	108063994	TCONS_00009477	+
chr5	99737145	99756452	TCONS_00009471+TCONS_00010049+TCONS_00010754	+
chr17	36158869	36162759	TCONS_00025224	−

chr8	40019006	40030549	TCONS_00014684	+
chr4	73435480	73435879	TCONS_00008102	+
chr8	62729589	62734403	TCONS_00015023	−
chr11	130937380	130957497	TCONS_00019541	+
chr6	159803508	159817475	TCONS_00012007+TCONS_00011404	+
chr11	41733918	41736153	TCONS_00019621	−
chr4	156207314	156225932	TCONS_l2_00021363+TCONS_l2_00022036	−
chr21	40360401	40384579	TCONS_l2_00017237+TCONS_l2_00017238+TCONS_l2_00017239+TCONS_l2_00017507+TCONS_l2_00017240+TCONS_l2_00017241+TCONS_l2_00017242+TCONS_l2_00017508	−
chr6	160131442	160134505	TCONS_l2_00024507+TCONS_l2_00024508+TCONS_l2_00024509	+
chrY	6317509	6325947	TTTY7B	+
chr16	51558115	51559006	TCONS_00024398	+
chr10	4692377	4720262	LINC00704	−
chr7	46017133	46030148	TCONS_00013437	+
chr1	29194087	29197779	TCONS_00000174	+
chr6	119671082	119738843	TCONS_00011937	+
chr2	10589126	10597755	TCONS_00004692+TCONS_00004693+TCONS_00004694+TCONS_00003567+TCONS_00003568+TCONS_00003569+TCONS_00003570+TCONS_00004695+TCONS_00004696+TCONS_00003571	+
chr7	99517711	99519083	TCONS_00013551	+
chr17	6258067	6265199	TCONS_00025541	−
chr16	79692081	79692378	TCONS_00024735	−
chr4	93183649	93198453	TCONS_00007429+TCONS_00007796	−
chr15	58572415	58576475	TCONS_00023687	−
chr6	167560024	167563500	TCONS_l2_00024523	+
chr15	29087009	29089115	TCONS_l2_00008965	−
chr17	38276065	38278125	TCONS_00025630+TCONS_00025631+TCONS_00026069	−
chr2	15830906	15859085	TCONS_00002688+TCONS_00003585	+
chr2	80926513	81097315	TCONS_00003327	−
chr9	118358893	118365964	TCONS_00016137	+
chr16	71547732	71551750	TCONS_00024452+TCONS_00024451+TCONS_00024453	+
chr10	29078240	29084979	LINC00837	−
chr10	88511412	88511863	TCONS_l2_00003144	+
chr7	7589264	7606237	TCONS_00013688+TCONS_00013689+TCONS_00013690+TCONS_00013691+TCONS_00014250+TCONS_00014251+TCONS_00014252	−
chr1	14609149	14630936	TCONS_00001392	−
chr4	60633534	60657832	TCONS_00007772	−
chr5	124329349	124331780	TCONS_00010462	−
chr1	224954058	224967057	TCONS_00001303+TCONS_00000410	+
chr2	225016562	225034805	TCONS_l2_00014275	+
chr4	120314433	120316288	TCONS_l2_00021261+TCONS_l2_00021262	−
chr6	159796864	159804337	TCONS_00012345+TCONS_00012346	−
chr2	71229661	71291873	TCONS_l2_00015840+TCONS_l2_00014643	−
chrX	105252196	105253194	TCONS_00017226	+

chr4	42708124	42709352	TCONS_00007521	+
chr18	7508432	7509419	TCONS_00026398	−
chr6	29444483	29447059	TCONS_00012136	−
chr12	130442122	130445606	TCONS_00020651+TCONS_00020652+TCONS_00020653	+
chr17	41999868	42016323	TCONS_l2_00010785+TCONS_l2_00010786+TCONS_l2_00010787+TCONS_l2_00010788+TCONS_l2_00010790+TCONS_l2_00010789+TCONS_l2_00010791+TCONS_l2_00010792+TCONS_l2_00010793	+
chr2	62683442	62688910	TCONS_00004274	−
chr15	24532702	24544974	TCONS_00023865	+
chr17	46810453	46821265	TCONS_l2_00011563+TCONS_l2_00010847	+
chr13	111461359	111472540	TCONS_l2_00007006+TCONS_l2_00007007	+
chr1	166864948	166877317	TCONS_00000666	−
chr6	113667194	113698013	TCONS_00012773+TCONS_00011540+TCONS_00012252+TCONS_00012253	−
chr4	1145160	1147489	TCONS_00007377	+
chr4	106461347	106473512	TCONS_00007808	−
chrY	16388092	16389369	TCONS_00017625	−
chr9	13441189	13487510	TCONS_l2_00028623+TCONS_l2_00028624+TCONS_l2_00028625+TCONS_l2_00029679	+
chrY	26316687	26318468	TCONS_l2_00030914	+
chr1	184618284	184622348	TCONS_00001756	−
chr1	71110666	71114581	TCONS_00001523	−
chr4	1000614	1002952	TCONS_00007951	+
chr4	153662550	153667773	TCONS_00007884	−
chr14	71075515	71108015	TCONS_00022374	−
chr6	143839018	143845396	TCONS_00011979	+
chr12	133692099	133698651	TCONS_00020666	+
chr5	139123577	139125847	TCONS_00010104	+
chr10	38464599	38503273	LOC100129055	+
chr20	56130115	56134053	TCONS_00028072	−
chrX	108811931	108812305	TCONS_l2_00030329	+
chr3	70654585	70666843	TCONS_00006530+TCONS_00006532+TCONS_00006533	−
chr20	23301591	23305689	TCONS_00028341	−
chr9	1045426	1049242	TCONS_00015901+TCONS_00015902+TCONS_00015639+TCONS_00015903	+
chr11	7009115	7018463	TCONS_00019568	−
chr2	110969106	110980517	LINC00116	−
chr15	102142910	102154530	TCONS_00023842+TCONS_00023843+TCONS_00023844+TCONS_00024221	−
chr13	84238356	84240952	TCONS_00022069	−
chr21	33890761	33892029	TCONS_00028994	+
chr5	127174178	127193496	TCONS_00009779	−
chr8	16225153	16230797	TCONS_00014938	−
chr11	117005775	117007618	TCONS_l2_00005211	−
chr2	189462696	189464061	TCONS_00004531	−
chr6	132455118	132490514	LOC100507254	+
chr3	195367106	195377621	TCONS_00007073+TCONS_00006377+TCONS_00007074+TCONS_00006378	+

chr2	128569621	128570335	TCONS_00003866	+
chr15	22195622	22199973	TCONS_00023212	+
chr8	124571706	124573897	TCONS_00015157	−
chr8	28911180	28911635	TCONS_00015260	+
chr7	64338163	64340136	TCONS_00013484	+
chr19	12754021	12754774	TCONS_l2_00012873+TCONS_l2_00012874	−
chr2	224370194	224375651	TCONS_00003117	+
chr2	162101250	162105241	TCONS_00002724+TCONS_00003039	+
chr2	98286206	98319529	LOC728537	+
chr15	43991625	43993449	TCONS_l2_00009062	−
chr17	72555684	72563679	TCONS_l2_00011463+TCONS_l2_00011464	−
chr21	40383133	40386878	TCONS_00029017	+
chr7	138087706	138089261	TCONS_l2_00026918	−
chr15	30799598	30808898	TCONS_l2_00008524+TCONS_l2_00008525+TCONS_l2_00008526+TCONS_l2_00008527	+
chr1	27364505	27391197	TCONS_00000869+TCONS_00000870	+
chr4	138114910	138119520	TCONS_00007404	+
chr11	68638132	68642010	TCONS_00019150	+
chr10	3954182	3978004	TCONS_l2_00002846+TCONS_l2_00003879+TCONS_l2_00003880	+
chr4	190700498	190861824	TCONS_l2_00021490+TCONS_l2_00021491+TCONS_l2_00021492+TCONS_l2_00022098+TCONS_l2_00021493+TCONS_l2_00022099+TCONS_l2_00022100+TCONS_l2_00022101+TCONS_l2_00021494+TCONS_l2_00021495+TCONS_l2_00021496+TCONS_l2_00021497	−
chrX	106947654	106951858	TCONS_00017228	+
chr2	123801120	123804985	TCONS_00003848	+
chr3	181281327	181283447	TCONS_00006717	−
chr1	106590150	106615801	TCONS_00001620+TCONS_00001621+TCONS_00002427+TCONS_00002428+TCONS_00001622	−
chr4	124945249	124949518	TCONS_00008567+TCONS_00008568+TCONS_00008569	−
chr1	225898670	225923976	TCONS_00000747	−
chr1	85463022	85463597	TCONS_00001009	+
chr1	187454055	187515717	TCONS_l2_00001689	−
chr2	222688046	222688435	TCONS_00004039	+
chr4	36498012	36643522	TCONS_l2_00021103+TCONS_l2_00021858+TCONS_l2_00021859+TCONS_l2_00021860	−
chr20	48780001	48782639	TCONS_00027944	+
chr2	171579719	171627276	TCONS_l2_00016011+TCONS_l2_00014980+TCONS_l2_00014981+TCONS_l2_00014982	−
chr1	27974930	27976234	TCONS_l2_00000181	+
chr2	146326862	146345592	TCONS_00003415	−
chr5	44615145	44633787	TCONS_00009955	+
chr2	121895708	121896762	TCONS_00004430	−
chr14	50410255	50428678	TCONS_00022368+TCONS_00022741	−
chr5	1931105	2067530	TCONS_00009832+TCONS_00009833+TCONS_00009834+TCONS_00009835+TCONS_00009326+TCONS_00009327+TCONS_00009328	+
chr8	100008717	100025293	TCONS_l2_00028339+TCONS_l2_00028340	−

chr8	29578776	29605625	LINC00589	−
chr3	67891019	67939460	TCONS_00006084	+
chr13	53058296	53058815	TCONS_00021655	−
chr2	8279532	8283399	TCONS_00002812	+
chr8	99327931	99333207	TCONS_00015111	−
chr10	52386924	52407050	TCONS_l2_00003053+TCONS_l2_00003054+TCONS_l2_00003055+TCONS_l2_00003983+TCONS_l2_00003056	+
chr6	53863688	53871950	MLIP-IT1	+
chrX	55934676	56041429	TCONS_l2_00030220+TCONS_l2_00030221	+
chr4	33435132	33439499	TCONS_00007506	+
chr14	101536824	101539988	TCONS_00022413+TCONS_00022876	−
chr20	1810675	1816693	TCONS_00028090	+
chr20	51474877	51479394	TCONS_00028067	−
chr12	127399783	127544942	TCONS_00020226	−
chr17	61977916	61979254	TCONS_00025457	+
chr16	65901327	65905189	TCONS_00024699	−
chr1	247496212	247556961	TCONS_00002272+TCONS_00001360+TCONS_00001361+TCONS_00001362	+
chr13	112760409	112761196	TCONS_00021697	−
chr6	30834759	30843695	TCONS_l2_00025370	−
chr5	133842305	133844920	TCONS_00009259	+
chr19	22208108	22217980	TCONS_00027227	−
chr6	163759374	163768065	DKFZp451B082	−
chr13	45225948	45275152	TCONS_00021544	+
chr3	9291438	9299191	TCONS_00006780+TCONS_00006781+TCONS_00005944+TCONS_00005493	+
chr2	151451616	151476581	TCONS_00003421	−
chr12	76953674	77009334	TCONS_l2_00005764+TCONS_l2_00005765	+
chr1	166356964	166421869	TCONS_00000665	−
chr3	151791249	151831224	TCONS_00006275	+
chr2	131174326	131186119	LOC100216479	−
chr1	89938509	89939606	TCONS_00001568	−
chr7	23245632	23247664	TCONS_00012871	+
chrX	12844420	12845076	TCONS_l2_00030147+TCONS_l2_00030148	+
chr10	38692121	38712064	TCONS_l2_00003945+TCONS_l2_00003946+TCONS_l2_00002990+TCONS_l2_00002991+TCONS_l2_00002992+TCONS_l2_00002993	+
chr14	105491240	105493581	TCONS_00022896	−
chr7	121856401	121858491	TCONS_00013068	+
chr13	100557626	100560611	TCONS_00021881	+
chr20	54611817	54615954	TCONS_00028230	+
chr17	79482373	79494802	TCONS_00025524+TCONS_00025202	+
chr3	191274952	191309646	TCONS_00006347+TCONS_00006348+TCONS_00005690+TCONS_00006349+TCONS_00005691	+
chr1	234782035	234818922	TCONS_00000069+TCONS_00000421+TCONS_00000422+TCONS_00000423	+
chr4	190200225	190201335	TCONS_00008716+TCONS_00009206	−
chr1	1533413	1545864	TCONS_00000803+TCONS_00000804+TCONS_00000033+TCONS_00000137+TCONS_00000805	+

chr15	47104362	47106170	TCONS_00023667	−
chr9	122131807	122135007	TCONS_00016147	+
chr3	127199533	127257825	TCONS_00005824+TCONS_00005468+TCONS_00006645+TCONS_00006646+TCONS_00006647+TCONS_00006648+TCONS_00006649+TCONS_00006650	−
chr12	80419461	80423155	TCONS_l2_00005785	+
chr14	21258299	21267971	TCONS_00022420	+
chr17	20512697	20536547	TCONS_l2_00011142+TCONS_l2_00011143+TCONS_l2_00011634	−
chr14	90918592	90923131	TCONS_00023147+TCONS_00022831	−
chr22	25608709	25609652	TCONS_00029371	+
chr16	19345522	19349104	TCONS_00024341	+
chr12	105836749	105837870	TCONS_00020904	−
chr20	46470886	46471713	TCONS_00028417	−
chr2	131424139	131437474	TCONS_00003872	+
chr16	84375964	84376974	TCONS_00024485	+
chr5	39721152	39721560	TCONS_l2_00022861	−
chr3	158586273	158602973	TCONS_00006290+TCONS_00006291	+
chr1	101616109	101641117	TCONS_00001078+TCONS_00002106+TCONS_00001079+TCONS_00001080	+
chr2	113436287	113438656	TCONS_00003820+TCONS_00004873	+
chr7	52561402	52569695	TCONS_00013447	+
chr17	60308879	60324419	TCONS_l2_00011391	−
chr2	213692663	213699821	TCONS_l2_00014219	+
chr10	119494367	119512834	TCONS_00018348+TCONS_00018349+TCONS_00017872+TCONS_00018350	+
chr20	47927061	47927848	TCONS_00028432	−
chr1	11669588	11673412	TCONS_00000153	+
chr4	11742038	11771223	TCONS_00008436+TCONS_00007716+TCONS_00009028	−
chr9	126772809	126773393	TCONS_00015748	+
chr1	14923167	14924613	TCONS_00001395	−
chr11	23782899	23789362	TCONS_00019592	−
chr7	2423756	2428232	TCONS_l2_00027030	+
chr14	86802754	86824026	TCONS_00022580	+
chr13	111463059	111465705	TCONS_00022314	−
chr7	133760596	133785005	TCONS_00014218	+
chr2	55378213	55397546	TCONS_00004256	−
chrY	13309475	13370674	TCONS_l2_00030931+TCONS_l2_00030932+TCONS_l2_00030933	−
chr2	107103833	107124389	TCONS_l2_00015431+TCONS_l2_00015432+TCONS_l2_00013879+TCONS_l2_00013880	+

chr2	111953743	112253382	TCONS_l2_00014783+TCONS_l2_00014784+TCONS_l2_00014785+TCONS_l2_00014786+TCONS_l2_00014787+TCONS_l2_00014788+TCONS_l2_00014789+TCONS_l2_00014790+TCONS_l2_00014791+TCONS_l2_00015909+TCONS_l2_00014792+TCONS_l2_00014793+TCONS_l2_00014794+TCONS_l2_00014795+TCONS_l2_00015910+TCONS_l2_00015911+TCONS_l2_00014796+TCONS_l2_00014798+TCONS_l2_00014797+TCONS_l2_00014799+TCONS_l2_00015912+TCONS_l2_00015913+TCONS_l2_00015914+TCONS_l2_00014800+TCONS_l2_00015915+TCONS_l2_00014801+TCONS_l2_00014802+TCONS_l2_00015916+TCONS_l2_00015917+TCONS_l2_00015918+TCONS_l2_00014803+TCONS_l2_00015919+TCONS_l2_00015920+TCONS_l2_00014804+TCONS_l2_00014805+TCONS_l2_00015921+TCONS_l2_00015922	−
chr15	32892388	32893206	TCONS_l2_00009427	+
chr7	155207581	155211508	TCONS_00013650+TCONS_00014237	+
chr19	59086766	59095762	MGC2752	+
chr4	90381044	90388932	TCONS_00008528	−
chr8	120049750	120050454	TCONS_00014840	+
chr2	108263408	108267421	TCONS_00004370	−
chr5	67975968	67976938	TCONS_00009990	+
chr5	8333596	8457677	LOC729506	−
chr16	32162609	32163874	HERC2P4	−
chr5	177663414	177664575	TCONS_00010856	+
chr18	6774030	6779022	TCONS_00026594	+
chr7	53448226	53486571	TCONS_00013804+TCONS_00013803+TCONS_00013802	−
chr3	101659703	101716770	LOC152225	+
chr13	112911042	112927327	TCONS_00021698+TCONS_00022317+TCONS_00022123+TCONS_00022124	−
chr6	31167942	31169695	TCONS_00011481	−
chr4	138969404	138984013	TCONS_l2_00020780	+
chr8	20917383	20986642	TCONS_00014947	−
chr20	23337231	23338851	TCONS_00028023+TCONS_00028022+TCONS_00028342+TCONS_00028343+TCONS_00028024+TCONS_00028344+TCONS_00028642	−
chr2	134785346	134788845	TCONS_00004454	−
chr7	149589257	149606765	TCONS_l2_00026264	+
chr14	54854660	54859274	TCONS_00022750	−
chr2	241861970	241869675	TCONS_l2_00014322	+
chr13	39887794	39911246	TCONS_00021975+TCONS_00021976	−
chr9	1633518	1929562	TCONS_00016530+TCONS_00015907	+
chr17	26557602	26561264	TCONS_00025599	−
chr5	12914180	13032998	TCONS_00009607	−
chr5	55832410	55841007	TCONS_00009670+TCONS_00010346	−
chr7	65300347	65310515	TCONS_l2_00025984	+
chr13	29152220	29174321	TCONS_l2_00006768	+
chr4	53581118	53588673	TCONS_l2_00021137	−

chr10	107830963	107832004	TCONS_00018599	−
chr13	23471169	23472320	BASP1P1	−
chr12	54536059	54546523	TCONS_00020435+TCONS_00020436+TCONS_00020437 +TCONS_00021139	+
chr12	126929748	126933882	TCONS_00020972	−
chr21	27722099	27722953	TCONS_00028905	−
chr7	153086676	153093207	TCONS_00013634+TCONS_00013635	+
chr21	40395696	40402611	TCONS_00029139+TCONS_00028936+TCONS_00029140	−
chr1	231454150	231456100	TCONS_00001324	+
chr20	46749594	46755525	TCONS_00028422	−
chr6	171004402	171006035	TCONS_00011200	+
chr9	128172737	128184020	TCONS_00016154+TCONS_00016693+TCONS_00016155	+
chr1	50723329	50723876	TCONS_00000933	+
chr11	103570086	103571381	TCONS_00019741	−
chr22	16162066	16172265	LINC00516	+
chr5	40260759	40267759	TCONS_00009389	+
chr2	64313484	64315380	TCONS_00003301	−
chr13	37363457	37369350	TCONS_l2_00006790+TCONS_l2_00006791+TCONS_ l2_00006792	+
chr16	66931240	66934554	TCONS_00024710+TCONS_00025039	−
chr1	60580547	60615351	TCONS_00000213+TCONS_00000965	+
chr22	21396681	21398538	P2RX6P	−
chr8	109928109	109930877	TCONS_00015134	−
chr9	38650196	38662716	TCONS_00015976+TCONS_00015978+TCONS_00016578	+
chr13	26626414	26627523	TCONS_00021614	−
chr9	11143876	11194381	TCONS_00016269	−
chr1	16983392	16993712	TCONS_l2_00001058	−
chr3	13692221	13788132	LINC00620	+
chr20	22665567	22709111	TCONS_00028127+TCONS_00028128+TCONS_00028516 +TCONS_00028517	+
chr10	65796105	65797040	TCONS_00017989	−
chr20	16089732	16091298	TCONS_00028321	−
chr19	55025381	55027669	TCONS_00027419	−
chr16	64294226	64294926	TCONS_l2_00010222	−
chr16	89557726	89565716	TCONS_00024914+TCONS_00024522	+
chr12	103325190	103345356	TCONS_00020899+TCONS_00021396	−
chr12	69177485	69187742	TCONS_00020464	+
chr16	24621561	24652285	TCONS_00024354	+
chrX	39868529	39871435	TCONS_00016930	+
chr9	129278696	129281087	TCONS_00015753	+
chr11	94883703	94892312	TCONS_00019107+TCONS_00019445	+
chr12	133696792	133706854	TCONS_l2_00006563+TCONS_l2_00006564	−
chr3	196181573	196192929	TCONS_l2_00019107	+
chr15	86621833	86623017	TCONS_00023767	−
chr1	47638527	47640990	TCONS_00001483	−
chr8	99057943	99059896	TCONS_00015327	+
chr10	44354859	44390881	LINC00840	+
chr7	63145118	63153062	TCONS_00013465	+

chr4	6675821	6677774	LOC93622	+
chr18	9008804	9010367	TCONS_00026400+TCONS_00026401	−
chr3	191470957	191477492	TCONS_00006350	+
chr3	181867283	181895007	TCONS_00006719+TCONS_00007316+TCONS_00007317	−
chr1	198867623	198906558	TCONS_00000111	−
chr1	53656725	53657133	TCONS_00000942	+
chr4	22990820	23125110	TCONS_l2_00020445+TCONS_l2_00020444+TCONS_l2_00020443+TCONS_l2_00021544+TCONS_l2_00020446+TCONS_l2_00021545+TCONS_l2_00021546+TCONS_l2_00021547+TCONS_l2_00020447	+
chr4	90507067	90623525	TCONS_00008530	−
chr19	58782949	58785962	TCONS_00027642	+
chr7	115287515	115316383	TCONS_00013230	−
chr12	103545604	103571987	TCONS_00021195+TCONS_00021196+TCONS_00020543+TCONS_00021197+TCONS_00021198+TCONS_00021199+TCONS_00020545+TCONS_00020544+TCONS_00021201+TCONS_00021200	+
chr7	2514360	2516528	TCONS_l2_00027263+TCONS_l2_00027264	−
chr5	92424509	92426530	TCONS_00010040	+
chr22	34745820	34756219	TCONS_00029688	−
chr4	38368535	38387380	TCONS_00007514	+
chr9	31909974	31917026	TCONS_00016287	−
chr17	56209	56601	TCONS_00025791	+
chr1	995080	1002533	TCONS_l2_00000030+TCONS_l2_00001952+TCONS_l2_00000031+TCONS_l2_00001953+TCONS_l2_00001954+TCONS_l2_00001955	+
chr16	56677599	56678853	MT1DP	+
chr17	35015951	35040059	TCONS_00025369	+
chrX	134083579	134087412	TCONS_00017013	+
chr6	160131219	160134936	TCONS_l2_00025008+TCONS_l2_00025009+TCONS_l2_00025010	−
chr9	25780054	25812963	TCONS_00015572+TCONS_00015657+TCONS_00016547+TCONS_00015936+TCONS_00015937+TCONS_00016548+TCONS_00015938	+
chr7	138885293	138886134	TCONS_00013609	+
chr4	138864151	138874368	TCONS_00008262+TCONS_00008263+TCONS_00008264	+
chr9	95555561	95559556	TCONS_00016074	+
chr10	6298406	6316084	TCONS_00018424	−
chr2	18604800	18606570	TCONS_00004157	−
chr9	109346423	109354233	TCONS_00015863	−
chr6	105374299	105379376	TCONS_00012237+TCONS_00012238	−
chr5	85382826	85385030	TCONS_l2_00022397+TCONS_l2_00022398	+
chr2	239840998	239847965	FLJ43879	−
chr2	3135353	3149551	TCONS_00003155+TCONS_00003156	−
chr14	38092102	38184001	TCONS_00022970	+
chr9	116413236	116445135	TCONS_l2_00029836+TCONS_l2_00028996	+
chr21	18235195	18246011	TCONS_00028873	−
chrY	8263084	8271013	TCONS_00017631	+
chr9	27245472	27246282	TCONS_00015939	+
chr9	138137542	138144554	TCONS_00015894	−

chr1	26325875	26331040	TCONS_00001436	−
chr20	7050261	7127303	TCONS_00027898	+
chr19	35067638	35068596	SCGB1B2P	−
chr1	38674706	38680439	LOC339442	−
chr4	88192262	88199855	TCONS_00008153	+
chr10	2114039	2123291	TCONS_00017756+TCONS_00017757	+
chr17	34897582	34900678	TCONS_00025223	−
chr1	117647169	117647504	TCONS_00002142	+
chr11	58652424	58660266	TCONS_l2_00005312	+
chr19	19867196	19874469	TCONS_00026934	+
chr15	24356507	24358424	TCONS_00023302	+
chr4	66724479	66725922	TCONS_00007546	+
chr8	70270873	70306748	TCONS_00014747	+
chr7	13141016	13743774	TCONS_00012955+TCONS_00012956	+
chr11	4415042	4432109	TCONS_00019831	+
chr12	75379561	75382123	TCONS_00020472	+
chr4	12225075	12252877	TCONS_l2_00021530+TCONS_l2_00020430+TCONS_ l2_00020429+TCONS_l2_00020428	+
chr4	59431580	59433840	TCONS_00008088	+
chr16	14456966	14464123	TCONS_00024264	−
chr9	136999157	137001037	TCONS_00016499+TCONS_00016500	−
chr3	147225698	147228122	TCONS_00006255	+
chr7	116203648	116254874	TCONS_l2_00027468+TCONS_l2_00026855+TCONS_ l2_00027469+TCONS_l2_00026856+TCONS_ l2_00027470+TCONS_l2_00026857+TCONS_l2_00027471	−
chr15	41244055	41245400	TCONS_00023380	+
chr9	135903518	135905802	TCONS_00016498	−
chr15	95635207	95676075	TCONS_00023798	−
chr8	125287977	125289693	TCONS_00014849+TCONS_00014850	+
chr17	4979205	4981678	TCONS_00025535	−
chr5	6795993	6826841	TCONS_00009340	+
chr21	46720160	46725172	TCONS_00028958	−
chr1	10516603	10520093	TCONS_00001938+TCONS_00000152	+
chrX	49155629	49157930	TCONS_00017170	+
chr19	34044018	34048344	TCONS_00027517+TCONS_00026996	+
chr4	4922513	4935669	TCONS_00007965	+
chr7	129246527	129251436	TCONS_00013953	−
chr10	45073475	45086387	TCONS_00018191	+
chr2	21316935	21319427	TCONS_00004172	−
chr18	45516668	45518484	TCONS_00026519	−
chr5	173213542	173217981	TCONS_00010562+TCONS_00011091+TCONS_00011092 +TCONS_00011093+TCONS_00011094+TCONS_0001056 3+TCONS_00011095	−
chr18	8974493	8998420	TCONS_00026275+TCONS_00026274+TCONS_00026597 +TCONS_00026596+TCONS_00026595	+
chr19	43882723	43883356	TCONS_l2_00013129	−
chr11	45011207	45029701	TCONS_00019628	−
chrY	9861073	9871730	TCONS_l2_00030927+TCONS_l2_00030928	−
chr12	20098289	20098872	TCONS_00020725	−

chr4	124247730	124249559	TCONS_00008217	+
chr20	60809846	60812575	TCONS_00028459	−
chr2	223181503	223186428	TCONS_l2_00014267+TCONS_l2_00014268+TCONS_l2_00014269+TCONS_l2_00014270+TCONS_l2_00014271+TCONS_l2_00015610	+
chr5	34651582	34652810	TCONS_00010297	−
chr7	4452562	4456136	TCONS_00013682	−
chr7	36015511	36028781	TCONS_00013778	−
chrX	15250368	15254002	TCONS_00017489	−
chr2	86116278	86144322	TCONS_00003756+TCONS_00004836+TCONS_00003757+TCONS_00003758+TCONS_00002710+TCONS_00002945+TCONS_00004837+TCONS_00004838	+
chr8	145069127	145082072	TCONS_00015196	−
chr6	11934223	11961218	TCONS_00011693+TCONS_00011694+TCONS_00012426	+
chr10	134757691	134761035	TCONS_00018394	+
chr21	37377457	37380899	TCONS_00029116	−
chr4	170833983	170862823	TCONS_00008928+TCONS_00008317+TCONS_00008929+TCONS_00008930	+
chr12	31903114	31932287	TCONS_00020754	−
chr7	46516055	46521176	TCONS_00013164+TCONS_00013165+TCONS_00013797	−
chr6	5851739	5870453	TCONS_00011258	+
chr10	1205708	1210612	LINC00200	+
chr5	135243862	135257996	TCONS_00010494	−
chr4	79567148	79605655	LOC100505702	+
chr12	67285593	67301326	TCONS_00020821	−
chr19	35034793	35036389	TCONS_l2_00013033+TCONS_l2_00013034	−
chr8	102019205	102029741	TCONS_l2_00028504	+
chr6	137865118	137865527	TCONS_l2_00024933	−
chr4	119541480	119541991	TCONS_00008208	+
chr5	7363063	7373626	TCONS_00010894+TCONS_00010895+TCONS_00010262+TCONS_00010263+TCONS_00009599+TCONS_00009600	−
chr8	1304795	1306100	TCONS_00014575	+
chr2	239697289	239698357	TCONS_00003520	−
chr1	120612586	120638073	TCONS_00001131	+
chr5	173128538	173135199	TCONS_00010181	+
chr2	221495107	221498693	TCONS_00004034	+
chr11	125801068	125809290	TCONS_00019794	−
chr1	118738866	118749628	TCONS_00001117	+
chr1	159968942	159987494	TCONS_00001704	−
chr15	83626882	83654620	TCONS_00023251	−
chr17	19374432	19398577	TCONS_00025218+TCONS_00025586	−
chr2	73090670	73112750	TCONS_00004306	−
chr19	4584362	4586076	TCONS_00026870	+
chr2	52721826	52727890	TCONS_00003283	−
chr1	212398619	212458414	TCONS_00001802+TCONS_00002577+TCONS_00002578+TCONS_00000724+TCONS_00001803+TCONS_00000725+TCONS_00000726+TCONS_00001804+TCONS_00001805	−

chr4	178607827	178612222	TCONS_00007910	−
chr8	54406309	54408063	TCONS_00015007	−
chr10	6667903	6668997	TCONS_00018111	+
chr5	10479483	10482421	TCONS_00009352	+
chr3	133784147	133794057	TCONS_00005831	−
chr4	109590009	109592299	TCONS_00008192	+
chr1	246846453	246855175	TCONS_00001350+TCONS_00000429	+
chr5	139529833	139531887	TCONS_00010108	+
chr3	171185873	171187422	TCONS_00005658	+
chr13	63609109	63671829	TCONS_00021810	+
chr9	120521861	120639328	TCONS_00016675+TCONS_00016141+TCONS_00016676 +TCONS_00015742+TCONS_00016142	+
chr5	172812059	172898212	TCONS_00010177+TCONS_00010178+TCONS_00010836	+
chr11	39115323	39115911	TCONS_00019620	−
chr19	53716201	53719355	ZNF818P	+
chr7	48700171	48702901	TCONS_00013167	−
chr4	38723386	38740755	TCONS_00008048	+
chr16	10715085	10716242	TCONS_00024577	−
chr12	133169646	133172672	TCONS_00021013+TCONS_00021014+TCONS_00021461	−
chr9	79131525	79163468	TCONS_00016019	+
chr4	185427282	185436808	TCONS_00007688	+
chr1	149993003	149998813	TCONS_00001675	−
chr9	44997777	44999351	TCONS_l2_00029735	+
chr22	41994034	41995651	TCONS_00029427	+
chrX	152583139	152587121	TCONS_l2_00030632	−
chr1	47643841	47644943	TCONS_00000515	−
chr1	74100899	74180897	TCONS_l2_00002080+TCONS_l2_00000337+TCONS_ l2_00002081	+
chr9	2992062	3014177	TCONS_l2_00029897	−
chr14	56967798	56970532	TCONS_00022518	+
chr2	108439520	108442583	LOC729121	−
chr14	64212691	64214050	TCONS_00022768	−
chr21	34402769	34407541	TCONS_00029274+TCONS_00029275	−
chr10	45586675	45587949	TCONS_00018195	+
chr3	12916997	12918609	TCONS_00005948	+
chr21	16134031	16135411	TCONS_00028792	+
chr1	28446484	28447850	TCONS_l2_00001111+TCONS_l2_00001110	−
chr5	1543089	1551825	TCONS_l2_00022746+TCONS_l2_00023633	−
chr1	202794329	202795421	LOC641515	+
chrX	115032519	115085422	TCONS_00017083+TCONS_00016951	−
chr20	56532182	56534716	TCONS_00028074	−
chr1	79775130	79779074	TCONS_00001533	−
chr6	170340754	170348805	TCONS_00012630+TCONS_00012027	+
chr17	81189540	81190888	TCONS_00025203	+
chrX	101804893	101826621	NXF4	+
chr12	65938019	65951091	TCONS_00020211	−
chr12	121342501	121345803	TCONS_00021221+TCONS_00020594+TCONS_00021222 +TCONS_00021223	+

chr11	114249483	114250761	TCONS_00019765	−
chr3	106527861	106562176	TCONS_00006580+TCONS_00006581+TCONS_00006582+TCONS_00007222+TCONS_00006583+TCONS_00006584+TCONS_00006585+TCONS_00007223+TCONS_00007224	−
chr6	153625730	153630117	TCONS_00011598+TCONS_00012813	−
chr4	78075946	78077856	TCONS_00008508+TCONS_00008509	−
chr10	131170474	131179081	TCONS_00018642+TCONS_00019053	−
chr13	20530917	20532202	TCONS_00022136	+
chr9	46122189	46123597	TCONS_00016324	−
chr21	23582258	23793139	TCONS_00028806	+
chr12	54089330	54090975	TCONS_00020432	+
chr13	75139302	75146748	TCONS_00021834+TCONS_00022205	+
chr6	1489678	1490173	TCONS_00011633	+
chr16	58881696	58913939	TCONS_00025031	−
chr11	45392948	45410059	TCONS_00019091	+
chr1	242072240	242075035	TCONS_00001347	+
chr7	76610139	76653076	DTX2P1-UPK3BP1-PMS2P11	+
chr3	75485367	75492775	TCONS_00006894+TCONS_00006099	+
chr5	4640817	4648367	TCONS_00009852+TCONS_00009334	+
chr2	178417963	178424476	TCONS_00003952	+
chr4	133452275	133467632	TCONS_00008254	+
chr6	156193878	156203534	TCONS_00011998	+
chr2	238059252	238061515	TCONS_00004059	+
chr1	213904759	213967930	TCONS_00000389	+
chr10	107771233	107773729	TCONS_00018322	+
chr6	134142285	134175130	MGC34034	+
chr14	39218543	39386086	LINC00639	−
chr8	23082744	23088426	TCONS_00014552	−
chr9	94912663	94919727	TCONS_l2_00030010	−
chr12	9208185	9217666	LINC00612	−
chr14	103605385	103606409	TCONS_l2_00007927	+
chr3	82025722	82026638	TCONS_00006548	−
chr7	99594667	99609249	TCONS_00013553+TCONS_00013554+TCONS_00014178+TCONS_00014179+TCONS_00014180+TCONS_00013555+TCONS_00014181	+
chr1	2728415	2729257	TCONS_00000457	−
chr5	1929007	1930577	TCONS_00010246	−
chrX	103315219	103317502	H2BFXP	−
chr4	9482045	9515589	TCONS_l2_00020402+TCONS_l2_00020403	+
chrX	149391378	149391818	TCONS_00017270	+
chr3	27674464	27675319	TCONS_l2_00019209	−
chr2	7469792	7472585	TCONS_00004124	−
chr8	144714589	144717048	TCONS_00015543	−
chr11	15645178	15682125	TCONS_00019242	+
chr6	161119320	161121664	TCONS_00011406	+
chr6	143287559	143358719	LOC100507489	−

chr17	7308194	7311462	TCONS_00025824+TCONS_00025825	+
chr12	58985431	59206436	TCONS_00020448+TCONS_00020449+TCONS_00021151 +TCONS_00021152	+
chr20	25225790	25227480	TCONS_00028353	−
chr14	27342317	27349901	TCONS_00022941+TCONS_00022942+TCONS_00022943	+
chr5	88240173	88259771	TCONS_00010983	−
chr17	65760074	65761939	TCONS_00025738	−
chr22	24647589	24661492	POM121L9P	+
chr6	164007615	164009197	TCONS_00011409	+
chr2	155316369	155317332	TCONS_00003038	+
chr2	199164087	199239821	TCONS_00002773+TCONS_00003477+TCONS_00003478	−
chr9	24046212	24146893	TCONS_00015932	+
chr5	70958562	70966823	TCONS_00009994+TCONS_00010712	+
chr2	16137171	16203007	TCONS_00003212	−
chrX	140013903	140079613	TCONS_00017017	+
chr14	97059040	97062437	TCONS_00022617+TCONS_00022618+TCONS_00023049	+
chr2	92275609	92294527	TCONS_00003765	+
chr15	98083277	98101959	TCONS_00023541	+
chr7	68505139	68511790	TCONS_00013020	+
chr3	149956306	149957996	TCONS_00005621+TCONS_00005622	+
chr1	119542967	119544028	TCONS_00000618+TCONS_00000619	−
chr10	133454227	133455011	TCONS_00018647	−
chr2	111467152	111474172	TCONS_00003369	−
chr5	3036785	3043131	TCONS_00009846+TCONS_00009847	+
chr2	150624020	150715705	TCONS_00003417+TCONS_00004472+TCONS_00003418	−
chr6	159429467	159432939	TCONS_00012343	−
chr4	119804941	119805840	TCONS_00008210	+
chr2	156837402	156844609	TCONS_00003910	+
chr15	29033389	29034538	LOC100289656	+
chr5	120109041	120126473	TCONS_00010075+TCONS_00010780+TCONS_00009497	+
chr4	166420980	166424851	TCONS_00008927	+
chr9	134691579	134696375	TCONS_00015769	+
chr20	62258580	62260177	TCONS_00027984+TCONS_00027985+TCONS_00028269	+
chr2	130783572	130808704	LOC440905	−
chr10	46310876	46312772	TCONS_l2_00004165	−
chr13	107109147	107112557	TCONS_00022103	−
chr3	130236019	130279028	TCONS_00006981	+
chr18	54721804	54739350	LINC-ROR	−
chr11	133523582	133689498	TCONS_00019808+TCONS_00020155+TCONS_00019809 +TCONS_00020156	−
chr10	133772474	133773355	TCONS_00018859	+
chr22	46937392	46944093	TCONS_00029435+TCONS_00029615	+
chr14	19856361	19925333	TCONS_l2_00008343+TCONS_l2_00008346+TCONS_ l2_00007970+TCONS_l2_00008347+TCONS_l2_00008348	−
chr1	229359208	229363309	TCONS_00000750	−
chr8	107147184	107149255	TCONS_00015132	−
chr2	30515650	30523357	TCONS_00005126+TCONS_00005127	−
chr1	43820355	43824390	TCONS_00000509+TCONS_00002363+TCONS_00002364	−

chr19	14430990	14436230	TCONS_00027200	−
chr1	14717205	14746458	TCONS_00001393+TCONS_00001394	−
chr2	139362080	139370737	TCONS_00003011	+
chr2	123561297	123567020	TCONS_00005246+TCONS_00005247+TCONS_00004432+TCONS_00004433+TCONS_00005248	−
chr13	80378553	80379820	TCONS_00021576	+
chr4	185286341	185303530	TCONS_00007450+TCONS_00009188+TCONS_00008689+TCONS_00009189+TCONS_00009190+TCONS_00008690+TCONS_00008691+TCONS_00008692	−
chr22	24349937	24352852	TCONS_l2_00017979+TCONS_l2_00018369	−
chr5	32947549	32962573	LOC340113	+
chr15	55464171	55470373	TCONS_00023278	−
chr22	39686316	39686778	TCONS_00029703	−
chr8	67876445	67894344	TCONS_00015032	−
chr10	37229037	37232860	TCONS_00017813	+
chr2	182286881	182290476	TCONS_00003461	−
chr9	35913871	35914639	TCONS_00015968+TCONS_00015969	+
chr10	30248094	30249155	TCONS_00018703+TCONS_00018704	+
chr17	26257005	26257874	TCONS_00025338	+
chr14	67649711	67652438	TCONS_00022773	−
chr2	98104748	98105300	TCONS_00004346	−
chr21	46475931	46488975	TCONS_00029182	−
chr15	86629044	86659952	TCONS_00023768+TCONS_00024199+TCONS_00024200+TCONS_00023769+TCONS_00024201	−
chr16	2688983	2696130	FLJ42627	+
chr12	32026186	32040159	TCONS_00020756	−
chr19	11645496	11646816	TCONS_00026898	+
chr1	157895764	157918861	TCONS_l2_00002224+TCONS_l2_00002225	+
chr3	27674417	27688218	TCONS_l2_00018477+TCONS_l2_00018478	+
chr17	76311810	76343879	TCONS_00025201	+
chr8	7010239	7044422	TCONS_l2_00028458	+
chr15	71088936	71095027	TCONS_l2_00009514+TCONS_l2_00009515	−
chr1	523009	532878	TCONS_00000122+TCONS_00000123+TCONS_00000124+TCONS_00000125	+
chr11	18134019	18137679	SAA3P	−
chr5	43065289	43067073	LOC100132356	−
chr16	87245728	87260023	TCONS_00024298	+
chr2	54659980	54661074	TCONS_00003680	+
chr18	39060236	39100561	KC6	−
chr11	126891665	126971201	TCONS_00020018+TCONS_00019528+TCONS_00019529+TCONS_00020021+TCONS_00020020+TCONS_00020019+TCONS_00020022+TCONS_00019114+TCONS_00020023	+
chr16	2787077	2802601	SRRM2-AS1	−
chr9	23264130	23438656	TCONS_00016284	−
chr7	123914733	123927598	TCONS_00013249	−
chr15	97815423	97862496	TCONS_00023816	−
chr21	44810094	44810769	TCONS_00029160	−
chr4	99104226	99106355	TCONS_00008531	−

chr19	57813909	57814300	TCONS_00027431	−
chr4	3650108	3650922	TCONS_00007959	+
chr1	149369294	149378297	FCGR1C	+
chr5	68902926	68919906	TCONS_l2_00023407+TCONS_l2_00023408+TCONS_l2_00023409+TCONS_l2_00023411+TCONS_l2_00023410+TCONS_l2_00023412	+
chr12	89516792	89563167	TCONS_00020866	−
chr5	57186154	57194989	TCONS_00009672	−
chr1	1210339	1215800	TCONS_00000799+TCONS_00000135+TCONS_00000136	+
chr1	219395590	219398839	TCONS_00000732	−
chr9	102303431	102318400	TCONS_00016438	−
chr22	36517580	36525898	TCONS_00029901+TCONS_00029691	−
chr11	30707956	30718696	TCONS_00019601+TCONS_00019602	−
chr6	14431958	14463629	TCONS_00011699+TCONS_00012428	+
chr11	50231598	50233106	TCONS_00019636	−
chr19	53407534	53418575	TCONS_l2_00013205	−
chr9	88371000	88380205	TCONS_l2_00029435+TCONS_l2_00029436	−
chr14	36930958	36931579	TCONS_00022471	+
chr2	105720871	105721924	TCONS_00003809+TCONS_00004866	+
chr2	108002088	108159105	TCONS_00003362	−
chr22	20186296	20192440	TCONS_00029362	+
chr9	98361827	98368568	TCONS_00015851	−
chr11	71576555	71639493	LOC100133315	−
chr13	80629411	80649832	TCONS_00022301+TCONS_00022065	−
chr13	79996143	80003586	TCONS_00022061	−
chr9	117418236	117425007	TCONS_00016467+TCONS_00016856+TCONS_00016857+TCONS_00016858+TCONS_00015871+TCONS_00015872+TCONS_00016859	−
chr20	30009242	30016983	DEFB122	−
chr2	216395170	216578618	TCONS_00003100+TCONS_00003101+TCONS_00003102+TCONS_00003103+TCONS_00003104	+
chr16	53073299	53078119	TCONS_00024657+TCONS_00024658	−
chr10	3085782	3089216	TCONS_00018405	−
chr4	123651344	123653613	CETN4P	−
chr12	125666413	125668887	TCONS_00020967	−
chr9	117881818	117900688	TCONS_00015874	−
chr21	44201184	44202343	TCONS_00028941	−
chr14	36690500	36704816	TCONS_00022963+TCONS_00022469	+
chr20	12845850	12933167	TCONS_00028007+TCONS_00028621+TCONS_00028622+TCONS_00028623+TCONS_00028320	−
chr12	127629420	127630577	TCONS_00020983	−
chr11	118662487	118665346	TCONS_00020001+TCONS_00019492	+
chr6	170453080	170454248	TCONS_00012836	−
chr1	228688983	228697976	TCONS_00001318+TCONS_00001319	+
chr5	946430	956635	TCONS_00009323	+
chr2	26521475	26529191	TCONS_00003609	+
chr1	226680710	226681472	TCONS_l2_00001825	−
chr10	122713694	122714232	TCONS_00018842+TCONS_00018841+TCONS_00018840	+
chr3	197836983	197838437	TCONS_l2_00020053+TCONS_l2_00020054	+

chr1	63662012	63664798	TCONS_00002048	+
chr6	135581134	135582071	TCONS_00011379	+
chr1	200929145	200934218	TCONS_l2_00000749	+
chr4	8560326	8562363	TCONS_00008751	+
chr6	14977019	15090234	TCONS_00011458	−
chr10	29656222	29657210	TCONS_00018151	+
chr4	188735823	188789707	TCONS_00008377	+
chr20	48909257	48931456	LOC284751	+
chr3	64430907	64441739	TCONS_00005531+TCONS_00006042	+
chr2	202921651	202922260	TCONS_00004557	−
chr10	13457202	13465133	TCONS_00017790	+
chr8	74189427	74190657	TCONS_l2_00028240	−
chr2	133043368	133051950	TCONS_00003407	−
chr13	47040339	47042412	TCONS_00021785	+
chr8	59426551	59439891	TCONS_00014721+TCONS_00014722	+
chr4	39481875	39483523	LOC401127	+
chr18	14857495	14909914	TCONS_l2_00011792	+
chr6	19538728	19539166	TCONS_00011714	+
chrX	102024095	102140338	LINC00630	+
chr13	64167617	64209643	TCONS_00021811+TCONS_00022189+TCONS_00022190	+
chr7	145372975	145378462	TCONS_00013270	−
chr5	64361893	64366318	TCONS_00009981	+
chr19	12663411	12664458	TCONS_00026826	+
chrX	124338418	124339139	TCONS_00017242	+
chr1	163393106	163394820	TCONS_00001181	+
chr7	25633838	25829649	TCONS_l2_00027297+TCONS_l2_00027298+TCONS_ l2_00026429+TCONS_l2_00026430+TCONS_ l2_00026431+TCONS_l2_00026432+TCONS_ l2_00026433+TCONS_l2_00026434+TCONS_ l2_00026435+TCONS_l2_00027299+TCONS_l2_00026436	−
chr2	38053390	38103451	TCONS_00002749+TCONS_00004206+TCONS_00003251 +TCONS_00003252+TCONS_00004207	−
chr21	44071701	44072723	TCONS_00029153	−
chr8	71383356	71397892	TCONS_00014749+TCONS_00015293	+
chr8	144161722	144164901	TCONS_l2_00028011	+
chr7	17105864	17133395	TCONS_00013360	+
chr8	9112315	9117805	TCONS_00014927+TCONS_00015406+TCONS_00014928	−
chr2	193508828	193510113	TCONS_00003471+TCONS_00003472	−
chr7	114719012	114766368	TCONS_l2_00027197+TCONS_l2_00027198+TCONS_ l2_00027199+TCONS_l2_00027200+TCONS_l2_00026163	+
chr11	130642666	130646675	TCONS_00019805	−
chr9	43066677	43089866	TCONS_l2_00029937+TCONS_l2_00029284+TCONS_ l2_00029938+TCONS_l2_00029285	−
chr4	23236248	23287196	TCONS_00007727	−
chr15	78181465	78191653	TCONS_l2_00008748+TCONS_l2_00008749+TCONS_ l2_00008750+TCONS_l2_00008751	+
chr11	90899912	90926626	TCONS_00019433+TCONS_00019967	+
chr3	66024757	66027673	TCONS_00006076+TCONS_00006078+TCONS_00006077	+
chr13	109962483	109964121	TCONS_00021595	+

chr4	111923366	111935138	TCONS_00008548	−
chr6	115793684	115807570	TCONS_00011935	+
chr2	128225150	128225676	TCONS_00003865	+
chr3	167584288	167588765	TCONS_00005879	−
chr21	45610322	45614187	TCONS_00029174	−
chr5	40052393	40053426	TCONS_00009388	+
chr15	93013469	93014805	TCONS_00023786+TCONS_00023787	−
chr3	39244496	39252892	TCONS_00005991+TCONS_00005992	+
chr21	11169666	11172046	TCONS_00028965	+
chr3	129612714	129627755	LOC100507032	+
chr6	145469832	145475587	TCONS_00011982	+
chr10	7583583	7589521	TCONS_00018118	+
chr5	42950963	42960649	TCONS_00009934	+
chr5	54913807	54916463	TCONS_00010338	−
chr3	149814230	149931513	TCONS_l2_00019929+TCONS_l2_00019930	+
chr22	44708869	44710872	TCONS_00029612	+
chr19	55219601	55224887	LILRP2	+
chr3	74955611	74956762	TCONS_00006097	+
chr11	103655495	103720223	TCONS_00019742+TCONS_00020125+TCONS_00020124 +TCONS_00019743+TCONS_00019744	−
chrX	56098009	56231479	TCONS_00017180	+
chr9	113361755	113367499	TCONS_00015735	+
chr3	182501024	182511182	TCONS_00005902+TCONS_00007318	−
chr9	7925578	7961234	TCONS_00015917	+
chr7	150040315	150055006	TCONS_00013626+TCONS_00013627+TCONS_00013628	+
chr6	24796945	24798091	TCONS_00011739	+
chr4	145772724	145795938	TCONS_00007406+TCONS_00008275	+
chr2	217081612	217084915	PKI55	+
chr1	59760542	59762053	TCONS_00001507+TCONS_00001508+TCONS_00001509	−
chr2	106209554	106227016	LOC285000	−
chr16	47883225	47921041	TCONS_00024268	−
chr9	68810281	68811704	TCONS_l2_00028787	+
chr8	97379635	97398645	TCONS_00015109	−
chr5	178191864	178203277	AACSP1	−
chr3	184119380	184168918	TCONS_00007050+TCONS_00006330+TCONS_00007051	+
chr2	104913938	104991859	TCONS_00004357+TCONS_00004358+TCONS_00004359	−
chr3	78136555	78141421	TCONS_00005545	+
chr18	33503683	33509979	TCONS_00026320	+
chr6	132223103	132241705	TCONS_l2_00025237+TCONS_l2_00025238+TCONS_ l2_00025239	+
chr2	85938746	85943145	TCONS_00004835	+
chr2	7561392	7590381	LOC100506274	+
chr14	74939815	74941018	TCONS_00022797	−
chr6	41137779	41145579	TCONS_00012168	−
chr11	70996105	70998121	TCONS_00019373	+
chr9	3684076	3689626	TCONS_00015789	−
chr14	59592025	59596263	TCONS_00022524	+

chr7	65958693	65960583	TCONS_00013488+TCONS_00013490+TCONS_00014154+TCONS_00014155	+
chr17	20279090	20279890	TCONS_l2_00011133	−
chr7	3179828	3214287	TCONS_00013677+TCONS_00012898	−
chr5	73623820	73630591	TCONS_00009701	−
chr2	131437623	131443435	CYP4F30P	+
chr21	23305635	23348000	TCONS_00028883	−
chr4	177796856	177801759	TCONS_00007687	+
chr2	47043807	47049799	LOC388948	+
chrX	38080644	38082920	TCONS_00016976	+
chr9	6704179	6707780	TCONS_00016532+TCONS_00015647	+
chr18	73931073	73933944	TCONS_00026372+TCONS_00026646+TCONS_00026647	+
chr8	47752508	47767407	LINC00293	+
chr15	79709125	79709673	TCONS_00023478	+
chr2	241320860	241327143	TCONS_00005360+TCONS_00004629	−
chr4	4763532	4789101	TCONS_00007381	+
chr13	19702683	19705456	TCONS_l2_00006694	+
chr1	233860045	233866505	TCONS_00001331+TCONS_00002260	+
chr10	116579688	116581199	TCONS_00019032	−
chr3	181839672	181859925	TCONS_00005673	+
chr9	89247062	89311411	TCONS_00015703	+
chr5	157929677	157942094	TCONS_00011076	−
chr13	46295094	46295826	TCONS_00021781	+
chr13	31623868	31630980	TCONS_00021963+TCONS_00021964+TCONS_00021965	−
chr15	70863298	70878945	TCONS_00023282	−
chr2	177708259	177711096	TCONS_00003950	+
chr7	46385371	46389981	TCONS_00013438	+
chr3	9640112	9641370	TCONS_00006435	−
chr10	70304127	70304647	TCONS_l2_00003602	−
chr7	121283388	121290414	TCONS_00013246	−
chr3	63132059	63134472	TCONS_00006041	+
chr10	4376602	4379400	TCONS_00018093	+
chr16	49477429	49487197	TCONS_00024637	−
chr12	40534259	40552325	TCONS_00020760+TCONS_00020761	−
chr2	240417595	240418376	TCONS_00004626	−
chr6	88997483	89130752	TCONS_00012221+TCONS_00011526	−
chr6	147180317	147184217	TCONS_00011392	+
chr18	5847053	5851746	TCONS_00026269	+
chr7	154795143	154797413	LOC202781	+
chr5	3345498	3346444	TCONS_00009849	+
chr6	37468257	37470210	TCONS_00011801	+
chr10	75540423	75541627	TCONS_00018537	−
chr10	15226942	15232617	TCONS_00018445	−
chr15	24646607	24656639	TCONS_00023311	+
chr6	66844838	66886208	TCONS_00011849	+
chr8	49221910	49222460	TCONS_00014999	−
chr14	45737887	45738830	TCONS_00022491	+
chr6	107150737	107167451	TCONS_00011913	+

chr15	80637197	80640120	TCONS_00023754+TCONS_00024185	−
chrX	48193110	48193874	TCONS_00017057	−
chr2	31046573	31083082	TCONS_00003624+TCONS_00003625+TCONS_00004741	+
chr6	50555320	50604918	TCONS_00011500+TCONS_00011501	−
chr2	21830528	21872435	TCONS_l2_00014468+TCONS_l2_00014469+TCONS_l2_00015738+TCONS_l2_00015739+TCONS_l2_00015740+TCONS_l2_00014470	−
chr2	89108797	89116314	TCONS_00004841+TCONS_00003762+TCONS_00004842+TCONS_00004843	+
chr1	142697421	142713605	ANKRD20A12P	−
chr5	180296862	180298361	TCONS_00010217	+
chr12	52233847	52234850	TCONS_00020785	−
chr1	209145799	209148652	TCONS_00000376	+
chr18	15301185	15304781	TCONS_00026681+TCONS_00026436	−
chr11	118935018	118938411	TCONS_00019777	−
chr17	4063018	4063996	TCONS_00025805+TCONS_00025806+TCONS_00025807	+
chr7	47009300	47067079	TCONS_l2_00027111+TCONS_l2_00027112+TCONS_l2_00025862	+
chr12	72647287	72667289	TRHDE-AS1	−
chr2	64412213	64432619	LINC00309	−
chr1	91248095	91317317	TCONS_00001579+TCONS_00001580+TCONS_00001581+TCONS_00000088+TCONS_00000089+TCONS_00000563+TCONS_00001582	−
chr4	63316228	63320355	TCONS_00008495	−
chr13	64410945	64416252	TCONS_l2_00006891+TCONS_l2_00006892	+
chr4	83206177	83218667	TCONS_00008138	+
chr1	420206	421839	TCONS_l2_00001928	+
chr22	18660805	18688682	TCONS_l2_00018306+TCONS_l2_00018307	−
chr3	98699902	98701940	TCONS_00005559	+
chr18	59246664	59274234	TCONS_00026538+TCONS_00026539+TCONS_00026540	−
chr6	1605724	1607305	TCONS_00011636	+
chr3	143753460	143755742	TCONS_00006254	+
chr10	81629875	81634783	TCONS_l2_00003650+TCONS_l2_00003651	−
chr13	112810023	112814358	TCONS_00022118	−
chr20	2644998	2646520	TCONS_00027896	+
chr6	143845464	143855908	TCONS_00011980	+
chr13	78849855	78851649	TCONS_00021673	−
chr8	65919160	65930062	TCONS_00014741+TCONS_00014742	+
chr13	40637747	40638543	TCONS_00021761+TCONS_00022157	+
chr7	115319400	115322672	TCONS_00012923	−
chr7	63484448	63487380	TCONS_00013473	+
chr2	169281145	169285565	TCONS_00004489+TCONS_00005270+TCONS_00004490	−
chr8	90597666	90659991	TCONS_l2_00028288+TCONS_l2_00028289+TCONS_l2_00028290+TCONS_l2_00028291+TCONS_l2_00028293+TCONS_l2_00028294+TCONS_l2_00028295	−
chr2	38658396	38673752	TCONS_00003641+TCONS_00002875	+
chr13	85455734	85466453	TCONS_00022072+TCONS_00022073	−
chr16	2708390	2723440	ERVK13-1	−
chr4	152840620	152865085	TCONS_00007660	+

chr4	22328990	22341289	LOC100505912	−
chr1	116783461	116784684	TCONS_00001636	−
chr21	45627833	45629968	TCONS_00029050	+
chr5	123676150	123676863	TCONS_00009771	−
chr22	20401139	20420591	TCONS_00029515	+
chr18	37235582	37245528	TCONS_00026613+TCONS_00026614+TCONS_00026615	+
chr2	13440811	13471719	TCONS_00003583	+
chr1	120615908	120619716	TCONS_00001648	−
chr10	103464409	103468158	TCONS_00018034	−
chr22	20291260	20300571	TCONS_l2_00017600+TCONS_l2_00018152+TCONS_l2_00017601+TCONS_l2_00017602	+
chr22	23974936	23978176	TCONS_00029658	−
chr4	174845360	174912012	TCONS_00007446+TCONS_00007901	−
chr11	116367616	116371347	TCONS_00019174	−
chr17	60363989	60374387	TCONS_l2_00011393+TCONS_l2_00011392+TCONS_l2_00011394+TCONS_l2_00011395	−
chr21	44776024	44777849	TCONS_00029045	+
chr18	24386465	24402547	TCONS_00026303	+
chr9	131097666	131100554	TCONS_00016481	−
chr6	37301809	37307755	TCONS_00012157	−
chr1	149691432	149720868	TCONS_l2_00002663+TCONS_l2_00001518+TCONS_l2_00001519+TCONS_l2_00001520+TCONS_l2_00001521	−
chr13	97798987	97799773	TCONS_00021876	+
chr9	36313544	36315318	TCONS_00016312	−
chr3	180604459	180628232	TCONS_l2_00019632	−
chr6	5030206	5044060	TCONS_00011154+TCONS_00011155+TCONS_00011660+TCONS_00011661	+
chrX	40936206	40937659	TCONS_l2_00030183	+
chr1	11432762	11438744	TCONS_00000839	+
chr7	62441009	62453054	TCONS_00013823	−
chr7	128552675	128553989	TCONS_l2_00026884	−
chr16	30278914	30346695	LOC595101	−
chr1	61125303	61291256	TCONS_00000081	−
chr1	236260402	236261074	TCONS_00002263+TCONS_00002264+TCONS_00002265+TCONS_00002266	+
chr9	66544445	66545920	TCONS_l2_00028762	+
chr3	139407245	139408184	TCONS_00005838	−
chr9	75838386	75839103	TCONS_00016356	−
chr12	96983231	96988177	TCONS_l2_00005840+TCONS_l2_00005841	+
chr15	28899588	28930410	HERC2P9	+
chr13	19774845	19820881	TCONS_l2_00007052+TCONS_l2_00007053+TCONS_l2_00007054+TCONS_l2_00007055	−
chr5	109243259	109247966	TCONS_00010056	+
chr8	84315993	84321130	TCONS_00014497	+
chr17	66805273	66820947	TCONS_00025742	−
chr1	166542930	166543981	TCONS_l2_00000673	+
chr10	3730918	3731751	TCONS_00018872+TCONS_00018873	−
chr7	63385165	63386655	TCONS_l2_00027378+TCONS_l2_00027379	−
chr14	82278093	82284567	TCONS_00022817	−

chr18	3462264	3480436	TCONS_00026396	−
chr7	19958603	20171342	TCONS_l2_00026409+TCONS_l2_00027285+TCONS_l2_00027286+TCONS_l2_00026410+TCONS_l2_00026411+TCONS_l2_00027287+TCONS_l2_00027288+TCONS_l2_00027289+TCONS_l2_00026412	−
chr1	161526080	161526868	TCONS_00000652	−
chr17	6840594	6857728	TCONS_00025293	+
chr12	119196362	119199469	TCONS_00020945+TCONS_00020944	−
chr20	25121434	25129426	LOC284798	−
chr17	2458604	2472677	TCONS_00025270+TCONS_00025271	+
chr20	43766347	43767617	TCONS_00028402	−
chr3	158487101	158502276	TCONS_l2_00018962	+
chr16	11536047	11585611	TCONS_l2_00010447+TCONS_l2_00010003	−
chr4	189717668	189718050	TCONS_00008980	+
chr5	117803851	117897818	TCONS_00009759+TCONS_00010445+TCONS_00011021 +TCONS_00010447+TCONS_00009760	−
chr1	168893336	168908348	TCONS_l2_00000681	+
chr11	79992036	80007893	TCONS_00019406	+
chr3	149919961	149942977	TCONS_00005846	−
chr16	68613486	68614640	TCONS_00024443	+
chr16	34327007	34329672	TCONS_l2_00009703	+
chr7	3133765	3157650	TCONS_00014249+TCONS_00013675+TCONS_00013676	−
chr10	133664310	133665914	TCONS_00018648	−
chr17	48870667	48872202	TCONS_00025429	+
chr3	57678932	57682323	TCONS_00005522	+
chr10	99582228	99601058	TCONS_00018295	+
chr10	49649950	49652001	TCONS_00018200	+
chr17	623631	624327	TCONS_00025799	+
chr15	48612328	48619538	TCONS_00023935	+
chr2	106998570	107007851	PLGLA	+
chr8	125205393	125220364	TCONS_00015161	−
chr4	163661820	163813536	TCONS_00007672+TCONS_00008309	+
chr5	16428645	16432545	TCONS_00009356	+
chr5	5688511	5701975	TCONS_00009856+TCONS_00009857+TCONS_00010610	+
chr13	21834168	21869577	TCONS_l2_00006714+TCONS_l2_00007358+TCONS_l2_00007359+TCONS_l2_00006715+TCONS_l2_00006716	+
chr7	148321791	148322542	TCONS_00013994	−
chr22	27600173	27601088	TCONS_00029400	+
chr4	189080968	189082293	TCONS_00008978+TCONS_00008378+TCONS_00007698	+
chr8	117616759	117627525	TCONS_00014837+TCONS_00014838	+
chr18	26582871	26584888	TCONS_00026308	+
chr1	16510023	16510657	TCONS_00000846	+
chr16	52656380	52660131	TCONS_00024419	+
chr1	13997965	14001403	TCONS_00000841	+
chr4	126991863	126993159	TCONS_00007614	+
chr1	44410042	44412223	TCONS_00000510+TCONS_00000511+TCONS_00000512	−
chr4	32226645	32230663	TCONS_00008034+TCONS_00008035	+
chr5	127999671	128078522	TCONS_00009508	+

chr10	10429716	10504510	TCONS_00018432+TCONS_00018433+TCONS_00018881+TCONS_00017926	−
chr9	98527280	98538745	TCONS_00016085+TCONS_00016656	+
chr5	74343544	74348668	TCONS_00009703+TCONS_00009704	−
chrX	281385	282054	LINC00685	+
chr13	89867101	89889144	TCONS_00021680+TCONS_00021681	−
chr7	46727477	46736720	TCONS_00012907	−
chr9	26716406	26718917	TCONS_00016286	−
chr2	111203460	111230652	LIMS3L	−
chr2	33825775	33881292	TCONS_l2_00013556+TCONS_l2_00013557+TCONS_l2_00013558+TCONS_l2_00013559	+
chrY	7302524	7388562	TCONS_00017630	+
chr22	44839207	44840668	TCONS_00029433	+
chr2	203175137	203177250	TCONS_00003990	+
chr3	187553660	187574106	TCONS_00006729	−
chr2	697153	714338	TCONS_00004095	−
chr3	134032418	134040013	TCONS_00005590	+
chr21	20649019	20656952	TCONS_00029086	−
chrY	24585737	24587584	TTTY6B	−
chr16	2390923	2476700	ABCA17P	+
chr19	20358336	20359682	TCONS_00027503	+
chr15	38360991	38365188	TCONS_00023630+TCONS_00023631+TCONS_00023245	−
chr12	120718115	120720608	TCONS_00020953	−
chr21	43459225	43475216	TCONS_00029031+TCONS_00029032	+
chr2	220603493	220607703	TCONS_00004031	+
chr1	101701239	101702084	TCONS_00000589	−
chr15	30846790	30848689	TCONS_l2_00008528+TCONS_l2_00008529	+
chrX	22716574	22787083	TCONS_00016972	+
chrX	47157251	47158120	TCONS_00016985	+
chr9	129289897	129292622	TCONS_00015754	+
chr7	153120803	153125424	TCONS_00014010	−
chr18	5209511	5214978	TCONS_00026245	+
chr13	84660710	84694677	TCONS_l2_00006958	+
chr5	95549050	95550779	TCONS_00009731	−
chr4	159178384	159202705	TCONS_l2_00020851+TCONS_l2_00021764+TCONS_l2_00020852	+
chr2	59662348	59669396	TCONS_00002900	+
chr18	24002982	24018001	TCONS_00026301+TCONS_00026302	+
chrY	231385	232054	LINC00685	+
chr22	17160318	17182301	TCONS_l2_00017861+TCONS_l2_00017862+TCONS_l2_00017863+TCONS_l2_00017864	−
chr3	192862253	192894653	TCONS_l2_00020300+TCONS_l2_00019664	−
chr8	88937964	88938489	TCONS_00015067	−
chrY	25537324	25538842	TCONS_l2_00030912	+
chr13	88453291	88456063	TCONS_00021582	+
chrY	900956	905100	TCONS_00017600	+
chr2	75146682	75151973	TCONS_00003323	−
chr18	14221252	14226965	TCONS_l2_00011988	−

chr6	26924772	26991753	LINC00240	+
chr20	18040137	18051652	TCONS_00027906	+
chr7	6992469	6993674	TCONS_00013687	−
chr14	19604880	19639483	TCONS_l2_00007611+TCONS_l2_00007612+TCONS_l2_00008288	+
chr2	121839013	121846302	TCONS_00003394	−
chr1	236120927	236138865	TCONS_00001333	+
chr12	74526956	74686411	LOC100507377	−
chr6	40239305	40244055	TCONS_00011485+TCONS_00012163	−
chr1	17516278	17524112	TCONS_00000159	+
chr11	59927128	59927785	TCONS_l2_00005025	−
chr11	65265233	65273939	MALAT1	+
chr20	57935982	57939828	TCONS_00028079	−
chr15	38276923	38352476	TCONS_00023351+TCONS_00023352+TCONS_00023353+TCONS_00023220+TCONS_00023915+TCONS_00023354+TCONS_00023355+TCONS_00023356	+
chr2	104473428	104496667	TCONS_00003346+TCONS_00003347	−
chr4	59912738	59940960	TCONS_00007770	−
chr8	124009622	124012419	TCONS_00015156	−
chr9	68324611	68340644	TCONS_l2_00029757	+
chr11	119600109	119610354	TCONS_00019495+TCONS_00019496+TCONS_00019497+TCONS_00019498	+
chr5	32925745	33298016	TCONS_l2_00023703+TCONS_00009631+TCONS_l2_00023705+TCONS_l2_00023706	−
chr2	88927347	88931337	TCONS_00002711	+
chr17	50995288	51004254	TCONS_00025434	+
chr7	142927260	142928714	TCONS_l2_00026251	+
chr6	49644372	49646502	TCONS_00012742	−
chr17	74116460	74117525	TCONS_00025770	−
chr8	1263347	1267655	TCONS_00014573+TCONS_00014574	+
chr20	22255046	22263310	TCONS_00027883+TCONS_00028017	−
chr2	46909764	46910666	TCONS_00004246	−
chr7	63174051	63175663	TCONS_00013179	−
chr11	46235389	46241181	TCONS_00019634	−
chr3	151256968	151318744	TCONS_00006684	−
chr6	142572984	142580769	TCONS_00011578	−
chr6	165241239	165243106	TCONS_00011411	+
chr3	12272665	12275746	TCONS_00006442	−
chr12	112277573	112280706	MAPKAPK5-AS1	−
chr6	168067524	168079903	TCONS_l2_00025541+TCONS_l2_00025034	−
chr5	178882416	178883096	TCONS_00009558	+
chr10	10407143	10408450	TCONS_00017925	−
chr3	42018710	42019290	TCONS_l2_00018516	+
chr1	75428998	75430802	TCONS_00000234	+
chr5	101836245	101841374	TCONS_00009473+TCONS_00009474	+
chr5	10330546	10332338	TCONS_00009873	+
chr11	21804219	21864624	TCONS_00019253+TCONS_00019254+TCONS_00019255	+
chr8	81806189	81864554	TCONS_00014542	+

chr5	107728769	107753700	TCONS_00010053	+
chr3	128957824	128966677	TCONS_00006224	+
chrX	118706845	118707686	TCONS_00017234	+
chr1	117315570	117325219	TCONS_l2_00001462+TCONS_l2_00001463	−
chr20	46584976	46589679	TCONS_00028557+TCONS_00027869+TCONS_00027939	+
chr14	36741574	36759236	TCONS_00022470	+
chr13	19408543	19446109	ANKRD20A9P	−
chr1	220658446	220660385	TCONS_00000736	−
chr1	143687130	143714180	LOC100130000	+
chr2	169197717	169200862	TCONS_00005268+TCONS_00004488+TCONS_00005269	−
chr13	53263722	53274883	TCONS_00021656	−
chr9	86188943	86190093	TCONS_00016378	−
chr10	109136308	109160994	TCONS_l2_00004033+TCONS_l2_00003238	+
chr1	106508402	106581195	TCONS_00001084	+
chr4	6305743	6309206	TCONS_00008415	−
chr4	111524517	111536571	TCONS_00007813	−
chr17	14934292	14935274	CDRT7	+
chr7	148240641	148265340	TCONS_00013993	−
chr18	52558108	52565201	TCONS_00026523	−
chr13	24905588	24911256	TCONS_00021481	+
chr21	23095613	23109639	LINC00317	−
chr13	115095034	115099423	TCONS_00021703	−
chr14	30637041	30766245	TCONS_00022407	−
chr15	98624860	98646939	TCONS_00023827+TCONS_00023826+TCONS_000 24214+TCONS_00024215+TCONS_00024216+TCO NS_00024217	−
chr2	77899341	77900918	TCONS_00002940	+
chr13	89193084	89197764	LINC00433	+
chr13	43423874	43437077	TCONS_00021981	−
chr6	110147725	110149988	TCONS_00011354	+
chr7	63575029	63611321	TCONS_l2_00025933+TCONS_l2_00027128	+
chr5	50225318	50230355	TCONS_00009659	−
chr20	10154168	10177638	TCONS_00027900+TCONS_00027901	+
chr13	105599344	105607681	TCONS_00022101	−
chr7	100254184	100264332	TCONS_00012886	+
chr10	3026556	3027331	TCONS_00017762	+
chr4	176366445	176372576	TCONS_00008664	−
chr14	98602415	98628990	TCONS_00022624	+
chr4	170952929	170954179	TCONS_00007445	−
chr19	24014400	24053826	TCONS_l2_00012428+TCONS_l2_00012429	+
chr1	207558529	207560344	TCONS_00001252	+
chr9	6645889	6670845	TCONS_l2_00028610+TCONS_l2_00028611+TCONS_ l2_00028612	+
chrX	134540021	134540794	TCONS_00016940	+
chr22	32517964	32529456	AP1B1P1	+
chr5	24880280	24882020	TCONS_00009907	+
chr9	68743531	68769869	TCONS_l2_00029973	−
chr3	171594142	171618530	TCONS_00005881	−

chr22	16162066	16172265	LOC101101776	+
chr16	56073890	56111327	TCONS_00024672+TCONS_00024673	−
chr13	55526438	55560817	TCONS_00021799	+
chr21	45870869	45875167	LRRC3-AS1	−
chr3	30560407	30568677	TCONS_l2_00018490+TCONS_l2_00018491+TCONS_l2_00019777	+
chr17	45940708	45942555	TCONS_00025121	+
chr6	114798514	114809916	TCONS_00011546+TCONS_00011547	−
chr2	79362629	79365553	REG1P	−
chr20	54036874	54043735	TCONS_00028069	−
chr5	102001911	102058026	TCONS_00010050+TCONS_00010755+TCONS_00010756	+
chr15	24426315	24445661	TCONS_00023304+TCONS_00023858	+
chr2	20251895	20254219	TCONS_00003222	−
chr21	19986749	20022678	TCONS_00028800	+
chr4	43765515	43768276	TCONS_00008060	+
chrX	106769825	106848480	TCONS_l2_00030321+TCONS_l2_00030322+TCONS_l2_00030323+TCONS_l2_00030324+TCONS_l2_00030325	+
chr9	66720898	66759372	TCONS_00015683+TCONS_00015684	+
chr14	102414635	102419663	TCONS_00022885+TCONS_00022884	−
chrY	2481029	2483388	TCONS_00017619	−
chr11	45746268	45749273	TCONS_00019631	−
chr7	63498206	63500337	TCONS_00013474	+
chr2	208527099	208543837	TCONS_l2_00015589+TCONS_l2_00014208+TCONS_l2_00014209+TCONS_l2_00014210	+
chr3	117376776	117381146	TCONS_00006167	+
chr7	56184206	56186864	TCONS_00014135	+
chr8	43341886	43349897	TCONS_l2_00027738	+
chr1	247444093	247453811	TCONS_l2_00000943+TCONS_l2_00000944	+
chr20	25982172	25982935	TCONS_00028032	−
chr5	17379015	17387419	LOC401177	−
chr12	115525789	115527098	TCONS_00020935	−
chr10	47270681	47273431	TCONS_l2_00004174	−
chr13	100738209	100741196	TCONS_00022099	−
chr6	90629915	90631479	TCONS_00011337	+
chr10	46778828	46790587	TCONS_l2_00003037	+
chr15	84868830	85748518	LOC642423	−
chr12	56849484	56850013	TCONS_l2_00006273	−
chr13	32420920	32533721	EEF1DP3	+
chr14	85995123	85995625	TCONS_00023024	+
chr11	104772276	104788902	LOC643733	−
chr7	66367520	66371249	TCONS_l2_00026666	−
chrX	119055224	119056803	TCONS_00017085	−
chr5	76369706	76370345	TCONS_00009428	+
chr16	54952777	54963101	CRNDE	−
chr8	144163842	144164901	TCONS_00015540	−
chr3	195725735	195731964	TCONS_00007353	−
chr9	76060541	76092053	TCONS_l2_00028842+TCONS_l2_00028843+TCONS_l2_00028844	+

chr4	153067537	153099725	TCONS_00007881	−
chr5	7272908	7277834	TCONS_00010614+TCONS_00009861+TCONS_00009862	+
chr14	98391947	98444461	C14orf64	−
chr3	190415712	190416637	TCONS_00005914	−
chr22	35024641	35034250	TCONS_00029584	+
chr10	11495945	11497386	TCONS_00017789	+
chr2	20786003	20793321	TCONS_l2_00014464+TCONS_l2_00014463+TCONS_l2_00014462+TCONS_l2_00015737+TCONS_l2_00014465	−
chr21	30565815	30660526	LINC00189	+
chr20	59652280	59652872	TCONS_00028080	−
chr2	47922886	47984932	TCONS_l2_00013621+TCONS_l2_00013622+TCONS_l2_00015325	+
chr2	220782592	220878593	TCONS_00004033	+
chr9	27829274	27844479	TCONS_00015573	+
chr4	26784349	26784724	TCONS_00008016	+
chr10	21462919	21463852	NEBL-AS1	+
chr17	77893156	77899334	TCONS_00025131+TCONS_00025523	+
chr12	9516719	9519991	TCONS_00020710	−
chrX	3114327	3118067	TCONS_00017130	+
chr10	54730759	54790030	TCONS_00018517+TCONS_00018518+TCONS_00018936+TCONS_00017983+TCONS_00018937+TCONS_00018520+TCONS_00018519	−
chr4	84009930	84010394	TCONS_00008143+TCONS_00008144	+
chr8	61234943	61245535	TCONS_00015018	−
chr10	9317796	9329020	TCONS_00017787	+
chr19	54102885	54106751	LOC284379	−
chr18	36155580	36157625	TCONS_00026494	−
chr4	76326405	76348233	TCONS_00007552+TCONS_00007553	+
chr1	221681901	221684321	TCONS_l2_00002307	+
chrY	27209230	27246039	TTTY4	−
chrX	57244100	57251237	TCONS_l2_00030703+TCONS_l2_00030228+TCONS_l2_00030229+TCONS_l2_00030230	+
chr2	124200842	124202021	TCONS_00003396	−
chr22	16101370	16193226	TCONS_l2_00017847+TCONS_l2_00018290+TCONS_l2_00018291+TCONS_l2_00018292+TCONS_l2_00017848+TCONS_l2_00017849+TCONS_l2_00017850+TCONS_l2_00018293+TCONS_l2_00018294+TCONS_l2_00017851+TCONS_l2_00017852+TCONS_l2_00018295+TCONS_l2_00018296+TCONS_l2_00017853	−
chr16	70236207	70237631	TCONS_l2_00009811	+
chr1	89500494	89501303	TCONS_00001017	+
chr11	30381557	30383421	TCONS_00019600	−
chr4	160319685	160322398	TCONS_00007670	+
chr2	105719308	105720606	TCONS_00004363	−
chr16	61501281	61502791	TCONS_00024429	+
chr5	44753051	44765846	TCONS_00009394	+
chr6	25061840	25063735	TCONS_00012109+TCONS_00011470	−
chr17_ctg5_hap1	1320861	1323954	TCONS_00030024	−

chr4	176267509	176324261	TCONS_00007906	−
chr13	63886242	63902270	TCONS_00022040+TCONS_00022290	−
chr7	68652820	68654305	TCONS_00013190	−
chr10	2229858	2231694	TCONS_00018399	−
chr9	11275314	11276314	TCONS_00015793	−
chr9	2919099	2949837	TCONS_l2_00029895+TCONS_l2_00029182+TCONS_ l2_00029896	−
chr13	31945228	31947414	TCONS_00021966	−
chr5	9854489	9890049	TCONS_00009350+TCONS_00009872+TCONS_00010622	+
chr5	72747323	72749220	TCONS_00009424	+
chr7	90940673	91144724	TCONS_l2_00026091+TCONS_l2_00027168+TCONS_ l2_00027169+TCONS_l2_00027170+TCONS_l2_00026092	+
chr14	101154891	101156618	TCONS_00022630	+
chr17	79759212	79761593	TCONS_00025782	−
chr7	15105832	15108276	TCONS_00012957	+
chr20	19189995	19190824	TCONS_00028327	−
chr11	116510189	116528970	TCONS_l2_00004778+TCONS_l2_00005336+TCONS_ l2_00004779+TCONS_l2_00004780+TCONS_l2_00004781	+
chr10	43979977	44021239	TCONS_l2_00003011+TCONS_l2_00003012	+
chr12	126650384	126656211	TCONS_00020628+TCONS_00021249+TCONS_00020 630+TCONS_00021248+TCONS_00021247+TCONS_0 0021246+TCONS_00021245+TCONS_00020629+TCO NS_00021250	+
chr10	31982012	31996316	TCONS_00017946	−
chr3	172278678	172313397	TCONS_00005882+TCONS_00005883+TCONS_00005475 +TCONS_00006708	−
chr1	242717038	242718022	TCONS_00001896	−
chr11	61740186	61744174	TCONS_00019645	−
chr14	45552254	45553180	TCONS_00022408+TCONS_00023103+TCONS_00022736 +TCONS_00022366	−
chr3	180465938	180528245	TCONS_l2_00019630	−
chr22	39688993	39691568	TCONS_00029426	+
chr21	15018961	15032639	TCONS_l2_00016969	+
chr10	90789621	90792766	TCONS_00018268	+
chr9	7782483	7788763	TCONS_00016265+TCONS_00016266	−
chr1	106432383	106434018	TCONS_l2_00001429	−
chr2	27938769	27980489	TCONS_00003611+TCONS_00004725+TCONS_00003612 +TCONS_00003614+TCONS_00004726+TCONS_0000361 5+TCONS_00003616+TCONS_00003617	+
chr4	27135618	27141673	TCONS_00007734	−
chr4	10739199	10751216	TCONS_00007982+TCONS_00007983+TCONS_00007984 +TCONS_00008752	+
chr9	7924794	7926107	TCONS_00016267	−
chr9	10096	10799	TCONS_00016239	−
chr2	28671034	28673051	TCONS_00003240	−
chr21	47472510	47473019	TCONS_00028863	+
chr8	26234088	26240466	TCONS_l2_00028127+TCONS_l2_00028128	−
chr6	36807550	36816400	TCONS_00011214+TCONS_00012156	−
chr18	53763146	53763519	TCONS_00026624	+
chr6	82981004	82982268	TCONS_00011877	+

chr4	6689175	6692251	TCONS_00007713+TCONS_00009023	−
chr3	147561910	147564444	TCONS_00005841	−
chr10	28615100	28618930	TCONS_00018462	−
chr5	33987091	34124633	C1QTNF3-AMACR	−
chr4	184254595	184271991	TCONS_00008682+TCONS_00009184+TCONS_00009185	−
chr6	6694900	6745648	TCONS_00012067+TCONS_00012068+TCONS_00011442+TCONS_00012069+TCONS_00012070+TCONS_00012071+TCONS_00012649+TCONS_00012072	−
chr17	20478827	20482234	TCONS_00026046+TCONS_00025588+TCONS_00025589+TCONS_00026047	−
chr2	79107908	79108749	TCONS_00004317	−
chr9	23895025	23898182	TCONS_l2_00029225	−
chr17	70026957	70035822	TCONS_00025128	+
chr2	16405315	16410408	TCONS_00002834	+
chr8	34938071	34951299	TCONS_00014672+TCONS_00015270	+
chr7	154999560	155001321	TCONS_00014013+TCONS_00014014+TCONS_00014015	−
chr12	3409393	3410207	TCONS_00020676	−
chr2	129622179	129626301	TCONS_00002721	+
chr6	170361247	170367494	TCONS_00012631	+
chr4	139198269	139207245	TCONS_00007853+TCONS_00008588	−
chr11	38668074	38688323	TCONS_00019276	+
chr10	123374939	123375353	TCONS_00018061	−
chr1	226271656	226278045	TCONS_l2_00002324+TCONS_l2_00000871+TCONS_l2_00002325	+
chr4	117410520	117436302	TCONS_00007816	−
chr4	59646791	59853878	TCONS_00007765+TCONS_00007766+TCONS_00008494+TCONS_00007767+TCONS_00009063+TCONS_00009062+TCONS_00009061+TCONS_00007768+TCONS_00007769	−
chr11	61355966	61375017	TCONS_00019096+TCONS_00019910+TCONS_00019143	+
chr1	164513991	164515524	TCONS_00001709	−
chr6	150361683	150363169	TCONS_00011594	−
chr10	125115957	125315947	TCONS_00017720+TCONS_00018366+TCONS_00018367+TCONS_00018368+TCONS_00017884+TCONS_00018369+TCONS_00018846+TCONS_00018845+TCONS_00018844+TCONS_00018370+TCONS_00018371	+
chr5	141615466	141616011	TCONS_00010119	+
chr3	94224395	94226462	TCONS_l2_00019381	−
chr2	217731056	217732536	TCONS_00002776	−
chr6	7427116	7453025	TCONS_00011672+TCONS_00011673+TCONS_00011674+TCONS_00012412+TCONS_00011675+TCONS_00012413	+
chr7	141940630	141943268	TCONS_l2_00027230	+
chr4	9699870	9700981	TCONS_l2_00020406	+
chrX	152864823	152869126	TCONS_00017274	+
chr22	29712789	29715727	TCONS_00029887	−
chr4	88183083	88187974	TCONS_00007791	−
chr10	52486382	52487593	TCONS_l2_00003579	−
chr12	76083364	76084611	TCONS_00020847	−
chr2	101338683	101339344	TCONS_00003341	−
chr14	29620347	29636919	TCONS_00022456	+

chr1	208844668	208901923	TCONS_l2_00001737+TCONS_l2_00001738	−
chr4	156436879	156438611	TCONS_00007665	+
chr1	59180600	59184660	TCONS_00000959+TCONS_00000960	+
chr9	23829671	23849912	TCONS_00015656	+
chr19	7420502	7441585	TCONS_l2_00012260+TCONS_l2_00012261	+
chr1	232853817	232862099	TCONS_00000419+TCONS_00001327	+
chr6	25997715	26006496	TCONS_00012113+TCONS_00012114	−
chr14	80931126	80938379	TCONS_00022565	+
chr8	82756446	82761791	TCONS_00014788	+
chr9	33730109	33749410	TCONS_00015948+TCONS_00016564+TCONS_00016565 +TCONS_00016566+TCONS_00016567	+
chr1	60880143	60881834	TCONS_00000967	+
chr13	93685239	93710179	TCONS_00021872+TCONS_00021873+TCONS_00021586	+
chr3	184096018	184097565	TCONS_00007049	+
chr5	1042043	1042914	TCONS_00009829	+
chr4	58292038	58332152	TCONS_00007386+TCONS_00008087+TCONS_00008796	+
chr5	65500952	65505274	TCONS_00009284	−
chr7	1659120	1682389	TCONS_00013315+TCONS_00014064+TCONS_00013316 +TCONS_00012940	+
chr6	109072857	109091145	LINC00222	+
chrY	24795438	24800925	TCONS_l2_00031002	+
chr1	45762366	45771290	TCONS_00001482+TCONS_00000513	−
chr1	14362	29370	WASH7P	−
chr5	98264838	98266713	LOC100289230	+
chr4	105475208	105541543	TCONS_l2_00020665+TCONS_l2_00020666+TCONS_ l2_00021662	+
chr5	89240727	89243390	TCONS_00009458	+
chr9	132094579	132121817	TCONS_l2_00029859+TCONS_l2_00029055+TCONS_ l2_00029056+TCONS_l2_00029057+TCONS_ l2_00029059+TCONS_l2_00029860+TCONS_ l2_00029060+TCONS_l2_00029061+TCONS_ l2_00029062+TCONS_l2_00029063+TCONS_l2_00029861	+
chr1	4611795	4612205	TCONS_00000144	+
chr13	35009591	35214822	LINC00457	−
chr5	151329132	151650009	TCONS_00009263	+
chr2	177502438	177520707	TCONS_00003057+TCONS_00004950+TCONS_00003947 +TCONS_00004951	+
chr4	41752362	41759358	TCONS_00007520	+
chr4	9672577	9686917	TCONS_l2_00020405	+
chr17	34365072	34367984	TCONS_00025615	−
chr2	8231978	8232539	TCONS_l2_00015234	+
chr19	20317538	20349229	TCONS_l2_00012917+TCONS_00026840	−
chr11	125461043	125462350	TCONS_00019792	−
chr8	75015625	75019100	TCONS_00014763	+
chr6	3832062	3842054	TCONS_00012402+TCONS_00012403	+
chr3	65160633	65179179	TCONS_00005534+TCONS_00006074+TCONS_00006874	+
chr2	132722304	132737446	TCONS_l2_00015976	−
chr9	39395734	39397403	TCONS_00015808	−
chr8	22808336	22814153	TCONS_00014650	+

chr16	86254429	86338058	TCONS_00025083+TCONS_00025082+TCONS_00025081+TCONS_00024760+TCONS_00024761+TCONS_00024762+TCONS_00024763+TCONS_00025084+TCONS_00025085+TCONS_00024764+TCONS_00025086	−
chr2	25194981	25262563	DNAJC27-AS1	+
chr7	144084026	144088382	TCONS_00014225+TCONS_00014226+TCONS_00013616+TCONS_00013617	+
chr10	49255088	49258429	TCONS_l2_00003557	−
chr1	35606941	35611998	TCONS_00001452+TCONS_00001453+TCONS_00002334	−
chr17	20691697	20692707	TCONS_00025860	+
chr11	111065464	111068473	TCONS_00019756	−
chr12	7281675	7282622	TCONS_00021280+TCONS_00021281	−
chr4	172210626	172219418	TCONS_00007896	−
chr3	1049819	1054618	TCONS_00005488	+
chr16	20598010	20601912	TCONS_l2_00010036+TCONS_l2_00010037	−
chrX	27882114	27882507	TCONS_l2_00030169	+
chr8	122709844	123006304	TCONS_00014845+TCONS_00015336+TCONS_00014847+TCONS_00015338+TCONS_00015337+TCONS_00015339	+
chr7	123315994	123319730	TCONS_00013943	−
chr7	69054796	69063004	TCONS_00013495	+
chr19	28409545	28447647	TCONS_l2_00012438+TCONS_l2_00012439	+
chr15	59060273	59063173	TCONS_00023279	−
chr5	2917973	2935296	TCONS_00010605+TCONS_00009840+TCONS_00009841+TCONS_00009842+TCONS_00009843+TCONS_00010606+TCONS_00010607	+
chr6	82553501	82648074	TCONS_00012216+TCONS_00011524+TCONS_00012217	−
chr14	95805196	95806028	TCONS_00022843	−
chr13	96737151	96739285	TCONS_00022089	−
chr18	57059887	57065685	TCONS_00026535	−
chr3	55330228	55334766	TCONS_00005520	+
chr11	58002206	58006146	TCONS_00019639	−
chr17	75930833	75931431	TCONS_00025509	+
chr8	74005610	74006141	TCONS_00014758	+
chr5	133135104	133156483	TCONS_00010790+TCONS_00010095	+
chr7	134102145	134102761	TCONS_00013975	−
chr9	32738952	32745638	TCONS_00015943+TCONS_00016551	+
chr9	69746919	69757784	TCONS_l2_00029980	−
chr8	134420233	134421667	TCONS_00015178	−
chr7	96250969	96293650	LOC100506136	−
chr10	995367	998124	TCONS_00018082	+
chr15	102495088	102496558	FAM138E	+
chr13	106249802	106251893	TCONS_00022102	−
chr7	65235901	65267658	TCONS_l2_00025979+TCONS_l2_00025980+TCONS_l2_00025981+TCONS_l2_00025982+TCONS_l2_00025983	+
chr5	25219082	25298721	TCONS_00009622	−
chr16	27389363	27389821	TCONS_00024360	+
chr12	95318401	95321851	TCONS_00020884	−
chr4	175444852	175461495	TCONS_l2_00022059	−

chr8	142384280	142392326	TCONS_00014880	+
chr6	6758511	6759524	TCONS_00011443	−
chr8	28915363	28922445	TCONS_00015261+TCONS_00015262	+
chr10	8083775	8084837	TCONS_00018119+TCONS_00018120	+
chr4	34122516	34271369	TCONS_l2_00021855+TCONS_l2_00021099+TCONS_l2_00021100+TCONS_l2_00021856+TCONS_l2_00021101	−
chr1	56336186	56345756	TCONS_00000951+TCONS_00002039	+
chr14	99750984	99763570	TCONS_00023170+TCONS_00023171+TCONS_00022868+TCONS_00022869	−
chr14	50320358	50329571	TCONS_00022986+TCONS_00022987	+
chr7	150245546	150260952	TCONS_l2_00026976	−
chr11	69002987	69015856	TCONS_00019367	+
chr1	173991647	173992123	TCONS_00000337	+
chr5	176170145	176170860	TCONS_00010189+TCONS_00009551	+
chr2	83094016	83095308	TCONS_00004320	−
chr1	174966629	174968522	TCONS_00002518	−
chr4	66535679	66559104	LOC100144602	+
chr17	15721320	15767468	TCONS_l2_00010577	+
chr3	149768638	149769297	TCONS_l2_00018923	+
chr4	120720251	120725675	TCONS_00007818+TCONS_00007432+TCONS_00009115+TCONS_00008563+TCONS_00008564	−
chr7	133759093	133759805	TCONS_00014217	+
chr4	10069734	10073254	TCONS_00007979	+
chr2	218851519	218858457	TCONS_00004027	+
chrY	8555397	8559482	TCONS_00017645	−
chr13	114920207	114988540	TCONS_l2_00007037+TCONS_l2_00007038+TCONS_00021922+TCONS_l2_00007040+TCONS_l2_00007041+TCONS_l2_00007042+TCONS_l2_00007043	+
chr22	37099963	37117494	TCONS_00029337	+
chr13	27781432	27782326	TCONS_00022237+TCONS_00022238+TCONS_00021947	−
chr6	159485760	159486305	TCONS_00011603	−
chr6	52529199	52533951	LOC730101	+
chr12	4017195	4029987	TCONS_00020678	−
chr1	184638176	184641354	TCONS_00001757	−
chr1	143347958	143405365	TCONS_l2_00002156+TCONS_l2_00002157+TCONS_l2_00000557+TCONS_l2_00002158+TCONS_l2_00002159+TCONS_l2_00002160+TCONS_l2_00002161+TCONS_l2_00000558+TCONS_l2_00002162+TCONS_l2_00002163+TCONS_l2_00000559+TCONS_l2_00000560+TCONS_l2_00002164+TCONS_l2_00002165+TCONS_l2_00002166+TCONS_l2_00002167+TCONS_l2_00002168+TCONS_l2_00000561+TCONS_l2_00002169+TCONS_l2_00002170+TCONS_l2_00000562+TCONS_l2_00002171+TCONS_l2_00002172+TCONS_l2_00000563	+
chr1	94784035	94790125	TCONS_00000574	−
chr15	26715903	26723019	TCONS_00023596	−
chr10	19493962	19498547	TCONS_l2_00002905	+
chr4	162164088	162173336	TCONS_00008637	−

chr1	219391858	219500273	TCONS_00002232+TCONS_00001278+TCONS_00001279+TCONS_00001280	+
chr12	106180061	106404785	TCONS_00020906	−
chr16	33412888	33424992	TCONS_l2_00009696+TCONS_l2_00009697	+
chr4	37003464	37020620	TCONS_00008038	+
chr1	79735992	79871942	TCONS_00002076+TCONS_00001003+TCONS_00002077+TCONS_00000238+TCONS_00002078+TCONS_00002079	+
chr14	56981069	57018025	TCONS_00022347	+
chrY	7672965	7678723	TTTY12	+
chr21	43208994	43211778	TCONS_00029147	−
chr7	25223965	25233536	TCONS_l2_00025724	+
chr1	56522357	56526505	TCONS_00001499	−
chr7	57879404	57897184	TCONS_l2_00025905+TCONS_l2_00025906	+
chr9	6047360	6066714	TCONS_00015646	+
chr2	103603781	103605023	TCONS_00002956	+
chr3	112876555	112902095	TCONS_00005570+TCONS_00005571+TCONS_00006154	+
chr9	135894806	135896562	TCONS_l2_00029098	+
chrX	55306542	55381743	TCONS_l2_00030215+TCONS_l2_00030216+TCONS_l2_00030696+TCONS_l2_00030217	+
chr2	6122110	6128364	LOC400940	+
chr4	115233102	115286113	TCONS_00008550+TCONS_00009107+TCONS_00008551+TCONS_00008552+TCONS_00009108+TCONS_00008553	−
chr1	152902516	152921686	TCONS_00000641	−
chr12	67788395	67815084	TCONS_00020459	+
chr19	8008906	8014249	TCONS_00026882	+
chr2	2645761	2660402	TCONS_00003151	−
chr11	61264004	61274382	TCONS_00019313+TCONS_00019909+TCONS_00019314+TCONS_00019315	+
chr7	26392	35472	TCONS_00013664	−
chr11	5226271	5228538	TCONS_00019139	+
chr8	9009253	9016127	TCONS_00014599+TCONS_00014600+TCONS_00015212+TCONS_00014601+TCONS_00014602+TCONS_00015213+TCONS_00014603	+
chr6	105851188	105927108	TCONS_00011908	+
chr20	11849692	11859217	TCONS_00028317+TCONS_00028617+TCONS_00028618+TCONS_00028619+TCONS_00028620	−
chr4	135854044	135855286	TCONS_00007837	−
chr9	136619187	136620083	TCONS_00016205	+
chr11	32472909	32522171	TCONS_l2_00004466+TCONS_l2_00004467+TCONS_l2_00004468	+
chr19	32880956	32896445	LOC400684	−
chr1	84830641	84863576	UOX	−
chr1	53905438	53923449	TCONS_00000946+TCONS_00002032	+
chr22	17182935	17185326	TCONS_l2_00017865	−
chr15	85070676	85077469	TCONS_00023996	+
chr4	117274143	117276371	TCONS_00008203	+
chr3	195722896	195755017	TCONS_l2_00019098+TCONS_l2_00019099	+
chr8	61822077	61823092	TCONS_00015022	−

chr2	84696981	84705149	TCONS_00004321	−
chr19	37264057	37266689	TCONS_00026788	+
chr15	24415175	24426091	TCONS_00023303+TCONS_00023857	+
chr16	33391293	33401545	TCONS_l2_00009695	+
chr11	45755963	45767510	TCONS_00019298	+
chr2	78315856	78354932	TCONS_00002942+TCONS_00003745+TCONS_00003746	+
chr4	118235753	118281795	TCONS_00007817+TCONS_00009109	−
chr17	49414076	49419932	TCONS_00025124	+
chr12	77622388	77622717	TCONS_00020849	−
chr5	72750012	72768721	TCONS_00009697	−
chr17	46782565	46784708	TCONS_00025175+TCONS_00025176	+
chr20	2174688	2181315	TCONS_00028091	+
chr10	26878794	26883251	LINC00264	+
chr22	45894164	45894662	TCONS_00029721	−
chrY	27209230	27246039	TTTY4B	−
chr2	86209699	86225242	TCONS_00004325	−
chr5	98825263	98835071	TCONS_l2_00023491	+
chr11	69184377	69187267	TCONS_00019368	+
chrY	9638762	9650854	TTTY22	+
chr5	114522574	114528483	TCONS_00011020	−
chr1	205251239	205264218	TCONS_00001780	−
chr5	4866626	4874872	TCONS_00009854+TCONS_00009335	+
chr8	49532379	49536448	TCONS_00014691+TCONS_00014692	+
chr5	70868036	70874285	TCONS_00010710+TCONS_00009991+TCONS_00009992 +TCONS_00010711+TCONS_00009993	+
chr6	170124795	170126184	TCONS_00012835+TCONS_00011627+TCONS_00012374	−
chr4	159687587	159689811	TCONS_00008635	−
chr13	44806061	44810195	TCONS_00021642	−
chr8	109504677	109559554	TCONS_00014831	+
chr6	25053855	25057301	TCONS_00011469	−
chr10	82289039	82295698	TCONS_00018984+TCONS_00018009	−
chr5	8525143	8580485	TCONS_00009868+TCONS_00010620+TCONS_00009347	+
chrX	28589649	28604512	TCONS_00017049	−
chr21	44384749	44385473	TCONS_00028943	−
chr17	75718954	75724641	TCONS_00025254	−
chr6	3026411	3027751	TCONS_00011649	+
chr3	164431883	164549268	TCONS_00005875+TCONS_00005876	−
chr5	20612734	20614874	TCONS_00009616	−
chr1	16123397	16126839	TCONS_00001406	−
chrY	28269867	28275354	TCONS_l2_00031006	+
chr5	95860971	95882222	TCONS_00009466	+
chr20	29872932	29874661	TCONS_l2_00016556	−
chr6	2854891	2876744	MGC39372	−
chr15	26640213	26734856	TCONS_00023889+TCONS_00023326+TCONS_00023257 +TCONS_00023258+TCONS_00023327	+
chr16	22386850	22393983	TCONS_l2_00009632	+
chr4	168228063	168229940	TCONS_00007443	−
chr16	32769845	32771901	TCONS_l2_00009683+TCONS_l2_00009684	+

chr2	128456823	128458363	TCONS_00004440	−
chr6	119855041	120010205	TCONS_00011938+TCONS_00011939	+
chr11	2900625	2902339	TCONS_00019566	−
chr3	75673411	75679341	TCONS_l2_00019360	−
chr1	51443233	51479174	TCONS_00000937+TCONS_00000938	+
chr17	21356142	21357099	TCONS_00025333	+
chr2	23553535	23554915	TCONS_00003233	−
chr15	24470891	24525611	TCONS_00023305+TCONS_00023859+TCONS_00023860+TCONS_00023861+TCONS_00023862+TCONS_00023863+TCONS_00023864+TCONS_00023306	+
chr13	88333940	88349465	TCONS_00021866+TCONS_00021867	+
chr1	11786468	11791920	TCONS_00001389	−
chrX	115803862	115812236	TCONS_l2_00030845	−
chr14	32543521	32545834	TCONS_00022460	+
chr13	53720674	53725943	TCONS_00021657	−
chr7	95987713	96005729	TCONS_00013542	+
chr11	129482020	129487164	TCONS_00019178	−
chr2	159542415	159610287	TCONS_l2_00015526	+
chr3	52273280	52275113	TCONS_00005410+TCONS_00005518	+
chr16	77786366	77809904	TCONS_00024473	+
chr5	126554726	126565505	TCONS_00010466+TCONS_00010467+TCONS_00010468	−
chr5	73568706	73617160	TCONS_00010377+TCONS_00009700+TCONS_00010378	−
chr3	117391001	117716439	TCONS_l2_00020176+TCONS_00006617+TCONS_00005820+TCONS_00007240+TCONS_00007241+TCONS_00006618+TCONS_00007242	−
chr3	47575529	47580118	TCONS_00006013	+
chr11	67326816	67330888	TCONS_00019680	−
chrY	14093473	14100009	TCONS_l2_00030990	+
chr4	70999321	71012421	CSN1S2BP	+
chr9	132028944	132074813	TCONS_l2_00029048+TCONS_l2_00029049+TCONS_l2_00029050+TCONS_l2_00029051+TCONS_l2_00029052+TCONS_l2_00029053+TCONS_l2_00029054	+
chr22	37946033	37947742	TCONS_00029423	+
chr1	208136996	208139265	TCONS_00001255	+
chr3	186525481	186535910	TCONS_00005685	+
chr1	181904062	181906113	TCONS_00001215	+
chrX	13346697	13348212	TCONS_l2_00030150	+
chr19	37025703	37026137	TCONS_00027011	+
chr16	59475882	59497658	TCONS_00024692	−
chrX	13284167	13321571	TCONS_00017040+TCONS_00017041	−
chr13	41371121	41495886	TPTE2P5	−
chr11	123184389	123185528	TCONS_00020146+TCONS_00019788	−
chr16	87527793	87552440	TCONS_00024257+TCONS_00024903+TCONS_00024501+TCONS_00024502	+
chr13	59104272	59104609	TCONS_l2_00006869	+
chr6	148454945	148458540	TCONS_00011591	−
chr19	8740117	8745199	TCONS_00026885	+
chr5	5046909	5049610	TCONS_00009591+TCONS_00009592	−
chr14	95982423	95984597	TCONS_l2_00007890	+

chr22	27703777	27713416	TCONS_00029565+TCONS_00029335+TCONS_00029566+TCONS_00029784	+
chr4	69914349	69915748	TCONS_00009070	−
chr1	31333698	31335492	TCONS_00001972+TCONS_00000881	+
chr1	207096852	207097961	TCONS_00002569+TCONS_00001785	−
chr11	65218532	65221007	TCONS_00019322	+
chr7	76531808	76548602	TCONS_l2_00027142+TCONS_l2_00026028+TCONS_l2_00027143	+
chrY	17571786	17572619	TCONS_00017638	+
chr3	63727851	63735595	TCONS_l2_00019332	−
chr15	30115425	30117856	TCONS_00023895	+
chr2	238337563	238343465	TCONS_00003518	−
chr20	60520108	60523627	TCONS_00028253	+
chr21	46490871	46493126	SSR4P1	−
chr14	103236547	103241108	TCONS_00022889	−
chr17	25667040	25680844	TCONS_l2_00011159+TCONS_l2_00011160	−
chr13	51657937	51659535	TCONS_00021551	+
chr10	58867073	58868602	TCONS_00018522	−
chr10	91451057	91457685	FLJ37201	−
chr6	119103871	119104581	LOC100287632	+
chr3	17784428	17787157	TCONS_00006798	+
chr3	78087856	78095945	TCONS_00005789	−
chr13	45902703	45903917	TCONS_00021991	−
chr7	36069221	36095041	TCONS_00012993	+
chr5	24835389	24840692	LOC340107	−
chr15	32698801	32727065	ULK4P3	−
chr6	14292149	14293374	TCONS_00012086	−
chr20	8945349	8948885	TCONS_00028101	+
chr20	32577408	32579666	TCONS_00028653	−
chr2	71923940	72005321	TCONS_00003731+TCONS_00003732	+
chr7	13893980	13898650	TCONS_00013123	−
chr1	18712323	18715008	TCONS_00000161	+
chr3	157694350	157695925	TCONS_00005860	−
chr8	33462453	33467086	TCONS_00014968	−
chr3	142645517	142661378	LOC100507389	+
chr18	73837962	73839080	TCONS_00026571	−
chr3	40807698	40904117	TCONS_00005514	+
chr6	166186540	166190289	TCONS_00012357+TCONS_00011613	−
chr21	45593932	45608380	TCONS_00029173	−
chr3	59627441	59644000	TCONS_00006518	−
chr20	46749143	46750292	TCONS_00028197	+
chr3	43156200	43168213	TCONS_00006001	+
chr10	27587363	27596344	TCONS_00018460+TCONS_00018461	−
chr20	22656710	22665982	TCONS_00027884+TCONS_00028020+TCONS_00028639	−
chr19	22442700	22467809	TCONS_00026953	+
chr4	188329844	188337228	TCONS_l2_00020949+TCONS_l2_00020950+TCONS_l2_00021793	+

chr18	76736573	76739081	TCONS_00026742+TCONS_00026743+TCONS_00026584+TCONS_00026744+TCONS_00026585	−
chr1	50782665	50786833	TCONS_00001492+TCONS_00002381+TCONS_00001493	−
chr17	69093915	69198318	TCONS_00025148	−
chr6	157541408	157542515	TCONS_00012341	−
chrY	20990485	20995792	TCONS_l2_00030938	−
chr1	219582617	219632787	TCONS_00001827+TCONS_00000733+TCONS_00000734+TCONS_00001828	−
chr6	35307829	35308188	TCONS_00011213	−
chr16	72463252	72567788	TCONS_00024252	+
chr16	56142867	56170675	TCONS_00024850+TCONS_00024851+TCONS_00024854+TCONS_00024853+TCONS_00024852	+
chr7	49751978	49760514	TCONS_00013443	+
chr19	1246146	1248229	TCONS_00027655+TCONS_00027154	−
chr2	242835752	242844846	TCONS_00004646+TCONS_00004647+TCONS_00004648	−
chr22	51110333	51112874	TCONS_l2_00017835	+
chr5	177661604	177662081	TCONS_00010855	+
chr15	85798751	85801879	TCONS_l2_00009263	−
chr13	23269999	23270909	TCONS_l2_00007075	−
chr11	32133596	32174461	TCONS_l2_00004957	−
chr6	168625959	168627197	TCONS_00011618	−
chr2	3616113	3622828	TCONS_00003161+TCONS_00004100	−
chr1	244080704	244210619	LOC339529	+
chr13	46259207	46275492	TCONS_l2_00007167	−
chr2	906612	916810	TCONS_00003147	−
chr9	7177450	7202875	TCONS_00015913+TCONS_00015914	+
chr12	88811915	88813084	TCONS_00020502	+
chr21	32333642	32361891	TCONS_00029109	−
chr6	170454222	170456494	TCONS_00012632+TCONS_00012028	+
chr12	128603110	128605993	TCONS_00020647+TCONS_00021256	+
chrX	88678737	88701982	TCONS_00016999+TCONS_00017000	+
chr20	20830161	20835182	TCONS_l2_00016499	−
chr2	1562698	1567389	TCONS_00004668+TCONS_00004669	+
chr18	57837848	57845359	TCONS_00026536	−
chr10	54316462	54515091	TCONS_l2_00004190+TCONS_l2_00003582	−
chr1	61005921	61106163	TCONS_00000531+TCONS_00001511	−
chr22	23878892	23889980	TCONS_l2_00017644+TCONS_l2_00017645	+
chr4	105575031	105887950	TCONS_l2_00021937	−
chr2	64501019	64550940	TCONS_00003709+TCONS_00004804	+
chr16	70835990	70840039	TCONS_00024294	+
chr10	115767944	115777979	TCONS_00018343	+
chr8	5916281	5923650	TCONS_00014918	−
chr16	70253484	70259936	LOC100506060	−
chr17	10750780	10751687	TCONS_00025308	+
chr7	112586295	112635753	TCONS_l2_00026845+TCONS_l2_00027458+TCONS_l2_00026846+TCONS_l2_00026847+TCONS_l2_00026848+TCONS_l2_00027459	−
chr9	132199779	132201397	TCONS_00016185+TCONS_00015763	+

chr14	101039829	101046700	TCONS_00022626+TCONS_00022627	+
chr13	74734586	74735847	TCONS_00021825	+
chr6	26673488	26688063	TCONS_00011747+TCONS_00011748+TCONS_00012442+TCONS_00011749+TCONS_00012443+TCONS_00011750+TCONS_00011751+TCONS_00011752+TCONS_00012444	+
chr6	19323614	19640814	TCONS_l2_00024638+TCONS_l2_00025345	−
chr9	102648728	102657226	TCONS_00016098	+
chr20	31802726	31804873	TCONS_00028036	−
chr11	107984368	107984754	TCONS_00019752	−
chr15	95126784	95128216	TCONS_00023797+TCONS_00024207	−
chr12	68359719	68364900	TCONS_00020462	+
chr20	56739117	56747176	TCONS_00028455	−
chr18	36171689	36172773	TCONS_00026495	−
chr1	5550602	5551645	TCONS_00000830	+
chr10	55050832	55070825	TCONS_00017984	−
chr4	33852213	33981558	TCONS_l2_00021564+TCONS_l2_00021565+TCONS_l2_00021566+TCONS_00007384	+
chr2	213660045	213683988	TCONS_00004995+TCONS_00004996+TCONS_00004007+TCONS_00004008+TCONS_00003093+TCONS_00003094	+
chr7	73695302	73703578	TCONS_00013864	−
chr1	212800569	212811902	TCONS_00001808+TCONS_00001809+TCONS_00001810+TCONS_00002580+TCONS_00001811+TCONS_00002581+TCONS_00002582	−
chr20	22449343	22452197	TCONS_00028331	−
chr22	20850199	20856009	TCONS_00029516+TCONS_00029762+TCONS_00029763+TCONS_00029517+TCONS_00029518+TCONS_00029519+TCONS_00029764+TCONS_00029520+TCONS_00029765+TCONS_00029521+TCONS_00029766	+
chr21	47476430	47477481	TCONS_00028864	+
chr16	85340979	85345537	TCONS_00024749	−
chr1	54982177	54983024	TCONS_00000947	+
chr1	234765057	234770526	LINC00184	+
chr6	10460125	10460788	TCONS_l2_00024608	−
chr3	194429150	194502897	LOC100507391	+
chr4	146389633	146390834	TCONS_00008605	−
chr6	31447759	31462537	TCONS_00012692	−
chr3	75668379	75672924	TCONS_l2_00019359	−
chr8	10708201	10714730	TCONS_00015214+TCONS_00014616	+
chr5	171165001	171172081	TCONS_00010828+TCONS_00010829+TCONS_00010166	+
chr5	52799691	52811661	TCONS_00010331	−
chr6	170337139	170337952	TCONS_00012375	−
chr18	24915365	25175128	TCONS_l2_00012033+TCONS_l2_00012207	−
chr9	38848558	38851913	TCONS_00015670	+
chr7	97595908	97601638	MGC72080	−
chr6	53426087	53481967	TCONS_00011219+TCONS_00011220+TCONS_00012189+TCONS_00011504+TCONS_00011505+TCONS_00011506+TCONS_00011507	−
chr12	127172956	127203891	TCONS_00021251+TCONS_00020634+TCONS_00020635	+
chr15	22546565	22570831	REREP3	+

chr2	99378401	99388659	TCONS_00003338+TCONS_00004347+TCONS_00004348+TCONS_00003339+TCONS_00005211	−
chr18	59414736	59416200	TCONS_00026541	−
chr1	88937999	88939861	TCONS_00001564	−
chr5	29355912	29396100	TCONS_00010290+TCONS_00010291+TCONS_00010292+TCONS_00009626+TCONS_00009627	−
chr6	183914	187741	TCONS_00011630	+
chr13	75223482	75307001	TCONS_00022050+TCONS_00022293	−
chr7	106264518	106269142	TCONS_00014189	+
chr10	49872244	49880469	TCONS_00017977	−
chr20	21510840	21511382	TCONS_00027910	+
chr11	74373981	74376920	TCONS_00019705	−
chr2	171826456	171829764	TCONS_00003936	+
chr5	135235958	135240982	TCONS_00010493	−
chr9	32724131	32761233	TCONS_l2_00029235	−
chr2	231849083	231860747	LOC348761	−
chr3	10766607	10780748	TCONS_00006440	−
chr17	80340379	80340907	TCONS_00026002	+
chr9	98312138	98315279	TCONS_00016084	+
chr2	108938694	108970254	SULT1C2P1	+
chr5	173763174	173959574	TCONS_l2_00022637+TCONS_l2_00022638+TCONS_l2_00023586+TCONS_l2_00023587+TCONS_l2_00023588+TCONS_l2_00023589+TCONS_l2_00022639+TCONS_l2_00022640+TCONS_l2_00023590+TCONS_l2_00023591+TCONS_l2_00022641	+
chr6	29113638	29128996	TCONS_l2_00024691	−
chr9	45004879	45023236	TCONS_00015675	+
chr1	33183335	33190838	TCONS_00000502	−
chr5	139976204	139992640	TCONS_l2_00022545	+
chr14	60706845	60712368	TCONS_00022372+TCONS_00023115	−
chr9	97109392	97109900	TCONS_l2_00029476	−
chr17	18343222	18346208	KRT16P1	+
chr2	65952596	66016024	TCONS_00004805+TCONS_00003716+TCONS_00002911+TCONS_00002912+TCONS_00002702+TCONS_00002703+TCONS_00002913+TCONS_00003717+TCONS_00002914+TCONS_00004806	+
chr4	147555779	147559915	TCONS_00008282	+
chr4	19748802	19756214	TCONS_00007726	−
chr6	58182220	58187271	TCONS_00011845	+
chr17	42835259	42835905	TCONS_00025402	+
chr10	31427803	31429308	TCONS_00018153	+
chr3	64438807	64448067	TCONS_00007178+TCONS_00006523	−
chr4	174978681	175141549	TCONS_l2_00021403+TCONS_l2_00021404+TCONS_l2_00021405+TCONS_l2_00021406+TCONS_l2_00021407+TCONS_l2_00022054+TCONS_l2_00021408+TCONS_l2_00021409+TCONS_l2_00022055+TCONS_l2_00021410+TCONS_l2_00021411+TCONS_l2_00022056+TCONS_00007904	−
chr8	89723724	89727163	TCONS_00014791	+
chr12	85306655	85386508	TCONS_00020497+TCONS_00020498	+

chr16	9100745	9101248	TCONS_00024324	+
chr13	66361679	66362844	TCONS_l2_00007244+TCONS_l2_00007245	−
chr20	40642674	40644118	TCONS_00028046	−
chr10	102819376	102820683	TCONS_00018588	−
chr16	87306766	87326034	TCONS_00025090+TCONS_00025091	−
chr7	33908113	33913843	TCONS_00013154	−
chr15	21145767	21198633	CT60	+
chr1	198952450	198954529	TCONS_00000364+TCONS_00000365	+
chr15	97725803	97767418	TCONS_00023815	−
chr17	8622165	8625087	TCONS_00025299	+
chr11	95347989	95421027	TCONS_00019736	−
chr11	79813116	79828211	TCONS_00019717	−
chr9	67340517	67343501	TCONS_00015993	+
chr5	6766004	6772066	TCONS_00009339	+
chrY	6110487	6111651	TTTY23	−
chr12	131780660	131782517	TCONS_00020228+TCONS_00021006	−
chr3	176411541	176431515	TCONS_00007043	+
chr3	153094620	153095351	TCONS_00005628	+
chr1	116473512	116474136	TCONS_00001115	+
chr6	32861953	32871535	LOC100294145	+
chr7	138912162	138915171	TCONS_l2_00026919+TCONS_l2_00026920	−
chr2	126456153	126523419	TCONS_00003850+TCONS_00002999	+
chr4	190802233	190806061	TCONS_00008989+TCONS_00007705	+
chrX	118425492	118469573	TCONS_00017009	+
chr8	16405933	16425143	TCONS_00014629	+
chr22	32366705	32369462	TCONS_00029581+TCONS_00029413	+
chr10	4242829	4285981	TCONS_00018416+TCONS_00018874+TCONS_00017906 +TCONS_00017907	−
chr12	122510024	122514686	TCONS_00020239	+
chr22	37748234	37750879	TCONS_00029349	−
chr9	113100291	113100899	TCONS_00016129	+
chr15	69373190	69388163	LINC00277	+
chr9	68427783	68454375	LOC642236	−
chr1	161265664	161266179	TCONS_00001179	+
chr8	143719905	143722021	TCONS_00015377	+
chr8	54190698	54216007	TCONS_00015002	−
chr1	52035312	52039739	TCONS_l2_00001233	−
chr11	68996233	69002418	TCONS_00019682+TCONS_00019683	−
chr7	95225994	95243031	TCONS_00013047+TCONS_00013048+TCONS_00013538 +TCONS_00013049	+
chr9	110100227	110101430	TCONS_00016665	+
chr22	48870120	48885122	TCONS_00029734	−
chr6	54704916	54705653	TCONS_00011510	−
chr7	20628686	20629433	TCONS_l2_00026416	−
chr19	28283232	28302110	TCONS_l2_00012432+TCONS_l2_00012433+TCONS_ l2_00012434+TCONS_l2_00012435+TCONS_l2_00012436	+
chr15	99570738	99574238	TCONS_00023546	+
chr8	72587564	72616812	TCONS_00014755	+

chr2	52949815	53188604	TCONS_l2_00015800+TCONS_l2_00014571+TCONS_l2_00014572	−
chr3	2004065	2029154	TCONS_00005723	−
chr3	150452163	150456770	TCONS_00007278+TCONS_00007279+TCONS_00007280+TCONS_00007281+TCONS_00007282	−
chr1	95051112	95057853	TCONS_00000575	−
chr16	86754185	86755601	TCONS_00024773+TCONS_00025088+TCONS_00025089+TCONS_00024280	−
chr1	30181698	30182394	TCONS_00000495	−
chr12	74795798	74796369	TCONS_00020846	−
chr2	62785198	62788934	TCONS_l2_00014600	−
chr5	1963723	1967268	TCONS_00009582	−
chr2	69059176	69064856	TCONS_00002930+TCONS_00002931	+
chr12	79194901	79196894	TCONS_00020490	+
chr14	99311233	99311934	TCONS_00022398	+
chr3	127752228	127763759	TCONS_00006652+TCONS_00007259	−
chr15	32698812	32727250	ULK4P2	−
chr7	93652145	93696062	TCONS_l2_00027175+TCONS_l2_00027176+TCONS_l2_00026102	+
chr11	21875259	21878026	TCONS_00019256	+
chr13	110053043	110053988	TCONS_00021596	+
chr22	16199666	16236639	TCONS_l2_00018298+TCONS_l2_00018297+TCONS_l2_00017854+TCONS_l2_00018299+TCONS_l2_00017855+TCONS_l2_00017856	−
chr16	31052374	31056802	TCONS_l2_00010403+TCONS_l2_00009664	+
chr6	28616063	28616843	TCONS_00011472	−
chr11	94370957	94373488	TCONS_00019442+TCONS_00019443+TCONS_00019973+TCONS_00019974	+
chr13	21138446	21141197	TCONS_00021926+TCONS_00021927+TCONS_00021928	−
chr5	153266988	153278547	TCONS_00009805	−
chr15	82380935	82390032	TCONS_l2_00009459+TCONS_l2_00008781+TCONS_l2_00008782	+
chr10	122368120	122571581	TCONS_00018358	+
chr9	16121131	16122432	TCONS_00015651	+
chr6	108582539	108583767	TCONS_00012566	+
chr12	47268597	47270578	TCONS_00020396	+
chr17	77796265	77801616	TCONS_l2_00011006+TCONS_l2_00011007+TCONS_l2_00011008	+
chr2	196545	211224	TCONS_00003526+TCONS_00002784+TCONS_00004654+TCONS_00003527	+
chr2	172101203	172157685	TCONS_00004496	−
chr12	126471669	126528013	TCONS_00020617+TCONS_00020618+TCONS_00020619+TCONS_00020620+TCONS_00020621+TCONS_00020622	+
chr17	38873487	38877280	TCONS_l2_00011646+TCONS_l2_00011647+TCONS_l2_00011219	−
chr6	5875164	5876103	TCONS_00011440	−
chr12	58263619	58290267	TCONS_00020255	−
chr4	120326678	120331815	FLJ14186	−
chr3	194426277	194427693	TCONS_00005703	+

chrY	9590765	9611928	TTTY1B	−
chr13	43011563	43059896	TCONS_00021769	+
chr8	41998258	41998755	TCONS_00014990	−
chr7	7294785	7317410	TCONS_00012946+TCONS_00012947	+
chrX	5224214	5225116	TCONS_l2_00030436	−
chr9	45436202	45441760	TCONS_l2_00029301	−
chr7	45831388	45863181	TCONS_l2_00025849+TCONS_l2_00025850+TCONS_l2_00025851+TCONS_l2_00025852+TCONS_l2_00025853+TCONS_l2_00025854+TCONS_l2_00025855+TCONS_l2_00025856+TCONS_l2_00025857+TCONS_l2_00025858	+
chr1	2730632	2734321	TCONS_00000815+TCONS_00001924	+
chr6	163810451	163814232	TCONS_00012353	−
chr2	9897625	9910470	TCONS_00003204	−
chr6	169365370	169367671	TCONS_00012022	+
chr2	107138359	107154535	TCONS_00003359	−
chr21	45894774	45899251	TCONS_l2_00017094+TCONS_l2_00017095	+
chr16	66041412	66048221	TCONS_00024700	−
chr9	121406610	121495055	TCONS_00016473+TCONS_00015878+TCONS_00016474+TCONS_00016864	−
chr19	30016347	30017286	TCONS_00027511	+
chr1	190594020	190770788	LOC440704	+
chr2	12739113	12745271	TCONS_00002826	+
chr4	3546453	3550523	TCONS_00007459+TCONS_00007460+TCONS_00007461	+
chr13	58785825	58807251	TCONS_00021555	+
chr10	110731194	110759905	TCONS_00018330+TCONS_00018815+TCONS_00018331	+
chr9	138244530	138246066	TCONS_00015774	+
chr4	135548788	135561139	TCONS_00008256	+
chr3	193558617	193561016	TCONS_00005695	+
chr4	156982169	156983119	TCONS_00008304	+
chr22	46271879	46283628	TCONS_00029490	−
chr13	57787187	57790887	TCONS_00022032	−
chr15	56835154	56921786	TCONS_l2_00009507	−
chr14	77391088	77394837	TCONS_00022561	+
chr5	178121721	178134039	TCONS_00011099	−
chr13	63321740	63370823	TCONS_00022039	−
chr7	45977477	45998250	TCONS_00013003+TCONS_00013436	+
chr21	37455157	37462712	TCONS_00028842	+
chr1	8938894	8939943	ENO1-AS1	+
chr17	75524207	75532721	TCONS_l2_00011599+TCONS_l2_00010985	+
chr19	40129325	40133041	LOC100129935	+
chr22	50055619	50062451	TCONS_00029736+TCONS_00029931+TCONS_00029932	−
chr5	96840400	97006750	TCONS_00009467	+
chr8	77070846	77075228	TCONS_00015042	−
chr20	55689971	55697148	TCONS_00027961	+
chr19	51689126	51694217	TCONS_l2_00012635+TCONS_l2_00012636	+
chr11	64685025	64691932	TCONS_00019147	+
chr2	57928211	57940316	TCONS_00003692+TCONS_00003693	+

chr1	24348319	24350190	TCONS_00000867	+
chr9	130779814	130819718	TCONS_00016479+TCONS_00015885	−
chr2	2838036	2842163	TCONS_00003153	−
chr7	88983550	89126232	TCONS_00013521+TCONS_00013522+TCONS_00013038 +TCONS_00013523+TCONS_00014171	+
chr10	4067109	4132028	TCONS_00018091+TCONS_00018671+TCONS_00018670 +TCONS_00018669+TCONS_00017773+TCONS_0001769 8+TCONS_00018092	+
chr19	33793763	33795963	CEBPA-AS1	+
chr17	72052482	72109041	TCONS_00025762+TCONS_00026157+TCONS_00026158 +TCONS_00025763+TCONS_00026159	−
chr11	116683920	116684719	TCONS_00019176	−
chr20	47100209	47107082	TCONS_00028426	−
chr3	8121009	8154611	TCONS_00005492	+
chr15	74509394	74514264	TCONS_l2_00008733+TCONS_l2_00008734	+
chr11	71991188	71992041	TCONS_l2_00004631+TCONS_l2_00004632	+
chr2	169957253	169957720	TCONS_00003045	+
chr10	73155810	73156474	TCONS_00018533	−
chr10	29420638	29423296	TCONS_00018149	+
chr20	48657244	48658166	LINC00651	−
chr11	125697476	125698358	TCONS_00019524	+
chr1	69709055	69715106	TCONS_00000544	−
chr6	147480061	147502128	TCONS_00011393	+
chr10	46193896	46217888	TCONS_l2_00003533+TCONS_l2_00003534+TCONS_ l2_00003535+TCONS_l2_00003536	−
chr14	62331609	62336605	TCONS_l2_00007764	+
chr2	11497641	11505762	TCONS_00003205	−
chr21	16212636	16216520	TCONS_00029195	+
chr8	39891376	39891902	TCONS_00014681	+
chr11	93342959	93343409	TCONS_l2_00005153	−
chr22	21522047	21546445	TCONS_l2_00018181+TCONS_l2_00018182+TCONS_ l2_00018183+TCONS_l2_00018184+TCONS_ l2_00018185+TCONS_l2_00017630+TCONS_l2_00018186	+
chr11	1798160	1799618	TCONS_00019163	−
chr2	97193680	97198043	TCONS_00002757	−
chr2	88320097	88320452	TCONS_00004326	−
chr8	7333626	7337622	TCONS_00015398+TCONS_00015399	−
chr6	10466993	10482177	TCONS_l2_00024609+TCONS_l2_00024610+TCONS_ l2_00025334	−
chr7	150130742	150145228	LOC285972	+
chr15	78079529	78081011	TCONS_00023469	+
chr4	188437307	188438362	TCONS_00007931	−
chr3	164867794	164875850	TCONS_00005877	−
chr9	98150704	98189098	TCONS_l2_00029479+TCONS_l2_00029480+TCONS_ l2_00029481+TCONS_l2_00030014	−
chr1	38677715	38679106	TCONS_00000896	+
chr12	54821655	54830949	TCONS_00021142+TCONS_00020439+TCONS_00020440	+
chr3	155761851	155762411	TCONS_l2_00019588	−
chr1	11433557	11434764	TCONS_00002290	−

chr8	95565966	95582860	TCONS_l2_00027895+TCONS_l2_00027896+TCONS_l2_00027897	+
chr6	68590881	68599080	TCONS_l2_00025412+TCONS_l2_00024805	−
chr5	5069305	5078424	TCONS_00009593	−
chr6	92338758	92400146	TCONS_l2_00025435	−
chr2	130000719	130112031	TCONS_00003868+TCONS_00003869	+
chr2	123247933	123262758	TCONS_00003845	+
chr10	108045009	108083285	TCONS_00018323+TCONS_00018324+TCONS_00018814	+
chr1	59486059	59510386	TCONS_l2_00002059+TCONS_l2_00000307+TCONS_l2_00000308+TCONS_l2_00000309	+
chr16	66104221	66167947	TCONS_00025035+TCONS_00024701+TCONS_00025036+TCONS_00024702	−
chr6	154832085	154843051	TCONS_00011996	+
chr16	20219935	20220684	TCONS_00024343	+
chr2	6453086	6465778	TCONS_00003174	−
chr17	48127713	48133103	LOC284080	−
chr3	197374634	197381275	TCONS_00006773	−
chr5	3417266	3536208	LOC285577	−
chr15	24545218	24571710	TCONS_00023866	+
chr7	149244194	149321991	TCONS_l2_00026947+TCONS_l2_00027514+TCONS_l2_00027515+TCONS_l2_00026948+TCONS_l2_00026949+TCONS_l2_00026950+TCONS_l2_00026951+TCONS_l2_00026952+TCONS_l2_00026953+TCONS_l2_00026954+TCONS_l2_00026955+TCONS_l2_00026959+TCONS_l2_00026958+TCONS_l2_00026957+TCONS_l2_00026956+TCONS_l2_00026960+TCONS_l2_00026961+TCONS_l2_00026962+TCONS_l2_00026963+TCONS_l2_00027516+TCONS_l2_00027517+TCONS_l2_00027518+TCONS_l2_00027519	−
chr5	7347099	7348225	TCONS_00009598+TCONS_00010261	−
chr7	1019650	1020276	TCONS_00013672	−
chr6	40681150	40683102	TCONS_l2_00025385+TCONS_l2_00024744	−
chrX	46404925	46407910	ZNF674-AS1	+
chr1	53814160	53814905	TCONS_00000522	−
chr12	38937980	38983614	TCONS_00020208	−
chr7	156850403	156851208	TCONS_00013103	+
chr3	119813742	119855628	TCONS_00005429+TCONS_00006171+TCONS_00006172+TCONS_00005579+TCONS_00006940+TCONS_00006173+TCONS_00006174+TCONS_00006941+TCONS_00006175+TCONS_00006176	+
chr14	103734249	103739946	TCONS_l2_00008270	−
chr16	50840167	50841523	TCONS_00024392	+
chr4	175276005	175297596	TCONS_00007905	−
chr9	126759340	126761864	TCONS_00016477	−
chr7	55659767	55660886	TCONS_00013015	+
chrX	68789512	68790200	TCONS_00017063	−
chr4	84589663	84652908	TCONS_00007787	−
chr13	81594776	81618584	TCONS_00021861+TCONS_00021577	+
chr9	47192550	47210048	TCONS_00015990	+
chr18	51105994	51107426	TCONS_00026206	−

chr18	57967896	57968494	TCONS_00026347	+
chr4	150273111	150817385	TCONS_l2_00021347+TCONS_l2_00022023+TCONS_l2_00022024+TCONS_l2_00022025+TCONS_l2_00021348+TCONS_l2_00022026+TCONS_l2_00021349+TCONS_l2_00021350+TCONS_l2_00022027+TCONS_l2_00022028	−
chr10	18941165	18943952	TCONS_00017795	+
chr5	79138491	79139170	TCONS_00010005	+
chr12	69800947	69801567	TCONS_00020465	+
chr1	234844781	234848736	TCONS_00001880	−
chr4	147030607	147043065	LOC100505545	−
chr1	106993265	107028330	TCONS_00001085+TCONS_00002109+TCONS_00001086	+
chr10	99069333	99071581	TCONS_l2_00003732	−
chr3	193994471	194005157	TCONS_00005699	+
chr1	101092606	101112560	LOC100128787	−
chr21	46419127	46424642	LINC00162	−
chr8	142208034	142209262	TCONS_00015184	−
chr10	3360887	3791666	TCONS_00017697+TCONS_00018086+TCONS_00018087	+
chr1	177362423	177546084	TCONS_00000692	−
chr15	43513512	43518404	TCONS_00023655	−
chr21	34854806	34856673	TCONS_00028919+TCONS_00029113	−
chr14	101586897	101587425	TCONS_00022877	−
chr10	4426438	4452804	TCONS_00017699+TCONS_00017774	+
chr6	80581590	80584327	TCONS_00011331	+
chr1	178459067	178468784	TCONS_l2_00001638+TCONS_l2_00002723	−
chr9	95853715	95857831	TCONS_l2_00029473+TCONS_l2_00030011	−
chr4	132694457	132712637	TCONS_00007624+TCONS_00007625+TCONS_00007626	+
chr15	69752807	69755112	TCONS_00023721+TCONS_00023722	−
chr6	961241	1101567	LOC285768	−
chr14	97472219	97474117	TCONS_00023164	−
chr13	43312392	43321582	TCONS_00021980+TCONS_00021979+TCONS_00022265	−
chr12	20278745	20282795	TCONS_00020728	−
chr17	47964961	47978257	TCONS_00025182	+
chr5	86957859	86974964	TCONS_00009443	+
chr4	189659523	189663173	TCONS_l2_00020965+TCONS_l2_00020964+TCONS_l2_00020966	+
chr8	50137497	50142346	TCONS_00014697+TCONS_00014698	+
chr9	121502841	121507590	TCONS_00015743	+
chr10	8092413	8095447	GATA3-AS1	−
chr22	19005347	19007761	DGCR9	+
chr12	10741077	10752434	KLRAP1	−
chr10	67345242	67449364	TCONS_00018743+TCONS_00018221+TCONS_00017835+TCONS_00018222	+
chr12	1762424	1778165	TCONS_00020668+TCONS_00021269	−
chr2	217726274	217730405	TCONS_00004576	−
chr19	35329965	35346208	TCONS_00027518+TCONS_00026786+TCONS_00026998	+
chr12	102317559	102318599	TCONS_00020896	−
chr9	141139721	141142607	TCONS_00016238	+
chr6	155701645	155702317	TCONS_00011599	−

chrY	26288535	26292234	TCONS_l2_00030913	+
chr2	64834446	64843616	LOC339807	+
chr5	151098090	151105861	TCONS_00010141	+
chr8	66898864	66933720	TCONS_00015444+TCONS_00015028+TCONS_00015445	−
chr3	177055416	177066954	TCONS_00005890	−
chr15	72078650	72082603	TCONS_00023453	+
chr4	190114254	190126031	TCONS_00007703+TCONS_00008981	+
chr7	156230483	156238282	LOC285889	−
chr11	62168660	62169776	TCONS_00019651	−
chr9	77070144	77092669	TCONS_00016360+TCONS_00016361	−
chr15	69854059	69863779	LOC145837	+
chr7	155005959	155008794	TCONS_00013649	+
chr2	121444211	121468093	TCONS_00003392+TCONS_00003393	−
chr4	190580759	190582640	TCONS_l2_00021807+TCONS_l2_00020977	+
chr15	20487997	20496811	CHEK2P2	+
chr22	38812661	38819028	TCONS_00029698+TCONS_00029699	−
chr2	207744136	207799719	TCONS_00003998+TCONS_00003999+TCONS_00004985 +TCONS_00004986+TCONS_00004000	+
chr15	38331916	38335102	TCONS_00023629	−
chr18	68002573	68019686	TCONS_00026634+TCONS_00026635+TCONS_00026357 +TCONS_00026637+TCONS_00026638	+
chr4	122996565	122999473	TCONS_l2_00021275+TCONS_l2_00021276	−
chr2	238199650	238203343	TCONS_00004621	−
chr15	83419351	83424082	TCONS_00023488	+
chr2	97948229	97957632	LOC100506123	+
chr8	69956047	69965108	TCONS_l2_00027829	+
chr7	75453418	75465507	TCONS_l2_00026014	+
chr1	161337812	161341754	TCONS_00000324	+
chr5	134460070	134466939	TCONS_00009791	−
chr7	63484796	63490480	LOC100506050	−
chr5	88762354	88765856	TCONS_00009721	−
chr3	149002730	149005632	TCONS_00006263	+
chr14	101123605	101139081	LINC00523	+
chr2	118593926	118596965	TCONS_00002986+TCONS_00003824+TCONS_00002987	+
chr17	80247922	80250690	TCONS_00025153	−
chr20	51226598	51233330	TCONS_00028705+TCONS_00028706+TCONS_00028707 +TCONS_00028708+TCONS_00028709	−
chr7	63360341	63385566	TCONS_l2_00025921+TCONS_l2_00025922+TCONS_ l2_00025923+TCONS_l2_00027126+TCONS_ l2_00025924+TCONS_l2_00025925	+
chr18	77585840	77616419	TCONS_00026384+TCONS_00026385	+
chr13	53767829	53775170	TCONS_l2_00006862	+
chr9	102293804	102304637	TCONS_00016097+TCONS_00016096+TCONS_00015724	+
chr5	142091748	142146100	TCONS_00010124+TCONS_l2_00022558+TCONS_ l2_00022559+TCONS_l2_00023561+TCONS_ l2_00022560+TCONS_l2_00022561+TCONS_ l2_00022562+TCONS_l2_00022563+TCONS_ l2_00022564+TCONS_l2_00022565	+
chr4	31172766	31213297	TCONS_00007502	+

chr11	104439083	104480062	TCONS_00019745	−
chr15	94073471	94076103	TCONS_00023520	+
chr21	29750553	30144463	TCONS_l2_00017186+TCONS_l2_00017187+TCONS_l2_00017188+TCONS_l2_00017477+TCONS_l2_00017478+TCONS_l2_00017189+TCONS_l2_00017190+TCONS_l2_00017191+TCONS_l2_00017192+TCONS_l2_00017479	−
chr10	3274485	3299721	TCONS_l2_00003355+TCONS_l2_00004078+TCONS_l2_00003356	−
chr2	46874505	46877634	TCONS_00004782+TCONS_00004781	+
chrY	28722010	28725947	TCONS_l2_00030917	+
chr3	86254391	86271969	TCONS_00006552	−
chr3	16569683	16581074	TCONS_00006452	−
chr5	99715209	99723958	LOC100133050	−
chr3	164924748	165373211	TCONS_l2_00019958+TCONS_l2_00019959+TCONS_l2_00018976+TCONS_l2_00019960+TCONS_l2_00018977+TCONS_l2_00018979+TCONS_l2_00018978+TCONS_l2_00018980+TCONS_l2_00018981+TCONS_l2_00018982+TCONS_l2_00019961	+
chrY	13500641	13538613	TCONS_l2_00030934	−
chr5	33009100	33025830	TCONS_00009376	+
chrX	7896716	8030696	TCONS_00017403	+
chr13	42079327	42080461	TCONS_00021766	+
chr1	211899562	211901111	TCONS_00001262	+
chr10	30865628	30869111	TCONS_00017805	+
chr15	63472809	63481559	TCONS_00023440	+
chr1	148933291	148951501	TCONS_00002468	−
chr3	128949393	128955149	TCONS_00006222+TCONS_00006223	+
chr17	5675554	5834016	LOC339166	+
chr7	26301389	26303446	TCONS_00013382	+
chr3	178819159	178865761	TCONS_00005477	−
chr16	24715881	24721546	TCONS_00024285	+
chr11	131062532	131064247	TCONS_00019542	+
chr10	6933419	6977358	TCONS_00017920+TCONS_00018426	−
chr11	65574367	65585604	TCONS_00019670+TCONS_00019671	−
chr1	103142328	103145365	TCONS_00001082	+
chr16	46658641	46660887	TCONS_l2_00010143	−
chrY	14465804	14468226	TCONS_l2_00030991	+
chr22	48537359	48539046	TCONS_00029439	+
chr6	53493178	53496192	TCONS_00011314+TCONS_00012509	+
chr9	2390027	2429986	TCONS_00015908	+
chr1	150134879	150153235	TCONS_00002160+TCONS_00002161	+
chr6	84437774	84446244	TCONS_00011525	−
chr5	35318320	35318753	TCONS_00010299	−
chr5	87484995	87485986	TCONS_00010981+TCONS_00010982	−
chr6	36084167	36091301	TCONS_00011799	+
chr5	163897276	163978193	TCONS_00010826+TCONS_00010163+TCONS_00010827	+

chr14	66578250	66596708	TCONS_00023004+TCONS_00023005+TCONS_00022532+TCONS_00022533+TCONS_00023006+TCONS_00022534+TCONS_00023007+TCONS_00023008+TCONS_00022535+TCONS_00023009	+
chr3	898807	899774	TCONS_00005487	+
chr2	60666528	60666905	TCONS_00004267	−
chr2	5950773	5952728	TCONS_00003173+TCONS_00004112	−
chr7	133969252	133970807	TCONS_00013082	+
chr11	22513641	22531940	TCONS_00019871+TCONS_00019259	+
chr18	45346666	45356490	TCONS_00026518	−
chr4	28345393	28395901	TCONS_00008454+TCONS_00008455+TCONS_00009035	−
chr1	27986839	27989233	TCONS_00000492	−
chr6	22349447	22517940	TCONS_00011730	+
chr7	20257209	20261315	TCONS_l2_00027063+TCONS_l2_00027064+TCONS_l2_00027065+TCONS_l2_00025701+TCONS_l2_00025702+TCONS_l2_00025703	+
chr7	36502632	36544121	TCONS_00012994	+
chr15	65128039	65128508	TCONS_00023699	−
chr3	147406753	147450204	TCONS_00006678	−
chr15	32474850	32500363	TCONS_00023604	−
chr19	30569112	30576907	TCONS_00026983	+
chr10	3247908	3248816	TCONS_00017765	+
chr12	32111689	32112260	TCONS_00021299	−
chr3	22423310	22597727	TCONS_l2_00019768+TCONS_l2_00019769	+
chr4	55809796	55822985	TCONS_00007533	+
chr7	25587382	25590682	TCONS_00013144	−
chr21	34224422	34261285	TCONS_00029002	+
chr5	140088281	140091097	TCONS_00010506	−
chrX	41134992	41136031	TCONS_00016979	+
chrX	20328060	20330276	TCONS_00017410	+
chr1	27533429	27535176	TCONS_l2_00000179+TCONS_l2_00000180	+
chr16	50441583	50442413	TCONS_00024391	+
chr9	94903749	94921890	LINC00475	+
chr2	67551759	67552436	TCONS_00002928	+
chr10	103082400	103082763	TCONS_00018033	−
chr16	55400658	55403877	TCONS_00024666	−
chr2	77879539	77881794	TCONS_00003324	−
chr11	123542616	123545215	TCONS_00019512	+
chr8	128698590	128746210	TCONS_00014535	−
chr15	21912351	21913857	TCONS_00023571	−
chr21	36096105	36109479	LINC00160	−
chr16	22934718	22959612	TCONS_00024611	−
chr16	32626088	32626953	TCONS_00024370	+
chr20	4630286	4634231	TCONS_l2_00016455	−
chr2	45401480	45482080	UNQ6975	−
chr11	6387577	6392797	TCONS_00019211+TCONS_00019212	+
chr1	158119644	158122023	TCONS_00001175	+
chr8	5517279	5522285	TCONS_00014914	−

chr19	56910271	56915279	TCONS_00027426	−
chr1	53832340	53833917	TCONS_00000523	−
chr13	70681345	70713885	ATXN8OS	+
chr7	98855833	98859259	TCONS_l2_00027182	+
chr7	51681947	51698566	TCONS_00013010	+
chr20	49615781	49617367	TCONS_00028217	+
chr7	127752094	127758774	TCONS_00013584	+
chr1	111023388	111033891	CYMP	+
chr1	233046450	233060203	TCONS_l2_00000903	+
chr4	59850000	59912677	TCONS_00007539+TCONS_00007540	+
chr12	87556357	87581209	TCONS_l2_00006345	−
chr9	138359934	138364095	TCONS_00015775	+
chr12	49194956	49204315	TCONS_00020777	−
chr14	105122494	105123499	TCONS_00022645	+
chr10	5558893	5565140	TCONS_00017727	−
chr3	147998604	148118862	TCONS_l2_00018911+TCONS_l2_00019924+TCONS_ l2_00019925+TCONS_l2_00018912	+
chr10	92618127	92621421	TCONS_00018282	+
chrX	139805658	139806390	TCONS_00017553+TCONS_00017554	−
chr10	99178541	99185802	TCONS_00017747	−
chr22	19654546	19655078	TCONS_00029361	+
chr15	29034980	29101720	LOC646278	+
chr20	4743265	4745021	TCONS_00028096	+
chr3	126006730	126020383	TCONS_00006634+TCONS_00007250+TCONS_000 06635+TCONS_00007251+TCONS_00005821+TCO NS_00006636	−
chr5	67483761	67501722	TCONS_00010707+TCONS_00009985+TCONS_00009986 +TCONS_00010708+TCONS_00010709	+
chr1	192486769	192536347	TCONS_00000706	−
chr1	110370977	110414058	TCONS_00000271	+
chr9	14025970	14030114	TCONS_00015650	+
chr17	49410040	49411451	TCONS_00025189	+
chr17	47325605	47336027	FLJ40194	+
chr4	188344049	188351539	TCONS_00008372	+
chr17	40697486	40700585	TCONS_l2_00010777+TCONS_l2_00010778	+
chr9	121571841	121575354	TCONS_00016143	+
chr6	164085403	164092730	TCONS_00012355	−
chr6	104910763	104951475	TCONS_00012236+TCONS_00012764	−
chr2	235993466	235997230	TCONS_00003516	−
chr22	17308364	17310225	HSFY1P1	+
chr14	54581571	54582221	TCONS_00022511	+
chr20	55171366	55173677	TCONS_00027959	+
chr19	43715943	43752798	LOC284344	−
chr1	143527405	143533272	TCONS_00000633+TCONS_00000634	−
chr4	137499811	137503256	TCONS_00008580	−
chr6	74779167	75400441	TCONS_l2_00025175+TCONS_l2_00024286+TCONS_000 11326+TCONS_l2_00024287	+
chr10	3346789	3356010	TCONS_00018409	−

chr15	39737150	39739940	TCONS_00023359	+
chr8	71701592	71702501	TCONS_00014752	+
chr4	184653378	184658726	TCONS_00007922	−
chr15	69578400	69580905	TCONS_00023718	−
chr19	54703321	54703838	TCONS_00026857	−
chr17	67957744	67979679	TCONS_l2_00010948+TCONS_l2_00010949+TCONS_l2_00011592+TCONS_l2_00011593+TCONS_l2_00010950+TCONS_l2_00010951	+
chr2	192559982	192563100	TCONS_00003974	+
chr4	70276576	70289107	TCONS_l2_00020565	+
chr6	166722953	166731401	TCONS_l2_00024517+TCONS_l2_00024518+TCONS_l2_00024519	+
chr9	129351873	129375979	TCONS_00015882+TCONS_00015883+TCONS_00015884	−
chr9	100164458	100174237	TCONS_00016430	−
chr17	7967151	7968061	TCONS_00025555	−
chr5	127018668	127038662	TCONS_00010470	−
chr5	120658245	120661532	TCONS_00009498	+
chr3	149983199	150014393	TCONS_l2_00020221+TCONS_l2_00019568+TCONS_l2_00019569+TCONS_l2_00019570	−
chr1	23608315	23610576	TCONS_00001416	−
chr9	132262772	132270807	TCONS_00016491	−
chr5	133764742	133796306	TCONS_00009788+TCONS_00010492	−
chr5	2560108	2561410	TCONS_00009838	+
chr12	799000	837499	TCONS_00020269	+
chr4	174374164	174442111	TCONS_00008650	−
chr9	132100958	132102959	TCONS_00016487	−
chr3	191466402	191467882	TCONS_00006736	−
chr11	38639775	38641425	TCONS_00019617	−
chr14	21668238	21675059	LINC00641	−
chr4	16321722	16357365	TCONS_00008003	+
chr21	20716830	20866326	TCONS_l2_00017162	−
chr5	54317122	54319997	TCONS_00010335+TCONS_00009666	−
chr14	56776790	56777445	TCONS_00022515	+
chr12	6301563	6307505	TCONS_00020230	+
chr1	37235456	37241369	TCONS_00000891+TCONS_00000185	+
chr10	112776578	112779659	TCONS_00018816	+
chr6	693957	713766	TCONS_00011632	+
chr7	155986797	156002534	TCONS_00013651	+
chr9	33487059	33489953	TCONS_00015945	+
chr19	50222691	50223369	TCONS_l2_00013175	−
chr14	48234156	48264217	LINC00648	−
chr17	44450179	44718040	NSFP1	+
chrX	74743968	74844631	TCONS_l2_00030263	+
chr2	111099475	111103519	TCONS_00004379	−
chr17	32496078	32510651	TCONS_00026052+TCONS_00025136+TCONS_00026053+TCONS_00025608	−
chr14	75727459	75735928	TCONS_00022555	+

chr2	62801491	62889929	TCONS_00004275+TCONS_00003300+TCONS_00004276+TCONS_00004277+TCONS_00004278+TCONS_00004279+TCONS_00002753+TCONS_00004280	−
chr2	241894036	241906868	LOC200772	−
chr16	26329457	26351967	TCONS_00024820+TCONS_00024287+TCONS_00024288+TCONS_00024355+TCONS_00024356+TCONS_00024821+TCONS_00024289	+
chr18	15305583	15308748	TCONS_l2_00011996	−
chr18	10244401	10247598	TCONS_00026200	−
chr1	221002597	221005768	TCONS_00000065	+
chrY	26356114	26360978	GOLGA2P2Y	−
chr1	219616120	219618818	TCONS_00001281	+
chr4	6202460	6235663	LOC285484	+
chr5	88877026	88973518	TCONS_00009456	+
chr3	75716700	75721122	TCONS_l2_00020140	−
chr16	52330608	52417666	TCONS_00024654+TCONS_00025008	−
chr2	118802136	118816029	TCONS_00003825+TCONS_00003826+TCONS_00004877+TCONS_00003827+TCONS_00003828	+
chr21	35732264	35734378	TCONS_00029005	+
chr14	90982163	90983553	TCONS_00022832	−
chr2	16535524	16613970	TCONS_00002835+TCONS_00002836	+
chr6	11417267	11489312	TCONS_l2_00024035+TCONS_l2_00024036+TCONS_l2_00024037+TCONS_l2_00024038+TCONS_l2_00025091	+
chr3	14689701	14690939	TCONS_00005746	−
chrX	38782353	38796758	TCONS_00017154	+
chr2	8062556	8116945	LOC339788	−
chr4	9533595	9534258	TCONS_l2_00020404	+
chr8	145925737	145934941	TCONS_l2_00028034+TCONS_l2_00028035+TCONS_l2_00028036	+
chr16	55423570	55461681	TCONS_00024848	+
chr9	130545365	130547450	TCONS_00016162+TCONS_00016163	+
chr13	25755579	25763898	TCONS_00021531	+
chrY	1465320	1468295	TCONS_00017617+TCONS_00017618	−
chr1	240821424	240821840	TCONS_00001893	−
chr3	195270871	195281946	TCONS_l2_00020037+TCONS_l2_00019084+TCONS_l2_00019085+TCONS_l2_00020038	+
chr5	172199211	172209337	TCONS_00010172+TCONS_00010832+TCONS_00010173	+
chr7	127116937	127125858	TCONS_00013077	+
chr7	560028	564869	FLJ44511	+
chr2	557825	578145	TCONS_00003532+TCONS_00004656+TCONS_00002792	+
chr1	71303920	71304747	TCONS_00000976	+
chr2	11485211	11489408	TCONS_00003574	+
chr20	3789141	3793080	TCONS_00027857+TCONS_00028488+TCONS_00028489	+
chr4	27738268	27751818	TCONS_00008021	+
chr9	76188531	76194414	TCONS_00015694	+
chr11	45377043	45378689	TCONS_00019292+TCONS_00019293+TCONS_00019893	+
chr15	98105175	98115885	TCONS_00023542	+

chr6	169818907	169846389	TCONS_00012370+TCONS_00012371+TCONS_00011248+TCONS_00012831+TCONS_00012372+TCONS_00012832+TCONS_00012373+TCONS_00012833+TCONS_00012834	−
chr16	23028847	23030781	TCONS_00024349	+
chrX	119124753	119127773	TCONS_00017237+TCONS_00017457	+
chr3	10801169	10805877	LINC00606	−
chr5	171960625	171963089	TCONS_00010169+TCONS_00010831	+
chr7	56563916	56564977	DKFZp434L192	+
chr2	305841	309379	TCONS_00003141+TCONS_00003142	−
chr2	136835462	136836083	TCONS_00003008	+
chr17	7040595	7049718	TCONS_l2_00010547	+
chr1	32813111	32814381	TCONS_00000884	+
chr4	68283023	68333995	TCONS_l2_00021895+TCONS_l2_00021166+TCONS_l2_00021896+TCONS_l2_00021167+TCONS_l2_00021897	−
chr15	80487820	80544417	TCONS_l2_00009224	−
chr20	25990435	26002430	LOC100134868	+
chr7	35654246	35660870	TCONS_00013776+TCONS_00014282	−
chr2	243173849	243176661	TCONS_00003525+TCONS_00004653	−
chr7	153445242	153446094	TCONS_00013637	+
chr21	39378846	39382920	TCONS_00028923	−
chr2	31494729	31496423	TCONS_00004742	+
chr2	6766241	6775067	TCONS_00003176+TCONS_00003177+TCONS_00004113	−
chr18	22096686	22098040	TCONS_00026460	−
chr8	137249498	137255634	TCONS_00015180+TCONS_00015528+TCONS_00015181	−
chr15	100330361	100347132	DNM1P46	−
chr18	68866638	68870617	TCONS_00026359	+
chr20	62133421	62149568	TCONS_00028590+TCONS_00028591+TCONS_00028592+TCONS_00028595+TCONS_00028594+TCONS_00027983	+
chr8	70234191	70238992	TCONS_00015037	−
chr9	133223564	133231246	TCONS_l2_00029086+TCONS_l2_00029087	+
chr4	63994030	64009599	TCONS_00007773	−
chr3	156799630	156806336	TCONS_00005633	+
chr15	101087957	101099488	PRKXP1	−
chr11	88071105	88161394	TCONS_l2_00004704+TCONS_l2_00004705+TCONS_l2_00004706+TCONS_l2_00004707+TCONS_l2_00004708+TCONS_l2_00004709+TCONS_l2_00005330+TCONS_l2_00004710+TCONS_l2_00004711	+
chr7	141968101	141972068	LOC730441	−
chr1	90752760	90763021	TCONS_00001577	−
chr7	13378826	13404674	TCONS_00013122	−
chr3	49586739	49591799	BSN-AS2	−
chr4	22346156	22348426	TCONS_00008446	−
chr16	16549482	16550981	TCONS_00024338	+
chr18	5880672	5890156	TCONS_00026270+TCONS_00026271	+
chr8	67383684	67388211	TCONS_00015292+TCONS_00014743+TCONS_00014744	+
chr1	146853914	146989699	LINC00624	−

chr15	28951503	28954493	TCONS_l2_00008958+TCONS_l2_00008959+TCONS_l2_00008960+TCONS_l2_00008961	−
chr4	107354683	107378478	TCONS_00008864+TCONS_00008865+TCONS_00008866+TCONS_00008867+TCONS_00008868+TCONS_00008186+TCONS_00007586+TCONS_00008187	+
chr17	13679960	13685245	TCONS_00025565+TCONS_00025566	−
chr3	181091678	181160296	TCONS_00007314+TCONS_00007315+TCONS_00006716+TCONS_00005897	−
chr9	89366554	89370049	TCONS_00016040	+
chr2	118966965	118981524	TCONS_00004419	−
chr13	49142298	49144479	TCONS_00022004	−
chr8	37575089	37591177	TCONS_00014676+TCONS_00014488	+
chr19	1261354	1262238	TCONS_00026864	+
chr2	64478231	64479974	TCONS_l2_00013678+TCONS_l2_00013679	+
chr16	28296206	28303385	TCONS_00024265	−
chr20	25989427	25991167	TCONS_00028033	−
chr8	136246374	136311962	LOC286094	+
chr14	73925556	73932873	TCONS_00022787+TCONS_00022788+TCONS_00022789+TCONS_00022790+TCONS_00022791	−
chr13	101591500	101596786	TCONS_l2_00007305	−
chr7	151647471	151652070	TCONS_00013273	−
chr2	180764787	180782371	TCONS_00003954	+
chr17	70399463	70588943	LINC00673	−
chr13	80211603	80240876	TCONS_00021853	+
chr4	187980348	187980856	TCONS_00008368	+
chr2	242483801	242498558	BOK-AS1	−
chr4	130692814	130876523	TCONS_l2_00021706+TCONS_l2_00021707+TCONS_l2_00020763+TCONS_l2_00021708+TCONS_l2_00020764+TCONS_l2_00021709	+
chr6	5031990	5055048	TCONS_00011439+TCONS_00012062+TCONS_00012063	−
chr4	13821734	13978699	TCONS_00007480	+
chr17	20432533	20460098	TCONS_l2_00011139	−
chr10	69609270	69610353	TCONS_00018225	+
chr7	53994369	54015437	TCONS_00013175	−
chr3	194566646	194567366	TCONS_00006371	+
chr11	72514717	72524260	TCONS_00019700	−
chr1	121315744	121322322	TCONS_00000627	−
chr12	78816662	78820523	TCONS_00020851	−
chr6	85589886	85677529	TCONS_l2_00024834	−
chr8	128091145	128094513	TCONS_00015167	−
chr16	34259780	34263920	TCONS_00024635	−
chr18	53440548	53448952	TCONS_00026525+TCONS_00026526+TCONS_00026208	−
chr7	117792883	117823998	TCONS_00013936+TCONS_00013937+TCONS_00013938	−
chr18	73408038	73424340	TCONS_00026214	−
chr9	89204719	89205038	TCONS_00016039	+
chr2	177389607	177396411	TCONS_00003450+TCONS_00004506	−
chr1	79789342	79790552	TCONS_00001535	−
chr4	65779999	65870218	LOC401134	−
chr20	25730059	25732440	TCONS_00028355	−

chr2	65128974	65159581	LOC400958	−
chr14	77507350	77552800	TCONS_00022802+TCONS_00022376+TCONS_00022377+TCONS_00023134+TCONS_00023135+TCONS_00022803+TCONS_00022804+TCONS_00022378	−
chr2	25427182	25432071	TCONS_00002860	+
chr10	127371812	127398246	LOC283038	+
chr8	13690518	13691959	TCONS_00014937	−
chr15	40716332	40728140	TCONS_00023374+TCONS_00023375+TCONS_00023923	+
chr2	151409046	151428735	TCONS_00003420	−
chr6	28805646	28806783	TCONS_00011473	−
chr3	193565532	193570323	TCONS_00006740	−
chr4	41885077	41896596	TCONS_00007752	−
chr15	74044453	74062814	TCONS_00023459+TCONS_00023975+TCONS_00023460+TCONS_00023976+TCONS_00023977+TCONS_00023461+TCONS_00023978	+
chr4	74576019	74580244	TCONS_00007551	+
chr6	150319155	150326280	RAET1K	−
chr18	11124	16882	TCONS_00026589+TCONS_00026223+TCONS_00026224	+
chr7	38052956	38055984	TCONS_00014106	+
chr8	61878746	61940024	TCONS_00014726+TCONS_00014727	+
chr16	60398716	60530011	TCONS_00024426	+
chr18	59319556	59329662	TCONS_00026348	+
chr12	101822394	101825034	TCONS_00020893	−
chr1	65437908	65510206	TCONS_l2_00002521+TCONS_l2_00001272+TCONS_l2_00002522+TCONS_l2_00002523+TCONS_l2_00001273+TCONS_l2_00001274+TCONS_l2_00001275+TCONS_l2_00001277+TCONS_l2_00001276+TCONS_l2_00002524+TCONS_l2_00001278+TCONS_l2_00001279+TCONS_l2_00001280	−
chr13	96301012	96329209	TCONS_00022088+TCONS_00021519	−
chr9	74920308	74960913	TCONS_l2_00028833+TCONS_l2_00029774+TCONS_l2_00028834+TCONS_l2_00028835+TCONS_l2_00028836+TCONS_l2_00028837	+
chr11	8032827	8038037	TCONS_00019117+TCONS_00020038	−
chr1	110751456	110752609	TCONS_00000601	−
chr8	64366195	64369530	TCONS_00014735	+
chr17	27894167	27895678	TCONS_00025884+TCONS_00025885+TCONS_00025886	+
chr4	90472507	90641106	TCONS_l2_00021641+TCONS_l2_00020644+TCONS_l2_00021642+TCONS_l2_00021643+TCONS_l2_00020645+TCONS_l2_00020646+TCONS_l2_00021644+TCONS_l2_00020647+TCONS_l2_00020648	+
chr7	26443108	26535986	LOC441204	+
chr17	3705458	3707099	TCONS_00026019	−
chr7	53582685	53584809	TCONS_00013172	−
chr1	56797824	56840926	TCONS_00001503	−
chr6	19802395	19804981	TCONS_00011208+TCONS_00011462+TCONS_00011463	−
chr4	185748078	185769013	TCONS_00008359	+
chr6	169686629	169689311	TCONS_00011624+TCONS_00011625	−
chr3	169683929	169684501	TCONS_00007041	+
chr1	23007533	23008941	TCONS_00000864	+

chr20	23635862	23637027	TCONS_00028025	−
chr5	34048573	34050025	TCONS_00009378	+
chr9	137371781	137375298	TCONS_00016208	+
chr10	54148995	54153749	TCONS_l2_00003581	−
chr13	23743974	23744736	TCONS_00021529	+
chr7	38391053	38394123	TCONS_l2_00026511	−
chr13	23682067	23692526	TCONS_00021723+TCONS_00021724+TCONS_00021725 +TCONS_00021726+TCONS_00022141	+
chr5	87705664	87794514	TCONS_00009718+TCONS_00009719	−
chr6	138698925	138704585	TCONS_l2_00024447	+
chr3	104731560	104736840	TCONS_00006139	+
chr12	119391194	119393930	TCONS_00020947+TCONS_00020948+TCONS_00020949	−
chr14	54651915	54654999	TCONS_00022749	−
chr6	10434549	10457014	TCONS_00011264+TCONS_00011265	+
chr7	57068863	57069879	TCONS_l2_00025900	+
chr5	177283141	177283894	TCONS_l2_00023603	+
chr21	16866251	16868443	TCONS_00028871	−
chr8	62697580	62798104	TCONS_00014729+TCONS_00014730+TCONS_00014731 +TCONS_00014732+TCONS_00014733	+
chr6	25732661	25733089	HIST1H2APS1	+
chr20	59048188	59055732	TCONS_00028250+TCONS_00027978	+
chr6	82523005	82523909	TCONS_00011224	−
chr8	78376247	78388737	TCONS_00014774	+
chr10	48320921	48326093	TCONS_l2_00003048	+
chr13	19961173	19962000	TCONS_l2_00006704	+
chr12	9523725	9534268	TCONS_l2_00006110	−
chr4	55711734	55725407	TCONS_00007532	+
chr20	6427370	6509106	TCONS_00027897	+
chr1	209701800	209741018	TCONS_00000720+TCONS_00000721	−
chr16	3980005	4002465	TCONS_00024948+TCONS_00024949+TCONS_00024562 +TCONS_00024563+TCONS_00024564+TCONS_0002456 5+TCONS_00024950	−
chr1	202830882	202844369	LOC148709	+
chr6	4610846	4612152	TCONS_00011153	+
chr20	39763826	39765073	TCONS_00028390	−
chr1	218205177	218207002	TCONS_00000392	+
chr10	126571528	126575196	TCONS_00018379	+
chrX	2531032	2533388	LINC00102	−
chr6	44047377	44072432	TCONS_l2_00024768+TCONS_l2_00024769+TCONS_ l2_00025392+TCONS_l2_00024770	−
chr19	51340357	51354377	TCONS_00027078+TCONS_00027079+TCONS_00027080	+
chr3	176262494	176283222	TCONS_00007042+TCONS_00006316	+
chr10	7115611	7118468	TCONS_00018115	+
chr7	113134743	113138753	TCONS_00013229	−
chr3	3292371	3668980	TCONS_00005724+TCONS_00005725+TCONS_00005726 +TCONS_00006398	−
chr1	201508268	201557780	TCONS_00002215+TCONS_00002216+TCONS_00001243	+
chr15	60598089	60599946	TCONS_00023429+TCONS_00023955	+
chr4	106274842	106280565	TCONS_00008185	+

chr20	22380971	22401281	LOC284788	−
chr19	53099937	53103405	ZNF137P	+
chr21	42953359	42954625	TCONS_00029024	+
chr9	101673453	101691817	TCONS_00016660+TCONS_00016095	+
chr3	72082432	72291716	TCONS_l2_00019347+TCONS_l2_00020132+TCONS_l2_00019348+TCONS_l2_00019349+TCONS_l2_00019350+TCONS_l2_00020133+TCONS_l2_00019351+TCONS_l2_00020134+TCONS_l2_00020135+TCONS_l2_00020136+TCONS_l2_00020137+TCONS_l2_00019352+TCONS_l2_00019353+TCONS_l2_00019354	−
chr1	41898931	41930507	TCONS_00000918	+
chr4	148488758	148515738	TCONS_l2_00021746+TCONS_l2_00020808+TCONS_l2_00020809+TCONS_l2_00020810	+
chr6	156693170	156694763	TCONS_00012002	+
chr22	17082801	17129720	TPTEP1	+
chr9	67030150	67269646	TCONS_l2_00028763+TCONS_l2_00028764+TCONS_00016583	+
chr4	75284634	75306174	TCONS_00007778	−
chr3	177534653	177617012	TCONS_00005441+TCONS_00005665	+
chr9	100660035	100664123	TCONS_00016844	−
chr10	92741635	92750450	TCONS_00018783	+
chr7	137029676	137039229	TCONS_00013265	−
chr19	28477699	28491271	TCONS_00027282+TCONS_00027283	−
chr22	34587063	34606437	TCONS_00029684+TCONS_00029685+TCONS_00029475	−
chr18	2350557	2371127	TCONS_00026233+TCONS_00026235	+
chr10	106234697	106240032	TCONS_00018037	−
chr1	212719036	212729407	TCONS_00000387	+
chr2	69059948	69064324	TCONS_00003319	−
chr2	43415402	43433742	TCONS_00003662	+
chr7	150885762	150902611	TCONS_l2_00026978+TCONS_l2_00026979+TCONS_l2_00027520	−
chr3	90255221	90257443	TCONS_l2_00019376	−
chr1	115642293	115645279	TCONS_00000280	+
chr5	54350256	54350968	TCONS_00009401	+
chr3	14389951	14398336	TCONS_l2_00020086+TCONS_l2_00019181	−
chr1	147249689	147267415	TCONS_00001139+TCONS_00000297	+
chrX	70165988	70166689	TCONS_00017192	+
chr20	17853283	17853698	TCONS_00028115	+
chr1	111390708	111391552	TCONS_l2_00001442	−
chr15	58177214	58177626	TCONS_00023686	−
chr20	22567875	22588609	TCONS_00028336+TCONS_00028019+TCONS_00028337+TCONS_00028338	−
chr6	129845158	129873732	TCONS_l2_00024911+TCONS_l2_00025470+TCONS_l2_00025471	−
chr3	96199795	96232359	TCONS_00006128	+
chr1	211809248	211816209	TCONS_00000380	+
chr15	97882983	97885435	TCONS_00023817	−
chr5	71393374	71395047	TCONS_00010370	−
chr4	1126097	1126765	TCONS_l2_00021500	+

chr3	97692621	97693948	TCONS_00006129	+
chr13	40768646	40794639	LINC00548	−
chr6	169575399	169582835	TCONS_00011246	−
chr1	88425115	88427536	TCONS_00001014	+
chr1	101788893	101855859	TCONS_00000261+TCONS_00000262+TCONS_00000263	+
chr6	775456	780214	TCONS_00011201	−
chr7	54874362	54879543	TCONS_00013806	−
chr15	28943751	28948787	TCONS_l2_00008956+TCONS_l2_00008957	−
chr15	36606146	36619385	TCONS_00023625	−
chr2	11988748	12006410	TCONS_00002824+TCONS_00003579	+
chr7	158413308	158416118	TCONS_l2_00027019	−
chr12	63013147	63013730	TCONS_00021153	+
chr3	194482847	194489441	TCONS_00007330+TCONS_00005926	−
chr14	74268468	74270630	TCONS_00023015	+
chr12	46773843	46888235	TCONS_00020768+TCONS_00021304+TCONS_00021305 +TCONS_00021306+TCONS_00021307	−
chr13	25325763	25329506	TCONS_l2_00007086	−
chr21	24437813	24503528	TCONS_l2_00017337	+
chr19	8784389	8790416	TCONS_l2_00012283	+
chr18	49090645	49105047	TCONS_00026329	+
chr20	47129933	47133716	TCONS_00028427+TCONS_00028428+TCONS_00028429	−
chr2	238832145	238842875	TCONS_00004623	−
chr6	49755162	49788663	TCONS_l2_00024241+TCONS_l2_00024243+TCONS_ l2_00024242+TCONS_l2_00024244+TCONS_l2_00025158	+
chr6	27661814	27678001	LOC100507173	+
chr9	66523532	66559785	TCONS_l2_00029960+TCONS_l2_00029311+TCONS_ l2_00029312+TCONS_l2_00029313+TCONS_ l2_00029314+TCONS_l2_00029961+TCONS_l2_00029316	−
chr5	151998525	152603103	TCONS_00009315+TCONS_00009316+TCONS_00009802 +TCONS_00009803+TCONS_00009804	−
chr5	53686668	53710899	TCONS_l2_00023392+TCONS_l2_00023393	+
chr4	139219649	139233812	TCONS_l2_00021309+TCONS_l2_00022002	−
chr1	30888672	30893955	TCONS_00000176	+
chr6	170584462	170588557	TCONS_00011425+TCONS_00011426	+
chr21	40229432	40230764	TCONS_00029016+TCONS_00029015+TCONS_00029215	+
chr7	30216948	30242002	TCONS_00013402	+
chr7	55744425	55746044	TCONS_l2_00026573	−
chr11	41872021	41883375	TCONS_00019280+TCONS_00019281+TCONS_00019890	+
chr18	36894251	36907091	TCONS_00026612	+
chr2	128193816	128228064	TCONS_00004437	−
chr14	97925207	97935153	TCONS_00022358+TCONS_00022620+TCONS_00022621 +TCONS_00022622+TCONS_00023050	+
chr10	27195824	27198867	TCONS_00018143	+
chr7	65112777	65183632	INTS4L2	+
chr2	11534107	11543203	LINC00570	+
chr2	186031529	186085106	TCONS_00003961+TCONS_00003962+TCONS_00003963 +TCONS_00003964+TCONS_00004962+TCONS_0000496 3+TCONS_00003965	+
chr1	51442717	51443912	TCONS_00001494	−

chr13	100229366	100232622	TCONS_00021588	+
chr13	25498812	25501871	TCONS_l2_00007498	−
chr11	45029832	45063247	TCONS_00019285	+
chr11	61965679	61974030	TCONS_00019647	−
chr20	62023923	62026342	TCONS_00027982	+
chr4	185818075	185818980	TCONS_00008360	+
chr1	46899499	46911374	LOC729041	+
chr10	134957244	134958417	TCONS_l2_00003853	−
chr21	21629065	21631133	TCONS_00028879	−
chr10	37908136	37921834	TCONS_00018164+TCONS_00018165+TCONS_00018711	+
chr7	1499554	1503644	TCONS_00014055+TCONS_00012939	+
chr6	132452888	132454497	TCONS_00012277	−
chr9	14993325	15019722	LOC389705	+
chr13	48096508	48104202	TCONS_00021788	+
chr6	14394557	14404520	TCONS_00011270	+
chr5	175546552	175552168	LOC100507387	+
chr21	31581469	31584101	LINC00307	−
chr12	116716129	116716619	TCONS_00020579	+
chr1	46921495	46948147	TCONS_00000191	+
chr6	31141512	31145676	PSORS1C3	−
chr14	19650018	19695180	TCONS_l2_00008289+TCONS_l2_00008290+TCONS_l2_00008291+TCONS_l2_00008292+TCONS_l2_00008293+TCONS_l2_00008294+TCONS_l2_00008295+TCONS_l2_00008296	+
chr11	44540875	44542479	TCONS_00019625	−
chr16	80103298	80195993	TCONS_00024483	+
chr3	6673221	6847146	TCONS_l2_00019131+TCONS_l2_00019132+TCONS_l2_00020062+TCONS_l2_00020063+TCONS_l2_00020064+TCONS_l2_00020065+TCONS_l2_00020066+TCONS_l2_00020067+TCONS_l2_00019133+TCONS_l2_00020068	−
chr7	149714423	149745413	TCONS_l2_00026970	−
chr5	117911270	117917019	TCONS_00009495	+
chr1	91316660	91320704	TCONS_00001025+TCONS_00001026+TCONS_00000246	+
chr9	42771708	42782612	TCONS_l2_00029727	+
chr14	66908663	66927208	TCONS_00022772+TCONS_00023122	−
chr20	30073581	30075377	LINC00028	+
chr12	78790310	78876773	TCONS_00020488+TCONS_00020489	+
chr10	79938928	79973839	TCONS_00018764+TCONS_00018247	+
chr13	30492784	30500788	TCONS_00021616	−
chr9	100340	115953	TCONS_l2_00029159+TCONS_l2_00029161+TCONS_l2_00029886+TCONS_l2_00029887+TCONS_l2_00029162+TCONS_l2_00029163+TCONS_l2_00029888	−
chr18	558300	571069	TCONS_00026227	+
chr9	92768991	92777861	TCONS_00016063	+
chr18	77343316	77345492	TCONS_00026588	−
chr7	158743207	158750045	TCONS_00014242	+
chr2	60909746	60924733	TCONS_l2_00014590	−
chr1	86064814	86066417	TCONS_00000244	+

chr21	41097822	41098875	TCONS_00029023	+
chr13	68599998	68628150	TCONS_00021819+TCONS_00022191+TCONS_00022192 +TCONS_00021820	+
chr1	191190289	191197597	TCONS_00000359	+
chr1	228623044	228629593	TCONS_00001853	−
chr3	106991458	107041611	TCONS_00005805	−
chr1	143188828	143189695	TCONS_00000294	+
chr8	49428328	49429763	TCONS_00015000+TCONS_00015439	−
chr2	101355699	101363828	TCONS_00003793+TCONS_00003794+TCONS_00004858 +TCONS_00004859+TCONS_00003795	+
chr5	41284857	41295239	TCONS_00010312+TCONS_00010313+TCONS_00010919	−
chr15	59155581	59160321	TCONS_l2_00009127+TCONS_l2_00009128	−
chr6	26839266	26924333	GUSBP2	−
chr9	93867239	93880236	TCONS_00015846+TCONS_00016403+TCONS_00016404 +TCONS_00016405	−
chr12	59523275	59524642	TCONS_00020450	+
chr10	107249094	107580247	TCONS_l2_00003753+TCONS_l2_00003754+TCONS_ l2_00004263+TCONS_l2_00004264+TCONS_l2_00003755	−
chr7	98861890	98868672	TCONS_l2_00027183	+
chr8	1321358	1323312	TCONS_00014576+TCONS_00015205	+
chr1	173978405	173985344	TCONS_00000685	−
chr6	150642054	150643510	TCONS_00012332	−
chr1	100728863	100731676	TCONS_00001608+TCONS_00000587+TCONS_00001609	−
chr12	12849323	12853012	TCONS_00021074	+
chr6	72160537	72168577	TCONS_00011324	+
chr1	159436111	159453166	TCONS_00000319	+
chr6	3925562	3926428	TCONS_00012054	−
chr15	23649023	23652484	TCONS_00023297	+
chr7	124778629	124783823	TCONS_l2_00026180	+
chr7	134855676	134857224	TCONS_00014220	+
chr17	63096774	63115696	TCONS_00025460+TCONS_00025962+TCONS_00025 462+TCONS_00025461+TCONS_00025963+TCONS_0 0025463+TCONS_00025964+TCONS_00025464+TCO NS_00025465	+
chr2	86200673	86203652	TCONS_00003331	−
chr1	82680904	82726817	TCONS_00000554	−
chr2	96455402	96464841	TCONS_l2_00015403+TCONS_l2_00013805	+
chr22	16352061	16352999	TCONS_l2_00017539	+
chr6	125691357	125766329	TCONS_00011552+TCONS_00012272+TCONS_00011553	−
chrX	73070670	73072528	TCONS_00017433	+
chr4	185395886	185407002	TCONS_00008693+TCONS_00009191	−
chr10	94966811	94967747	TCONS_l2_00003176	+
chrX	139763271	139769870	TCONS_00017097	−
chr8	37417495	37423139	TCONS_00014674	+
chr1	198975170	198990166	TCONS_00000112	−
chr5	160358786	160365633	LOC285629	−
chr22	32723158	32739092	TCONS_00029472	−
chr5	73407471	73408058	TCONS_00009425	+
chr21	40346355	40349700	TCONS_00028933	−

chr9	127277217	127278335	TCONS_00015749	+
chr4	171196396	171201364	TCONS_00008643	−
chr22	46944748	46950100	TCONS_00029616	+
chr8	127643997	127651364	TCONS_00015165	−
chr19	16176493	16177108	TCONS_00026919+TCONS_00027491	+
chr1	101121835	101171700	TCONS_00001058	+
chr2	114426743	114430768	TCONS_00002985	+
chr11	80800493	80808462	TCONS_00019407	+
chr1	146644350	146648626	TCONS_00000054+TCONS_00002153	+
chr14	105532038	105540262	TCONS_00022898+TCONS_00022899+TCONS_00023178	−
chr15	37656400	37660936	TCONS_00023628	−
chr1	54881929	54886533	TCONS_00000201	+
chr5	7306622	7318373	TCONS_00009863	+
chr5	180634462	180644800	TCONS_00010589	−
chr1	840487	841186	TCONS_00000796	+
chr8	1244294	1250827	LOC286083	−
chr5	68477525	68484948	TCONS_00009286	−
chr1	22351707	22357715	LINC00339	+
chr10	62917130	62927455	TCONS_00018525	−
chr3	102379501	102392850	TCONS_00006565+TCONS_00005802+TCONS_00006566 +TCONS_00006567	−
chr15	70127573	70135306	LINC00593	+
chr8	67122577	67123886	TCONS_00015031	−
chr17	80340701	80341524	TCONS_00025785	−
chr10	95748253	95753461	TCONS_00018288	+
chr4	178514675	178544931	TCONS_00008348	+
chr1	68808088	68849925	TCONS_l2_00001282	−
chr19	51385352	51399654	KLKP1	−
chr3	126392790	126394236	TCONS_00006206	+
chr7	26109126	26170398	TCONS_00013740+TCONS_00013741+TCONS_00013743 +TCONS_00013742	−
chr4	180087670	180091375	TCONS_00007914+TCONS_00007915	−
chr6	125995499	126041364	LOC643623	+
chr7	127284471	127292066	TCONS_00013947+TCONS_00013252	−
chr6	112697490	112717623	TCONS_00012248+TCONS_00012771+TCONS_00012249 +TCONS_00012250+TCONS_00012251	−
chr5	76272216	76276874	TCONS_00010717	+
chr1	21737969	21739761	TCONS_l2_00001072	−
chr2	240832073	240834891	TCONS_00004627	−
chr1	18335986	18337010	TCONS_00001409	−
chr1	244938389	244944172	TCONS_00001900	−
chr4	27219101	27283847	TCONS_00007495	+
chr5	17130137	17217531	LOC285696	−
chr11	72911525	72916441	TCONS_00019383+TCONS_00019384	+
chr2	122978162	123006408	TCONS_00003842	+
chr1	88365592	88442197	TCONS_00000558+TCONS_00001561+TCONS_00001562	−
chr3	182699182	182704748	TCONS_00006328	+
chr3	11104002	11113767	TCONS_00005945	+

chr18	32902395	32906945	TCONS_00026319	+
chr1	30486799	30510459	TCONS_00000496+TCONS_00000079	−
chr2	154277040	154306334	TCONS_l2_00014951+TCONS_l2_00015996	−
chr17	48358938	48365239	TCONS_00025237+TCONS_00025694+TCONS_00025695	−
chr2	177042909	177043737	TCONS_00003056	+
chr17	53527224	53536223	TCONS_00025697	−
chr1	166304121	166308626	TCONS_00000328+TCONS_00001187	+
chr2	37827040	37839573	TCONS_00003638+TCONS_00003639+TCONS_00003640	+
chr12	115451715	115532938	TCONS_00020576	+
chr22	42834029	42846772	TCONS_00029431+TCONS_00029604+TCONS_00029821	+
chr5	72678458	72707071	TCONS_l2_00022365	+
chr3	129672948	129688137	TCONS_00005827	−
chr2	56301789	56317511	TCONS_00002752	−
chr4	1031541	1045258	TCONS_00007952	+
chr4	165928238	165929111	TCONS_00007891	−
chr9	1041219	1042520	TCONS_00015785	−
chr6	159774954	159775705	TCONS_00011604	−
chr6	170761923	170762322	TCONS_l2_00025060	−
chr10	103070775	103071262	TCONS_00017859	+
chr16	23031684	23035511	TCONS_00024350	+
chr3	99219191	99224352	TCONS_00005560	+
chr2	137086863	137087505	TCONS_l2_00014930+TCONS_l2_00014931+TCONS_ l2_00014932	−
chr8	143279717	143290364	LINC00051	+
chr7	141212323	141229061	TCONS_00013982+TCONS_00013984+TCONS_00013983 +TCONS_00014425+TCONS_00013985	−
chr7	181484	182694	TCONS_00013671	−
chr5	65702782	65866254	TCONS_00009983	+
chrX	97098492	97101678	TCONS_l2_00030270	+
chr9	106723071	106726064	TCONS_00016450	−
chr4	128018046	128391724	TCONS_l2_00021964+TCONS_l2_00021287+TCONS_ l2_00021965+TCONS_l2_00021288+TCONS_l2_00021966	−
chr9	32679841	32705364	TCONS_00016289	−
chr1	119870799	119873438	TCONS_00000287	+
chr3	164388666	164403864	TCONS_00005874	−
chr5	21138915	21172730	TCONS_00010281	−
chr12	11700964	11717335	LOC338817	+
chr8	39267715	39274897	TCONS_00014981	−
chr1	154442113	154445591	TCONS_00002485	−
chr18	76555423	76558441	TCONS_00026382	+
chr7	74047827	74048200	TCONS_00012882	+
chr9	133028065	133072354	TCONS_l2_00029085	+
chr22	23395711	23396939	TCONS_00029526	+
chr15	22525616	22527776	TCONS_00023572	−
chr7	45034628	45038116	TCONS_00014119	+
chrY	23745486	23756552	TTTY13	−
chr12	43081007	43101757	TCONS_00020384	+
chr1	36127392	36130797	TCONS_00001454	−

chr13	31377343	31384782	LINC00398	+
chr9	34985498	34989275	TCONS_00016298	−
chr7	91005392	91186163	TCONS_00013884+TCONS_00013205	−
chr3	167110584	167126307	TCONS_00005644	+
chr12	10725617	10727581	TCONS_00021064	+
chr16	47007717	47012770	TCONS_00024378	+
chr9	102347480	102582301	TCONS_00016440+TCONS_00016441+TCONS_00015632+TCONS_00016442+TCONS_00016847+TCONS_00016443+TCONS_00016444	−
chr7	155058857	155059762	TCONS_00014236+TCONS_00013101	+
chrY	9528709	9531308	TTTY8	+
chr21	38666996	38668774	TCONS_00029013	+
chr8	122654339	122655444	TCONS_00015151	−
chr8	11225911	11296166	C8orf12	+
chr8	37455062	37456790	TCONS_00014977	−
chr21	23027359	23058649	TCONS_00028881	−
chr14	95024730	95027008	TCONS_00022841	−
chr9	110779853	110810255	TCONS_l2_00028987	+
chrX	119370309	119379122	NKAPP1	−
chr15	40780324	40800938	TCONS_00023376	+
chr6	53247135	53247532	TCONS_00011503	−
chr9	67327421	67340215	TCONS_l2_00029332+TCONS_l2_00029333+TCONS_l2_00029334+TCONS_l2_00029335+TCONS_l2_00029336+TCONS_l2_00029337+TCONS_l2_00029338+TCONS_l2_00029339+TCONS_l2_00029340	−
chr17	18353521	18354072	TCONS_l2_00010595	+
chr14	100635055	100638515	TCONS_00022874	−
chr3	13974553	13978444	FGD5P1	+
chr9	107854176	107881755	TCONS_00016111	+
chr11	3602162	3602848	TCONS_l2_00004897	−
chr3	49838879	49839621	TCONS_00006508	−
chr12	55803574	55808808	TCONS_l2_00006264	−
chr2	231751261	231769232	LOC151484	+
chr4	152588376	152591654	TCONS_00007658+TCONS_00008904	+
chr21	26499782	26507561	TCONS_00028816	+
chr19	30185930	30187465	TCONS_00027288+TCONS_00027289	−
chr9	34889060	34895782	TCONS_l2_00029251	−
chr10	35104695	35105314	TCONS_00017812	+
chr18	2167794	2170196	TCONS_00026232	+
chr3	156535040	156536805	TCONS_00006285	+
chr7	56646792	56666464	TCONS_l2_00025889	+
chr5	12837290	12862122	TCONS_00010272	−
chr3	168720862	168723254	TCONS_00005655	+
chr2	216773381	216777417	TCONS_00004014	+
chr8	70041903	70043442	TCONS_l2_00027830+TCONS_l2_00027831	+
chr22	32772651	32780329	LOC339666	+
chr2	132384450	132384816	TCONS_l2_00014908	−
chr10	2208526	2211654	TCONS_00017758	+

chr9	10613202	10618218	TCONS_00015648	+
chr11	46277871	46292165	TCONS_00019635	−
chr5	179719195	179722253	TCONS_00010210+TCONS_00009562	+
chr12	50305565	50324095	TCONS_00020419+TCONS_00020187+TCONS_00020420 +TCONS_00020421	+
chr1	229916291	229926495	TCONS_00001856	−
chr1	95546646	95550539	TCONS_00001045	+
chr12	17144476	17144926	TCONS_l2_00005546	+
chr20	25744102	25781927	FAM182B	−
chr9	110267407	110277663	TCONS_00016120+TCONS_00016121+TCONS_00016122 +TCONS_00016666	+
chr10	89584477	89601145	TCONS_00018994	−
chr8	144507227	144511408	TCONS_00014883	+
chrY	58838501	58842077	TCONS_00017657	+
chr16	14503947	14512732	TCONS_00024590+TCONS_00024591	−
chr1	24863138	24882323	TCONS_00000487+TCONS_00002307+TCONS_00001429	−
chr2	3751407	3891877	TCONS_l2_00013440	+
chr5	62597333	62597863	TCONS_l2_00022340	+
chr12	70612843	70619160	TCONS_l2_00006311+TCONS_l2_00006312+TCONS_ l2_00006313+TCONS_l2_00006314	−
chr2	33152194	33171202	LOC100271832	+
chr19	22798361	22806773	TCONS_00026805	−
chr8	20831497	20852630	LOC286114	+
chr14	59546412	59590964	TCONS_00022523	+
chr11	81807520	82115382	TCONS_l2_00005138	−
chr9	125163249	125165185	TCONS_00015879	−
chr20	54039581	54084752	TCONS_00027956+TCONS_00027957	+
chr13	47029338	47035293	TCONS_l2_00007398+TCONS_l2_00006838	+
chr2	132394598	132407188	TCONS_l2_00015488+TCONS_l2_00013996+TCONS_ l2_00013997	+
chrX	21911718	21929813	TCONS_00016971+TCONS_00017411	+
chr2	48357865	48392255	TCONS_00003672	+
chr1	25907969	25916875	TCONS_00000169+TCONS_00000868	+
chr4	41874781	41884628	TCONS_00008475+TCONS_00007751+TCONS_00007421 +TCONS_00007420	−
chr11	34876859	34880602	TCONS_00019614	−
chr1	224284517	224298709	TCONS_00001839	−
chr20	18485234	18485738	TCONS_l2_00016171	+
chr1	134773	140566	LOC729737	−
chr7	1306989	1308903	TCONS_00012938	+
chr20	7328114	7349144	TCONS_00027858	+
chr2	233285371	233286171	TCONS_00003513	−
chr2	38767123	38769283	TCONS_00004212	−
chr20	61669363	61685215	LOC100144597	+
chr16	72425087	72463966	TCONS_00025047+TCONS_00024721	−
chr5	64782271	64782875	TCONS_00009677	−
chr6	31409444	31414750	TCONS_00011289	+
chr14	19935905	19974840	TCONS_l2_00008349	−

chr21	35779935	35780677	TCONS_00029006	+
chr5	102779412	102834870	TCONS_l2_00023847	−
chr11	2016406	2019065	H19	−
chr13	39783260	39790406	TCONS_00021973	−
chr2	108370568	108438585	TCONS_00002974+TCONS_00002975	+
chr19	56035725	56037773	TCONS_00027424	−
chr1	185527502	185597659	TCONS_00002542+TCONS_00002543+TCONS_00000109+TCONS_00001758+TCONS_00001759+TCONS_00000110+TCONS_00000700+TCONS_00002544	−
chr5	72509774	72590761	TCONS_00009417+TCONS_00009418+TCONS_00009419+TCONS_00009420+TCONS_00009421	+
chr1	35081180	35083207	TCONS_00001451	−
chr8	102326509	102328921	TCONS_00014813	+
chr2	692083	693235	TCONS_00003144	−
chr5	82146852	82155888	TCONS_00010392	−
chr1	232447238	232448857	TCONS_00000418	+
chr14	103550492	103556344	TCONS_l2_00007925	+
chr11	133902167	133911236	LOC100128239	+
chr2	153032565	153039439	TCONS_00003905	+
chr4	83534266	83542590	LINC00575	−
chr11	119758324	119804313	TCONS_00019500+TCONS_00020005	+
chr2	155548248	155553147	TCONS_l2_00014953	−
chr3	39219384	39223013	TCONS_00005989+TCONS_00005990	+
chr5	55354877	55363199	TCONS_00009403	+
chr22	20186253	20192441	LOC284865	−
chr2	64503035	64503985	TCONS_00004284	−
chr2	220594832	220602464	TCONS_00003111	+
chr3	166647134	166674718	TCONS_00006705	−
chr10	48497449	48499077	TCONS_00018499	−
chr11	125976783	125979103	TCONS_00019525	+
chr17	36309310	36325402	TCONS_l2_00011199	−
chr1	195727482	195732530	TCONS_00000709	−
chr2	45875882	45877964	TCONS_00004240	−
chrX	36011376	36033116	TCONS_00017150+TCONS_00017413	+
chr17	8084794	8086775	TCONS_00025294	+
chr22	28071819	28110959	TCONS_00029667+TCONS_00029464	−
chr10	2488447	2544356	TCONS_00017899+TCONS_00017900+TCONS_00017901+TCONS_00018400+TCONS_00018401+TCONS_00018864+TCONS_00018865+TCONS_00018866+TCONS_00018867+TCONS_00018402+TCONS_00018403	−
chr13	95141147	95143272	TCONS_00022085	−
chr2	86042253	86053911	LOC284950	+
chr8	11434044	11438850	LINC00208	+
chr10	36185802	36187891	TCONS_00017953	−
chr9	46359242	46380257	TCONS_00015818	−
chr9	82489611	82501593	TCONS_00015838	−
chr13	111608813	111612429	TCONS_00022117	−
chr18	53665694	53696360	TCONS_00026337+TCONS_00026338	+
chr18	42157985	42159894	TCONS_00026506	−

chr5	4452043	4868256	TCONS_l2_00023649+TCONS_l2_00022761+TCONS_l2_00023650+TCONS_l2_00022762	−
chr17	68884337	68884918	TCONS_00025745	−
chr7	76592343	76606972	TCONS_l2_00026696	−
chr17	21825370	21826499	FAM27L	+
chr2	91874128	91900136	TCONS_l2_00015860+TCONS_l2_00014688+TCONS_l2_00014689	−
chr12	128399955	128436097	LINC00507	+
chr7	19039116	19095789	TCONS_00013713+TCONS_00014261+TCONS_00013714	−
chr7	55807836	55826773	TCONS_l2_00026575+TCONS_l2_00026576+TCONS_l2_00026577	−
chr10	58058713	58074528	TCONS_00018206+TCONS_00018732	+
chr15	82318361	82322412	TCONS_00023759+TCONS_00023760	−
chr14	62037258	62121431	FLJ22447	+
chr7	56680291	56683113	TCONS_00013453+TCONS_00013454	+
chr19	37176046	37178351	TCONS_l2_00013085+TCONS_l2_00013086+TCONS_l2_00013384+TCONS_l2_00013385	−
chr16	34765218	34766124	TCONS_00024636	−
chr6	156632038	156684785	TCONS_00012619+TCONS_00012001+TCONS_l2_00025276	+
chr2	195208993	195284355	TCONS_l2_00015562+TCONS_l2_00015563+TCONS_l2_00015564+TCONS_l2_00014172+TCONS_l2_00014173	+
chr19	50472912	50479076	SIGLEC16	+
chr7	45763386	45808617	SEPT7P2	−
chr5	1040858	1048427	TCONS_00010232+TCONS_00010873+TCONS_00010233+TCONS_00010234+TCONS_00010235+TCONS_00010874+TCONS_00010236+TCONS_00010237+TCONS_00010875+TCONS_00010876+TCONS_00010238+TCONS_00010877+TCONS_00010878+TCONS_00010879+TCONS_00010239+TCONS_00010240	−
chr3	186156944	186175019	TCONS_00006341+TCONS_00006342+TCONS_00007056+TCONS_00007057+TCONS_00007058+TCONS_00005680+TCONS_00006343+TCONS_00005681+TCONS_00005682+TCONS_00005683+TCONS_00007059	+
chr5	97421964	97496897	TCONS_00009733	−
chr9	85068260	85070166	TCONS_00015701+TCONS_00016624	+
chr19	29704189	29706659	TCONS_00026977+TCONS_00026978+TCONS_00026979	+
chr6	12322520	12335036	TCONS_00011696	+
chr13	112976881	112985539	TCONS_00021699	−
chr18	10371396	10372540	TCONS_00026416	−
chr12	108296927	108297548	LOC728739	−
chr4	149948597	149983674	TCONS_00007871	−
chr10	973603	988855	TCONS_l2_00002834+TCONS_l2_00002835+TCONS_l2_00002836+TCONS_l2_00002837+TCONS_l2_00003861+TCONS_l2_00002838+TCONS_l2_00003862	+
chr13	44974184	44980091	TCONS_00022267+TCONS_00021511+TCONS_00021986+TCONS_00022268	−
chr1	149591179	149592306	TCONS_00000305	+
chr22	48535278	48536818	TCONS_00029493	−
chr10	46737613	48952629	BMS1P1	−
chr22	18512151	18520734	FLJ41941	+

chr1	48154135	48169113	TCONS_l2_00002041+TCONS_l2_00000270	+
chr14	38761469	38780995	TCONS_00022480	+
chr8	55971997	55975518	TCONS_00014701	+
chr12	125229280	125229642	TCONS_00020614	+
chr15	41198496	41202517	TCONS_00023377+TCONS_00023378+TCONS_00023222 +TCONS_00023924+TCONS_00023379	+
chr4	184434393	184478022	TCONS_00008949	+
chr4	128015586	128017878	TCONS_00008571+TCONS_00008572	−
chr22	30101245	30115848	TCONS_00029466+TCONS_00029467+TCONS_00029468	−
chr2	121300485	121301902	TCONS_00003390	−
chr15	63383481	63402781	TCONS_00023960+TCONS_00023439	+
chr11	94464169	94476109	TCONS_00019977+TCONS_00019978	+
chr9	90892189	90893536	TCONS_00016396	−
chr17	21516455	21548969	TCONS_00025335+TCONS_00025867	+
chr5	693675	785669	TCONS_l2_00022134	+
chrY	24585740	24587606	TTTY6	−
chr11	3210776	3211159	TCONS_00019136	+
chr8	144795188	144796371	TCONS_00015544	−
chr13	65214335	65216446	TCONS_00022046	−
chr13	81278918	81280841	TCONS_00022066	−
chr3	39018393	39024793	TCONS_00006482+TCONS_00006483	−
chr3	31496782	31497222	TCONS_l2_00018492	+
chr8	22536669	22537772	TCONS_00014950	−
chr10	26153169	26171989	TCONS_00018141	+
chr13	22446935	22452299	LINC00424	−
chr5	17404005	17442654	TCONS_00010633+TCONS_00010634+TCONS_0 0010635+TCONS_00009358+TCONS_00009886+ TCONS_00009887+TCONS_00009359+TCONS_0 0010636+TCONS_00010637+TCONS_00009888+- TCONS_00009360+TCONS_00010638	+
chr1	169073362	169074145	TCONS_00000330	+
chr10	60001	60544	TCONS_00017894	−
chr17	66201329	66208783	TCONS_l2_00010942+TCONS_l2_00010943	+
chr22	24643891	24646939	TCONS_l2_00017980+TCONS_l2_00017981	−
chr16	71466770	71467279	TCONS_00024718	−
chr17	59475313	59476820	TCONS_00025946	+
chr16	51680005	51680947	TCONS_l2_00009739	+
chr3	27753781	27755870	TCONS_00005969+TCONS_00005970+TCONS_00005971	+
chr15	20505566	20531084	TCONS_00023846+TCONS_00023287+TCONS_00023288 +TCONS_00023847	+
chr12	11583172	11588092	TCONS_00021291+TCONS_00020720	−
chr8	31133101	31179420	TCONS_00014665+TCONS_00014666	+
chr5	164662759	164668941	TCONS_00010542+TCONS_00010543+TCONS_00011081	−
chrX	1515320	1518295	TCONS_00017028+TCONS_00017029	−
chr5	123428531	123472967	TCONS_00010459+TCONS_00010460	−
chr9	128507381	128508398	TCONS_00016158	+
chr10	47654473	47657410	TCONS_l2_00003545	−
chr12	26097795	26103421	TCONS_00020735+TCONS_00020736	−
chr17	10698230	10707416	LINC00675	−

chr3	166641738	166643655	TCONS_00006704	−
chr20	24071567	24092596	TCONS_00028348	−
chr22	30871275	30875210	TCONS_00029793	+
chr2	235017360	235018125	TCONS_00003127	+
chr16	24247404	24249367	TCONS_00024351	+
chr17	8670878	8672155	TCONS_00025302	+
chr18	10626221	10628526	TCONS_00026673	−
chr6	168643618	168663261	TCONS_00011418	+
chr21	16133802	16135509	TCONS_00029079+TCONS_00029080	−
chr4	189096293	189099524	TCONS_00008379	+
chr20	30691028	30694658	TCONS_00028365	−
chr19	23171145	23186022	TCONS_l2_00012946	−
chr17	3608266	3608812	TCONS_00025531	−
chr1	67987996	67998015	TCONS_00002051+TCONS_00002052+TCONS_00002053+TCONS_00000971+TCONS_00002054+TCONS_00000219	+
chr19	56399226	56401109	TCONS_00027115	+
chr8	93681177	93695674	TCONS_00014793+TCONS_00014794	+
chr6	16126537	16129130	TCONS_00012092	−
chr5	180632361	180633120	TCONS_00009825	−
chr2	105197279	105198348	TCONS_00003351	−
chr22	17350368	17352016	TCONS_00029359	+
chr2	102661125	102674449	TCONS_00003801	+
chr10	3574286	3579223	TCONS_00017904+TCONS_00017905	−
chr7	112256535	112257317	TCONS_00013564	+
chrX	100258821	100262956	TCONS_00017001	+
chr10	38717074	38741081	LOC399744	+
chr19	5482069	5487673	TCONS_00027165	−
chr10	49291426	49294910	TCONS_l2_00003561	−
chr8	142524044	142528696	TCONS_l2_00028009	+
chr15	48080272	48083451	TCONS_00023668	−
chr2	132783681	132796342	TCONS_l2_00014911+TCONS_l2_00014912	−
chr15	78423909	78439502	TCONS_00023472	+
chr14	70278479	70283285	TCONS_00022543	+
chr21	35303516	35343487	LINC00649	+
chr6	17587064	17587418	TCONS_l2_00024066	+
chr1	164879222	164881715	TCONS_00001710	−
chr10	134824440	134825383	TCONS_00018653+TCONS_00018654+TCONS_00018655+TCONS_00019067+TCONS_00019066+TCONS_00018656+TCONS_00018657	−
chr7	130033937	130035446	TCONS_00013589	+
chr18	70951946	70953165	TCONS_00026557	−
chr6	22589366	22594062	TCONS_00011467	−
chr19	14903114	14903565	TCONS_00026907	+
chr16	81110924	81112455	TCONS_00024295	+
chr5	33162227	33163075	TCONS_l2_00022249	+
chr2	74622325	74640230	TCONS_l2_00014653+TCONS_00004313	−
chr13	52741845	52768602	MRPS31P5	−

chr4	90412098	90412901	TCONS_00008164	+
chr7	100106988	100117666	TCONS_00013556+TCONS_00013052	+
chr3	2139211	2139848	TCONS_00006396	−
chr3	152487185	152517694	TCONS_00006280	+
chr11	104071253	104112755	TCONS_00019456	+
chr5	44742520	44808883	TCONS_l2_00022885+TCONS_l2_00022886+TCONS_l2_00023727+TCONS_l2_00023728+TCONS_l2_00022887+TCONS_l2_00023729+TCONS_l2_00022888+TCONS_l2_00023730	−
chr2	22421608	22422083	TCONS_00004715	+
chr6_apd_hap1	4131253	4134818	TCONS_00029940	+
chr10	102950527	102953866	TCONS_00018799+TCONS_00018311	+
chr16	29538929	30317244	LOC440354	−
chr1	134767	139723	TCONS_l2_00000001+TCONS_l2_00000002+TCONS_l2_00001922+TCONS_l2_00001923	+
chr3	24141465	24144738	LINC00691	−
chr5	72742184	72742811	TCONS_00009423	+
chr9	42844370	67032072	LOC286297	−
chr8	30044817	30047775	TCONS_00014557	−
chr8	67094960	67099083	TCONS_00015029	−
chr2	145281131	145282228	TCONS_00004465+TCONS_00004466+TCONS_00004467	−
chr14	29488159	29566484	TCONS_00022453+TCONS_00022454+TCONS_00022944+TCONS_00022455	+
chr22	34672765	34680814	TCONS_00029686+TCONS_00029687	−
chr1	95940293	95944912	FLJ31662	+
chr17	21760936	21792968	TCONS_00025219+TCONS_00025593+TCONS_00025594	−
chr11	69229075	69231600	TCONS_00019101	+
chr6	169252754	169256278	TCONS_00012365	−
chr2	19296231	19299842	TCONS_00004704	+
chr6	25937562	25938029	TCONS_00011741	+
chr8	142121974	142123085	TCONS_00014876	+
chr10	30569015	30569839	TCONS_00017944	−
chr5	8835495	8840502	TCONS_00010265	−
chr9	139155771	139159083	TCONS_00015778	+
chr10	132102128	132102815	TCONS_00018389	+
chr15	23187729	23208357	WHAMMP3	−
chr7	156225217	156228527	TCONS_00013278	−
chr4	176157751	176159457	TCONS_00008663	−
chr19	15962877	15965591	TCONS_l2_00012889	−
chr10	72689682	72691814	TCONS_00017840	+
chr5	157929835	157930377	TCONS_00010150	+
chr1	222763273	222766374	TCONS_00000396+TCONS_00000397	+
chr20	23778778	23786300	TCONS_00027915	+
chr18	37646250	37679196	TCONS_00026191	+
chr15	62929371	62937380	MGC15885	−
chr14	50328594	50331183	TCONS_00022740	−
chr12	93397534	93771512	LOC643339	−
chr11	72866936	72873599	TCONS_00019701	−

chr5	42157044	42157966	TCONS_00010676	+
chr13	85446167	85450526	TCONS_00022071	−
chr15	74346640	74348558	TCONS_00023980	+
chr16	3661780	3693096	TCONS_00024800	+
chr9	19461158	19464522	TCONS_l2_00029905	−
chr10	22533728	22542978	TCONS_00018450	−
chr9	100153119	100158973	LOC286359	−
chr2	127475776	127511762	TCONS_00004880+TCONS_00003851	+
chr1	196014057	196129619	TCONS_00001767+TCONS_00000710	−
chr10	44396813	44407926	TCONS_00017961+TCONS_00018491+TCONS_00018926 +TCONS_00018927+TCONS_00018492	−
chr1	142785222	142891271	TCONS_l2_00001486+TCONS_l2_00001487+TCONS_ l2_00002625+TCONS_l2_00001488+TCONS_ l2_00002626+TCONS_l2_00001489+TCONS_ l2_00001490+TCONS_l2_00001491+TCONS_ l2_00001492+TCONS_l2_00001493+TCONS_l2_00001494	−
chr16	14096241	14109875	TCONS_00024585+TCONS_00024586+TCONS_00024587 +TCONS_00024588	−
chr9	84354041	84370367	TCONS_00016024+TCONS_00015699	+
chr8	64619954	64655523	TCONS_00015286+TCONS_00014736	+
chr15	30763429	30782651	TCONS_l2_00008517+TCONS_l2_00008518+TCONS_ l2_00008519+TCONS_l2_00008520+TCONS_ l2_00008521+TCONS_l2_00009422+TCONS_ l2_00008522+TCONS_l2_00008523	+
chr17	20978869	21020917	TCONS_00025162+TCONS_00025863+TCONS_00025864 +TCONS_00025865	+
chr9	128506033	128508019	TCONS_00015880	−
chr3	18735379	18959184	TCONS_00005496+TCONS_00005497	+
chr15	40213228	40218031	TCONS_00023920+TCONS_00023363+TCONS_00023364 +TCONS_00023260	+
chr9	90441425	90452141	TCONS_l2_00030001	−
chr3	167967310	168548374	EGFEM1P	+
chr13	77174564	77303640	TCONS_00022053	−
chr10	5658825	5660124	TCONS_00017914	−
chr18	53727807	53735555	TCONS_00026719	−
chr4	38511388	38519677	TCONS_00007515	+
chr3	19717560	19729972	TCONS_00006454	−
chr7	91268162	91272827	TCONS_00013206	−
chr14	64812191	64814329	TEX21P	−
chr6	134957627	134963633	TCONS_00011377	+
chr10	107899924	107959138	TCONS_00017716+TCONS_00017863+TCONS_00017864 +TCONS_00017865+TCONS_00017866	+
chr3	125984829	125994041	TCONS_00006633+TCONS_00006632+TCONS_00007248 +TCONS_00007249	−
chr10	8093127	8095448	TCONS_00018677+TCONS_00017782+TCONS_00018678	+
chr10	47279192	47289833	TCONS_00017972+TCONS_00017973	−
chr5	98860221	98913717	TCONS_l2_00022453+TCONS_l2_00022454+TCONS_ l2_00023492	+
chr3	155572513	155581520	TCONS_00007014+TCONS_00006284+TCONS_00007015	+
chr10	87339779	87340216	TCONS_00018560	−

chr9	141090383	141093775	TCONS_00015898	−
chr7	10000651	10021444	TCONS_00012949	+
chrY	27524447	27540866	TCONS_l2_00031062	−
chr9	139141825	139166993	TCONS_00016506+TCONS_00016894+TCONS_00016895+TCONS_00016507+TCONS_00016508+TCONS_00016896+TCONS_00016897+TCONS_00016898+TCONS_00016509+TCONS_00016510+TCONS_00016899+TCONS_00016900+TCONS_00016901+TCONS_00016511+TCONS_00016902+TCONS_00016512	−
chr21	45595372	45596336	TCONS_00028854	+
chr18	54712815	54716542	TCONS_00026340	+
chr14	96181820	96223116	TCONS_00022357	+
chr12	68825627	68845357	TCONS_00021346+TCONS_00021345+TCONS_00021344+TCONS_00021343+TCONS_00021347	−
chr9	111014434	111017100	TCONS_00015868	−
chr12	90365931	90460893	TCONS_00020511	+
chr6	153860947	153862090	TCONS_00011994	+
chr4	171502621	171526135	TCONS_l2_00022045	−
chr19	32456553	32516901	TCONS_00027296+TCONS_00027726+TCONS_00027297	−
chr16	62630276	62632353	TCONS_00024292	+
chrY	58912269	58915039	TCONS_00017659+TCONS_00017660+TCONS_00017661	+
chr8	129417516	129440162	TCONS_00014536	−
chr7	45228588	45232084	TCONS_00014121	+
chr1	6784697	6790072	TCONS_00000147+TCONS_00000148+TCONS_00001934+TCONS_00000149	+
chr7	33832780	33842768	TCONS_00013152+TCONS_00013153	−
chr1	59597608	59612479	HSD52	−
chr2	68589206	68591456	TCONS_00003728	+
chr16	85979025	85984351	TCONS_00024489	+
chr3	104536326	104626299	TCONS_00006568	−
chr14	87630872	87646829	TCONS_00022582+TCONS_00022583	+
chr4	149944324	149945305	TCONS_00008287	+
chr4	110224191	110237291	TCONS_00007592	+
chr12	76704292	76709665	TCONS_l2_00005763	+
chr19	23086798	23097056	TCONS_00027231	−
chr9	110381806	110384012	TCONS_00016125+TCONS_00016668	+
chr16	213181	215176	TCONS_l2_00009549	+
chr4	183795492	183798859	TCONS_00009180+TCONS_00009181+TCONS_00009182	−
chr5	55744362	55777773	TCONS_00010339+TCONS_00010340+TCONS_00010341+TCONS_00009668+TCONS_00009669	−
chr18	69444968	69465957	TCONS_00026360	+
chr16	468211	471156	TCONS_00024531	−
chr7	6120734	6132716	TCONS_00012943+TCONS_00012944+TCONS_00014068+TCONS_00012945	+
chr19	48824598	48826469	TCONS_00027066+TCONS_00027589+TCONS_00027590+TCONS_00027067	+
chrY	15271184	15273460	TCONS_l2_00031044	−
chr4	140522357	140527570	TCONS_00008592	−
chr12	10741446	10742144	TCONS_00021065	+

chr1	198776622	198796345	TCONS_00000712	−
chr18	32523190	32523644	TCONS_00026485	−
chr13	20137464	20138901	TCONS_l2_00007059	−
chr6_mann_hap4	4301449	4305045	TCONS_00030000	+
chr17	60980515	60982300	TCONS_00025456	+
chr8	67331822	67341212	LOC100505676	−
chr7	149697841	149702213	TCONS_00013093	+
chr12	125765710	125774685	TCONS_00020616	+
chr3	67346724	67356783	TCONS_00005535+TCONS_00005536	+
chr5	180261303	180262712	TCONS_00011109	−
chr7	39773167	39834222	LINC00265	+
chr3	112861197	112886666	TCONS_00005819	−
chr16	9595636	9612183	TCONS_00024325+TCONS_00024326	+
chr1	220605800	220608985	TCONS_00001283	+
chrX	38639280	38659679	TCONS_00017307	−
chr13	87564173	87589062	TCONS_00021581	+
chr6	166253092	166253578	TCONS_00012358	−
chr9	138469277	138472027	TCONS_00016503	−
chr5	57362439	57364565	TCONS_00010348	−
chr12	19189967	19201653	TCONS_l2_00005549	+
chr9	75797125	75799444	TCONS_00016355	−
chr3	193788002	193826963	TCONS_00006354+TCONS_00007061+TCONS_00007062+TCONS_00006355+TCONS_00007063+TCONS_00006356+TCONS_00005697	+
chr15	41455315	41456708	TCONS_00023647	−
chr1	73240864	73256963	TCONS_00000983+TCONS_00000227	+
chr17	66179199	66185240	TCONS_00025970+TCONS_00025471+TCONS_00025971	+
chr14	22191912	22192543	TCONS_l2_00007617	+
chr1	46702304	46705490	TCONS_00000926	+
chr2	78245158	78517815	TCONS_00002756	−
chr5	710470	767576	TCONS_l2_00022720+TCONS_l2_00023627+TCONS_l2_00022721+TCONS_l2_00022722+TCONS_l2_00022723+TCONS_l2_00022724	−
chr10	83633135	83634234	TCONS_00018558	−
chr18	73649664	73650179	TCONS_00026569	−
chr7	54643745	54646487	TCONS_00012908	−
chr3	146639710	146643005	TCONS_00005610+TCONS_00005611	+
chr7	108549510	108592723	TCONS_00013057	+
chr1	62102824	62113990	TCONS_00000968	+
chr6	47277791	47280162	TCONS_00011823	+
chr4	174438541	174439852	TCONS_00008935	+
chrX	10210379	10333363	TCONS_00016964	+
chr10	120001007	120027222	TCONS_00018827+TCONS_00018828+TCONS_00018830+TCONS_00017873+TCONS_00018351+TCONS_00017874+TCONS_00017875+TCONS_00017876+TCONS_00018352+TCONS_00018831	+
chr1	178621017	178631642	TCONS_00001208	+
chr11	81674331	81682373	TCONS_00019958	+

chr11	130650471	130654253	TCONS_00019806	−
chr7	63466642	63478469	TCONS_l2_00025928	+
chr20	24911284	24913619	TCONS_00028028	−
chr11	42275392	42283462	TCONS_00019282	+
chr5	6773581	6776088	TCONS_00009860	+
chr12	92860455	92877562	TCONS_00020193+TCONS_00021186+TCONS_00021187	+
chr14	69328473	69329425	TCONS_l2_00007785+TCONS_l2_00007786	+
chr6	16226470	16227830	TCONS_00012093	−
chr20	38283980	38289630	TCONS_00028667+TCONS_00028386+TCONS_00028387 +TCONS_00028668	−
chr6	170209414	170210198	TCONS_00011628	−
chr4	93103767	93104992	TCONS_l2_00021217	−
chr2	73533090	73534993	TCONS_00005177	−
chr22	16373081	16377057	TCONS_00029329	+
chr5	86042635	86045588	TCONS_00009253+TCONS_00009439+TCONS_00010733	+
chr10	103503658	103526776	TCONS_00018590+TCONS_00019012+TCONS_00018591	−
chr2	20314686	20317929	TCONS_l2_00013502	+
chr5	51971361	51985880	TCONS_00010330	−
chr13	45457978	45464410	TCONS_00021987+TCONS_00021988+TCONS_00022269 +TCONS_00021989+TCONS_00021990	−
chr2	43324884	43370236	TCONS_00003266+TCONS_00004229+TCONS_00004230	−
chr15	24219700	24224356	TCONS_00023301	+
chr10	122938214	122945480	TCONS_00017882	+
chr11	2629558	2721228	KCNQ1OT1	−
chr10	44124265	44170147	ZNF32-AS3	+
chr22	16915159	16940793	TCONS_l2_00017857	−
chr12	126478716	126486554	TCONS_00020969	−
chr5	24882052	24885570	TCONS_00009620	−
chr22	22012017	22016642	TCONS_l2_00017631+TCONS_l2_00017632+TCONS_ l2_00017633+TCONS_l2_00017634	+
chr19	22085706	22101719	TCONS_00026952	+
chr7	129964184	129971373	TCONS_00014212	+
chr3	16566329	16573120	TCONS_00005953+TCONS_00006794	+
chr17	72549868	72554633	TCONS_00025765	−
chr2	122536828	122567536	TCONS_l2_00013946+TCONS_l2_00015466+TCONS_ l2_00015467+TCONS_l2_00015468+TCONS_ l2_00013947+TCONS_l2_00013948	+
chr22	36068836	36085420	TCONS_00029586+TCONS_00029418	+
chr10	131581117	131584811	TCONS_00017891	+
chr1	209541007	209553165	TCONS_l2_00002292+TCONS_l2_00000794+TCONS_ l2_00002293	+
chr16	21238729	21244853	TCONS_00024600	−
chr6	168595038	168605181	TCONS_00011416	+
chr19	22674956	22676132	TCONS_00026954	+
chr17	43654822	43676339	TCONS_l2_00011287+TCONS_l2_00011288+TCONS_ l2_00011289	−
chr3	148164073	148173245	TCONS_00006679	−
chr3	196417084	196420127	TCONS_00007086	+
chr9	22646199	22824212	FLJ35282	+

chr16	86716081	86730051	TCONS_00024495+TCONS_00024897+TCONS_00024496	+
chr22	32555441	32556379	TCONS_00029471	−
chr12	54026622	54036661	TCONS_l2_00006239+TCONS_l2_00006240	−
chr4	1553726	1554076	TCONS_00008407	−
chr1	234663637	234667525	LOC100506795	−
chr2	160780450	160792478	TCONS_00005258+TCONS_00005259	−
chr8	31257807	31258630	TCONS_00014668	+
chr1	95081953	95089740	TCONS_00001590+TCONS_00002406+TCONS_00000576	−
chr8	21222916	21267010	TCONS_00014948	−
chr20	48927248	48937879	TCONS_l2_00016931+TCONS_l2_00016666	−
chr8	76034610	76048950	TCONS_00014765	+
chr4	165798156	165818676	LOC100506013	+
chr10	45594924	45650044	RSU1P2	−
chr18	49574648	49691677	TCONS_00026330	+
chr8	7170368	7177473	DEFB109P1B	+
chr12	82950644	82954933	TCONS_00020858	−
chr7	26638503	26657903	TCONS_00013749+TCONS_00013750	−
chr7	76312462	76318469	TCONS_00013502	+
chr18	22250888	22251670	TCONS_00026461	−
chr4	116843171	117218316	TCONS_l2_00021950+TCONS_l2_00021951	−
chr2	4184234	4188321	TCONS_00003166	−
chr5	76393282	76393984	TCONS_00009708	−
chr3	62254	66367	TCONS_00005935+TCONS_00005485	+
chr5	139152113	139154991	TCONS_00009794	−
chr15	30704541	30712576	TCONS_l2_00008970	−
chr9	110477206	110477768	TCONS_00016459	−
chr15	85788461	85790478	TCONS_l2_00009466+TCONS_l2_00008801	+
chr5	10521850	10527001	TCONS_00009874	+
chr10	94819420	94820183	TCONS_00018025	−
chr2	88436735	88438049	TCONS_00005194	−
chr20	20702317	20703034	TCONS_00028013	−
chr1	231014538	231025361	TCONS_00002621	−
chr7	64541828	64549243	TCONS_l2_00025952+TCONS_l2_00025953+TCONS_ l2_00025954	+
chr2	123223599	123229276	TCONS_00003843+TCONS_00003844	+
chr14	25581978	25591222	TCONS_00022931	+
chr2	183982268	183983470	TCONS_00004961	+
chr15	36338208	36341709	TCONS_00023346	+
chr5	137919521	137928979	TCONS_00010103	+
chr13	28527615	28528466	TCONS_00021951	−
chrX	152435330	152439737	TCONS_00017103+TCONS_00017104	−
chr13	78036678	78039992	TCONS_00021838	+
chr4	189376732	189523062	LOC401164	+
chr12	98108898	98110881	TCONS_00020536+TCONS_00021193	+
chr17	31271487	31277548	TCONS_00025893+TCONS_00025894+TCONS_00025895 +TCONS_00025350	+
chr16	83964877	83967981	TCONS_00024895	+
chr4	39144101	39179316	TCONS_00008469	−

chr15	91382899	91396016	TCONS_00023505+TCONS_00023506	+
chr1	50772147	50773062	TCONS_00000934	+
chr7	127861457	127876469	TCONS_00013948	−
chr20	55841853	55858051	TCONS_00027871+TCONS_00027962	+
chr2	239463090	239469165	TCONS_00005040+TCONS_00005041	+
chr6	103982912	103988696	TCONS_00011907+TCONS_00012563	+
chr11	1792623	1793111	TCONS_00019559	−
chr7	56545203	56552105	TCONS_00013811+TCONS_00014305+TCONS_00014306 +TCONS_00012909	−
chr1	104615645	104619693	LOC100129138	+
chr22	21311691	21318877	TCONS_00029333	+
chr16	33726351	33736655	TCONS_l2_00010134	−
chr15	53092221	53098374	TCONS_00023680+TCONS_00024124+TCONS_00024125 +TCONS_00023681+TCONS_00023682	−
chr10	134827223	134828171	TCONS_00018395+TCONS_00018396	+
chr22	37361289	37364213	LOC100506241	+
chr1	148556023	148558168	TCONS_00002154+TCONS_00002155+TCONS_00001141	+
chr3	106469675	106489988	TCONS_00006579	−
chr9	138999331	138999842	TCONS_00016505	−
chr22	50984904	50986244	TCONS_00029630	+
chr12	9709910	9728864	TCONS_l2_00006576+TCONS_l2_00005493+TCONS_ l2_00005494+TCONS_l2_00005495+TCONS_l2_00005496	+
chr15	44715559	44718499	TCONS_00023389	+
chr3	179243602	179245572	TCONS_00006712	−
chrY	23557034	23563448	RBMY2EP	−
chr2	151468895	151492179	TCONS_00003031+TCONS_00004917+TCONS_00004 918+TCONS_00004919+TCONS_00003032+TCONS_0 0003033+TCONS_00003034+TCONS_00003035+TCO NS_00004921	+
chr4	34659228	34671054	TCONS_00007508+TCONS_00007509+TCONS_00007510	+
chrX	8831902	8835689	TCONS_00017037+TCONS_00017289	−
chr19	12670361	12671726	TCONS_l2_00012321	+
chr10	81136765	81137298	TCONS_00018545	−
chr10	110209781	110226098	TCONS_00018600	−
chr7	10676620	10677930	TCONS_00013695	−
chr4	139230865	139345498	LINC00499	+
chr1	200917966	200926877	TCONS_l2_00000747+TCONS_l2_00000748	+
chr3	130532579	130555401	TCONS_l2_00018861+TCONS_l2_00018862+TCONS_ l2_00018863	+
chr5	115074779	115092222	TCONS_00010062+TCONS_00010063	+
chr9	130349968	130354487	TCONS_00016160	+
chr3	177159709	177470492	LINC00578	+
chr7	63157086	63167629	TCONS_l2_00025918	+
chr13	95364970	95368197	TCONS_00021502	+
chr5	161336738	161428237	TCONS_00010539+TCONS_00010540	−
chr8	6844700	6866346	DEFT1P2	−
chr15	92346239	92347243	TCONS_00023783	−
chr7	51969078	52019930	TCONS_00013445	+
chr1	171584011	171595732	TCONS_00001200+TCONS_00000333	+

chr1	757786	759075	TCONS_00001367	−
chrX	39681271	39681758	TCONS_00017155	+
chr14	53635772	54153976	TCONS_l2_00008306+TCONS_l2_00007728+TCONS_l2_00007729+TCONS_l2_00007730+TCONS_l2_00007731+TCONS_l2_00008307+TCONS_l2_00008308+TCONS_l2_00008309	+
chr4	108500199	108507955	TCONS_00008189	+
chr9	75477340	75504807	TCONS_l2_00029403+TCONS_l2_00029404+TCONS_l2_00029991+TCONS_l2_00029990+TCONS_l2_00029989+TCONS_l2_00029405+TCONS_l2_00029406	−
chr3	140311062	140355193	TCONS_l2_00019558	−
chr14	36417310	36419101	TCONS_l2_00007673+TCONS_l2_00007674+TCONS_l2_00007675	+
chr17	20419278	20420554	TCONS_l2_00011136	−
chr11	63944169	63946364	TCONS_00020073	−
chr1	142840927	142861553	TCONS_l2_00000554	+
chr14	40938354	40939586	TCONS_00022486	+
chr7	41137093	41173105	TCONS_00013425+TCONS_00012998	+
chr19	54368017	54369394	TCONS_00026834	+
chr5	42922836	42924839	TCONS_00009933	+
chr10	30842783	30843412	TCONS_00017945	−
chr17	38092115	38096062	TCONS_00025381	+
chr6	150255663	150256756	TCONS_00011399+TCONS_00011984	+
chr2	21320551	21322019	TCONS_00004173	−
chr1	30464332	30465136	TCONS_00000878+TCONS_00001970	+
chr3	184433380	184477461	TCONS_00006332+TCONS_00007053+TCONS_00007052+TCONS_00006333+TCONS_00006334+TCONS_00006335+TCONS_00006336+TCONS_00006337+TCONS_00007054	+
chr10	122521324	122610691	WDR11-AS1	−
chr1	95783151	95787167	TCONS_00001047	+
chr16	32300868	33298702	LOC390705	−
chr8	126479045	126485552	TCONS_00014852	+
chr1	192904813	192924493	TCONS_00001762+TCONS_00000707+TCONS_00001763+TCONS_00001764+TCONS_00001765+TCONS_00002545	−
chr1	116463389	116466789	TCONS_00001635	−
chr16	86320037	86326995	LOC146513	+
chr12	68100640	68123221	TCONS_l2_00006305+TCONS_l2_00006658+TCONS_l2_00006306	−
chr5	124204487	124211743	TCONS_00010461	−
chr14	57418305	57421277	TCONS_00022758	−
chr9	82006182	82006559	TCONS_l2_00028855	+
chr1	48671637	48672515	TCONS_00000198	+
chr2	2874330	2875003	TCONS_00003154	−
chr7	45817781	45828649	TCONS_l2_00025847+TCONS_l2_00025848	+
chr2	19562729	19563854	TCONS_00004162	−
chr10	10976903	10994126	LINC00710	−
chr10	30280163	30290010	TCONS_00018899	−
chr4	29119795	29204392	TCONS_00008029+TCONS_00007500	+

chr9	87172160	87199274	TCONS_00016637+TCONS_00016033+TCONS_00016034	+
chr16	52615180	52624898	TCONS_00024415	+
chr1	234701044	234705834	TCONS_00000420	+
chr3	99226031	99245957	TCONS_00005801	−
chr17	48553180	48555752	TCONS_l2_00011669	−
chr8	71437329	71448723	TCONS_00015038	−
chr20	48782899	48788279	TCONS_00027945+TCONS_00027946	+
chr20	24123226	24125022	TCONS_00028026	−
chr10	63541514	63581160	TCONS_00017987+TCONS_00018526+TCONS_00018527	−
chr14	55553592	55561333	TCONS_00022751	−
chr1	229394028	229406788	TCONS_l2_00002790+TCONS_l2_00001839+TCONS_l2_00001840	−
chr9	121713385	121742701	TCONS_00016144+TCONS_00016145+TCONS_00016146	+
chr1	182031812	182038388	TCONS_l2_00002726	−
chr6	8984905	9046711	TCONS_00011689+TCONS_00011690	+
chr13	32035642	32057434	TCONS_00021748	+
chr15	36782442	36799342	TCONS_00023350	+
chr4	157545914	157563834	TCONS_00009151+TCONS_00009152+TCONS_00009153	−
chr13	42535305	42555701	VWA8-AS1	+
chr4	53656161	53681631	LOC152578	+
chr6	140297467	140414872	TCONS_00011196+TCONS_00012613+TCONS_00011387+TCONS_00011970+TCONS_00011971+TCONS_00011972+TCONS_00011388+TCONS_00011973+TCONS_00011389	+
chr1	171652534	171653191	TCONS_l2_00001619	−
chr18	716089	717930	TCONS_00026391	−
chr6	52066228	52068399	TCONS_00011831	+
chr12	29193463	29209554	TCONS_00020366+TCONS_00021091	+
chr10	125185229	125187156	TCONS_00018062	−
chr19	36087585	36092403	TCONS_l2_00012482+TCONS_l2_00012483+TCONS_l2_00012484+TCONS_l2_00013318	+
chr9	138023133	138027186	TCONS_00016212	+
chr6	159884340	159888920	TCONS_00012008+TCONS_00012009	+
chr6	28580905	28597774	TCONS_00011763	+
chr2	15703525	15713992	TCONS_00003584+TCONS_00002829	+
chr2	227150455	227198292	TCONS_00004599	−
chr22	33504515	33512280	TCONS_00029416	+
chr7	123560905	123564184	TCONS_00013573	+
chr8	57942969	57965702	TCONS_00014706	+
chr9	33504535	33511164	SUGT1P1	−
chr1	203395870	203398365	TCONS_00001775	−
chr11	331734	333645	TCONS_00019181	+
chr2	181556789	181589386	TCONS_00003955	+
chr1	207352622	207357525	TCONS_l2_00000782+TCONS_l2_00000783	+
chr10	30131628	30132208	TCONS_00018898	−
chr2	79102285	79107703	TCONS_00004316	−
chr18	73944963	73971999	TCONS_00026373+TCONS_00026374+TCONS_00026648	+

chr16	79709816	79841790	TCONS_00024477+TCONS_00024478+TCONS_00024892+TCONS_00024893+TCONS_00024479+TCONS_00024894+TCONS_00024480+TCONS_00024481+TCONS_00024482	+
chr8	26723017	26724760	TCONS_00014512	−
chr22	45529639	45559662	LOC100506714	−
chr5	141227143	141231803	TCONS_00009526	+
chr6	125633313	125634852	TCONS_00012271	−
chr2	201979497	201980387	TCONS_00005314	−
chr7	6895314	6899423	TCONS_l2_00026374+TCONS_l2_00026375	−
chr19	46560932	46580887	TCONS_00026818	−
chr19	32593958	32595603	TCONS_00026991+TCONS_00026992	+
chr5	67804065	67861330	TCONS_00010945+TCONS_00010362+TCONS_00010363+TCONS_00010946+TCONS_00010948+TCONS_00010947+TCONS_00010949+TCONS_00010950+TCONS_00009686	−
chr2	2886700	2898250	TCONS_00004099	−
chr2	103489798	103512180	TCONS_l2_00015871+TCONS_l2_00014742+TCONS_l2_00014743	−
chr1	173832386	173833079	GAS5-AS1	+
chr1	65532310	65533420	TCONS_00000543	−
chrX	134169666	134174761	TCONS_00017250	+
chr1	220533073	220533525	TCONS_00000735	−
chr7	98902269	98908748	TCONS_l2_00027184+TCONS_l2_00026117+TCONS_l2_00026118	+
chr7	155985721	155986607	TCONS_00014032	−
chr2	165824448	165841004	TCONS_00004485+TCONS_00005263	−
chr6	39916124	39920971	TCONS_00011810	+
chr5	118096169	118121154	TCONS_00009761	−
chr1	195450556	195469368	TCONS_00001232	+
chr9	67579807	67627731	TCONS_00015600+TCONS_00015824	−
chr8	9895361	9909473	TCONS_00014932	−
chr6	91054938	91072351	TCONS_00011177	+
chr20	18768615	18775228	LINC00652	−
chr17	29036626	29097068	SUZ12P1	+
chr15	82152107	82259442	TCONS_00023758	−
chr3	11763260	11766484	TCONS_00006441	−
chr6	224950	226400	TCONS_00011250	+
chrY	9448330	9452762	RBMY3AP	−
chr13	43787255	43787647	TCONS_00021771	+
chr2	1576326	1584083	TCONS_00002796	+
chr2	170961711	170963144	TCONS_00003931	+
chr18	42257061	42259599	TCONS_00026507	−
chr5	26483820	26484811	TCONS_00010284	−
chr2	156868129	157111432	TCONS_l2_00015999+TCONS_l2_00016000+TCONS_l2_00014958	−
chr4	111288133	111291542	TCONS_00008869	+
chr21	23774315	23847245	TCONS_l2_00017171+TCONS_l2_00017172	−
chr5	25100851	25192187	TCONS_l2_00022814+TCONS_l2_00022815	−

chr20	44543295	44544555	TCONS_00028410	−
chr12	114241429	114244372	TCONS_00020566	+
chr1	193653376	193657559	TCONS_00000708	−
chr7	93940476	94018102	TCONS_00013536	+
chrY	28767932	28768991	TCONS_l2_00030956	−
chr19	28129391	28137384	TCONS_00026812	−
chr8	135732688	135736134	TCONS_00014864	+
chr2	96331832	96334463	TCONS_00002712	+
chr13	106063173	106066577	TCONS_00021888	+
chr3	106824691	106825838	TCONS_l2_00019431	−
chr3	128774334	128777479	TCONS_00006219+TCONS_00006218	+
chr3	146109150	146139933	TCONS_l2_00020211+TCONS_l2_00019562+TCONS_ l2_00019563+TCONS_l2_00019564	−
chr1	170240546	170253349	LOC284688	−
chr2	740304	741130	TCONS_00005054	−
chr7	55661470	55662699	TCONS_00013016	+
chr1	56441023	56483498	TCONS_00000953	+
chr18	73764462	73768286	TCONS_00026570	−
chr1	1980669	1981566	TCONS_00001374+TCONS_00000453	−
chr9	47162727	47164029	TCONS_00015989	+
chr2	43028634	43038968	TCONS_00004223+TCONS_00004224	−
chr4	191028242	191030033	TCONS_00008726	−
chr17	13289860	13290390	TCONS_00025563	−
chr5	54359007	54362058	TCONS_00009402	+
chr2	14756552	14770316	TCONS_00002828+TCONS_00002827	+
chr10	34048641	34061608	LINC00838	−
chr11	55061179	55065708	TRIM51HP	−
chr17	36606633	36608764	TCONS_00025620+TCONS_00025622+TCONS_000 25623+TCONS_00025624+TCONS_00025625+TCO NS_00025626	−
chr16	55819981	55827371	TCONS_l2_00009765	+
chrX	2527306	2575270	CD99P1	+
chr3	152365935	152368236	TCONS_00005625	+
chr7	4473994	4518319	TCONS_00013326	+
chrX	27847891	27851835	TCONS_l2_00030168	+
chr17	58160927	58165828	LOC645638	−
chr7	389824	390706	TCONS_00014246	−
chr9	96717879	96721422	TCONS_00015719+TCONS_00015720	+
chr4	63796124	63814906	TCONS_l2_00020549	+
chr20	26230309	26232123	TCONS_00028362	−
chr2	155822025	155824515	TCONS_00003424	−
chr15	52407274	52409155	TCONS_00024123	−
chr19	32563115	32565007	TCONS_00026989+TCONS_00026990	+
chr3	59035911	59105235	TCONS_00006038+TCONS_00005526	+
chr2	13424960	13430867	TCONS_00003582	+
chr11	91527512	91545447	TCONS_00019434+TCONS_00019435+TCONS_00019968 +TCONS_00019969+TCONS_00019436+TCONS_0001943 7+TCONS_00019970+TCONS_00019438	+

chr6	44041650	44045689	TCONS_00011305	+
chr14	25564556	25571468	TCONS_00022436	+
chr2	151008158	151022842	TCONS_00004473	−
chr10	105697904	105726769	TCONS_00018594+TCONS_00018595	−
chr7	48883532	48885282	TCONS_00014132	+
chr7	93891298	94021491	TCONS_00013886+TCONS_00013887+TCONS_00014353 +TCONS_00013888	−
chr9	70093000	70165106	TCONS_l2_00028817	+
chr21	10450461	10599892	TCONS_l2_00017127+TCONS_l2_00017415+TCONS_ l2_00017128+TCONS_l2_00017129+TCONS_00029076	−
chr21	17259153	17267300	TCONS_00028969	+
chr1	223266594	223269136	TCONS_00001833+TCONS_00002599	−
chr2	42329148	42329924	TCONS_00004216	−
chr4	184393764	184400227	TCONS_00008948	+
chr5	141543140	141548174	TCONS_00010118	+
chr6	117993976	117996447	TCONS_00011550	−
chr3	185543989	185545049	TCONS_00006340	+
chr14	27067611	27249810	TCONS_00022933+TCONS_00022440+TCONS_00022936 +TCONS_00022935+TCONS_00022934+TCONS_0002244 1+TCONS_00022937+TCONS_00022938+TCONS_000224 42+TCONS_00022443	+
chr12	8383645	8395542	FAM86FP	−
chr10	63238855	63255993	TCONS_00018214+TCONS_00018215+TCONS_00018740	+
chr4	1573436	1582249	TCONS_00009007+TCONS_00008408+TCONS_00007708	−
chr4	3675320	3679582	LOC100133461	−
chr6	14391462	14393519	TCONS_00011457	−
chr22	23893497	23894725	TCONS_00029531	+
chr20	30940478	30945985	TCONS_00027922	+
chr2	105320216	105321967	TCONS_00002963	+
chr15	94074271	94086452	TCONS_00023794	−
chr18	19928868	19929243	TCONS_00026454	−
chr6	170563422	170571657	LOC154449	−
chr5	33229829	33255659	TCONS_l2_00022250+TCONS_l2_00023369	+
chr5	91990555	92014848	TCONS_00009462+TCONS_00009463	+
chr10	23864529	23875172	TCONS_00018138+TCONS_00018685+TCONS_00018139	+
chr13	114580581	114582349	TCONS_00021605	+
chr2	138859454	138863475	TCONS_00003009	+
chr1	213990570	214089305	TCONS_00001815+TCONS_00002584+TCONS_00002585	−
chr2	230989750	231032210	TCONS_00004051+TCONS_00003119	+
chr4	40267092	40268077	TCONS_00007518	+
chr10	5636954	5638081	TCONS_00017912	−
chr12	52486858	52488557	TCONS_00021312	−
chr5	40389129	40389924	TCONS_00009928	+
chr7	36878577	36880978	TCONS_00012997	+
chr9	133231492	133253556	TCONS_l2_00029088+TCONS_l2_00029089+TCONS_ l2_00029090	+
chr6	42420213	42423732	TCONS_00011815	+
chr7	66795257	66829655	TCONS_00013855+TCONS_00013856+TCONS_00013188	−
chr18	11366766	11378345	TCONS_00026421+TCONS_00026674+TCONS_00026422	−

chr3	177652611	177655450	TCONS_00005666	+
chr3	86313705	86316546	TCONS_00005798+TCONS_00007195	−
chr20	54676818	54680622	TCONS_00028572+TCONS_00028231	+
chr11	2350162	2350837	TCONS_00019564	−
chr3	44040886	44059829	TCONS_00006002	+
chr3	97949910	97956219	TCONS_00006562+TCONS_00007208+TCONS_00007209	−
chr10	42737724	42772399	TCONS_l2_00002997+TCONS_l2_00002998+TCONS_ l2_00002999	+
chr1	219781270	219786516	TCONS_l2_00001786+TCONS_l2_00001787	−
chr12	18906533	18926324	TCONS_00020348	+
chr7	56726844	56742773	TCONS_00013455+TCONS_00013456+TCONS_00013457 +TCONS_00013458+TCONS_00013459+TCONS_0001346 0+TCONS_00013461+TCONS_00013462	+
chr21	9517795	9540519	TCONS_00029067	−
chr4	89282461	89283590	TCONS_00008156+TCONS_00008836	+
chr5	149810792	149821794	TCONS_00010140	+
chr12	111374406	111375250	LOC100131138	+
chrY	28483532	28500466	TCONS_l2_00030954	−
chr2	88272040	88273168	TCONS_l2_00013752+TCONS_l2_00013753	+
chr22	17061967	17063492	TCONS_l2_00018300	−
chr2	190202503	190305828	TCONS_00004532+TCONS_00005292	−
chr17	70068474	70089622	TCONS_00025197+TCONS_00025480	+
chrX	2737206	2742169	TCONS_00017283	−
chrX	151889195	151890140	TCONS_00017102	−
chr5	114999015	115009135	TCONS_00010443	−
chr8	37439300	37440484	TCONS_00014675	+
chr2	112141166	112142811	TCONS_l2_00013897	+
chr7	65466089	65496528	TCONS_l2_00026644+TCONS_l2_00026645	−
chr17	21123514	21133235	TCONS_00025331	+
chr11	29616467	29652239	TCONS_00019875+TCONS_00019876+TCONS_00019268	+
chr9	90477703	90481205	TCONS_l2_00028882+TCONS_l2_00029789	+
chrX	53388441	53393518	TCONS_00017515	−
chr7	155029884	155045775	TCONS_l2_00026989+TCONS_l2_00026991	−
chr12	92951148	92977935	TCONS_00020518	+
chr22	17227760	17229328	TCONS_00029344	−
chr5	73617456	73636616	TCONS_00009426+TCONS_00009998+TCONS_000 10713+TCONS_00010714+TCONS_00009251+TCO NS_00010715	+
chr2	78639673	78769187	TCONS_l2_00015845	−
chr7	50292780	50296511	TCONS_00014296+TCONS_00013799	−
chr4	99627529	99628312	TCONS_00008532	−
chr10	16569514	16581773	TCONS_00018127	+
chrX	128809964	128812358	TCONS_00017369	−
chr22	20958200	20960593	TCONS_l2_00017899	−
chr4	169931532	169932431	TCONS_00008315+TCONS_00008316	+
chr22	21055402	21058891	TMEM191A	+
chr8	23082734	23088439	LOC389641	+
chr7	157074230	157075380	TCONS_00013284	−

chr6	122426345	122432157	TCONS_00012264	−
chr1	200380829	200447421	TCONS_00001233+TCONS_00000061+TCONS_00000367	+
chr13	81231053	81270425	TCONS_l2_00006951	+
chr6	159528338	159537085	TCONS_00012005+TCONS_00011402	+
chr1	78695283	78835147	MGC27382	+
chr8	133697316	133699876	TCONS_00015177	−
chr7	128273069	128281168	TCONS_00014209	+
chr2	100107781	100125484	TCONS_00003791	+
chr21	16290855	16319417	TCONS_00028793	+
chr17	66638398	66638883	TCONS_00025472	+
chr7	155061986	155069592	TCONS_00014020+TCONS_00014447	−
chr4	190628730	190642667	TCONS_l2_00021489	−
chr1	84542062	84543595	TCONS_00001546	−
chr3	105592596	105599421	TCONS_00006570	−
chr21	30291160	30295198	TCONS_00029108	−
chr7	15728700	15730479	TCONS_00013124	−
chr3	58419601	58470480	TCONS_00006864+TCONS_00006034+TCONS_00006035 +TCONS_00006865+TCONS_00006036+TCONS_0000686 6+TCONS_00006867	+
chr18	11159318	11159699	TCONS_00026420	−
chr3	159818478	159820661	TCONS_l2_00019954	+
chr1	222262511	222560776	TCONS_l2_00002779	−
chr2	739588	740177	TCONS_00005052+TCONS_00005053	−
chr2	206702660	206712309	TCONS_00003991	+
chr9	76026382	76028250	TCONS_00016357	−
chr10	91923670	91965545	TCONS_00018281	+
chrX	49004870	49012503	TCONS_00017320	−
chr11	68763662	68764006	TCONS_00019361	+
chr1	38940868	38942156	TCONS_00000188	+
chr21	42931052	42934885	TCONS_00028939	−
chr21	47513178	47517444	TCONS_00028961	−
chr11	11656826	11672435	TCONS_00019576	−
chr4	135345022	135379007	TCONS_00007627	+
chr12	102089780	102091162	TCONS_00021393	−
chr19	35414103	35417732	TCONS_00026830	+
chr1	151120885	151123810	TCONS_00001679	−
chr4	158493642	158497303	LOC340017	+
chr21	20418629	20420003	TCONS_00028801	+
chr13	99229498	99231177	TCONS_00021587+TCONS_00021878+TCONS_00021879	+
chr11	59710363	59737619	TCONS_00019094	+
chr13	50638816	50640735	TCONS_00021550	+
chr12	9600969	9614245	TCONS_00020322	+
chr13	57711063	57711858	TCONS_00022031	−
chrX	22446249	22512830	TCONS_l2_00030668	+
chr21	20673930	20676056	TCONS_00028803	+
chr12	73318544	73367380	TCONS_00020470	+
chr5	45890318	45896072	TCONS_00009396	+
chr20	47656924	47662581	TCONS_00028695+TCONS_00028061	−

chr20	26113523	26114834	TCONS_00028034	−
chr3	112315410	112322287	TCONS_00006930	+
chrX	150561134	150564686	TCONS_00017386	−
chr13	54334856	54349119	TCONS_00021795	+
chr20	46421066	46428516	TCONS_00028182	+
chr6	42909008	42915981	TCONS_00012717	−
chr7	42366038	42368604	TCONS_00013784	−
chr12	128571077	128602890	TCONS_00020992+TCONS_00021450+TCONS_00021451+TCONS_00020993+TCONS_00021452+TCONS_00021453	−
chr3	12818116	12833839	TCONS_l2_00019174	−
chr3	13691792	13695339	TCONS_00006446+TCONS_00007118+TCONS_00007119	−
chr11	29103586	29180505	TCONS_00019597	−
chr9	11987	14525	DDX11L5	+
chr3	59521935	59522709	TCONS_l2_00018583	+
chr12	97488889	97521452	TCONS_00020535	+
chr1	120388521	120389383	TCONS_00001130	+
chr5	44698533	44700910	TCONS_00009648	−
chr4	123545866	123547447	TCONS_00008215	+
chr2	238854397	238870550	TCONS_00004063	+
chr20	36199533	36205636	TCONS_00028157	+
chr2	73383766	73385965	TCONS_00004307	−
chr16	87152785	87153455	TCONS_00024500	+
chr1	156471576	156478185	TCONS_00001690	−
chr4	185192863	185198481	TCONS_00008687+TCONS_00009186	−
chr2	105363095	105374177	LOC284998	−
chr1	157256918	157257785	TCONS_00001693	−
chr3	101431278	101432260	PDCL3P4	+
chr4	139741111	139933800	TCONS_00007856+TCONS_00007857+TCONS_00008590	−
chr6	3893360	3894524	TCONS_00011255	+
chr5	70741443	70742396	TCONS_00009415	+
chr5	97912188	97914015	TCONS_l2_00022445	+
chr10	42383279	42396474	TCONS_00017955+TCONS_00017956	−
chr22	27539439	27555840	TCONS_00029399	+
chr12	112331318	112333002	TCONS_00021409	−
chrX	43278113	43297884	TCONS_00017157	+
chr12	3865741	3867330	TCONS_00020677	−
chr3	187715210	187721778	TCONS_00005912+TCONS_00005913	−
chrX	3820107	3855883	TCONS_l2_00030431+TCONS_l2_00030777+TCONS_l2_00030432+TCONS_l2_00030433+TCONS_l2_00030434	−
chr2	16190549	16225923	TCONS_00002831+TCONS_00002832	+
chr20	10836923	10847830	TCONS_00028105+TCONS_00028106+TCONS_00027903	+
chr16	74876359	74877177	TCONS_l2_00009838	+
chr13	25588663	25596910	TCONS_l2_00006759	+
chr7	27495996	27498498	TCONS_l2_00026452	−
chr5	12574969	12805295	CT49	+
chr1	230959387	230960453	TCONS_00000755	−
chr22	45831744	45844624	TCONS_00029489	−

chr9	110102407	110116943	TCONS_00016118+TCONS_00016119	+
chr9	81750350	81753633	TCONS_00016366+TCONS_00016816+TCONS_00016367	−
chr11	95250172	95251265	TCONS_00019735	−
chr2	205340079	205372395	TCONS_00003082	+
chr21	46767517	46773433	TCONS_00029062	+
chr6	142454227	142456288	TCONS_l2_00025263	+
chr1	184298205	184301960	TCONS_00000348	+
chr13	40177556	40215175	TCONS_00021760	+
chr2	200472791	200529926	TCONS_l2_00015569+TCONS_l2_00014184+TCONS_ l2_00015570+TCONS_l2_00014185+TCONS_ l2_00015571+TCONS_l2_00014186	+
chrX	135991638	135992594	TCONS_00017379	−
chr2	155792213	155801648	TCONS_l2_00014955	−
chr1	120106503	120115199	HSD3BP4	+
chr13	44495592	44561640	TCONS_l2_00006815+TCONS_l2_00006816	+
chr10	38156418	38175426	TCONS_l2_00002983+TCONS_l2_00002984+TCONS_ l2_00002985	+
chr11	119817137	119821850	TCONS_00019502	+
chr2	243001271	243002921	TCONS_00005049	+
chr20	1385807	1404641	TCONS_l2_00016420+TCONS_l2_00016421+TCONS_ l2_00016422+TCONS_l2_00016423	−
chr7	29724770	29727859	DPY19L2P3	+
chr9	3526723	3694788	TCONS_l2_00029674+TCONS_l2_00028602+TCONS_ l2_00028603+TCONS_l2_00028604+TCONS_ l2_00029675+TCONS_l2_00028605+TCONS_ l2_00028606+TCONS_l2_00029676+TCONS_ l2_00028607+TCONS_l2_00028608	+
chr10	60226958	60227567	TCONS_00018207	+
chr21	26825507	26839042	TCONS_00028901+TCONS_00028902	−
chr2	221655798	221662613	TCONS_00003112	+
chr2	46520809	46523596	TCONS_00004241	−
chr4	137039830	137316626	TCONS_l2_00021990+TCONS_l2_00021991	−
chr10	130711143	130714535	TCONS_00017889+TCONS_00018384	+
chr6	160060339	160061133	TCONS_00011605	−
chr1	212826523	212838945	TCONS_l2_00001761+TCONS_l2_00002765+TCONS_ l2_00001762	−
chr3	72042684	72046036	TCONS_00006093	+
chr9	122257653	122286403	TCONS_00015744	+
chr5	37071723	37072811	TCONS_00010304	−
chr5	2737003	2737395	TCONS_00010248	−
chr17	32806331	32806976	TCONS_00025167+TCONS_00025168	+
chr3	125933339	125934349	TCONS_00006199	+
chr19	40448555	40449653	TCONS_l2_00013109+TCONS_l2_00013110	−
chr7	136848880	136850347	TCONS_l2_00026236	+
chr1	168286744	168288713	TCONS_00001189	+
chr1	156584263	156587138	TCONS_00001691	−
chr14	48702994	48704782	TCONS_00022738	−
chr7	55808105	55816936	TCONS_l2_00025878	+
chr6	62340207	62371274	TCONS_00012198	−

chr5	177236176	177236486	TCONS_l2_00022656	+
chr13	91145542	91187593	TCONS_00021501+TCONS_00021870	+
chr15	69921213	69964240	TCONS_00023451	+
chr7	134368896	134372742	TCONS_00013083	+
chr16	16450071	16464251	TCONS_l2_00009611+TCONS_l2_00009612+TCONS_l2_00009613	+
chr20	46020673	46041071	TCONS_l2_00016926+TCONS_l2_00016639	−
chr22	18721397	18736925	TCONS_l2_00018144+TCONS_l2_00017580	+
chrX	125243745	125249545	TCONS_00017010	+
chr4	176379440	176387848	TCONS_00007907	−
chr20	5479218	5485242	LINC00654	−
chr5	16373470	16440190	TCONS_00009610+TCONS_00010277+TCONS_00010278+TCONS_00010904	−
chr13	78587018	78627730	LINC00446	+
chr1	107360861	107408733	TCONS_l2_00002120+TCONS_l2_00000468+TCONS_l2_00000469+TCONS_l2_00000470	+
chr2	144557036	144577168	TCONS_00003014	+
chr20	46988654	46999381	LINC00494	+
chr2	213775727	213784533	TCONS_00003095	+
chr10	49265281	49275670	TCONS_l2_00003558+TCONS_l2_00004183+TCONS_l2_00003559+TCONS_l2_00003560	−
chr6	107165327	107235300	LOC100422737	−
chr9	44104420	44110582	TCONS_l2_00029294	−
chr9	116613053	116613376	TCONS_l2_00029566	−
chr2	98086678	98100408	TCONS_00002947	+
chr20	6711403	6716783	TCONS_l2_00016472+TCONS_l2_00016841	−
chr20	61405473	61408208	LINC00659	−
chrY	14774298	14804153	TTTY15	+
chr14	84369854	84381682	TCONS_00023141+TCONS_00022818+TCONS_00022819	−
chr5	119581143	119669197	TCONS_00009764	−
chr2	118515139	118561750	TCONS_00003385	−
chr6	110870790	110874405	TCONS_00011916	+
chr6	137992331	137994453	TCONS_00012290	−
chr9	38127478	38134699	TCONS_00015975	+
chr9	116381508	116384914	TCONS_00015737	+
chr2	226750369	226756263	TCONS_00004598	−
chr9	11277589	11277964	TCONS_00015918	+
chrY	21440566	21442464	TCONS_00017639	+
chr11	123946697	123947693	TCONS_00019513	+
chr16	34338531	34339155	TCONS_l2_00009704	+
chr12	52604714	52617597	LINC00592	+
chr9	130919332	130921896	TCONS_00016167+TCONS_00016706	+
chr3	97796999	97801489	TCONS_00006130	+
chr1	25367664	25373839	TCONS_00001961	+
chr13	95954705	95956401	TCONS_00021689	−
chr18	63887404	63922089	TCONS_00026548	−
chr10	34198166	34206732	TCONS_00017811	+
chr3	149735043	149740537	TCONS_00006680	−

chr5	141536509	141548749	TCONS_00010518	−
chr5	149016983	149020628	TCONS_00010530	−
chr2	55852073	55858573	TCONS_00003286	−
chr21	14918354	14930782	TCONS_l2_00017319+TCONS_l2_00016967	+
chr7	69057888	69063004	TCONS_00013859+TCONS_00013860+TCONS_00013861+TCONS_00013862+TCONS_00013863+TCONS_00013193	−
chrX	136638350	136646546	TCONS_00017094+TCONS_00017095	−
chr6	122004299	122004989	TCONS_l2_00024401	+
chr20	5412603	5426394	LINC00658	−
chr14	21668434	21672514	TCONS_00022389	+
chr14	51411365	51413305	TCONS_00022499	+
chr22	23909251	23915309	TCONS_00029370	+
chr4	124246744	124247269	TCONS_00008216	+
chr3	161534697	161539696	TCONS_00005865	−
chr19	51773414	51774824	TCONS_00027396	−
chrX	56316470	56325981	TCONS_00017181	+
chr14	103653558	103655365	LINC00605	−
chr17	8608555	8609483	TCONS_00025556	−
chr12	77407017	77407562	TCONS_00020480	+
chr1	182096432	182097800	TCONS_00000695	−
chr11	17716818	17719033	TCONS_00019586+TCONS_00020045+TCONS_00019587	−
chr3	90263034	90264881	TCONS_l2_00019377	−
chr2	43199538	43233380	TCONS_00004225+TCONS_00003262	−
chr1	223316723	223321526	TCONS_00001294+TCONS_00000408	+
chr9	109864325	109865153	TCONS_00015865	−
chrX	39796557	39797694	TCONS_00017156	+
chr7	50241835	50243914	TCONS_00013168	−
chr13	30893204	30919526	TCONS_00021744	+
chr2	130345374	130351825	TCONS_00003870	+
chr1	205744730	205752610	TCONS_00001250	+
chr6	144606291	144609320	TCONS_00012614	+
chr3	6532166	6778421	TCONS_l2_00019755+TCONS_00005490+TCONS_00005491+TCONS_l2_00019758+TCONS_l2_00019759+TCONS_l2_00018432	+
chr12	58982070	58983668	TCONS_00020447	+
chr21	46790863	46791842	TCONS_00029063	+
chr15	26110448	26122552	TCONS_00023322+TCONS_00023886+TCONS_00023323	+
chr1	156416204	156426369	TCONS_l2_00002668+TCONS_l2_00001557+TCONS_l2_00002669+TCONS_l2_00001558	−
chr8	40156244	40210633	TCONS_00014983+TCONS_00015431+TCONS_00014984+TCONS_00014985	−
chr13	53705021	53711469	TCONS_00022029	−
chr1	226409812	226410285	TCONS_00001313	+
chr3	110246329	110248429	TCONS_00005810	−
chr2	231450743	231451708	TCONS_00004610	−
chr22	18879968	18882205	TCONS_00029644	−
chr10	4395530	4397192	TCONS_00018094	+
chr7	16625596	16626306	TCONS_l2_00026389	−

chr3	151354248	151375840	TCONS_l2_00018933+TCONS_l2_00019933+TCONS_l2_00018934	+
chr5	1159526	1161418	TCONS_00010241	−
chr15	79512304	79513126	TCONS_00023477	+
chr4	26861428	26862250	TCONS_00007732+TCONS_00007731+TCONS_00007733+TCONS_00008453	−
chr15	99635728	99641040	TCONS_00023831+TCONS_00024220	−
chr11	45772392	45773152	TCONS_00019633	−
chr11	119808789	119810635	TCONS_00019501	+
chr17	4703748	4710294	TCONS_l2_00011049+TCONS_l2_00011050	−
chr3	128191835	128193950	TCONS_00006208	+
chr17	41393291	41393906	TCONS_00025394	+
chrY	10035280	10036679	TCONS_l2_00030893	+
chr3	177483365	177484684	TCONS_00007046	+
chr3	120530352	120555220	TCONS_00006620+TCONS_00005465	−
chr10	31119045	31124561	TCONS_00018468+TCONS_00018469	−
chr14	74289262	74296838	TCONS_00022375	−
chr5	60920229	60923894	TCONS_00009279+TCONS_00010353	−
chr14	39377287	39434491	TCONS_00022481+TCONS_00022971	+
chr7	98297763	98300444	TCONS_00013544	+
chr10	81388513	81391944	TCONS_00018002+TCONS_00018003	−
chr17	26256574	26301249	TCONS_l2_00011637+TCONS_l2_00011165	−
chr11	61238445	61239949	TCONS_00020070	−
chr3	83825693	83828115	TCONS_00006549	−
chr5	68430029	68455323	TCONS_00010366	−
chrY	9528709	9531308	TTTY8B	+
chr10	54210638	54230293	TCONS_00017736+TCONS_00017981	−
chr4	157563418	157565001	TCONS_00008305+TCONS_00008306	+
chr18	4470282	4485710	TCONS_00026244	+
chr14	105111662	105122019	TCONS_00022895	−
chrX	4689124	4692236	TCONS_00017284	−
chr2	7213467	7215379	TCONS_00004684	+
chr12	127615944	127630565	TCONS_00020641	+
chr10	4457497	4468371	TCONS_00017908	−
chr7	29676027	29677540	TCONS_00012987	+
chr7	72333318	72339655	SPDYE7P	−
chr1	159746250	159749163	TCONS_00002502+TCONS_00001701+TCONS_00001700+TCONS_00001702+TCONS_00001703	−
chr2	133092597	133096192	TCONS_00003878+TCONS_00003879+TCONS_00004898+TCONS_00004899+TCONS_00003880	+
chr2	242961960	242968327	TCONS_l2_00015204+TCONS_l2_00016125	−
chr20	30863239	30865358	TCONS_00028366	−
chr4	2011391	2043486	TCONS_00008409+TCONS_00009008+TCONS_00008410	−
chr14	101798302	101801206	TCONS_00022878+TCONS_00022879	−
chr20	25715264	25734528	TCONS_l2_00016214+TCONS_l2_00016215+TCONS_l2_00016216+TCONS_l2_00016217+TCONS_l2_00016218	+
chr2	124774435	124782750	TCONS_00003397	−
chr20	46653532	46702929	TCONS_00028194+TCONS_00028195+TCONS_00028559+TCONS_00028560+TCONS_00028196	+

chr11	108370157	108372543	TCONS_00019459	+
chr7	70972	71835	TCONS_00013110	−
chr16	26596055	26606756	TCONS_l2_00010069+TCONS_l2_00010450	−
chr9	107032509	107049236	TCONS_l2_00029822+TCONS_l2_00029823	+
chr13	89925457	89932494	TCONS_00021585	+
chr5	4974803	5034380	TCONS_00009590+TCONS_00010253	−
chr22	23732792	23744799	ZDHHC8P1	−
chr19	76220	77690	FAM138A	−
chrY	24585087	24631739	TCONS_00017615+TCONS_00017579+TCONS_00017616	+
chr4	164028214	164041637	TCONS_00008639	−
chr10	92805565	92821916	LINC00502	+
chr1	158096904	158098261	TCONS_00002497+TCONS_00001694	−
chr3	171612226	171613030	TCONS_00005659	+
chr18	29281969	29306687	TCONS_l2_00012045+TCONS_l2_00012046	−
chr7	25594360	25607592	TCONS_00013731	−
chr2	82049489	82077656	TCONS_00003749	+
chr15	94026418	94041974	TCONS_00023519	+
chr8	128902874	129113499	PVT1	+
chr16	3039055	3044510	LINC00514	+
chr2	105421883	105467934	LOC100506421	−
chr1	227581292	227618721	TCONS_l2_00002326+TCONS_l2_00002327	+
chr20	51237377	51266970	TCONS_00027950	+
chr4	97231852	97285603	TCONS_00007398	+
chr11	141453	180409	TCONS_l2_00005353+TCONS_l2_00004867+TCONS_l2_00004868+TCONS_l2_00004869	−
chr6	143268774	143279584	TCONS_l2_00024456+TCONS_l2_00024457+TCONS_l2_00025264+TCONS_l2_00024458	+
chr17	4560923	4567717	TCONS_00025533	−
chr1	218338445	218340185	TCONS_00001275	+
chr2	3605976	3609340	LOC100506054	+
chr17	71908271	71911948	TCONS_00025482	+
chr1	142826654	142829106	TCONS_l2_00000553	+
chr6	106806441	106807592	TCONS_00011531	−
chr10	134757471	134778928	TCONS_00018074+TCONS_00018073+TCONS_00018075	−
chr9	89999784	90001522	TCONS_00016390+TCONS_00016391	−
chr9	46787357	46843989	TCONS_l2_00029305+TCONS_l2_00029306	−
chr15	74210409	74211722	TCONS_00023266	+
chr14	38218187	38219095	TCONS_l2_00007692	+
chr8	104456732	104457927	TCONS_00015126+TCONS_00015127	−
chr15	47083523	47099833	TCONS_l2_00008623	+
chr2	74977311	75005821	TCONS_l2_00014655	−
chr3	27872379	27875627	TCONS_00005456	−
chr19	32433233	32436345	TCONS_00027295	−
chr5	149719783	149720699	TCONS_00010531	−
chr3	182698647	182703735	TCONS_00007319	−
chr7	156737415	156738339	TCONS_00013102	+
chr9	75705956	75726824	TCONS_00016354	−

chr9	140065310	140067457	TCONS_l2_00030092+TCONS_l2_00029661+TCONS_l2_00029662+TCONS_l2_00030093+TCONS_l2_00030094	−
chr20	46431523	46440897	TCONS_00028183	+
chr4	152812455	152849800	TCONS_00008623+TCONS_00007880+TCONS_00009147+TCONS_00008624	−
chr13	62897790	62902966	TCONS_l2_00007418	+
chr2	57947954	57953284	TCONS_00003694+TCONS_00003695	+
chr8	47609252	47610282	TCONS_l2_00027739	+
chr16	32005808	32013474	TCONS_l2_00009677	+
chr21	35552978	35562220	LINC00310	+
chr1	41329586	41342342	TCONS_l2_00002032+TCONS_l2_00000244	+
chr11	98360474	98365880	TCONS_00019739	−
chr6	2791142	2822143	TCONS_00012047	−
chr1	222054323	222151865	TCONS_00000738+TCONS_00000739	−
chr16	71360074	71362646	TCONS_00024449+TCONS_00024450	+
chr16	84818677	84829057	TCONS_00024486	+
chr2	201560446	201658941	AOX2P	+
chr11	65266414	65278498	TCONS_00019666+TCONS_00019667+TCONS_00019669+TCONS_00020083	−
chr5	65803372	65807715	TCONS_00009678+TCONS_00010358+TCONS_00010943	−
chr1	157019846	157021546	TCONS_00001692	−
chr5	149083585	149101072	TCONS_00010139	+
chr5	50746992	50757923	TCONS_00009399	+
chr4	190598435	190600102	TCONS_00008717	−
chrX	73327062	73327716	TCONS_l2_00030260	+
chr20	36305312	36311636	LOC100287792	+
chr18	61672062	61688260	TCONS_00026351	+
chr22	37860583	37862888	TCONS_00029696+TCONS_00029904	−
chrX	23791109	23801073	TCONS_00017047	−
chr14	46175495	46181226	TCONS_00022737	−
chr12	10433068	10443823	TCONS_00021289	−
chr21	25801019	25920256	TCONS_l2_00016988+TCONS_l2_00017342+TCONS_l2_00017341+TCONS_l2_00017343	+
chr1	222139683	222158306	TCONS_00000394+TCONS_00000395	+
chr8	29368979	29371353	TCONS_00014958	−
chr20	37034660	37045444	TCONS_00027888	−
chr18	36472506	36477474	TCONS_00026497	−
chr10	81213170	81214510	TCONS_00018249	+
chr8	34642570	34650462	TCONS_00014671	+
chr18	11185330	11186676	TCONS_00026277	+
chrY	1836240	1837669	TCONS_00017603	+
chr10	30127645	30128665	TCONS_00018702	+
chr5	67725581	67730308	TCONS_l2_00022934+TCONS_l2_00023764	−
chr8	142350648	142354720	LOC731779	+
chr8	86413304	86436470	TCONS_00014789	+
chr2	173188078	173190935	TCONS_00005278	−
chr13	41870339	41873857	TCONS_00021765	+
chr2	78176532	78178621	TCONS_00004315	−

chr7	57269356	57271289	TCONS_l2_00026594	−
chr9	71911597	71921972	BANCR	−
chr10	124102981	124104332	TCONS_00018621	−
chr5	134571924	134583867	LOC340073	−
chr4	160587655	160698936	TCONS_00007890	−
chr7	33769904	33771676	TCONS_00013151	−
chr16	33350480	33357427	TCONS_00024633	−
chr2	166931799	166938251	TCONS_00003929	+
chr10	26932037	26942383	LINC00202-2	+
chr10	126617452	126619962	TCONS_00018063	−
chr15	30938318	31065209	LOC100288637	+
chr2	140227345	140235317	TCONS_00003013	+
chr13	60788486	60789080	TCONS_00021661	−
chr11	10905706	10920824	TCONS_00019118+TCONS_00020040	−
chr2	5741872	5831918	TCONS_00004106+TCONS_00003170+TCONS_00004107+TCONS_00003171+TCONS_00004108+TCONS_00005064+TCONS_00005065+TCONS_00005066+TCONS_00005067+TCONS_00004109+TCONS_00005068+TCONS_00004110+TCONS_00003172	−
chr17	27337875	27339680	TCONS_00025602	−
chr2	95924701	95925721	TCONS_00003770	+
chr2	218147407	218200843	TCONS_00004026+TCONS_00003110	+
chr5	168017702	168044050	TCONS_l2_00022611	+
chr9	45436911	45441766	TCONS_l2_00028750	+
chr12	42157956	42159375	TCONS_00020381+TCONS_00021102+TCONS_00021104+TCONS_00021107+TCONS_00021106+TCONS_00020382+TCONS_00020383	+
chr16	67525954	67540403	TCONS_00024870+TCONS_00024442	+
chr5	154496132	154499168	TCONS_00010145	+
chr1	103817769	103828355	TCONS_00000264	+
chr1	3539429	3541333	TCONS_00002281+TCONS_00000461	−
chr10	91613273	91674712	TCONS_00018277+TCONS_00018278+TCONS_00018279+TCONS_00018280+TCONS_00018782	+
chr7	95101147	95103310	TCONS_00013046	+
chr14	104314058	104324386	LINC00637	+
chr16	75602	87928	TCONS_l2_00009916+TCONS_l2_00009917+TCONS_l2_00009918	−
chr3	197340898	197354752	LOC220729	−
chr1	87781430	87786243	TCONS_00001557	−
chr15	101359043	101365077	TCONS_00023836	−
chr20	54206130	54242676	TCONS_00028445+TCONS_00028446	−
chr2	221614393	221631427	TCONS_00004592+TCONS_00004593	−
chr11	115937628	115951753	TCONS_00019771+TCONS_00019770+TCONS_00020135+TCONS_00019772	−
chr6	167803650	167804979	TCONS_00012017	+
chr21	43099462	43117496	LINC00111	+
chr19	43057348	43057999	TCONS_l2_00013121	−
chr3	186210912	186212820	TCONS_00006344	+

chr15	33595778	33602861	TCONS_l2_00009004+TCONS_l2_00009005+TCONS_l2_00009006+TCONS_l2_00009007	−
chr8	12722983	12729454	TCONS_00014625	+
chr1	23908137	23919157	TCONS_00000866	+
chr7	124027821	124029556	TCONS_00013574	+
chr7	67485240	67497677	TCONS_00013018	+
chr17	46777978	46781655	TCONS_00025417	+
chr2	229294878	229379412	TCONS_00004049	+
chr17	20841851	20885670	TCONS_l2_00011537+TCONS_l2_00011539	+
chr4	188225237	188426767	LOC339975	−
chr4	138404923	138405512	TCONS_00008259	+
chr3	132733848	132755896	TCONS_00005829	−
chr2	238031553	238032740	TCONS_00003517	−
chr4	55242169	55242919	TCONS_00007531	+
chr4	23724885	23735559	TCONS_00007728+TCONS_00008448	−
chr15	41849136	41849865	TCONS_l2_00008597	+
chr22	39462294	39465987	TCONS_00029808+TCONS_00029809	+
chr22	42833352	42833949	TCONS_00029706	−
chrX	287992	288942	TCONS_00017110	+
chr17	32488643	32491175	TCONS_00025352	+
chr4	71764944	71767830	TCONS_l2_00021172	−
chr10	57911542	57914731	TCONS_00018521	−
chr7	110072296	110174811	TCONS_00013225+TCONS_00014403	−
chr7	123634685	123635466	TCONS_l2_00027207	+
chr3	64430158	64431152	TCONS_l2_00020130	−
chr16	49954641	49958065	TCONS_00024301	−
chr1	173204199	173446294	LOC100506023	−
chr7	140773120	140774682	TCONS_00013981	−
chr21	35577356	35698013	TCONS_l2_00017382+TCONS_l2_00017027	+
chr14	71647679	71648822	TCONS_00022778	−
chr8	96981661	96996866	TCONS_00014799	+
chr3	115156547	115157442	TCONS_00006158	+
chr1	113289871	113291621	TCONS_l2_00000491	+
chr5	36487554	36522410	TCONS_l2_00022844	−
chr22	42062545	42063304	TCONS_00029913	−
chr11	111284967	111288911	LOC100132078	−
chr21	29911640	29912677	LINC00161	+
chr16	89978912	89981576	TCONS_l2_00010436	+
chr17	55148883	55162434	TCONS_00025702+TCONS_00025703+TCONS_00026124+TCONS_00025704+TCONS_00026125	−
chr2	37716639	37764397	TCONS_00003637	+
chr12	125226847	125227701	TCONS_00020613	+
chr9	35929476	35937148	TCONS_00015970+TCONS_00015971+TCONS_00015665	+
chr6	112221692	112223054	TCONS_00011357	+
chr6	4466645	4467252	TCONS_00011658	+
chr2	39010342	39012094	TCONS_00004213	−
chr1	219254317	219347130	LOC643723	−
chr12	77086300	77088341	TCONS_00020848	−

chr21	27804917	27807987	TCONS_00029272	−
chr14	102144092	102144767	TCONS_l2_00007922	+
chr1	110426798	110428886	TCONS_00000600	−
chr5	73379893	73399041	TCONS_00009699	−
chr10	29386256	29386746	TCONS_00018148	+
chr22	32510916	32517659	TCONS_00029895+TCONS_00029677	−
chr1	150504637	150509813	TCONS_00001146+TCONS_00002162	+
chr1	56427584	56436730	TCONS_00000952	+
chr5	174178307	174277728	TCONS_00009545+TCONS_00009546	+
chr11	12683884	12685249	TCONS_00019577	−
chr17	25664177	25666026	TCONS_00025337	+
chr19	22715448	22719350	TCONS_00026956+TCONS_00026957+TCONS_00026958	+
chr13	113341524	113344135	TCONS_00022125+TCONS_00022126+TCONS_00022318	−
chr1	47971932	47973315	TCONS_00001488	−
chr4	188454032	188593795	TCONS_l2_00022091+TCONS_l2_00022092+TCONS_ l2_00021476+TCONS_l2_00022093+TCONS_ l2_00021477+TCONS_l2_00022094+TCONS_ l2_00021478+TCONS_00009203	−
chr20	29559468	29565969	TCONS_00028363	−
chr19	51288913	51289379	TCONS_l2_00013180	−
chr14	96107825	96108855	TCONS_00022616	+
chr2	43053477	43054362	TCONS_l2_00014533	−
chr12	116971978	116973135	TCONS_00020258	−
chr21	29094698	29123552	LINC00113	+
chr21	23119293	23169735	TCONS_00028805+TCONS_00028764	+
chr22	33560049	33562425	TCONS_00029417	+
chr4	119348948	119350584	TCONS_00007603	+
chr11	17402306	17403209	TCONS_00019861	+
chr2	62642329	62691205	TCONS_00003701+TCONS_00002902	+
chr12	30908008	30933672	TCONS_00020231	+
chr9	126762615	126763637	TCONS_00016151	+
chr13	45915480	45965618	TPT1-AS1	+
chr22	49246608	49262410	TCONS_00029624	+
chr5	74902446	74906582	TCONS_00009999	+
chr8	136683446	136685646	TCONS_00015179	−
chr3	147913516	147997885	TCONS_00005842	−
chrY	27670208	27673951	TCONS_l2_00030950	−
chr13	95157018	95201597	TCONS_00022086+TCONS_00022304	−
chrX	154578072	154579221	TCONS_l2_00030769+TCONS_l2_00030412	+
chr6	34187155	34188704	TCONS_l2_00024184	+
chr14	36516920	36518080	TCONS_00022707	−
chr2	238521620	238535168	TCONS_00003132	+
chr14	36604916	36645857	PTCSC3	−
chr15	74342347	74346215	TCONS_00023979	+
chr3	64064037	64073039	LOC100287879	−
chr5	81676936	81682335	TCONS_00010007+TCONS_00009252	+
chr4	132777217	132897368	TCONS_l2_00021296+TCONS_l2_00021297+TCONS_ l2_00021973	−

chr4	135898726	135948909	TCONS_00008258	+
chr6	113201574	113204294	TCONS_00011925	+
chr10	77185872	77190266	TCONS_00018246	+
chr12	62072122	62076090	TCONS_l2_00006293	−
chr6	79980095	79980669	TCONS_00011329	+
chr12	77718728	78222393	TCONS_00020482+TCONS_00020483+TCONS_00020484+TCONS_00020485+TCONS_00021168+TCONS_00020486+TCONS_00020487	+
chr22	21642519	21646214	TCONS_l2_00018336	−
chr17	16826104	16828924	TCONS_l2_00011115	−
chr2	88055479	88285309	RGPD1	−
chr10	3474759	3477922	TCONS_00018410	−
chr6	45540498	45545853	TCONS_00011819	+
chr6	170483166	170494926	TCONS_00012633+TCONS_00012032+TCONS_00012634	+
chr12	127115661	127175206	TCONS_00021432+TCONS_00020973+TCONS_00021433+TCONS_00020974+TCONS_00021434+TCONS_00021435+TCONS_00021436	−
chr8	36823936	36839479	TCONS_00014969	−
chr2	28582745	28585818	TCONS_00003618	+
chr12	8717334	8718789	TCONS_00020304+TCONS_00021040	+
chr17	48286333	48291820	TCONS_00025184	+
chr1	230450246	230451789	TCONS_00001323	+
chr13	79980444	79998468	RBM26-AS1	+
chrY	7610631	7690901	TCONS_l2_00030920+TCONS_l2_00031020+TCONS_l2_00030921+TCONS_l2_00030922	−
chr15	101390036	101408768	TCONS_00023271+TCONS_00023564+TCONS_00023565+TCONS_00024030	+
chr1	26542603	26556426	TCONS_l2_00002008+TCONS_l2_00002009+TCONS_l2_00000169+TCONS_l2_00000170+TCONS_l2_00002010+TCONS_l2_00000171+TCONS_l2_00000172+TCONS_l2_00000173	+
chr13	85470379	85476607	TCONS_00022074	−
chr1	209498777	209501877	TCONS_00002227+TCONS_00000377	+
chr8	29672520	29686020	TCONS_00014556	−
chr9	98828121	98864194	LOC158435	+
chr13	68155305	68157361	TCONS_00022047	−
chr17	79361985	79367287	TCONS_00025781	−
chr16	48654151	48656141	TCONS_00024384	+
chr5	43014831	43018913	LOC648987	−
chr20	58907391	58909955	TCONS_00028717+TCONS_00028718+TCONS_00028719	−
chr3	134979870	135158671	TCONS_00006667+TCONS_00005832+TCONS_00005833	−
chr11	27528399	27719718	BDNF-AS	+
chr21	26758133	26804013	LINC00158	−
chr8	5858959	5860627	TCONS_00014916+TCONS_00014917	−
chr11	123077369	123082410	TCONS_00019786+TCONS_00020145+TCONS_00019787	−
chr3	90274756	90291164	TCONS_l2_00019378+TCONS_l2_00019379+TCONS_l2_00019380	−
chr4	1108985	1116952	TMED11P	−
chr2	35024917	35388178	TCONS_00002871+TCONS_00004747	+

chr9	94967638	94972440	TCONS_00015716	+
chr1	39512712	39514128	TCONS_00001990	+
chr7	129165373	129172230	TCONS_00013587	+
chr9	38070201	38074401	TCONS_00016315+TCONS_00016316+TCONS_00016770 +TCONS_00016317	−
chr6	52804524	52810263	TCONS_l2_00025400	−
chr10	96857903	96880251	TCONS_l2_00003718	−
chr17	77791522	77793387	TCONS_00025520	+
chr10	42970939	42990785	LINC00839	+
chr7	158801045	158818929	LINC00689	+
chr7	79327350	79328605	TCONS_00013515	+
chr2	38359779	38367600	TCONS_l2_00015764+TCONS_l2_00014514	−
chr20	4173737	4176600	LOC728228	+
chr8	41041413	41042272	TCONS_00014987	−
chr1	143211528	143275032	TCONS_l2_00002635+TCONS_l2_00002636+TCONS_ l2_00002637+TCONS_l2_00001496+TCONS_ l2_00002638+TCONS_l2_00002639+TCONS_ l2_00001497+TCONS_l2_00001498+TCONS_ l2_00002642+TCONS_l2_00002641+TCONS_l2_00002640	−
chr4	138728860	138737556	TCONS_00007634	+
chr8	1748169	1749522	TCONS_00014578	+
chr7	25870839	25872690	TCONS_00013738+TCONS_00014274+TCONS_00014275	−
chr17	39264470	39272543	TCONS_l2_00010766+TCONS_l2_00010767+TCONS_ l2_00010768+TCONS_l2_00010769+TCONS_l2_00010770	+
chr10	2211334	2218626	TCONS_00017897	−
chr6	126380720	126400068	TCONS_00012595+TCONS_00011946+TCONS_00012596	+
chr17	76275712	76302264	TCONS_00025511	+
chr8	67112686	67121948	TCONS_00015030	−
chr8	55765016	55769672	TCONS_l2_00027766	+
chr11	33835687	33843901	TCONS_00019609+TCONS_00019610	−
chr7	155174771	155189051	TCONS_00012896	+
chr1	229027198	229033564	TCONS_00001854	−
chr4	43342430	43347617	TCONS_00008780+TCONS_00008059+TCONS_00007523	+
chr17	29904909	29912680	TCONS_00025345	+
chr2	164173749	164175329	TCONS_00003923	+
chr3	137490050	137499720	TCONS_l2_00018876+TCONS_l2_00018877+TCONS_ l2_00018879+TCONS_l2_00019889+TCONS_ l2_00019890+TCONS_l2_00019891+TCONS_ l2_00019892+TCONS_l2_00018880	+
chr3	186172770	186211450	LOC253573	−
chr1	103820969	104068139	TCONS_00000592+TCONS_00000593+TCONS_00001614 +TCONS_00000594+TCONS_00000595+TCONS_0000161 5+TCONS_00002423	−
chr20	2651191	2673011	TCONS_00028292+TCONS_00028293	−
chr13	63113850	63148472	TCONS_00022037	−
chr9	99837953	99844227	LOC340508	−
chr1	110828999	110881793	LOC440600	−
chr3	197937116	197955676	TCONS_l2_00020055	+

chr1_ gl000192_ random	429711	440505	TCONS_00030025	−
chr15	80481448	80490229	TCONS_00023479	+
chr8	103895319	103906082	TCONS_00015119	−
chr20	55680797	55682184	TCONS_00027960	+
chr3	13170597	13324482	TCONS_l2_00019176+TCONS_l2_00019177	−
chr3	75273688	75289618	TCONS_00006098	+
chr8	79854913	79855506	TCONS_00014779	+
chr1	248675756	248676345	TCONS_00001364	+
chr11	109651723	109694574	TCONS_00019753	−
chr9	137378128	137437051	TCONS_00016209+TCONS_00016210+TCONS_00016211	+
chr4	147143417	147152283	TCONS_l2_00020804+TCONS_l2_00020805+TCONS_ l2_00020806	+
chr1	63466168	63478142	TCONS_00000534+TCONS_00000533+TCONS_00000532	−
chr10	23380525	23384128	TCONS_00018451	−
chr6	43690523	43705589	TCONS_00011493+TCONS_00012174	−
chr9	81758092	81760715	TCONS_00016368	−
chr2	129821419	129829298	TCONS_00003401	−
chr3	41012032	41013069	TCONS_00005765	−
chr2	98081676	98091049	LOC100506123	−
chr3	43388411	43393454	SNRK-AS1	−
chr20	24860690	24861148	TCONS_00028351	−
chr9	33583983	33585615	TCONS_00015946	+
chr7	106415522	106421611	TCONS_00013055+TCONS_00014191+TCONS_00013558	+
chrX	73633219	73640357	TCONS_00017345	−
chr2	30566102	30627001	TCONS_00004193+TCONS_00005128+TCONS_00004194 +TCONS_00004195	−
chr5	149855079	149865586	TCONS_00010532+TCONS_00011069	−
chr21	44851213	44859330	TCONS_l2_00017085+TCONS_l2_00017086+TCONS_ l2_00017087	+
chr14	92511120	92516990	TCONS_00022381+TCONS_00022382	−
chr6	171030442	171045633	TCONS_00012033+TCONS_00011427+TCONS_00011428	+
chr12	58488696	58491865	TCONS_00020815	−
chr11	38699009	38700126	TCONS_00019277	+
chr13	48510591	48513514	TCONS_00022278	−
chr3	108447296	108447928	TCONS_00006603	−
chr7	91510143	91515201	TCONS_00013528	+
chr8	123790129	123792481	TCONS_00015155	−
chr2	103628237	103636356	TCONS_00002957	+
chr2	123235690	123262687	TCONS_00005244+TCONS_00004431+TCONS_00005245	−
chr9	87650623	87655849	TCONS_00016379+TCONS_00016822	−
chr3	23236301	23244446	TCONS_00006455+TCONS_00007129+TCONS_00005747 +TCONS_00005748	−
chr6	139613296	139645051	TCONS_00012298+TCONS_00012299+TCONS_00011574	−
chr12	124688048	124691914	TCONS_00020963	−
chr2	11239977	11272302	FLJ33534	−
chr22	23804280	23829167	TCONS_l2_00017937+TCONS_l2_00018354+TCONS_ l2_00017938	−

chr15	28567837	28579781	TCONS_l2_00008465+TCONS_l2_00008466	+
chr13	44596471	44604599	LINC00284	+
chrX	102882033	102883590	TCONS_00017446	+
chr1	94610667	94611175	TCONS_00000247	+
chr15	74099035	74102646	TCONS_00023729	−
chrY	21034387	21040114	NCRNA00185	−
chr2	21319126	21319427	TCONS_00004713	+
chr6	106616369	106629165	TCONS_00011910	+
chr15	93135802	93139933	TCONS_00023789+TCONS_00023790	−
chr1	41430713	41432244	TCONS_00001475	−
chr10	125319367	125321719	TCONS_00018847+TCONS_00018372+TCONS_00018848+TCONS_00018849+TCONS_00018373+TCONS_00018374+TCONS_00018375	+
chr2	81689819	81694070	TCONS_00003328	−
chr5	25188427	25306281	TCONS_00009908+TCONS_00009373+TCONS_00010648	+
chr7	389257	391503	TCONS_00013287+TCONS_00013288+TCONS_00014042+TCONS_00014043	+
chr18	2506566	2510776	TCONS_00026393+TCONS_00026394	−
chr22	39608719	39610817	TCONS_00029702	−
chr13	80930453	80937734	TCONS_00021859	+
chr13	74987094	74993252	TCONS_00021517	−
chr11	115605578	115628362	TCONS_00019480	+
chr11	110962786	110968063	TCONS_00019755	−
chr10	52415727	52420028	TCONS_l2_00003984	+
chr3	193848405	193848990	TCONS_00005920	−
chr7	66311611	66355447	TCONS_l2_00025990+TCONS_l2_00025991+TCONS_l2_00025992+TCONS_l2_00025993+TCONS_l2_00025994	+
chr1	803451	812182	FAM41C	−
chr15	62488170	62516293	TCONS_l2_00009132+TCONS_l2_00009133+TCONS_l2_00009509+TCONS_00024132	−
chr13	83229727	83239567	TCONS_00021863	+
chr10	127473652	127474348	TCONS_00018636	−
chr5	86361561	86370138	TCONS_00010980	−
chr11	17088667	17089834	TCONS_00019246	+
chr6	94596618	94599128	TCONS_00011339	+
chr1	12587486	12588702	TCONS_00000840+TCONS_00000154	+
chr12	98879322	98897633	LOC643770	−
chr4	113437964	113439635	TCONS_00008870+TCONS_00008198	+
chr17	21792693	21813708	TCONS_l2_00010629	+
chr14	54235660	54317600	TCONS_00022411	−
chr7	45241393	45243749	TCONS_00014122	+
chr5	2940842	2967742	TCONS_00009844+TCONS_00009845+TCONS_00009329	+
chr17	58200325	58213083	TCONS_l2_00011375+TCONS_l2_00011376+TCONS_l2_00011377+TCONS_l2_00011378+TCONS_l2_00011379+TCONS_l2_00011380+TCONS_l2_00011381	−
chr4	137717877	138133953	TCONS_00007845+TCONS_00007846	−
chr3	107853894	107857879	TCONS_00006148	+
chr17	25745031	25758649	TBC1D3P5	+
chr5	126565033	126567810	TCONS_00010083	+

chr1	22744726	22745435	TCONS_00001415	−
chr13	40755946	40763167	LINC00332	+
chrY	9544433	9552871	TTTY7B	−
chr11	50257776	50313168	TCONS_l2_00004519+TCONS_l2_00004520+TCONS_l2_00004521+TCONS_l2_00004522	+
chr2	126867676	126875562	TCONS_00003398	−
chr17	19542441	19543863	TCONS_00025587	−
chr5	177357466	177360935	TCONS_l2_00022657	+
chr19	32583596	32596822	TCONS_00026845	−
chrY	1801477	1824878	TCONS_00017601+TCONS_00017602	+
chr12	90341471	90343569	TCONS_00020510	+
chr10	125702798	125703913	TCONS_00019041+TCONS_00018624	−
chr5	91745963	91996570	TCONS_00009725+TCONS_00009726+TCONS_00009727	−
chr13	114629487	114631964	LINC00565	−
chr9	132020633	132023860	TCONS_00015756+TCONS_00015757	+
chr7	45808705	45814375	TCONS_l2_00025845+TCONS_l2_00025846	+
chr7	27401265	27449622	TCONS_00013398+TCONS_00012984+TCONS_00014097+TCONS_00013399	+
chr10	2342513	2357268	LINC00701	−
chr7	79085481	79096779	TCONS_00013201+TCONS_00014342	−
chr17	53403118	53405152	TCONS_00025244	−
chr4	120038634	120049505	TCONS_00009112+TCONS_00008560+TCONS_00009111+TCONS_00008559+TCONS_00008561	−
chr8	32785452	32903006	TCONS_l2_00027706+TCONS_l2_00027707+TCONS_l2_00028478+TCONS_l2_00027708+TCONS_l2_00027709	+
chr4	13841060	13842697	TCONS_00009029	−
chr12	114514951	114532989	TCONS_00020569	+
chr16	86750479	86775053	TCONS_00024497+TCONS_00024498+TCONS_00024898+TCONS_00024499	+
chr19	9944974	9945573	TCONS_00027178	−
chr2	130324223	130351825	TCONS_00004443+TCONS_00004444	−
chr1	149230539	149232553	TCONS_00001142+TCONS_00000055+TCONS_00002156	+
chr9	44181550	44183417	TCONS_00016323	−
chr21	25227798	25231436	TCONS_00028976	+
chr10	122738902	122740689	TCONS_00018360	+
chr1	194118603	194167824	TCONS_l2_00002271	+
chr5	85814416	85815676	TCONS_00009438	+
chr10	30944418	30946464	TCONS_00017807	+
chr9	67288343	67334319	TCONS_l2_00028766+TCONS_l2_00028767+TCONS_l2_00028768+TCONS_l2_00029755+TCONS_l2_00028769+TCONS_l2_00028770	+
chr19	9293020	9293791	TCONS_l2_00012837	−
chr2	165509841	165522523	TCONS_00004484	−
chr21	10186569	10215575	TCONS_l2_00017125+TCONS_00029073	−
chrY	9590765	9611898	TTTY1	−
chr10	112779678	112782449	TCONS_00018817	+
chr2	106986278	106992912	TCONS_l2_00013877	+
chr7	123974348	123993469	TCONS_00013071	+
chr6	38681827	38682993	TCONS_00011301	+

chr1	11371804	11374861	TCONS_00001387	−
chr7	114701591	114871409	TCONS_l2_00027461	−
chr10	4698348	4704606	LINC00705	+
chr20	59044635	59048080	TCONS_00028720+TCONS_00028721+TCONS_00028722	−
chr6	6759609	6760176	TCONS_00011669	+
chr5	66915619	67107273	TCONS_l2_00023403+TCONS_l2_00022344+TCONS_ l2_00022345+TCONS_l2_00022346+TCONS_l2_00023404	+
chr6	93098931	93105082	TCONS_00011889	+
chrX	134807294	134819989	TCONS_l2_00030863+TCONS_l2_00030864	−
chr2	168149681	168414843	TCONS_00003044	+
chr12	6404577	6405936	TCONS_00020288	+
chr16	50175043	50178523	TCONS_00024639	−
chr8	133865865	133867309	TCONS_00014863	+
chr5	179084763	179095672	TCONS_00009559+TCONS_00010208+TCONS_00009560 +TCONS_00009561+TCONS_00010209	+
chr2	114334959	114336429	FAM138B	+
chr8	126953411	126967652	TCONS_l2_00027973	+
chr9	37362834	37363153	TCONS_00015974	+
chr2	98104121	98104688	TCONS_00004345	−
chr2	99348118	99368056	TCONS_00003790	+
chr8	86864119	86866829	TCONS_00015064	−
chr5	74213551	74319753	TCONS_l2_00023792	−
chr21	15033887	15050511	TCONS_l2_00016970	+
chr5	61044433	61086886	TCONS_00010701+TCONS_00009979+TCONS_00009980	+
chr19	28281401	28284848	LINC00662	−
chr12	102349395	102356105	TCONS_00020538	+
chr2	67148642	67149631	TCONS_00004294+TCONS_00003314+TCONS_00004293	−
chr9	93719542	93727675	TCONS_00015845	−
chr3	190500012	190500863	TCONS_00006733	−
chr17	75543023	75561103	LOC100507351	+
chr1	161389333	161390169	TCONS_00001706	−
chr21	19849676	19858917	TCONS_00028875	−
chr20	61732644	61735737	HAR1A	+
chr1	89510013	89512879	TCONS_00001018	+
chr13	25767245	25784433	TCONS_l2_00007372	+
chr18	77437294	77439308	TCONS_00026747	−
chr7	157230890	157232826	TCONS_00014460+TCONS_00014459	−
chr7	135665698	135705544	TCONS_00013605	+
chr8	130363938	130692485	TCONS_00014564	−
chr17	55777833	55778887	TCONS_00025441	+
chrY	15229651	15261435	TCONS_00017637	+
chr4	138466885	138524584	TCONS_00007633	+
chr7	137848186	137849383	TCONS_00013088	+
chr6	4341703	4347376	TCONS_00012058+TCONS_00012644+TCONS_00012645 +TCONS_00012646+TCONS_00012647+TCONS_0001264 8+TCONS_00011438	−
chr20	49944701	49948181	TCONS_00028442	−
chr4	4711947	4716200	TCONS_00008413	−

chr1	167145154	167165029	TCONS_00000667+TCONS_00000668+TCONS_00000669 +TCONS_00000670	−
chr1	155966114	155972355	TCONS_00000643	−
chr12	88045659	88064325	TCONS_00020861	−
chr4	142161893	142199943	TCONS_00007859	−
chr6	50429754	50489408	TCONS_00011171+TCONS_00011311	+
chr17	67708659	67842667	TCONS_00025474	+
chr13	112849403	112850190	TCONS_00022122	−
chr15	39460957	39469127	TCONS_00023357	+
chr5	68128412	68257985	TCONS_l2_00023767	−
chr3	40758901	40762498	TCONS_00006492	−
chr4	92809037	92825486	TCONS_00007573	+
chr18	76555629	76556807	TCONS_00026583+TCONS_00026741	−
chr13	48337599	48338514	TCONS_l2_00006843	+
chr10	103012578	103023307	TCONS_00017749	−
chr12	67964817	67983740	TCONS_00020825	−
chr13	19433967	19434882	TCONS_l2_00007350	+
chr1	109754802	109756393	TCONS_00001091	+
chr5	269973	271631	TCONS_00009573	−
chr2	91750767	91769187	TCONS_l2_00013779+TCONS_l2_00013780+TCONS_ l2_00013781+TCONS_l2_00013782+TCONS_ l2_00013783+TCONS_l2_00013784	+
chr19	36370792	36376739	TCONS_00027327	−
chr5	66563850	66566010	TCONS_00009410	+
chr9	137185777	137187243	TCONS_00015890	−
chr9	107752367	107754062	TCONS_00016452+TCONS_00015860	−
chr11	63538430	63580588	TCONS_00019653	−
chr9	78194585	78203464	TCONS_00015697	+
chr12	51365312	51366728	TCONS_00020784	−
chr10	131076515	131089986	TCONS_00017890	+
chr4	167309853	167447271	TCONS_00007893	−
chr19	32562520	32562996	TCONS_00027298	−
chr1	236105853	236107105	TCONS_l2_00002808	−
chr2	113576774	113581878	TCONS_00004393	−
chr15	20723929	20727148	TCONS_l2_00009397+TCONS_l2_00008401	+
chr13	67946519	67954112	TCONS_l2_00007421+TCONS_l2_00006899	+
chr18	10017463	10020821	TCONS_00026598	+
chr17	19328662	19364306	TCONS_l2_00011128+TCONS_l2_00011129+TCONS_ l2_00011628+TCONS_l2_00011130+TCONS_l2_00011629	−
chr10	118036310	118040487	TCONS_00018346	+
chr2	121481838	121491735	TCONS_00003837	+
chr20	22989738	23011443	TCONS_00027885+TCONS_00028339+TCONS_00028640	−
chrY	6225260	6229454	TCONS_00017622	−
chr12	33466706	33470504	TCONS_00020757	−
chr2	78015508	78020213	TCONS_l2_00015391	+
chr6	47824777	47838098	TCONS_00011825	+
chr11	92973271	92985208	TCONS_00019441	+
chr10	110759920	110778745	TCONS_00018332	+

chr5	21616385	21676223	TCONS_00009902	+
chrX	1691556	1704599	TCONS_00017120	+
chr3	125366455	125369330	TCONS_00006194+TCONS_00006195	+
chr4	131297384	131307526	TCONS_00007622	+
chr4	10686627	10699285	TCONS_00007471	+
chr16	14112738	14114934	TCONS_00024263+TCONS_00024589	−
chr21	24733426	24757156	D21S2088E	−
chr10	82409553	82413488	TCONS_00017847	+
chr12	76407804	76408563	TCONS_00020475	+
chr15	28579830	28582388	TCONS_l2_00008467	+
chr1	4000672	4012643	LOC728716	+
chr19	37168290	37177738	TCONS_00027012	+
chr12	48606567	48639062	TCONS_00020406+TCONS_00020407	+
chr2	151839020	151859988	TCONS_00003036	+
chr4	60416860	60496042	TCONS_00007771	−
chr17	12453285	12540504	LINC00670	+
chrX	41907136	41914089	TCONS_00017055	−
chr1	98538887	98675034	TCONS_l2_00000435+TCONS_l2_00000436	+
chr2	739751	754320	TCONS_l2_00013420+TCONS_l2_00013421+TCONS_ l2_00013422+TCONS_l2_00015217+TCONS_ l2_00013423+TCONS_l2_00015218+TCONS_ l2_00013424+TCONS_l2_00013425+TCONS_l2_00013426	+
chr16	76733678	76737172	TCONS_00024471	+
chr8	64681988	64698054	LOC286184	+
chr5	91378414	91815133	TCONS_00009460+TCONS_00009256+TCONS_00010039 +TCONS_00009461	+
chr3	197363226	197374168	TCONS_00006770+TCONS_00006771+TCONS_00006772	−
chr13	47402276	47407401	TCONS_00021786	+
chr9	96619365	96620615	TCONS_00015718	+
chr12	122501254	122508477	TCONS_l2_00005949+TCONS_l2_00005950+TCONS_ l2_00005951+TCONS_l2_00005952+TCONS_ l2_00005953+TCONS_l2_00005954+TCONS_ l2_00005955+TCONS_l2_00005956	+
chr2	216734646	216735241	TCONS_00003106	+
chr16	66926840	66930900	TCONS_00024437+TCONS_00024438	+
chr5	130588733	130593138	TCONS_00010089+TCONS_00010090+TCONS_00010091	+
chr16	3056586	3057374	TCONS_00024547	−
chr13	30446750	30462542	TCONS_00021535	+
chr9	67270215	67289492	AQP7P1	−
chr6	148059208	148064467	TCONS_00012327+TCONS_00011589	−
chr13	51095069	51101585	TCONS_00021652	−
chr10	3876142	3876920	TCONS_00017770	+
chr6	147162525	147525750	STXBP5-AS1	−
chr20	46845008	46860369	TCONS_00028423	−
chr19	23257999	23332763	TCONS_l2_00012415+TCONS_l2_00012416+TCONS_ l2_00012417+TCONS_l2_00012418+TCONS_ l2_00012419+TCONS_l2_00012420+TCONS_l2_000124 21+TCONS_00026966+TCONS_l2_00013309+TCONS_ l2_00012423	+

chr19	35339893	35351417	TCONS_00027734+TCONS_00027735+TCONS_00027309+TCONS_00027310	−
chr2	197502840	197504260	TCONS_00004541	−
chr14	45234277	45237334	TCONS_00022730+TCONS_00022731	−
chr3	184474177	184490943	TCONS_l2_00020288+TCONS_l2_00019640	−
chr15	87965967	87973948	TCONS_00023770+TCONS_00023771	−
chr12	131895171	131896691	TCONS_00021007	−
chr2	88045926	88047354	TCONS_00003759	+
chr10	29085001	29097156	TCONS_l2_00002935+TCONS_l2_00003923	+
chr6	71318500	71318880	TCONS_l2_00024815	−
chr1	161401998	161410779	TCONS_l2_00001586	−
chr7	126990182	126991577	TCONS_00013076	+
chr7	57277500	57290593	TCONS_l2_00026595+TCONS_l2_00027368+TCONS_l2_00026598+TCONS_l2_00026596+TCONS_l2_00027369	−
chr11	45743858	45746106	LOC100507384	+
chr16	87351111	87351640	TCONS_00024899+TCONS_00024900	+
chr20	61747569	61750559	TCONS_00028587+TCONS_00028263	+
chr4	119388742	119414716	TCONS_l2_00020697	+
chrX	64586534	64618554	TCONS_00017426+TCONS_00017189	+
chr8	55696076	55750199	TCONS_l2_00027764+TCONS_l2_00027765	+
chr10	121380628	121381179	TCONS_l2_00003283	+
chr8	685887	686579	TCONS_00014570	+
chr20	10972035	10981873	TCONS_00028108+TCONS_00028500+TCONS_00028501	+
chr1	143231332	143232085	TCONS_00001137	+
chr3	50706849	50709456	TCONS_00006023	+
chr9	131213110	131214331	TCONS_00016482	−
chr12	108233090	108240199	TCONS_00020911+TCONS_00021404	−
chr16	32941725	32950672	TCONS_l2_00009694	+
chr5	42985140	42993435	TCONS_00010320+TCONS_00010926+TCONS_00009277+TCONS_00010321	−
chr1	210463516	210466574	TCONS_00001798+TCONS_00001799	−
chrX	84465712	84474704	TCONS_00016998+TCONS_00017205	+
chr4	16115965	16122540	TCONS_00008444	−
chr16	75741011	75743679	TCONS_l2_00010287	−
chr2	89065419	89106109	ANKRD36BP2	+
chr6	137303296	137314368	NHEG1	−
chr5	50176069	50177481	TCONS_00009957	+
chr2	51427647	51452705	TCONS_l2_00014568+TCONS_l2_00015797+TCONS_l2_00014569	−
chr16	17107604	17119655	TCONS_00024339	+
chr18	69402399	69432114	TCONS_00026550	−
chr2	101768122	101771872	TCONS_00002716	+
chr21	41099682	41102607	TCONS_00028774+TCONS_00028849	+
chr3	83280678	83282224	TCONS_00006111	+
chr10	119232726	119236135	TCONS_l2_00004278	−
chr4	9701064	9705109	TCONS_l2_00020407	+
chr1	16847189	16848303	TCONS_00000157+TCONS_00000848	+
chr5	139531024	139532171	TCONS_00010505	−

chr11	76475920	76479444	TCONS_00019713	−
chrX	9217960	9243246	TCONS_l2_00030659	+
chr4	166651932	166664223	TCONS_00007892	−
chr12	67611	69070	TCONS_00020181	+
chr7	128166278	128170972	TCONS_00013585	+
chr11	7749640	7751004	TCONS_00019217	+
chr13	91763974	91793697	TCONS_00022209+TCONS_00021871	+
chr3	195311256	195327734	TCONS_00005711	+
chr10	81598817	81600004	TCONS_00018253	+
chr2	131451087	131476463	TCONS_l2_00015483+TCONS_l2_00015484+TCONS_ l2_00015485+TCONS_l2_00013985+TCONS_ l2_00013986+TCONS_l2_00013987+TCONS_l2_00015486	+
chr15	61696313	61703410	TCONS_00023434+TCONS_00023435	+
chr14	101913790	101915200	TCONS_00022637	+
chr4	185427373	185458712	TCONS_00009192	−
chr4	153021906	153025789	TCONS_00007661+TCONS_00007410	+
chr16	32274231	32276669	TCONS_00024267	−
chr16	64294313	64295136	TCONS_00024435	+
chr7	136090011	136122158	TCONS_00013086+TCONS_00013087	+
chr2	221747474	221802061	TCONS_00004596	−
chr11	93932429	93932887	TCONS_00019731	−
chrX	39260506	39294078	TCONS_00017054	−
chr12	43038040	43040281	TCONS_00020763	−
chr2	66610438	66619582	TCONS_l2_00015360	+
chr10	33973683	33975992	TCONS_00017810	+
chr9	134430077	134433446	TCONS_l2_00029094	+
chr12	104756233	104762789	TCONS_00020550	+
chr1	188477669	188505596	TCONS_00000353	+
chr6	29465286	29478333	TCONS_l2_00025119+TCONS_l2_00024139	+
chr9	118369851	118505348	TCONS_00016469+TCONS_00016862	−
chrX	79483990	79565796	TCONS_l2_00030834+TCONS_l2_00030544+TCONS_ l2_00030545	−
chr3	18626907	18628395	TCONS_00005961	+
chr7	100434936	100450238	TCONS_00013214	−
chr10	13569855	13572257	TCONS_l2_00002890	+
chr5	172044800	172055726	TCONS_00010170	+
chr1	119802888	119870538	TCONS_00000620+TCONS_00002455+TCONS_00001644	−
chr22	37284331	37285494	TCONS_00029694	−
chr9	95909322	95910838	TCONS_00015717+TCONS_00016075	+
chr4	186937392	186941235	TCONS_00008698	−
chr1	232310678	232315859	TCONS_00000761	−
chr10	33247773	33371030	TCONS_l2_00003931+TCONS_l2_00003932+TCONS_ l2_00002962+TCONS_l2_00002963	+
chr22	29227139	29227916	TCONS_00029886	−
chr14	29736872	29744334	TCONS_00022697	−
chr15	89584261	89622992	TCONS_00023492+TCONS_00023493+TCONS_00023494 +TCONS_00024002+TCONS_00023495	+
chr15	90889763	90892679	GABARAPL3	−

chr16	9761808	9773387	TCONS_00024327	+
chr18	74985916	75042311	TCONS_00026380	+
chr13	80367943	80369398	TCONS_00021854	+
chr8	55291718	55293397	TCONS_00014700	+
chr15	92155314	92180308	TCONS_00023507+TCONS_00024008	+
chr1	84464970	84466352	TCONS_00001545	−
chr16	8345918	8363665	TCONS_00024322+TCONS_00024804	+
chr18	8559865	8563192	TCONS_00026399	−
chr17	26575812	26577914	TCONS_00025600	−
chr6	81128031	81175644	TCONS_00011870+TCONS_00011871+TCONS_00012537+ TCONS_00011332+TCONS_00011872+TCONS_00011873+ TCONS_00011333+TCONS_00011334+TCONS_00011874	+
chr12	58959743	59175567	TCONS_l2_00006655+TCONS_l2_00006289+TCONS_ l2_00006290+TCONS_l2_00006291+TCONS_l2_00006292	−
chr5	100348032	100367064	TCONS_00010427	−
chr8	145703379	145705868	TCONS_00015381	+
chr6	132406224	132420419	TCONS_00011370+TCONS_00011371	+
chr10	54711605	54715218	TCONS_l2_00003584	−
chr13	112749810	112762329	TCONS_00021911+TCONS_00021601	+
chr6	164767522	164769677	TCONS_00011612	−
chr10	9012088	9015431	TCONS_00017786	+
chr6	12583147	12585636	TCONS_00011454	−
chr5	42918466	42920126	TCONS_00010319	−
chr1	24523479	24526136	TCONS_00001425	−
chr5	30101586	30102332	TCONS_00010293	−
chr10	115244764	115252063	TCONS_00018604+TCONS_00018605	−
chr1	182055686	182059247	TCONS_00000346	+
chr1	4192617	4194185	TCONS_00000823	+
chr22	26557876	26559180	TCONS_00029458	−
chr10	30964483	30980403	TCONS_l2_00002951+TCONS_l2_00002952+TCONS_ l2_00002953	+
chr4	188534086	188647874	TCONS_00008373+TCONS_00008973+TCONS_00007696 +TCONS_00008374+TCONS_00008974+TCONS_0000897 5+TCONS_00007697+TCONS_00008976+TCONS_000083 75+TCONS_00008376+TCONS_00008977	+
chr3	38462818	38467813	TCONS_l2_00018503	+
chr22	24340595	24347258	GSTTP1	−
chr2	13022134	13066803	TCONS_00004149	−
chr15	48095586	48138432	TCONS_00023669+TCONS_00023670+TCONS_00024118 +TCONS_00023671	−
chr10	43008961	43048280	ZNF37BP	−
chr10	61348908	61371006	TCONS_l2_00003986+TCONS_l2_00003063	+
chr6	78359953	78636743	TCONS_l2_00024299+TCONS_l2_00024300	+
chr12	5385995	5487416	TCONS_00020281+TCONS_00020282	+
chr15	85047738	85060078	GOLGA6L5	−
chr21	14721585	14724906	TCONS_l2_00016966	+
chr22	27617310	27622760	TCONS_l2_00018380+TCONS_l2_00018381+TCONS_ l2_00018382+TCONS_l2_00017993	−
chr19	5558185	5581226	TCONS_00026823	+

chr5	553527	554002	TCONS_00010227	−
chr9	67917363	67925019	TCONS_l2_00029341	−
chr20	5445342	5451680	TCONS_00028304	−
chr10	87191699	87192271	TCONS_00018013	−
chr17	1243628	1244183	TCONS_00025268	+
chr19	12301794	12304786	TCONS_00026839	−
chr9	72402048	72409255	TCONS_00016002	+
chr1	225888415	225904316	TCONS_00001306+TCONS_00001307+TCONS_00001308 +TCONS_00002241	+
chr15	59698610	59702722	TCONS_l2_00009129	−
chr9	77791049	77794685	TCONS_00016018	+
chr4	120597484	120610372	TCONS_00008211	+
chr13	21808707	21809343	TCONS_00021528	+
chr7	123175278	123175887	TCONS_00014197+TCONS_00013572	+
chr2	21910306	21933524	LOC645949	−
chr2	13863173	13898277	TCONS_00002687	+
chr17	40346647	40348097	TCONS_00025392	+
chr10	8939952	8956559	TCONS_00017785	+
chr13	64311568	64316701	OR7E156P	+
chr14	44861702	44914002	TCONS_00022726+TCONS_00022727	−
chr2	241520914	241521560	TCONS_00004632	−
chr2	13106908	13147138	LOC100506474	−
chr1	27852316	27857072	TCONS_00000171	+
chr14	52536230	52540595	TCONS_00022504	+
chr1	88817765	88821171	TCONS_00001563	−
chr6	169343891	169358002	TCONS_00012367	−
chr14	101402075	101402816	TCONS_00022632	+
chr7	55827936	55846856	TCONS_l2_00025879+TCONS_l2_00025880+TCONS_ l2_00025881+TCONS_l2_00025882+TCONS_l2_00025883	+
chrX	103230502	103232785	H2BFXP	+
chr1	207589903	207597521	TCONS_00001253	+
chr1	947377	948573	TCONS_00000447	−
chr12	14898794	14903603	TCONS_00020723	−
chr7	82797450	82804744	TCONS_00014349+TCONS_00013876	−
chr18	52773135	52791537	TCONS_00026207+TCONS_00026718+TCONS_00026524	−
chr12	59832093	59835248	TCONS_00020451	+
chr13	51764578	51766020	TCONS_00022022	−
chr20	16588321	16620313	TCONS_00028113+TCONS_00028504	+
chr12	114182382	114211488	TCONS_00020222+TCONS_00020223	−
chr18	69449270	69454242	TCONS_00026551	−
chr16	25078258	25080275	TCONS_00024286+TCONS_00024246	+
chr5	18704542	18746282	TCONS_00009612	−
chr5	145780544	145786922	TCONS_00009536	+
chr8	142264400	142276581	TCONS_00015535	−
chr10	91675246	91717130	TCONS_00017745	−
chr15	93632685	93763011	TCONS_00023515+TCONS_00023516+TCONS_00024014 +TCONS_00023517	+

chr2	45148216	45166338	TCONS_00003267+TCONS_00003268+TCONS_00003269+TCONS_00003270	−
chr1	73771635	73820934	TCONS_00000985+TCONS_00002067+TCONS_00002066+TCONS_00002065+TCONS_00002064+TCONS_00000046+TCONS_00000228+TCONS_00000229+TCONS_00000230+TCONS_00000231	+
chr16	34742761	34758882	TCONS_l2_00009710	+
chr1	188674754	188680011	TCONS_00000701	−
chr17	55043460	55048783	TCONS_00025439	+
chr20	5466188	5493123	TCONS_00028097+TCONS_00028098+TCONS_00028492+TCONS_00028491+TCONS_00028490+TCONS_00028493	+
chr7	9765726	9767835	TCONS_l2_00025651	+
chr6	28996286	29003941	TCONS_00012135	−
chr10	93542596	93558048	TCONS_00018997+TCONS_00018998+TCONS_00018023+TCONS_00018024	−
chr2	64530721	64568781	TCONS_00005167+TCONS_l2_00015820+TCONS_l2_00014613+TCONS_00003303	−
chr1	229228562	229230652	TCONS_00000749	−
chr21	37756425	37757219	TCONS_00029009	+
chr11	22467261	22513556	TCONS_00019591	−
chr1	44498210	44499602	TCONS_00002376	−
chr20	32905454	32943260	TCONS_l2_00016575	−
chr14	101426541	101429203	TCONS_00022633	+
chr7	48748092	48750837	TCONS_00013442	+
chr8	77318799	77436562	TCONS_00014767+TCONS_00014768+TCONS_00014769	+
chrX	3782439	3799884	TCONS_l2_00030776	−
chr10	125333743	125343283	TCONS_00017885	+
chr1	111030302	111032880	TCONS_00000602	−
chr3	172142213	172144915	TCONS_l2_00019618	−
chr16	13352217	13444277	TCONS_00024332	+
chr12	105905269	105939323	TCONS_l2_00005873	+
chr2	38095119	38099030	TCONS_00004750	+
chr18	14477954	14498705	CXADRP3	−
chr2	46726369	46727385	TCONS_00004244+TCONS_00005144+TCONS_00004245	−
chr6	15161164	15184930	TCONS_00012088	−
chr19	46713499	46718094	DKFZp434J0226	+
chr4	99706580	99708527	TCONS_00008533	−
chr2	65158445	65161625	TCONS_00003711	+
chr13	39107480	39119436	TCONS_00021639	−
chr19	23491584	23500868	TCONS_00027504	+
chr11	5127292	5128760	TCONS_00019166	−
chr10	48919831	48922964	TCONS_l2_00003552	−
chr17	67353284	67355360	TCONS_00025743	−
chr10	126920240	126952165	TCONS_00018380+TCONS_00018381	+
chr20	22201192	22203921	TCONS_00027913	+
chr13	63149147	63167507	TCONS_00021809	+
chr4	139694699	139722793	TCONS_00007641+TCONS_00007642+TCONS_00007643	+
chr5	43874469	43886730	TCONS_00009393	+

chr19	21744136	21752048	TCONS_00027223+TCONS_00027224	−
chr7	104650989	104654588	LOC100216545	−
chr2	114727055	114729927	TCONS_00004414	−
chr14	23956450	23978797	TCONS_00022427	+
chr2	157173940	157174852	TCONS_00005257	−
chr12	39313439	39317740	TCONS_00020378	+
chr11	118789488	118796280	TCONS_00019776	−
chr8	20809080	20831345	TCONS_00014944+TCONS_00014946+TCONS_00014945	−
chr5	14971051	14993054	TCONS_00009878+TCONS_00010630+TCONS_00009879	+
chr8	16325251	16353693	TCONS_00014939	−
chr22	42548208	42551006	TCONS_l2_00018409+TCONS_l2_00018072	−
chr18	9894757	9896018	TCONS_00026415	−
chr9	120224572	120228538	TCONS_00015876	−
chr1	198566854	198567998	TCONS_00000711	−
chr13	19184438	19185815	TCONS_00021606	−
chr4	82523897	82965397	TCONS_l2_00021192+TCONS_l2_00021909+TCONS_l2_00021910+TCONS_l2_00021911+TCONS_l2_00021912+TCONS_l2_00021193	−
chr1	87819210	87837338	LOC100505768	−
chr11	56615954	56645554	TCONS_00019093	+
chr14	38033434	38048129	TCONS_00022476	+
chrX	102204237	102219282	TCONS_00017439+TCONS_00017440+TCONS_00017441	+
chr10	114578564	114590547	TCONS_00018337	+
chr6	93299040	93299804	TCONS_00012225	−
chrX	114999954	115005243	TCONS_00017455	+
chr19	46676750	46683946	TCONS_00027380	−
chr11	94818143	94821880	TCONS_00019733+TCONS_00019734	−
chr4	182178898	182186252	TCONS_00008673+TCONS_00007918	−
chr16	48536186	48538305	TCONS_00024381	+
chr14	105542841	105553887	TCONS_l2_00008281	−
chr6	158211221	158226738	TCONS_00012342	−
chr20	12915491	12919905	TCONS_l2_00016159+TCONS_l2_00016734	+
chr3	53112200	53120488	TCONS_00006513+TCONS_00007175	−
chr9	93736514	93747020	TCONS_00016402	−
chr20	18248713	18253698	TCONS_00028505	+
chr3	148994297	149009587	TCONS_l2_00020218	−
chr15	55219997	55384319	TCONS_l2_00009437+TCONS_00023416	+
chr1	221728934	221736978	TCONS_00001829	−
chr21	29488417	29600986	TCONS_l2_00017475+TCONS_l2_00017476+TCONS_l2_00017184+TCONS_l2_00017185	−
chr13	19836940	19919113	ANKRD26P3	−
chr1	224770788	224785257	TCONS_l2_00000862	+
chr19	31619016	31620542	TCONS_00027290	−
chr13	100150594	100153447	TCONS_00022098+TCONS_00021693	−
chr9	122809390	122829429	TCONS_00016148+TCONS_00016149+TCONS_00016678	+
chr1	110138847	110140403	TCONS_00000270	+
chr9	92214916	92215681	TCONS_00016398	−
chr4	16290104	16303012	TCONS_00008002	+

chr5	40308112	40313013	TCONS_00010311	−
chr7	134046101	134049381	TCONS_00013974	−
chrX	6585906	6639585	TCONS_00017036	−
chr5	177387462	177392855	TCONS_00010575+TCONS_00010576+TCONS_00011098	−
chr1	235087871	235105809	TCONS_00001881+TCONS_00001882+TCONS_00000118 +TCONS_00001883+TCONS_00000765+TCONS_0000076 6+TCONS_00000767+TCONS_00001884+TCONS_000018 85+TCONS_00000768+TCONS_00001886	−
chr9	69065461	69130929	TCONS_l2_00028789+TCONS_l2_00028790+TCONS_ l2_00028791+TCONS_l2_00028796+TCONS_ l2_00028795+TCONS_l2_00028794+TCONS_ l2_00028793+TCONS_l2_00028797+TCONS_ l2_00028798+TCONS_l2_00028799+TCONS_ l2_00028800+TCONS_l2_00029762+TCONS_ l2_00029763+TCONS_l2_00028801+TCONS_ l2_00028802+TCONS_l2_00028803+TCONS_ l2_00028804+TCONS_l2_00028805+TCONS_ l2_00028806+TCONS_l2_00028807+TCONS_ l2_00028808+TCONS_l2_00028809+TCONS_ l2_00028810+TCONS_l2_00028811+TCONS_ l2_00028812+TCONS_l2_00028813	+
chr6	47413501	47415423	TCONS_00011824	+
chr19	22511312	22514194	TCONS_00027229	−
chr16	90168702	90204399	TCONS_l2_00010437+TCONS_l2_00009911	+
chr10	26924675	26924975	TCONS_00018456	−
chr2	234684370	234688428	TCONS_l2_00015623	+
chr4	156881332	156895608	TCONS_00008303	+
chr6	148338558	148342110	TCONS_00011590	−
chr21	29010981	29047091	TCONS_00028982+TCONS_00028983+TCONS_00028825	+
chr6	1458802	1555481	TCONS_l2_00024558+TCONS_l2_00024559+TCONS_ l2_00024560+TCONS_l2_00024561+TCONS_ l2_00024562+TCONS_l2_00024563+TCONS_l2_00024564	−
chr10	134779038	134790001	LOC399829	+
chr10	5667061	5668697	TCONS_l2_00002856	+
chrX	102997792	103001839	TCONS_00017449	+
chr13	85685979	85722284	TCONS_00022076+TCONS_00022302+TCONS_00022077	−
chr20	18305463	18309622	TCONS_l2_00016492	−
chr4	64627275	64651637	TCONS_00008093+TCONS_00008094	+
chr13	19114637	19124837	TCONS_00021521	+
chr2	120030214	120040231	TCONS_00004420+TCONS_00004421+TCONS_00004422	−
chr12	115748571	115759015	TCONS_00020937	−
chr7	156775941	156777103	TCONS_00013655	+
chr1	40932412	40938148	TCONS_00000506	−
chrY	25082602	26753172	TTTY4	+
chr5	7289852	7305002	TCONS_l2_00022176+TCONS_l2_00022177+TCONS_ l2_00022178	+
chr2	66924257	66930359	TCONS_00003311+TCONS_00003312+TCONS_00003313	−
chr12	83850418	83866379	TCONS_00020859	−
chr9	82604786	82649802	TCONS_00016023+TCONS_00015698	+
chr2	217719105	217719914	TCONS_00004575+TCONS_00003497	−
chr7	35535631	35549873	TCONS_00013156	−

chrX	70980625	70981509	TCONS_00017067	−
chrY	8517467	8518535	TCONS_00017632	+
chr10	2543389	2612742	TCONS_00018083+TCONS_00018084+TCONS_00018085 +TCONS_00017760+TCONS_00017761	+
chr10	113201149	113236372	TCONS_00018336	+
chr3	15193350	15198825	TCONS_l2_00020087	−
chr17	14198829	14200917	TCONS_00025210	−
chr12	127836280	127836889	TCONS_00020646	+
chr12	54582954	54584598	TCONS_00020438	+
chr8	60473892	60506377	TCONS_00015017	−
chr3	194681821	194686205	TCONS_00006373	+
chr18	3347688	3350585	TCONS_00026395	−
chr6	133125513	133134602	TCONS_00012278	−
chr1	1944652	1946969	TCONS_00000074+TCONS_00001373	−
chr22	20956763	20957575	TCONS_l2_00017898	−
chr20	46523116	46528819	TCONS_00028418+TCONS_00028419	−
chr10	81420645	81439751	TCONS_00017844+TCONS_00017843+TCONS_00018250 +TCONS_00018766+TCONS_00018767+TCONS_0001825 1+TCONS_00018768	+
chr17	34897577	34900643	TCONS_l2_00011553	+
chr6	86367932	86373867	TCONS_l2_00024837	−
chr10	87209349	87251753	TCONS_00017850	+
chr2	66462509	66463345	TCONS_00003309	−
chr4	38281624	38289952	TCONS_00008776+TCONS_00008044+TCONS_00008045 +TCONS_00008046+TCONS_00007513	+
chr8	98413783	98414097	TCONS_00014802	+
chr13	80615858	80626564	TCONS_00022063+TCONS_00022064	−
chr2	60577730	60580151	TCONS_00003294	−
chr10	93542607	93557593	TCONS_00018285	+
chr3	96336029	96337039	TCONS_00006915+TCONS_00006916+TCONS_00006917 +TCONS_00006918+TCONS_00006919	+
chr1	50717182	50723876	TCONS_00001490+TCONS_00002380	−
chr2	108783601	108834297	TCONS_l2_00014767+TCONS_l2_00015893+TCONS_ l2_00014768	−
chr2	196434352	196478715	TCONS_l2_00016053+TCONS_l2_00015044+TCONS_ l2_00015045	−
chr3	81043000	81146631	TCONS_00006898+TCONS_00006106+TCONS_00005550 +TCONS_00006107+TCONS_00005551	+
chr17	15317202	15319513	TCONS_00025573	−
chr1	149590752	149591762	TCONS_00000639	−
chr16	76613405	76692375	TCONS_l2_00009841+TCONS_l2_00010424+TCONS_ l2_00009842	+
chr19	20655812	20657239	TCONS_00026939	+
chr1	194320073	194321556	TCONS_00000363	+
chr21	44818608	44821739	TCONS_00029046	+
chr6	6901255	6918948	TCONS_00011260	+
chr5	17444119	17485937	TCONS_00009361+TCONS_00009362+TCONS_00009889	+
chr14	36738278	36742658	TCONS_00022711	−
chrY	9387051	9388485	TCONS_l2_00030891	+

chr21	38411521	38414294	TCONS_00029124	−
chr2	214032966	214039035	TCONS_00004009+TCONS_00004997	+
chr8	115315119	115323497	TCONS_00015139	−
chr16	80547387	80555350	TCONS_00024738	−
chrX	36737877	36762061	TCONS_l2_00030175+TCONS_l2_00030176	+
chr1	222645271	222652914	TCONS_l2_00001793	−
chr13	86203729	86216180	TCONS_00022079+TCONS_00022303	−
chr21	46798116	46799539	TCONS_00028960	−
chr1	193273875	193335083	TCONS_00000361+TCONS_00001231	+
chr19	43130188	43130796	TCONS_l2_00013122	−
chr8	144747990	144750647	TCONS_00014884	+
chr2	45395722	45396553	TCONS_00002885	+
chr14	45551563	45552164	TCONS_00022735	−
chr3	152874300	152879564	TCONS_00005850	−
chr2	126464387	126483468	TCONS_00004435	−
chr12	97205524	97269279	TCONS_l2_00005843+TCONS_l2_00005844+TCONS_ l2_00005845	+
chr11	4687857	4693464	TCONS_00019203+TCONS_00019202	+
chr8	127339490	127341774	TCONS_00014545	+
chr15	25361692	25367623	IPW	+
chr22	27727380	27775226	TCONS_00029567+TCONS_00029568+TCONS_00029402 +TCONS_00029785	+
chr4	180310288	180386459	TCONS_00007916	−
chr6	169362705	169364436	TCONS_00011620	−
chr4	85292546	85301342	TCONS_00007790	−
chr13	107306228	107324528	LINC00443	+
chr10	102095320	102106147	TCONS_00018032	−
chr12	54139830	54145742	TCONS_00020789+TCONS_00021315	−
chr8	12623571	12668910	LOC340357	−
chr16	84961894	84971665	TCONS_00024487	+
chr9	70843567	70844228	TCONS_00015686	+
chr2	127656113	127659673	TCONS_l2_00013962+TCONS_l2_00013961+TCONS_ l2_00013960+TCONS_l2_00013963+TCONS_ l2_00013964+TCONS_l2_00015473+TCONS_ l2_00015474+TCONS_l2_00015475+TCONS_l2_00015476	+
chr1	34830406	34833852	TCONS_00000886	+
chr5	21323945	21341484	TCONS_00009617	−
chr1	36133845	36134778	TCONS_00000887	+
chr6	107143410	107147869	TCONS_00011348+TCONS_00011912	+
chr1	56611393	56620896	TCONS_00000208	+
chr16	14823517	14827301	TCONS_l2_00010019	−
chr7	5862791	5894066	ZNF815P	+
chr10	50504157	50507063	TCONS_00017978+TCONS_00017979+TCONS_00018508	−
chr4	142489834	142497244	TCONS_00008600	−
chr11	49007280	49009104	TCONS_l2_00004998	−
chr18	56734223	56735708	TCONS_l2_00011858	+
chr22	39487087	39488860	TCONS_l2_00017771+TCONS_l2_00018262+TCONS_ l2_00017772	+
chr2	65257849	65278178	TCONS_00003712+TCONS_00003713+TCONS_00003714	+

chr2	113399407	113401757	FLJ42351	−
chr9	88358355	88401763	TCONS_l2_00028872	+
chrX	46988764	46990742	TCONS_00017420	+
chr2	747658	748673	TCONS_00005055+TCONS_00005056+TCONS_00005057 +TCONS_00005058	−
chr4	9173538	9174908	TCONS_00007978	+
chr12	76646055	76698873	TCONS_00020476+TCONS_00020477+TCONS_00021166+ TCONS_00021167+TCONS_00020478+TCONS_00020479	+
chr1	208901290	208910106	TCONS_00001257+TCONS_00000375+TCONS_00002225 +TCONS_00001258+TCONS_00002226	+
chr5	87988462	87989789	TCONS_00009720	−
chr9	98412436	98478000	TCONS_l2_00029810	+
chr1	148852393	148853717	TCONS_00000300	+
chr10	71296260	71297873	TCONS_00018944+TCONS_00018531	−
chr8	21465111	21467737	TCONS_00014647	+
chr13	112547393	112555530	TCONS_00021908+TCONS_00021910+TCONS_00022218 +TCONS_00021599+TCONS_00021600	+
chr7	64551040	64601217	TCONS_l2_00025955+TCONS_l2_00025956+TCONS_ l2_00025957+TCONS_l2_00025958+TCONS_l2_00025959	+
chr18	36174332	36308283	TCONS_00026496+TCONS_00026704	−
chr3	166287481	166288551	TCONS_00005878	−
chr8	72459748	72464753	TCONS_00014754+TCONS_00015298	+
chr4	69652271	69653039	TCONS_l2_00020560	+
chr8	101439820	101444867	TCONS_00015113+TCONS_00015114	−
chr6	11865833	11868724	TCONS_00012082	−
chrX	8196412	8423740	TCONS_00017137	+
chr10	119217607	119250420	TCONS_00018347	+
chr14	55658458	55661716	TCONS_00022513	+
chr17	8574079	8594549	TCONS_00025298	+
chr6	20321341	20333471	TCONS_00011716	+
chr1	50786946	50790716	TCONS_00000935	+
chr2	139228226	139231502	TCONS_00003010	+
chr7	77299282	77300699	TCONS_00013023	+
chr1	48549625	48607894	TCONS_l2_00000272	+
chr5	74195136	74199389	TCONS_00010379	−
chr1	17006850	17015231	TCONS_l2_00001059+TCONS_l2_00001060+TCONS_ l2_00001061	−
chr1	206214526	206223548	TCONS_00000716	−
chr5	139138175	139139292	TCONS_00010105+TCONS_00010106	+
chr20	32316737	32319634	TCONS_00028037	−
chr5	92005457	92007287	TCONS_00010406	−
chr18	54697181	54711425	TCONS_00026339+TCONS_00026627+TCONS_00026628	+
chr3	154229324	154257098	TCONS_00005853	−
chr1	56289440	56333921	TCONS_00002038+TCONS_00000206+TCONS_00000950	+
chr8	67836941	67838589	TCONS_00014745	+
chr2	5866581	5870474	TCONS_00002802	+
chr9	42493626	42497836	TCONS_l2_00029281	−
chr2	18732038	18734485	TCONS_00003589	+
chr12	89175623	89178025	TCONS_00020864+TCONS_00020865	−

chr21	47392727	47394868	TCONS_00029064+TCONS_00029252	+
chr1	56880635	56881638	TCONS_00000209+TCONS_00000955+TCONS_00002041 +TCONS_00000956	+
chr15	64996715	65010473	TCONS_00023963	+
chrX	147579633	147582135	TCONS_00017100	−
chr6	78060412	78078829	TCONS_00012215	−
chr5	27472399	27496508	LOC643401	+
chr20	6349444	6350307	TCONS_00028099	+
chr10	38064134	38073059	TCONS_00018905+TCONS_00018904+TCONS_00017954	−
chr9	132192931	132213701	TCONS_00015888+TCONS_00016489+TCONS_00015889 +TCONS_00016490	−
chr10	124578418	124586370	TCONS_l2_00003297+TCONS_l2_00003298+TCONS_ l2_00004051	+
chrX	950956	955100	TCONS_00016958	+
chr3	10809149	10813403	TCONS_00005494	+
chr1	459656	461954	TCONS_00000121	+
chr14	24344929	24347913	TCONS_00022677	−
chr10	77056141	77121436	ZNF503-AS1	+
chr7	154995524	154996899	TCONS_00013648+TCONS_00013100	+
chr10	32702426	32725020	TCONS_l2_00003470	−
chr7	51469227	51470384	TCONS_00013801	−
chr18	62539154	62660069	TCONS_00026544+TCONS_00026545+TCONS_00026724 +TCONS_00026546	−
chr4	10409917	10411772	TCONS_00007980	+
chr15	43995753	43997041	TCONS_l2_00009063	−
chr1	143356184	143357032	TCONS_00001660	−
chr6	114844608	114866483	TCONS_00012261+TCONS_00011548	−
chr10	6319650	6377937	LOC399715	+
chr1	33603694	33606476	TCONS_00001450	−
chr22	48409497	48411394	TCONS_00029621	+
chr11	72239231	72246063	TCONS_00019699	−
chr7	77144084	77163609	TCONS_00014341+TCONS_00013867	−
chr4	149539477	149540339	TCONS_00008615	−
chr2	111160496	111181393	LOC440895	−
chr12	70290958	70297801	TCONS_l2_00005745	+
chr20	48642152	48647008	TCONS_00028433+TCONS_00028434	−
chr7	141112379	141116190	TCONS_00013266+TCONS_00013267	−
chr4	45194851	45197494	TCONS_00008064	+
chr12	39481529	39486291	TCONS_00020759	−
chr19	10762538	10764548	ILF3-AS1	−
chr9	2541097	2541635	TCONS_00015640	+
chr6	57690895	57691645	TCONS_00011318	+
chr9	137159370	137160655	TCONS_00016207	+
chr9	69893984	69901473	TCONS_l2_00029383	−
chr7	22602956	22613617	LOC100506178	+
chr13	20856669	20857338	TCONS_00021711	+
chr2	181940778	181970695	TCONS_00003460	−
chr15	84295107	84302519	TCONS_00023491	+

chr1	161607438	161608218	TCONS_00000653	−
chr12	93569675	93575919	TCONS_00020523	+
chr9	110329107	110338504	TCONS_00016667+TCONS_00016123	+
chr16	73846493	73849940	TCONS_00024465	+
chr8	8271720	8282139	TCONS_l2_00028090	−
chr2	96379286	96384234	TCONS_00004332	−
chr17	20824338	20833046	TCONS_00025161	+
chr2	57541338	57542640	TCONS_00004261	−
chr7	12536055	12585630	TCONS_l2_00027052+TCONS_l2_00025670+TCONS_ l2_00027053+TCONS_l2_00027054+TCONS_ l2_00025671+TCONS_l2_00025672	+
chr21	15225712	15236072	TCONS_00028968	+
chr8	22402216	22408678	TCONS_00015250+TCONS_00015251+TCONS_00015252	+
chr2	19675211	19719465	TCONS_00002842+TCONS_00004705+TCONS_00003591	+
chr20	23168344	23169661	TCONS_00028641	−
chr5	2069055	2069531	TCONS_00009836	+
chr1	26373156	26375590	TCONS_00001962	+
chr10	131904273	131907101	CTAGE7P	+
chr21	37377636	37379899	TCONS_00028841	+
chr1	76175700	76189696	TCONS_00000549	−
chr20	55733858	55737430	TCONS_00028451	−
chr21	24775997	24786569	TCONS_l2_00017174	−
chr6	19807811	19809526	TCONS_00011715	+
chr8	48909213	48911333	TCONS_l2_00028191	−
chr6	71104590	71109120	TCONS_00011322+TCONS_00012512+TCONS_00011854 +TCONS_00012513	+
chr17	37706690	37723895	TCONS_00025374+TCONS_00025375+TCONS_00025376 +TCONS_00025377+TCONS_00025378	+
chr15	78439743	78441489	TCONS_00023745	−
chr10	132608003	132609030	TCONS_00018646	−
chr19	46684560	46699678	TCONS_00027056	+
chr18	3466271	3478974	TCONS_00026238+TCONS_00026237+TCONS_00026239 +TCONS_00026187	+
chr8	37182936	37189507	TCONS_00014970+TCONS_00014971+TCONS_00014972 +TCONS_00015425	−
chr7	63831907	63849519	TCONS_00014150+TCONS_00013475+TCONS_00014151 +TCONS_00014152	+
chr1	212603322	212606095	TCONS_00001806+TCONS_00002579	−
chr1	95816807	95817810	TCONS_00000584	−
chrX	142840832	142911228	TCONS_l2_00030756	+
chr10	91406046	91410579	TCONS_00018019	−
chr7	5653430	5656062	TCONS_00013686	−
chr1	204001575	204010392	LINC00303	−
chr20	4301207	4304982	TCONS_00028298	−
chr20	36120874	36137759	TCONS_00028042+TCONS_00028043+TCONS_00028662	−
chr4	38194961	38208228	TCONS_00008464	−
chr22	31365634	31375380	TUG1	+

chr2	30555416	30583316	TCONS_00004738+TCONS_00004739+TCONS_00002863+TCONS_00002691+TCONS_00004740+TCONS_00003621+TCONS_00003622	+
chr19	8018416	8020786	TCONS_00026883	+
chr15	69884468	69905229	TCONS_00023235	+
chr19	29374427	29388915	TCONS_00026974+TCONS_00026975	+
chr1	175846479	175849604	TCONS_00000338	+
chr20	10733926	10745934	TCONS_00028002	−
chr6	17332461	17359544	TCONS_00011710	+
chr13	24043651	24061603	LINC00327	+
chr2	110744557	110752858	LOC440894	+
chrX	152876149	152878380	TCONS_00017027	+
chr14	34561378	34697119	TCONS_00022701+TCONS_00022702	−
chr7	156696055	156714884	TCONS_00013653	+
chrX	20329961	20336200	TCONS_00017300	−
chr3	189846778	189847483	TCONS_00006346	+
chr18	47967292	47967952	TCONS_00026521	−
chr20	19029907	19031200	TCONS_00028118	+
chr3	120005001	120008319	TCONS_00006177	+
chr17	35289409	35294022	TCONS_00025137+TCONS_00026057	−
chr2	91909643	91911547	TCONS_l2_00014690	−
chr5	42318256	42374527	TCONS_00010318	−
chr5	72236631	72251181	TCONS_00010375	−
chr8	102137266	102138373	TCONS_00015115	−
chr20	61265033	61266856	TCONS_00028083	−
chr10	48979932	48981202	TCONS_l2_00003554	−
chr8	66073380	66092575	LINC00251	−
chr2	8147901	8468549	LINC00299	−
chr1	852953	854817	LOC100130417	−
chr18	41903400	42111662	TCONS_00026205+TCONS_00026706+TCONS_00026707	−
chr7	17719173	17720283	TCONS_00012959	+
chr3	184096018	184097565	TCONS_00007321	−
chr16	86052353	86061287	TCONS_00024490	+
chr18	14918961	14931880	TCONS_00026292	+
chr1	116706905	116708862	TCONS_00000282	+
chr1	111794402	111795135	TCONS_l2_00000487	+
chr3	133774100	133776492	TCONS_00005830	−
chr3	197185276	197193976	TCONS_00005721	+
chr17	62962668	62971703	AMZ2P1	−
chr20	59654098	59659077	TCONS_00028251+TCONS_00028252+TCONS_00027979+TCONS_00028580	+
chr1	160864697	160866290	TCONS_l2_00000663	+
chr11	1799971	1825792	TCONS_l2_00004338	+
chr2	130352023	130354693	TCONS_00003871	+
chr4	38278024	38281503	TCONS_00008465	−
chr3	4521579	4534847	TCONS_00006399+TCONS_00005727	−
chr2	26894122	26897332	TCONS_00003237	−

chr10	37524718	37591595	TCONS_l2_00002970+TCONS_l2_00003937+TCONS_ l2_00002971+TCONS_l2_00002972+TCONS_l2_00002973	+
chr4	77829330	77831274	TCONS_00009076	−
chr9	70707658	70711592	TCONS_l2_00028819	+
chr11	130949313	130953304	TCONS_00019807	−
chr15	21932514	21940739	LOC646214	−
chr4	178649911	178911904	LOC285501	+
chr1	59613313	59615760	TCONS_00002046	+
chr9	24545400	24592305	TCONS_00015933+TCONS_00016544+TCONS_00016545 +TCONS_00015934+TCONS_00016546	+
chr2	121935903	121940156	TCONS_00002991	+
chr2	217852693	217861287	TCONS_00004025	+
chr2	166837227	166841278	TCONS_00003928	+
chr16	75793927	75795749	TCONS_00024468	+
chr17	20615759	20641178	LOC100287072	+
chr17	31205449	31233602	TCONS_00025892+TCONS_00025166	+
chr13	106359218	106414143	LINC00343	+
chr3	193918479	193921896	TCONS_00006357	+
chr7	84161792	84213638	TCONS_00012885+TCONS_00013033	+
chr1	160446	161525	TCONS_00000119	+
chr12	106136548	106149079	TCONS_00020905	−
chr18	57792170	57796757	TCONS_00026346	+
chr11	67544666	67550430	TCONS_00019149	+
chr13	88507102	88525012	TCONS_00021868	+
chr8	6844700	6866346	DEFT1P	−
chr13	25297237	25310786	TCONS_00021941	−
chr16	58456606	58477085	TCONS_00024424	+
chr6	150260453	150262631	TCONS_00011985+TCONS_00012617	+
chr6	29675362	29688930	TCONS_l2_00024693+TCONS_l2_00024694+TCONS_ l2_00024695+TCONS_l2_00024696	−
chr1	156358018	156365401	TCONS_00000317	+
chr1	54377676	54378843	TCONS_00001498	−
chr9	70958614	70966054	TCONS_00016340	−
chr8	76851942	76864003	TCONS_00014766	+
chr3	196326511	196327216	TCONS_00006387	+
chr13	62578226	62603681	TCONS_00021662+TCONS_00022036	−
chr11	121740077	121760026	TCONS_00019506	+
chr1	149672997	149677287	TCONS_l2_00002208+TCONS_l2_00000599	+
chr13	84570331	84572249	TCONS_00021864	+
chr13	19968875	19982245	TCONS_l2_00006705+TCONS_l2_00006706	+
chr9	139506486	139519693	TCONS_00016513+TCONS_00016903+TCONS_00016514 +TCONS_00016515	−
chr13	90208156	90214259	TCONS_l2_00006966	+
chr12	40789712	40834900	TCONS_00021300+TCONS_00021301+TCONS_00020762	−
chr10	2541913	2542762	TCONS_00017759	+
chr4	156902136	156938403	TCONS_l2_00020844+TCONS_l2_00020845	+
chr5	72066458	72067544	TCONS_00010374	−
chr16	27279526	27301789	FLJ21408	+

chr7	81205702	81320722	TCONS_l2_00027424+TCONS_l2_00027425	−
chr20	24278221	24298722	TCONS_00028027	−
chr12	4809607	4813248	TCONS_00020685	−
chr3	150608647	150610167	TCONS_00006273	+
chr6	143267184	143277835	TCONS_00011579	−
chr5	61003088	61029193	TCONS_l2_00022337	+
chr6	15695349	15758477	TCONS_00012090+TCONS_00012660+TCONS_00012091 +TCONS_00012661+TCONS_00012662	−
chr4	127484733	127485437	TCONS_l2_00020754	+
chr6	158703295	158733390	TCONS_00011401	+
chr10	85926986	85931832	TCONS_00017740	−
chrX	114957297	114959383	TCONS_00017006+TCONS_00017007	+
chr1	211886532	211887659	TCONS_00001801	−
chr5	71982939	72058747	TCONS_l2_00023427+TCONS_l2_00023428+TCONS_ l2_00022362+TCONS_l2_00023429+TCONS_ l2_00022363+TCONS_l2_00022364	+
chrX	2484070	2527190	TCONS_00017281+TCONS_00017282	−
chr20	58090246	58091094	TCONS_00027969	+
chr9	106842305	106854755	TCONS_00015859+TCONS_00016451+TCONS_00016850	−
chr10	120763048	120763396	TCONS_00018058	−
chrX	153562310	153563387	TCONS_00017107	−
chr21	44373890	44376488	TCONS_00028850	+
chr7	134030823	134035543	TCONS_00013264	−
chr18	10407027	10410448	TCONS_00026418+TCONS_00026671	−
chr20	52416362	52417857	TCONS_00028068	−
chr12	130518357	130526887	LOC100190940	−
chr3	54048257	54065456	TCONS_00006514	−
chrX	134252882	134254405	LINC00633	−
chr4	15852163	15857611	TCONS_00008443	−
chr22	48844275	48864492	TCONS_00029839+TCONS_00029623+TCONS_00029440 +TCONS_00029840	+
chr5	142869420	142910915	TCONS_00009795	−
chr19	57352270	57359922	MIMT1	+
chr7	76668797	76682355	LOC100132832	+
chr10	19219126	19219643	TCONS_00018132	+
chr13	75321859	75325492	TCONS_00021835	+
chr1	161732646	161736000	TCONS_00000655+TCONS_00000656	−
chr12	126578653	126585339	TCONS_00020623+TCONS_00021243+TCONS_00021244 +TCONS_00020625	+
chrX	70994970	71004546	TCONS_00017068+TCONS_00017335	−
chr4	52910971	52913124	TCONS_00007527+TCONS_00008791+TCONS_00008072	+
chr11	49426360	49438324	TCONS_l2_00005001+TCONS_l2_00005002	−
chr14	103039845	103041292	TCONS_00022887	−
chr7	92074774	92076644	TCONS_00013529	+
chr1	71181616	71185972	TCONS_00000546	−
chrY	9385994	9386866	TCONS_l2_00030890	+
chr2	25956629	25959457	TCONS_00005117	−

chr16	88210942	88220609	TCONS_00024507+TCONS_00024904+TCONS_00024905+TCONS_00024508+TCONS_00024509+TCONS_00024510	+
chr3	125518978	125519991	TCONS_l2_00019483	−
chr9	69651361	69664949	LOC100133920	+
chr18	73936399	73963287	TCONS_00026572+TCONS_00026740	−
chr6	77484704	77698599	TCONS_00012521+TCONS_00011863+TCONS_00011864	+
chr12	131941578	131943433	TCONS_00020658	+
chr2	172967734	172974710	TCONS_00003049	+
chr3	81295730	81346496	TCONS_00005792+TCONS_00005793	−
chr11	58853837	58861440	TCONS_l2_00004527	+
chr10	48577521	48593703	TCONS_00018504	−
chrX	135025017	135026977	TCONS_l2_00030612	−
chr18	11447608	11490670	TCONS_00026423+TCONS_00026675+TCONS_00026676	−
chr3	194565038	194567506	TCONS_00006757	−
chr1	222763365	222765741	TCONS_00001831	−
chr14	101908423	101910649	TCONS_00022636	+
chr15	39047157	39049710	TCONS_00023634+TCONS_00024073	−
chr21	39695557	39705343	TCONS_00028924+TCONS_00029125+TCONS_00028925	−
chr15	89045175	89049018	TCONS_00023283	−
chr17	79995797	79997235	TCONS_00025996	+
chr2	98069548	98071970	TCONS_00003334	−
chr6	45523580	45545334	TCONS_00012731+TCONS_00012732+TCONS_00012737+TCONS_00012736+TCONS_00012735+TCONS_00012734+TCONS_00012733+TCONS_00012182+TCONS_00012183+TCONS_00011496+TCONS_00012739	−
chr2	23580387	23583247	TCONS_00003234	−
chr16	2916362	2918212	TCONS_00024545+TCONS_00024546	−
chr18	71336695	71358564	TCONS_00026560+TCONS_00026561+TCONS_00026736+TCONS_00026737	−
chr4	43459544	43494560	TCONS_00007756+TCONS_00009042	−
chr3	69002584	69018827	TCONS_00006085	+
chr2	54937397	54938672	TCONS_00003683	+
chr6	149882288	149885085	TCONS_00011398	+
chr1	31933168	31944351	TCONS_00001448	−
chr5	157912250	157922781	TCONS_00010149	+
chr1	147209596	147210939	TCONS_00001668	−
chr3	70666965	70669429	TCONS_00006087+TCONS_00006884	+
chr5	124298627	124317909	TCONS_00010078+TCONS_00010079	+
chr14	101819694	101822512	TCONS_00022880	−
chr11	127140891	127206921	TCONS_00019530+TCONS_00019115	+
chr8	103541045	103541931	TCONS_00014814	+
chr17	68896839	68916705	TCONS_00025479	+
chr12	69853359	69854790	TCONS_00020466	+
chr19	29709834	29710603	TCONS_00026980	+
chr13	49144775	49147453	TCONS_00021650	−
chr20	24045120	24047430	TCONS_00028138	+
chr2	114576628	114578362	TCONS_00004409	−
chr4	128540065	128545185	TCONS_00008575	−

chr2	181966659	182264280	TCONS_00003060+TCONS_00003061+TCONS_00002726+TCONS_00003062+TCONS_00003063+TCONS_00003960	+
chr11	106450235	106475095	TCONS_00019749	−
chr1	4615911	4616444	TCONS_00001381	−
chr4	181826709	181838975	TCONS_00008671	−
chr15	99637426	99641439	TCONS_00023547	+
chr16	60852523	60855071	TCONS_00024693	−
chr1	86966982	86969353	TCONS_00001555	−
chr4	8160852	8166042	TCONS_00008419	−
chr13	45620725	45657260	TCONS_00021545+TCONS_00022170	+
chr16	75259972	75262093	TCONS_l2_00010423	+
chr21	29420733	29509928	TCONS_00028766+TCONS_00028827+TCONS_00029205+TCONS_00029206+TCONS_00028828+TCONS_00028829+TCONS_00028984	+
chr5	79904442	79918343	TCONS_00009713	−
chr8	58658708	58662754	TCONS_00014492	+
chr3	155008021	155011489	LOC100507537	−
chr14	101936417	101937343	TCONS_00022882	−
chr13	39141861	39153653	LINC00366	+
chr11	69911595	69920017	TCONS_00019372+TCONS_00019942	+
chr4	39540664	39545555	TCONS_00009040	−
chr6	85130746	85265870	TCONS_l2_00025430+TCONS_l2_00025431+TCONS_l2_00024833+TCONS_l2_00025432	−
chr20	1675851	1678576	TCONS_l2_00016426	−
chr1	239413979	239416637	TCONS_00001891	−
chr8	143276214	143277877	TCONS_00015185	−
chr2	217398740	217443933	TCONS_00004019+TCONS_00004020	+
chr3	125962461	125964427	TCONS_00006631	−
chr19	23505740	23507220	TCONS_00027716	−
chrX	118940398	118966262	TCONS_l2_00030586+TCONS_l2_00030587	−
chr7	77038	80886	TCONS_00012931+TCONS_00013285	+
chr16	51096098	51129392	TCONS_00024643	−
chr4	17460264	17462011	TCONS_00008445	−
chr7	86954664	86974808	TP53TG1	−
chr4	66864564	67015730	TCONS_l2_00021607+TCONS_00008096+TCONS_00007547	+
chr2	111133765	111142102	LOC440894	−
chrY	2477306	2525270	CD99P1	+
chr20	58656497	58662619	TCONS_00028716	−
chr1	163229640	163230449	TCONS_00000327	+
chr3	170046226	170072227	TCONS_00006308+TCONS_00005656	+
chr5	105539003	105541178	TCONS_00010434	−
chr6	16150994	16153073	TCONS_00011706	+
chr4	14384747	14410702	TCONS_00007719	−
chr19	55728028	55732984	TCONS_00026835	+
chr1	9561151	9563530	TCONS_00000469	−
chrX	45707327	45710946	TCONS_l2_00030808+TCONS_l2_00030809+TCONS_l2_00030477	−

chr19	23487809	23493504	TCONS_00027241+TCONS_00027243+TCONS_00027712+TCONS_00027713+TCONS_00027715+TCONS_00027244+TCONS_00027245+TCONS_00027246	−
chr8	1983386	1983812	TCONS_00014579	+
chrX	27177022	27417114	TCONS_l2_00030797+TCONS_l2_00030466+TCONS_l2_00030798	−
chr2	172629992	172632354	TCONS_l2_00015537	+
chr21	25333220	25339588	TCONS_00028889	−
chr2	101802575	101807916	TCONS_00004353	−
chr21	34430135	34437289	TCONS_00028834	+
chr9	68726541	68748372	LOC100132352	+
chr11	60943361	60944048	TCONS_00019311	+
chr14	84667417	84688047	TCONS_00022820	−
chr1	88928658	88931553	TCONS_00001015+TCONS_00001016	+
chr15	96533823	96795377	TCONS_00023538+TCONS_00023539	+
chr2	196397759	196403405	TCONS_00003073	+
chr14	55695908	55696519	TCONS_00023109	−
chr17	13523046	13530958	TCONS_00025843+TCONS_00025310+TCONS_00025842	+
chr12	67829112	67829455	TCONS_00020823	−
chr3	39821027	39824881	TCONS_l2_00020106	−
chr6	2917128	2941664	TCONS_00011253+TCONS_00011647	+
chr1	237070344	237091621	TCONS_00002269+TCONS_00001335+TCONS_00001336+TCONS_00001337	+
chr13	48399465	48481492	TCONS_l2_00007538+TCONS_l2_00007175+TCONS_00022001+TCONS_l2_00007539	−
chr3	118207645	118215620	TCONS_00006934+TCONS_00006168+TCONS_00006935+TCONS_00006936	+
chr1	234845005	234855967	TCONS_00000424+TCONS_00000070+TCONS_00001332	+
chr6	14005151	14007132	TCONS_00011269	+
chr16	81772679	81774183	TCONS_00024256	+
chr7	115430301	115431383	TCONS_00013570	+
chr18	37421139	37456359	TCONS_00026204	−
chr7	124638324	124641124	TCONS_00013576	+
chr19	58521909	58537013	TCONS_l2_00013262	−
chr18	22208146	22242162	LOC729950	+
chr18	11487834	11506982	TCONS_00026599+TCONS_00026278+TCONS_00026279+TCONS_00026600+TCONS_00026280+TCONS_00026281+TCONS_00026282+TCONS_00026601	+
chr7	151106322	151110132	WDR86-AS1	+
chr15	40609595	40615570	TCONS_00023640	−
chr7	150102840	150109558	LOC728743	+
chr1	86093302	86094050	TCONS_00001012	+
chr7	1609709	1629261	PSMG3-AS1	+
chr15	20433049	20448145	TCONS_l2_00008906+TCONS_l2_00008907	−
chr5	108833578	108834809	TCONS_00009478	+
chr4	143772645	143778965	TCONS_00008273	+
chr9	66333367	66346175	TCONS_l2_00028759	+
chr1	246933184	246945218	TCONS_l2_00001903+TCONS_l2_00001904+TCONS_l2_00001905+TCONS_l2_00001906	−

chr10	85432162	85435195	TCONS_00018010	−
chr2	37971476	37974189	TCONS_00002873	+
chr10	26213377	26222639	TCONS_00017939	−
chr18	42235955	42238832	TCONS_00026326	+
chr6	122238492	122283567	TCONS_00011942+TCONS_00012593+TCONS_00011943	+
chr1	21912965	21917680	TCONS_00000166	+
chr8	6825663	6826635	DEFA10P	−
chr20	47032443	47035369	TCONS_00028425	−
chr3	78316090	78343881	TCONS_00005546+TCONS_00005547+TCONS_00005548	+
chr1	18328349	18335092	TCONS_00001408	−
chr19	20959100	20983629	TCONS_l2_00012388+TCONS_l2_00012389+TCONS_ l2_00013303+TCONS_l2_00012390	+
chr9	96192624	96193581	TCONS_00015849	−
chr1	212731196	212734736	TCONS_00001266+TCONS_00001267+TCONS_00001268	+
chr5	111964133	111967797	TCONS_00009482+TCONS_00009483+TCONS_00009484	+
chr17	56066403	56072211	TCONS_00025146+TCONS_00025147+TCONS_00025707 +TCONS_00025708+TCONS_00025709	−
chr1	28567780	28573670	TCONS_00001440+TCONS_00001441	−
chr19	23003240	23004064	TCONS_00026959	+
chr2	23597678	23604170	TCONS_00005115+TCONS_00003235+TCONS_00003236	−
chr11	22501632	22513577	TCONS_00019258	+
chr16	51421606	51559447	TCONS_00024645+TCONS_00024646+TCONS_00024647 +TCONS_00024648	−
chr10	92793811	92796960	TCONS_00018573	−
chrX	57148095	57154235	TCONS_00016988+TCONS_00017182	+
chr5	60617326	60627805	TCONS_00010351+TCONS_00010352	−
chr2	121311319	121315685	TCONS_00003391	−
chr10	26866505	26868309	TCONS_00018454+TCONS_00018455+TCONS_00017941 +TCONS_00017940	−
chr1	181205524	181207740	GM140	+
chr18	73834953	73857210	LOC339298	+
chr7	106593673	106635509	TCONS_00013559	+
chr1	29196995	29203848	TCONS_00000493+TCONS_00001444	−
chr19	47929263	47930655	TCONS_00027064	+
chr8	1376463	1378077	TCONS_00014902	−
chr9	69269462	69272421	TCONS_00016337	−
chr2	45396702	45402931	TCONS_00003663+TCONS_00004770	+
chr12	63683874	63755075	TCONS_00020820	−
chr22	28070072	28073554	TCONS_00029569	+
chr7	130600639	130624493	TCONS_00013597+TCONS_00013598+TCONS_00013599 +TCONS_00013600	+
chr19	54107291	54110228	TCONS_l2_00013223	−
chr10	736184	737679	TCONS_00018077	+
chr19	23687934	23695476	TCONS_l2_00012964	−
chr2	112022860	112044842	TCONS_00002976+TCONS_00003819	+
chr1	60539554	60542979	TCONS_00000964	+
chr5	176153943	176166459	TCONS_00009816	−
chr15	93325223	93332339	TCONS_00023791+TCONS_00023792+TCONS_00023793	−

chrX	105708482	105711579	TCONS_l2_00030573	−
chr21	33393279	33394095	TCONS_00028914	−
chr16	53412402	53423133	TCONS_00024420	+
chr2	22759262	22762702	TCONS_00004716+TCONS_00002853	+
chr2	157890362	157906713	TCONS_00003914+TCONS_00003915	+
chr4	25866410	25871707	TCONS_00008452+TCONS_00007729+TCONS_00007730	−
chr18	76399459	76401023	TCONS_00026381	+
chr2	107871147	107982329	TCONS_00003360+TCONS_00003361	−
chr10	7176323	7186792	TCONS_00018117	+
chr13	25691351	25693540	TCONS_00021734+TCONS_00022147	+
chr7	50518216	50521151	TCONS_00013800+TCONS_00013170	−
chr15	62466084	62478265	TCONS_00023690	−
chr7	3233737	3234428	TCONS_00013678	−
chr3	183613837	183622086	TCONS_l2_00020286	−
chr4	129504154	129522562	TCONS_00007825+TCONS_00007826	−
chr1	43585819	43611958	TCONS_00000039+TCONS_00000921	+
chr13	63757234	63801189	TCONS_00021665	−
chr11	61215891	61218493	TCONS_00020069	−
chr20	18774693	18776709	LOC100270804	+
chr14	101872355	101874412	TCONS_00022881	−
chr2	119357361	119360542	TCONS_00003389	−
chr3	154687072	154688012	TCONS_00005855	−
chr10	45731338	45738615	TCONS_l2_00003529	−
chr13	80140881	80144983	TCONS_00021675	−
chr6	106525744	106527926	TCONS_00011909	+
chr13	114446312	114448413	TCONS_00022320	−
chr10	86331899	86335575	TCONS_00018256	+
chr18	36046480	36052660	TCONS_00026493	−
chr3	129985909	129995570	TCONS_00006656+TCONS_00006657+TCONS_00007265 +TCONS_00006658	−
chr3	80810191	80838114	TCONS_00006546+TCONS_00005790+TCONS_00006547	−
chr12	89968491	89972126	TCONS_00020509	+
chr20	22370851	22371509	TCONS_00028125	+
chr15	48284932	48341686	TCONS_00023390	+
chr11	125794238	125795994	TCONS_00020151	−
chr11	41830600	41871849	TCONS_00019279	+
chr1	31297075	31307282	TCONS_l2_00002019+TCONS_l2_00002020+TCONS_ l2_00000198	+
chr2	895985	897649	TCONS_00005059	−
chr17	20640243	20648591	TCONS_l2_00011144	−
chr1	2481359	2484284	LOC115110	−
chr4	120852844	120885655	TCONS_l2_00020729+TCONS_l2_00020730	+
chr21	9825744	9826389	TCONS_00029193	+
chr8	49998879	50013639	TCONS_00014694	+
chr2	242626503	242633704	TCONS_l2_00014330+TCONS_l2_00014331+TCONS_ l2_00014333+TCONS_l2_00015631	+
chr5	53955988	53999188	TCONS_00010334	−
chr11	33025139	33037285	TCONS_00019608	−

chr8	65309156	65324649	TCONS_00015025	−
chr9	94246565	94249578	TCONS_00016406	−
chr5	172233452	172234199	TCONS_00010556	−
chr7	135344960	135345928	TCONS_l2_00026230	+
chr8	96219235	96228602	C8orf69	−
chr10	43366969	43369508	TCONS_00017817	+
chr3	153252332	153697975	TCONS_00005851+TCONS_00005852	−
chr4	90459367	90472707	TCONS_00008529+TCONS_00009085	−
chr9	46687557	46746820	KGFLP1	+
chr13	88261060	88262077	TCONS_00022208	+
chr6	19707697	19749995	TCONS_00012103+TCONS_00012676+TCONS_00012104 +TCONS_00011460+TCONS_00012105+TCONS_00011461	−
chr2	108683755	108684693	TCONS_00004371	−
chr5	67802062	67807619	TCONS_00009987	+
chr3	122998230	122999473	TCONS_00006187+TCONS_00006188	+
chr6	113038590	113058872	TCONS_00011923	+
chr15	21365491	21366478	TCONS_00023293	+
chr7	106065909	106151990	TCONS_00013557	+
chr11	129636811	129684687	TCONS_00019802+TCONS_00019803	−
chr16	32181954	32199434	TCONS_l2_00010108+TCONS_l2_00010457	−
chr3	30553387	30567941	TCONS_00006465+TCONS_00005754	−
chr16	19365544	19405174	TCONS_00024596+TCONS_00024975+TCONS_00024597	−
chr7	25878711	25888418	TCONS_00013381	+
chr7	56216915	56246839	TCONS_l2_00025884	+
chr2	96627469	96658969	TCONS_l2_00014714+TCONS_l2_00014715	−
chr2	66892907	66957289	TCONS_00003719+TCONS_00002918+TCONS_00002919 +TCONS_00002920+TCONS_00002921	+
chr8	86445765	86446811	TCONS_00014790	+
chr7	62854166	62882424	TCONS_l2_00027124+TCONS_00013017+TCONS_00014 146	+
chr8	117441845	117460587	TCONS_00015140	−
chr20	47012066	47013638	TCONS_00028059	−
chr1	40623424	40626915	TCONS_00001468	−
chr16	68642997	68645496	TCONS_00024444	+
chr5	65643590	65736315	TCONS_00010941+TCONS_00010940+TCONS_00010942	−
chr10	73638114	73638865	TCONS_00017841	+
chr5	136070616	136090375	TCONS_00009524	+
chr2	12675862	12805597	TCONS_00004148	−
chr22	35846034	35851349	TCONS_00029585+TCONS_00029795	+
chr3	166896245	166901168	TCONS_00006706	−
chr9	7344597	7345605	TCONS_00016745	−
chr13	104975778	105020809	TCONS_00021887	+
chr10	27546619	27548374	TCONS_00018458	−
chr2	123643945	123644599	TCONS_00003847	+
chr9	11097567	11109984	TCONS_00016268	−
chr17	74803171	74819136	TCONS_00025498	+
chr5	4135795	4143761	TCONS_00009332	+
chr11	122178110	122179274	TCONS_00019509	+

chr1	54447917	54452234	TCONS_00000200	+
chr5	7372699	7391998	TCONS_00009864+TCONS_00009344+TCONS_00009865	+
chr17	36202573	36244363	LOC284100	−
chr1	149615886	149616885	TCONS_00002157+TCONS_00002158+TCONS_00001143	+
chr5	81190738	81196638	TCONS_00010006	+
chr16	15013098	15014767	TCONS_l2_00009604+TCONS_l2_00009605	+
chr4	171663620	171664869	TCONS_00007895	−
chr19	23031069	23044102	TCONS_l2_00012412	+
chr9	132323020	132326647	TCONS_00016492	−
chr5	173664897	173670279	TCONS_00010182	+
chr5	524820	526709	TCONS_00009322	+
chr16	63100447	63651969	TCONS_l2_00010220+TCONS_l2_00010221	−
chr17	67703268	67703659	TCONS_l2_00011437	−
chr17	47995	49628	TCONS_00025258	+
chr4	154561438	154601686	TCONS_l2_00020823	+
chr11	87070956	87073696	TCONS_00019424	+
chr13	32011875	32017603	TCONS_00021747	+
chr9	39722426	39817486	TCONS_l2_00029273+TCONS_l2_00029274	−
chr10	5606767	5608680	TCONS_00018421	−
chr5	158848340	158871718	TCONS_00010154	+
chr10	130757036	130758326	TCONS_00018068	−
chr2	554475	559467	TCONS_00004094	−
chr5	128679236	128729628	TCONS_00010086	+
chr13	110784346	110785661	TCONS_00022114	−
chr6	101410581	101411032	TCONS_00011906	+
chr2	33050510	33162270	LINC00486	+
chr20	51068003	51072158	TCONS_00028065	−
chr2	1624282	1629191	TCONS_00003150	−
chr13	73987610	74222390	TCONS_00021669+TCONS_00021670	−
chr15	77991686	77992117	TCONS_00023984	+
chr4	149614151	149621313	TCONS_00008286+TCONS_00007654	+
chr15	70264901	70316821	TCONS_00023723+TCONS_00023724	−
chr5	180618046	180618852	TCONS_00009824	−
chr1	25579798	25594376	TCONS_00000490+TCONS_00002308+TCONS_00001430 +TCONS_00001431	−
chr19	58486502	58488209	TCONS_00027142	+
chr3	128182437	128191160	DNAJB8-AS1	+
chr6	112194867	112197726	TCONS_00011921	+
chr13	33383328	33485790	LINC00423	−
chr1	175873898	175889649	TCONS_00000691	−
chr1	211849016	211863445	TCONS_00001260+TCONS_00000381+TCONS_00000382 +TCONS_00001261+TCONS_00000383	+
chr2	114737146	114764887	LOC440900	+
chr17	7607077	7608261	TCONS_00025553	−
chr6	78190744	78213653	TCONS_l2_00024298	+
chr15	101273239	101274044	TCONS_00023563	+
chr5	38783604	38793270	TCONS_00009926+TCONS_00009387	+
chr10	8137923	8139176	TCONS_00018121	+

chr20	47126391	47133716	TCONS_00028205+TCONS_00028207+TCONS_00028208	+
chr1	439365	453948	TCONS_l2_00001929+TCONS_l2_00001930+TCONS_l2_00001931	+
chr1	20569588	20571148	TCONS_00000163	+
chr3	129816625	129830276	FAM86HP	−
chr3	154544805	154578806	TCONS_00005854	−
chr16	62713184	62714028	TCONS_00024431	+
chr12	127354040	127359236	LOC440117	−
chr7	132333553	132413528	FLJ40288	+
chr10	3052723	3055303	TCONS_00017763	+
chr21	34563457	34563753	TCONS_l2_00017203	−
chr15	72099421	72101842	TCONS_00023454	+
chr4	133757927	133909832	TCONS_00007833+TCONS_00007834	−
chr12	103344390	103345223	TCONS_00020541	+
chr7	63201459	63227867	TCONS_l2_00026610+TCONS_l2_00026611	−
chr17	75663172	75663808	TCONS_00025508	+
chr18	25512853	25513932	TCONS_00026697+TCONS_00026698	−
chr17	67586136	67624492	TCONS_00025126+TCONS_00025192	+
chr9	125165686	125211256	TCONS_00015746	+
chr3	197360474	197372891	TCONS_l2_00019116+TCONS_l2_00020052	+
chr20	59205912	59228954	TCONS_l2_00016820	+
chr19	56578050	56590164	TCONS_00026836	+
chr6	83122462	83125545	TCONS_00011878	+
chr2	16408295	16409462	TCONS_00003215	−
chr11	64187863	64188528	TCONS_00019097	+
chr2	222597441	222601602	TCONS_00004038	+
chr6	18744007	18757698	TCONS_00012100	−
chr9	1298277	1328584	TCONS_00015905+TCONS_00016529+TCONS_00016528+TCONS_00016527+TCONS_00015904+TCONS_00015906	+
chr16	75535386	75550207	TCONS_l2_00010283+TCONS_l2_00010284+TCONS_l2_00010285+TCONS_l2_00010286	−
chr18	44812095	45120916	TCONS_00026192	+
chr16	899232	900609	TCONS_00024310	+
chr9	71155952	71258607	TCONS_l2_00029772	+
chr1	31984036	31989846	LOC284551	+
chr13	81853964	81876842	TCONS_00021862+TCONS_00021578	+
chr3	102444625	102446410	TCONS_00006137	+
chr9	87234248	87271496	TCONS_00016036	+
chr2	221605024	221605403	TCONS_00004035	+
chr3	187166633	187167238	TCONS_00005686	+
chr8	62671660	62678528	TCONS_00014518	−
chr16	75614470	75622358	TCONS_00024730	−
chr10	124516210	124557161	FLJ46361	+
chr4	129575030	129581852	TCONS_00008252	+
chr3	43820453	43821474	TCONS_00005768	−
chr5	8457800	8463209	LOC100505738	+
chr15	52977218	52982263	TCONS_00023409+TCONS_00023942+TCONS_00023410	+

chr11	121760051	121777195	TCONS_00019507+TCONS_00019508	+
chr16	76262321	76269536	TCONS_l2_00010290+TCONS_l2_00010291	−
chr3	28179941	28182228	TCONS_00006461	−
chr9	76538935	76593537	TCONS_00016621+TCONS_00016015+TCONS_00016016	+
chr3	125546116	125634748	TCONS_l2_00019484+TCONS_l2_00019485+TCONS_l2_00019486+TCONS_l2_00020179+TCONS_l2_00019487+TCONS_l2_00019488+TCONS_l2_00019489+TCONS_l2_00019490+TCONS_l2_00019491+TCONS_l2_00019492+TCONS_l2_00020180+TCONS_l2_00019493+TCONS_l2_00019494	−
chr12	53547053	53548417	TCONS_l2_00005685+TCONS_l2_00005686+TCONS_l2_00005687	+
chr2	67789199	67831420	TCONS_00002929+TCONS_00003726+TCONS_00004809+TCONS_00004810+TCONS_00004811+TCONS_00003727	+
chr20	55305453	55306516	TCONS_00028071	−
chr9	88472600	88488125	TCONS_00016638+TCONS_00016038	+
chr12	102718465	102745141	TCONS_l2_00006386+TCONS_l2_00006387+TCONS_l2_00006388	−
chr21	37477179	37481988	TCONS_00028843+TCONS_00029007+TCONS_00029008	+
chr10	20886074	20898900	TCONS_00018683+TCONS_00018684+TCONS_00018133	+
chr4	6315486	6319667	TCONS_00007969	+
chr8	39961636	39984685	TCONS_00014682+TCONS_00014683	+
chr7	39803198	39807261	TCONS_00013783	−
chr1	83001858	83021203	TCONS_00001536	−
chr5	178365678	178368084	TCONS_00009823	−
chr2	105104914	105136671	TCONS_00003350	−
chr13	111635654	111642911	TCONS_l2_00007471	+
chr13	90203039	90204606	TCONS_00021869	+
chr19	41324357	41332324	TCONS_l2_00013113	−
chr13	19582399	19586774	LINC00442	+
chr3	184222901	184226187	TCONS_00005677	+
chr1	108593042	108594976	TCONS_00001088	+
chr9	42275939	42298165	TCONS_l2_00029721	+
chr2	31935275	31990384	TCONS_00004196	−
chr15	39592647	39603018	TCONS_00023638+TCONS_00024080	−
chr2	102604265	102605462	TCONS_00003799+TCONS_00003800	+
chr9	65467783	65469026	TCONS_00015682	+
chrX	281389	285848	TCONS_l2_00030415+TCONS_l2_00030416+TCONS_l2_00030417+TCONS_l2_00030418	−
chr20	22753435	22754566	TCONS_00028129	+
chr2	176986339	176987094	TCONS_00003443	−
chr4	113614024	113627873	TCONS_l2_00021247	−
chr17	15469376	15472238	TCONS_00025574	−
chr12	115134050	115136892	TCONS_00020933+TCONS_00020934	−
chr15	85857857	85873522	TCONS_00023765+TCONS_00024194	−
chr11	76493141	76494238	TCONS_00019714+TCONS_00020102	−
chr15	26696898	26704722	TCONS_00023594+TCONS_00023595	−
chr7	79875725	79941772	TCONS_00013202	−
chr10	5276321	5305165	TCONS_00017775	+

chr21	34216677	34222244	TCONS_00028917	−
chr6	40346163	40347631	TDRG1	+
chr5	95169505	95188626	TCONS_l2_00023026+TCONS_l2_00023027	−
chr1	77685227	77686969	TCONS_00000997+TCONS_00000998	+
chr11	3647690	3658789	TRPC2	+
chr6	230932	237179	TCONS_00011631	+
chr1	180100864	180103986	TCONS_00001210	+
chr20	47935316	47948551	TCONS_00028210	+
chr16	60855592	60856769	TCONS_00024427	+
chr4	101699739	101712392	TCONS_00007799	−
chr20	4409712	4412358	TCONS_00027875+TCONS_00027990	−
chr1	183408443	183412805	TCONS_00001746	−
chr15	84860600	84878025	LOC440300	+
chr19	52097833	52106091	TCONS_l2_00012645	+
chr7	155213901	155249794	TCONS_00013277	−
chr14	94830363	94833021	TCONS_l2_00008202	−
chr2	114799666	114800908	TCONS_00004415	−
chr1	85691288	85710928	TCONS_00001011	+
chr7	90996450	90997593	TCONS_00014352	−
chr8	7958282	8042348	TCONS_l2_00028082+TCONS_l2_00028083+TCONS_ l2_00028084	−
chr2	238135863	238166293	TCONS_00004062+TCONS_00005038+TCONS_00005037	+
chr14	62022962	62044967	TCONS_00022763+TCONS_00023118+TCONS_00023117 +TCONS_00022764+TCONS_00023119+TCONS_0002276 5+TCONS_00022412	−
chr2	85142960	85143829	TCONS_l2_00013742	+
chr11	9137687	9142948	TCONS_00019570+TCONS_00019571	−
chr3	86708206	86771837	TCONS_00006120+TCONS_00006906+TCONS_000 06907+TCONS_00006122+TCONS_00006121+TCO NS_00006908	+
chr2	23957295	23958476	TCONS_00004183	−
chr20	25936453	25949923	TCONS_00027863	+
chr15	86623086	86668723	TCONS_00024001	+
chr20	11247307	11254031	LOC339593	−
chr2	104741726	104763687	TCONS_00003348	−
chr2	238130858	238134992	TCONS_00004061	+
chr1	224133092	224222680	TCONS_l2_00001800+TCONS_l2_00001801+TCONS_ l2_00001804+TCONS_l2_00001802+TCONS_ l2_00001805+TCONS_l2_00001806+TCONS_ l2_00001807+TCONS_l2_00001808+TCONS_l2_000018 09+TCONS_00001838+TCONS_l2_00001811+TCONS_ l2_00001812+TCONS_l2_00001813	−
chr17	72970796	72972374	TCONS_00025490	+
chr4	127149786	127164167	TCONS_00008570	−
chr4	38603968	38609799	TCONS_l2_00021108+TCONS_l2_00021109+TCONS_ l2_00021110+TCONS_l2_00021111	−
chrX	73164159	73290217	JPX	+
chrX	101032469	101035614	TCONS_l2_00030558+TCONS_l2_00030559+TCONS_ l2_00030560	−
chr18	12739784	12749484	TCONS_00026679	−

chr12	5641076	5643050	TCONS_00020691	−
chr12	2815382	2835915	TCONS_l2_00006048	−
chr12	120807243	120809491	TCONS_l2_00006603	+
chr18	71615131	71625153	TCONS_00026366	+
chr5	172722154	172724670	TCONS_00010176	+
chr17	20422625	20432422	TCONS_l2_00011633+TCONS_l2_00011137+TCONS_l2_00011138	−
chr12	26287897	26334216	TCONS_00020184	+
chr10	130833655	130834943	TCONS_00018641	−
chr3	127400341	127404145	TCONS_00006651	−
chr7	26266058	26284739	TCONS_00013744	−
chr3	193551438	193555388	TCONS_00006739	−
chr3	193965437	193976672	TCONS_00005698+TCONS_00006358	+
chr7	156016154	156028784	TCONS_00014238+TCONS_00013652	+
chr21	18126838	18188007	TCONS_00028872	−
chr21	45619132	45621216	TCONS_00029175	−
chr10	3985205	4006403	TCONS_00018089+TCONS_00018090	+
chr6	86354449	86356991	TCONS_00012219	−
chr4	120559333	120578304	TCONS_00008562	−
chr15	90646498	90676937	TCONS_00024004+TCONS_00023503	+
chr19	39504600	39505190	TCONS_00027754	−
chr5	166331944	166334981	TCONS_00010544	−
chr16	10608824	10619948	TCONS_00024576	−
chr17	63074409	63087681	TCONS_00025730	−
chr1	16944751	16957401	CROCCP2	−
chr7	52492897	52493547	TCONS_00013446	+
chr21	47874902	47878593	TCONS_l2_00017538+TCONS_l2_00017315	−
chr3	174156363	174455769	TCONS_00005438+TCONS_00005440+TCONS_00006311+TCONS_00006312+TCONS_00006313+TCONS_00006314+TCONS_00006315	+
chr1	176176784	176198485	TCONS_00000339+TCONS_00001205	+
chr3	64928165	64973762	TCONS_00007180+TCONS_00007181+TCONS_00007182	−
chr12	40579811	40617605	TCONS_00020243	−
chrY	23300674	23332061	TCONS_00017655+TCONS_00017640+TCONS_00017656+TCONS_00017641+TCONS_00017642	+
chr22	32904729	32905176	TCONS_00029899	−
chr8	65716461	65730447	TCONS_00014740	+
chr9	137519676	137521252	TCONS_00015772	+
chr11	115794359	115800208	TCONS_00019481+TCONS_00019998	+
chr8	98630165	98630871	TCONS_00015110	−
chr15	57611129	57617222	TCONS_00023426	+
chr16	88277183	88279261	TCONS_00024512	+
chr19	56717368	56718434	TCONS_l2_00012725	+
chr1	52016846	52032193	TCONS_l2_00001232	−
chr17	35078783	35080651	TCONS_00025370	+
chr15	74200491	74202126	TCONS_l2_00009193	−
chr5	180846763	180848396	TCONS_00009271	+
chr2	10702421	10706471	TCONS_00002818	+

chrX	13353360	13359944	LOC100133123	+
chr12	34190307	34209704	TCONS_00020375	+
chr4	180092018	180227936	TCONS_00008353	+
chr3	59450056	59496404	TCONS_00005780	−
chrY	27209230	27246039	TTTY4C	−
chr1	56700768	56703535	TCONS_00002040+TCONS_00000954	+
chr6	20331468	20334683	TCONS_00012677+TCONS_00012106	−
chr20	22490753	22504856	TCONS_00028332+TCONS_00028633+TCONS_00028333 +TCONS_00028634+TCONS_00028635	−
chr4	105828563	105891241	TCONS_00007585	+
chr12	9492506	9493304	TCONS_00020316	+
chr2	52925300	52926173	TCONS_00003675	+
chr6	93432721	93433547	TCONS_l2_00025436	−
chr19	53471504	53496784	ZNF702P	−
chr7	123284404	123284959	TCONS_00014198	+
chr8	144499834	144501963	TCONS_00014882	+
chr15	53084847	53087549	TCONS_00023411	+
chr7	50314386	50315218	TCONS_00013169	−
chr10	46737613	48952629	BMS1P5	−
chr2	175197325	175198448	TCONS_00004501	−
chr9	43184317	43189410	TCONS_l2_00029287+TCONS_l2_00029288+TCONS_ l2_00029289	−
chr16	29437125	29443132	TCONS_l2_00010079+TCONS_l2_00010080+TCONS_ l2_00010081	−
chr4	190197916	190198838	TCONS_00008385+TCONS_00008386+TCONS_00008387 +TCONS_00008982+TCONS_00008983	+
chr6	82647456	82659629	TCONS_00011875	+
chr22	24385938	24401899	GSTTP2	−
chr8	12219528	12268510	FAM66A	+
chr21	22939084	22987758	TCONS_00028804	+
chr12	19824093	19826192	TCONS_00020354	+
chr2	51559800	51571385	TCONS_00005147+TCONS_00005148+TCONS_00005149	−
chr6	79519432	79523020	TCONS_00011522	−
chr8	2387147	2415857	TCONS_00014912	−
chr7	89748714	89754914	DPY19L2P4	+
chr16	73982488	74028605	TCONS_00024726	−
chrX	20967707	20971852	TCONS_00017301	−
chr4	162021047	162046045	TCONS_00008636	−
chr10	103461754	103464357	TCONS_00018589	−
chr1	146473499	146475178	TCONS_00000636+TCONS_00000637	−
chr2	177588996	177684038	TCONS_l2_00016030+TCONS_l2_00016031+TCONS_ l2_00016032	−
chr6	53794739	53862463	TCONS_l2_00024264+TCONS_l2_00025166	+
chr17	15179194	15182832	TCONS_00025572+TCONS_00025212	−
chr8	135768594	135780922	TCONS_00015367+TCONS_00015368+TCONS_00015369 +TCONS_00015370+TCONS_00014865	+
chr2	7865833	7870836	TCONS_00004689+TCONS_00002811	+
chr13	78553901	78571978	TCONS_00021518+TCONS_00022056+TCONS_00022057 +TCONS_00022299+TCONS_00021672	−

chr21	25261114	25262857	TCONS_00028888	−
chr14	103673868	103675204	TCONS_l2_00007928	+
chr2	108665656	108669211	TCONS_00003364	−
chr1	157202021	157206926	TCONS_00000648	−
chr7	31023666	31024824	TCONS_00013407	+
chr8	111991098	112039752	TCONS_00015135+TCONS_00015136+TCONS_00015488 +TCONS_00015489	−
chr5	42994639	42997566	TCONS_00009935	+
chr1	182681805	182683733	TCONS_00001216+TCONS_00000347	+
chr16	88227739	88228689	TCONS_00024511	+
chr1	181159607	181160285	TCONS_00000694	−
chr21	31456109	31462294	TCONS_00028991	+
chr2	107201803	107277026	TCONS_00003812	+
chr6	46182812	46184920	TCONS_00011821	+
chr6	44184676	44185959	TCONS_l2_00024225	+
chr1	161259151	161262269	TCONS_00001705	−
chr12	10393135	10412929	TCONS_00020330	+
chr3	16276632	16283253	TCONS_00006792	+
chr5	15192248	15266650	TCONS_00009609	−
chr3	18486516	18572768	TCONS_l2_00019762+TCONS_l2_00018460+TCONS_ l2_00018461+TCONS_l2_00018462+TCONS_ l2_00019763+TCONS_l2_00018463+TCONS_l2_00018464	+
chr15	25247918	25281705	TCONS_00023878+TCONS_00023879+TCONS_00023880 +TCONS_00023881+TCONS_00023882	+
chr1	192685463	192685814	TCONS_l2_00000739	+
chr17	79604197	79606203	TCONS_00025132	+
chr7	98283482	98286896	TCONS_00013900	−
chr6	156135179	156139135	TCONS_00012339	−
chr3	5297220	5298446	TCONS_00005729	−
chr3	40503867	40508645	TCONS_00005763	−
chr15	89897150	89901885	TCONS_00023778	−
chr15	84748939	84795353	EFTUD1P1	+
chr3	164078868	164137295	TCONS_00006292	+
chr1	173143439	173144642	TCONS_00000683	−
chrX	100759568	100764657	TCONS_00017353	−
chrX	20004935	20007897	LOC729609	−
chr5	139541538	139553608	TCONS_00009260+TCONS_00010109+TCONS_00010110+ TCONS_00010803+TCONS_00010801+TCONS_00010800 +TCONS_00010799+TCONS_00010798+TCONS_00010111 +TCONS_00010112+TCONS_00010805+TCONS_00010113	+
chr16	24258414	24259194	TCONS_00024352	+
chr1	45282505	45283445	TCONS_00002016	+
chr14	77426018	77432564	TCONS_00022562+TCONS_00022563	+
chr3	134042592	134059334	TCONS_00006665+TCONS_00007270	−
chr1	224179798	224198290	TCONS_l2_00000846+TCONS_l2_00000847+TCONS_ l2_00000848+TCONS_l2_00000849+TCONS_ l2_00000850+TCONS_l2_00000851+TCONS_ l2_00002321+TCONS_l2_00002322	+
chr16	88143566	88148142	TCONS_00024780	−

chr8	135862178	135894142	TCONS_00014548	+
chr3	36312168	36351498	TCONS_00005987	+
chr19	47742378	47747476	TCONS_00027385	−
chr9	70941645	70945547	TCONS_00016601+TCONS_00016602+TCONS_00016603+TCONS_00016604+TCONS_00016605+TCONS_00015688+TCONS_00015689	+
chr16	22445951	22447406	TCONS_00024348	+
chr2	159695270	159713813	TCONS_l2_00014046	+
chr16	62145898	62164547	TCONS_00024430	+
chr7	142539828	142552743	TCONS_00013988	−
chr19	36198272	36199158	TCONS_00026831	+
chr1	190447356	190463427	TCONS_00001227+TCONS_00000060+TCONS_00002206+TCONS_00001228+TCONS_00000356+TCONS_00001229+TCONS_00001230+TCONS_00000357+TCONS_00000358	+
chr17	38688846	38695010	TCONS_00025633+TCONS_00025634	−
chr21	26258990	26275070	TCONS_00028892	−
chr1	149238888	149265510	TCONS_l2_00000590+TCONS_l2_00002188+TCONS_l2_00000591+TCONS_l2_00002189+TCONS_l2_00002190+TCONS_l2_00002191+TCONS_l2_00002192+TCONS_l2_00002193+TCONS_l2_00000592	+
chr17	14276848	14324343	TCONS_00025313+TCONS_00025157	+
chr12	128662789	128676029	TCONS_00021454+TCONS_00020994+TCONS_00020995+TCONS_00020996	−
chr3	66831272	66874793	TCONS_00006527	−
chr6	40685387	40688616	TCONS_00011812	+
chr17	59470733	59477096	TCONS_00025721+TCONS_00025722+TCONS_00025723+TCONS_00025724+TCONS_00025725	−
chr7	95069323	95071713	TCONS_00013537	+
chr1	232295837	232321389	TCONS_00000417	+
chr6	8342170	8343254	TCONS_00011444	−
chr9	108870332	109367180	TCONS_00015582+TCONS_l2_00029827+TCONS_l2_00028969+TCONS_l2_00029828+TCONS_l2_00028970	+
chr1	175172364	175173985	TCONS_00000690	−
chrX	62646439	62780873	LOC92249	−
chr2	75572395	75575381	TCONS_00003744	+
chr2	388412	416885	TCONS_00002788	+
chr6	164529652	164530726	TCONS_00011611	−
chr13	50210642	50214262	TCONS_00022282	−
chr8	127630408	127634583	TCONS_00015497	−
chr13	27947485	27948503	TCONS_00021533	+
chr17	36443036	36452228	TCONS_00025619	−
chrX	102906453	102926745	TCONS_l2_00030566	−
chr2	16383698	16385494	TCONS_00002833	+
chr9	92731704	92767420	TCONS_00016644+TCONS_00016062+TCONS_00015707+TCONS_00016645	+
chr4	108908048	108910629	TCONS_00008546	−
chr10	81664654	81691557	LOC100288974	+
chrY	22681834	22686319	TCONS_00017652	−
chr17	10781800	10784191	TCONS_00025309	+

chr9	40293022	40324812	TCONS_l2_00028735+TCONS_l2_00028736+TCONS_l2_00028737+TCONS_l2_00028738+TCONS_l2_00028739	+
chr5	78623523	78631046	TCONS_00010383	−
chr15	40616037	40619095	TCONS_l2_00008581+TCONS_l2_00008582+TCONS_l2_00008583	+
chrX	45364633	45489447	TCONS_00016983+TCONS_00016932+TCONS_00016984+TCONS_00017158	+
chrX	37748183	37758208	TCONS_00017415+TCONS_00017416	+
chr13	91508606	91510193	TCONS_00022082	−
chr19	54278778	54279108	TCONS_l2_00012696	+
chr11	10804833	10813847	TCONS_00019231	+
chr21	40218171	40220568	TCONS_00028847	+
chrX	154576450	154603823	TMLHE-AS1	−
chr2	130626766	130635126	TCONS_00003402	−
chr9	74872252	74872699	TCONS_00015691	+
chr15	61890713	61897440	TCONS_00023230	+
chr5	163875251	163896926	TCONS_00011079+TCONS_00011080	−
chr11	46181249	46183116	TCONS_00019299	+
chr8	54427731	54436491	TCONS_00014514	−
chrX	130115564	130192120	TCONS_00017090+TCONS_00017091	−
chr17	17577340	17581002	TCONS_00025215	−
chr7	62809448	62812152	LOC100287704	+
chr2	183536804	183539242	TCONS_00005286+TCONS_00004513+TCONS_00004514	−
chr2	238037370	238039886	TCONS_00004618	−
chr1	98676267	98738214	LOC729987	+
chr17	48580773	48585689	TCONS_00025238	−
chr4	156127681	156129583	TCONS_00007886	−
chr5	65594231	65622820	TCONS_00010702+TCONS_00010703+TCONS_00009982	+
chr15	91203465	91208176	TCONS_00023239	+
chr8	31033695	31034708	TCONS_00014664	+
chr15	82585621	82924242	LOC390660	+
chr1	8268732	8275270	TCONS_00000150	+
chr17	16925489	16926546	TCONS_00025321	+
chr11	2012326	2014699	TCONS_00019135	+
chr5	53072051	53115516	TCONS_l2_00022896+TCONS_l2_00022897+TCONS_l2_00023738+TCONS_l2_00023739	−
chr4	23372017	23394795	TCONS_00008447	−
chr17	32517544	32523223	TCONS_00025609	−
chr9	89563613	89616948	LOC100506834	+
chr22	24002052	24026054	TCONS_00029778	+
chr2	42615499	42652207	TCONS_00004219+TCONS_00004220+TCONS_00004221+TCONS_00005137	−
chr7	130565751	130598069	LOC646329	−
chr7	156774470	156774876	TCONS_00013654	+
chr3	125619686	125628844	TCONS_l2_00018805	+
chr19	13710595	13734962	TCONS_l2_00012876	−
chr7	56682442	56685650	TCONS_00013818+TCONS_00013819+TCONS_00014308	−
chr15	95562063	95613962	TCONS_00023528	+

chr16	74299394	74302955	TCONS_00024467	+
chr1	178675709	178676861	TCONS_00001209	+
chr21	46654267	46678645	TCONS_00028775+TCONS_00029245	+
chr3	129931663	129992649	COL6A4P2	+
chr7	100946270	100947840	TCONS_l2_00026141	+
chr8	78290938	78363014	TCONS_00014772+TCONS_00014773	+
chr18	74327169	74337889	TCONS_00026218+TCONS_00026219+TCONS_00026652 +TCONS_00026378	+
chr20	61416930	61423490	TCONS_00028465+TCONS_00028725	−
chr5	108804805	108812483	TCONS_00010054+TCONS_00010055	+
chr11	61883944	61891289	TCONS_00019646	−
chr7	34249138	34295955	TCONS_00012990	+
chr16	33110462	33123475	TCONS_l2_00010459+TCONS_l2_00010116+TCONS_ l2_00010117+TCONS_l2_00010118+TCONS_ l2_00010119+TCONS_l2_00010120	−
chr5	72733584	72734462	TCONS_00009997	+
chr9	92503174	92504508	TCONS_00015841	−
chr6	170470885	170475667	TCONS_00012377	−
chr3	71834486	71837924	TCONS_00006091+TCONS_00006888+TCONS_00006887 +TCONS_00006886+TCONS_00006092	+
chr16	89979190	89983349	TCONS_l2_00010376+TCONS_l2_00010377+TCONS_ l2_00010378+TCONS_l2_00010379+TCONS_ l2_00010380+TCONS_l2_00010381	−
chr4	32441610	32561930	TCONS_00008457	−
chr21	30367200	30372267	TCONS_l2_00017194+TCONS_l2_00017195+TCONS_ l2_00017196	−
chr2	236086540	236087549	TCONS_00003129	+
chr1	11824462	11826573	TCONS_00000038	+
chr20	62030714	62034595	TCONS_00028265+TCONS_00028266+TCONS_00028267 +TCONS_00028268	+
chr6	170481454	170492362	TCONS_00012378+TCONS_l2_00025054+TCONS_ l2_00025055+TCONS_l2_00025056+TCONS_l2_00025057	−
chrY	6182796	6184282	TCONS_00017621	−
chr4	152182570	152184119	TCONS_l2_00021752	+
chr1	187412738	187446354	TCONS_00000351+TCONS_00000352	+
chr1	200661853	200663035	TCONS_00001234	+
chr4	144207446	144251011	TCONS_00007644	+
chr11	112035955	112036888	TCONS_00019760	−
chr8	102165619	102166287	TCONS_00014811	+
chr2	221670539	221718382	TCONS_00004594+TCONS_00005339+TCONS_00004595	−
chr12	132148446	132149209	TCONS_00021260	+
chr5	172243602	172252561	TCONS_00010833+TCONS_00010174	+
chr6	164348098	164429693	TCONS_00012013	+
chr19	36077048	36078334	TCONS_00027005	+
chr2	21267101	21268881	TCONS_00003597+TCONS_00003598	+
chr21	23381263	23470778	TCONS_l2_00017448+TCONS_l2_00017449+TCONS_ l2_00017169+TCONS_l2_00017450+TCONS_l2_00017170	−
chr3	112362185	112368458	TCONS_00006616	−
chr6	1308821	1311276	TCONS_00012039+TCONS_00012040	−

chr2	112365418	112370095	TCONS_00002979	+
chr6	28555155	28559524	TCONS_00011167	+
chr9	93063184	93195787	TCONS_00015842+TCONS_00015843+TCONS_00016826	−
chr1	167498603	167502878	TCONS_00001714	−
chr2	18825464	19125152	TCONS_00003590+TCONS_00002841	+
chr2	208091673	208093032	TCONS_00003086	+
chr1	230774214	230776446	TCONS_00001864	−
chr22	20970518	21011209	TCONS_l2_00018332+TCONS_l2_00017900+TCONS_ l2_00017901+TCONS_l2_00018333+TCONS_l2_00017902	−
chr9	76986447	76998446	TCONS_00015695	+
chr8	141646243	141648531	TCONS_00014875	+
chr17	5372397	5373687	TCONS_00025290	+
chr2	171626760	171627337	TCONS_00004944	+
chr1	117338494	117349012	TCONS_00001640	−
chr5	177366560	177410055	TCONS_l2_00022658+TCONS_l2_00022659+TCONS_ l2_00022661+TCONS_l2_00023604+TCONS_ l2_00022662+TCONS_l2_00022663+TCONS_ l2_00022664+TCONS_l2_00022665+TCONS_ l2_00023605+TCONS_l2_00023606+TCONS_ l2_00023607+TCONS_l2_00022666+TCONS_ l2_00022667+TCONS_l2_00023608+TCONS_ l2_00023609+TCONS_l2_00022668+TCONS_00010852+T CONS_00010853	+
chr2	3579405	3584463	TCONS_00002736+TCONS_00003157+TCONS_00003158 +TCONS_00003159+TCONS_00003160	−
chr4	104472084	104480284	TCONS_00007806	−
chr2	229554325	229579362	TCONS_00004050	+
chr11	573808	575885	LOC143666	−
chr4	121560245	121571951	TCONS_00007606+TCONS_00007607	+
chr1	227976988	227979782	TCONS_00001315	+
chr18	77341499	77346761	TCONS_00026383	+
chr16	73092703	73093074	TCONS_00024725	−
chr6	37517780	37518636	TCONS_00011300	+
chr5	67734922	67740786	TCONS_00009685	−
chr1	54440301	54441604	TCONS_l2_00001240+TCONS_l2_00001241	−
chr13	110675351	110707299	TCONS_00021900+TCONS_00021598	+
chr4	95038943	95128707	TCONS_l2_00021928	−
chr4	33517099	33522181	TCONS_00008036	+
chr2	161777345	161850979	TCONS_00004479	−
chr2	136770035	136774067	TCONS_00003007	+
chr4	171900344	171916283	TCONS_l2_00020874+TCONS_l2_00020875	+
chr6	1321763	1335838	TCONS_00012639+TCONS_00012041+TCONS_00012640	−
chr13	64402902	64418307	TCONS_l2_00007566+TCONS_l2_00007233+TCONS_ l2_00007234+TCONS_l2_00007235+TCONS_ l2_00007567+TCONS_l2_00007236+TCONS_ l2_00007237+TCONS_l2_00007238+TCONS_ l2_00007239+TCONS_l2_00007240+TCONS_ l2_00007241+TCONS_l2_00007242	−
chr9	46661242	46662837	TCONS_00015819+TCONS_00015820	−
chrX	16599800	16601770	TCONS_00017142	+

chr2	6775745	6791019	TCONS_00004114+TCONS_00005069+TCONS_00004115+TCONS_00005070+TCONS_00003178+TCONS_00005071+TCONS_00003179+TCONS_00003180+TCONS_00003181+TCONS_00004116	−
chr7	19393157	19618229	TCONS_l2_00027062+TCONS_l2_00025700	+
chr6	159990581	160016234	TCONS_00012347+TCONS_00012818+TCONS_00011239+TCONS_00012348+TCONS_00012349	−
chr1	89873238	89890493	GBP1P1	+
chr2	206853275	206854321	TCONS_00004561	−
chr22	36948212	36948747	TCONS_00029420	+
chr8	102300022	102305710	TCONS_00014812	+
chr10	6335137	6335689	TCONS_l2_00003387	−
chr2	82210396	82232824	TCONS_00003329	−
chr17	30728988	30738705	TCONS_00025349	+
chr9	138096568	138129443	TCONS_00015893	−
chr17	15663681	15669096	TCONS_l2_00011108+TCONS_l2_00011109+TCONS_l2_00011622	−
chr1	43323293	43354460	TCONS_l2_00002036	+
chr4	157033788	157065044	TCONS_00008628+TCONS_00008629+TCONS_00008630+TCONS_00009149+TCONS_00009150+TCONS_00008631	−
chr22	44736116	44740506	TCONS_00029613	+
chr15	41578207	41598741	TCONS_00023648+TCONS_00024099	−
chrY	9555262	9558905	TTTY21B	−
chr5	147618054	147646815	TCONS_l2_00022568+TCONS_l2_00023566	+
chr15	65016565	65016901	TCONS_00023442	+
chr2	151422644	151432787	TCONS_l2_00014029+TCONS_l2_00015519+TCONS_l2_00014030	+
chr2	192479791	192502235	TCONS_00004969+TCONS_00003973	+
chr2	176762064	176768960	TCONS_00003055	+
chr22	46409178	46410258	TCONS_00029727	−
chr12	116198115	116207965	TCONS_00020578	+
chr18	75683255	75692482	TCONS_00026222	−
chr21	45587386	45588356	TCONS_00029313+TCONS_00029172	−
chr2	66124756	66126649	TCONS_00002915	+
chr6	138051307	138060120	TCONS_00011570	−
chr1	188838608	188856565	TCONS_00000702	−
chrX	100671835	100672797	TCONS_00017209	+
chr19	6656385	6662832	TCONS_00026873	+
chr8	38411282	38414039	TCONS_00014979	−
chr7	35771347	35840234	TCONS_l2_00026506+TCONS_l2_00026507+TCONS_l2_00026508+TCONS_l2_00026509+TCONS_l2_00027330+TCONS_l2_00027331+TCONS_l2_00027332	−
chr2	5413886	5434651	TCONS_00003553	+
chr2	12246710	12271674	TCONS_00002825	+
chr20	62959556	62961294	TCONS_00028271	+
chr5	95179752	95185762	TCONS_00010747+TCONS_00010748+TCONS_00010749+TCONS_00010750+TCONS_00010751	+
chr2	30911934	30920389	TCONS_00003623	+
chr9	35406752	35483026	ATP8B5P	+

chr13	35270892	35273146	TCONS_00021750	+
chr21	14371997	14389013	TCONS_00028865	−
chr13	91779867	91863952	LINC00379	−
chrY	6274285	6296485	TTTY2	−
chrX	134229015	134232733	LINC00087	−
chr1	223354486	223361496	TCONS_00000409	+
chr14	27244062	27291394	TCONS_00022689	−
chr3	103646039	103730568	TCONS_00005423	+
chr18	70535041	70548634	TCONS_00026361+TCONS_00026362+TCONS_00026639 +TCONS_00026363+TCONS_00026364	+
chr6	168185219	168197539	C6orf123	−
chr9	41952399	41955076	MGC21881	−
chr7	108962377	108966372	TCONS_00013223	−
chr6	91323087	91344320	TCONS_00011887	+
chr9	92500998	92523200	TCONS_00016060+TCONS_00015706+TCONS_00016061	+
chr6	6891670	6899654	TCONS_00011670	+
chr1	212640158	212641085	TCONS_00000727	−
chr5	148442880	148489350	TCONS_l2_00023567+TCONS_l2_00022571+TCONS_ l2_00023568+TCONS_l2_00023569+TCONS_ l2_00023570+TCONS_l2_00023571+TCONS_l2_00023572	+
chr11	69290399	69291552	TCONS_00020094	−
chr12	90970004	90987585	TCONS_00020513	+
chr3	39418964	39422626	TCONS_00005994	+
chr2	58503012	58523683	TCONS_00002895+TCONS_00003696	+
chr5	95583480	95614455	TCONS_00009465	+
chr2	47079159	47080635	TCONS_00003277	−
chr6	11990575	12001446	TCONS_l2_00024614+TCONS_l2_00025337	−
chr5	116256539	116260291	TCONS_00010444	−
chr9	126101093	126102106	TCONS_00016476	−
chr4	24667011	24670951	TCONS_00008011	+
chr17	48292522	48294117	TCONS_00025692	−
chr11	28535561	28548491	TCONS_00019266	+
chr6	40825508	40827706	TCONS_l2_00024745	−
chrX	44494093	44495332	TCONS_l2_00030185	+
chr16	72276919	72283140	TCONS_00024719	−
chr13	90129862	90152763	TCONS_00021682	−
chr8	55778401	55783713	TCONS_l2_00027767	+
chr6	72185562	72189187	TCONS_00011855	+
chr13	19620293	19692457	TCONS_l2_00007482+TCONS_l2_00007483+TCONS_ l2_00007050+TCONS_l2_00007051	−
chr3	184491164	184494210	TCONS_00006338	+
chr21	16439391	16440158	TCONS_00028794	+
chr19	53727162	53727906	TCONS_00027618+TCONS_00027619+TCONS_00027620 +TCONS_00027621	+
chr10	119875117	119878421	TCONS_00019038	−
chr4	138533727	138672165	TCONS_00008581+TCONS_00007847+TCONS_00009122 +TCONS_00007848+TCONS_00008582+TCONS_0000912 3+TCONS_00009124	−
chr1	30613110	30614454	TCONS_00000175	+

chr18	56327109	56327937	TCONS_00026344	+
chr8	9045822	9060629	TCONS_00014604+TCONS_00014482+TCONS_00014605+TCONS_00014607	+
chr6	52555628	52557865	TCONS_l2_00024786	−
chr17	30018982	30023945	TCONS_00025347	+
chrX	38729909	38745299	TCONS_00017153	+
chr5	111992123	112018667	TCONS_00009753	−
chr22	20330726	20331888	TCONS_00029446	−
chr9	65734404	65782814	TCONS_00016326+TCONS_00016773+TCONS_00016774+TCONS_00016775+TCONS_00016327	−
chr7	104581510	104602779	TCONS_00013054+TCONS_00012887	+
chr6	28864307	28865099	TCONS_00011278	+
chrX	1886240	1887669	TCONS_00016961	+
chr2	20263617	20306590	TCONS_00003223	−
chr19	53812504	53819086	TCONS_l2_00012693	+
chr12	58325232	58329947	LOC100506844	−
chr15	20100335	20145193	TCONS_00023286	+
chr5	103716257	103716740	TCONS_00009744	−
chr10	112305413	112307703	TCONS_00018333+TCONS_00018334	+
chr1	185339834	185344123	TCONS_00001221	+
chr10	128102438	128110448	LINC00601	−
chr4	152720585	152754128	TCONS_00008289+TCONS_00007659	+
chr15	68126648	68131217	TCONS_00023281	−
chr19	32696967	32707388	TCONS_00026993	+
chr4	147878590	147896609	TCONS_00007651+TCONS_00007652	+
chr20	51039020	51041964	TCONS_00028704	−
chr4	106924474	106943635	TCONS_00007810	−
chr16	84981784	84984093	TCONS_00025076+TCONS_00024747+TCONS_00025077	−
chr4	66734676	66744701	TCONS_00008095	+
chr8	47722340	47730705	TCONS_00014998	−
chr8	77396211	77403443	TCONS_00015043	−
chr10	37668110	37669239	TCONS_00018162	+
chr4	133512244	133599658	TCONS_l2_00021715	+
chrX	130836678	130964671	LOC286467	−
chr1	224396449	224400981	TCONS_00000742+TCONS_00000743	−
chr7	27240040	27246130	HOTTIP	+
chr1	48727015	48730430	TCONS_00001489	−
chr3	106631985	106646828	TCONS_00006586+TCONS_00006587	−
chr3	125934371	125966235	TCONS_00006200	+
chr13	71589273	71742549	LINC00348	+
chr19	52282890	52284578	TCONS_00027397	−
chr16	89225628	89230083	LINC00304	+
chr21	40241911	40328392	TCONS_l2_00017229+TCONS_l2_00017230+TCONS_l2_00017231+TCONS_l2_00017232+TCONS_l2_00017233+TCONS_l2_00017504+TCONS_l2_00017505+TCONS_l2_00017234	−
chr17	33558469	33569908	TCONS_00025221+TCONS_00025612+TCONS_00025613	−
chr13	30727523	30743424	TCONS_l2_00006770+TCONS_l2_00006771+TCONS_l2_00007378	+

chr10	48987630	49018413	TCONS_00018506+TCONS_00017975+TCONS_00017976	−
chr17	65038519	65040458	TCONS_00025731+TCONS_00025732+TCONS_00025733	−
chr2	28646221	28646862	TCONS_00005118	−
chr13	80140837	80145923	TCONS_00021852	+
chr3	149756947	149758445	TCONS_00006267	+
chr5	68334345	68339648	TCONS_00009689	−
chr15	33491502	33496534	TCONS_00023343	+
chr12	68726572	68742427	TCONS_00021342	−
chr3	116958058	116963238	TCONS_00006164+TCONS_00006165	+
chr3	152368258	152457738	TCONS_00005626+TCONS_00007007+TCONS_00006276+TCONS_00006277+TCONS_00006278+TCONS_00007008+TCONS_00006279+TCONS_00007009	+
chr7	153406738	153413531	TCONS_00014012	−
chr5	78654497	78663947	TCONS_00010966	−
chr18	66817067	66832387	TCONS_00026354	+
chr6	20042903	20045304	TCONS_l2_00024643	−
chr10	121940742	122047981	TCONS_00017881+TCONS_00018356+TCONS_00018357+TCONS_00018838	+
chrX	139298187	139298956	TCONS_00017096	−
chr2	132889690	132893383	TCONS_l2_00014913	−
chr6_apd_hap1	1108577	1112186	TCONS_00029938	+
chr12	6281426	6282555	TCONS_00020285+TCONS_00020286	+
chr13	114459242	114459561	TCONS_00022130	−
chr16	59436484	59445573	TCONS_00024691	−
chr8	11203437	11204999	TCONS_l2_00028538	−
chr4	174615438	174620132	TCONS_00009167+TCONS_00008651	−
chr11	114250772	114251212	TCONS_00019479	+
chr21	15215454	15220685	C21orf15	−
chr8	96998341	97005331	TCONS_00014561	−
chr12	91299314	91312458	TCONS_00020868	−
chr16	87172049	87188136	TCONS_00024776	−
chr8	126761307	126762525	TCONS_00015163	−
chr14	71759472	71788653	TCONS_00022779+TCONS_00023124+TCONS_00022780+TCONS_00022781+TCONS_00022782+TCONS_00022783+TCONS_00022784+TCONS_00022785+TCONS_00023125+TCONS_00022786+TCONS_00023126	−
chr11	6677940	6678468	TCONS_00019214	+
chr17	26075314	26081148	TCONS_l2_00010642+TCONS_l2_00010643	+
chr9	140762365	140771749	TCONS_00016518+TCONS_00016519+TCONS_00016520	−
chr21	45621173	45622579	TCONS_00028855	+
chr2	241845860	241859705	TCONS_l2_00014321	+
chr1	33171296	33171923	TCONS_00001449	−
chr1	20525484	20529294	TCONS_00001952+TCONS_00000859	+
chrX	698300	698866	TCONS_l2_00030421	−
chr7	64520284	64541655	TCONS_l2_00026622+TCONS_l2_00027383+TCONS_l2_00026623	−
chr5	116366896	116374265	TCONS_00009489	+
chr16	52689323	52690584	TCONS_l2_00009761	+

chr7	158751572	158763083	TCONS_00013658	+
chr12	127359391	127373237	TCONS_00021252+TCONS_00020639+TCONS_00021253	+
chrX	147133553	147134430	TCONS_l2_00030383+TCONS_l2_00030384	+
chr22	27053446	27072440	MIAT	+
chr6	164092630	164195657	TCONS_00012012+TCONS_00011199+TCONS_00011410 +TCONS_00012625	+
chr22	49262582	49294198	LOC100128946	+
chr15	83876655	83896949	TCONS_00023269	+
chr20	17206104	17206710	TCONS_00028323	−
chr5	171209759	171222385	TCONS_l2_00022616+TCONS_l2_00022617	+
chr3	14431283	14432524	TCONS_00006447	−
chr10	36311805	36315277	TCONS_00018160	+
chr4	1282936	1283492	TCONS_00009003	−
chr7	63539167	63546016	TCONS_l2_00027127+TCONS_l2_00025931+TCONS_ l2_00025932	+
chr9	102337772	102339339	TCONS_00016439	−
chr13	44946024	44947669	TCONS_00021985	−
chr7	81157719	81191099	TCONS_l2_00027423	−
chr17	14737941	14803874	TCONS_l2_00010573+TCONS_l2_00010574+TCONS_ l2_00011523	+
chr11	116644105	116644921	TCONS_00019155	+
chr11	104209820	104229723	TCONS_00019457	+
chr2	15922618	15926006	TCONS_00003586	+
chr2	70316853	70330166	TCONS_l2_00015374+TCONS_l2_00013708	+
chr5	107035536	107052542	TCONS_00009750+TCONS_00009751	−
chr5	159003428	159012901	TCONS_00010155+TCONS_00010823	+
chr12	125510478	125513897	TCONS_00020260	−
chr16	80862435	80926493	TCONS_00024742+TCONS_00024276	−
chr10	10826402	10836877	SFTA1P	−
chr17	8123948	8127361	LINC00324	−
chr5	36372266	36397435	TCONS_00010302	−
chr2	75701579	75709705	TCONS_00002939	+
chr7	84277854	84509872	TCONS_l2_00026731+TCONS_l2_00026732	−
chr15	41576201	41591795	OIP5-AS1	+
chrX	134568606	134569791	TCONS_l2_00030611	−
chr9	72009222	72013753	TCONS_00016344	−
chr13	80446721	80492170	TCONS_00021500+TCONS_00021855	+
chr14	24096370	24096959	TCONS_00022925	+
chr11	11264297	11266408	TCONS_00019575	−
chr15	24591801	24603338	TCONS_00023307+TCONS_00023867	+
chr9	37904374	37905621	TCONS_00016575	+
chr7	73668832	73669929	TCONS_00014161+TCONS_00013496	+
chr11	9776317	9781080	LOC440028	−
chr20	46750577	46755530	TCONS_00028198	+
chr17	9703978	9712361	TCONS_l2_00011085	−
chr16	51349202	51352003	TCONS_00024397	+
chr18	41860743	41871727	TCONS_00026504+TCONS_00026505	−
chr15	28982729	29003508	WHAMMP2	+

chr10	73134945	73141151	TCONS_00018230	+
chr17	18315660	18317702	TCONS_00025855+TCONS_00025856+TCONS_00025324	+
chr1	95749339	95753228	TCONS_00001046	+
chr9	46844359	46845159	TCONS_00015681	+
chr9	138307020	138307423	TCONS_00016214	+
chr12	70099753	70132348	TCONS_00021356+TCONS_00021357+TCONS_00021358+TCONS_00020213+TCONS_00020214+TCONS_00021360	−
chr18	27227189	27229540	TCONS_00026309	+
chr13	91543208	91578851	LINC00410	−
chr5	173756615	173757506	TCONS_00009542	+
chr12	5088059	5090604	TCONS_00020280	+
chr4	27264128	27268876	TCONS_00007736	−
chr10	3507669	3545058	TCONS_00018411+TCONS_00018412+TCONS_00018413+TCONS_00018870+TCONS_00017903+TCONS_00018871	−
chr13	20753146	20759620	TCONS_l2_00007060	−
chr6	140177241	140198528	TCONS_00011576	−
chr10	89146179	89154374	TCONS_l2_00003696+TCONS_l2_00003697+TCONS_l2_00003698	−
chr2	199052526	199056245	TCONS_00003475+TCONS_00003476	−
chr1	191827837	191859359	TCONS_00000704+TCONS_00000705	−
chr5	116751208	116915439	LOC728342	+
chr5	141202480	141204895	TCONS_00010517	−
chr11	96530591	96531901	TCONS_00019449	+
chr6	137736806	137739549	TCONS_00011961	+
chr11	10952831	10955264	TCONS_00019237	+
chr12	49455911	49457826	TCONS_00020413	+
chr4	76279286	76287776	LOC441025	+
chr19	45248302	45250906	TCONS_00027767	−
chr6	169563323	169596780	TCONS_00012024	+
chr20	52556699	52559047	TCONS_00027955+TCONS_00028228	+
chr7	57712077	57713315	TCONS_l2_00026602	−
chr6	14474804	14512461	TCONS_00011700+TCONS_00011701+TCONS_00011702	+
chr16	54881151	54888858	TCONS_00024847	+
chrX	26177618	26180485	TCONS_l2_00030165	+
chr5	67819705	67825834	TCONS_00009413	+
chr6	30484043	30488506	TCONS_00011283+TCONS_00011774	+
chr15	48279856	48299630	TCONS_00023672+TCONS_00024119+TCONS_00023673	−
chr11	61236176	61241062	TCONS_00019312	+
chr10	105682668	105683570	TCONS_00018319	+
chr7	2477398	2488305	TCONS_00012865+TCONS_00013319+TCONS_00013320+TCONS_00013321+TCONS_00013322	+
chr4	188866788	188881648	TCONS_00008710	−
chr11	45602136	45605013	TCONS_00019294+TCONS_00019295+TCONS_00019296	+
chr4	104882773	104958080	TCONS_00007581+TCONS_00008182	+
chr6	68750266	68819883	TCONS_00011852+TCONS_00012511+TCONS_00011853+TCONS_00011321	+
chr3	116638699	116651245	TCONS_00006163+TCONS_00005428	+
chr1	147281915	147283468	TCONS_00001669	−

chr10	37591922	37636288	TCONS_l2_00002974+TCONS_l2_00002975+TCONS_l2_00002976+TCONS_l2_00002977+TCONS_l2_00003938+TCONS_l2_00003939+TCONS_l2_00003940	+
chr3	107732834	107758725	TCONS_00006601+TCONS_00006602	−
chr2	61368727	61372110	LOC339803	−
chr8	112711744	112712497	TCONS_00014832	+
chr17	49021465	49026111	TCONS_l2_00011572+TCONS_l2_00011573	+
chr6	115319522	115323156	TCONS_00011934	+
chr5	144818651	144869293	TCONS_00009797+TCONS_00010521+TCONS_00010522	−
chr6	21980814	21981701	TCONS_00011466	−
chr7	37532460	37570312	TCONS_00013414	+
chr1	234805269	234831900	TCONS_00000764	−
chr12	125636081	125638533	TCONS_00020615	+
chr7	30428962	30452157	TCONS_00013403+TCONS_00013404+TCONS_00013405+TCONS_00012988+TCONS_00014101+TCONS_00013406	+
chr17	55264509	55282436	TCONS_00025440	+
chr5	43024144	43024564	TCONS_00010677	+
chr9	111034533	111035976	TCONS_00016128	+
chr8	61545724	61548309	TCONS_00015021	−
chr19	45041045	45060150	CEACAM22P	−
chr2	9778901	9789568	TCONS_00002816	+
chr22	27063656	27068617	TCONS_00029347	−
chr6	83386875	83387744	TCONS_00012218	−
chr10	7487520	7513904	TCONS_00017921	−
chr5	89196481	89200725	TCONS_00009457	+
chr9	33719688	33722553	TCONS_00015660	+
chr3	155008977	155011965	TCONS_00005632	+
chr17	53690343	53725799	TCONS_00025436+TCONS_00025437	+
chr19	13781331	13789989	TCONS_00027198+TCONS_00027685+TCONS_00027686	−
chr3	107560509	107596915	LINC00635	−
chr2	139175857	139259268	TCONS_l2_00015981+TCONS_l2_00015982+TCONS_l2_00014934	−
chr2	9344859	9346598	TCONS_00004137+TCONS_00004138	−
chr6	23337938	23397703	TCONS_l2_00024089+TCONS_l2_00025105+TCONS_l2_00024090+TCONS_00012436+TCONS_00011734	+
chr12	9381129	9386803	A2MP1	−
chr2	56750280	56816630	TCONS_00003691	+
chr4	177567677	177592811	TCONS_00008665+TCONS_00008666	−
chr7	157258925	157292410	TCONS_00013104	+
chr2	92176819	92179491	TCONS_00004331	−
chr5	66928094	66993025	TCONS_l2_00023760+TCONS_l2_00022931+TCONS_l2_00022932	−
chr2	217732735	217733879	TCONS_00003109	+
chr11	119478390	119489008	TCONS_00019778	−
chr22	50981206	50983413	TCONS_00029745	−
chr19	19852148	19863638	TCONS_l2_00012909	−
chr10	81585658	81587358	LOC642361	+
chr1	116831856	116837107	TCONS_00002447	−

chr12	49183785	49184350	TCONS_00020412	+
chr7	25360921	25366434	TCONS_00013143	−
chr13	91739508	91741847	TCONS_00021685	−
chr13	20700739	20711260	TCONS_00021710	+
chr15	28954720	28972488	TCONS_l2_00008962+TCONS_l2_00008964	−
chrX	112859587	112861440	TCONS_00017082	−
chr22	23834998	23857412	TCONS_00029527+TCONS_00029528+TCONS_00029776 +TCONS_00029529+TCONS_00029369	+
chr1	21742815	21743867	TCONS_00000165	+
chr2	196313256	196343649	TCONS_00003072	+
chr6	156814360	156820905	TCONS_00011600+TCONS_00011601	−
chr20	453141	459285	TCONS_00028276	−
chr7	110231030	110242165	TCONS_00013563	+
chr4	146520214	146522021	TCONS_00008276+TCONS_00007648	+
chrY	16020072	16027914	TCONS_l2_00030903+TCONS_l2_00030904	+
chr7	51453002	51463804	TCONS_l2_00026562+TCONS_l2_00026563	−
chr17	10690548	10695151	TCONS_00025559	−
chr15	91061800	91068340	TCONS_00023504	+
chr6	27983256	28028206	TCONS_l2_00024679+TCONS_l2_00024680	−
chr17	80338989	80340511	TCONS_00025154	−
chr15	98173187	98196515	TCONS_l2_00009351	−
chr1	8138470	8185235	TCONS_00000832	+
chr4	11916291	11921312	TCONS_00007478	+
chr11	134582351	134585997	TCONS_00019550+TCONS_00019551	+
chr8	64297397	64330488	TCONS_00014519	−
chr3	135551402	135559458	TCONS_00006668	−
chr2	235591337	235632116	TCONS_00004057	+
chr7	100942894	100944551	TCONS_00013053	+
chr6	2637170	2640623	TCONS_00011252+TCONS_00012395	+
